# Prevalence of occupational moral injury and post-traumatic embitterment disorder: a systematic review and meta-analysis

**DOI:** 10.1136/bmjopen-2023-071776

**Published:** 2024-02-20

**Authors:** Chloe J Brennan, Carl Roberts, Jon C Cole

**Affiliations:** Psychology, University of Liverpool, Liverpool, UK

**Keywords:** OCCUPATIONAL & INDUSTRIAL MEDICINE, Systematic Review, EPIDEMIOLOGY, MENTAL HEALTH, PSYCHIATRY

## Abstract

**Objectives:**

Occupational moral injury and post-traumatic embitterment disorder (PTED) describe the psychological distress caused by exposure to injustice at work. This meta-analysis aims to determine the prevalence of occupational moral injury and PTED and establish whether prevalence estimates differ depending on occupation.

**Design:**

A systematic review and meta-analysis.

**Data sources:**

Google Scholar, PubMed, APA PsycINFO, Web of Science Core Collection, Scopus, ScienceDirect and Sage Journals Online were searched in June 2020 and updated in November 2022.

**Eligibility criteria for selecting studies:**

Observational studies that measured prevalence or average scores of moral injury, or PTED in any occupational group and any geographical location.

**Data extraction and synthesis:**

Two independent reviewers screened and coded eligible studies. Study design, participant demographics, sampling method, location, measurement tool and prevalence or average scores were extracted. Risk of bias was assessed using the Quality Assessment Checklist for Prevalence Studies tool. Meta-analysis was conducted using random effects models. Results that could not be combined were summarised qualitatively in a narrative synthesis using the Guidance for Systematic Reviews.

**Results:**

In total, 88 studies across armed forces and veterans, healthcare, first responders, educators, journalists, child protection service employees, the unemployed, public-sector employees and mixed occupations were included. Studies included in each separate meta-analysis based on the measure used ranged from 2 to 30. The pooled prevalence of clinically relevant moral injury in healthcare professionals was 45%, and exposure to any potentially morally injurious event (PMIE) across occupations was 67%. Exposure to transgressions by others and betrayal was significantly lower in the armed forces than civilian occupations. Pooled prevalence of PTED across occupations was 26%.

**Conclusion:**

Exposure to PMIEs, moral injury symptoms and PTED are prevalent at work and exposure to transgressions by others and betrayal are more likely in civilian occupations than the armed forces.

**PROSPERO registration number:**

CRD42020191766.

**STRENGTHS AND LIMITATIONS OF THIS STUDY**

- Guidelines from the Preferred Reporting Items for Systematic Reviews and Meta-Analyses Protocols were used to prepare this review.
- The Joanna Briggs Institute methodology for prevalence and incidence studies was used to develop the eligibility criteria, a search strategy, data extraction and risk of bias assessment.
- A comprehensive computer literature search of seven databases (Google Scholar, PubMed, APA PsycINFO, Web of Science Core Collection, Scopus, ScienceDirect and Sage Journals Online) was run in June 2020 and updated in November 2022.
- There was high heterogeneity among studies, even after employing subgroup analyses, so findings must be interpreted with caution.
- Only peer-reviewed articles written in English were included which may lead to publication bias.

## Introduction

Exposure to traumatic events at work is commonplace, and meta-analyses have identified certain occupational groups that do not have special training to deal with traumatic events^1^ and are at a higher risk of developing work-related post-traumatic stress disorder (PTSD).^1 2^ Individuals may also experience morally traumatic events at work, through interpersonal conflicts, making challenging ethical decisions and exposure to events that contradict what the person perceives to be ‘right’ based on their perception, values, beliefs and morals.^3–5^ Moral injury and post-traumatic embitterment disorder (PTED) are two concepts that have emerged to explain the harm caused by exposure to unjust and potentially morally injurious events (PMIEs). Neither moral injury nor PTED are categorised in current diagnostic manuals, such as the Diagnostic and Statistical Manual of Mental Disorders, V.5 or International Classification of Diseases, V.11, but they can have a disabling impact on individuals, such as social isolation, difficulty with relationships and difficulty maintaining employment. Organisations may experience higher rates of sickness absence and ill health retirement in employees and embitterment can lead to protracted litigation against employers.^6 7^

Moral injury refers to the psychological distress caused by exposure to PMIEs that involve perpetrating, witnessing or learning about acts that violate moral beliefs^4^ or experiences of betrayal.^5^ Traditionally, moral injury has been studied in the armed forces after witnessing the horrors of war, but in 2018, a meta-analysis showed that occupational moral injury is present in civilian occupations such as journalists, veterinarians, the police and teachers.^8^ Moral injury shares similarities in aetiology and symptomology with PTED,^9 10^ which is a reactive disorder caused by exposure to an unjust event and is characterised by feelings of embitterment, helplessness and hostility.^3^ Research has shown that PTED often occurs at work, for instance, 73% of PTED patients reported the triggering event as work-related.^11^ PTED is evident in occupations such as the armed forces,^12 13^ educators,^14 15^ healthcare professionals,^9 16 17^ the unemployed^18 19^ and in a mix of occupations.^20 21^

Despite this, there is no clear evidence for how widespread occupational moral injury and PTED are. As far as we are aware, no meta-analyses exist that combine prevalence estimates of PTED, and current attempts to combine the prevalence of morally transgressive acts^22^ and moral injury symptoms^23^ rely on a narrative synthesis of military studies only. Since certain occupational groups that do not have special training to deal with traumatic events are at a higher risk of developing work-related PTSD,^1^ it is possible that certain occupations have a higher risk of developing work-related moral injury and PTED following ethically challenging events. To accurately estimate the prevalence of occupational moral injury and PTED, and investigate whether rates differ depending on occupation, systematic methods are needed.

It is challenging to estimate the pooled prevalence of moral injury and PTED as there is no gold standard measurement approach. A review by Koenig *et al* 24 identified 17 scales that measure one or two aspects of moral injury, and five comprehensive measures of either exposure to PMIEs, moral injury symptoms or both. Researchers report prevalence using inconsistent cut-off values, and some only report average scores. Similarly, when reporting prevalence of PTED, researchers can use the PTED diagnostic interview,^25^ or the PTED self-rating scale, though three different cut-off values are used to indicate clinically relevant embitterment at three different levels of severity.^3^ Despite variability between studies, generating pooled prevalence estimates in different occupations is valuable because it can inform preventive strategies and policy decisions.

This current systematic review and meta-analysis aims to (1) determine the prevalence of occupational moral injury and PTED in military and civilian professions, (2) establish whether prevalence estimates differ depending on occupation, (3) establish whether methodological differences between studies influence prevalence estimates and (4) record the variables associated with occupational moral injury and PTED.

## Methods

Guidelines from the Preferred Reporting Items for Systematic Reviews and Meta-Analyses Protocols (PRISMA)^26^ were used to prepare this review. The Joanna Briggs Institute (JBI) methodology for prevalence and incidence studies^27^ were used to develop the eligibility criteria (table 1), a search strategy (online supplemental table 1), data extraction (online supplemental table 2) and risk of bias assessment (online supplemental table 3). This review was registered with the international prospective register of systematic review PROSPERO network.^28^

10.1136/bmjopen-2023-071776.supp1 Supplementary data

**Table 1:** Inclusion and exclusion criteria

Inclusion criteria	
Condition (outcome of interest)	Studies measuring the prevalence (%) or average scores (M and SD) of PTED, including chronic embitterment. Studies measuring prevalence (%) or average scores (M and SD) of moral injury. Uses PTED diagnostic interview, PTED self-rating scale or a comprehensive moral injury measure.
Context	All geographical locations.
Population	PTED or moral injury must have been measured in relation to occupation including studies reporting on multiple occupations, the unemployed or retired. 18+, any sex, ethnicity, educational status or socio-economic status.
Exclusion criteria	
Condition	Studies using the Bern Embitterment Inventory (BEI) to measure PTED. Studies which only measured theoretical subcomponents or limited dimensional scales of moral injury.
Context	Clinical patients in treatment or intervention studies.
Population	Student only samples. Entirely clinical samples or all exposed to a specific stressor that is, bullied at work, all exposed to a moral transgression, all injured at work, etc.

PTED, post-traumatic embitterment disorder.

### Search strategy and study selection

A computer literature search was run in June 2020 using seven databases, Google Scholar, PubMed, APA PsycINFO through the EBSCOhost interface, Web of Science Core Collection, Scopus, ScienceDirect and Sage Journals online. Articles related to PTED were included from 2003, which was when the first conceptual definition for PTED was developed,^29^ and articles related to moral injury were included from 2009 in line with the first comprehensive scientific definition of moral injury.^4^ Search terms were used as free text terms and combined with Boolean operators, so the same keywords could be replicated in each database. The search was restricted to peer-reviewed articles written in English. References were managed in EndNote (Clarivate Analytics, Philadelphia) and Excel. The searches were updated in November 2022. Details of the search are listed in online supplemental table 1. One researcher (CB) screened all titles, abstracts and full texts and a second reviewer (CR) screened 15% of the titles and abstracts and 100% of the full texts against the eligibility criteria. Any discrepancies over the inclusion of studies were resolved through discussion. At every stage of screening, inter-rater agreement was >90%, with Cohen’s kappa >0.90, indicating almost perfect agreement.^30^

The Condition, Context, Population mnemonic^25^ was used to determine inclusion and exclusion criteria and is outlined in table 1. Studies were included if they measured the prevalence or average scores of PTED or moral injury (including PMIEs) using the PTED diagnostic interview,^25^ PTED self-rating scale^3^ or a comprehensive and standardised measure of moral injury. Studies included in this review measured moral injury as a whole construct rather than measuring the separate theoretical elements, such as guilt and self-forgiveness (ie, see Koenig *et al* 24 for a review). For inclusion, moral injury and PTED must have been measured in an occupational group, in relation to work, or in unemployed or retired samples. Any working age, sex, ethnicity, country, educational status and sociodemographic status were included. Secondary outcomes of interest were univariate associates of moral injury or PTED. The review included observational studies (ie, cross-sectional, retrospective, prospective, cohort and longitudinal) and excluded intervention studies or reviews. Only peer-reviewed articles written in English were eligible. When multiple papers were identified using the same data, the most relevant paper was included.

### Data extraction

The following data were extracted; author and year published, study design, number of participants, mean age, the proportion of men, proportion of white ethnicity, socioeconomic status, geographical location, occupation, length of time in the job, sampling method including setting and dates, the measurement tool for moral injury or PTED, prevalence (N and proportion), average scores (mean and SD) and any conflict of interest. Secondary outcomes included univariate associates of moral injury and PTED, including the variable name, the measure used and the direction of effect (positive, negative or no association). For longitudinal and cohort studies, prevalence estimates or average scores from the baseline wave were extracted unless only subsequent waves were reported. CB contacted the authors to obtain relevant information that was not available in the text. One researcher (CB) completed the data extraction, and a second reviewer (CR) screened 100% of the data extraction to check for accuracy.

### Quality assessment

Quality assessment was conducted using the Quality Assessment Checklist for Prevalence Studies created by Hoy *et al*.^31^ This tool assesses the possibility of bias in design, conduct and analysis, including sample representativeness, sampling, non-response bias, data collection method, appropriateness of case definition, study instrument and statistical analysis. Each item is coded as 0 (yes, low risk) or 1 (no, high risk) and quality assessment scores were summed, and categorised into low (0–3), moderate (4–6) and high (7–9) risk. Two researchers (CB and CR) conducted the quality assessment, and any discrepancies were resolved through discussion (see online supplemental table 3).

### Data synthesis and analysis

We conducted the meta-analyses using R (V.4.2.1) with the Meta package.^32^ All meta-analyses used a random effects model using the restricted maximum-likelihood estimator due to the high heterogeneity associated with observational studies.^33^ Separate meta-analyses were conducted with the prevalence of PTED as the outcome, and then the prevalence of moral injury as the outcome. For PTED prevalence, three separate analyses were conducted based on a cut-off >1.6, then 2 and then 2.5, which represents different levels of clinical severity.^3^ For moral injury, due to the heterogeneity in moral injury measures (see table 2 for an overview), a separate meta-analysis was conducted for each moral injury measure and their associated subscales. Some moral injury scales do not use cut-off values to estimate the prevalence and only report average scores, so additional meta-analyses were conducted with the mean of PTED and mean of moral injury scores as the outcome.

**Table 2:** PTED and moral injury measurement scales included in the systematic review

Measure	k	Events or symptoms	Decision regarding classification	Subscales	Cut-off value	Scale information
PTED self-rating scale^3^	11	Symptoms linked to event	Assess embitterment reactions to event(s) specified in instructions.	Unidimensional	>1.6, >2, >2.5	19 items (0–4)
MIES^113^	38	Combined	Assesses exposure to PMIEs and effects (ie, feeling troubled by those acts).	Bryan *et al* 44—three factors: transgressions-others, transgressions-self and betrayal. Nash *et al* 113—two factors: perpetration and betrayal. Richardson *et al* 58—two factors: MI-self and MI-other. Lamb *et al* 88—three factors: commission, omission and betrayal.	4+ (slightly agree) 5+ (moderately agree) 6+ (strongly agree)*	9 items (1–6 Likert)
MIQ-M^45^	9	Combined	Assesses military-specific PMIEs, and effects.	Primarily unidimensional. Lancaster and Harris^66^—two factors: MIQ causes and MIQ effect	None	19 items (1–4 Likert)
MIQ-T^46^	1	Combined	Assesses teacher-specific and effects.	Unidimensional	None	12 items (1–4 Likert)
MIQ-M modified^65^	1	Events and symptoms	Modified PMIE items from the original MIQ-M which conflated PMIEs and effects into PMIEs and defining characteristics.	Three PMIE subscales—AoW, PCoW and LFB and three subscales for their defining characteristics	2+ (seldom) on each item	22 PMIE items and five defining characteristics items per PMIE (1–4 Likert)
EMIS-M^47^	7	Symptoms	Assesses guilt, shame, moral concerns, self-condemnation, social withdrawal and inability to forgive self, anger, feelings of betrayal, revenge and disgust.	two subscales: self-directed and other-directed	None	17 items (1–5 Likert)
EMIS-SF^120^	1	Symptoms	Assesses emotions (guilt and shame) and beliefs/attitudes (moral disgust and mistrust).	Unidimensional	None	4 items (1–5 Likert)
MISS-HP^93^	12	Symptoms	Assesses betrayal, guilt, shame, moral concerns, loss of trust, loss of meaning, difficulty forgiving, self-condemnation, religious struggle and loss of religious/spiritual faith.	Unidimensional	>36†	10 items (1–10 visual analogue Likert)
MIOS^6^	4	Symptoms linked to PMIE	The symptom scale refers to the most distressing PMIE. Symptoms include self-blame, loss of faith, trust, worthiness, goodness in others, honesty, pride, self-hatred, disgust and anger.	Two subscales: Shame-related (SR) and trust violation-related (TVR)	None‡	14 items (0–4)
BMIS^97^	1	Events and symptoms	Endorsement of at least one PMIE assessed needed to complete symptom questions.	Two subscales: BMIS-event (BMIS-E) and BMIS-sequelae (BMIS-S)	2+PMIE Symptom subscale: None	7 items (0–3)
MIA-PSP^100^	1	Combined	Assesses feeling ‘bothered’ by PSP-specific PMIEs, and symptoms (ie, feelings of sadness, anger, self-hatred, guilt, shame, disgust and fear).	Three subscales: perpetration, betrayal and emotional sequelae	None	17 items (1-6)
Toronto scale for journalists^98^	1	Combined	Assesses journalism-specific PMIEs and effects (ie, feeling troubled by those PMIEs).	Three subscales: organisational/management; individuals/non-management and online	None	9 items (0–4)

The number of items and scoring is based on the typical use of the scale.

*Some papers modified the Likert scale and used a cut-off of 3+ ‘sometimes’ and 4+ ‘often’^51^ or 3+ ‘agree’.^70 90^

†The German MISS-HP (G-MISS-HP) uses a 9 item scale with a cut-off>28.5,^107^ The Persian MISS-HP uses a cut-off>36.5.^92^

‡PMIE questions precede the MIOS symptoms scale and can be quantified as endorsed (yes/no).

AoW, Atrocities of War; BMIS, Brief Moral Injury Screen; EMIS-M, Expressions of Moral Injury Scale-Military Version; EMIS-SF, Expressions of Moral Injury Scale-Short Form; LFB, Leadership Failure or Betrayal; MIA-PSP, Moral Injury Assessment for Public Safety Personnel; MIES, Moral Injury Events Scale; MIOS, Moral Injury Outcome Scale; MIQ-M, Moral Injury Questionnaire—Military version; MIQ-T, Moral Injury Questionnaire—Teacher version; MISS-HP, Moral Injury Symptoms Scale—Health Professionals; PCoW, Psychological consequences of War; PTED, post-traumatic embitterment disorder.

Subgroup analyses were conducted if there were sufficient studies of k >10,^34^ grouping studies based on occupation. Forest plots were generated, displaying the prevalence and 95% CIs for each estimate, the overall pooled estimate and 95% CIs for each occupational group. Additional subgroup analyses were conducted based on study quality (low or medium risk) and on cut-off value used for moral injury prevalence meta-analyses. If a study reported the prevalence of moral injury using multiple cut-off scores in the same paper, then the most common across studies was chosen for inclusion. A random effect model with a common estimate for τ2 was used in subgroup analyses due to k ≤5 in some of the subgroups.^35^ In order to determine whether any of the subgroups were statistically different, a *Q*-test with p<0.05 was used based on the overall subgroup results. No overlap in 95% CIs was used to determine which groups were significantly different from one another.^36^

For meta-analyses that used prevalence estimates as the outcome, the Freeman-Tukey double arcsine transformation method was applied, which is effective when there are small sample sizes and extreme prevalence estimates.^37 38^ Heterogeneity was assessed using *I* ^2^ with a cut-off of >50% suggesting high heterogeneity.^39^ Significance was assessed using *x^2^* for Cochrane’s Q (p<0.05). Funnel plots are not suitable to assess publication bias in meta-analyses of proportions.^40^ Therefore, a sensitivity analysis was used, comparing prevalence and average scores when outliers were removed, which were extremely small or large values that fell outside of the CI of the pooled effect.^41^

Finally, where prevalence or average scores could not be pooled due to inconsistent reporting or insufficient studies (*k*=1), narrative synthesis was used to describe estimates. The narrative synthesis also described the univariate associates of PTED and moral injury using the Guidance for Systematic Reviews.^42^ Associates were grouped into demographic, mental health and individual/work variables, and the percentage of studies that reported each type of variable was calculated. Vote counting was used to determine whether the overall relationship between variables with PTED and moral injury was positive, negative or no association.

### Patient and public involvement

We conducted no primary research involving patients because the systematic review focused on published literature.

## Results

### Study characteristics

**Figure 1:**
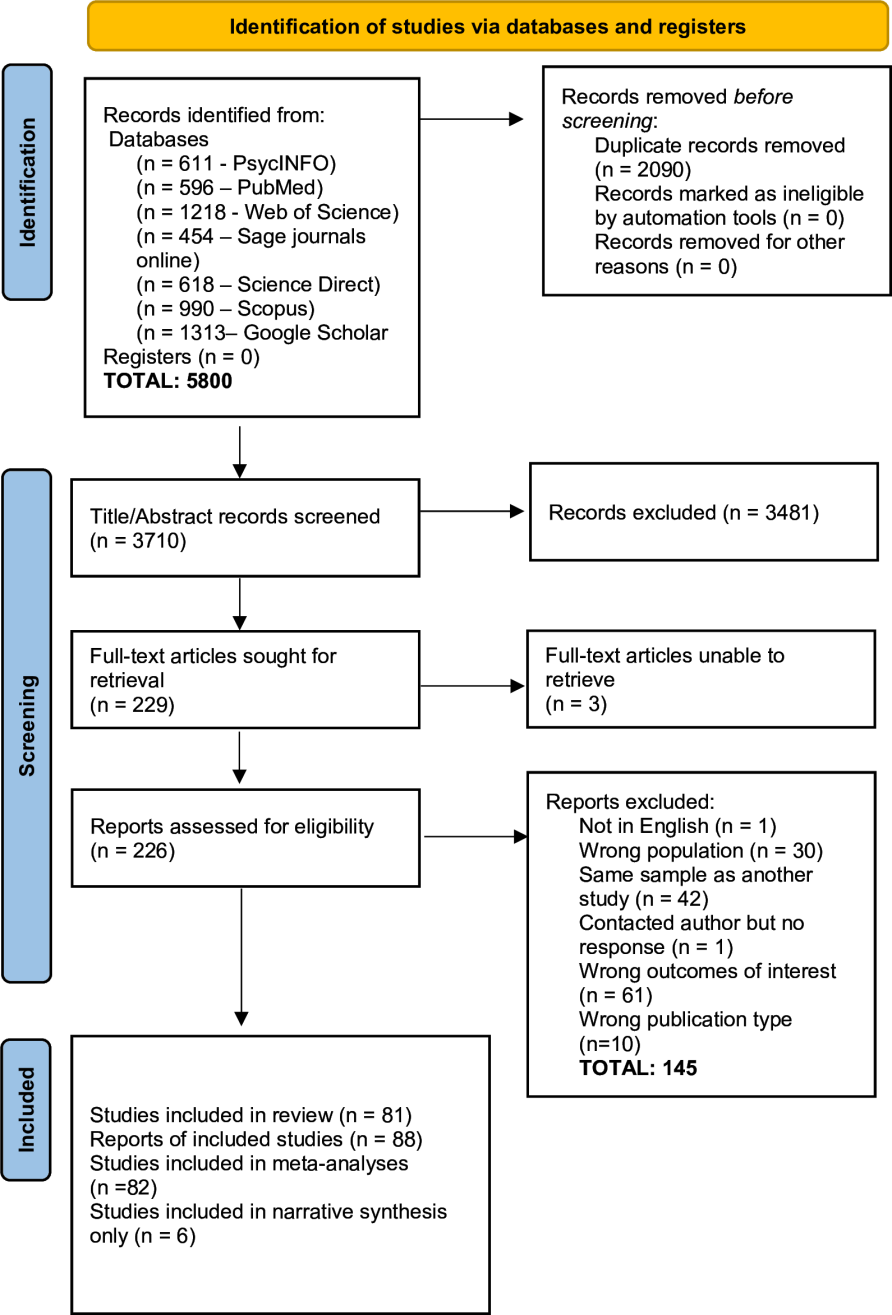
Preferred Reporting Items for Systematic Reviews and Meta-Analyses (PRISMA) flow diagram.

The PRISMA flowchart delineates the review process (see figure 1). Of the 81 full texts included in the review,^6 12–21 43–112^ some reported multiple studies in the same paper, so there were 88 separate studies included in the review. Of these, six reported prevalence estimates or average scores that could not be pooled, so they were included in the narrative synthesis only. Of the 88 studies included, 10 measured PTED, one measured both PTED and moral injury and 77 measured moral injury. The number of studies included in each separate meta-analysis based on the measure used ranged from 2 to 30.

The characteristics of included studies are in online supplemental table 2 and the risk of bias information is in online supplemental table 3. Of the 88 studies, 10 had a low risk of bias (11%) and all others were medium risk, 77 (87.5%) were cross-sectional and all others were longitudinal, and sample sizes ranged from 38 to 14 057. Despite a small number of PTED studies, there was a varied geographical distribution, with three studies being conducted in the United Kingdom (UK) and one study each conducted in Germany, Turkey, Bosnia and Herzegovina, Cyprus, Pakistan and South Korea. Occupations also varied with samples, including healthcare (n=4), educators (n=2), mixed occupations (n=2), the unemployed (n=2), veterans (n=1) and the public sector (n=1). In contrast, most moral injury studies were conducted in the USA (n=41), and in military samples (n=39). Health and social care workers (HSCWs) were the second biggest occupational group (n=32), and other occupations were less represented, including first responders (n=2), educators (n=2), journalists (n=2) and Child Protection Services (CPS) employees (n=1). Geographically, aside from the USA, studies were conducted in the UK (n=6), Canada (n=5), Israel (n=4), Australia (n=3), Ireland (n=2), China (n=2) and other countries with only one study included El Salvador, Korea, Finland, Pakistan, Romania, Iran, Honduras, Italy, Croatia, German-speaking populations, and Turkey.

Table 2 shows the characteristics of the PTED, and moral injury measurement scales included in the review. All studies measuring PTED used the PTED-self rating scale, in contrast to studies measuring moral injury, which included 11 distinct scales, five of which conflated PMIEs with symptoms in the same items, two scales assessed both PMIEs and symptoms but separately, and four assessed moral injury symptoms only. The most common moral injury scale was the Moral Injury Events Scale (MIES)^113^ (n=38), which conflates events and symptoms, followed by the moral injury symptom scale for healthcare professionals (MISS-HP),^93^ which assesses clinically relevant symptoms of moral injury in healthcare professionals (n=12). Most moral injury scales are occupation-specific, and there was variation in reporting regarding the number of items, scoring, the cut-off value used, and subscale factor structure endorsed across the moral injury scales.

### Prevalence meta-analyses

Online supplemental figures 1–9 show the forest plots with pooled prevalence estimates for PTED and moral injury, split by occupation where possible (k>10).

#### Post-traumatic embitterment disorder

The pooled prevalence of PTED when using a cut-off of >1.6 was 37%–95% CI (24.4% to 49.9%), when using a cut-off of >2 was 26%–95% CI (16.1% to 37.4%), and when using a cut-off of >2.5 was 11%–95% CI (4% to 21.3%). No subgroup analyses were conducted due to k ≤10.

#### Moral injury

Using the MISS-HP, the pooled prevalence of clinically relevant signs of moral injury in healthcare professionals was 45%–95% CI (36.4% to 53.5%). Across occupations, the pooled prevalence of exposure to any PMIE on the MIES scale was 67%–95% CI (59.0% to 74.5%), exposure to perpetration-based PMIEs was 20%–95% CI (1.1% to 53.7%), transgressions by self PMIEs was 31%–95% CI (24.7% to 37.5%), transgressions by other PMIEs was 48%–95% CI (38.0% to 57.5%) and betrayal-based PMIEs was 46%–95% CI (39.5% to 52.6%). While not significantly different, exposure to any PMIE was lowest in first responders (58%, 95% CI (30.9% to 82.5%)) followed by the armed forces (59%, 95% CI (45.9% to 72.4%)), healthcare professionals (71%, 95% CI (60.7% to 81.0%)) and educators (88%, 95% CI (82.5% to 91.7%)). Furthermore, while not significantly different, exposure to transgressions by self was also lowest in first responders (21%, 95% CI (16.1% to 26.8%)), followed by CPS professionals (24%, 95% CI (11.4% to 40.2%)), the armed forces (29%, 95% CI (19.9% to 39.6%)), healthcare professionals (33%, 95% CI (23.4% to 43.9%)) and educators (43%, 95% CI (36.0% to 49.5%)).

There were significant differences in the prevalence of exposure to transgressions by others, and betrayal-based PMIEs based on occupation (*p*’s<0.01). Lack of overlap in CI shows that the armed forces were exposed to significantly fewer transgressions by others (31%, 95% CI (20.1% to 41.8%)) compared with healthcare professionals (55%, 95% CI (43.9% to 65.6%)), first responders (61%, 95% CI (54.3% to 66.9%)), educators (80%, 95% CI (74.4% to 85.3%)) and CPS professionals (92%, 95% CI (78.6% to 98.3%)), though first responders, educators and CPS professionals included only one study each. Furthermore, the armed forces were exposed to significantly fewer betrayal events (33%, 95% CI (26.3% to 39.4%)) compared with healthcare workers (58%, 95% CI (51.0% to 65.1%)) and educators (68%, 95% CI (61.3% to 74.0%)). While not significantly different, first responders had higher estimates than the armed forces (52%, 95% CI (35.2% to 69.1%)), and those in the CPS had the lowest exposure to betrayal, but this was based on only one study (32%, 95% CI (17.5% to 48.7%)).

### Mean score meta-analyses

Online supplemental figures 10–24 show the forest plots with pooled mean estimates for PTED and moral injury, split by occupation where possible (k>10).

#### Post-traumatic embitterment disorder

The pooled mean for the PTED scale across occupations was 1.52, and this was not significantly different according to occupation due to wide CIs.

#### Moral injury

Since most moral injury scales only report average scores (ie, mean and SD) and not prevalence estimates, pooled mean estimates for the Moral Injury Questionnaire—Military version (MIQ-M), Expressions of Moral Injury Scale (EMIS), Moral Injury Outcome Scale (MIOS), MISS-HP and MIES were conducted. Only the pooled mean for transgressions by others on the MIES (M=3.16) significantly differed across occupations (p<0.01), with significantly lower scores in the armed forces (2.66, 95% CI (2.23 to 3.09)) compared with first responders, educators and CPS.

### Sensitivity analyses: outliers removed

Table 3 shows the pooled prevalence and mean estimates, before and after removing outliers for PTED and moral injury.

**Table 3:** Pooled prevalence and mean estimates for PTED and moral injury, with and without outliers

Measure	With outliers			Without outliers		
	k	Pooled % (95% CI)	I2	k	Pooled % (95% CI)	I2
PTED 1.6	9	36.65 (24.38 to 49.85)	98%	6	31.27 (23.87 to 39.17)	84%
PTED 2	8	26.03 (16.09 to 37.38)	97%	7	21.21 (16.40 to 26.44)	75%
PTED 2.5	6	22.31 (4.02 to 21.32)	98%	7	8.16 (6.37 to 10.14)	62%
MIES any	23	67 (59.01 to 75.54)	98%	11	73.36 (70.18 to 76.42)	76%
MIES transgressions—self	27	30.95 (24.72 to 37.54)	99%	11	22.99 (21.62 to 24.32)	4%
MIES transgressions—others	25	47.73 (38.04 to 57.51)	98%	14	49.20 (43.82 to 54.59)	93%
MIES betrayal	28	46.03 (39.54 to 52.59)	98%	11	46.12 (40.97 to 51.32)	91%
MIES perpetration	2	20.26 (1.11 to 52.70)	98%		No outliers	
MISS-HP	10	44.88 (36.40 to 53.50)	98%	7	43.33 (39.30 to 47.41)	85%
	**k**	**Pooled mean (95% CI)**	**I2**	***k***	**Pooled mean (95% CI)**	**I2**
PTED	11	1.52 (1.10 to 1.93)	99%	7	1.33 (1.18 to 1.49)	97%
MIES total	29	2.58 (2.35 to 2.82)	99%	16	2.61 (2.52 to 2.70)	86%
MIES transgressions—self	27	2.26 (1.99 to 2.52)	99%	12	2.16 (2.95 to 2.28)	77%
MIES transgressions—others	24	3.16 (2.81 to 3.52)	99%	10	3.05 (2.89 to 3.20)	83%
MIES betrayal	30	2.74 (2.51 to 2.98)	99%	15	2.77 (2.67 to 2.86)	80%
MIES perpetration	5	2.55 (2.09 to 3.02)	99%	4	2.77 (2.60 to 2.95)	83%
MIQ-M total	5	1.77 (1.58 to 1.96)	97%	4	1.84 (1.66 to 2.02)	91%
MIQ-M causes	4	1.42 (1.21 to 1.60)	96%	3	1.31 (1.26 to 1.36)	75%
EMIS total	4	2.22 (1.90 to 2.54)	99%		No outliers	
EMIS self	4	1.96 (1.68 to 2.24)	92%		No outliers	
EMIS others	5	2.51 (2.28 to 2.74)	93%	4	2.61 (2.46 to 2.76)	55%
MISS-HP	12	36.73 (33.39 to 40.07)	99%	7	35.42 (34.14 to 39.87)	76%
MIOS total	4	27.75 (24.19 to 31.31)	95%	3	25.88 (24.45 to 27.20)	66%
MIOS shame	4	12.77 (10.43 to 15.12)	98%	3	11.42 (11.01 to 11.83)	0%
MIOS trust	4	14.97 (13.65 to 16.28)	94%		No outliers	

EMIS, Expressions of Moral Injury Scale; MIES, moral injury events scale; MIOS, Moral Injury Outcome Scale; MIQ-M, Moral Injury Questionnaire—Military version; MISS-HP, Moral Injury Symptoms Scale—Health Professionals; PTED, post-traumatic embitterment disorder.

#### Prevalence

Pooled prevalence estimates for PTED decreased when outliers were removed, using all three cut-offs. Prevalence was reduced to 31%–95% CI (23.9%, 39.2%) I^2^=84%, when using a cut-off >1.6, reduced to 21%–95% CI (16.4% to 26.4%) I^2^=75%, when using a cut-off >2 and decreased to 8%–95% CI (6.4% to 10.1%) I^2^=62%, when using a cut-off>2.5.

The prevalence of moral injury in healthcare professionals on the MISS-HP was 43%, 95% CI (39.3% to 47.4%) I^2^=85%, and the prevalence of exposure to transgressions by self PMIEs decreased to 23%–95% (CI 21.6% to 24.3%) I^2^=4%. All other types of PMIEs increased, for example, exposure to any PMIE increased to 73%–95% CI (70.2% to 76.4%) I^2^=76%, transgressions by others increased to 49%–95% CI (43.8% to 54.6%) I^2^=93%, and betrayal stayed relatively similar at 46%–95% CI (41.0% to 51.3%) I^2^=93%. Except for exposure to transgressions by self, heterogeneity remained high even after removing outliers (≥50%).

#### Mean scores

Pooled mean estimates decreased for six of the moral injury scales or subscales after removing outliers and increased for six of the scales (see table 3). Except for the MIOS trust subscale, heterogeneity remained high even after removing outliers (≥50%).

### Subgroup analyses: study quality and cut-off value

Additional subgroup analyses were conducted based on the risk of bias where possible (k >10; online supplemental table 3). For prevalence meta-analyses, exposure to any PMIE was significantly lower in low-risk studies (47%, 95% CI (32.4 to 61.9), *k*=5) compared with medium risk (72%, 95% CI (64.8% to 79.2%), *k*=18, p=0.003). Furthermore, exposure to transgressions by self was significantly lower in low-risk studies (16%, 95% CI (84.7% to 25.2%), *k*=4) compared with medium risk (35%, 95% CI (28.3% to 41.8%), *k*=22, p=0.006), and exposure to betrayal PMIEs was significantly lower in low-risk studies (30%, 95% CI (18.5% to 43.7%), *k*=5) compared with medium-risk studies (50%, 95% CI (43.1% to 56.3%), *k*=23, p=0.011). The pooled prevalence estimates of transgressions by others were not significantly different based on the risk of bias (p=0.254). When comparing pooled mean estimates based on the risk of bias, only the pooled mean for betrayal was significantly lower in low-risk studies (2.03, 95% CI (1.33 to 2.73), *k*=3) compared with medium risk (2.83, 95% CI (2.60 to 3.06), *k*=27, p=0.034). For the prevalence meta-analyses, different cut-off values were used to signify moral injury on the MIES and the MISS-HP (see table 2). Therefore, additional subgroup analyses based on the cut-off value were conducted (see online supplemental table 5) and found no significant differences in pooled prevalence estimates depending on the cut-off value (p’s >0.05).

### Narrative synthesis

#### Prevalence and mean scores

Online supplemental table 6 shows the prevalence and average scores for moral injury scales that could not be combined (*k*=1), including different variations of MIES subscales (ie, MI-other; 20%, MI-commission; 14%, MI-omission; 5%), or moral injury profiles based on the MIES (no moral distress; 42%, moral distress-other; 19%, witnessing-only; 16%, moral distress-self; 8%, moral distress-self and other; 15%). Variations of the MIQ-M include the teacher version (MIQ-T; M=1.16) or the modified version. Other moral injury scales were only included in one study each, such as the Moral Injury Assessment for Public Safety Personnel (MIA-PSP; M=59.6), or the Toronto moral injury scale for journalists (M=1.5). See online supplemental table 6 for specific average scores or prevalence estimates for the associated subscales.

#### Univariate associates of PTED and moral injury

A narrative review was used to synthesise the associates of PTED and moral injury due to the heterogeneity of variables measured, with the exception of mental health variables, which were frequently measured in moral injury studies; however, meta-analyses of this relationship have been conducted recently.^24 114 115^

#### Demographic variables

Of the PTED studies, six (55%) measured demographic variables. Overall, there was no association with gender, a positive association with length of time in the job and a mixed association with age, with two papers finding a positive association, and two papers finding no association. Many variables were measured less frequently across studies (ie, k <3), such as ethnicity, professional role, hours worked, having dependents, marital status, income and education level. Of the moral injury studies, 31 (40%) measured demographic variables. Overall, there was no association with gender, age, length of time in the job, marital status, ethnicity/race/country of birth, education and income. Several variables were measured less frequently (ie, k <3), such as being unemployed and having dependents, and several papers measured variables specific to the work environment such as professional role, though varied job roles preclude comparison.

#### Mental health variables

Of the PTED studies, seven (64%) measured mental health variables and found positive associations with depression, stress, anxiety, existing mental health diagnoses, psychological distress, negative affect and a negative association with positive affect and employee well-being, though none of these variables was measured frequently (k <3). Of the moral injury studies, 51 (65%) measured mental health variables and found positive associations with depression, PTSD or PTSD subscales, guilt or shame, anger, anxiety, burnout or its subscales, drug and alcohol use and a negative association to well-being. Other variables related to mental health appeared less frequently (ie, k <3).

#### Individual/work characteristics

Of the PTED studies, 10 (91%) measured individual/work characteristics. Justice beliefs were negatively associated with PTED. Other variables were measured less frequently (k<3) such as rumination, unemployment duration and appraisal, supervisory control, job demands, work attitudes, social support, self-efficacy, leadership behaviours and personality traits such as optimism, self-esteem and resilience, though generally PTED was associated with undesirable work factors. Of the moral injury studies, 40 (51%) measured individual/work characteristics. The most common positive association was with combat exposure. Religiosity was negatively associated with moral injury, although two papers found a positive association, turnover intentions were positively associated with moral injury, and resilience associations were mixed, showing positive, negative and no associations with moral injury. COVID-related factors were measured across several studies, but variations in the type of COVID variables measured preclude comparison. Many variables related to personality variables, beliefs and specific work environmental factors were measured less frequently (k<3).

## Discussion

This is the first meta-analysis of the prevalence and average scores of occupational moral injury and PTED, so the first aim of the paper has been met. The results showed that across occupations, 37% of staff scored above a differentiation point for elevated embitterment (≥1.6), 26% had clinically relevant embitterment (≥2) and 11% showed embitterment of a clinically significant intensity with improved clinical practicability and specificity (≥2.5). Findings also showed that across occupations, 67% were exposed to at least one PMIE, 20% were exposed to a perpetration-based PMIE, 31% committed a moral transgression, 48% witnessed a transgression by others and 46% were exposed to betrayal PMIEs at work. The second aim was to establish whether prevalence estimates differ, depending on occupation, and this aim has been partially met because some moral injury scales were occupation specific or used different subscales meaning they could not be pooled across occupations. Despite this, exposure to transgressions by others and betrayal were significantly lower in the armed forces compared with healthcare workers and educators. Finally, clinical levels of moral injury in healthcare professionals, according to the MISS-HP, was 45%. Since several moral injury scales did not provide prevalence estimates, mean scores were also pooled, and for PTED, it did not differ according to occupation. Mean scores of exposure to transgressions by others were significantly lower in the armed forces compared with first responders, CPS, and educators.

The third aim was to determine whether methodological differences between studies influenced prevalence estimates, and this was shown by methodological differences between studies precluding some analyses. In addition, prevalence estimates for exposure to any PMIE, transgressions by self and betrayals were lower in low-risk compared with medium-risk studies, and the mean scores for betrayal were also lower in low-risk studies. Furthermore, prevalence estimates for moral injury were not significantly different based on the cut-off value used by the researcher. The final aim of the systematic review was to record the variables associated with moral injury and PTED. A narrative review identified variables measured in association with moral injury and PTED, which fell into three categories: demographics, mental health and individual/work characteristics. Gender was found to have no association with moral injury and PTED, whereas the length of time in the job had a positive association, and age had a positive or no association with PTED. In contrast, age, length of time in the job, marital status, ethnicity/race/country of birth, education and income all had no association with moral injury. Other demographic variables varied and were usually only included in one or two studies. Moral injury and PTED showed a positive association with mental health variables, including depression, stress, anxiety and employee well-being, and moral injury studies studied the relationship with a wider range of disorders such as PTSD and anxiety, and emotional (ie, guilt, anger, shame), behavioural (ie, burnout, compassion fatigue) and spiritual variables. Finally, individual/work variables differed between the two constructs, with moral injury studies focusing primarily on the combat environment as a risk for military samples and PTED focusing on justice beliefs as protective in civilian samples. Furthermore, religiosity and resilience were measured in relation to moral injury with mixed results, and turnover intentions were positively associated with moral injury. Individual risk and resiliency factors such as optimism, self-esteem and personality traits were also identified, but less frequently. Due to the infrequency of variables measured, the associations reported here are inconclusive, and this aim has only been partially met.

The results of this study should be considered in light of several strengths and limitations. The study protocol was preregistered on PROSPERO and followed the JBI guidance for systematic reviews of prevalence and incidence data, which ensures the methods were robust.^27^ In line with guidance from the Cochrane Handbook,^116^ two researchers screened 15% of all abstracts and titles, and 100% of full texts, data extraction and quality assessment. However, using three screeners would improve objectivity. Despite using robust methods, there was high heterogeneity within individual studies, and among studies, even after employing subgroup analyses to explain the variability based on study quality, the cut-off score used and sensitivity analyses to remove outliers. While heterogeneity is often high in prevalence meta-analyses, and the median *I* ^2^ was found to be 96.9% in a review of 134 prevalence meta-analyses,^117^ caution must be taken when interpreting our findings. Furthermore, only 11% of studies had a low risk of bias, and often the primary aim of the included studies was not to produce prevalence data. This may explain the use of inappropriate statistical methods (ie, lack of CIs or weighting) and variability in reporting only average scores or prevalence data based on the various subscale configurations or using different cut-off values. To deal with this heterogeneity, separate meta-analyses were conducted based on the type of data reported (ie, prevalence or average scores) and the measure used, reducing the number of studies included in each meta-analysis and thus power, meaning that subgroup analyses based on occupation were not always possible. Finally, while we searched a relatively high number of databases, it is possible that references from the grey literature have been missed.

Despite the limitations, this review adds to the growing literature on the prevalence of moral injury and PTED. Prior estimates of PTED in the general population were at 2.5%^3^ and this compares to 26% across occupations in this review. This confirms the findings by Linden *et al*,^11^ which found that 73% of PTED patients reported the triggering event as work-related and shows unjust and embittering events often occur in the workplace. Using the most stringent cut-off, occupational health services could expect 11% of their staff to display clinically severe levels of PTED according to these findings. There were not enough studies to conduct subgroup analyses based on the prevalence of PTED across occupations, though subgroup analyses, using the mean PTED scores, showed no significant difference based on occupation. This might suggest that PTED is widespread across professions. However, this analysis was based on only 11 studies, and the inclusion of more studies would be needed to confirm that this effect is not due to low statistical power.

Previous attempts to collate prevalence estimates for moral injury have relied on a narrative synthesis of the prevalence of morally transgressive acts^22^ or moral injury symptoms in military samples.^23^ Findings from this meta-analysis show that 67% of people across occupations have been exposed to at least one PMIE in their jobs, which is a high proportion. Furthermore, staff were more often exposed to transgressions by others (48%) and betrayal PMIEs (46%) than PMIEs perpetrated by themselves (31%), which mirrors qualitative findings during the pandemic, which shows healthcare staff experienced institutional betrayal more than perpetration-based moral injuries.^118 119^ Estimates of exposure to PMIEs are taken from the MIES,^22^ which was the most widely used scale to measure moral injury across studies in this review. While it is arguable that not all individuals exposed to PMIEs will experience moral injury symptoms, the MIES does confound events and effects by asking participants if they feel troubled by their exposure to moral violations.^24^ This suggests that a high proportion of staff are not only exposed to events that are morally injurious but are also affected by this. This is supported by further findings from this meta-analysis, which showed that 45% of healthcare workers had clinically relevant signs of moral injury according to the MISS-HP, which is the only moral injury symptom scale, which uses a clinical cut-off.^93^ Since the MISS-HP is healthcare specific, and the other symptom scales in this review only reported mean scores and not prevalence estimates, it is not possible to determine whether any occupations are at a greater risk of clinically relevant moral injury.

However, the review was able to show that members of the armed forces were exposed to significantly fewer transgressions by others and betrayal PMIEs than healthcare workers and educators, and the mean scores for transgressions by others were significantly lower in the armed forces than first responders, CPS and educators. Therefore, despite the definitions of moral injury originating from the military,^4 5^ this shows that civilian occupations are at an even greater risk of exposure to PMIEs and of feeling troubled by these. This mirrors findings which show that certain occupations have a higher risk of developing work-related PTSD, such as professions that do not have special training to deal with traumatic events.^1^ It is possible that military training is a protective factor and armed forces are less troubled by betrayal trauma as a result.

This meta-analysis identified several methodological issues. Prevalence of exposure to any PMIE, transgressions by self and betrayals were lower in low risk compared with medium-risk studies, and the prevalence of PTED differed depending on the cut-off value used. As highlighted by Koenig *et al* 24 there are several moral injury scales, which conflate events and symptoms (MIES, MIQ), and our review has identified more recently developed scales, which also do this (ie, MIA-PSP, Toronto scale for journalists). Other scales only measure symptoms (ie, EMIS, MIOS, MISS-HP), while many scales are occupation-specific precluding comparisons across occupations, and not all scales have established cut-off values, which contributes to inconsistencies in this area. Furthermore, there is a lack of randomly selected, nationally representative, epidemiological studies on the prevalence of moral injury and PTED in specific populations of interest (ie, the armed forces, HSCWs, first responders, educators, journalists, CPS employees) and in the general working population.

These findings reveal several implications and gaps that can form the basis of future research. First, clinically relevant signs of PTED are much higher in work settings than in the general population, and just under half of the healthcare professionals showed clinical levels of moral injury. Furthermore, occupations such as healthcare professionals, educators and the CPS are especially likely to experience transgressions by others and healthcare professionals, and educators are also more likely to experience betrayals at work than the armed forces. Understanding the prevalence of moral injury and PTED in different occupations is critical for developing targeted prevention strategies and can guide policy. However, these occupations relate to statutory organisations and frontline services, and it is possible that other civilian occupations have a lower, or moderate risk of exposure to PMIEs, and moral injury. Research across a broader range of professionals to understand which civilian occupations are at low, moderate, and high risk of exposure to PMIEs, moral injury and PTED would be valuable. The narrative synthesis showed that moral injury and PTED are associated with poor mental health outcomes such as depression, stress and anxiety, and poor work outcomes such as sickness absence, and turnover intentions. Therefore, employers, occupational health, and clinicians should be aware of these conditions and provide appropriate support. Currently, there are no proven recommended treatments for moral injury and PTED, and this research shows organisations will need strategies to support employees given the high risk of experiencing moral injury and PTED. Standardised measurements of moral injury and PTED, and proven treatment options need to be developed. In addition, while the number of studies on moral injury has grown exponentially in recent years, research on occupational PTED still represents a significant gap, and the lack of studies precluded prevalence subgroup analyses based on occupation. More studies are needed in this area. Second, while there were more moral injury studies, most were conducted with military or healthcare professionals, and other occupations such as educators, journalists, CPS, and first responders were represented in only one or two studies. Prevalence estimates indicate educators and CPS employees may be at an especially high risk, and more studies are needed to confirm these findings. Finally, most moral injury studies were conducted in the USA, and studies are needed across more geographical locations.

In conclusion, we are the first to provide meta-analytic evidence that the prevalence of moral injury and PTED at work are high. We identified occupations at a greater risk of exposure to PMIEs but inconsistencies in measurement scales, subscale configurations or cut-off values used among studies precluded some analyses. To strengthen the reliability of these findings, researchers must agree on a gold-standard approach to measuring moral injury and PTED, across a range of low, moderate and high-risk occupations with appropriate cut-off values. Despite this, high prevalence rates of moral injury and PTED show that proven, recommended treatment options and guidance should be developed to support employees and can inform policymakers on which areas of research and prevention to prioritise.

## Supplementary Material

Reviewer comments

Author's manuscript

## Data Availability

All data relevant to the study are included in the article or uploaded as supplementary information.
