# Supplementary material for: Prevalence of occupational moral injury and post-traumatic embitterment disorder: a systematic review and meta-analysis
Source: BMJ Open. 2024 Feb 20;14(2):e071776. doi: 10.1136/bmjopen-2023-071776 (PMC10882372; doi:10.1136/bmjopen-2023-071776)
Supplement: Supplementary data [file bmjopen-2023-071776supp001.pdf]

Supplementary materials

Supplementary Table 1.
Search strategy

Search date: 22 June 2020 and updated 2 November 2022

Moral injury search strategy

APA PsycINFO (EBSCOhost interface)

(("moral injur*" OR "transgressive act*" OR "moral transgression")) AND ((Veteran* OR military OR army OR marine* OR "national service" OR "territorial army" OR "armed forces" OR "special forces" OR RAF OR "air force" OR navy OR "service personnel" OR "ex-service personnel" OR submariner* OR sailor* OR seamen OR seaman OR airmen OR airman OR soldier* OR troop* OR "enlisted personnel" OR "armed forces" OR "armed services" OR police* OR fireman OR firemen OR firefighter* OR teacher* OR "humanitarian aid worker" OR "relief aid worker" OR "social worker" OR "disaster responder" OR policeman OR policemen OR policewoman OR "law enforcement" OR healthcare OR nurse OR doctor OR frontline OR "health professional" OR "hospital staff" OR "hospital worker" OR work OR occupation OR job OR "emergency service"))). Filters Published: 2009-2022; Expanders - Apply equivalent subjects; Narrow by Language: - English, Search modes - Find all my search terms.

PubMed

("moral injur*" OR "morally injurious" OR PMIE* OR "potentially morally injurious" OR "moral transgression" OR "transgressive act*") AND (Veteran* OR military OR army OR marine* OR "national service" OR "territorial army" OR "armed forces" OR "special forces" OR RAF OR "air force" OR navy OR "service personnel" OR "ex-service personnel" OR submariner* OR sailor* OR seamen OR seaman OR airmen OR airman OR soldier* OR troop* OR "enlisted personnel" OR "armed forces" OR "armed services" OR police* OR fireman OR firemen OR firefighter* OR teacher* OR "humanitarian aid worker" OR "relief aid worker" OR "social worker" OR "disaster responder" OR policeman OR policemen OR policewoman OR "law enforcement" OR healthcare OR nurse OR doctor OR frontline OR "health professional" OR "hospital staff" OR "hospital worker" OR work OR job OR occupation OR "emergency service*") Filters: English; Published 2009-2022; all fields.

Web of Science Core Collection

Brennan CJ, *et al. BMJ Open* 2024; 14:e071776. doi: 10.1136/bmjopen-2023-071776

(TS=((mora* injur* OR moral transgression*) AND (Veteran* OR military OR army OR marine* OR "national service" OR "territorial army" OR "armed forces" OR "special forces" OR RAF OR "air force" OR navy OR "service personnel" OR "ex-service personnel" OR submariner* OR sailor* OR seamen OR seaman OR airmen OR airman OR soldier* OR troop* OR "enlisted personnel" OR "armed forces" OR "armed services" OR police* OR fireman OR firemen OR firefighter* OR teacher* OR "humanitarian aid worker" OR "relief aid worker" OR "social worker" OR "disaster responder" OR policeman OR policemen OR policewoman OR "law enforcement" OR healthcare OR nurse OR doctor OR frontline OR "health professional" OR "hospital staff" OR "hospital worker" OR work OR job OR "emergency service" OR occupation)) and Article (Document Types) and English (Languages) and 2009-2022 (Publication Years)

Sage Journals Online

[[All "moral injur*"] OR [All "transgressive act*"] OR [All "moral transgression*"]] AND [[All veteran*] OR [All military] OR [All army] OR [All marine*] OR [All "national service"] OR [All "territorial army"] OR [All "armed forces"] OR [All "special forces"] OR [All raf] OR [All "air force"] OR [All navy] OR [All "service personnel"] OR [All "ex-service personnel"] OR [All submariner*] OR [All sailor*] OR [All seamen] OR [All seaman] OR [All airmen] OR [All airman] OR [All soldier*] OR [All troop*] OR [All "enlisted personnel"] OR [All "armed forces"] OR [All "armed services"] OR [All police*] OR [All fireman] OR [All firemen] OR [All firefighter*] OR [All teacher*] OR [All "humanitarian aid worker"] OR [All "relief aid worker"] OR [All "social worker"] OR [All "disaster responder"] OR [All policeman] OR [All policemen] OR [All policewoman] OR [All "law enforcement"] OR [All healthcare] OR [All nurse] OR [All doctor] OR [All frontline] OR [All "health professional"] OR [All "hospital staff"] OR [All "hospital worker"] OR [All work] OR [All occupation] OR [All "emergency service*"] OR [All job]]
Filters: Research articles, Published 2009-2022.

ScienceDirect

all = ("moral injury" OR "morally injurious" OR "moral transgression" OR "transgressive act") AND (veteran OR police OR job OR healthcare OR occupation). Filters: Research articles; Published 2009-2022.

Scopus

TITLE-ABS-KEY (("mora*injur*" OR "transgression") AND (veteran* OR police OR military OR "law enforcement" OR healthcare OR occupation)). Filters: DOCTYPE “ar”; Published 2009-2022; Language "English".

Google Scholar

All in the title: "moral injury" . Filters: = Published 2009-2022; Language "English".

<u>PTED search strategy</u>

APA PsycINFO (EBSCOhost interface)

((PTED or “post traumatic embitterment disorder” OR embittered OR embitterment)) AND ((Veteran* OR military OR army OR marine* OR "national service" OR "territorial army" OR "armed forces" OR "special forces" OR RAF OR "air force" OR navy OR "service personnel" OR "ex-service personnel" OR submariner* OR sailor* OR seamen OR seaman OR airmen OR airman OR soldier* OR troop* OR "enlisted personnel" OR "armed forces" OR "armed services" OR police* OR fireman OR firemen OR firefighter* OR teacher* OR "humanitarian aid worker" OR "relief aid worker" OR "social worker" OR "disaster responder" OR policeman OR policemen OR policewoman OR "law enforcement" OR healthcare OR nurse OR doctor OR frontline OR "health professional" OR "hospital staff" OR "hospital worker" OR work OR occupation OR job OR emergency service*)).
Filters - Published: 2003-2022; Expanders - Apply equivalent subjects; Narrow by Language: - English’; Exclude Dissertations; Search modes - Find all my search terms.

PubMed

(“post traumatic embitterment disorder” OR PTED OR embittered OR embitterment) AND (Veteran* OR military OR army OR marine* OR "national service" OR "territorial army" OR "armed forces" OR "special forces" OR RAF OR "air force" OR navy OR "service personnel" OR "ex-service personnel" OR submariner* OR sailor* OR seamen OR seaman OR airmen OR airman OR soldier* OR troop* OR "enlisted personnel" OR "armed forces" OR "armed services" OR police* OR fireman OR firemen OR firefighter* OR teacher* OR "humanitarian aid worker" OR "relief aid worker" OR "social worker" OR "disaster responder" OR policeman OR policemen OR policewoman OR "law enforcement" OR healthcare OR nurse OR doctor OR frontline OR "health professional" OR "hospital staff" OR "hospital worker" OR work OR job OR occupation OR "emergency service"). Filters: English; Published 2009-2022; all fields.

Web of Science Core Collection

Brennan CJ, *et al. BMJ Open* 2024; 14:e071776. doi: 10.1136/bmjopen-2023-071776

(TS=("post traumatic embitterment disorder" OR PTED OR embittered OR embitterment) AND TS=(Veteran* OR military OR army OR marine* OR "national service" OR "territorial army" OR "armed forces" OR "special forces" OR RAF OR "air force" OR navy OR "service personnel" OR "ex-service personnel" OR submariner* OR sailor* OR seamen OR seaman OR airmen OR airman OR soldier* OR troop* OR "enlisted personnel" OR "armed forces" OR "armed services" OR police* OR fireman OR firemen OR firefighter* OR teacher* OR "humanitarian aid worker" OR "relief aid worker" OR "social worker" OR "disaster responder" OR policeman OR policemen OR policewomen OR "law enforcement" OR healthcare OR nurse OR doctor OR frontline OR "health professional" OR "hospital staff" OR "hospital worker" OR work OR occupation OR job OR emergency service*)) and Article (Document Types) and English (Languages) and 2003-2022 (Publication Years)

Sage Journals Online

[[All "post traumatic embitterment disorder"] OR [All pted]] OR [All "chronic embitterment"]] AND [[All veteran*] OR [All military] OR [All army] OR [All marine*] OR [All "national service"] OR [All "territorial army"] OR [All "armed forces"] OR [All "special forces"] OR [All raf] OR [All "air force"] OR [All navy] OR [All "service personnel"] OR [All "ex-service personnel"] OR [All submariner*] OR [All sailor*] OR [All seamen] OR [All seaman] OR [All airmen] OR [All airman] OR [All soldier*] OR [All troop*] OR [All "enlisted personnel"] OR [All "armed forces"] OR [All "armed services"] OR [All police*] OR [All fireman] OR [All firemen] OR [All firefighter*] OR [All teacher*] OR [All "humanitarian aid worker"] OR [All "relief aid worker"] OR [All "social worker"] OR [All "disaster responder"] OR [All policeman] OR [All policemen] OR [All policewoman] OR [All "law enforcement"] OR [All healthcare] OR [All nurse] OR [All doctor] OR [All frontline] OR [All "health professional"] OR [All "hospital staff"] OR [All "hospital worker"] OR [All work] OR [All occupation] OR [All job] OR [All "emergency service*"]]. Filters: Research articles, Published 2003-2022.

ScienceDirect

("post traumatic embitterment disorder" OR pted OR embitterment OR embittered) AND (veteran OR police OR "law enforcement" OR healthcare OR occupation). Filters: Research articles; Published 2003-2022.

Scopus

ALL ((pted OR "post traumatic embitterment disorder" OR embittered OR embitterment) AND (veteran* OR police OR military OR job OR healthcare OR work OR occupatio n)). Filters: DOCTYPE “ar”; Published 2003-2022; Language "English".

**Google Scholar**
All: pted OR embitterment OR "post traumatic embitterment disorder". Filters: = Published 2009-2022; Language "English".

Brennan CJ, *et al. BMJ Open* 2024; 14:e071776. doi: 10.1136/bmjopen-2023-071776

Supplementary Table 2.
Data extraction table for studies included in the systematic review

Author (Year)	Study Design	N_a	Occupation	Sampling method	Mean age	% Male	% White	Location	RR	Measurement tool	Associations (+) significant positive correlation (-) significant negative correlation (0) not significant	Prevalence N (%)	Total M(SD)	Hoy	Conflict of Interest
PTED only studies															
Dunn & Sensky. 2018	Cross-sectional	79	Healthcare NHS staff.	Convenience Occupational health department October 2015-March 2016.	44 (12)	33%	NR	UK	NR	PTED scale 1.6+ and 2+	(Mental) health<br>Depression (HADS) (+)<br>Individual and/or work characteristics<br>Affective rumination (WRRQ) (+)<br>Detachment (WRRQ) (-)<br>Problem solving pondering (WRRQ) (0)<br>Positive beliefs about rumination (PBRS) (0)<br>Sickness absence (0)<br>Demographics<br>Gender (0)	1.6 cut off: 37 (47%)<br>2 cut off: 28(35%)	1.5 (1.1)	5	NR
Linden & Rotter 2019	Cross-sectional	102	Unemployed<br>Duration > 1 year (32.4%)	Convenience	36.6 (12.1)	59%	NR	Germany	NR	PTED scale 2+	(Mental) health<br>Psychological distress (GHQ-28) (+)<br><br>Individual and/or work characteristics<br>Feelings of injustice (+)<br>Worse appraisal of unemployment (+)<br>Duration of unemployment (+)<br>Demographics<br>Gender (0)<br>Age (+)	Cut off 2: 26 (25.5%)	1.24 (0.92)	5	None

Michailidis & Cropley. 2018	Longitudinal (T1)	352	Multiple.	Convenience	NR	NR	NR	Cyprus	NR	PTED scale 1.6+	(Mental) health<br>Negative affect (+)<br>Individual and/or work characteristics<br>Organisational procedural justice (OJP) (-)<br>Organisational distributive justice (OJP) (-)<br>Organisational interpersonal justice (OJP) (-)<br>Organisational informational justice (OJP) (-)<br>Supervisory control (+)<br>Job demands (0)	Cut off 1.6: 55 (35%)	1.28 (0.92)	5	None
Sensky, Salimu, Ballard & Pereira. 2015	Cross sectional	236	NHS staff, primarily nurses (N = 67, 28%) and healthcare assistants (n = 49, 21%). 45% worked for the trust for <5 years.	Convenience September-November 2009 Represents sample frame.	NR	45%	NR	UK.	NR	PTED scale 1.6+	Individual and/or work characteristics<br>Work attitudes - felt obligation (0)<br>Work attitudes - organisational support (-)<br>Work attitudes - employer-employee reciprocity (0)<br>Work attitudes - work effort (0)<br>Work attitudes - procedural justice (-)<br>Management referral to occupational health (+)<br>Certified sickness absence (+)	Cut off 1.6: 68 (28.81%)<br>Cut off 2: 43 (18.2%)<br>Cut off 2.5: 21 (8.9%)	1.11(0.97)	4	None

											Uncertified sickness absence (+)<br>Work stress - demands (HSE) (0)<br>Work stress - control (HSE) (0)<br>Work stress - management support (HSE) (-)<br>Work stress - peer support (HSE) (-)<br>Work stress – relationships (HSE) (0)<br>Work stress - role (HSE) (-)<br>Work stress – change (HSE) (-)<br>Demographics:<br>Gender (0)<br>Age (0)<br>Years since qualifying (0)<br>More years in present job (+)				
Sabic, Sabic, & Baltic-Mujanovic, 2018	Cross sectional	87	War veterans of the war in Bosnia and Herzegovnia (1992-1995). Average time since they were a soldier M = 23.86 years.	Convenience	51.42 (5.98)	100%	NR	Bosnia and Herzegovina	NR	PTED scale 1.6+, 2+ and 2.5+	NR	Cut off 1.6: 15 (17.24%)<br>Cut off 2: 9 (10.34%)<br>Cut off 2.5: 1 (1.15%)	0.75(0.73)	5	None

Michailidis & Cropley. 2017	Cross sectional	337	Multiple	Convenience	36.87 (12.4)	26.1 %	NR	NR	NR	PTED scale 1.6+, 2+ and 2.5+	(Mental) health<br>Negative affect (PANAS) (+)<br>Positive affect (PANAS) (-)<br><br>Individual and/or work characteristics<br>Affective rumination (WRRQ) (+)<br>Problem solving pondering (WRRQ) (+)<br>Detachment (WRRQ) (-)<br>Job demands (JCQ) (0)<br>Workplace social support (-)<br>Organisational procedural justice (OJP) (-)<br>Organisational distributive justice (OJP) (-)<br>Organisational interpersonal justice (OJP) (-)<br>Organisational informational justice (OJP) (-)<br>Supervisory control (WCOWP) (+)<br><br>Demographics<br>Gender (+)<br>Age (0)<br>Dependent children (0)	Cut off 1.6: 253 (75%)<br>Cut off 2: 209 (62%)<br>Cut off 2.5: 152 (45%)	2.44(.96)	5	None

											Hours worked per week (+)<br>Years in current job (+)				
Karatuna & Gok 2014	Cross-sectional	397	Public sector, social security institutions<br>Non-managerial position = 83.1%	Convenience	NR	57.2 %		Turkey	39.1 1%	PTED scale 1.6+, 2+, 2.5+	Individual and/or work characteristics<br>Workplace bulling (NAQ-R) (+)<br>Victimisation from bullying (+)<br>Victims of bulling (+)	Cut off 1.6: 106 (26.7%)<br>Cut off 2: 74 (18.6%)<br>Cut off 2.5: 41 (10.3%)	1.00(1.04)	5	NR
Rubab & Tariq 2022	Cross-sectional	300	Private school teachers.	Purposive.	31 (7.9)	50%	NR	NR	NR	PTED scale 1.6+, 2+, 2.5+	(Mental) health<br>Depression (DASS-21) (+)<br>Stress (DASS-21) (+)<br>Anxiety (DASS-21) (+)<br>Depression, stress, and anxiety total (DASS-21) (+)<br><br>Individual and/or work characteristics<br>Generalised self-efficacy (GSES) (-)	Cut off 1.6: 121 (40.3%)<br>Cut off 2: 77 (25.7%)<br>Cut off 2.5: 26 (8.7%)	1.44 (0.70)	5	NR
Saleem, Bashir & Abrar, 2022	Longitudinal (T2)	398	Public sector university academics.	Simple random sampling.	44.5	64%	NR	Pakistan	73 %	PTED scale (mean only)	(Mental) health<br>Employee well-being (-)<br>Individual and/or work characteristics	NR	3.16 (1.12)	5	None

Shin & You 2022	Cross-sectional	1074	Unemployed.	Convenience	47.1 (13.6)	47.6 %	NR	South Korea	NR	PTED scale 1.6+, >1.6 ≥ 2, 2.5+	Leaders' ethical behaviours (-)<br>Employee core self-evaluation (-)<br>Individual and/or work characteristics<br>Work types (0)<br>Unemployment duration (0)<br>Unemployment route (0)<br>Negative life events (+)<br>General belief in a just world (BJW) (-)<br>Personal BJW (-)<br>Demographics<br>Gender (0)<br>Age (+)<br>Marital status (divorced/separated) (+)<br>Educational level (-)<br>Income (-)	Cut off 1.6: 581 (54.1%)<br>Cut off 1.6 - 2.5: 479 (44.6%)<br>Cut off 2.5: 102 (9.5%)	1.64 (0.70)	5	None
PTED and moral injury study															
Brennan, McKay & Cole, 2022	Longitudinal (T1)	400	Health and social care<br>Allied health (14.5%)<br>Primary care practitioner (15.8%)<br>Managerial (10.3%)<br>Health info/science (9.3%)	Convenience September-October 2020.	36.84 (10.7)	24.5 %	63%	UK	89.5 %	MIES 4+ (slightly agree)<br>3 subscales (transgressions-others, transgressions-self, betrayal)<br><br>PTED 1.6+ and 2+ and 2.5	Transgressions-self<br>(Mental) health:<br>Current mental health diagnoses (0)<br>Individual and/or work characteristics<br>Exposure to occupational stressors (+)<br>Pessimism (LOTR) (+)<br>Consideration of immediate	MIES:<br>Overall %<br>289 (72.3%)<br>Per subscale %<br>Transgressions-others: 213 (53.3%)<br><br>Transgressions-self: 131 (32.8%)<br>Betrayal: 227 (56.8%)	MIES:<br>Overall M/SD<br>2.55(1.13)<br>Subscale M/SD<br>Transgressions-others: 2.98(1.48)<br><br>Transgressions-self: 2.17(1.22)<br>Betrayal: 2.77(1.38)	2	ESRC and University funding

	Psychological (6.8%)<br>Corporate (16.3%)<br>Clinical support (26.3%)	consequences (CFC-14) (+)<br>Consideration of future consequences (CFC-14) (0)<br>Resilient coping style (BRCS) (0)<br>Self-esteem (RSES) (-)<br>Procedural justice (BJW) (-)<br>Distributive justice (BJW) (0)<br>Social desirability (-)<br>Demographics *<br>Age (0)<br>Gender (0)<br>Professional role (0)<br>Ethnicity (0)<br>Years in current role (0)<br>Transgressions-others<br>(Mental) health:<br>Current mental health diagnoses (0)<br>Individual and/or work characteristics<br>Exposure to occupational stressors (+)<br>Pessimism (LOTR) (+)<br>Consideration of immediate consequences (CFC-14) (0)<br>Consideration of future consequences (CFC-14) (0)	MIES per item %:<br>Item 1: 179 (44.8%)<br>Item 2: 167 (41.8%)<br>Item 3: 76 (19%)<br>Item 4: 94 (23.5%)<br>Item 5: 83 (20.8%)<br>Item 6: 78 (19.5%)<br>Item 7: 161 (40.3%)<br>Item 8: 124 (31%)<br>Item 9: 158 (39.5%)<br>PTED overall %:<br>1.6 cut off: 121 (30.3%)<br>2 cut off: 77 (19.3%)<br>2.5 cut off: 30 (7.5%)	PTED:<br>Overall M/SD 1.12(0.90)

Resilient coping style (BRCS) (+)
Self-esteem (RSES) (-)
Procedural justice (BJW) (-)
Distributive justice (BJW) (0)
Social desirability (-)
Betrayal:
Mental health:
Current mental health diagnoses (+)
Individual and/or work characteristics
Exposure to occupational stressors (+)
Pessimism (LOTR) (+)
Consideration of immediate consequences (CFC-14) (+)
Consideration of future consequences (CFC-14) (0)
Resilient coping style (BRCS) (0)
Self-esteem (RSES) (-)
Procedural justice (BJW) (-)
Distributive justice (BJW) (-)
Optimism (LOTR) (-)
Social desirability (-)
PTED:
Mental health:

	Current mental health diagnoses (+)<br>Individual and/or work characteristics<br>Exposure to occupational stressors (+)<br>Pessimism (LOTR) (+)<br>Consideration of immediate consequences (CFC-14) (+)<br>Consideration of future consequences (CFC-14) (0)<br>Resilient coping style (BRCS) (0)<br>Self-esteem (R-SES) (-)<br>Procedural justice (BJW) (-)<br>Distributive justice (BJW) (-)<br>Optimism (LOTR) (-)<br>Social desirability (-)<br>Demographics<br>Age (+)<br>*PTED and all MIES subscales
Moral injury only studies	

Bryan et al., 2016<br><br>Sample 2	Cross-sectional	935	Serving military personnel. Army national guard (84%).	Convenience	27.05 (8.11)	82.3 %	57.4%	USA	NR	MIES 6+ (strongly agree)<br>3 subscales (transgressions-others, transgressions-self, betrayal)	Individual and/or work characteristics<br>Transgressions-others subscale:<br>Pre-deployment stressors (DDRI) (+)<br>Combat experiences (DDRI) (+)<br>Aftermath of battle (DDRI) (+)<br>Transgressions-self subscale:<br>Pre-deployment stressors (DDRI) (0)<br>Combat experiences (DDRI) (0)<br>Aftermath of battle (DDRI) (0)<br>Betrayal subscale:<br>Pre-deployment stressors (DDRI) (+)<br>Combat experiences (DDRI) (0)<br>Aftermath of battle (DDRI) (0)<br>Demographics<br>Gender (0)	Overall %<br>NR<br>Per subscale %<br>Cut off 6<br>Transgressions-others: 363 (38.8%)<br>Transgressions-self: 508 (54.3%)<br>Betrayal: 540 (47.8%)	Overall M/SD<br>NR<br>Subscale M/SD<br>Transgressions-others: 4.32(1.67)<br>Transgressions-self: 4.92(1.51)<br>Betrayal: 4.63(1.63)	5	None
Currier, Holland, Drescher et al., 2015<br><br>Sample 1	Cross-sectional	131	Veterans. Deployed to Iraq and/or Afghanistan<br>Marine Corps (38.32%), Active-Duty Army (35.1%)	Convenience	28.47 (5.87)	88%<br><br>Consistent with military demographic trends	26%<br><br>Consistent with ethnic diversity of the region of study	USA	NR	MIQ-M<br>20 items<br>Unidimensional.<br>Average 3.02 years since event (returning from war zone).	(Mental) health<br>Suicide risk (SQB-R) (0)<br>PTSD (PCL-C) (+)<br>Depression (PHQ-9) (+)<br>Individual and/or work characteristics:<br>Combat exposure (CES) (+)	Overall<br>NR<br>Per subscale<br>NR	Overall M/SD<br>32.4(12.8)<br>Subscale M/SD<br>NR	3	NR

			and Navy (22.9%).								Work and social adjustment (WSAS) (+)<br>Active duty and marine's vs other service branches (+)<br>Deployments (+)<br>Demographics<br>Older age (+)<br>Gender (0)<br>Ethnicity (0)				
Currier, Holland, Rojas-Flores et al., 2015	Cross sectional.	257	Teachers. Classroom teachers (83.7%). 13.7% administrators e.g., principal (13.7%), and both classroom teacher and school administrator (2.7%) Average time in role 15.44 (10.29).	Convenience March 2012	42.02 (13.12)	31.4 %	NR	El Salvador	95 %	MIQ-T 12 items Unidimensional.	(Mental) health<br>PTSD Reexperiencing (LASC) (+)<br>PTSD Avoidance (LASC) (+)<br>PTSD Hyperarousal (LASC) (+)<br>Physical burnout (SMBM) (+)<br>Cognitive burnout (+)<br>Emotional burnout (+)<br>Individual and/or characteristics<br>Violence exposure (SECV) (+)<br>Meaning made of stress comprehensibility (ISLES) (-)<br>Meaning made of stress footing in the world (ISLES) (-)	Overall NR<br>Per subscale NR	Overall M/SD 13.92(6.71)<br>No subscales	4	NR

Currier et al., 2017<br><br>Sample 2	Cross-sectional	624	Veterans. 1+ deployments to a war zone during their service.<br><br>49.2% (Army), 10.6% (Marine Corps), 22.1% (Navy), 21.8% (Air Force), 0.8% (Coast Guard).<br><br>97.4% (Active Duty), 11.9% (Military Reserves), 6.3% (National Guard	Convenience	NR	81.4 %	10.9%	USA	7.3 %	EMIS<br>2 subscales (self-directed MI and other-directed MI).<br>MIQ-M<br>7 items<br>1 subscale (causes)<br>MIES<br>2 subscales (perpetration and betrayal)	(Mental) health<br>PTSD (PCL 5) (+)<br>Depression (PHQ-8) (+)<br>Alcohol misuse (AUDIT-C) (+)<br>Anger (DAR-5) (-)<br>Guilt (PFQ-2) (+)<br>Shame (PFQ-2) (+)<br>Individual and/or work characteristics<br>Combat exposure – Survival threat (DDRI) (+)<br>Combat exposure – Aftermath of violence (DDRI) (+)	Overall NR<br>Per subscale NR	EMIS:<br>Overall M/SD 32.14(14.84)<br>Subscale M/SD<br>Self-MI 14.92(7.49)<br>Other-MI 17.22(8.23)<br>MIQ-M<br>Overall M/SD NR<br>Subscale causes M/SD 9.29(3.41)<br>MIES<br>Overall M/SD NR<br>Subscales<br>Perpetration 2.68(1.50)<br>Betrayal 2.80(1.66)	5	NR

Forkus, Juliana & Weiss. 2019	Cross sectional	203	Veterans. Deployed to Iraq or Afghanistan. Army (52.20%), Air Force (19.20%), Navy (15.80%), and Marine Corps (12.80%).	Convenience	35.08	77.30%	70.40%	NR	29.17%	MIES Unidimensional	(Mental) health<br>PTSD (PCL-5) (+)<br>Depression (PHQ) (+)<br>Alcohol misuse (+)<br>Drug misuse (+)<br>Deliberate self-harm history (+)<br>Deliberate self-harm versatility (+)<br>Individual and/or work characteristics<br>Self-compassion (-)	NR	Overall M/SD 2.95(1.41)	5	NR
Frankfurt et al., 2018.	Longitudinal secondary data analysis. MIES reported at T2	310	Veterans<br><br>Post 9/11.<br><br>Army (90.3%). Active duty (96.8%).<br><br>Average years in role 13.50 (7.61)	Convenience	40.67 (8.55).	76%	57%<br><br>Reflected geographic area	USA	NR	MIES 2 subscales (perpetration and betrayal)	(Mental) health<br>Military sexual trauma (DDRI) (+) for perpetration and betrayal subscales<br>Depression (+) for perpetration and betrayal subscales<br>PTSD (+) for perpetration and betrayal subscales<br>Shame (+) for perpetration and betrayal subscales<br>Guilt (+) for perpetration and betrayal subscales<br>Individual and/or work characteristics<br>Combat exposure (0)*<br>Combat exposure (+)**<br>*betrayal<br>**perpetration	NR	Overall M/SD NR<br>Subscales<br>Perpetration 3.00(1.40)<br>Betrayal 3.41(1.64)	4	None

Griffin et al., 2020<br><br>Narrative synthesis only	Cross sectional.	498	Military service members and veterans<br><br>Army (22.49%), Navy (17.47%), Air Force (45.78%), Marines (11.24%), Coastguard (2.61%). Active Duty (29.52%).	Convenience	NR	73.90 %	NR	USA	NR	MIES<br>5 of 9 items to create 5 profiles	NR due to using 'profiles'	Overall %<br>NR<br>Subscales<br>No moral distress (42% of sample)<br>Moral Distress-Other (19%)<br>Witnessing-Only (16%)<br>Moral distress-Self (8%)<br>Moral distress-Self and Other (15%)	NR	5	NR
Jinkerson, Battles. 2019.	Cross sectional	72	Veterans Vietnam and post-Vietnam eras.<br><br>Army (59.7%), Navy (13.9%), Air Force (12.5%), Marine Corps (12.5%)	Convenience	NR	87.5 %	45.8%	USA	76 %	MIQ-M<br>20 items<br>Unidimensional	(Mental) health<br>Meaning in life (-)<br>Depression (PHQ-2) (+)<br>Anxiety (GAD-2) (+)<br>PTSD (PCL-5) (+)<br>PTSD Reexperiencing (PCL-5) (+)<br>PTSD avoidance (PCL-5) (+)<br>PTSD Negative emotions (PCL-5) (+)<br>PTSD hyperarousal/hypervigilance (PCL-5) (+)<br>Global guilt (TRGI) (+)<br>Distress (TRGI) (+)	Per item %<br>(top 5 items)<br>Betrayal by military/political leaders N = 32 (44.5%)<br>experiencing events that were chaotic and beyond control N = 32 (44.4%)<br>failing to save the life of someone in war N = 24 (33.3%)<br>surviving when others	Overall M/SD<br>3.48(0.35)<br>Subscales<br>NR	4	NR

											Guilty cognitions (TRGI) (+)<br>Wrongdoing (TRGI) (+)<br>Lack of justification (TRGI) (0)	didn't N = 21 (29.2%) feeling betrayal by trusted civilians N = 32 (29.2%)			
Jordan et al., 2017.	Longitudinal. Data from 1 month post deployment reported here and from cohort 4 from the parent study.	867	Currently serving active-duty marines from a single infantry battalion engaged in heavy ground combat while deployed to Afghanistan.	Convenience	NR	NR	NR	USA	NR	MIES 4+ (slightly agree)<br>Subscales: 2 subscales (transgressions-self and betrayal)	(Mental) health<br>PTSD (PLC-S) (+)<br>Guilt/shame (PANAS) (+) *<br>Anger (PANAS) (+) *<br>Individual and/or characteristics<br>Dissociation (PDEQ) (+) *<br>Combat exposure (CES) (+) *<br>* transgressions-self and betrayal subscales	Overall %<br>NR<br>Per subscale<br>Transgressions-self 214 (24.1%)<br>Betrayal 252 (28.4%)<br>Transgressions-self and betrayal: 333 (37.5%)<br>Per item %<br>Item 1: 165 (18.6%)<br>Item 2: 89 (10%)<br>Item 3: 128 (14.4%)<br>Item 4: 103 (11.6%)<br>Item 7: 179 (20.2%)<br>Item 8: 133 (15%)<br>Item 9: 132 (14.9%)	Overall M/SD<br>NR, only used below subscales<br>Subscales<br>Transgressions-self = 1.87(1.17)<br>Betrayal = 2(1.23)	5	NR

Lee Aldwin, Kang. 2022	Cross sectional.	367<br><br>354 responded to MIES	Veterans<br><br>Deployed to the Vietnam war in 1664-1973.<br><br>Army (88%).	Convenience	72 (2.66)	100%	NR	Korea	37.4 %	MIES 3+ (sometimes) and 4+ (often)<br>5-point scale not 6 (1=never, 2=seldom, 3=sometimes, 4=often, 5=always).<br><br>3 subscales (transgressions-others, transgressions-self, betrayal)<br><br>40-50 years since event	(Mental) health<br>PTSD (Checklist for DSM-5) (+)<br>Depression (BSI) (+)<br>Anxiety (BSI) (+)<br><br>Individual and/or work characteristics<br>Optimism (LOT) (-)<br>Unit cohesion (-)<br>Homecoming experience (DDRI-2) (0)<br>Combat exposure (CES) (+)<br>Malevolent environments (CES) (+)<br>Perceived threat (CES) (+)<br>Demographics<br>Age (0)<br>Education (-)<br>Marital status (0)<br>Income (0)	Overall %<br>Cut off 4+: 104(29.4%)<br>Cut off 3+: 176(49.7%)<br>Subscales %<br>Transgressions-others cut off 4+: 57(16.1%)<br>Transgressions-self cut off 4+: 53(14.9%)<br>Betrayal cut off 4+: 59(16.7%)<br>Transgressions-others cut off 3+: 130(36.7%)<br>Transgressions-self cut off 3+: 115(32.5%)<br>Betrayal cut off 3+: 121(34.2%)	Overall M/SD<br>1.80(0.80)<br>Subscales<br>Transgressions-others: 2.04(1.04)<br>Transgressions-self: 1.69(0.86)<br>Betrayal: 1.80(0.91)	5	None
Ogle, Reichwald, Rutland 2018<br><br>Narrative synthesis only	Cross sectional	356	Serving military analysts<br><br>Geospatial intelligence analysts (29%), cryptologic language analysts (54.8%) who	Convenience	NR	70.3 %	NR	USA	NR	MIES<br>10 items instead of 9<br>3 subscales (transgressions-others, transgressions-self, betrayal)	(Mental) health<br>PTSD (PCL-5) (+) *<br>PTSD Avoidance (PCL-5) (+) *<br>PTSD Mood/cognition (PCL-5) (+) *<br>PTSD Hypervigilance (PCL-5) (+) *<br>Reliving (PCL-5) (+) *	Overall %<br>NR<br>Subscales %<br>NR<br>Per item % split by combat exposure level<br>Item 1: None = 9.1%, Low = 21%, Middle =	Overall M/SD<br>NR<br>Subscales M/SD<br>NR	5	NR

provide support to remote combat and global vigilance operations and who have routine exposure to related video, audio, still images, and other media.		Social adjustment (-) *<br>Affect work/home (+) *<br>Individual and/or work characteristics<br>Combat exposure (CES) (+) *<br>Work satisfaction (-) *transgressions-self and betrayal<br>Work satisfaction (0) transgressions-others<br>Combat ambiguity (+) *<br>* Transgressions-others, transgressions-self and betrayal subscales	26%, High = 47.3%<br>Item 2: None = 11.6%, %, Low = 11.6%, Middle = 6.2%, High = 15.4%<br>Item 3: None = 4.7%, %, Low = 29%, Middle = 46.4%, High = 60.5%<br>Item 4: None = 2.3%, %, Low =8.2%, Middle = 5%, High = 11%,<br>Item 5: None = 2.3%, Low = 9.4%, Middle = 4.1%, High = 9.9%<br>Item 6: None = 4.6%, Low =7.1%, Middle = 2%, High = 8.8%<br>Item 7: None = 2.3%, Low =6.9%, Middle = 2%, High = 7.7%, r_s = .109<br>Item 8: None = 11.3%, Low =17.5%,

												Middle = 10.1%, High = 20.9%<br>Item 9: None = 7%, Low =11.7%, Middle = 3%, High = 13.2%<br>Item 10: None = 7%, Low =10.5%, Middle = 5%, High = 7.7%			
Wisco et al., 2017.	Cross sectional.	564	Veterans. Experienced combat. Army 41%.	Epidemiological sampling, recruited from the National Health and Resilience in Veterans Study (NHRVS).	NR	93%	76.2%	USA	25 %	MIES: 5+ (moderate) 3 subscales (transgressions-others, transgressions-self, betrayal) 33.7 years (19.1) since event (last combat deployment)	Individual and/or work characteristics<br>Vietnam war era (0)<br>Military branch (army) (+)<br>Multiple deployments (+)<br>Moderate/heavy combat (+)<br>Demographics<br>Age (0)<br>Gender (0)<br>White race (-)<br>College education (-)<br>Annual income >60k (-)<br>Currently employed (+)	*Reports weighted % and SE<br><br>Overall %<br>223, (41.8%, 2.1%))<br><br>Subscale %<br>Transgressions -Others 126, (25.5%, 1.8%))<br><br>Transgressions -Self 48 (10.8%, 1.3%)<br><br>Betrayal 142 (25.1%, 1.8%)<br><br>Per item<br>Item 1: 115 (23.8%, 1.8%)<br>Item 2: 65, (14.1%, 1.5%)	Overall M/SD 2.11(1.08)<br>Subscales M/SD<br><br>Transgressions -others 2.71(1.66)<br><br>Transgressions -self 1.75(1.16)<br><br>Betrayal 2.19(1.29)	1	None

												Item 3: 37 (8.9%, 1.2%)<br>Item 4: 30 (5.9%, 1%)<br>Item 5: 21 (3.9%, 0.8%)<br>Item 6: 19 (3.6%, 0.8%)<br>Item 7: 107 (18.6%, 1.7%)<br>Item 8: 81 (15%, 1.5%)<br>Item 9: 31 (5%, 0.9%)			
Zerach, Levi-Belz. 2017	Cross sectional	191	Veterans. 100% army.	Convenience<br><br>March-July 2017.	25.39 (2.37)	85.4 %	NR	Israel	86.8 %	MIES 4+ (slightly agree)<br>3 subscales (transgressions-others, transgressions-self, betrayal)<br>Time since deployment 4.36(2.27) years.<br><br>MIQ-M 2+ (seldom)<br>14 causal items<br>1 subscale (causes)	(Mental) health<br>Depression (DAQ) and MIQ-M (0)<br>Depression (DAQ) and MIES (+) *<br>PTSD (PCL-5) and betrayal (+)<br>PTSD (PCL-5) and transgression-others, transgressions-self and MIQ-M (0)<br>Guilt-global (TRGI) and MIQ-M and MIES (+) *<br>Guilt-distress (TRGI) and MIQ-M and MIES (+) *<br>Guilt-cognitions (TRGI) and MIQ-M and MIES (+) *<br>Shame-intrinsic (TSRI) and MIQ-M and MIES (+) *	Overall %<br>92(48.4%)<br>Subscales<br>Transgressions-others 64 (33.7%)<br>Transgressions-self 42 (21.9%)<br><br>Betrayal 59 (31%)<br><br>Per item MIES %<br><br>Item 1: 55 (29%) Item 2: 42 (22%)<br>Item 3: 23 (12%) Item 4: 25 (13%)<br>Item 5: 19 (10%)	Overall M/SD<br>2.00(0.97)<br>Subscales<br>MIES<br>Transgressions-others<br>2.42(1.39)<br>Transgressions-self<br>1.68(1.05)<br>Betrayal<br>2.14(1.22)<br>Subscales<br>MIQ-M<br>MIQ causes<br>1.34(0.34)	4	NR

											Shame-extrinsic (TSRI) and MIQ-M and MIES (+) * Self-disgust-self (TRSI) and MIQ-M and MIES (0) * Self-disgust -ways (TSRI) and betrayal (+), Self-disgust ways (TSRI) and transgressions-self, transgressions-others, and MIQ-M (0) Individual and/or work characteristics Combat exposure (CES) (+) MIQ-M Combat exposure (0) MIES * *transgressions-self, transgressions-other and betrayal subscales	Item 6: 23 (12%) Item 7: 48 (25%) Item 8: 32 (17%) Item 9: 29 (15%) Per item MIQ-M % Item 4: 86 (45.2%) Item 8: 60 (31.4%)			
Papazoglou et al., 2019.	Cross sectional.	390	Service police officers. National Police of Finland. Average years of police experience 16.87(9.11)	Convenience	41.21 (8.42)	73.5 %	100%	Finland	86 %	MIES 5+ (moderately agree) and 6+ (strongly agree) Item 6 not included 2 subscales (perpetration and betrayal)	(Mental) health PTSD (PCL-C) (+) * Compassion fatigue (CSF) (+) * Compassion satisfaction (+) * Individual and/or work characteristics Dark triad (SD3) (+) * *Perpetration and betrayal subscales	Overall % Overall, MI (moderate): 289 (74%) Subscale % Perpetration 152 (39%) (moderate) Betrayal 250 (64%) (moderate) Perpetration 39 (10%) (high)	Overall M/SD NR Subscale M/SD Perpetration 2.61 (1.19) Betrayal 3.77 (.98)	4	NR

													Betrayal 94 (24%) (high)			
Richardson, Chesnut et al., 2020	Longitudinal (T2)	5,227	Veterans. Recently separated from service<br><br>Army (36.8%), Navy (22.4%), Air Force (20.5%), marines (20.2%).	Epidemiological, Recruited from The Veteran Metrics Initiative (TVMI) study.	NR	82.1 %	66.9%	USA	10.67%	MIES 4+ (slightly agree)<br>2 subscales, (MI-self and MI-other)<br>Within 90 days since event (separation from service)	NR		Overall %<br>NR<br>Subscale %<br>MI-Self: 516 (9.87%)<br>MI-Other: 1033 (19.76%)<br>Per item prevalence<br>Item 1: 2432 (46.53%)<br>Item 2: 1496 (28.62%)<br>Item 3: 833(15.94%)<br>Item 4: 750(14.35%)<br>Item 5: 813(15.55%)<br>Item 6: 741(14.18%)<br>Item 7: 1955(37.4%)<br>Item 8: 1472 (28.16%)<br>Item 9: 1039 (19.88%)	Overall M/SD<br>2.24(1.07)<br>Subscale M/SD<br>MI-Self: 1.79(1.25)<br>MI-Other: 2.51(1.40)	2	None

Feinstein, Pavisian, Storm. 2018	Cross sectional	80	Journalists. Years in role 18.28 (8.09)<br><br>months covering the refugee crisis 19.65 (19.41)	Convenience	42.95 (8.44)	58.8 %	70.40%	Nine European and American news organisations	70.2 %	MIES 6 items 1 subscale (perpetration)	(Mental) health<br>PTSD intrusion (IES-R) (+)<br>PTSD avoidance (IES-R) (+)<br>PTSD arousal (IES-R) (+)<br>Depression (BDI-II) (0)<br>Alcohol consumption (0)<br>Individual and/or work characteristics<br>Higher workload in past year (+)<br>Control over resources (-)<br>Guilt (+)<br>Demographics<br>Having children (+) t-test<br>Gender (0)<br>Age (0)<br>Education (0)<br>Marriage (0)<br>Marital status (0)	Overall % NR<br>Subscales % NR	Overall M/SD NR (only one subscale)<br>Subscales Perpetration 2.83(0.93)	5	HR is director of INSI
Sugrue, 2020	Cross sectional	218	K-12 education<br><br>Classroom teacher (50.5%), special education staff (19.3%), Non-classroom instruction	Convenience	42.6 (11.9)	22.9 %	77.1%	USA	<7 %	MIES 4+ (slightly agree)<br>3 subscales (transgressions-others, transgressions-self, betrayal)	(Mental) health<br>Transgressions-others subscale:<br>Personal burnout (CBI) (+)<br>Work burnout (CBI) (+) *<br>Client burnout (CBI) (+)<br>Global guilt (TRGI) (+)<br>Distress Scale (TRGI) (+)	Overall % 191 (85.3%).<br>Subscale %<br><br>Transgressions-others, 175 (80.2%)<br><br>Transgressions-self, 98 (45.4%)	Overall M/SD 3.49(1.24)<br>Subscale M/SD<br>Transgressions-others 4.4(1.5)<br>Transgressions-self, 2.9(1.5)<br>Betrayal 3.8(1.5)	5	NR

al support (10.1%), School social workers (5%), English language learner (ELL) teachers (5%), Specialist teachers (4.6%), School psychologists (3.7%), School counsellors (0.9%), school nurses (0.5%)<br><br>Average years working in education 15.0(10.1)	Hindsight bias (TRGI)<br>Wrongdoing (TRGI)<br>Lack of justification (TRGI) (0)<br>Stress of conscience (internal and external subscales and total (SCQ)) (+)<br>Transgressions-self subscale:<br>Personal burnout (CBI) (+)<br>Work burnout (CBI) (+) *<br>Client burnout (CBI) (0)<br>Global guilt (TRGI) (+)<br>Distress Scale (TRGI) (+)<br>Hindsight bias (TRGI)<br>Wrongdoing (TRGI)<br>Lack of justification (TRGI) (+)<br>Stress of conscience (internal and external subscales and total (SCQ)) (+)<br>Betrayal subscale:<br>Personal burnout (CBI) (+)<br>Work burnout (CBI) (+) *<br>Client burnout (CBI) (+)<br>Global guilt (TRGI) (+)	Betrayal, 148 (68.4%)<br><br>Transgressions -others, 115(52.7%) – moderate (5) cut off

											Distress Scale<br>(TRGI) (+)<br>Hindsight bias<br>(TRGI)<br>Wrongdoing (TRGI)<br>Lack of justification<br>(TRGI) (0)<br>Stress of conscience<br>(internal and external<br>subscales and total<br>(SCQ)) (+)<br>Individual and/or<br>work characteristics<br>Transgressions-others<br>subscale<br>Intention to leave<br>(ILS) (+)<br>Transgressions-self<br>subscale<br>Intention to leave<br>(ILS) (0)<br>Betrayal subscale<br>Intention to leave<br>(ILS) (+)				
Aldridge,<br>Scott,<br>Paskell.<br>2019.	Cross<br>sectional.	104	Veterans.<br><br>British<br>Army,<br>Royal<br>Navy or<br>Royal Air<br>Force and<br>had<br>experience<br>d active<br>deploymen<br>t.	Convenience<br><br>Feb 2017-<br>Nov 2017.	47<br>(10.8)	95.2<br>%	NR	UK	NR	MIQ-M<br>19 items<br>Unidimensi<br>onal.	(Mental) health<br>PTSD (PCL-M) (+)<br>Individual<br>characteristics<br>Guilt proneness<br>(TOSCA)<br>(-)<br>Shame proneness<br>(TOSCA) (0)<br>Demographics<br>Age (0)<br>Gender (0)<br>Length of service (0)<br>Previous therapy (0)	Overall %<br>NR<br>Subscale %<br>NR	Overall M/SD<br>36.75(10.12)<br>Subscale<br>M/SD<br>NR	5	NR

Andrukonis, Potopopova. 2020.	Cross sectional.	153	Animal shelter.<br><br>Shelter director (n=16), assistant director (4), animal shelter supervisor/manager (21), animal control officer (18), kennel attendant/animal care tech (29), customer service representative (13), vet/vet tech/medical (22), behaviour/training (9), volunteer/rescue/community (3), other (22)	Convenience<br><br>Dec 2017-April 2018.	NR	NR	NR	USA	NR	MIES Higher score represents lower moral injury Unidimensional.	Individual and/or work characteristics<br>Shelter type (0)<br>Admission type (0)<br>Euthanizes in job (-)<br>Demographics<br>Months on the job (0)	Overall % NR<br><br>Subscale % NR	Overall M/SD 0.96(4.22)<br>Subscale M/SD NR	5	None

			Years in job M=5.9													
Haight., Sugrue, Calhoun. 2017.	Cross sectional	38	CPS Social worker (n = 13) County attorney (n = 5) Guardians ad litem (n = 5) Student attorneys (n = 10) Law school clinic and parent support staff (n = 5) Years in CPS 8.5(8.6)	Convenience	NR	18%	66%	USA	56.33%	MIES 4+ (slightly agree)<br><br>3 subscales (transgressions-others, transgressions-self, betrayal)	Demographics Age (0) Gender (0) Race (0) Years of experience in CPS (0) Years of education and transgressions-self and betrayal (-)	Overall % NR Subscale %<br><br>Transgressions-others 35(92%)<br><br>Transgressions-self 9(24%)<br><br>Betrayal 12(32%)	Overall M/SD NR Subscale M/SD Transgression-others 5.1(1) Transgression-self 2.7(1.4) Betrayal 3.1(1.2)	5	NR	

Battles et al. 2019.	Cross sectional	380	Currently serving military and veterans that had deployed to 1+ war zones for 3+ months. Navy (43.5% of men, 53.3% of women). No longer serving (67.1% of men, 66.7%) of women. Length of time serving (men 12.88, 10, women, 9.84, 8)	Convenience	35.29 (9.58)	68.42 %	71.9% of men and 61.7% of women	USA	NR	MIQ-M 12 causal items 1 subscale (causes)	(Mental) health<br>Spiritual injury (+)<br>Total alcohol use (AUDIT) in men (+)<br>Total alcohol use in women (0)<br>Alcohol consumption (AUDIT) in men (+)<br>Alcohol consumption (AUDIT) in women (0)<br>Alcohol dependence (AUDIT) in men (+)<br>Alcohol dependence (AUDIT) in women (0)<br><br>Individual and/or work characteristics<br>Combat exposure (CES) (+)<br>Military status (+) (0)<br>Military branch (+)<br><br>Demographics<br>Years in service (0)	Overall % NR<br>Subscale % NR	Overall M/SD NR<br>Subscale M/SD (causes 12 items) 1.26(0.42)	5	NR
Braitman et al., 2018.<br><br>Narrative synthesis only	Cross sectional	328	Current military or veterans with deployed experience Veterans (69.5%) National Guard/Reservists	Convenience	32.42 (7.71)	56.1 %	68.0%	USA	NR	MIQ-M modified 2+ (seldom) 27 PMIE items plus 135 follow up questions (5 per PMIE item)	(Mental) health<br>Depression (CES-D-10) (+)<br>Anxiety (KAS) (+)<br>PTSD (PCL-5) (+)<br>Hazardous alcohol use (AUDIT) (+)<br>Drug abuse (DAST) (+)<br>Drug abuse (DAST) (0) *	Overall % NR<br><br>Subscale % NR<br><br>Per item %<br>Item 7: 216 (65.9%)<br>Item 8: 174 (53%)	Overall M/SD NR<br>Subscale M/SD<br><br>AoW PMIEs 1.77(0.63)<br>PCoW PMIEs 1.99(0.66)<br>LFB PMIEs 2.12(0.78)	5	None

(19.5%) Active-duty members (9.5%) Army (46.3%) Navy (29.9%) Air force (7.9%) Marines (5.5%) National guard (4%) Reserves (4.6%).	3 subscales. (atrocities at war (AoW); psychological consequences of war (PCoW), leadership failure or betrayal (LFB).	All subscales *LFT and AoW	Item 10: 182(55.5%) Item 11: 155(47.3%) Item 15: 181(55.2%) Item 16: 150(45.7%) Item 17: 178(54.3%) Item 19. 191(58.2%) Item 22: 179(54.6%) Item 23: 170(51.8%) Item 24: 198(60.4%) Item 26: 192(58.5%) Item 27: 183(55.8%) Item 6: 208 (63.4%) Item 9: 231 (70.4%) Item 13: 233 (71%) Item 18: 227(69.2%) Item 21: 220(67.1%) Item 25: 199(60.7%) Item 1: 218(66.5%) Item 2: 225(68.6%) Item 4.	AoW defining characteristics 2.21(0.67) PCoW defining characteristics 2.14(0.66) LFB defining characteristics 2.12(0.67) All defining characteristics 2.11(0.66)

Brennan CJ, *et al. BMJ Open* 2024; 14:e071776. doi: 10.1136/bmjopen-2023-071776

												239(72.9%)<br>Item 3:<br>200(61%)<br>Item 5:<br>168(51.2%)<br>Item 12:<br>208(63.4%)<br>Item 14:<br>174(53%)<br>Item 20.<br>214(65.2%)			
Lancaster, Harris, 2018.	Cross sectional	182	Veterans Deployed to Iraq or Afghanistan<br>Army (61.0%)<br>Air Force (17.0%),<br>Marine Corps (14.8%),<br>Navy (7.1%).	Convenience	33.66 (7.23)	80.2 %	78.0%	NR	32.33%	MIES<br>3 subscales (transgressions-others, transgressions-self, betrayal)<br><br>MIQ-M<br>19 items<br>2 subscales (causes and effect)	(Mental) health<br>Anger (DAR-5) (+) *<br>Depression (PHQ) (+) *<br>Alcohol (AUDIT) (+) *<br>PTSD (PCL-5) (+) *<br>Guilt (+) *<br>Shame (+) *<br><br>Individual and/or work characteristics<br>Combat (CES) (+)*<br>CES (0)**<br><br>All subscales<br>*transgressions-others and transgressions-self subscales<br>**betrayal subscale	Overall %<br>NR<br><br>Subscale %<br>NR	Overall M/SD MIES<br>2.75(1.32)<br><br>Subscale M/SD MIES<br>Transgressions-others<br>3.49(1.50)<br><br>Transgressions-self<br>2.45(1.53)<br><br>Betrayal<br>2.67(1.57)<br><br>Overall M/SD MIQ-M<br>33.70(11.90)<br><br>Subscale M/SD MIQ-M<br>Effect<br>11.54(4.21)<br><br>Causes<br>22.19(8.32)	5	NR

Lancaster, 2018	Cross sectional	161	Veterans. Post 9/11<br><br>Army (43.5%), Navy (19.9%), Air Force (16.1%), Marine Corps (13.7%), Coast Guard (6.8%).	Convenience	35.08 (9.62)	71.4 %	73.9%	NR	5.18 %	MIES<br>7 of 9 items<br>2 subscales (transgressions-self and betrayal)	(Mental) health<br>PTSD (PTSD checklist 5) (+) *<br>Depression (PHQ) (+) *<br>Anger (+) *<br>Guilt/shame (SSGS) (+) *<br><br>Individual and/or work characteristics<br>Transgressive acts (TAS) (+) *<br>*transgressions-self and betrayal subscales	Overall %<br>NR<br>Subscales %<br>NR<br>*Range 12.6% to 23.3% on items	Overall M/SD<br>NR (only 2 subscales used)<br><br>Subscales M/SD<br>Transgressions-self, 2.56(1.43)<br>Betrayal 2.72(1.46)	5	NR
Akhtar, Faize, Malik & Tabusam, 2022	Cross-sectional	108	Healthcare<br>Doctors (51.9%)<br>Nurse (26.9%)<br>Paramedics (21.3%)	Purposive<br><br>August 2020-January 2021.	27.67 (6.17)	41.7 %	NR	Pakistan.	NR	MISS-HP 36+	Individual and/or work characteristics<br>Psychological resilience (CD-RISC-10): (-)<br>Demographics<br>Age (0)<br>Experience (-)<br>Work hours (+)<br>Female gender (+)<br>Marital status (0)<br>Hospital type (0)<br>Profession (0)<br>Speciality/department psychiatry (+)<br><br>Work area/duty station (0)	Overall %<br>75 (69.44%)	Overall M/SD<br><br>43.36 (9.64)	6	NR

Qi et al., 2022<br><br>Two studies.	Study 1: Cross-sectional<br>Study 2: Cross-sectional	Study 1: 3006<br>Study 2:<br><br>3465	Study 1: Healthcare Nurse (18%)<br>Internal medicine (19.4%)<br>Obstetrics/ gynaecology/paediatrics (34.7%)<br>Surgery (9.6%)<br>Psychiatry (11.4%)<br>Other<br>Study 2: Healthcare Nurse (14.7%)<br>Internal medicine (44.7%)<br>Obstetrics/ gynaecology/paediatrics (21.1%)<br>Surgery (4.9%)<br>Psychiatry (8.5%)<br>Other (6.1)	Study 1: Snowball. March-April 2020.<br>Study 2: Snowball. March-April 2021	Study 1: NR<br>Study 2:<br><br>NR	Study 1: 34.9 %<br>Study 2: 24.6 %	Study 1: NR<br>Study 2: NR	Study 1: China<br>Study 2: China	Study 1: 75 %<br>Study 2:<br><br>86 %	Study 1: MISS-HP 36+ EMIS-SF<br>Study 2: MIISS-HP 36+	Study 1: (Mental) health Depression (PHQ) (+)<br>Anxiety (GAD-7) (+)<br>Burnout emotional exhaustion (MBI-HSMP) (+)<br>Burnout reduced personal accomplishment (MBI-HSMP) (-)<br>Burnout depersonalisation (MBI-HSMP) (+)<br>Well-being (SFI) (-)<br>* EMIS and MISS-HP<br>Study 2: NR at univariate level.	Study 1: Overall, MISS-HP % 1695 (56.4%)<br>Study 2: Overall, MISS-HP % 1270 (36.7)	Study 1: MISS-HP overall: 46.9 (12.7)<br>EMIS-SF overall: 2.55 (0.80)<br>Study 2: MISS-HP: 45.9(11.3)	5	None

Amsalem et al., 2021	Longitudinal	350	Healthcare<br>Nurses (68%)<br>Physicians (15%)<br>Emergency medical technicians (9%)<br>Physical therapists (3%)<br>Pharmacists (2%)<br>Social workers and other therapists (1%)<br>Other (2%)	Convenience<br>September-December 2020	34.8 (11.5)	26%	73%	USA	NR	MIES: 3+ (agree)<br>Likert scale 1 (disagree), 2 (strongly disagree) 3 (agree) 4 (strongly agree)<br><br>3 subscales (transgressions-others, transgressions-self, betrayal)	(Mental) health<br>Depression (PHQ) (+)<br>Anxiety (GAD-7) (+)<br>Suicidal ideation (+)<br>PTSD (PC-PTSD) (+)<br>Demographics<br>Age (0)<br>Experience (-)<br>Work hours (+)<br>Female gender (+)<br>Marital status (0)<br>Hospital type (0)<br>Profession (0)<br>Speciality/department psychiatry (+)<br>Work area/duty station (0)	Overall %<br>267 (76.29%)<br>Per subscale %<br>Transgressions-others: 215 (61.43%)<br><br>Transgressions-self: 101 (28.86)<br>Betrayal: 205 (58.57%)<br>Per item:<br>Item1: 192 (55%)<br>Item 2: 171 (49%)<br>Item 3: 66 (19%)<br>Item 4: 68 (19%)<br>Item 5: 71 (20%)<br>Item 6: 72 (21%)<br>Item 7: 157 (45%)<br>Item 8: 111 (32%)<br>Item 9: 152 (43%)	Overall M/SD<br>1.98 (0.72)<br>Subscale M/SD<br>Transgressions-others: 2.41(1.04)<br><br>Transgressions-self: 1.63(0.82)<br>Betrayal: 2.15(0.92)	5	None

Perez, Larson & Bair 2021	Cross-sectional	85	Veterans Served between 2001-2014. Army (63.5%) Marines (8.2%) Navy (10.6%) Air Force (15.3%) Coast Guard (2.4%)	Convenience	NR	68.2 %	78.8%	USA	NR	EMIS 2 subscales (self-directed MI and other-directed MI)	Individual and/or work characteristics Fairness (MFQ) (0) Loyalty (MFQ) (+) * Loyalty (MFQ) (0) ** Authority (MFQ) (0) Sanctity (MFQ) (0) *Self-MI **Other-MI	Overall % NR Per subscale NR	Overall M/SD 2.45(0.98) Subscale M/SD Self-MI 2.16(1.08) Other-MI 2.80(1.06)	5	NR
Benatov, Zerach & Levi-Belz 2022	Cross-sectional	296	Health and social care Doctor (34.1%) Nurse (including midwives) (44.4%) Social and psychological care (17.4%) Clinical support (5.9%)	Convenience	40.28 (10.8)	22.4 %	NR	Israel	NR	MIES 4+ (slightly agree) 3 subscales (transgressions-others, transgressions-self, betrayal) MISS-HP 36+	(Mental) health Depression (PHQ) (+) Anxiety (GAD-7) (+) Individual and/or work characteristics Thwarted belonginess (INQ) (+) *For MISS-HP and MIES scales	MIES: Overall %: NR Per subscale % Transgressions-others: 146 (49.3%) Transgressions-self: 94 (31.8%) Betrayal: 184 (62.2%) Per item %: Item 1: 135 (45.5) Item 2: 108 (36.5%) Item 7: 135 (45.5%) Item 9: 94 (31.8%)	MIES: Overall M/SD (From supplement) 22.54(10.48) Subscale M/SD Transgressions-others: 4.08(2.71) Transgressions-self: 1.49(0.81) Betrayal: 2.81(1.33) MISS-HP overall: 33.90(12.72)	5	NR

												MISS-HP overall %<br>120 (40.7%)			
Borges et al., 2021	Longitudinal	211	Healthcare<br>Physician (12.32%)<br>Nurse (10.43%)<br>Mental health provider (61.14%)<br>Occupational therapist (1.90%)<br>Nurse practitioner (2.37%)<br>Physician assistant (0.47%)	Convenience<br>May 2020 – March 2021	NR	16.26 %	89.16%	USA	NR	MIES 2+ (moderately agree)<br>Lower score indicates higher moral injury<br>2 subscales (perpetration and betrayal)	Demographics<br>Sex (0)<br>Race (0)<br>Profession (0)<br>No other variables reported at univariate level	Overall %<br>109 (51.66%)<br>Per subscale %<br>Perpetration: 77 (36.49%)<br>Betrayal: 88 (41.71%)	Overall M/SD<br>NR<br>Subscale M/SD<br>NR	5	None
Boscarino et al., 2022<br><br>Narrative synthesis only	Cross-sectional	1032	Military veterans.	Random sample of veterans on a registry care at a private hospital.<br><br>February 2016 – February 2017	NR	95.5 %	NR	USA	55 %	MIES 75th percentile<br>Unidimensional	(Mental) health<br>Cage positive alcohol score (+)<br>Used alc/drugs to cope (+)<br>Pain interferes (+)<br>Sleep problems (+)<br>Concussion history (+)	Overall %<br>764 (74%)<br>Subscale %<br>NR	Overall M/SD<br>NR<br>Subscale M/SD<br>NR	3	None

											Mental health treatment in past year (+)<br>Opioid dependence past year (+)<br>Individual/work characteristics<br>Guard/reserve (-)<br>Combat high (+)<br>Drafted (+)<br>Vietnam war deployment (+)<br>Low unit support/morale (+)<br>High stress past year (+)<br>High lifetime trauma (+)<br>Low homecoming support (+)<br>High child abuse/neglect (+)<br>High fear of death (+)<br>High neuroticism (+)<br>Low self-esteem (+)<br>Repression to cope (+)<br>High anomie (+)<br>Anti-social personality (+)<br>Used marijuana 50+ times (+)<br>Demographics<br>Age (+)				
Brady et al., 2021	Cross-sectional<br>.	390	Nursing home staff. Nurses (30.8%)	Convenience November 2020 – January 2021.	NR	12.8 %	83.3%	Republic of Ireland.	10.2 %	MIES 4+ (slightly agree)	Demographics<br>HCA compared to non-clinical staff (+)	Overall %<br>237 (60.8%)<br>Per subscale %	Overall M/SD<br>2.35(1.09)<br>Subscale M/SD	4	Honoraria for board meeting.

			Healthcare assistants (44.1%)<br>Non-clinical (25.1%)							Cut off: 4+ (Slightly agree)<br>3 subscales (transgressions-others, transgressions-self, betrayal)		Transgressions-others: 189 (48.5%)<br>Transgressions-self: 92 (23.6%)<br><br>Betrayal: 166 (42.6%)	Transgressions-others: 2.93(1.51)<br>Transgressions-self: 1.97(1.19)<br>Betrayal: 2.46(1.34)		
Brady et al., 2022	Cross-sectional	377	Hospital Doctors (48%)<br>Nurses (44%)<br>Radiographers (8%)	Convenience<br>January – March 2021	NR	22.3 %	90.5%	Republic of Ireland. Dublin.	6%	MIES 4+ (slightly agree)<br>Cut off: 4+ (Slightly agree)<br>3 subscales (transgressions-others, transgressions-self, betrayal)	Demographics<br>Professional role - doctors (-)	Overall %<br>268 (71.1%)<br>Per subscale %<br>Transgressions-others: 197 (52.3%)<br>Transgressions-self: 92 (24.4%)<br>Betrayal: 210 (55.7%)	Overall M/SD<br>2.48(1.10)<br>Subscale M/SD<br>Transgressions-others: 3.01(1.56)<br>Transgressions-self: 1.97(1.12)<br>Betrayal: 2.81(1.44)	6	
Chandrabhatla et al., 2022	Cross-sectional		Hospitalist	Convenience<br>September 2019 – August 2020	NR	48%	NR	USA	52 %	MIES Unidimensional	(Mental health):<br>Burnout (0)<br>Burnout during COVID subsample (+)	Overall %<br>60 (71.4%)*<br>Subscale %<br>Transgressions-others: 49(58.3%)**<br>Transgressions-self: 23(27.4)*<br>Betrayal: 37(44%)*<br>*N=78<br>**N=77	Overall M/SD<br>2.44(1.07)*<br>Per subscale M/SD<br>Transgressions-others: 3.41(1.64)**<br>Transgressions-self: 1.88(1.15)***<br>Betrayal: 2.54(1.31)***<br>*N=73<br>**N=74<br>***N=76	5	None

Dale et al., 2021	Longitudinal	265	Healthcare	Convenience October 2020 – March 2021	37.62 (11.1)	18.1 %	77.7%	USA	NR	MIES 3 subscales (transgressions-others, transgressions-self, betrayal)	NR at univariate level	Overall % NR (7 items only) Subscale % Transgressions -others: 6 (2.3%) Transgressions -self only: 61 (23%) Perpetration: 21 (7.9%) Perpetration (high): 88 (33.2%)	Overall M/SD NR Per subscale M/SD Transgressions -others: 2.54(1.45) Transgressions -self: 1.66(1.05)	5	None
Plouffe et al., 2021<br><br>Two samples	Longitudinal (T2)	<u>1:</u> 803<br><br><u>2:</u> 1510	Military. <u>Sample 1:</u> active duty <u>Sample 2 -</u> released.	Nationally representative sample. January – May 2018 (T2)	NR	<u>Sample 1:</u> 87.53 % <u>Sample 2:</u> 88.02 %	NR	Canada	68 %	MIES Unidimensional	NR	Overall % NR for both samples Subscale % NR for both samples<br><br>Per item % <u>Sample 1:</u> Item 1: 64.6% Item 2: 37.3% Item 3: 16.9% Item 4: 13.2% Item 5: 19.9% Item 6: 14.9% Item 7: 48.5% Item 8: 34.5% Item 9: 27.5% <u>Sample 2:</u> Item 1: 66.1% Item 2: 42.2% Item 3: 20.4% Item 4: 17.3%	Overall M/SD Sample 1: 2.46(4.53) Sample 2: 2.63(4.66)<br><br>Subscale M/SD NR for both samples	2	None

												Item 5: 24.4%<br>Item 6: 21.3%<br>Item 7: 53%<br>Item 8: 37.8%<br>Item 9: 34.6%			
Nichter et al., 2021	Cross-sectional	1321	Veterans. All reported exposure to combat/war zone. Army (47.6%) Navy (14.6%) Air Force (6.3%) Marines (6.3%) National Guard/Reserves/Coast Guard (9.3%)	National random samples and apply post-stratification wrights to generalise to entire US veteran population. 2019-2020	59.1 (16.6)	93.7 %	75.1%	USA	NR	MIES 5+ (moderate) 3 subscales (transgressions-others, transgressions-self, betrayal)	NR at univariate level.	*Reports weighted % and SE<br><br>Overall % 535, (36.8%, 1.3%)<br><br>Subscale % Transgressions -Others 316, (23.1%, 1.2%)<br><br>Transgressions -Self 152 (11.2%, 0.9%)<br><br>Betrayal 189 (24.5%, 1.3%)<br><br>Per item<br>Item 1: 279 (21%, 1.1%)<br>Item 2: 189, (13.6%, 1.0%)<br>Item 3: 90 (6.5%, 0.7%)<br>Item 4: 88 (6.0%, 0.8%)	Overall M/SD 2.0(1.1)<br>Subscales M/SD<br><br>Transgressions -others 2.5(1.6)<br><br>Transgressions -self 1.7(1.1)<br><br>Betrayal 2.1(1.3)	1	NR

												Item 5: 77<br>(6.0%, 0.7%)<br>Item 6: 71<br>(5.9%, 0.7%)<br>Item 7: 289<br>(17.9%, 1.1%)<br>Item 8: 214<br>(14.9%, 1.0%)<br><br>Item 9: 79<br>(6.5%, 0.6%)			
Fitzpatrick et al., 2022	Cross-sectional.	676	Nurses.<br>Staff nurses (65%)<br>Nurse leaders (35%)	Convenience<br>June - August 2021.	43.88 (1.99)	8.3%	89.6%	USA	NR	MISS-HP	(Mental) health<br>Lower well-being (+)<br>Individual and/or work characteristics<br>Resilience (-)<br>Demographics<br>Staff nurses (+)	Overall %<br>NR	Overall M/SD<br>42.21(15.73)	5	NR
Hagerty & Williams, 2022<br><br>Narrative synthesis only	Cross-sectional	1122	Healthcare<br>Nurse (60.4%)<br>Physician (5%)<br>Physician assistant (1.7%)<br>Respiratory therapist (3.6%)<br>Other health-care roles (28%)<br>Provided care in ICU during the	Convenience<br>May 2020 – August 2020.	39.29	11.2 %	NR	USA	NR	MIES 4+ (slightly agree)<br>3 subscales (transgressions-others, transgressions-self, betrayal)	(Mental) health<br>Loneliness (DJGLS) (+)<br>Demographics<br>Age (-)<br>Access to PPE (-)<br>Number of COVID-19 patient deaths that respondents personally treated (+)	Overall %<br>NR<br><br>Subscale %<br>NR<br><br>Per item %<br><br>Item 2: 525 (47.2%)<br>Item 7: 747 (67.2%)<br>Item 4: 234 (21%)	Overall M/SD<br>NR<br>Subscale M/SD<br>NR	5	None

			pandemic (47.6%)												
Hamrick et al., 2022	Cross-sectional	154	Military (serving and veterans). Former/retired (77.9%). Army (20.8%) Navy (52.6%) Airforce (16.2%) Marines (4.5%) Coast Guard (0.6%) National Guard (7.8%) (Above) reserves (20.8%) Other (0.6%)	Convenience February – April 2019	36.6 (11.8)	0%	72.1%	USA	NR	EMIS-M 1 subscale (other-directed)	(Mental) health Depression (+) Anxiety (+) Suicidal ideation (0) Substance use (0) Individual and/or work characteristics Sexual harassment (+)<br><br>*All correlations with the other-directed MI subscale.	Overall % NR<br><br>Subscale % NR	Overall M/SD NR Subscale M/SD Other-directed MI 2.66(1.17)	5	None

Hines et al., 2020	Cross-sectional	219	Health and social care<br>Attending physician (47%)<br>Fellow physician (18%)<br>Resident physician (20%)<br>Other (15%)	Convenience<br>March – April 2020	39.10 (11.1)	43%	NR	USA	26 %	MIES<br>2 subscales (perpetration, and betrayal).	NR at univariate level.	Overall %<br>NR<br><br>Subscale %<br>NR	Overall M/SD<br>1.79 (0.87)<br>Subscale M/SD<br>Perpetration 1.65 (0.87)<br>Betrayal 2.10 (1.28)	5	NR
Hinkel et al., 2022	Cross-sectional	59	Veterans.	Convenience<br>November 2018 – November 2019	NR	86.4 %	59.3%	USA	NR	EMIS-M<br>2 subscales (self-directed MI and other-directed MI)	(Mental) health<br>PTSD (PCL-5) (+)<br>MDD (PHQ-8) (+)<br>Chronic pain (BPI-SF) (+)	Overall %<br>NR<br><br>Subscale %<br>NR	Overall M/SD<br>NR<br>Subscale M/SD<br>Self-directed MI 2.29(1.09)<br>Other-directed MI 2.54(1.32)	5	NR
Kelley et al., 2021	Cross-sectional	269	Veterans.<br>Navy (45.4%)<br>Marine Corps (21.9%)<br>Army (19.7%)	Convenience	36.5 (11)	49.8 %	74.3%	USA	NR	EMIS-M<br>2 subscales (self-directed MI and other-directed MI)	(Mental) health<br>Suicidal ideation (+)<br>PTSD diagnosis (+)<br>Individual/work characteristics<br>Presence of meaning in life (MLQ) (-)<br>Searching for meaning in life (MLQ) (0)<br>Demographics<br>Veteran status (+)<br>Age (0)<br>*Both EMIS subscales	Overall %<br>NR<br><br>Subscale %<br>NR	Overall M/SD<br>NR<br>Subscale M/SD<br>Self-directed MI 1.83(0.86)<br>Other-directed MI 2.48(1.11)	5	None

La Fleur et al., 2020	Cross-sectional	212	Veterans. Marine Corps (13.7%) Army (26.4%) Navy (13.7%) Air Force (5.7%) Reserves (2.8%) National Guard (4.2%) Multiple (33.5%)	Convenience	NR	80.7 %	74.1%	USA	NR	MIQ-M 2.80+ 19 items Unidimensional.	(Mental) health Life satisfaction (0) Individual/work characteristics Disclosure support (0) Disclosure concerns (+)	Overall % 27% Overall % NR	Overall M/SD 2.04(0.79) Subscale M/SD NR	6	None
Lamb et al., 2021	Cross-sectional (cohort).	2166	Healthcare Doctor (11.7%) Nurse (29.9%) Other clinical (26.8%) Non-clinical (32.3%)	Convenience April-June 2020.	NR	59.4 %	24.6%	UK	12 %	MIES 5+ (moderately agree) Subscales: 3 subscales (commission, omission and betrayal)	Demographics Age (-) Sex other (+) Relationship status divorced/separated/widowed/single (+) Ethnicity Asian (+) Country of birth (0) Length of time living in UK (-) Main role nurse (+)	*Reports weighted % Overall % 602 (25.09%) Subscale % Commission: 346 (14.28%) Omission: 111 (4.57%) Betrayal: 439 (18.11%)	Overall M/SD 1.72 (1.19) Subscale M/SD Commission: 1.71 (1.06) Omission: 1.47 (1.1) Betrayal: 1.8 (1.14)	2	Grant.
Litam et al., 2021	Cross-sectional	109	Healthcare Physicians (37%) Nurses (57%) Other professionals (6.4%)	Convenience May-July 2020	37.50 (12.4)	24%	75.2%	USA	NR	MIES Lower score indicates higher moral injury Unidimensional	(Mental) health Compassion satisfaction (PROQOL) (+) Burnout (PROQOL) (+) Secondary traumatic stress (PROQOL) (+)	Overall % NR Subscale % NR	Overall M/SD 3.03(0.72) Subscale M/SD NR	5	None.

Litz et al., 2022<br><br>Four studies	Cross-sectional	1: 533<br>2: 363<br>3: 264<br>4: 118	Samples 1, 2 and 3: Veterans<br>Sample 4: Defence members and veterans.	Sample 1: Convenience<br>Samples 2, 3 and 4: NR	Sample 1: 51.87 (9.77)<br>Samples 2, 3 and 4: NR	Sample 1: 71%<br>Sample 2: 73.6 %<br>Sample 3: 67%<br>Sample 4: 65.9 %	NR for all	Sample 1: Canada<br>Sample 2: USA<br>Sample 3: UK<br>Sample 4: Australia	NR for all	MIOS<br>2 subscales (shame-related outcomes and trust violation-related outcomes)	NR at univariate level.	Sample 1:<br>Overall %<br>366 (68.7%)<br>Subscale %<br>MI-Self: 265 (49.7%)<br>MI-Other: 378 (71%)<br>Impact from an MI-Other: 440 (82.5%)<br>Sample 2:<br>Overall %<br>283 (78%)<br>Subscale %<br>MI-Self: 142 (39%)<br>MI-Other: 142 (39%)<br>Impact from an MI-Other: 142 (39%)<br>Sample 3:<br>Overall %<br>264 (100%)<br>Subscale %<br>MI-Self: 174 (65.9%)<br>MI-Other: 170 (64.4%)<br>Impact from an MI-Other: 186 (70.1%)<br>Sample 4:<br>Overall %<br>NR<br>Subscale %<br>MI-Self: 65 (55%)	Sample 1:<br>Overall M/SD<br>25.31(11.38)<br>Subscale M/SD<br>Shame-related outcomes: 11.28(6.64)<br>Trust violation outcomes: 14.03(5.85)<br>Sample 2:<br>Overall M/SD<br>25.14(11.36)<br>Subscale M/SD<br>Shame-related outcomes: 11.36(6.82)<br>Trust violation outcomes: 13.78(5.58)<br>Sample 3:<br>Overall M/SD<br>32.87(10.54)<br>Subscale M/SD<br>Shame-related outcomes: 16.29(6.20)<br>Trust violation outcomes: 16.58(5.35)<br>Sample 4:<br>Overall M/SD<br>27.74(10.42)<br>Subscale M/SD	5<br><br>4: 6	None

												MI-Other: 93 (79%)<br><br>Impact from an MI-Other: 103 (87%	Shame-related outcomes: 12.17(6.35)<br>Trust violation outcomes: 15.56(5.52)		
Maftei & Holman (2021)	Cross-sectional	114	Physicians	Convenience April 2020	38.85 (9.82)	24.6 %	NR	Romania	1.14 %	MIES 4+ (agree)<br>1-5 Likert scale<br>3 subscales (transgressions-others, transgressions-self, betrayal)	(Mental health)<br>Emotional impact (0) *<br>Emotional impact (+) **<br>Physical impact (+)<br>Demographics<br>Age (0)<br>Working experience (0)<br>*non-covid-19 medical unit<br>** covid-19 medical unit	Overall %<br>80 (70.2%)<br><br>Per subscale %<br>Transgressions -others: 61(53.5%)<br>Transgressions -self: 31(27.2%)<br>Betrayal 66(57.9%)<br>Per item %<br>Item 1: 45 (39.5%)<br>Item 2: 39 (34.2%)<br>Item 3: 14 (12.3%)<br>Item 4: 15 (13.2%)<br>Item 5: 20 (17.5%)<br>Item 6: 22 (19.3%)<br>Item 7: 35 (30.7%)<br>Item 8: 37 (32.5%)	Overall<br>2.68(0.90)<br><br>Subscale M/SD<br>Transgressions -others: 3.06(1.26)<br>Transgressions -self: 2.20(1.05)<br>Betrayal 3.06(1.08)	5	None

												Item 9: 33 (33%)			
Maguen et al., 2022	Cross-sectional	14057	Post 9/11 veterans.<br>Army (50.5%)<br>Navy/Coast Guard (17.9%)<br>Marines (13.3%)<br>Air Force (18.3%)	Nationally representative, population-based sampling frame<br>April – August 2018	NR	82.3%	66.2%	USA	40%	MIES 4+ (slightly agree)<br>3 subscales (transgressions-others, transgressions-self, betrayal)	Demographics<br>Female gender (+) *<br>Female gender (-) **<br><br>*transgressions-others and betrayal<br>** transgressions-self	Overall %<br>7830 (55.7%)<br>Per subscale %<br>Transgressions-others 6059 (43.1%)<br>Transgressions-self 3064 (21.8%)<br>Betrayal 5651 (40.2%)	Overall M/SD<br>NR<br>Subscale M/SD<br>NR	2	None
Malakoutikhah et al., 2022	Cross-sectional	455	Healthcare<br>Operating room (6.3%)<br>Nursing (73.7%)<br>Anaesthesiology (8.2%)<br>Medicine (7.5%)<br>Midwifery (2.7%)<br>Nursing assistant (1.6%)	Convenience<br>September 2021 – February 2022	NR	16.9%	NR	Iran	NR	MISS-HP 36.5+<br>Item content varied	(Mental) health<br>General health (GHQ) (+)<br>PTSD (IES-R) (+)	Overall %<br>190 (42.7%)<br>Per item %<br>Scoring strongly agree:<br>Item 2: 13 (5.1%)<br>Item 3: 5 (2%)<br>Item 4: 15 (6%)<br>Item 5: 71(28%)<br>Item 6: 37 (14.6%)<br>Item 7: 71(28%)<br>Item 8: 39 (15.5%)<br>Item 9: 4(1.6%)<br>Item 10: 1 (0.4%)	Overall M/SD<br>35.66(12.08)	5	None.

												Item 11: 3 (1.2%)			
Mantri et al., 2020	Cross-sectional	181	Healthcare<br>Physicians (70.7%)<br>Nurse (9.4%)<br>Other (19.9%)	Convenience<br>November 2019 – March 2020	NR	63.5 %	74.5%	USA	NR	MISS-HP 36+<br><br>EMIS-M<br>2 subscales (self-directed MI and other-directed MI)	(Mental) health<br>Burnout (MBI) (+)<br>Anxiety (GAD-7) (+)<br>Depression (PHQ-9) (+)<br>Individual/work characteristics<br>Religiosity (-)<br>*MISS-HP correlations	MISS-HP<br><br>Overall %<br>86 (47.5%)	MISS-HP<br>Overall M/SD<br>36.8(13.3)<br>EMIS-HP<br>Overall M/SD<br>2.01(0.78)<br>Subscales M/SD<br>NR	5	None
Mantri et al., 2021<br><br>Two samples	Cross-sectional	Sample 1:<br>450<br><br>Sample 2:<br>1381	Healthcare<br>Sample 1:<br>Nurse (31.8%)<br>Physician (25.8%)<br>APP (18.9%)<br>Chaplaincy (14.2%)<br>Social worker (2.9%)<br>Other (6.4%)<br>Sample 2:<br>Nurse (56.5%)<br>Physician (12%)	Convenience.<br><br>Sample 1:<br><br>April-May 2020<br><br>Sample 2:<br><br>October-November 2020	NR	Sample 1:<br><br>12.4 %<br><br>Sample 2:<br><br>9.5%	Sample 1:<br><br>88.7%<br><br>Sample 2:<br><br>93.3%	Global<br><br>Primarily North America (USA)	Sample 1:<br><br>72.8 %<br><br>Sample 2:<br>73.7 %	MISS-HP 36+	(Mental) health<br>Burnout emotional exhaustion (aMBI) (-)<br>Burnout depersonalisation (aMBI) (-)<br>Burnout reduced personal/professional accomplishment (aMBI) (+)<br>Individual/work characteristics<br>COVID experience (0) *<br>COVID experience (+) **<br>Religiosity (DUREL) (-)<br>Demographics<br>Age (-)	Overall %<br>Sample 1:<br>84 (18.6%)<br>Sample 2:<br>591 (42.8%)	Overall M/SD<br>Sample 1:<br>27.4(11.6)<br>Sample 2:<br>36.4(13.8)	5	None

			APP (19.8%)<br>Housekeeping (0.1%)<br>Food services (0.1%)<br>Interpreter (0.1%)<br>Chaplaincy (0.1%)<br>Social worker (1.3%)<br>Other (19.9%)								Sex (0)<br>Race (0)<br>Marital status (0) *<br>Marital status (+) **<br>Profession (-)<br>*sample 1<br>** sample 2				
Morris, Webb & Devlin, 2022	Cross-sectional	237	Healthcare<br>Dietetics (2.5%)<br>Education (2.5%)<br>Medical Doctor (0.8%)<br>Nursing (Registered) (26.3%)<br>Nursing (Unregistered) (46.6%)<br>Occupational Therapy (5.1%)<br>Psychology (11.4%)<br>Psychiatry (1.3%)	Convenience	NR	34.2 %	78.9%	UK	NR	MIES 6+ (strongly agree)<br>3 subscales (transgressions-others, transgressions-self, betrayal)	(Mental) health<br>Burnout (ProQoL) (+)<br>Secondary traumatic stress (ProQoL) (+)<br>Compassion satisfaction (ProQoL) (-)<br>Individual/work characteristics<br>Impact of COVID-19 (0)<br>Demographics<br>Gender (0)<br>Ethnicity (0)<br>Gendered ethnicity (0)	Overall %<br>183 (78.5%) *<br>Subscale %<br>Transgressions-others: 154 (67%) **<br>Transgressions-self: 79 (34.3%) **<br>Betrayal: 154 (66.1%) *<br>*n = 233<br>**n = 230	Overall M/SD<br>2.90 (1.41)<br>Subscale M/SD<br>Transgressions-others: 3.79(1.86)<br>Transgressions-self: 2.13(1.47)<br>Betrayal 3.38(1.83)	5	None

			Recreational Co-ordinator (1.7%)<br>Social Work (0.8%)<br>Other (0.4%)												
Rushton et al., 2022	Cross-sectional	595	Healthcare<br>Nurse (58.1%)<br>Physician (11.8%)<br>Respiratory therapist (6.3%)<br>Nurse practitioner/physician assistant (7.1%)<br>Other (16.7%)	Convenience<br>June 2020 – November 2020	NR	NR	82.4%	USA	NR	MISS-HP 36+	Individual/work characteristics<br>Ethical concerns (ECI) (+)<br>Involvement in COVID-19 clinical care (+)<br>Practice location (0)<br>Speciality (0)<br>Demographics<br>Years in profession (-)<br>Profession (0)<br>Education (0)<br>Religious/spiritual preference (+)	Overall %<br>32.4%	Overall M/SD<br>30.57(12.11)	5	None
Nieuwsma et al., 2020	Cross-sectional	315	Post 9/11 veterans with warzone experience.<br>Army (67%)<br>Navy (13.2%)<br>Air Force (9.9%)<br>Marines (9.9%)	Convenience	46.36 (10.4)	86.6 %	55.6%	USA	33.5 %	MIES<br>2 subscales (perpetration and betrayal)<br>MIQ-M<br>20 items<br>Unidimensional.<br>BMIS 2+ (agree)<br>2 subscales (event and sequela).	(Mental) health<br>Depression (BDI-2) (+)<br>Suicidal ideation (BSI) (+)<br>Alcohol use disorder (AUDIT) (+)<br>Drug abuse (DAST) (+)<br>PTSD (DTS) (+)<br>*all correlations positive for BMIS, MIES and MIQ-M	Overall %<br>NR<br>Subscale %<br><br>BMIS-E<br>137 (44.1%)<br>NR for BMIS-S subscale<br>*n = 311	Overall M/SD<br>BMIS<br>3.71(4.38)<br>MIES<br>2.57(1.26)<br>MIQ-M<br>1.49(0.47)<br>Subscale M/SD<br><br>NR for all scales<br>*n = 311	5	NR

Osmann et al., 2022	Cross-sectional	159	Journalists	Convenience	44.72 (12.1)	52%	NR	NR	72 %	Toronto Moral Injury Scale for Journalists<br>3 subscales (organisational, Individuals, and online)	(Mental) health<br>PTSD (PLC-5) (+)<br>Anxiety (BAI) (+)<br>Depression (BDI-II) (+)<br>Demographics<br>Marital status (0)<br>Education (0)	Overall %<br>NR<br>Subscale %<br>NR	Overall M/SD<br>1.50(1.27)<br>Subscale M/SD<br>Organisational/management: 1.54(1.32)<br>Individuals/Nonmanagement: 1.18(1.19)<br>Online: 1.68(1.27)	5	None
Rodríguez et al., 2021	Cross-sectional	169	Healthcare<br>Physicians (86.39%)<br>Nurses (13.61%)	Convenience	36.04 (10.5)	23.7 %	NR	Honduras	NR	MISS-HP 36+	(Mental) health<br>Depression (PHQ-9 (+)<br>Anxiety (GAD-7) (+)	Overall %<br>77 (45.6%)	Overall M/SD<br>34.80(15.81)	5	None
Roth et al., 2022	Cross-sectional	270	Personal Safety Personnel (PSP).<br>Firefighter (19%)<br>Paramedic (44%)<br>Police (17%)<br>Dispatcher (6%)<br>Other (14%)	Convenience	35 (9.5)	63%	85%	Canada and USA	NR	MIA-PSP<br>3 subscales (perpetration, betrayal and emotional sequalae)<br><br>MIES<br>Unidimensional.	(Mental) health<br>Shame (TRSI) (+)<br>Guilt (TRGI) (+)<br>PTSD (PCL-5) (+)<br>Depression, anxiety and stress (DASS-21) (+)<br>Dissociation (MDI) (+)<br>Disability, functioning and health (WHODAS) (+)	Overall %<br>NR for either scale	Overall M/SD<br>MIA-PSP<br>59.6(21.9)<br>MIES<br>3.56(1.39)<br>Subscale M/SD<br>MIA-PSP<br>Emotional Sequalae: 25(9.7)<br>Perpetration: 15(8.5)<br>Betrayal: 19.6(7.7)	5	None

													Subscale M/SD<br>MIES<br>NR		
Russell & Mussap, 2022<br><br>Two studies	Cross-sectional	Study 1: 183<br>Study 2: 393	Study 1:<br><br>Current and former military personnel.<br><br>Currently serving (50.3%)<br><br>Study 2:<br><br>Current and former military personnel.<br><br>Currently serving (39.7%)	Study 1 and 2: Convenience.<br><br>.	Study 1: 43.3 (11.9)<br>Study 2: 44 (12)	Study 1: 83.6 %<br>Study 2: 81.4 %	Study 1: 84.7%<br>Study 2: 81.7%	Study 1: Global but mainly Australian (89%)<br>Study 2: Global but primarily Australian (85%)	Study 1: NR<br>Study 2: NR	Study 1:<br>MIES 4+ (slightly agree)<br>3 subscales (transgressions-others, transgressions-self, betrayal)<br>Study 2:<br>MIES 4+ (slightly agree)<br>3 subscales (transgressions-others, transgressions-self, betrayal)	Study 1:<br>(Mental) health<br>PTSD (PCL-M) (+)<br>Post-traumatic growth (PTGI-X) (0)<br>Personal well-being (PWI) (-)<br>Individual/work characteristics:<br>Intrusive rumination (ERRI) (+)<br>Deliberate rumination (ERRI) (+)<br>Exposure to danger/combat (EDC) (0) *<br>Exposure to danger/combat (EDC) (+) **<br>Witnessing the consequences of war (WDW) (+)<br>Demographics<br>Age (0)<br>Study 2:<br>(Mental) health<br>PTSD (PCL-M) (+)<br>Post-traumatic growth (PTGI-X) (0)	Study 1:<br>Overall %<br>132 (72.1%)<br>Subscale %<br>Transgressions-self: 97 (53%)<br>Transgressions-others: 48 (26.2%)<br>Betrayal: 99 (53.8%)<br>Study 2:<br>Overall %<br>300 (76.6%)<br>Subscale %<br>Transgressions-self: 109 (59.4%)<br>Transgressions-others: 62 (33.8%)<br>Betrayal: 112 (61.4%)	Study 1:<br>Overall M/SD: 2.25(1.12)<br>Subscale M/SD:<br>Transgressions-self: 2.88(1.63)<br>Transgressions-others: 1.73(1.20)<br>Betrayal: 2.54(1.45)<br>Study 2:<br>Overall M/SD: 2.60(1.28)<br>Subscale M/SD:<br>Transgressions-self: 3.23(1.74)<br>Transgressions-others: 2.05(1.42)<br>Betrayal: 2.9(1.58)	5	None

											Personal well-being (PWI) (-)<br>Individual/work characteristics:<br>Threat bias (IBIP) (+)<br>Exposure to danger/combat (EDC) (+) ***<br>Exposure to danger/combat (EDC) (0) ****<br>Witnessing the consequences of war (WDW) (+)<br>Demographics<br>Age (0)<br>*Transgressions-self<br>**Transgressions-other and betrayal<br>***Transgressions-self and transgressions-others<br>****Betrayal				
Saba et al., 2022	Longitudinal.<br><br>12-month time point.	1005	Veterans.<br>Air Force (14.53%)<br>Army (65.27%)<br>Marine Corps (9.65%)<br>Navy (10.55%	Convenience<br>February 2021.	34.83 (3.61)	91.14 %	84.58%	NR	21.3 %	MIES 4+ (slightly agree)<br>3 subscales (transgressions-others, transgressions-self, betrayal)	(Mental) health<br>PTSD (PCL-5) (+)<br>Depression (PHQ-9) (+)<br>Anxiety (GAD-7) (+)<br>Anger (DAR-R) (+)	Overall %<br>881 (87.66%)<br>Subscale %<br>Transgressions-self: 802 (79.80%)<br>Transgressions-others: 303 (30.15%)<br>Betrayal: 300 (29.85%)	Overall M/SD: 2.75(0.81)<br>Subscale M/SD:<br>Transgressions-self: 2.65(1.20)<br>Transgressions-others: 2.92(0.84)<br>Betrayal: 2.58(1.11)	5	None

Senger, Torres & Ratcliff, 2022	Cross-sectional	242	First responders. <u>In person sample: (n=91)</u> Firefighter (37.4%) Law enforcement (20.90%) Paramedic (2.20%) EMT (4.40) Multiple (35.20%) <u>Online sample (n=151):</u> Firefighter (6%) Law enforcement (38.40%) Paramedic (16.60%) EMT (5.30%) Search and rescue worker (3.30%) Other (1.30%) Multiple (29.10%)	Convenience January – December 2020	NR	<u>In person sample;</u> 97.8 % <u>Online sample:</u> 75.50 %	<u>Online sample:</u> 83.30% <u>In person sample:</u> 88.70%	USA	NR	MIES 4+ (slightly agree) 3 subscales (transgressions-others, transgressions-self, betrayal)	Individual/work characteristics: Pre vs post COVID (-) Military service (-) Demographics Occupation type (+) Time in occupation (+) Age (+) Gender (0) Race/ethnicity (0) Education (0) Marital status (+) Income (0) Religion (0)	Overall % 98 (40.65%) Subscale % Transgressions -self: 51 (21.23%) Transgressions -others: 147 (60.75%) Betrayal: 97 (39.97%) Per item % Item 1: 174 (72.2%) Item 2: 119 (49.3%) Item 3: 48 (19.7%) Item 4: 56 (23.3%) Item 5: 53 (21.8%) Item 6: 49 (20.1%) Item 7: 102 (42%) Item 8: 94 (38.7%) Item 9: 95 (39.2%)	Overall M/SD: 2.7 (1.1) Subscale M/SD: Transgressions -self: 2.1 (1.3) Transgressions -others: 3.8 (1.4) Betrayal: 2.9 (1.5)	6

Brennan CJ, *et al. BMJ Open* 2024; 14:e071776. doi: 10.1136/bmjopen-2023-071776

Stanojević & Čartolovni, 2022	Cross-sectional	162	Nurses. Palliative nurses (47.5%) Oncology nurses (52.5%)	Convenience January - June 2021.	NR	13%	NR	Croatia	64.8 %	MISS-HP 36+	(Mental) health:<br>Moral distress (+)<br>Individual/work characteristics:<br>Left or considered leaving clinical position (+)<br>Considering about leaving clinical position (+)<br>Demographics<br>Age (0)<br>Work experience (0)<br>Education (0)<br>Gender (0)<br>Specialisation (0)	Overall % NR	Overall M/SD 37.63(12.19)	5	None
Testoni et al., 2022	Cross-sectional	270	Healthcare professionals. Physician (65% Other healthcare provider (35%)	Convenience July - December 2020	44.5 (12.2)	30%	NR	Italy	NR	MIES 4+ (slightly agree) 3 subscales (transgressions-others, transgressions-self, betrayal)	(Mental) health:<br>Alienation (+)<br>Individual/work characteristics:<br>Self-efficacy (NYPQS) (-)<br>Humanisation patient (HTAS) (0)<br>Humanisation colleague (HTAS) (-)<br>Overall humanisation (HTAS) (-)<br>Dehumanisation patient (HTAS) (+)<br>Dehumanisation colleague (HTAS) (+)<br>Overall dehumanisation (HTAS) (+)	Overall % 212 (78.5%)<br>Subscale %<br>Transgressions-self: 78 (28.9%)<br>Transgressions-others: 166 (61.5%)<br>Betrayal 166 (61.55)	Overall M/SD 2.62(1.18)<br>Subscale M/SD<br>Transgressions-self: 2.06(1.45)<br>Transgressions-others: 3.23(1.56)<br>Betrayal 2.96(1.48)	5	None

Thomas, Weiss, Forkus & Contractor, 2022	Cross-sectional	496	Veterans. Army (63.7%) Navy (9.9%) Air Force (19%) Marines (6.3%) Coast Guard (1.2%)	Convenience	37.80 (11.4)	70.5 %	71.1%	USA	18.8 %	MIES 3 subscales (transgressions-others, transgressions-self, betrayal)	(Mental) health: Alcohol misuse (AUDIT) (+) Individual/work characteristics: Total religiosity (DUREL) (+) Organisational religiosity (DUREL) (+) Nonorganizational religiosity (DUREL) (0) Intrinsic religiosity (DUREL) (0)	Overall % NR Subscale % NR	Overall M/SD 3.19(1.40) Subscale M/SD NR	5	NR
Trifunovic-Koenig et al., 2022	Cross-sectional	156	Healthcare Physician (16%) Nurse (general) (33.3%) Paediatric nurse (1.3%) Paramedic (23.7%) Geriatric nurse (9%) Speech therapist (clinical linguistic) (7.1%) Medical assistant (3.2%) Medical student (1.3%)	Convenience March - June 2021	40.7 (10.2)	34.6 %	NR	German speaking	3.5 %	MISS-HP 28.5+ 9 item scale	(Mental) health: Psychological distress (+) Physical distress (+) Individual/work characteristics: Colleague support (+) Supervisor support (+) Institutional support (+) Professional self-efficacy (+) Turnover intentions (+) Absenteeism (+) Resilience (+)	Overall % 85 (54.5%)	Overall M/SD 32.31(13.26)	5	None

			Physiotherapist (0.6%)<br>Midwife (0.6%)<br>Respiratory therapist (1.3%)<br>Administration worker/Technician (0.6%)												
Ulusoy & Çelik 2022	Cross-sectional	124	Healthcare workers.<br>Doctors (67.7%)<br>Nurses (21.8%)<br>Medical secretaries (4.8%)<br>Other support staff (5.6%)	Convenience<br>January - April 2021	NR	NR	NR	Turkey	36 %	MIES 4+ (slightly agree)<br>Unidimensional	(Mental) health<br>Burnout emotional exhaustion (MBI) (+)<br>Burnout depersonalisation (MBI) (+)<br>Burnout reduced personal accomplishment (MBI) (0)<br>Obstruction to valued living (VQ) (+)<br>Depression, anxiety, and stress (DASS-21) (+)<br>Psychological inflexibility and experiential avoidance (AAQ-II) (+)	Overall %<br>116 (93.5%)<br><br>Subscale %<br>Transgressions-self: 78 (62.9%)<br>Transgressions-others: 109 (87.9%)<br>Betrayal: 96 (77.4%)	Overall M/SD<br>3.42(0.99)<br><br>Subscale M/SD<br>Transgressions-self: 2.82(1.31)<br>Transgressions-others: 4.51(1.34)<br>Betrayal: 3.51(1.28)	5	None

Williamson Murphy & Greenberg, 2022	Cross-sectional	90	Veterinary	Convenience December 2020 – May 2021	36.8 (10.2)	12.2 %	70%	UK	27 %	MIES 5+ (moderately agree)<br>3 subscales (transgressions-others, transgressions-self, betrayal)	(Mental) health<br>PTSD (ITQ) (+)<br>Alcohol (AUDIT-C) (0)<br>Common mental disorders (PHQ-4)) (0)<br><br>Demographics<br>Age (0)<br>Years of experience (0)<br>Role type (0)	Overall %<br>80 (88.9%)<br><br>Subscale %<br><br>Transgressions-self: 51 (56.7%)<br>Transgressions-others: 63 (70%)<br>Betrayal: 62 (68.9%)	Overall M/SD NR<br>Subscale M/SD NR	5	None
Zahiriharsini et al., 2022<br><br>Narrative synthesis	Cross-sectional	572	Healthcare workers and leaders<br>Nurse (60.5%)<br>Patient support worker (2.9%)<br>Physician (1%)<br>Manager (11.1)<br>Others (25%)	Convenience February - June 2021	40.30 (10.5)	11.4 %	NR	Canada Quebec	NR	EMIS-M low MI: <2.5, moderate MI: 2.5 ≤ score <3.5, and high MI: ≤ 3.5<br><br>2 subscales (Self-directed MI and other-directed MI)	NR at univariate level	Overall %<br>Moderate to high: 79 (13.81%)<br>Subscale %<br>Self-directed MI:<br>Moderate to high: 58 (10.14%)<br>Other-directed MI:<br>Moderate to high: 167 (29.19%)	Overall M/SD: NR<br>Subscale M/SD: NR	5	None

Levi-Belz Shemesh & Zerach 2022	Cross-sectional	190	Combat veterans.<br>Infantry units (58.4%)<br>Armoured corps (13.2%)<br>Artillery corps (7.4%)<br>Navy (4.7%)<br>Military police, intelligence, or Air Force (13.6%)	Convenience<br><br>January - July 2018.	26 (2.61)	87%	NR	Israel	NR	MIES 4+ (slightly agree)<br><br>3 subscales (transgressions-others, transgressions-self, betrayal)<br><br>Average time since discharge 4.26(2.37)	(Mental) health<br>Depression (PHQ-8) (+)<br>Current suicide ideation (SBQ-R) (+) *<br>current suicide ideation (SBQ-R) (0) **<br>Self-disclosure (DDI) (0)<br>Individual/work characteristics<br>Combat exposure (+)<br>Time from demobilisation (0)<br>*Transgressions-self and betrayal<br>**Transgressions-others	Overall %<br>NR<br>Per subscale %<br>Transgressions-others: 69 (36.1%)<br>Transgressions-self: 44 (23.2%)<br>Betrayal: 67 (35.2%)<br>Per item %<br>Item 1: 61 (32.1%)<br>Item 2: 47 (24.7%)<br>Item 7: 52 (27.4%)	Overall M/SD<br>NR<br>Subscale M/SD<br>Transgressions-others: 2.41(1.49)<br>Transgressions-self: 1.81(1.08)<br>Betrayal: 2.02(1.06)	5	NR
Schwartz Halperin & Levi-Belz, 2022	Cross-sectional	336	Veterans<br><br>Armoured corps (9.2%)<br>Artillery corps (8.3%)<br>Combat engineering (8%)<br>Other (34.6%)	Convenience<br><br>August - November 2019	26.16 (3.06)	83.9 %	NR	Israel	58 %	MIES 4+ (slightly agree)<br><br>1 subscale (transgressions-self)<br><br>Average time since discharge 4.2(2.45)	(Mental) health<br>Shame (TRSI) (+)<br>Current suicidal ideation (SBQ-R) (+)<br>Individual/work characteristics<br>Collective hatred (HAS) (0)<br>All correlations are for transgressions-self subscale.	Overall %<br>NR<br>Per subscale %<br>NR<br>Per item %<br>Item 3: (24.18%)	Overall M/SD<br>NR<br>Subscale M/SD<br>Transgressions-self: 2.12(1.26)	5	None

Note. For longitudinal studies, data extracted from baseline wave unless only available at a subsequent wave. Associations reported use total scale but if no total scale associations are reported, then subscales associations were reported. CPS = Child Protection Services; RR = Response rate; NR = No response; MIES = Moral injury events scales; EMIS-M = Expressions of Moral Injury Scale-Military Version; MISS-HP = Moral Injury Symptoms Scale – Health Professionals; MIA-PSP = Moral Injury Assessment for Public Safety Personnel; MIQ-M = Moral Injury Questionnaire—Military version, BMIS = Brief Moral Injury Screen; BMIS-E = Brief Moral Injury Screen Event subscale, EMIS-SF = Expressions of Moral Injury Scale-Short Form; MIQ-T = Moral Injury Questionnaire—Teacher version; MIQ-M = Moral Injury Questionnaire—Military version; MIOS = Moral Injury Outcome Scale

Supplementary Table 3.
Hoy Risk of Bias for studies included in the systematic review

Authors (Year)	Representative of national population	Representative of target population	Random selection	Non-response bias	Directly collected	Acceptable case definition	Reliable and valid instrument	Same mode of data collection	Appropriate numerator(s) and denominator(s) for the parameter of interest	Hoy score
**PTED only studies**										
Dunn & Sensky 2018	1	1	1	1	0	0	0	0	1	5
Linden & Rotter 2019	1	1	1	1	0	0	0	0	1	5
Michailidis & Cropley 2018	1	1	1	1	0	0	0	0	1	5
Sensky, Salimu, Ballard & Pereira 2015	1	0	1	1	0	0	0	0	1	4
Sabic, Sabic, & O Baltic-Mujanovic 2018	1	1	1	1	0	0	0	0	1	5
Michailids & Cropley 2017	1	1	1	1	0	0	0	0	1	5
Karatuna & Gok 2014	1	1	1	1	0	0	0	0	1	5
Rubab & Tariq 2022	1	1	1	1	0	0	0	0	1	5
Saleem, Bashir & Abrar 2022	1	1	0	1	0	0	0	1	1	5
Shin & You 2021	1	1	1	1	0	0	0	0	1	5
**Moral injury and PTED study**										

Brennan, McKay & Cole 2022	1	0	1	0	0	0	0	0	0	2
**Moral injury only studies**										
Bryan et al., 2016	1	1	1	1	0	0	0	0	1	5
Currier, Holland, Drescher & Foy 2015	0	0	1	1	0	0	0	0	1	3
Currier, Holland, Rojas-Flores, Herrera & Foy 2015	1	1	1	0	0	0	0	0	1	4
Currier et al., 2017	1	1	1	1	0	0	0	0	1	5
Forkus,Juliana & Weiss 2019	1	1	1	1	0	0	0	0	1	5
Frankfurt et al., 2018.	1	0	1	1	0	0	0	0	1	4
Griffin et al., 2020	1	1	1	1	0	0	0	0	1	5
Jinkerson & Battles 2019	1	1	1	0	0	0	0	0	1	4
Jordan & Nash 2017	1	1	1	1	0	0	0	0	1	5
Lee, Aldwin & Kang 2022	1	1	1	1	0	0	0	0	1	5
Ogle, Reichwald, & Rutland 2018	1	1	1	1	0	0	0	0	1	5
Wisco et al., 2017	0	0	0	1	0	0	0	0	0	1
Zerach & Levi-Belz., 2017	1	1	1	0	0	0	0	0	1	4
Papazoglou et al., 2019	1	1	1	0	0	0	0	0	1	4
Richardson, Chesnut et al., 2020	0	0	0	1	0	0	0	0	1	2
Feinstein, Pavisian, Storm., 2018	1	1	1	1	0	0	0	0	1	5
Sugrue 2020	1	1	1	1	0	0	0	0	1	5

Aldridge, Scott, Paskell 2019	1	1	1	1	0	0	0	0	1	5
Andrukonis, Protopopov. 2020	1	1	1	1	0	0	0	0	1	5
Haight, Sugrue & Calhoun 2017	1	1	1	1	0	0	0	0	1	5
Battles et al., 2019	1	1	1	1	0	0	0	0	1	5
Braitman et al., 2018	1	1	1	1	0	0	0	0	1	5
Lancaster & Harris 2018	1	1	1	1	0	0	0	0	1	5
Lancaster 2018	1	1	1	1	0	0	0	0	1	5
Akhtar et al., 2022	1	1	1	1	0	0	0	1	1	6
Qi et al., 2022	1	1	1	1	0	0	0	0	1	5
Ansalem et al., 2021	1	1	1	1	0	0	0	0	1	5
Perez, Larson & Bair 2021	1	1	1	1	0	0	0	0	1	5
Benatoz, Zerach & Levi-Belz 2022	1	1	1	1	0	0	0	0	1	5
Borges et al., 2021	1	1	1	1	0	0	0	0	1	5
Boscarino et al., 2022	1	0	0	1	0	0	0	0	1	3
Brady et al., 2021	0	0	1	1	0	0	0	0	1	4
Brady et al., 2022	1	1	1	1	0	0	0	0	1	6
Chandrabhatla et al., 2022	1	1	1	1	0	0	0	0	1	5
Dale et al., 2021	1	1	1	1	0	0	0	0	1	5
Plouffe et al., 2021	0	0	0	1	0	0	0	0	1	2

Brennan CJ, *et al. BMJ Open* 2024; 14:e071776. doi: 10.1136/bmjopen-2023-071776

Nichter et al., 2020	0	0	0	1	0	0	0	0	0	1
Fitzpatrick et al., 2022	1	1	1	1	0	0	0	0	1	5
Hagerty & Williams 2022	1	1	1	1	0	0	0	0	1	5
Hamrick et al., 2022	1	1	1	1	0	0	0	0	1	5
Hines et al., 2020	1	1	1	1	0	0	0	0	1	5
Hinkel et al., 2022	1	1	1	1	0	0	0	0	1	5
Kelley et al., 2021	1	1	1	1	0	0	0	0	1	5
La Fleur et al., 2020	1	1	1	1	0	0	0	1	1	6
Lamb et al., 2021	1	0	0	1	0	0	0	0	0	2
Litam et al., 2021	1	1	1	1	0	0	0	0	1	5
Litz et al., 2022 Samples 1, 2 and 3	1	1	1	1	0	0	0	0	1	5
Litz et al., 2022 Sample 4	1	1	1	1	0	0	0	1	1	6
Maftei & Holman 2021	1	1	1	1	0	0	0	0	1	5
Maguen et al., 2021	0	0	0	1	0	0	0	0	1	2
Malakoutikhah et al., 2022	1	1	1	1	0	0	0	0	1	5
Mantri et al., 2020	1	1	1	1	0	0	0	0	1	5
Mantri et al., 2021	1	1	1	1	0	0	0	0	1	5
Morris, Webb & Devlin 2022	1	1	1	1	0	0	0	0	1	5

Rushton et al., 2022	1	1	1	1	0	0	0	0	1	5
Nieuwsma et al., 2020	1	1	1	1	0	0	0	0	1	5
Osmann et al., 2022	1	1	1	1	0	0	0	0	1	5
Rodríguez et al., 2021	1	1	1	1	0	0	0	0	1	5
Roth et al., 2022	1	1	1	1	0	0	0	0	1	5
Russel & Mussap 2022	1	1	1	1	0	0	0	0	1	5
Saba et al., 2022	1	1	1	1	0	0	0	0	1	5
Senger, Torres & Ratcliff 2022	1	1	1	1	0	0	0	1	1	6
Stanojević & Čartolovni 2022	1	1	1	1	0	0	0	0	1	5
Testoni et al., 2022	1	1	1	1	0	0	0	0	1	5
Thomas et al., 2022	1	1	1	1	0	0	0	0	1	5
Trifunovic-Koenig, et al 2022	1	1	1	1	0	0	0	0	1	5
Ulusoy & Çelik 2022	1	1	1	1	0	0	0	0	1	5
Williamson, Murphy & Greenberg 2022	1	1	1	1	0	0	0	0	1	5
Zahiriharsini et al., 2022	1	1	1	1	0	0	0	0	1	5
Levi-Belz, Shemesh & Zerach 2022	1	1	1	1	0	0	0	0	1	5
Schwartz, Halperin & Levi-Belz 2022	1	1	1	1	0	0	0	0	1	5

Note. 1 = yes (high risk), 0 = no (low risk) 7-9 = High, 4-6 = Medium, 0-3 = Low. PTED = post-traumatic embitterment disorder

Supplementary Figures: Forest Plots for Meta-Analysis Findings

*Analyses using **prevalence** estimates as outcome*

Supplementary Figure 1.
PTED pooled prevalence estimates, using 1.6 cut off.

Study	Cases	Total	Prevalence	95% C.I.
Brennan 2022	121	400	30.25	[25.78; 35.01]
Dunn 2018	37	79	46.84	[35.51; 58.40]
Katatuna 2014	106	397	26.70	[22.41; 31.34]
Michailids 2018	55	352	15.62	[11.99; 19.85]
Michailids 2017	253	337	75.07	[70.10; 79.60]
Rubab 2022	121	300	40.33	[34.74; 46.12]
Sabic 2018	15	87	17.24	[9.98; 26.84]
Sensky 2015	68	236	28.81	[23.12; 35.05]
Shin 2022	581	1074	54.10	[51.06; 57.11]
**Random effects model**		**.**	**36.65**	**[24.38; 49.85]**

Heterogeneity: $I^2 = 98\%$, $^2 = 0.0403$, $^2_8 = 455.07$ ($p < 0.01$)

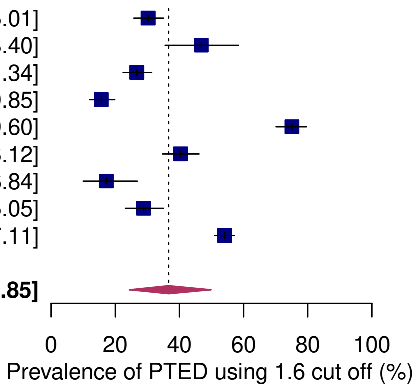

Supplementary Figure 2.
PTED pooled prevalence estimates, using 2 cut off.

Study	Cases	Total	Prevalence	95% C.I.
Brennan 2022	77	400	19.25	[15.50; 23.46]
Dunn 2018	28	79	35.44	[25.00; 47.01]
Katatuna 2014	74	397	18.64	[14.93; 22.83]
Linden 2019	26	102	25.49	[17.38; 35.08]
Michailidis 2017	209	337	62.02	[56.60; 67.22]
Rubab 2022	77	300	25.67	[20.82; 31.00]
Sabic 2018	9	87	10.34	[4.84; 18.73]
Sensky 2015	43	236	18.22	[13.51; 23.75]
**Random effects model**		**.**	**26.03**	**[16.09; 37.38]**

Heterogeneity: $I^2 = 97\%$, $^2 = 0.0293$, $^2_7 = 232.77$ ($p < 0.01$)

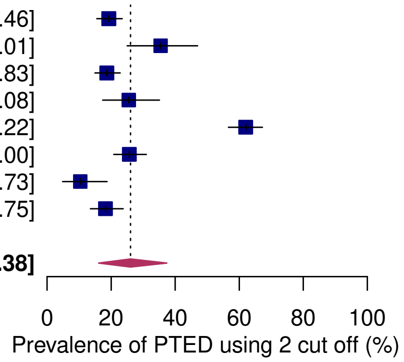

Supplementary Figure 3.
PTED pooled prevalence estimates, using 2.5 cut off.

Study	Cases	Total	Prevalence	95% C.I.
Brennan 2022	30	400	7.50	[5.12; 10.53]
Katatuna 2014	41	397	10.33	[7.51; 13.75]
Michailidis 2017	152	337	45.10	[39.71; 50.59]
Rubab 2022	26	300	8.67	[5.74; 12.44]
Sabic 2018	1	87	1.15	[0.03; 6.24]
Sensky 2015	21	236	8.90	[5.59; 13.28]
Shin 2022	102	1074	9.50	[7.81; 11.41]
**Random effects model**		**.**	**11.21**	**[4.02; 21.32]**

Heterogeneity: I^2 = 97%, ^{2} = 0.0334, $^2_6$ = 237.59 (p < 0.01)

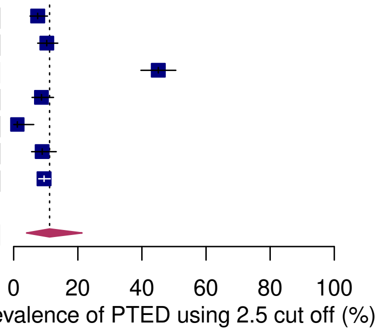

Supplementary Figure 4.
Any MIES item pooled prevalence estimates, split by occupation.

Study	Cases	Total	Prevalence	95% C.I.
Healthcare				
Amsalem 2021	267	350	76.29	[71.47; 80.65]
Borges 2021	109	211	51.66	[44.70; 58.57]
Brady 2021	237	390	60.77	[55.73; 65.65]
Brady 2022	268	377	71.09	[66.23; 75.62]
Brennan 2022	289	400	72.25	[67.58; 76.58]
Chandrabhatla 2022	60	78	76.92	[66.00; 85.71]
Lamb 2021	602	2166	27.79	[25.91; 29.73]
Maftei 2021	80	114	70.18	[60.89; 78.38]
Morris 2022	183	233	78.54	[72.71; 83.63]
Testoni 2022	212	270	78.52	[73.13; 83.27]
Ulusoy 2022	116	124	93.55	[87.68; 97.17]
Williamson 2022	80	90	88.89	[80.51; 94.54]
Random effects model		.	**71.40**	**[60.72; 81.01]**
Heterogeneity: I^2 = 99%, ^{2} = 0.0382, $^2_{11}$ = 1160.88 (p < 0.01)				
Armed forces				
Lee 2020	176	354	49.72	[44.39; 55.05]
Maguen 2022	7830	14057	55.70	[54.88; 56.53]
Nichter 2021	535	1321	40.50	[37.84; 43.20]
Russell 2022 study one	132	183	72.13	[65.04; 78.49]
Russell 2022 study two	300	393	76.34	[71.82; 80.45]
Saba 2022	881	1005	87.66	[85.47; 89.63]
Wisco 2017	223	564	39.54	[35.48; 43.71]
Zerach 2017	92	191	48.17	[40.90; 55.50]
Random effects model		.	**59.53**	**[45.90; 72.44]**
Heterogeneity: I^2 = 99%, ^{2} = 0.0382, $^2_7$ = 830.68 (p < 0.01)				
First responders				
Papazoglou 2019	289	390	74.10	[69.45; 78.38]
Senger 2022	98	242	40.50	[34.26; 46.97]
Random effects model		.	**57.86**	**[30.91; 82.51]**
Heterogeneity: I^2 = 99%, ^{2} = 0.0382, $^2_1$ = 71.65 (p < 0.01)				
Education				
Sugrue 2020	191	218	87.61	[82.49; 91.68]
**Random effects model**		**.**	**67.00**	**[59.01; 74.54]**

Heterogeneity: I^2 = 99%, ^{2} = 0.0398, $^2_{22}$ = 2212.51 (p = 0)
Residual heterogeneity: I^2 = 99%, ^{2} = 0.0382, $^2_{19}$ = 2063.21 (p = 0)
Test for subgroup differences: $^2_3$ = 3.95, df = 3 (p = 0.27)

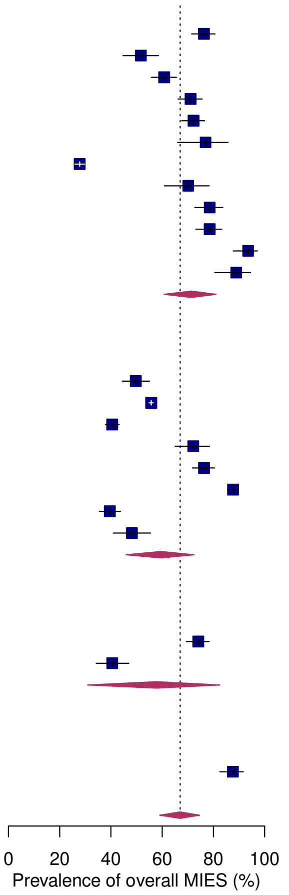

Supplementary Figure 5.
MIES pooled perpetration prevalence estimates.

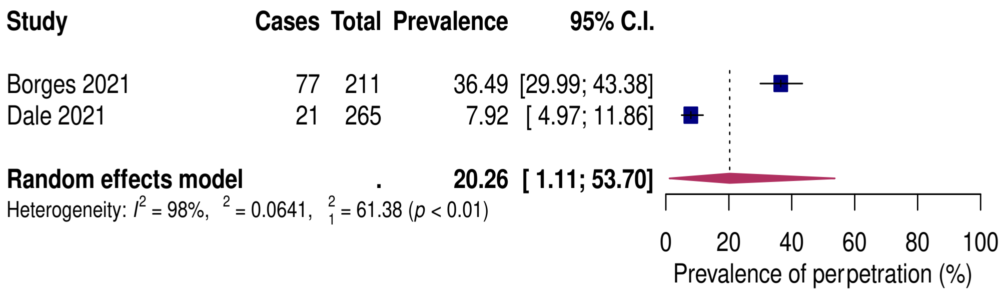

Supplementary Figure 6.
MIES transgressions-self pooled prevalence estimates, split by occupation.

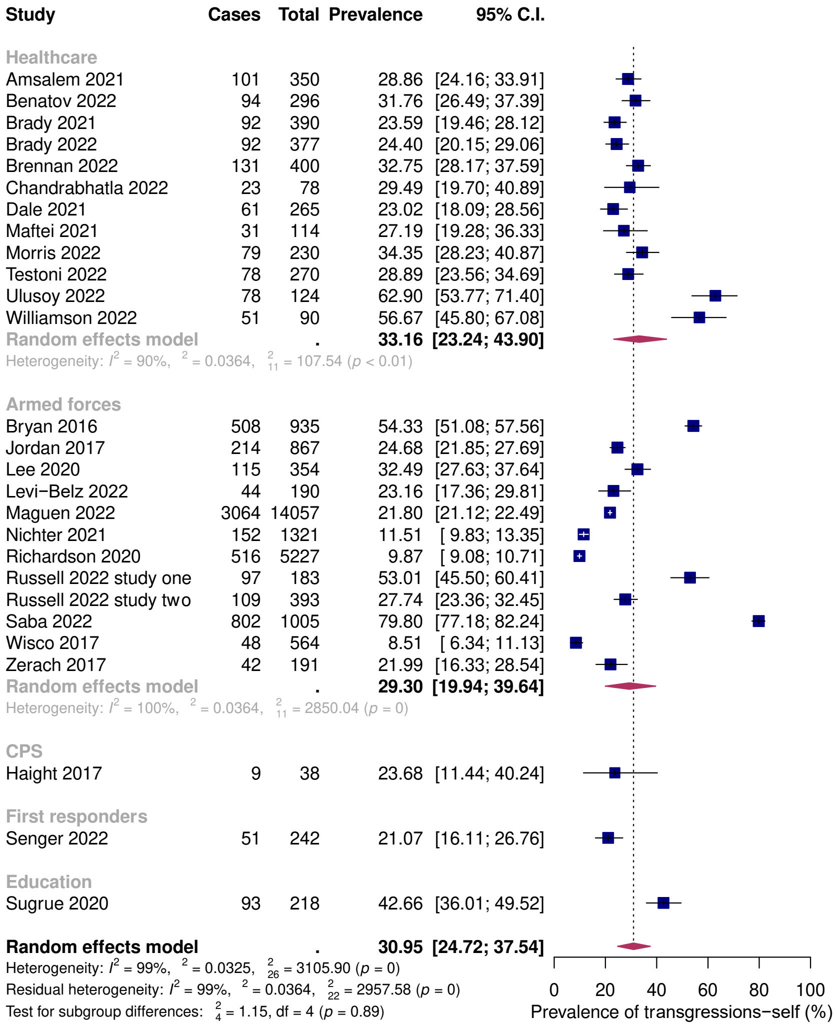

Supplementary Figure 7.

MIES transgressions-others pooled prevalence estimates, split by occupation.

Study	Cases	Total	Prevalence	95% C.I.
**Healthcare**				
Amsalem 2021	215	350	61.43	[56.11; 66.56]
Benatov 2022	146	296	49.32	[43.49; 55.17]
Brady 2021	189	390	48.46	[43.40; 53.55]
Brady 2022	197	377	52.25	[47.08; 57.39]
Brennan 2022	213	400	53.25	[48.23; 58.22]
Chandrabhatla 2022	49	77	63.64	[51.88; 74.30]
Dale 2021	6	265	2.26	[0.84; 4.86]
Maftei 2021	61	114	53.51	[43.93; 62.90]
Morris 2022	154	230	66.96	[60.47; 73.00]
Testoni 2022	166	270	61.48	[55.39; 67.32]
Ulusoy 2022	109	124	87.90	[80.83; 93.07]
Williamson 2022	63	90	70.00	[59.43; 79.21]
**Random effects model**		.	**54.88**	**[43.91; 65.62]**
Heterogeneity: I^2 = 98%, ^{2} = 0.0360, ${}^2_{11}$ = 591.65 (p < 0.01)				
**Armed forces**				
Bryan 2016	363	935	38.82	[35.69; 42.03]
Lee 2020	130	354	36.72	[31.69; 41.98]
Levi−Belz 2022	69	190	36.32	[29.48; 43.59]
Maguen 2022	6059	14057	43.10	[42.28; 43.93]
Nichter 2021	316	1321	23.92	[21.64; 26.32]
Russell 2022 study one	48	183	26.23	[20.02; 33.23]
Russell 2022 study two	62	393	15.78	[12.31; 19.76]
Saba 2022	303	1005	30.15	[27.32; 33.09]
Wisco 2017	126	564	22.34	[18.97; 26.01]
Zerach 2017	64	191	33.51	[26.86; 40.68]
**Random effects model**		.	**30.36**	**[20.07; 41.76]**
Heterogeneity: I^2 = 98%, ^{2} = 0.0360, ${}^2_{9}$ = 475.46 (p < 0.01)				
**CPS**				
Haight 2017	35	38	92.11	[78.62; 98.34]
**First responders**				
Senger 2022	147	242	60.74	[54.28; 66.94]
**Education**				
Sugrue 2020	175	218	80.28	[74.37; 85.34]
**Random effects model**		.	**47.73**	**[38.04; 57.51]**

Heterogeneity: I^2 = 98%, ^{2} = 0.0607, ${}^2_{24}$ = 1464.59 (p < 0.01)

Residual heterogeneity: I^2 = 98%, ^{2} = 0.0360, ${}^2_{20}$ = 1067.12 (p < 0.01)

Test for subgroup differences: ${}^2_{4}$ = 20.01, df = 4 (p < 0.01)

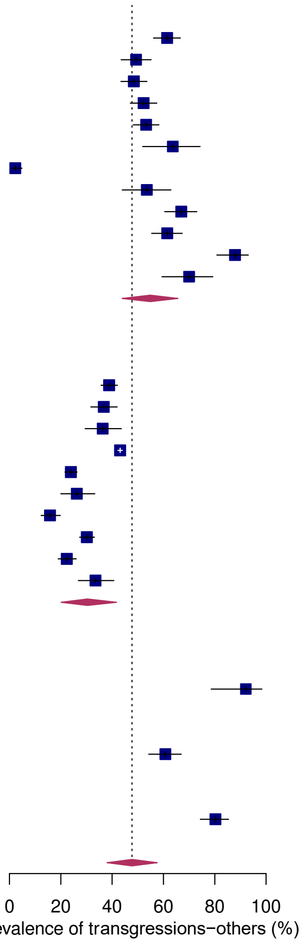

Supplementary Figure 8.
MIES betrayal pooled prevalence estimates, split by occupation.

Study	Cases	Total	Prevalence	95% C.I.
Healthcare				
Amsalem 2021	205	350	58.57	[53.21; 63.78]
Benatov 2022	184	296	62.16	[56.37; 67.71]
Borges 2021	88	211	41.71	[34.98; 48.67]
Brady 2021	166	390	42.56	[37.60; 47.64]
Brady 2022	210	377	55.70	[50.53; 60.79]
Brennan 2022	227	400	56.75	[51.74; 61.66]
Chandrabhatla 2022	37	78	47.44	[36.01; 59.07]
Maftei 2021	66	114	57.89	[48.29; 67.08]
Morris 2022	154	233	66.09	[59.63; 72.15]
Testoni 2022	166	270	61.48	[55.39; 67.32]
Ulusoy 2022	96	124	77.42	[69.04; 84.44]
Williamson 2022	62	90	68.89	[58.26; 78.23]
Random effects model		.	**58.12**	**[50.96; 65.12]**
Heterogeneity: I^2 = 89%, ^{2} = 0.0147, $^2_{11}$ = 98.31 (p < 0.01)				
Armed forces				
Bryan 2016	540	935	57.75	[54.51; 60.94]
Jordan 2017	252	867	29.07	[26.06; 32.21]
Lamb 2021	439	2166	20.27	[18.59; 22.02]
Lee 2020	121	354	34.18	[29.25; 39.38]
Levi−Belz 2022	67	190	35.26	[28.49; 42.51]
Maguen 2022	5651	14057	40.20	[39.39; 41.02]
Nichter 2021	189	1321	14.31	[12.46; 16.31]
Russell 2022 study one	99	183	54.10	[46.59; 61.47]
Russell 2022 study two	112	393	28.50	[24.09; 33.24]
Saba 2022	300	1005	29.85	[27.03; 32.79]
Wisco 2017	142	564	25.18	[21.64; 28.97]
Zerach 2017	59	191	30.89	[24.42; 37.97]
Random effects model		.	**32.67**	**[26.29; 39.38]**
Heterogeneity: I^2 = 99%, ^{2} = 0.0147, $^2_{11}$ = 1001.5 (p < 0.01)				
CPS				
Haight 2017	12	38	31.58	[17.50; 48.65]
First responders				
Papazoglou 2019	250	390	64.10	[59.12; 68.87]
Senger 2022	97	242	40.08	[33.86; 46.55]
Random effects model		.	**52.32**	**[35.23; 69.13]**
Heterogeneity: I^2 = 97%, ^{2} = 0.0147, $^2_{1}$ = 35.05 (p < 0.01)				
Education				
Sugrue 2020	148	218	67.89	[61.25; 74.04]
**Random effects model**		.	**46.03**	**[39.54; 52.59]**

Heterogeneity: I^2 = 98%, ^{2} = 0.0300, $^2_{27}$ = 1764.34 (p = 0)
Residual heterogeneity: I^2 = 98%, ^{2} = 0.0147, $^2_{23}$ = 1134.87 (p < 0.01)
Test for subgroup differences: $^2_{4}$ = 30.29, df = 4 (p < 0.01)

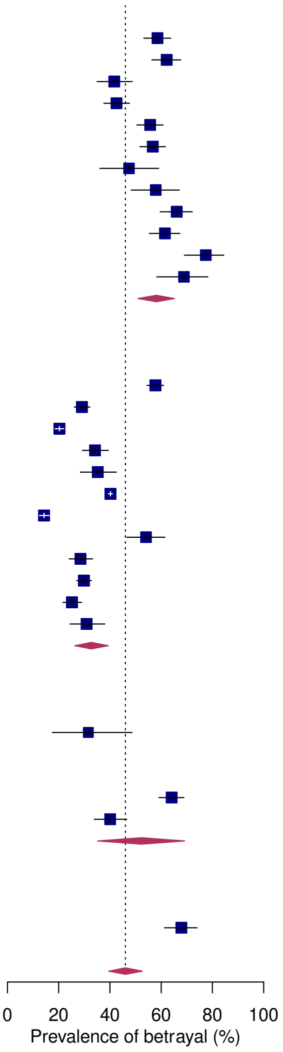

Brennan CJ, *et al. BMJ Open* 2024; 14:e071776. doi: 10.1136/bmjopen-2023-071776

Supplementary Figure 9.
MISS-HP pooled prevalence estimates.

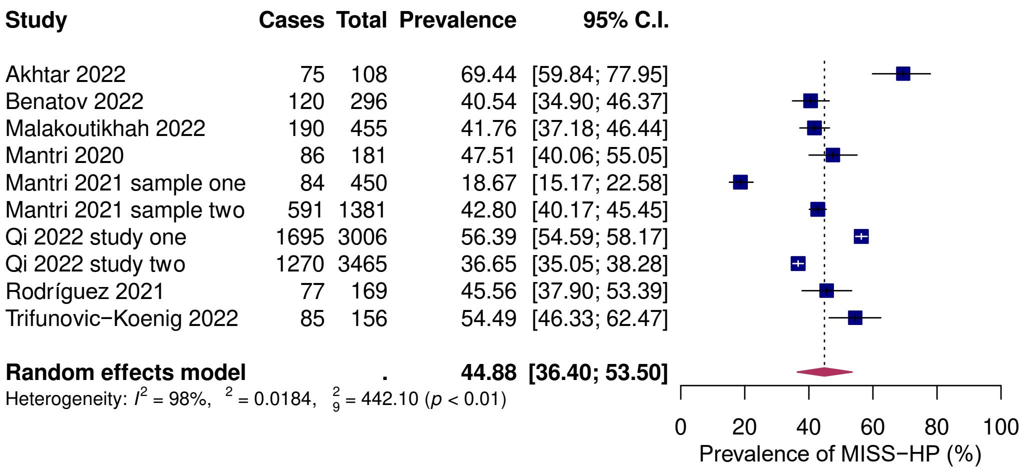

*Analyses using **pooled mean** estimates for PTED and moral injury, split by occupation*

Supplementary Figure 10.
PTED pooled mean estimates, split by occupation.

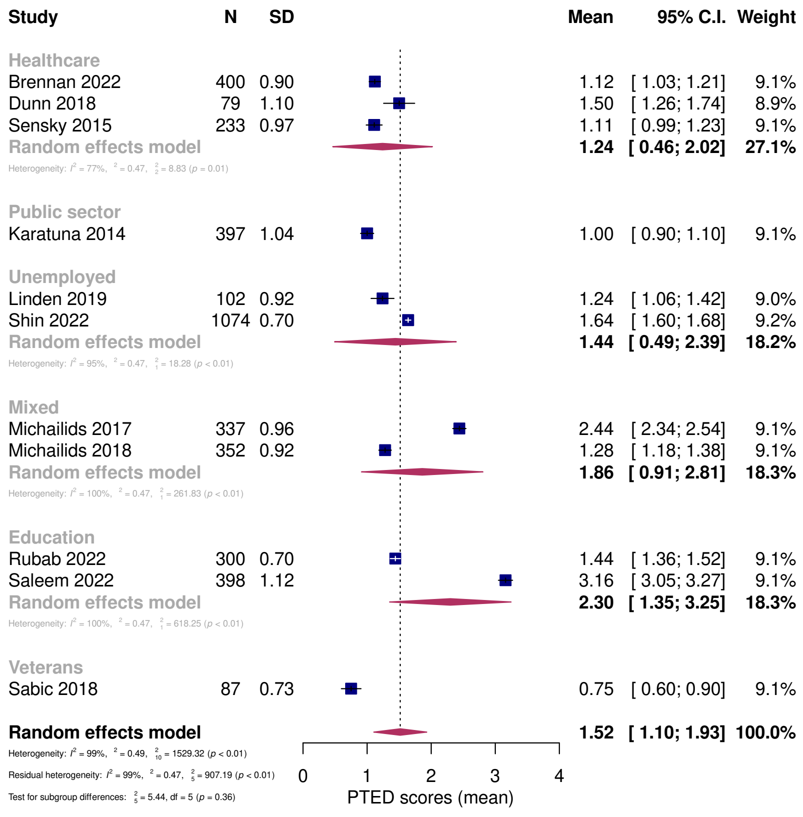

Brennan CJ, *et al. BMJ Open* 2024; 14:e071776. doi: 10.1136/bmjopen-2023-071776

Supplementary Figure 11.
Overall MIES pooled mean estimates, split by occupation.

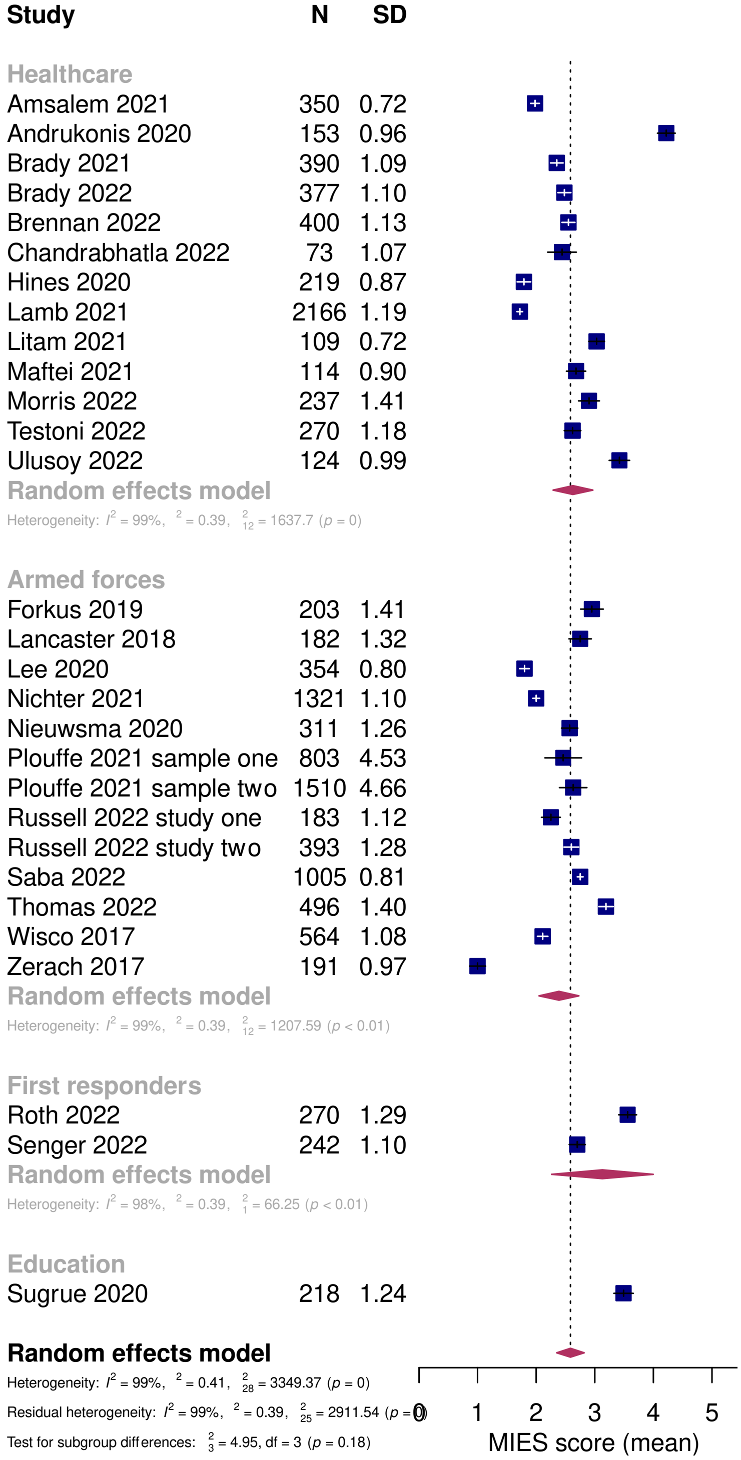

Supplementary Figure 12.

MIES perpetration pooled mean estimates.

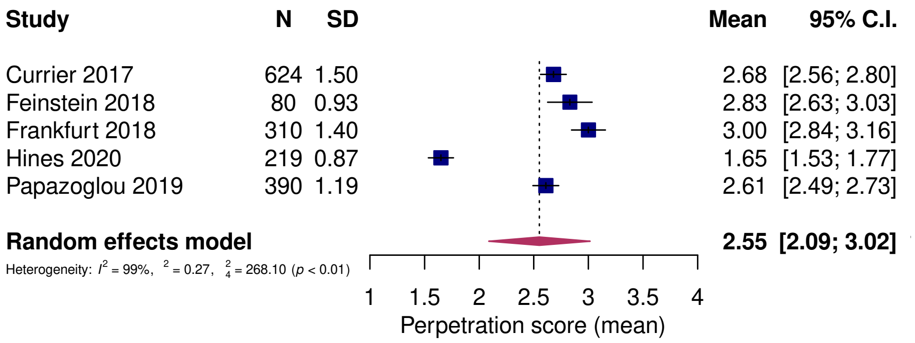

Supplementary Figure 13.

MIES transgressions-self pooled mean estimates, split by occupation.

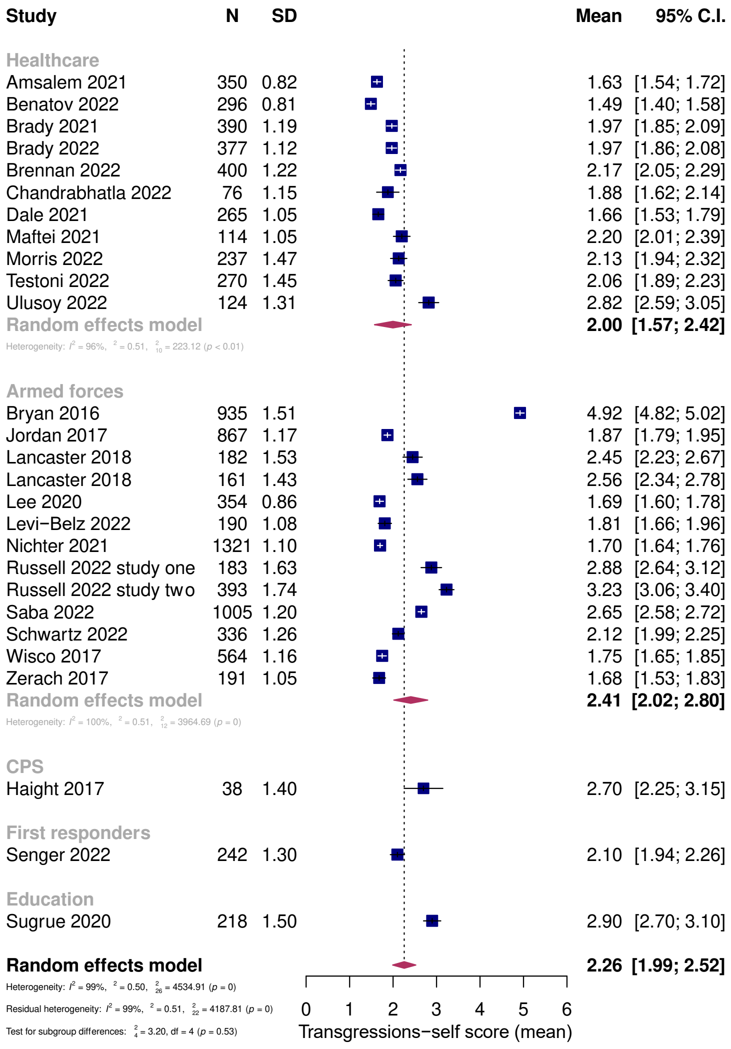

Brennan CJ, *et al. BMJ Open* 2024; 14:e071776. doi: 10.1136/bmjopen-2023-071776

Supplementary Figure 14.

MIES transgressions-others pooled mean estimates, split by occupation.

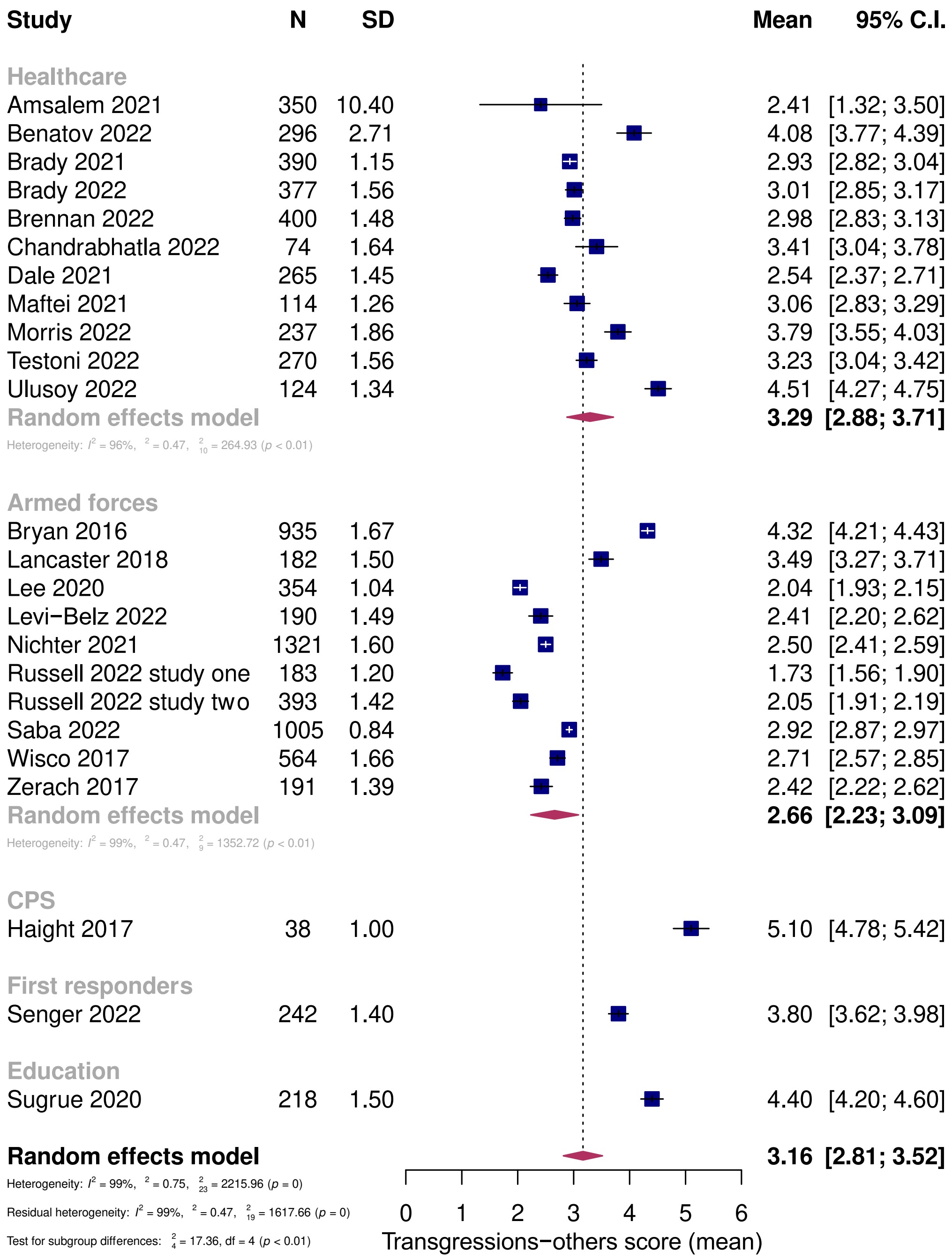

Brennan CJ, *et al. BMJ Open* 2024; 14:e071776. doi: 10.1136/bmjopen-2023-071776

Supplementary Figure 15.
MIES betrayal pooled mean estimates, split by occupation.

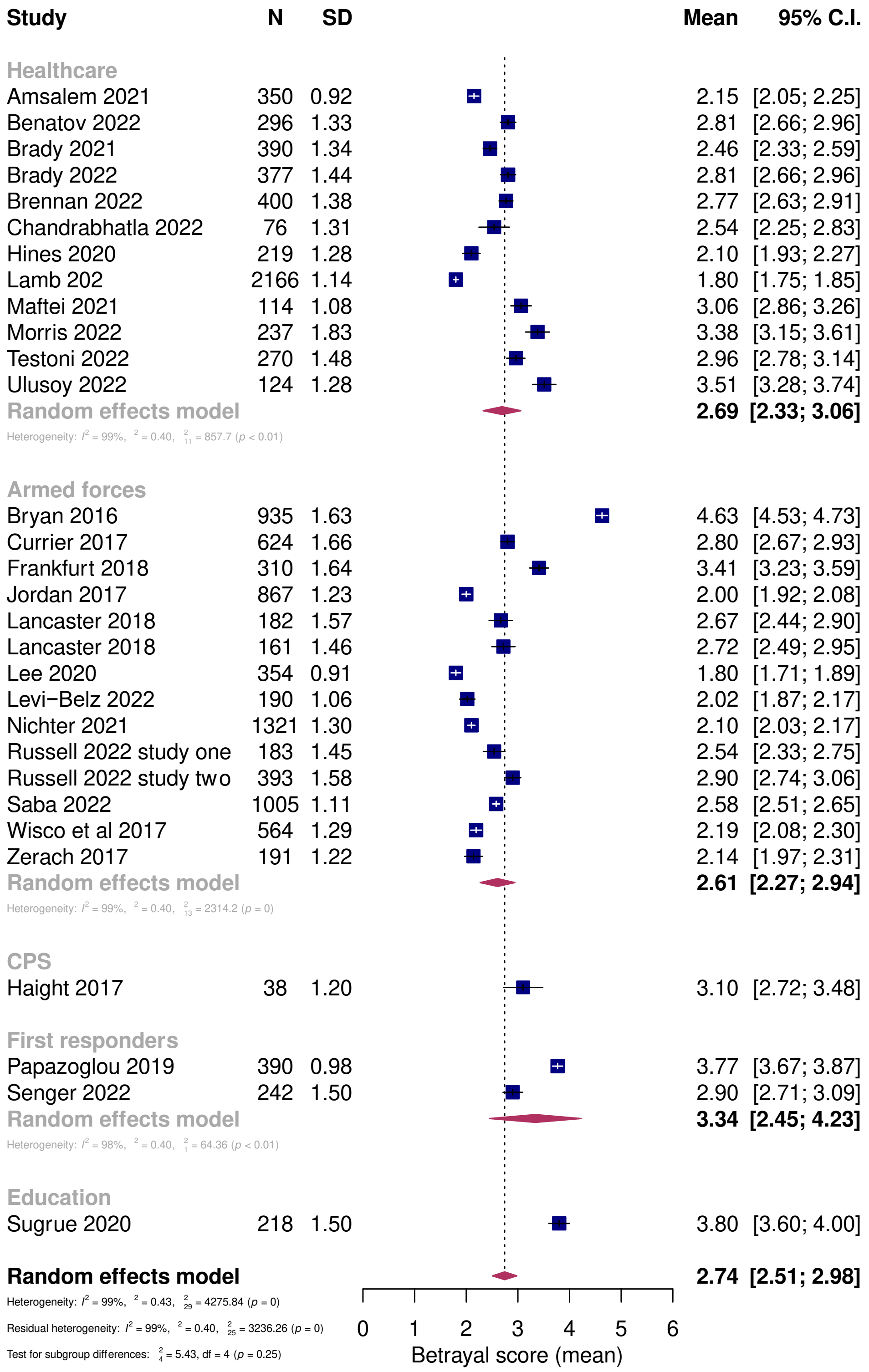

Brennan CJ, *et al. BMJ Open* 2024; 14:e071776. doi: 10.1136/bmjopen-2023-071776

Supplementary Figure 16.
MIQ-M total pooled mean estimates.

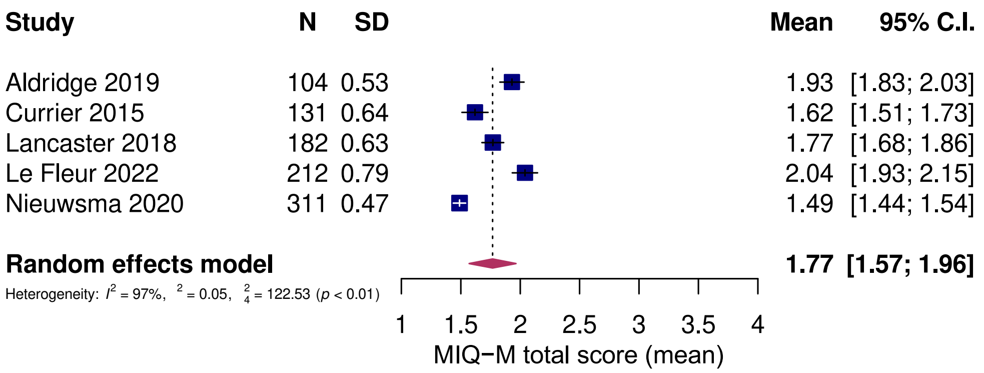

Supplementary Figure 17.
MIQ-M causes pooled mean estimates.

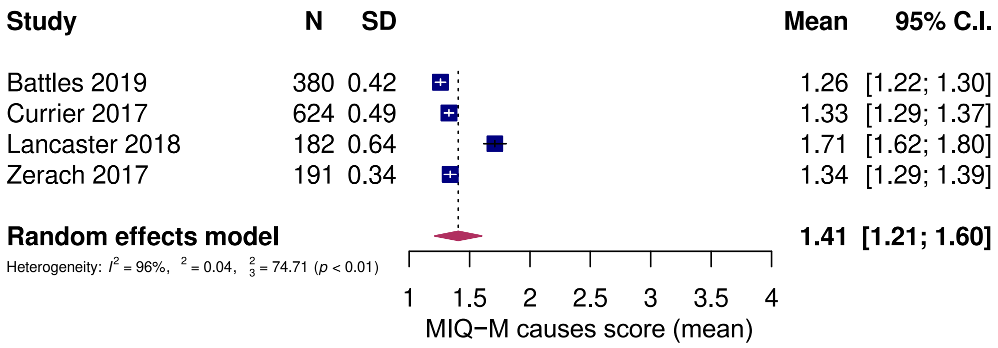

Supplementary Figure 18.
EMIS total pooled mean estimates.

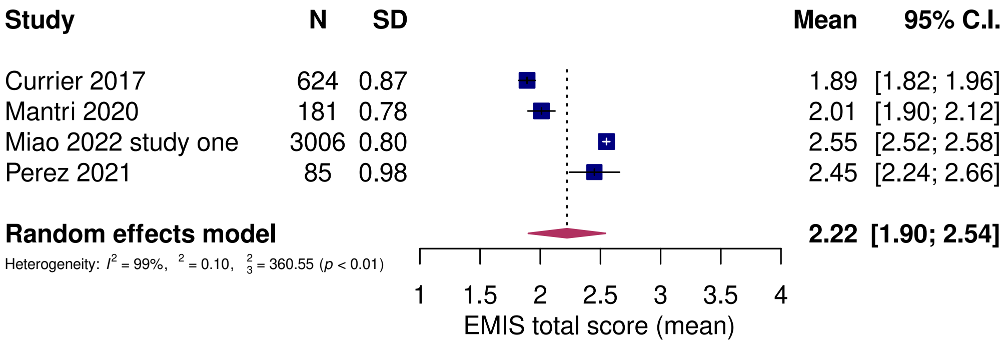

Brennan CJ, *et al. BMJ Open* 2024; 14:e071776. doi: 10.1136/bmjopen-2023-071776

Supplementary Figure 19.
EMIS self pooled mean estimates.

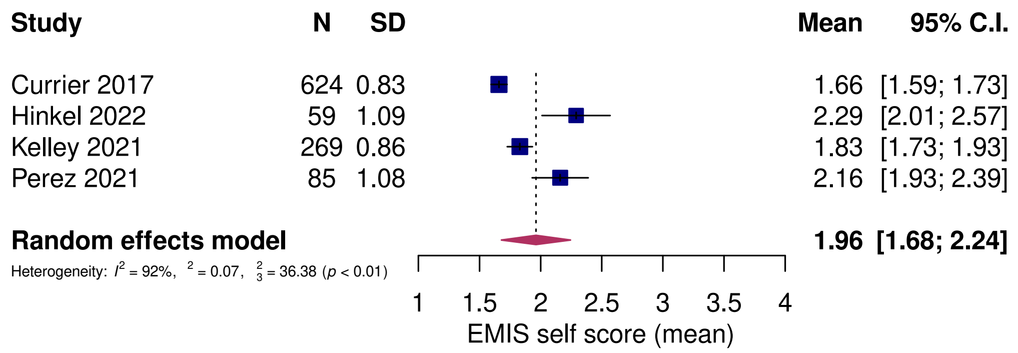

Supplementary Figure 20.
EMIS other pooled mean estimates.

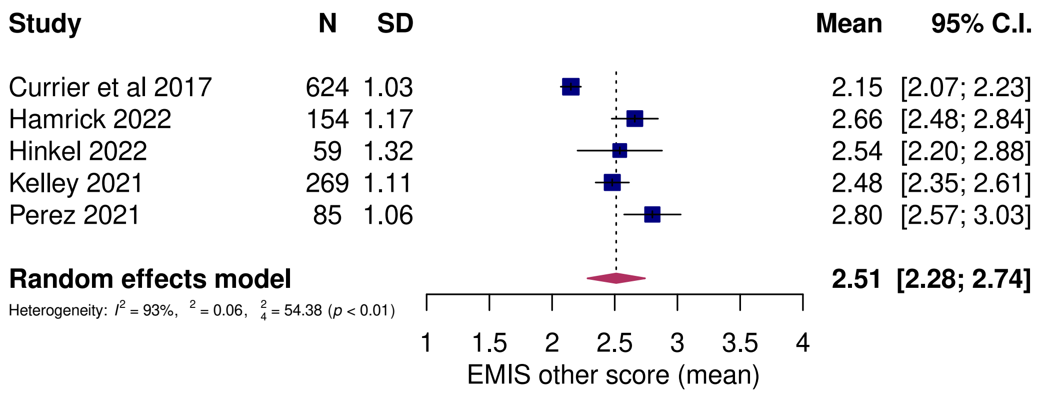

Supplementary Figure 21.
MISS-HP pooled mean estimates.

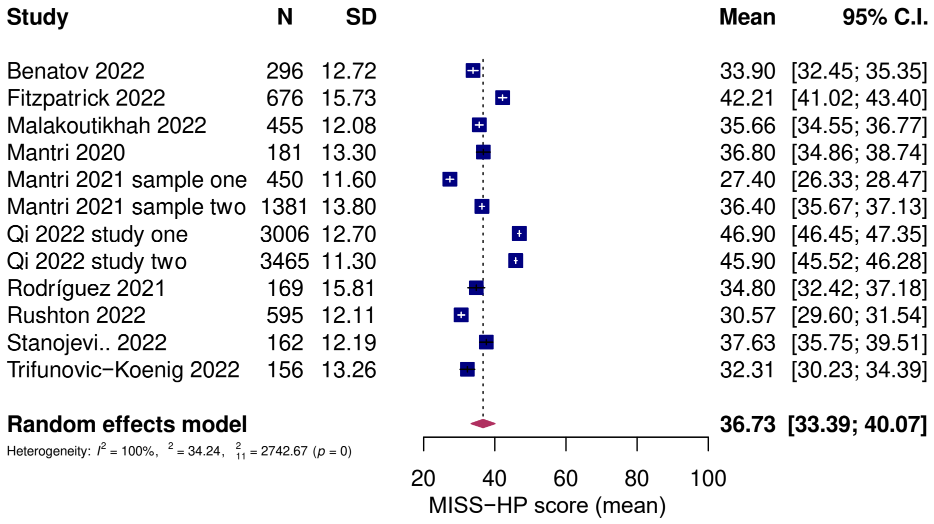

Brennan CJ, *et al. BMJ Open* 2024; 14:e071776. doi: 10.1136/bmjopen-2023-071776

Supplementary Figure 22.

MIOS total pooled mean estimates.

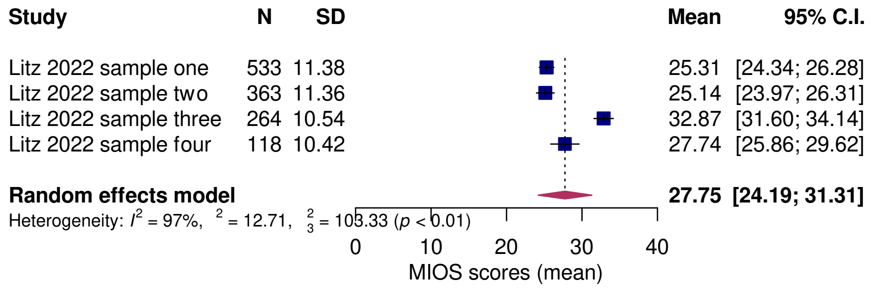

Supplementary Figure 23.

MIOS shame pooled mean estimates.

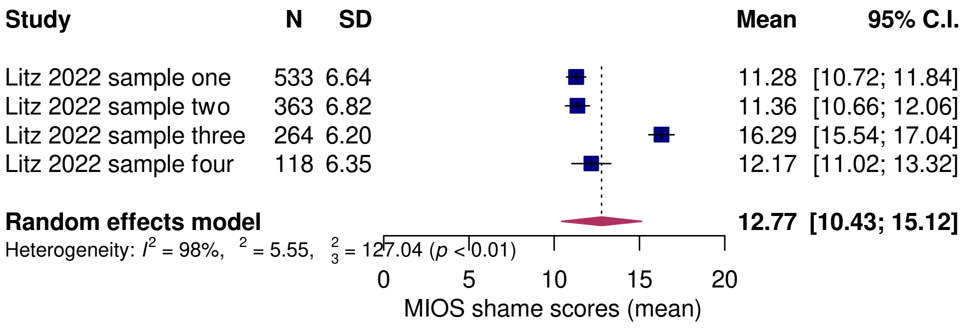

Supplementary Figure 24.

MIOS trust pooled mean estimates.

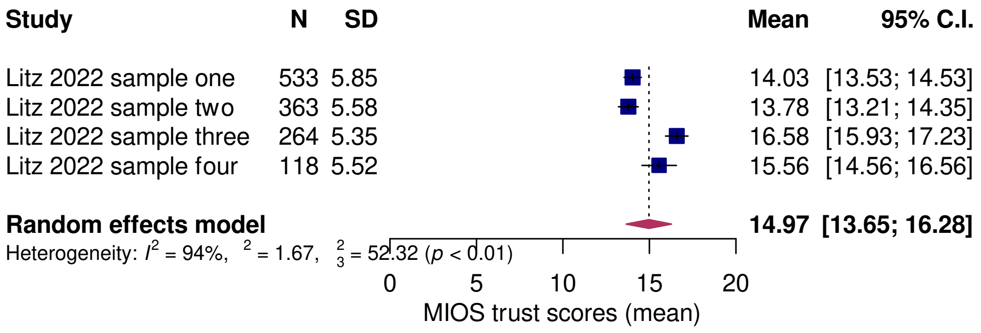

Supplementary Table 4.

Subgroup analyses based on study quality (mean and prevalence)

Measure	Medium risk			Low risk			
	k	Pooled mean [95% CI]	I^2	*k*	Pooled mean [95% CI]	I^2	P value
PTED	10	1.56 [1.11, 2.01]	99%	1	1.12 [1.03, 1.21]	-	0.566
MIES total	24	2.67 [2.42, 2.92]	99%	5	2.18 [1.62, 2.73]	97%	0.114
MIES transgressions-self	25	2.30 [2.02, 2.58]	99%	2	2.17 [0.75, 2.70]	0%	0.267
MIES transgressions-others	22	3.22 [2.85, 3.58]	99%	2	2.60 [1.40, 3.81]	85%	0.342
MIES betrayal	27	2.83 [2.60, 3.06]	99%	3	2.03 [1.33, 2.73]	97%	0.034
	k	Pooled prevalence (95% CI)	I^2	*k*	Pooled prevalence [95% CI]	I^2	P value
MIES	18	72.30 [64.83, 79.20]	97%	5	47.06 [32.42, 61.92]	99%	0.003
Transgressions-self	22	34.92 [28.32, 41.82]	99%	5	15.94 [84.7, 25.21]	98%	0.006
Transgressions-others	21	50.23 [39.64, 60.81]	98%	4	35.11 [14.67, 58.94]	99%	0.254
Betrayal	23	49.69 [43.09, 56.29]	97%	5	30.33 [18.51, 43.66]	99%	0.011

Note. PTED = post-traumatic embitterment disorder, MIES = Moral Injury Events Scale.

Brennan CJ, *et al. BMJ Open* 2024; 14:e071776. doi: 10.1136/bmjopen-2023-071776

Supplementary Table 5.
Subgroup analyses based on cut off value (prevalence)

Measure	Slightly agree		Moderately agree		Strongly agree		Agree		NR[a]		
	k	Pooled %	*k*	Pooled %	*k*	Pooled %	*k*	Pooled %	*k*	Pooled %	P value
MIES overall	14	72.47 [63.07, 80.98]	6	54.13 [38.88, 69]		N/A	2	73.36 [47.45, 92.77]	1	49.72 [15.70, 83.88]	0.152
Transgressions -self	20	31.93 [24.52, 39.81]	3	22.06 [74.1, 41.63]	1	54.33 [20.26, 86.24]	2	28.04 [84.7, 53.41]	1	23.02 [1.68, 58.11]	0.578
Transgressions -others	18	52.22 [41.78, 62.56]	3	37.46 [15.33, 62.77]	1	38.82 [53.39, 80.92]	2	57.56 [27, 85.26]	1	02.26 [0, 31.56]	0.057
Betrayal	19	46.87 [38.98, 54.83]	6	37.54 [24.56, 51.49]	1	57.75 [24.89, 87.16]	1	58.24 [33.89, 80.65]		-	0.409
Measure	28.5		36		36.5						
	k	Pooled %	*k*	Pooled %	*k*	Pooled %					P value
MISS-HP	1	54.49 [46.61, 62.25]	8	44.17 [33.84, 54.77]	1	41.76 [37.26, 46.33]					0.802

Note. [a] For MIES any this was "sometimes". MIES = Moral Injury Events Scale; MISS-HP = Moral Injury Symptom Scale-Healthcare Professionals

Brennan CJ, *et al. BMJ Open* 2024; 14:e071776. doi: 10.1136/bmjopen-2023-071776

Supplementary Table 6.
Prevalence and average scores for moral injury scales that were not combined for narrative synthesis (k=1)

**Measure**	**Mean(SD)**	**Measure**	**Prevalence (%)**
MIES-other (items 1-2, 7-9)	2.51 (1.40)	MIES-other (items 1-2, 7-9)	19.76%
MIES-commission (items 1-4)	1.71 (1.06)	MIES-commission (items 1-4)	14.28%
MIES-omission (items 5-6)	1.47 (1.1)	MIES-omission (items 5-6)	4.57%
MIQ-T	1.16 (0.56)	MIES-no moral distress	42%
MIQ-M modified		MIES-moral distress-other	19%
MIQ-M modified AoW	1.77 (0.63)	MIES-witnessing-only	16%
MIQ-M modified PCoW	1.99 (0.66)	MIES-moral distress-self	8%
MIQ-M modified LFB	2.12 (0.78)	MIES-moral distress-self and other	15%
MIQ-M modified AoW defining characteristics	2.21 (0.67)	EMIS-M self-directed ≥2.5 score <3.5	10.14%
MIQ-M modified PCoW defining characteristics	2.14 (0.66)	EMIS-M other-directed ≥2.5 score <3.5	29.19%
MIQ-M modified LFB defining characteristics	2.12 (0.67)		
BMIS total	3.71 (4.38)	BMIS event	44.1%
MIA-PSP total	59.6 (21.9)		
MIA-PSP emotional sequalae	25 (9.7)		
MIA-PSP perpetration	15 (8.5)		
MIA-PSP betrayal	19.6 (7.7)		
Toronto total	1.5 (1.27)		
Toronto organisation	1.54 (1.32)		
Toronto individual	1.18 (1.19)		
Toronto online	1.68 (1.27)		

Note. MI = moral injury; MIES = Moral Injury Events Scale; EMIS-M = Expressions of Moral Injury Scale-Military Version; MIA-PSP = Moral Injury Assessment for Public Safety Personnel; MIQ-M = Moral Injury Questionnaire—Military version; BMIS = Brief Moral Injury Screen; MIQ-T = Moral Injury Questionnaire—Teacher version; AoW = atrocities of war; LFB = leadership failure or betrayal; PCoW = psychological consequences of war
