# Supplementary material for: Prevalence of occupational moral injury and post-traumatic embitterment disorder: a systematic review and meta-analysis
Source: BMJ Open. 2024 Feb 20;14(2):e071776. doi: 10.1136/bmjopen-2023-071776 (PMC10882372; doi:10.1136/bmjopen-2023-071776)
Supplement: Author's manuscript [file bmjopen-2023-071776.draft_revisions.pdf]

BMJ Open is committed to open peer review. As part of this commitment we make the peer review history of every article we publish publicly available.

When an article is published we post the peer reviewers' comments and the authors' responses online. We also post the versions of the paper that were used during peer review. These are the versions that the peer review comments apply to.

The versions of the paper that follow are the versions that were submitted during the peer review process. They are not the versions of record or the final published versions. They should not be cited or distributed as the published version of this manuscript.

BMJ Open is an open access journal and the full, final, typeset and author-corrected version of record of the manuscript is available on our site with no access controls, subscription charges or pay-per-view fees (http://bmjopen.bmj.com).

If you have any questions on BMJ Open's open peer review process please email info.bmjopen@bmj.com

BMJ Open

A systematic review and meta-analysis on the prevalence of occupational moral injury and post-traumatic embitterment disorder

Journal:	*BMJ Open*
Manuscript ID	bmjopen-2023-071776
Article Type:	Original research
Date Submitted by the Author:	11-Jan-2023
Complete List of Authors:	Brennan, Chloe; University of Liverpool, Psychology<br>Roberts, Carl; Institute of Psychology, Health and Society, University of Liverpool, UK, Department of Psychological Sciences,<br>Cole, Jon; University of Liverpool
Keywords:	OCCUPATIONAL & INDUSTRIAL MEDICINE, Systematic Review, EPIDEMIOLOGY, MENTAL HEALTH, PSYCHIATRY

SCHOLARONE™
Manuscripts

I, the Submitting Author has the right to grant and does grant on behalf of all authors of the Work (as defined in the below author licence), an exclusive licence and/or a non-exclusive licence for contributions from authors who are: i) UK Crown employees; ii) where BMJ has agreed a CC-BY licence shall apply, and/or iii) in accordance with the terms applicable for US Federal Government officers or employees acting as part of their official duties; on a worldwide, perpetual, irrevocable, royalty-free basis to BMJ Publishing Group Ltd ("BMJ") its licensees and where the relevant Journal is co-owned by BMJ to the co-owners of the Journal, to publish the Work in this journal and any other BMJ products and to exploit all rights, as set out in our licence.

The Submitting Author accepts and understands that any supply made under these terms is made by BMJ to the Submitting Author unless you are acting as an employee on behalf of your employer or a postgraduate student of an affiliated institution which is paying any applicable article publishing charge ("APC") for Open Access articles. Where the Submitting Author wishes to make the Work available on an Open Access basis (and intends to pay the relevant APC), the terms of reuse of such Open Access shall be governed by a Creative Commons licence – details of these licences and which Creative Commons licence will apply to this Work are set out in our licence referred to above.

Other than as permitted in any relevant BMJ Author's Self Archiving Policies, I confirm this Work has not been accepted for publication elsewhere, is not being considered for publication elsewhere and does not duplicate material already published. I confirm all authors consent to publication of this Work and authorise the granting of this licence.

For peer review only

A systematic review and meta-analysis on the prevalence of occupational moral injury and post-traumatic embitterment disorder

Chloe J. Brennan[1], Carl A. Roberts[1], Jon C. Cole[1]

[1] Department of Psychology, University of Liverpool, United Kingdom

For peer review only

Keywords: Mental health, Psychiatry, Occupational & Industrial Medicine, Systematic review, Epidemiology

Word count: 5637

[1] **Correspondence to**
Chloe Brennan
Eleanor Rathbone Building
Bedford Street South
Liverpool
L69 7ZT
Telephone: +44 (0)151 794 2000
hlcbrenn@liverpool.ac.uk

For peer review only - http://bmjopen.bmj.com/site/about/guidelines.xhtml

Abstract:

Objective

There is no consensus on the prevalence of occupational moral injury and post-traumatic embitterment disorder (PTED), and whether this differs across occupations. This is the first meta-analysis to investigate the pooled prevalence and associates of occupational moral injury and PTED.

Design

A systematic review and meta-analysis.

Setting

International.

Participants

A total of 88 studies with samples size ranging from 38 – 14,057 were included.

Main outcome measures

The pooled prevalence, and norms of moral injury and PTED. Secondary outcome measures were the associates of moral injury and PTED.

Results

Of the 88 studies included, 77 measured moral injury only, 1 measured both moral injury and PTED and 10 measured PTED only. The number of studies included in each separate meta-analysis based on the measure used ranged from 2 to 30. The pooled prevalence of moral injury of clinical relevance in healthcare professionals was 45%, and pooled prevalence of exposure to any morally injurious event across occupations was 67%. Exposure to transgressions by others and betrayal based morally injurious events were significantly lower in the armed forces than in civilian occupations. The pooled prevalence of PTED across occupations was 26%. A narrative synthesis identified that studies measured the association

For peer review only

between moral injury and PTED with demographic, (mental) health, and individual/work variables.

Conclusions

Exposure to morally injurious events, moral injury symptoms, and PTED are prevalent across work settings and exposure to transgressions by others and betrayal events are more likely in civilian occupations.

Strengths and limitations:

- This represents the first pooled prevalence and norm estimates of morally injurious events, moral injury symptoms and PTED using meta-analysis methods
- Findings show civilian occupations are at greater risk of exposure to morally injurious events which can inform policy
- The meta-analysis highlights important limitations in the literature which cause high heterogeneity across studies
- Only peer-reviewed articles written in English were included which may lead to publication bias

For peer review only

Introduction:

Exposure to traumatic events at work is commonplace, and meta-analyses have identified occupational groups that are at a higher risk of developing work-related PTSD.[1, 2] The prevalence of occupational PTSD varies greatly among studies (i.e., 8.4-41.4%), and is related to traumatic events such as being bullied, handling corpses, and accidents at work.[2] However, many individuals also experience morally traumatic events at work, through interpersonal conflicts, making challenging ethical decisions, and exposure to events that contradict what is 'right'.[3-5] Two concepts have emerged in the literature to explain the harm caused by exposure to unjust and morally injurious events (MIEs), and these are called moral injury and PTED.

When an individual experiences events that they perceive to be unjust, they may respond with feelings of embitterment, helplessness, and hostility. In 2003, Linden[6] introduced PTED as a potential diagnosis when the intensity and duration of embitterment reactions increase and become associated with significant impairment. Research has shown that PTED often occurs at work, for instance, 73% of PTED patients reported the triggering event as work-related[7], and research has shown PTED is evident in specific occupations such as the armed forces[8, 9], educators[10, 11], healthcare professionals[12-14], the unemployed[15, 16], and in a mix of occupations[17, 18].

PTED shares similarities in aetiology and symptomology with moral injury[14, 19], however, moral injury originated specifically to explain military experiences. Moral injury was introduced in the 1990s to describe the psychological suffering of Vietnam veterans following betrayal by leaders.[5] Scientific research on moral injury began later, in 2009 with the publication of a comprehensive definition by Litz et al.,[4] which encompasses harm caused by perpetrating, witnessing, or learning about acts that violate moral beliefs. The moral injury events scale (MIES) was the first designed to specifically measure moral injury

For peer review only - http://bmjopen.bmj.com/site/about/guidelines.xhtml

and accounted for both definitions (i.e., transgressions by self, by others, and betrayal).[20] The scale, like most of the subsequent moral injury scales, is military-specific, (i.e., see [21] for a review) and so unlike PTED, moral injury research has typically been dominated by studies in the armed forces. However, prior to the COVID-19 pandemic, a meta-analysis showed that occupational moral injury is also evident in civilian occupations such as journalists, veterinarians, the police, and teachers[22], and since the outbreak of the pandemic, there has been a proliferation of interest in moral injury in health and social care.

Despite this, there is no clear evidence for how widespread occupational moral injury and PTED are. Several inconsistencies in reporting make comparison across studies difficult. First, moral injury and PTED are stressor-related conditions and exposure to one or more negative life events triggers the symptoms. Aside from the PTED diagnostic interview[23], there is only one self-rating scale that measures reactive embitterment, and participants respond regarding the event(s) which are specified in the instructions.[3] In contrast, there are several scales designed to assess different aspects of moral injury, and a review by Koenig, Pearce, and Youssef[21] found that there were 17 scales measuring only one or two aspects of moral injury, and so arguably do not measure the construct as a whole. Of the 5 comprehensive measures designed to assess multiple dimensions of moral injury, some only measure symptoms, whereas others conflate exposure to MIEs with symptoms. The second inconsistency relates to using established cut-off values to estimate prevalence. Since PTED is considered a psychiatric disorder, three cut-off values are used to indicate clinically relevant embitterment at three different levels of severity[3] On the other hand, there is disagreement among researchers as to whether moral injury is a clinical disorder, or simply a risk factor for other disorders and so only some moral injury scales use established cut-off values. Other researchers simply report norms for the scales.

For peer review only - http://bmjopen.bmj.com/site/about/guidelines.xhtml

Since research shows that certain occupations have a higher risk of developing work-related PTSD, especially in professionals that do not have special training to deal with traumatic events[1], it is possible that certain occupations are also at a higher risk of developing occupational moral injury and PTED following ethically challenging events. However, as far as we are aware, no meta-analyses exist which combine prevalence estimates, or establish whether certain occupations are more at risk, and current attempts to combine the prevalence of transgressive acts[24] and moral injury symptoms[25] rely on a narrative synthesis of military studies. Furthermore, whilst moral injury and PTED share many similarities, they are not often studied together, and it is useful to investigate whether they share associations with each other and similar variables.

The current systematic review and meta-analyses aim to; (i) determine the prevalence of occupational moral injury and PTED, (ii) establish whether prevalence estimates differ depending on occupation, (iii) establish whether methodological differences between studies influence prevalence estimates, and (iv) record the variables associated with occupational moral injury and PTED.

For peer review only

Methods:

Guidelines from the Preferred Reporting Items for Systematic Reviews and Meta-Analyses Protocols (PRISMA)[26] were used to prepare this review. The Joanna Briggs Institute (JBI) methodology for prevalence and incidence studies[27] was used to develop the eligibility criteria (Table 1), a search strategy (Supplementary Table 1), data extraction (Supplementary Appendix A) and risk of bias assessment (Supplementary Table 2). This review is registered with the international prospective register of systematic reviews PROSPERO network (registration no. CRD42020191766).

Eligibility criteria:

The "CoCoPop" mnemonic[27] was used to determine inclusion and exclusion criteria and are outlined in Table 1. Studies were included if they measured the prevalence and/or norms of PTED and/or moral injury using the PTED diagnostic interview,[23] PTED self-rating scale,[3] or a comprehensive and standardised measure of moral injury. To be included in the review, studies had to measure moral injury as a whole construct rather than measuring the separate theoretical elements, such as guilt and self-forgiveness (i.e., see [21] for a review). PTED and moral injury must have been measured in an occupational group, in relation to work, or in unemployed or retired samples. Any working age, sex, ethnicity, country, educational status, and socio-demographic status was included. Secondary outcomes of interest were univariate associates of PTED and/or moral injury. The review included observational studies (i.e., cross-sectional, retrospective, prospective, cohort and longitudinal) and excluded intervention studies or reviews. Where multiple papers were identified using the same data, the most relevant paper was included.

Search strategy:

A computer literature search was run in June 2020 using seven databases, Google Scholar, PubMed, PsycINFO, Web of Science, Scopus, ScienceDirect and Sage Journals

For peer review only

online. Articles related to PTED were included from 2003, which is when the first conceptual definition for PTED was developed[6] and articles related to moral injury were included from 2009 in line with the first comprehensive scientific definition of moral injury.[4] Search terms were used as free text terms and combined with Boolean operators. The search was restricted to peer-reviewed articles written in English. References were managed in EndNote (Clarivate Analytics, Philadelphia) and Excel. The searches were updated in November 2022.

Data extraction

One researcher (CB) screened all titles, abstracts, and full texts and a second reviewer (CR) screened 15% of the titles and abstracts and 100% of the full texts. Any discrepancies over the inclusion of studies were resolved through discussion. At every stage of screening, inter-rater agreement was >90%, with Cohen's kappa >0.90, indicating almost perfect agreement.[28]

The following data was extracted; author (year), study design, number of participants, mean age, the proportion of males, proportion of white ethnicity, socio-economic status, geographical location, occupation, length of time in the job, sampling method including setting and dates, the measurement tool for PTED and/or moral injury, prevalence (N and proportion), norms (Mean and SD), and any conflict of interest. Secondary outcomes included univariate associates of PTED and moral injury including the variable name, the measure used, and the direction of effect (positive, negative or no association). For longitudinal and cohort studies, prevalence estimates or norms from the baseline wave were extracted unless only subsequent waves were reported. CB contacted the authors to obtain relevant information that was not available in the text. One researcher (CB) completed the data extraction, and a second reviewer (CR) screened 100% of the data extraction to check for accuracy.

Quality assessment

For peer review only

Quality assessment was conducted using the Quality Assessment Checklist for Prevalence Studies created by Hoy et al.,[29]. This tool assesses the possibility of bias in design, conduct and analysis including sample representativeness, sampling, non-response bias, data collection method, appropriateness of case definition, study instrument, and statistical analysis. Each item is coded as 0 (yes, low risk), or 1 (no, high risk) and quality assessment scores were summed, and categorised into low (0-3), moderate (4-6) and high (7-9) risk. One researcher (CB) conducted the quality assessment (see Supplementary Table 2).

Data analysis

We conducted meta-analyses using R (4.2.1) with the Meta package. All meta-analyses used a random effects model using the restricted maximum-likelihood estimator (REML) due to the high heterogeneity associated with observational studies.[30] Separate meta-analyses were conducted with the prevalence of PTED as the outcome, and then the prevalence of moral injury as the outcome. For PTED prevalence, three separate analyses were conducted based on a cut-off ≥1.6, then 2 and then 2.5 which represents different levels of clinical severity.[3] For moral injury, due to the heterogeneity in moral injury measures (i.e., see Table 2 for an overview), separate meta-analyses were conducted for each moral injury measure and their associated subscales. Some moral injury scales do not use cut-off values to estimate the prevalence and only report norms, so additional meta-analyses were conducted with the mean of PTED and mean of moral injury scores as the outcome.

Subgroup analyses were conducted if there were sufficient studies of k ≥10[31], grouping studies based on occupation. Forest plots were generated, displaying the prevalence and 95% CIs for each estimate, the overall pooled estimate, and 95% CIs for each occupational group. Additional subgroup analyses were conducted based on study quality (low or medium risk) and on cut-off value used for moral injury prevalence meta-analyses. If a study reported the prevalence of moral injury using multiple cut-off scores in the same

For peer review only - http://bmjopen.bmj.com/site/about/guidelines.xhtml

paper, then the most common across studies was chosen for inclusion. A random effect model with a common estimate for τ^2 was used in subgroup analyses due to $k \leq 5$ in some of the subgroups.[32] In order to determine whether any of the subgroups were statistically different, a Q-test with $p<0.05$ was used based on the overall subgroup results. No overlap in 95% CIs was used to determine which groups were significantly different from one another.[33]

For meta-analyses that used prevalence estimates as the outcome, the Freeman-Tukey double arcsine transformation method was applied, which is effective when there are small sample sizes and extreme prevalence estimates.[34, 35] Heterogeneity was assessed using I^2 with a cut-off of >50% suggesting high heterogeneity.[36] Significance was assessed using x^2 for Cochrane's Q ($p<0.05$). Funnel plots are not suitable to assess publication bias in meta-analyses of proportions.[37] Therefore, a sensitivity analysis was used, comparing prevalence and norm estimates when outliers were removed, which were extremely small or large values that fell outside of the CI of the pooled effect.[38]

Finally, where prevalence or norms could not be pooled due to inconsistent reporting or insufficient studies (k=1), narrative synthesis was used to describe estimates. The narrative synthesis also describes the univariate associates of PTED and moral injury using the Guidance for Systematic Reviews.[39] Associates were grouped into demographic, (mental) health and individual/work variables, and the percentage of studies that reported each type of variable was calculated. Vote counting was used to determine whether the overall relationship between variables with PTED and moral injury was positive, negative or no association.

Patient and public involvement

As a systematic review focused on published literature no primary research involving patients was conducted.

For peer review only - http://bmjopen.bmj.com/site/about/guidelines.xhtml

Table 1.
Inclusion and exclusion criteria

**Inclusion criteria**	
Condition (outcome of interest)	• Studies measuring the prevalence or norms of PTED, including chronic embitterment.<br>• Studies measuring prevalence or norms of moral injury.<br>• Uses PTED diagnostic interview, PTED self-rating scale, or a comprehensive moral injury measure.
Context	• All geographical locations.
Population	• PTED and/or moral injury must have been measured in relation to occupation including studies reporting on multiple occupations, the unemployed or retired.<br>• 18+, any sex, ethnicity, educational status, or socio-economic status.
**Exclusion criteria**	
Condition	• Studies using the Bern Embitterment Inventory (BEI) to measure PTED.<br>• Studies which only measured theoretical subcomponents or limited dimensional scales of moral injury.
Context	• Clinical patients in treatment or intervention studies.
Population	• Student only samples.<br>• Entirely clinical samples or all exposed to a specific stressor i.e., bullied at work, all exposed to a moral transgression, all injured at work etc.

PTED; post-traumatic embitterment disorder

For peer review only

Results:

Study characteristics:

The PRISMA flow chart delineates the review process (see Figure 1). Of the 81 full texts included in the review[8, 10, 12-18, 41-111], some reported multiple studies in the same paper so there were 88 separate studies included in the review. Of these, six reported prevalence estimates and/or norms that could not be pooled and so were included in the narrative synthesis only. Of the 88 studies included, 10 measured PTED, one measured both PTED and moral injury, and 77 measured moral injury. The number of studies included in each separate meta-analysis based on the measure used ranged from 2 to 30.

The characteristics of included studies are in Supplementary Appendix A and the risk of bias information is in Supplementary Table 2. Of the 88 studies, 9 had a low risk of bias (10%) and all others were medium risk, 77 (87.5%) were cross-sectional, and sample sizes ranged from 38 to 14,057. Despite a small number of PTED studies, there was a varied geographical distribution, with 3 studies being conducted in the UK and one study each

For peer review only - http://bmjopen.bmj.com/site/about/guidelines.xhtml

conducted in Germany, Turkey, Bosnia and Herzegovina, Cyprus, Pakistan, and South Korea. Occupations also varied with samples including healthcare (n = 4), education (n = 2), mixed occupations (n = 2), the unemployed (n = 2), veterans (n = 1) and the public sector (n = 1). In contrast, most moral injury studies were conducted in the US (n = 41), and in military samples (n = 39). HSCWs were the second biggest occupational group (n = 32), and other occupations were less represented including first responders (n = 2), educators (n = 2), journalists (n = 2), and CPS employees (n = 1). Geographically, aside from the US, studies were conducted in the UK (n = 6), Canada (n = 5), Israel (n = 4), Australia (n = 3), Republic of Ireland (n = 2), China (n = 2), and other countries with only one study included El Salvador, Korea, Finland, Pakistan, Romania, Iran, Honduras, Italy, Croatia, German-speaking populations, and Turkey.

Table 2 shows the characteristics of the PTED and moral injury measurement scales included in the review. All studies measuring PTED used the PTED-self rating scale, in contrast to studies measuring moral injury which included 11 distinct scales, 5 of which conflated MIEs with symptoms in the same items, 2 scales assessed both MIEs and symptoms but separately, and 4 assessed moral injury symptoms only. The most common moral injury scale was the MIES (n = 38) which conflates events and symptoms, followed by the moral injury symptom scale for healthcare professionals (MISS-HP) which assesses clinically relevant symptoms of moral injury in healthcare professionals (n = 12). Most moral injury scales are occupation-specific, and there was variation in reporting regarding the number of items, scoring, the cut-off value used, and subscale factor structure endorsed across the moral injury scales.

For peer review only - http://bmjopen.bmj.com/site/about/guidelines.xhtml

Table 2

PTED and moral injury measurement scales included in the systematic review

Measure	*k*	Events or symptoms	Decision regarding classification	Subscales	Cut off value	Scale information
PTED self-rating scale[3]	11	Symptoms linked to event	Assess embitterment reactions to event(s) specified in instructions	Unidimensional	≥1.6, ≥2, ≥2.5	19 items (0-4)
MIES[20]	38	Combined	Assesses exposure to MIEs and effects (i.e., feeling troubled by those acts).	Bryan et al.,[41] 3 factors: transgressions-others, transgressions-self, and betrayal. Nash et al.,[20] 2 factors: perpetration and betrayal. Richardson et al.,[55] 2 factors: MI-self and MI-other. Lamb et al., 2021 3 factors: commission, omission and betrayal.	4+ (slightly agree) 5+ (moderately agree) 6+ (strongly agree)$_b$	9 items (1-6 Likert)
MIQ-M[42]	9	Combined	Assesses military specific MIEs, and effects.	Primarily unidimensional.<br><br>Lancaster and Harris[63] 2 factors: MIQ-causes and MIQ-effect	None	19 items (1-4 Likert)
MIQ-T[43]	1	Combined	Assesses teacher specific and effects	Unidimensional	None	12 items (1-4 Likert)
MIQ-M modified[62]	1	Events and symptoms	Modified MIE items from the original MIQ-M which conflated MIEs and effects into MIEs and defining characteristics	3 MIEs subscales AoW, PCoW and LFB and 3 subscales for their defining characteristics	2+ (seldom) on each item	22 MIEs items and 5 defining characteristics items per MIE (1-4 Likert)
EMIS-M[44]	7	Symptoms	Assesses guilt, shame, moral concerns, self-condemnation, social withdrawal, and inability to forgive self, anger, feelings of betrayal, revenge, and disgust.	2 subscales: self-directed and other-directed	None	17 items (1-5 Likert)
EMIS-SF[111]	1	Symptoms	Assesses emotions (guilt and shame) and beliefs/attitudes (moral disgust and mistrust).	Unidimensional	None	4 items (1-5 Likert)

For peer review only - http://bmjopen.bmj.com/site/about/guidelines.xhtml

MISS-HP[91]	12	Symptoms	Assesses betrayal, guilt, shame, moral concerns, loss of trust, loss of meaning, difficulty forgiving, self-condemnation, religious struggle, and loss of religious/spiritual faith.	Unidimensional	>36[a]	10 items (1-10 visual analogue Likert)
MIOS[87]	4	Symptoms linked to MIE	The symptom scale refers to the most distressing MIE. Symptoms include self-blame, loss of faith, trust, worthiness, goodness in others, honesty, pride, self-hatred, disgust and anger.	2 subscales: Shame-related (SR) and trust violation-related (TVR)	None[c]	14 items (0-4)
BMIS[95]	1	Events and symptoms	Endorsement of at least one MIE assessed needed to complete symptom questions	2 subscales: BMIS-event (BMIS-E) and BMIS-sequalae (BMIS-S)	2+ MIE Symptom subscale: None	7 items (0-3).
MIA-PSP[98]	1	Combined	Assess feeling "bothered" by PSP specific MIEs, and symptoms (i.e., feelings of sadness, anger, self-hatred, guilt, shame, disgust, and fear).	3 subscales: perpetration, betrayal and emotional Sequalae	None	17 items (1-6)
Toronto scale for journalists[96]	1	Combined	Assesses journalism specific MIEs and effects (i.e., feeling troubled by those MIEs).	3 subscales: organisational/management; individuals/Non-management, and online	None	9 items (0-4)

Note: The number of items and scoring is based on the typical use of the scale.[a]The German MISS-HP (G-MISS-HP) uses a 9 item scale with a cut off >28.5[105],The Persian MISS-HP uses a cut off >36.5[90] [b]Some papers modified the Likert scale and used a cut off of 3+ "sometimes" and 4+ "often[50] or 3+ "agree"[67, 88] [c]MIE questions precede the MIOS symptoms scale and can be quantified as endorsed (yes/no). PTED; post-traumatic embitterment disorder, MIES; moral injury events scale; EMIS-M; Expressions of Moral Injury Scale-Military Version; MISS-HP; Moral Injury Symptoms Scale – Health Professionals, MIA-PSP; Moral Injury Assessment for Public Safety Personnel, MIQ-M; Moral Injury Questionnaire—Military version, BMIS; Brief Moral Injury Screen; EMIS-SF; Expressions of Moral Injury Scale-Short Form, MIQ-T; Moral Injury Questionnaire—Teacher version, AoW; Atrocities of War, PCoW; Psychological consequences of War, LFB; Leadership Failure or Betrayal, MIOS; Moral Injury Outcome Scale

For peer review only

Prevalence meta-analyses:

Supplementary Figures 1-8 show the forest plots with pooled prevalence estimates for PTED moral injury, split by occupation where possible (k>10).

PTED

The pooled prevalence of PTED when using a cut-off of ≥1.6 was 37% [95% CI 24.4% to 49.9%], when using a cut-off of ≥2 was 26% [95% CI 16.1% to 37.4%], and when using a cut-off of ≥2.5 was 11% [95% CI 4% to 21.3%]. No subgroup analyses were conducted due to k≤10.

Moral injury

Using the MISS-HP, the pooled prevalence of clinically relevant signs of moral injury in healthcare professionals was 45% [95% CI 36.4% to 53.5%]. Across occupations, the pooled prevalence of exposure to any MIE on the MIES scale was 67% [95% CI 59% to 74.5%], exposure to perpetration based MIEs was 20% [95% CI 1.1% to 53.7%], transgressions by self MIEs was 31% [95% CI 24.7% to 37.5%], transgressions by others MIEs was 48% [95% CI 38% to 57.5%], and betrayal based MIEs was 46% [39.5% to 52.6%]. Whilst not significantly different, exposure to any MIE was lowest in first responders (58%, 95% CI 30.9% to 82.5%) followed by the armed forces (59%, 95% CI 45.9% to 72.4%), healthcare professionals (71%, 95% CI 60.7% to 81%) and education professionals (88%, 95% CI 82.5% to 91.7%). Furthermore, whilst not significantly different, exposure to transgressions by self were also lowest in first responders (21%, 95% CI 16.1% to 26.8%), followed by CPS professionals (24%, 95% CI 11.4% to 40.2%), the armed forces (29%, 95% CI 19.9% to 39.6%), healthcare professionals (33%, 95% CI 23.4% to 43.9%) and education professionals (43%, 95% CI 36% to 49.5%).

There were significant differences in the prevalence of exposure to transgressions by others, and betrayal based MIEs based on occupation (*p*'s<0.01). Lack of overlap in CI shows

For peer review only - http://bmjopen.bmj.com/site/about/guidelines.xhtml

the armed forces were exposed to significantly fewer transgressions by others (31%, 95% CI 20.1 % to 41.8%) compared to healthcare professionals (55%, 95% CI 43.9% to 65.6%), first responders (61%, 95% CI 54.3% to 66.9%), education professions (80%, 95% CI 74.4% to 85.3%) and CPS professionals (92%, 95% CI 78.62%–98.34%) though first responders, education and CPS professionals included only one study each. Furthermore, the armed forces were exposed to significantly fewer betrayal events (33%, 95% CI 26.3% to 39.4%) compared to healthcare workers (58%, 95% CI 51% to 65.1%) and education professionals (68%, 95% CI 61.3% to 74%). Whilst not significantly different, first responders had higher estimates than the armed forces (52%, 95% CI 35.2% to 69.1%), and those in the CPS had the lowest exposure to betrayal but this was based on only one study (32%, 95% CI 17.5% to 48.7%).

Norms meta-analyses:

Supplementary Figures 9-21 show the forest plots with pooled mean estimates for PTED and moral injury, split by occupation where possible (k>10).

PTED

The pooled mean for the PTED scale across occupations was 1.52, and this was not significantly different according to occupation due to wide confidence intervals.

Moral injury

Since most moral injury scales only report norms and not prevalence estimates, pooled mean estimates for the MIQ-M, EMIS, MIOS, MISS-HP and MIES were conducted. Only the pooled mean for transgressions by others on the MIES (M=3.16) significantly differed across occupations ($p<0.01$), with significantly lower scores in the armed forces (2.66, 95% CI 2.23 to 3.09) compared to first responders, education professionals, and the CPS.

For peer review only

Sensitivity analyses: outliers removed

Table 3 shows the pooled prevalence and mean estimates, before and after removing outliers for PTED and moral injury.

Prevalence

Pooled prevalence estimates for PTED decreased when outliers were removed, using all three cut-offs. Prevalence was reduced to 31% (95% CI 23.9% to 39.2%, $I^2 = 84\%$) when using a cut off ≥1.6, reduced to 21% (95% CI 16.40% to 26.4%, $I^2 = 75\%$) when using a cut off ≥2 and decreased to 8% (95% CI 6.4% to 10.1%, $I^2 = 62\%$) when using a cut off ≥2.5.

The prevalence of moral injury on the MISS-HP remained similar (i.e., 43%, 95% CI 39.3 % to 47.41%, $I^2 = 85\%$), and the prevalence of exposure to transgressions by self MIEs decreased to 23% (95% CI 21.6% to 24.3%, $I^2 = 4\%$). All other types of MIEs increased, i.e., exposure to any MIE increased to 73% (95% CI 70.2% to 76.4%, $I^2 = 76\%$), transgressions by others increased to 49% (95% CI 43.8% to 54.59%, $I^2 = 93\%$), and betrayal stayed relatively similar at 46% (95% CI 41% to 51.3%, $I^2 = 93\%$). Except for exposure to transgressions by self, heterogeneity remained high even after removing outliers (≥50%).

Norms

Pooled mean estimates decreased for six of the moral injury scales and/or subscales after removing outliers and increased for six of the scales (see Table 3). Except for the MIOS trust subscale, heterogeneity remained high even after removing outliers (≥50%).

Subgroup analyses: study quality and cut-off value

Additional subgroup analyses were conducted based on the risk of bias where possible (k≥10; Supplementary Table 3). For prevalence meta-analyses, exposure to any MIE was significantly lower in low-risk studies (47 %, 95% CI 32.42 to 61.92, k=5) compared to high-risk (72.30%, 95% CI 64.83-79.20, k=18, p=0.003). Furthermore, exposure to transgressions

For peer review only - http://bmjopen.bmj.com/site/about/guidelines.xhtml

by self was significantly lower in low-risk studies (16%, 95% CI 84.7% to 25.2%, *k*=4) compared to high-risk (35%, 95% CI 28.3% to 41.8%, *k*=22, *p*=0.006), and exposure to betrayal MIEs was significantly lower in low-risk studies (30%, 95% CI 18.5% to 43.7%, *k*=5) compared to medium-risk studies (50%, 95% CI 43.1% to 56.3%, *k*=23, p=0.011). The pooled prevalence estimates transgressions by others were not significantly different based on the risk of bias (*p*=0.254). When comparing pooled mean estimates based on the risk of bias, only the pooled mean for betrayal was significantly lower in low-risk studies (2.03, 95% CI 1.33 to 2.73, *k*=3) compared to high risk (2.83, 95% CI 2.60 to 3.06, *k*=27, p=0.034). For the prevalence meta-analyses, different cut-off values were used to signify moral injury on the MIES and the MISS-HP (see Table 2). Therefore, additional subgroup analyses based on the cut-off value were conducted (see Supplementary Table 4) and found no significant differences in pooled prevalence estimates depending on the cut-off value (p's>0.05).

Table 3.
Pooled prevalence and mean estimates for PTED and moral injury, with and without outliers

Measure	With outliers			Without outliers		
	k	Pooled % (95% CI)	I^2	*k*	Pooled % (95% CI)	I^2
PTED 1.6	9	36.65 (24.38 to 49.85)	98%	6	31.27 (23.87 to 39.17)	84%
PTED 2	8	26.03 (16.09 to 37.38)	97%	7	21.21 (16.40 to 26.44)	75%
PTED 2.5	6	22.31 (4.02 to 21.32)	98%	7	8.16 (6.37 to 10.14)	62%
MIES any	23	67 (59.01 to 75.54)	98%	11	73.36 (70.18 to 76.42)	76%
MIES transgressions-self	27	30.95 (24.72 to 37.54)	99%	11	22.99 (21.62 to 24.32)	4%
MIES transgressions-others	25	47.73 (38.04 to 57.51)	98%	14	49.20 (43.82 to 54.59)	93%
MIES betrayal	28	46.03 (39.54 to 52.59)	98%	11	46.12 (40.97 to 51.32)	91%
MIES perpetration	2	20.26 (1.11 to 52.70)	98%		No outliers	
MISS-HP	10	44.88 (36.40 to 53.50)	98%	7	43.33 (39.30 to 47.41)	85%

For peer review only - http://bmjopen.bmj.com/site/about/guidelines.xhtml

	k	Pooled mean (95% CI)	I^2	*k*	Pooled mean (95% CI)	I^2
PTED	11	1.52 (1.10 to 1.93)	99%	7	1.33 (1.18 to 1.49)	97%
MIES total	29	2.58 (2.35 to 2.82)	99%	16	2.61 (2.52 to 2.70)	86%
MIES transgressions-self	27	2.26 (1.99 to 2.52)	99%	12	2.16 (2.95 to 2.28)	77%
MIES transgressions-others	24	3.16 (2.81 to 3.52)	99%	10	3.05 (2.89 to 3.20)	83%
MIES betrayal	30	2.74 (2.51 to 2.98)	99%	15	2.77 (2.67 to 2.86)	80%
MIES perpetration	5	2.55 (2.09 to 3.02)	99%	4	2.77 (2.60 to 2.95)	83%
MIQ-M total	5	1.77 (1.58 to 1.96)	97%	4	1.84 (1.66 to 2.02)	91%
MIQ-M causes	4	1.42 (1.21 to 1.60)	96%	3	1.31 (1.26 to 1.36)	75%
EMIS total	4	2.22 (1.90 to 2.54)	99%		No outliers	
EMIS self	4	1.96 (1.68 to 2.24)	92%		No outliers	
EMIS others	5	2.51 (2.28 to 2.74)	93%	4	2.61 (2.46 to 2.76)	55%
MISS-HP	12	36.73 (33.39 to 40.07)	99%	7	35.42 (34.14 to 39.87)	76%
MIOS total	4	27.75 (24.19 to 31.31)	95%	3	25.88 (24.45 to 27.20)	66%
MIOS shame	4	12.77 (10.43 to 15.12)	98%	3	11.42 (11.01 to 11.83)	0%
MIOS trust	4	14.97 (13.65 to 16.28)	94%		No outliers	

PTED; post-traumatic embitterment disorder, MIES; moral injury events scale; EMIS-M; Expressions of Moral Injury Scale-Military Version; MISS-HP; Moral Injury Symptoms Scale – Health Professionals, MIQ-M; Moral Injury Questionnaire—Military version, MIOS; Moral Injury Outcome Scale

For peer review only

<u>Narrative synthesis:</u>

<u>Prevalence</u>

Supplementary Table 5 shows the prevalence and norms for moral injury scales that could not be combined (*k*=1) including different variations of MIES subscales i.e., (MI-other; 20%, MI-commission; 14%, MI-omission; 5%), or moral injury profiles based on the MIES (no moral distress; 42%, moral distress-other; 19%, witnessing-only; 16%, moral distress-self; 8%, moral distress-self and other; 15%). Variations of the MIQ-M include the teacher

For peer review only - http://bmjopen.bmj.com/site/about/guidelines.xhtml

version (MIQ-T; M=1.16), or the modified version. Other moral injury scales were only included in one study each, such as the PSP (M=59.6), or the Toronto moral injury scale for journalists (M=1.5). See supplementary Table 5 for specific norms and/or prevalence estimates for the associated subscales.

<u>*Univariate associates of PTED and moral injury*</u>

A narrative review was used to synthesise the associates of PTED and moral injury due to the heterogeneity of variables measured, with the exception of (mental) health variables which were frequently measured in moral injury studies, however, meta-analyses of this relationship have been conducted recently.[21, 113, 114]

Demographic variables:

Of the PTED studies, six (55%) measured demographic variables. Overall, there was no association with gender, a positive association with length of time in the job, and a mixed association with age, with 2 papers finding a positive association, and 2 papers finding no association. Many variables were measured less frequently across studies (i.e., k<3), such as ethnicity, professional role, hours worked, having dependents, marital status, income, and education level. Of the moral injury studies, 31 (40%) measured demographic variables. Overall, there was no association with gender, age, length of time in the job, marital status, ethnicity/race/country of birth, education, and income. Several variables were measured less frequently (i.e., k<3), such as being unemployed and having dependents, and several papers measured variables specific to the work environment such as professional role, though varied job roles preclude comparison.

(Mental) health variables:

For peer review only - http://bmjopen.bmj.com/site/about/guidelines.xhtml

Of the PTED studies, seven (64%) measured (mental) health variables and found positive associations with depression, stress, anxiety, existing mental health diagnoses, psychological distress, negative affect and a negative association with positive affect and employee well-being though none of these variables were measured frequently (k<3). Of the moral injury studies, 51 (65%) measured (mental) health variables and found positive associations with depression, PTSD and/or PTSD subscales, guilt and/or shame, anger, anxiety, burnout and/or its subscales, drug and/or alcohol use, and a negative association to well-being. Other variables related to (mental) health appeared less frequently (i.e., k<3).

Individual/work characteristics:

Of the PTED studies, 10 (91%) measured individual/work characteristics. Justice beliefs were negatively associated with PTED. Other variables were measured less frequently (k<3) such as rumination, unemployment duration and appraisal, supervisory control, job demands, work attitudes, social support, self-efficacy, leadership behaviours, and personality traits such as optimism, self-esteem, and resilience, though generally PTED was associated with undesirable work factors. Of the moral injury studies, 40 (51%) measured individual/work characteristics. The most common positive association was with combat exposure. Religiosity was negatively associated with moral injury, although two papers found a positive association, turnover intentions were positively associated with moral injury, and resilience associations were mixed, showing positive, negative, and no associations with moral injury. COVID-related factors were measured across several studies, but variations in the type of COVID variables measured preclude comparison. Many variables related to personality variables, beliefs, and specific work environmental factors were measured less frequently (k<3).

For peer review only - http://bmjopen.bmj.com/site/about/guidelines.xhtml

Discussion

This is the first meta-analysis of the prevalence and norms of occupational moral injury and PTED, with results showing that across occupations, 37% of staff display embitterment of clinical relevance (≥1.6), 26% showed more practically relevant levels (≥2), and 11% showed severe clinical levels of PTED (≥2.5). Findings also showed that across occupations, 67% were exposed to at least one MIE, 20% were exposed to a perpetration based MIE, 31% committed a moral transgression, 48% witnessed a transgression by others, and 46% were exposed to betrayal MIEs at work. Exposure to transgressions by others and betrayal were significantly lower in the armed forces compared with healthcare workers and education professionals. Finally, clinical levels of moral injury in healthcare professionals according to the MISS-HP was 45%. Since several moral injury scales did not provide prevalence estimates, pooled norms were also calculated, and the mean scores for PTED did not differ according to occupation. Mean scores of exposure to transgressions-by others were significantly lower in the armed forces compared to first responders, the CPS, and education professionals. Overall, prevalence estimates for exposure to any MIE, transgressions by self and betrayals were lower in low risk compared to medium-risk studies, and the mean scores for betrayal was also lower in low-risk studies. Furthermore, prevalence estimates for moral injury were not significantly different based on the cut-off value used by the researcher.

The narrative review identified variables measured in association with moral injury and PTED fell into three categories: demographics, (mental) health and individual/work characteristics. Gender was found to have no association with moral injury and PTED, whereas the length of time in the job had a positive association, and age had a positive or no association with PTED. In contrast, age, length of time in the job, marital status, ethnicity/race/country of birth, education and income all had no association with moral injury. Other demographic variables varied and were usually only included in one or two

For peer review only - http://bmjopen.bmj.com/site/about/guidelines.xhtml

For peer review only

studies. Moral injury and PTED showed a positive association with (mental) health variables including depression, stress, anxiety, and employee well-being, and moral injury studies studied the relationship with a wider range of disorders such as PTSD and anxiety, and emotional (i.e., guilt, anger, shame), behavioural (i.e., burnout, compassion fatigue) and spiritual variables. Finally, individual/work variables differed between the two constructs, with moral injury studies focusing primarily on the combat environment as a risk for military samples and PTED focusing on justice beliefs as protective in civilian samples. Furthermore, religiosity and resilience were measured in relation to moral injury with mixed results, and turnover intentions were positively associated with moral injury. Individual risk and resiliency factors such as optimism, self-esteem and personality traits were also identified, but less frequently.

The results of this study should be considered in light of several strengths and limitations. The study protocol was pre-registered on Prospero and followed the Joanna Briggs Institute guidance for systematic reviews of prevalence and incidence data which ensures the methods were robust.[28] However, despite using robust methods, there was high heterogeneity amongst studies, even after employing subgroup analyses to explain the variability based on study quality, the cut-off score used and sensitivity analyses to remove outliers. Whilst heterogeneity is often high in prevalence meta-analyses, and the median I^2 was found to be 96.9% in a review of 134 prevalence meta-analyses[114], caution must be taken when interpreting our findings. Furthermore, only 10% of studies had a low risk of bias, and often the primary aim of the included studies was not to produce prevalence data. This may explain the use of inappropriate statistical methods (i.e., lack of confidence intervals or weighting), and variability in reporting only norms or prevalence data based on the various subscale configurations or using different cut-off values. To deal with this heterogeneity, separate meta-analyses were conducted based on the type of data reported (i.e.,

For peer review only - http://bmjopen.bmj.com/site/about/guidelines.xhtml

prevalence or norms), and the measure used, reducing the number of studies included in each meta-analysis and thus power, meaning that subgroup analyses based on occupation were not always possible. Finally, whilst we searched a relatively high number of databases, it is possible that references from the grey literature have been missed.

Despite the limitations, this review adds to the growing literature on the prevalence of moral injury and PTED. Prior estimates of PTED in the general population were at 2.5%[3] and this compares to 26% across occupations in this review. This confirms the findings by Linden et al.,[7] which found that 73% of PTED patients reported the triggering event as work-related and shows unjust and embittering events often occur in the workplace. Using the most stringent cut-off, occupational health services could expect 11% of their staff to display clinically severe levels of PTED according to these findings. There were not enough studies to conduct subgroup analyses based on the prevalence of PTED across occupations, though subgroup analyses using the mean PTED scores showed no significant difference based on occupation. This might suggest that PTED is widespread across professions. However, this analysis was based on only 11 studies, and the inclusion of more studies would be needed to confirm that this effect is not due to low statistical power.

Previous attempts to collate prevalence estimates for moral injury have relied on a narrative synthesis of the prevalence of transgressive acts[24] or moral injury symptoms in military samples.[25] Findings from this meta-analysis shows that 67% of people across occupations have been exposed to at least one MIE in their jobs, which is a high proportion. Furthermore, staff were more often exposed to transgressions by others (48%) and betrayal MIEs (46%) than MIEs perpetrated by themselves (31%) which mirrors qualitative findings during the pandemic, which shows healthcare staff experienced institutional betrayal more than perpetration based moral injuries.[115, 116] Estimates of exposure to MIEs are taken from the MIES,[20] which was the most widely used scale to measure moral injury across

For peer review only - http://bmjopen.bmj.com/site/about/guidelines.xhtml

studies in this review. Whilst it is arguable that not all individuals exposed to MIEs will experience moral injury symptoms, the MIES does confound events and effects by asking participants if they feel troubled by their exposure to moral violations.[21] This suggests that a high proportion of staff are not only exposed to events that are morally injurious, but are also affected by this. This is supported by further findings from this meta-analysis which showed that 45% of healthcare workers had clinically relevant signs of moral injury according to the MISS-HP which is the only moral injury symptom scale which uses a clinical cut off.[91] Since the MISS-HP is healthcare specific, and the other symptom scales in this review only reported mean scores and not prevalence estimates, it is not possible to determine whether any occupations are at a greater risk of clinically relevant moral injury.

However, the review was able to show that members of the armed forces were exposed to significantly fewer transgressions by others and betrayal MIEs than healthcare workers and education professionals, and the mean scores for transgressions by others were significantly lower in the armed forces than first responders, the CPS, and education professionals. Therefore, despite the definitions of moral injury originating from the military [4, 5], this shows that civilian occupations are at an even greater risk of exposure to MIEs and of feeling troubled by these. This mirrors findings which show that certain occupations have a higher risk of developing work-related PTSD, such as professions that do not have special training to deal with traumatic events.[1] It is possible that military personnel expect to be exposed to moral traumas and are less troubled as a result.

Importantly, this meta-analysis identified several methodological issues. Prevalence of exposure to any MIE, transgressions by self and betrayals were lower in low risk compared to medium-risk studies and the prevalence of PTED differed depending on the cut-off value used. As highlighted by Koenig, Pearce, and Youssef[21] there are several moral injury scales, which conflate events and symptoms (MIES, MIQ) and our review has identified

For peer review only - http://bmjopen.bmj.com/site/about/guidelines.xhtml

more recently developed scales which also do this (i.e., MIA-PSP, Toronto scale for journalists). Other scales only measure symptoms (i.e., EMIS, MIOS, MISS-HP), many scales are occupation-specific precluding comparisons across occupations, and not all scales have established cut-off values, which contributes to inconsistencies in this area. Furthermore, there is a lack of nationally representative, epidemiological studies on the prevalence of moral injury and PTED.

These findings reveal several implications and gaps that can form the basis of future research. First, clinically relevant signs of PTED are much higher in work settings than in the general population, and just under half of the healthcare professionals showed clinical levels of moral injury. Furthermore, civilian occupations are especially likely to experience transgressions by others and betrayals at work. The narrative synthesis showed that moral injury and PTED are associated with poor mental health outcomes such as depression, stress and anxiety, and poor work outcomes such as sickness absence and turnover intentions. Therefore, employers, occupational health, and clinicians should be aware of these conditions, and provide appropriate support. The number of studies on moral injury has grown exponentially in recent years, however, research on occupational PTED still represents a significant gap and the lack of studies precluded prevalence subgroup analyses based on occupation. More studies are needed in this area. Second, whilst there were more moral injury studies, most were conducted with military or healthcare professionals, and other occupations such as education, journalists, CPS, and first responders were represented in only one or two studies. Prevalence estimates indicate educational and CPS employees may be at an especially high risk, and more studies are needed to confirm these findings. Finally, most moral injury studies were conducted in the US, and studies are needed across more geographical locations. Finally, there were only 10 studies with low risk of bias and more

For peer review only - http://bmjopen.bmj.com/site/about/guidelines.xhtml

large-scale high-quality epidemiological studies aimed at estimating the prevalence of PTED and moral injury are needed.

Figure 1.
Preferred Reporting Items for Systematic Reviews and Meta-Analyses (PRISMA) flow diagram.

For peer review only

Contributor and guarantor information:

CB designed the project and data collection tools, collected the data, cleaned, analysed and interpreted the data and drafted and revised the paper. She is guarantor. CR assisted with the study design, data collection and revised the paper. JC assisted with the study design and revised the paper. All authors gave final approval for the publication of this manuscript. The corresponding author attests that all listed authors meet authorship criteria and that no others meeting the criteria have been omitted.

Copyright:

The Corresponding Author has the right to grant on behalf of all authors and does grant on behalf of all authors, an exclusive licence (or non-exclusive for government employees) on a

worldwide basis to the BMJ Publishing Group Ltd to permit this article (if accepted) to be published in BMJ editions and any other BMJPGL products and sublicences such use and exploit all subsidiary rights, as set out in our licence.

Competing interest statement:

None declared

Funding:

CB was supported by the Economic and Social Research Council UK [ES/P000665/1].

Role of sponsor:

The study sponsors did not play a role in the study design, management, data collection, analyses, in the interpretation of the data or in the writing of the manuscript or the decision to submit for publication.

Transparency declaration:

The lead author affirms that this manuscript is an honest, accurate, and transparent account of the study being reported; that no important aspects of the study have been omitted; and that any discrepancies from the study as planned have been explained.

Ethical approval:

Ethical approval is not required for this study design.

Data availability statement:

No additional data are available.

Dissemination declaration:

Results from this study will be disseminated to relevant audiences through University of Liverpool communications.

For peer review only - http://bmjopen.bmj.com/site/about/guidelines.xhtml

For peer review only

References:

1. Skogstad M, Skorstad M, Lie A, et al. Work-related post-traumatic stress disorder. Occupational medicine. 2013 Apr 1;63(3):175-82. https://doi.org/10.1093/occmed/kqt003
2. Lee W, Lee YR, Yoon JH, et al. Occupational post-traumatic stress disorder: an updated systematic review. BMC public health. 2020 Dec;20(1):1-2. https://doi.org/10.1186/s12889-020-08903-2
3. Linden M, Baumann K, Lieberei B, et al. The post-traumatic embitterment disorder self-rating scale (PTED scale). Clinical Psychology & Psychotherapy: An

International Journal of Theory & Practice. 2009 Mar;16(2):139-47. https://doi.org/10.1002/cpp.610

4. Litz BT, Stein N, Delaney E, et al. Moral injury and moral repair in war veterans: A preliminary model and intervention strategy. Clinical psychology review. 2009 1;29(8):695-706. https://doi.org/10.1016/j.cpr.2009.07.003

5. Shay J. Moral injury. Psychoanalytic psychology. 2014 Apr;31(2):182. https://doi.org/10.1037/a0036090

6. Linden M. Posttraumatic embitterment disorder. Psychotherapy and psychosomatics. 2003;72(4):195-202. https://doi.org/10.1159/000070783

7. Linden M, Rotter M, Baumann K, et al. Posttraumatic embitterment disorder: Definition, evidence, diagnosis, treatment. Hogrefe & Huber Publishers; 2007.

8. Sabic D, Sabic A, Batic-Mujanovic O. Embitterment in war veterans with posttraumatic stress disorder. Medical Archives. 2018 Apr;72(2):125. doi: 10.5455/medarh.2018.72.125-130

9. Muschalla B, Rau H, Willmund GD, Knaevelsrud C. Work disability in soldiers with posttraumatic stress disorder, posttraumatic embitterment disorder, and not-event-related common mental disorders. Psychological trauma: theory, research, practice, and policy. 2018 Jan;10(1):30. https://doi.org/10.1037/tra0000293

10. Rubab M, Tariq F. Embitterment, Self-Efficacy and Mental Health Problems in private school teachers in Pakistan. PSYCHOLOGY AND EDUCATION. 2022;59(2):572-81.

11. Saleem A, Bashir M, Abrar M. Ethical behaviors by leaders act as a stimulant to the wellbeing of employees by restraining workplace embitterment. Frontiers in Public Health. 2022 Sep 29;10:974642. DOI: 10.3389/fpubh.2022.974642

For peer review only

For peer review only - http://bmjopen.bmj.com/site/about/guidelines.xhtml

12. Dunn JM, Sensky T. Psychological processes in chronic embitterment: The potential contribution of rumination. Psychological Trauma: Theory, Research, Practice, and Policy. 2018 Jan;10(1):7. https://doi.org/10.1037/tra0000291

13. Sensky T, Salimu R, Ballard J, et al. Associations of chronic embitterment among NHS staff. Occupational Medicine. 2015 Aug 1;65(6):431-6. https://doi.org/10.1093/occmed/kqv089

14. Brennan CJ, McKay MT, Cole JC. Morally injurious events and post-traumatic embitterment disorder in UK health and social care professionals during COVID-19: a cross-sectional web survey. BMJ open. 2022 May 1;12(5):e054062. doi: 10.1136/bmjopen-2021-054062

15. Shin J, You M. Embitterment among the unemployed: A multiple mediation model of belief in a just world. Work. 2022: 1-0. DOI: 10.3233/WOR-205228

16. Linden M, Rotter M. Unemployment and embitterment in contrast to general psychological distress. Work. 2019 Jan 1;62(1):133-8. DOI: 10.3233/WOR-182848

17. Michailidis E, Cropley M. Exploring predictors and consequences of embitterment in the workplace. Ergonomics. 2017 Sep 2;60(9):1197-206. https://doi.org/10.1080/00140139.2016.1255783

18. Michailidis E, Cropley M. Investigating the predictors of workplace embitterment using a longitudinal design. Occupational Medicine. 2018 Nov 16;68(8):523-9. https://doi.org/10.1093/occmed/kqy121

19. Carter C. Organisational injustice in UK frontline services and onset of Moral Injury, Post Traumatic Embitterment Disorder (PTED) and PTSD. International journal of law, crime and justice. 2021 Sep 1;66:100483 https://doi.org/10.1016/j.ijlcj.2021.100483

For peer review only

20. Nash WP, Marino Carper TL, Mills MA, Au T, Goldsmith A, Litz BT. Psychometric evaluation of the moral injury events scale. Military medicine. 2013 Jun 1;178(6):646-52. https://doi.org/10.7205/MILMED-D-13-00017

21. Koenig HG, Youssef NA, Pearce M. Assessment of moral injury in veterans and active duty military personnel with PTSD: A review. Frontiers in psychiatry. 2019 Jun 28;10:443. https://doi.org/10.3389/fpsyt.2019.00443

22. Williamson V, Stevelink SA, Greenberg N. Occupational moral injury and mental health: systematic review and meta-analysis. The British Journal of Psychiatry. 2018 Jun;212(6):339-46. DOI: https://doi.org/10.1192/bjp.2018.55

23. Linden M, Baumann K, Rotter M, Schippan B. Diagnostic criteria and the standardized diagnostic interview for posttraumatic embitterment disorder (PTED). International Journal of Psychiatry in Clinical Practice. 2008 Jan 1;12(2):93-6. https://doi.org/10.1080/13651500701580478

24. Frankfurt S, Frazier P. A review of research on moral injury in combat veterans. Military psychology. 2016 Jun 9;28(5):318-30. https://doi.org/10.1037/mil0000132

25. Koenig HG, Al Zaben F. Moral injury: An increasingly recognized and widespread syndrome. Journal of religion and health. 2021 Oct;60(5):2989-3011. https://doi.org/10.1007/s10943-021-01328-0

26. Page MJ, McKenzie JE, Bossuyt PM, Boutron I, Hoffmann TC, Mulrow CD, Shamseer L, Tetzlaff JM, Akl EA, Brennan SE, Chou R. The PRISMA 2020 statement: an updated guideline for reporting systematic reviews. Systematic reviews. 2021 Dec;10(1):1-1. https://doi.org/10.1186/s13643-021-01626-4

27. Munn Z, Moola S, Lisy K, Riitano D, Tufanaru C. Methodological guidance for systematic reviews of observational epidemiological studies reporting prevalence

For peer review only

For peer review only - http://bmjopen.bmj.com/site/about/guidelines.xhtml

and cumulative incidence data. International journal of evidence-based healthcare. 2015 Sep 1;13(3):147-53. DOI: 10.1097/XEB.0000000000000054

28. McHugh ML. Interrater reliability: the kappa statistic. Biochemia medica. 2012 Oct 15;22(3):276-82.30

29. Hoy D, Brooks P, Woolf A, et al. Assessing risk of bias in prevalence studies: modification of an existing tool and evidence of interrater agreement. Journal of clinical epidemiology. 2012 Sep 1;65(9):934-9. https://doi.org/10.1016/j.jclinepi.2011.11.014

30. Langan D, Higgins JP, Jackson D, Bowden J, Veroniki AA, Kontopantelis E, Viechtbauer W, Simmonds M. A comparison of heterogeneity variance estimators in simulated random-effects meta-analyses. Research synthesis methods. 2019 Mar;10(1):83-98. https://doi.org/10.1002/jrsm.1316

31. Schwarzer G. Meta-Analysis in R. Systematic Reviews in Health Research: Meta-Analysis in Context. 2022 Apr 22:510-34. https://doi.org/10.1002/9781119099369.ch26

32. Borenstein M, Hedges LV, Higgins JP, Rothstein HR. Introduction to meta-analysis. John Wiley & Sons; 2021 Apr 6.

33. Cumming G, Finch S. Inference by eye: confidence intervals and how to read pictures of data. American psychologist. 2005 Feb;60(2):170. https://doi.org/10.1037/0003-066X.60.2.170

34. Lipsey MW, Wilson DB. Practical meta-analysis. SAGE publications, Inc; 2001.

35. Viechtbauer W. Conducting meta-analyses in R with the metafor package. Journal of statistical software. 2010;36(3):1-48. DOI: 10.18637/jss.v036.i03

For peer review only

36. Higgins JP, Thompson SG. Quantifying heterogeneity in a meta-analysis. Statistics in medicine. 2002 Jun 15;21(11):1539-58. https://doi.org/10.1002/sim.1186

37. Wang N. How to conduct a meta-analysis of proportions in R: a comprehensive tutorial. New York, NY, John Jay College of Criminal Justice, 0–62. 2018 Jun.

38. Viechtbauer W, Cheung MW. Outlier and influence diagnostics for meta-analysis. Research synthesis methods. 2010 Apr;1(2):112-25. https://doi.org/10.1002/jrsm.11

39. Popay J, Roberts H, Sowden A, Petticrew M, Arai L, Rodgers M, Britten N, Roen K, Duffy S. Guidance on the conduct of narrative synthesis in systematic reviews. A product from the ESRC methods programme Version. 2006 Apr 1;1(1):b92.

40. Karatuna I, Gök S. A study analyzing the association between post-traumatic embitterment disorder and workplace bullying. Journal of Workplace Behavioral Health. 2014 Apr 3;29(2):127-42

41. Bryan CJ, Bryan AO, Anestis MD, et al. Measuring moral injury: Psychometric properties of the moral injury events scale in two military samples. Assessment. 2016 Oct;23(5):557-70. https://doi.org/10.1177/1073191115590855

42. Currier JM, Holland JM, Drescher K, et al. Initial psychometric evaluation of the Moral Injury Questionnaire—Military version. Clinical psychology & psychotherapy. 2015 Jan;22(1):54-63. https://doi.org/10.1002/cpp.1866

43. Currier JM, Holland JM, Rojas-Flores L, et al. Morally injurious experiences and meaning in Salvadorian teachers exposed to violence. Psychological trauma: theory, research, practice, and policy. 2013;7(1):24. https://doi.org/10.1037/a0034092

For peer review only

44. Currier JM, Farnsworth JK, Drescher KD, et al. Development and evaluation of the Expressions of Moral Injury Scale—Military Version. Clinical psychology & psychotherapy. 2017 May;25(3):474-88. https://doi.org/10.1002/cpp.2170

45. Forkus SR, Breines JG, Weiss NH. Morally injurious experiences and mental health: The moderating role of self-compassion. Psychological trauma: theory, research, practice, and policy. 2019 Sep;11(6):630. https://doi.org/10.1037/tra0000446

46. Frankfurt SB, DeBeer BB, Morissette SB, Kimbrel NA, La Bash H, Meyer EC. Mechanisms of moral injury following military sexual trauma and combat in post-9/11 US war veterans. Frontiers in psychiatry. 2018 Nov 2;9:520. https://doi.org/10.3389/fpsyt.2018.00520

47. Griffin BJ, Williams CL, Shaler L, Dees RF, Cowden RG, Bryan CJ, Litz B, Purcell N, Burkman K, Maguen S. Profiles of moral distress and associated outcomes among student veterans. Psychological Trauma: Theory, Research, Practice, and Policy. 2020 Oct;12(7):669. https://doi.org/10.1037/tra0000584

48. Jinkerson JD, Battles AR. Relationships between moral injury syndrome model variables in combat veterans. Traumatology. 2019 Mar;25(1):33. https://doi.org/10.1037/trm0000163

49. Jordan AH, Eisen E, Bolton E, Nash WP, Litz BT. Distinguishing war-related PTSD resulting from perpetration-and betrayal-based morally injurious events. Psychological Trauma: Theory, Research, Practice, and Policy. 2017 Nov;9(6):627. https://doi.org/10.1037/tra0000249

50. Lee H, Aldwin CM, Kang S. Do different types of war stressors have independent relations with mental health? Findings from the Korean Vietnam Veterans Study.

For peer review only - http://bmjopen.bmj.com/site/about/guidelines.xhtml

Psychological trauma: theory, research, practice, and policy. 2022 Jul;14(5):840. https://doi.org/10.1037/tra0000557

51. Ogle AD, Reichwald R, Rutland JB. Psychological impact of remote combat/graphic media exposure among US Air Force intelligence personnel. Military Psychology. 2018 Nov 2;30(6):476-86. https://doi.org/10.1080/08995605.2018.1502999

52. Wisco BE, Marx BP, May CL, Martini B, Krystal JH, Southwick SM, Pietrzak RH. Moral injury in US combat veterans: Results from the national health and resilience in veterans study. Depression and anxiety. 2017 Apr;34(4):340-7. https://doi.org/10.1002/da.22614

53. Zerach G, Levi-Belz Y. Moral injury process and its psychological consequences among Israeli combat veterans. Journal of Clinical Psychology. 2018 Sep;74(9):1526-44. https://doi.org/10.1002/jclp.22598

54. Papazoglou K, Blumberg D, Briones-Chiongbian V, Russo C, Koskelainen M. Exploring the roles of moral injury and personality in police traumatization. Crisis, Stress, and Human Resilience: An International Journal. 2019 Apr 25;1(1):32-56.

55. Richardson CB, Chesnut RP, Morgan NR, et al. Examining the factor structure of the moral injury events scale in a veteran sample. Military medicine. 2020 Jan;185(1-2):e75-83. https://doi.org/10.1093/milmed/usz129

56. Feinstein A, Pavisian B, Storm H. Journalists covering the refugee and migration crisis are affected by moral injury not PTSD. JRSM open. 2018 Feb;9(3):2054270418759010. https://doi.org/10.1177/2054270418759010

For peer review only - http://bmjopen.bmj.com/site/about/guidelines.xhtml

57. Sugrue EP. Moral injury among professionals in K–12 education. American Educational Research Journal. 2020 Feb;57(1):43-68. https://doi.org/10.3102/00028312198486

58. Aldridge V, Scott H, Paskell R. Investigating the relationship between guilt and shame proneness and moral injury in veterans that have experienced active deployment. Military Behavioral Health. 2019 Jan 2;7(1):83-91. https://doi.org/10.1080/21635781.2019.1580641

59. Andrukonis A, Protopopova A. Occupational health of animal shelter employees by live release rate, shelter type, and euthanasia-related decision. Anthrozoös. 2020 Jan 2;33(1):119-31.

60. Haight W, Sugrue EP, Calhoun M. Moral injury among child protection professionals: Implications for the ethical treatment and retention of workers. Children and Youth Services Review. 2017 Nov 1;82:27-41. https://doi.org/10.1016/j.childyouth.2017.08.030

61. Battles AR, Kelley ML, Jinkerson JD, C. Hamrick H, F. Hollis B. Associations among exposure to potentially morally injurious experiences, spiritual injury, and alcohol use among combat veterans. Journal of Traumatic Stress. 2019 Jun;32(3):405-13. https://doi.org/10.1002/jts.22404

62. Braitman AL, Battles AR, Kelley ML, Hamrick HC, Cramer RJ, Ehlke S, Bravo AJ. Psychometric properties of a Modified Moral Injury Questionnaire in a military population. Traumatology. 2018 Dec;24(4):301. https://doi.org/10.1037/trm0000158

63. Lancaster SL, Harris JI. Measures of morally injurious experiences: A quantitative comparison. Psychiatry research. 2018 Jun 1;264:15-9. https://doi.org/10.1016/j.psychres.2018.03.057

For peer review only

64. Lancaster SL. Negative outcomes after morally injurious experiences: A replication and extension. Psychological Trauma: Theory, Research, Practice, and Policy. 2018 Jul;10(4):456. https://doi.org/10.1037/tra0000341

65. Akhtar M, Faize FA, Malik RZ, et al. Moral injury and psychological resilience among healthcare professionals amid COVID-19 pandemic. Pakistan Journal of Medical Sciences. 2022 May 18;38(5). https://doi.org/10.12669/pjms.38.5.5122

66. Qi M, Hu X, Liu J, et al. The impact of the COVID-19 pandemic on the prevalence and risk factors of workplace violence among healthcare workers in China. Frontiers in public health. 2022;10. https://doi.org/10.3389/fpubh.2022.938423

67. Amsalem D, Lazarov A, Markowitz JC, et al. Psychiatric symptoms and moral injury among US healthcare workers in the COVID-19 era. BMC psychiatry. 2021 Dec;21(1):1-8. https://doi.org/10.1186/s12888-021-03565-9

68. Perez D, Larson P, Bair J. US veterans experience moral injury differently based on moral foundations preferences. Journal of Community Engagement and Scholarship. 2021 Aug;13(4):13.

69. Benatov J, Zerach G, Levi-Belz Y. Moral injury, depression, and anxiety symptoms among health and social care workers during the COVID-19 pandemic: The moderating role of belongingness. International journal of social psychiatry. 2022:00207640221099421. https://doi.org/10.1177/00207640221099421

70. Borges LM, Holliday R, Barnes SM, et al. A longitudinal analysis of the role of potentially morally injurious events on COVID-19-related psychosocial functioning among healthcare providers. PloS one. 2021 Nov 12;16(11):e0260033. https://doi.org/10.1371/journal.pone.0260033

For peer review only

For peer review only - http://bmjopen.bmj.com/site/about/guidelines.xhtml

71. Boscarino JA, Adams RE, Wingate TJ, et al. Impact and risk of moral injury among deployed veterans: implications for veterans and mental health. Frontiers in psychiatry. 2022;13. https://doi.org/10.3389/fpsyt.2022.899084

72. Brady C, Fenton C, Loughran O, et al. Dublin hospital workers' mental health during the peak of Ireland's COVID-19 pandemic. Irish Journal of Medical Science (1971-). 2022 Jun 22:1-0. https://doi.org/10.1007/s11845-022-03056-0

73. Brady C, Fenton C, Loughran O, et al. Nursing home staff mental health during the Covid-19 pandemic in the Republic of Ireland. International journal of geriatric psychiatry. 2021; 37(1), 1-10. https://doi.org/10.1002/gps.5648

74. Chandrabhatla T, Asgedom H, Gaudiano ZP, de Avila L, Roach KL, Venkatesan C, Weinstein AA, Younossi ZM. Second victim experiences and moral injury as predictors of hospitalist burnout before and during the COVID-19 pandemic. Plos one. 2022 Oct 4;17(10):e0275494. https://doi.org/10.1371/journal.pone.0275494

75. Dale LP, Cuffe SP, Sambuco N, et al. Morally distressing experiences, moral injury, and burnout in Florida healthcare providers during the COVID-19 pandemic. International Journal of Environmental Research and Public Health. 2021 Nov 24;18(23):12319. https://doi.org/10.3390/ijerph182312319

76. Plouffe RA, Easterbrook B, Liu A, et al. Psychometric evaluation of the moral injury events scale in two Canadian armed forces samples. Assessment. 2023 Jan;30(1):111-23. https://doi.org/10.1177/10731911211044198

77. Nichter B, Norman SB, Maguen S, et al. Moral injury and suicidal behavior among US combat veterans: Results from the 2019–2020 National Health and Resilience in Veterans Study. Depression and anxiety. 2021 Jun;38(6):606-14. https://doi.org/10.1002/da.23145

For peer review only - http://bmjopen.bmj.com/site/about/guidelines.xhtml

78. Fitzpatrick JJ, Pignatiello G, Kim M, et al. Moral Injury, Nurse Well-being, and Resilience Among Nurses Practicing During the COVID-19 Pandemic. JONA: The Journal of Nursing Administration. 2022 Jul 1;52(7/8):392-8. DOI: 10.1097/NNA.0000000000001171

79. Hagerty SL, Williams LM. Moral Injury, Traumatic Stress, and Threats to Core Human Needs in Health-Care Workers: The COVID-19 Pandemic as a Dehumanizing Experience. Clinical Psychological Science. 2022 Jan 10:21677026211057554. https://doi.org/10.1177/21677026211057554

80. Hamrick HC, Ehlke SJ, Davies RL, et al. Moral injury as a mediator of the associations between sexual harassment and mental health symptoms and substance use among women veterans. Journal of interpersonal violence. 2022 Jun;37(11-12):NP10007-35. https://doi.org/10.1177/0886260520985485

81. Hines SE, Chin KH, Levine AR, et al. Initiation of a survey of healthcare worker distress and moral injury at the onset of the COVID-19 surge. American journal of industrial medicine. 2020 Sep 1. DOI: 10.1002/ajim.23157

82. Hinkel HM, Currier JM, Isaak SL, et al. Moral injury and chronic pain among military veterans in an integrated behavioral health clinic. Psychological trauma: theory, research, practice, and policy. 2022 Mar 24. https://doi.org/10.1037/tra0001230

83. Kelley ML, Chae JW, Bravo AJ, et al. Own soul's warning: Moral injury, suicidal ideation, and meaning in life. Psychological trauma: theory, research, practice, and policy. 2021 Aug 5. https://doi.org/10.1037/tra0001047

84. La Fleur R, McDonald M, Senecal G, et al. The Impact of Moral Injury and Disclosure of Military Experiences on Veterans. Journal of Humanistic

For peer review only

Psychology. 2020 Nov 20:0022167820972214. https://doi.org/10.1177/0022167820972214

85. Lamb D, Gnanapragasam S, Greenberg N, Bhundia R, et al. Psychosocial impact of the COVID-19 pandemic on 4378 UK healthcare workers and ancillary staff: initial baseline data from a cohort study collected during the first wave of the pandemic. Occupational and environmental medicine. 2021 Nov 1;78(11):801-8. http://dx.doi.org/10.1136/oemed-2020-107276

86. Litam SD, Balkin RS. Moral injury in health-care workers during COVID-19 pandemic. Traumatology. 2021 Mar;27(1):14. https://doi.org/10.1037/trm0000290

87. Litz BT, Plouffe RA, Nazarov A, et al. Defining and assessing the syndrome of moral injury: initial findings of the Moral Injury Outcome Scale Consortium. Frontiers in psychiatry. 2022;13. https://doi.org/10.3389/fpsyt.2022.923928

88. Maftei A, Holman AC. The prevalence of exposure to potentially morally injurious events among physicians during the COVID-19 pandemic. European Journal of Psychotraumatology. 2021 Jan 1;12(1):1898791. https://doi.org/10.1080/20008198.2021.1898791

89. Maguen S, Griffin BJ, Vogt D, et al. Moral injury and peri-and post-military suicide attempts among post-9/11 veterans. Psychological medicine. 2022 Jan 17:1-0. https://doi.org/10.1017/S0033291721005274

90. Malakoutikhah A, Zakeri MA, Koenig HG, et al. The psychometric properties of the Persian version of the moral injury symptoms scale-health care professionals version. Frontiers in psychology. 2022:4803. https://doi.org/10.3389/fpsyg.2022.978572

For peer review only - http://bmjopen.bmj.com/site/about/guidelines.xhtml

91. Mantri S, Lawson JM, Wang Z, et al. Identifying moral injury in healthcare professionals: The moral injury symptom scale-HP. Journal of religion and health. 2020 Oct;59(5):2323-40. https://doi.org/10.1007/s10943-020-01065-w

92. Mantri S, Song YK, Lawson JM, et al. Moral injury and burnout in health care professionals during the COVID-19 pandemic. The Journal of Nervous and Mental Disease. 2021 Oct 1;209(10):720-6. DOI: 10.1097/NMD.0000000000001367

93. Morris DJ, Webb EL, Devlin P. Moral injury in secure mental healthcare part II: experiences of potentially morally injurious events and their relationship to wellbeing in health professionals in secure services. The Journal of Forensic Psychiatry & Psychology. 2022 Sep 3;33(5):726-44. https://doi.org/10.1080/14789949.2022.2111319

94. Rushton CH, Thomas TA, Antonsdottir IM, Net al. Moral injury and moral resilience in health care workers during COVID-19 pandemic. Journal of palliative medicine. 2022 May 1;25(5):712-9. https://doi.org/10.1089/jpm.2021.0076

95. Nieuwsma JA, Brancu M, Wortmann J, et al. Screening for moral injury and comparatively evaluating moral injury measures in relation to mental illness symptomatology and diagnosis. Clinical Psychology & Psychotherapy. 2021 Jan;28(1):239-50. https://doi.org/10.1002/cpp.2503

96. Osmann J, Page-Gould E, Inbar Y, et al. Validation of the Toronto Moral Injury Scale for Journalists. Traumatology. 2022 Aug 25. https://doi.org/10.1037/trm0000409

97. Rodríguez EA, Agüero-Flores M, Landa-Blanco M, et al. Moral injury and light triad traits: anxiety and depression in health-care personnel during the

For peer review only

coronavirus-2019 pandemic in Honduras. Hispanic health care international. 2021 Dec;19(4):230-8. https://doi.org/10.1177/1540415321104237

98. Roth SL, Andrews K, Protopopescu A, et al. Development and preliminary evaluation of the moral injury assessment for public safety personnel. Traumatology. 2022 Mar 3. https://doi.org/10.1037/trm0000367

99. Russell B, Mussap AJ. Rumination and threat-biased interpretation mediate posttraumatic stress and growth responses to military stressors. Military Psychology. 2022 Oct 2:1-6. https://doi.org/10.1080/08995605.2022.2127618

100. Saba SK, Davis JP, Lee DS, et al. Moral injury events and behavioral health outcomes among American veterans. Journal of anxiety disorders. 2022 Aug 1;90:102605. https://doi.org/10.1016/j.janxdis.2022.102605

101. Senger AR, Torres D, Ratcliff CG. Potentially morally injurious events as a mediator of the association of gratitude and mindfulness with distress. Psychological Trauma: Theory, Research, Practice, and Policy. 2022 Mar 31. https://doi.org/10.1037/tra0001233

102. Stanojević S, Čartolovni A. Moral distress and moral injury and their interplay as a challenge for leadership and management: The case of Croatia. Journal of Nursing Management. 2022 Oct 4. https://doi.org/10.1111/jonm.13835

103. Testoni I, Brondolo E, Ronconi L, et al. Burnout following moral injury and dehumanization: A study of distress among Italian medical staff during the first COVID-19 pandemic period. Psychological Trauma: Theory, Research, Practice, and Policy. 2022 Aug 4. https://doi.org/10.1037/tra0001346

104. Thomas ED, Weiss NH, Forkus SR, et al. Examining the interaction between potentially morally injurious events and religiosity in relation to alcohol misuse

For peer review only

among military veterans. Journal of Traumatic Stress. 2022 Feb;35(1):314-20. https://doi.org/10.1002/jts.22682

105. Trifunovic-Koenig M, Strametz R, Gerber B, Mantri S, Bushuven S. Validation of the German Version of the Moral Injury Symptom and Support Scale for Health Professionals (G-MISS-HP) and Its Correlation to the Second Victim Phenomenon. International Journal of Environmental Research and Public Health. 2022 Jan;19(8):4857. https://doi.org/10.3390/ijerph19084857

106. Ulusoy S, Çelik Z. The Silent Cry of Healthcare Workers: A Cross-Sectional Study on Levels and Determinants of Burnout among Healthcare Workers after First Year of the Pandemic in Turkey. Psychiatry and Clinical Psychopharmacology. 2022 Mar 1;32(1):63-71. DOI:10.5152/pcp.2022.21248

107. Williamson V, Murphy D, Greenberg N. Experiences and impact of moral injury in UK veterinary professional wellbeing. European journal of psychotraumatology. 2022 Dec 31;13(1):2051351. https://doi.org/10.1080/20008198.2022.2051351

108. Zahiriharsini A, Gilbert-Ouimet M, Langlois L, Biron C, Pelletier J, Beaulieu M, Truchon M. Associations between psychosocial stressors at work and moral injury in frontline healthcare workers and leaders facing the COVID-19 pandemic in Quebec, Canada: A cross-sectional study. Journal of Psychiatric Research. 2022 Nov 1;155:269-78. https://doi.org/10.1016/j.jpsychires.2022.09.006

109. Levi-Belz Y, Shemesh S, Zerach G. Moral injury and suicide ideation among combat veterans: The moderating role of self-disclosure. Crisis: The Journal of Crisis Intervention and Suicide Prevention. 2022 Feb 9. https://doi.org/10.1027/0227-5910/a000849

For peer review only

110. Schwartz G, Halperin E, Levi-Belz Y. Moral injury and suicide ideation among combat veterans: The role of trauma-related shame and collective hatred. Journal of interpersonal violence. 2022 Aug;37(15-16):NP13952-77. DOI: 10.1177/08862605211007932

111. Currier JM, Isaak SL, McDermott RC. Validation of the Expressions of Moral Injury Scale-Military version-short form. Clinical psychology & psychotherapy. 2020 Jan;27(1):61-8. https://doi.org/10.1002/cpp.2407

112. Hall NA, Everson AT, Billingsley MR, Miller MB. Moral injury, mental health and behavioural health outcomes: A systematic review of the literature. Clinical psychology & psychotherapy. 2022 Jan;29(1):92-110. https://doi.org/10.1002/cpp.2607

113. McEwen C, Alisic E, Jobson L. Moral injury and mental health: A systematic review and meta-analysis. Traumatology. 2021 Sep;27(3):303. https://doi.org/10.1037/trm0000287

114. Migliavaca CB, Stein C, Colpani V, Barker TH, Ziegelmann PK, Munn Z, Falavigna M, Prevalence Estimates Reviews—Systematic Review Methodology Group (PERSyst). Meta-analysis of prevalence: I 2 statistic and how to deal with heterogeneity. Research synthesis methods. 2022 May;13(3):363-7. https://doi.org/10.1002/jrsm.1547

115. French L, Hanna P, Huckle C. “If I die, they do not care”: UK National Health Service staff experiences of betrayal-based moral injury during COVID-19. Psychological Trauma: Theory, Research, Practice, and Policy. 2022 Mar;14(3):516. https://doi.org/10.1037/tra0001134

116. Hegarty S, Lamb D, Stevelink SA, Bhundia R, Raine R, Doherty MJ, Scott HR, Marie Rafferty A, Williamson V, Dorrington S, Hotopf M. ‘It hurts your heart’:

For peer review only

frontline healthcare worker experiences of moral injury during the COVID-19 pandemic. European journal of psychotraumatology. 2022 Dec 19;13(2):2128028. https://doi.org/10.1080/20008066.2022.2128028

For peer review only

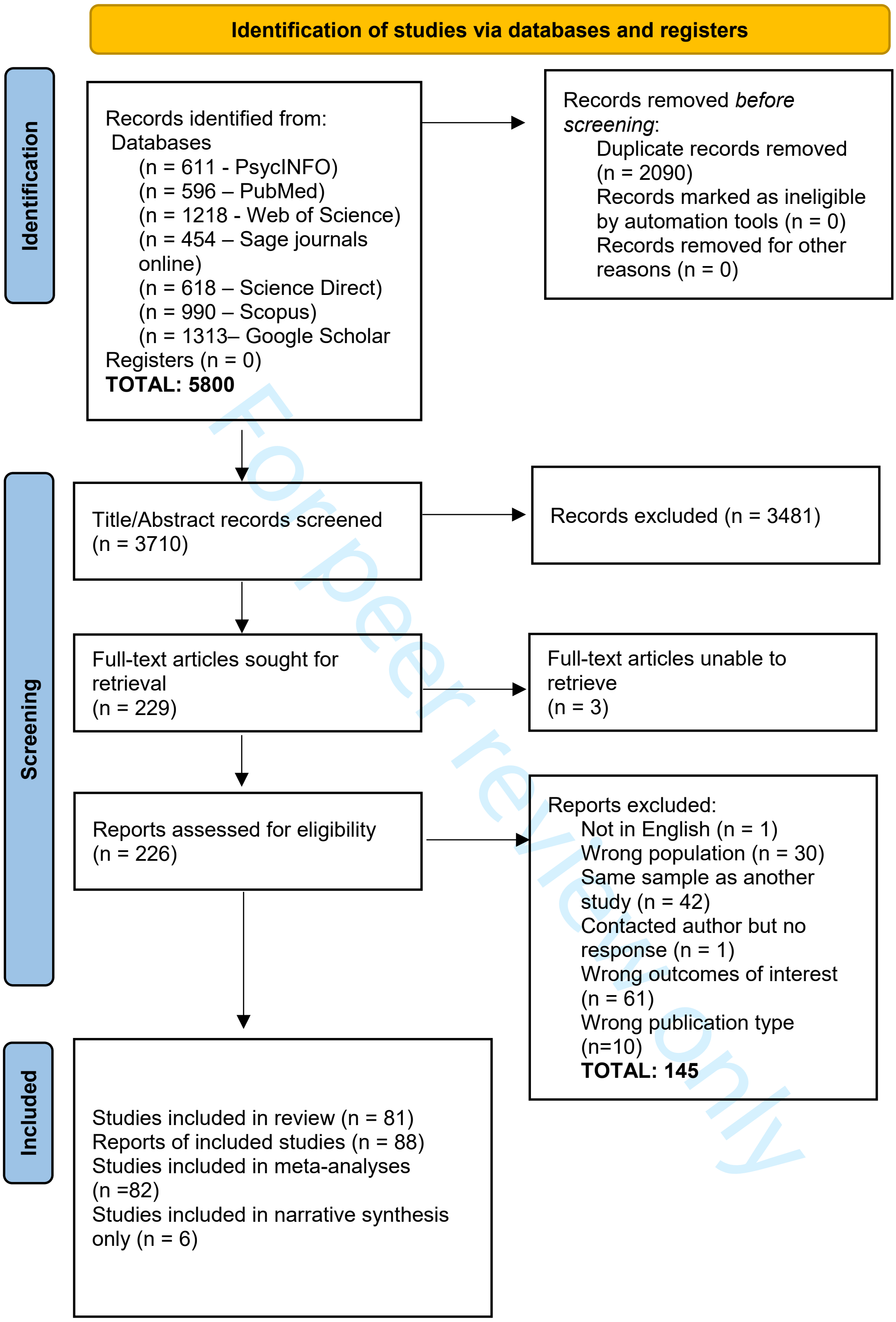

For peer review only

Supplementary materials:

Supplementary table 1. Search terms used in electronic literature databases

Keyword	Search engine variants
#1. PTED	"post-traumatic embitterment disorder", PTED, embitterment, embittered.
#2. Moral injury	"mora* inju*", "transgressive act*", "moral transgression".
#3. Occupation	Veteran* OR military OR army OR marine* OR "national service" OR "territorial army" OR "armed forces" OR "special forces" OR RAF OR "air force" OR navy OR "service personnel" OR "ex-service personnel" OR submariner* OR sailor* OR seamen OR seaman OR airmen OR airman OR soldier* OR troop* OR "enlisted personnel" OR "armed forces" OR "armed services" OR police* OR fireman OR firemen OR firefighter* OR teacher* OR "humanitarian aid worker" OR "relief aid worker" OR "social worker" OR "disaster responder" OR policeman OR policemen OR policewoman OR "law enforcement" OR healthcare OR nurse OR doctor OR frontline OR "health professional" OR "hospital staff" OR "hospital worker" OR work OR occupation OR job OR "emergency service*"

#1 AND #3 (PTED searches). #1 AND #2 (moral injury searches). Limits: English, publication year >2003 for PTED and >2009 for moral injury.

For peer review only

For peer review only - http://bmjopen.bmj.com/site/about/guidelines.xhtml

Supplementary table 2. Hoy Risk of Bias for included studies

Authors (Year)	Representative of national population	Representative of target population	Random selection	Non-response bias	Directly collected	Acceptable case definition	Reliable and valid instrument	Same mode of data collection	Appropriate numerator(s) and denominator(s) for the parameter of interest	Hoy score
**PTED only studies**										
Dunn & Sensky 2018	1	1	1	1	0	0	0	0	1	5
Linden & Rotter 2019	1	1	1	1	0	0	0	0	1	5
Michailidis & Cropley 2018	1	1	1	1	0	0	0	0	1	5
Sensky et al 2015	1	0	1	1	0	0	0	0	1	4
Sabic, Sabic, & O Baltic-Mujanovic 2018	1	1	1	1	0	0	0	0	1	5
Michailids & Cropley 2017	1	1	1	1	0	0	0	0	1	5
Karatuna & Gok 2014	1	1	1	1	0	0	0	0	1	5
Rubab & Tariq 2022.	1	1	1	1	0	0	0	0	1	5

For peer review only

For peer review only - http://bmjopen.bmj.com/site/about/guidelines.xhtml

Saleem, Bashir & Abrar, 2022	1	1	0	1	0	0	0	1	1	5
Shin & You 2021	1	1	1	1	0	0	0	0	1	5
**PTED and moral injury study**										
Brennan, McKay & Cole, 2022	1	0	1	0	0	0	0	0	0	2
**Moral injury only studies**										
Bryan et al., 2016	1	1	1	1	0	0	0	0	1	5
Currier et al., 2015	0	0	1	1	0	0	0	0	1	3
Currier et al., 2013	1	1	1	0	0	0	0	0	1	4
Currier, et al., 2017	1	1	1	1	0	0	0	0	1	5
Forkus, Juliana & Weiss. 2019	1	1	1	1	0	0	0	0	1	5
Frankfurt et al. 2018.	1	0	1	1	0	0	0	0	1	4
Griffin et al., 2020	1	1	1	1	0	0	0	0	1	5
Jinkerson & Battles. 2019.	1	1	1	0	0	0	0	0	1	4
Jordan & Nash. 2017.	1	1	1	1	0	0	0	0	1	5
Lee, Aldwin & Kang. 2020	1	1	1	1	0	0	0	0	1	5
Ogle, Reichwald, & Rutland. 2018	1	1	1	1	0	0	0	0	1	5

For peer review only - http://bmjopen.bmj.com/site/about/guidelines.xhtml

For peer review only

Wisco et al. 2017.	0	0	0	1	0	0	0	0	0	1
Zerach & Levi-Belz. 2017	1	1	1	0	0	0	0	0	1	4
Papazoglou et al. 2019.	1	1	1	0	0	0	0	0	1	4
Richardson et al. 2020	0	0	0	1	0	0	0	0	1	2
Feinstein, Pavisian, Storm H. 2018	1	1	1	1	0	0	0	0	1	5
Sugrue. 2020	1	1	1	1	0	0	0	0	1	5
Aldridge, Scott, Paskell. 2019.	1	1	1	1	0	0	0	0	1	5
Andrukonis, Protopopova. 2020.	1	1	1	1	0	0	0	0	1	5
Haight, Sugrue & Calhoun. 2017.	1	1	1	1	0	0	0	0	1	5
Battles et al. 2019.	1	1	1	1	0	0	0	0	1	5
Braitman et al. 2018.	1	1	1	1	0	0	0	0	1	5
Lancaster & Harris. 2018.	1	1	1	1	0	0	0	0	1	5
Lancaster. 2018.	1	1	1	1	0	0	0	0	1	5
Aktar, Faize, Malik & Tabusam, 2022	1	1	1	1	0	0	0	1	1	6
Qi et al., 2022	1	1	1	1	0	0	0	0	1	5
Ansalem et al., 2021	1	1	1	1	0	0	0	0	1	5
Perez, Larson & Bair 2021	1	1	1	1	0	0	0	0	1	5
Benatoz, Zerach & Levi-Belz 2022	1	1	1	1	0	0	0	0	1	5

For peer review only

Borges et al., 2021	1	1	1	1	0	0	0	0	1	5
Boscarino et al., 2022	1	0	0	1	0	0	0	0	1	3
Brady et al., 2021	0	0	1	1	0	0	0	0	1	4
Brady et al., 2022	1	1	1	1	0	0	0	0	1	6
Chandrabhatla et al., 2022	1	1	1	1	0	0	0	0	1	5
Dale et al., 2021	1	1	1	1	0	0	0	0	1	5
Plouffe et al., 2021	0	0	0	1	0	0	0	0	1	2
Nichter et al., 2020	0	0	0	1	0	0	0	0	0	1
Fitzpatrick et al., 2022	1	1	1	1	0	0	0	0	1	5
Hagerty & Williams, 2022	1	1	1	1	0	0	0	0	1	5
Hamrick et al., 2022	1	1	1	1	0	0	0	0	1	5
Hines et al., 2021	1	1	1	1	0	0	0	0	1	5
Hinkel et al., 2022	1	1	1	1	0	0	0	0	1	5
Kelley et al., 2021	1	1	1	1	0	0	0	0	1	5
La Fleur et al., 2020	1	1	1	1	0	0	0	1	1	6

For peer review only

Lamb et al., 2021	1	0	0	1	0	0	0	0	0	2
Litam et al., 2021	1	1	1	1	0	0	0	0	1	5
Litz et al., 2022 Samples 1, 2 and 3	1	1	1	1	0	0	0	0	1	5
Litz et al., 2022 Sample 4	1	1	1	1	0	0	0	1	1	6
Maftei & Holman. 2021	1	1	1	1	0	0	0	0	1	5
Maguen et al., 2021	0	0	0	1	0	0	0	0	1	2
Malakoutikhah et al., 2022	1	1	1	1	0	0	0	0	1	5
Mantri et al., 2020	1	1	1	1	0	0	0	0	1	5
Mantri et al., 2021	1	1	1	1	0	0	0	0	1	5
Morris, Webb & Devlin, 2022	1	1	1	1	0	0	0	0	1	5
Rushton et al., 2022	1	1	1	1	0	0	0	0	1	5
Nieuwsma et al., 2020	1	1	1	1	0	0	0	0	1	5
Osmann et al., 2022	1	1	1	1	0	0	0	0	1	5
Rodríguez et al., 2021	1	1	1	1	0	0	0	0	1	5

For peer review only - http://bmjopen.bmj.com/site/about/guidelines.xhtml

Roth et al., 2022	1	1	1	1	0	0	0	0	1	5
Russel & Mussap, 2022	1	1	1	1	0	0	0	0	1	5
Saba et al., 2022	1	1	1	1	0	0	0	0	1	5
Senger, Torres & Ratcliff, 2022	1	1	1	1	0	0	0	1	1	6
Stanojević & Čartolovni, 2022	1	1	1	1	0	0	0	0	1	5
Testoni et al., 2022	1	1	1	1	0	0	0	0	1	5
Thomas, Weiss, Forkus & Contractor, 2022	1	1	1	1	0	0	0	0	1	5
Trifunovic-Koenig, et al 2022	1	1	1	1	0	0	0	0	1	5
Ulusoy & Çelik 2022	1	1	1	1	0	0	0	0	1	5
Williamson, Murphy & Greenberg, 2022	1	1	1	1	0	0	0	0	1	5
Zahiriharsini et al., 2022	1	1	1	1	0	0	0	0	1	5
Levi-Belz, Shemesh & Zerach 2022	1	1	1	1	0	0	0	0	1	5
Schwartz, Halperin & Levi-Belz,	1	1	1	1	0	0	0	0	1	5

Note: 1 = no (high risk), 0 = no (low risk) 7-9 = High, 4-6 = Medium, 0-3 = Low.

For peer review only - http://bmjopen.bmj.com/site/about/guidelines.xhtml

For peer review only

Supplementary figures: Forest Plots for Meta-Analysis Findings

*Analyses using **prevalence** estimates as outcome*

Supplementary figure 1. PTED prevalence using 1.6 cut off

Study	Cases	Total	Prevalence	95% C.I.
Dunn 2018	37	79	46.84	[35.51; 58.40]
Michailids 2018	55	352	15.62	[11.99; 19.85]
Sensky 2015	68	236	28.81	[23.12; 35.05]
Sabic 2018	15	87	17.24	[9.98; 26.84]
Michailids 2017	253	337	75.07	[70.10; 79.60]
Katatuna 2014	106	397	26.70	[22.41; 31.34]
Rubab 2022	121	300	40.33	[34.74; 46.12]
Shin 2022	581	1074	54.10	[51.06; 57.11]
Brennan 2022	121	400	30.25	[25.78; 35.01]
**Random effects model**		**.**	**36.65**	**[24.38; 49.85]**

Heterogeneity: $I^2 = 98\%$, $\tau^2 = 0.0403$, $c^2_8 = 455.07$ ($p < 0.01$)

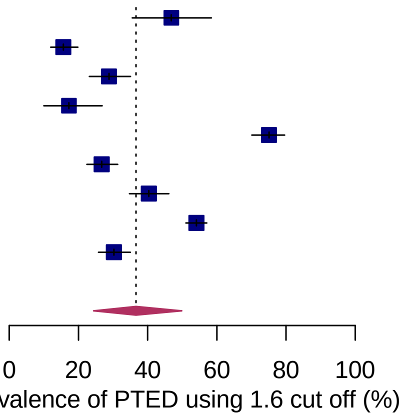

Supplementary figure 2. PTED prevalence using 2 cut off

Study	Cases	Total	Prevalence	95% C.I.
Dunn 2018	28	79	35.44	[25.00; 47.01]
Linden 2019	26	102	25.49	[17.38; 35.08]
Sabic 2018	9	87	10.34	[4.84; 18.73]
Katatuna 2014	74	397	18.64	[14.93; 22.83]
Sensky 2015	43	236	18.22	[13.51; 23.75]
Michailidis 2017	209	337	62.02	[56.60; 67.22]
Rubab 2022	77	300	25.67	[20.82; 31.00]
Brennan 2022	77	400	19.25	[15.50; 23.46]
**Random effects model**		**.**	**26.03**	**[16.09; 37.38]**

Heterogeneity: $I^2 = 97\%$, $\tau^2 = 0.0293$, $c^2_7 = 232.77$ ($p < 0.01$)

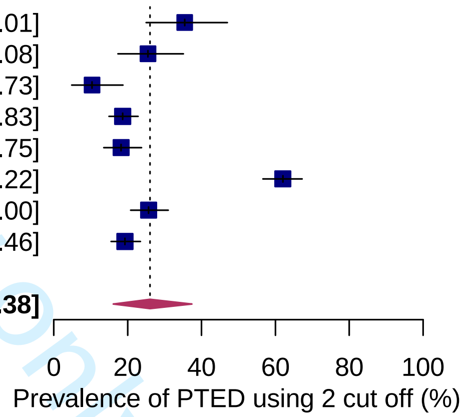

Supplementary figure 3. PTED prevalence using 2.5 cut off

Study	Cases	Total	Prevalence	95% C.I.
Sabic 2018	1	87	1.15	[0.03; 6.24]
Katatuna 2014	41	397	10.33	[7.51; 13.75]
Sensky 2015	21	236	8.90	[5.59; 13.28]
Michailidis 2017	152	337	45.10	[39.71; 50.59]
Rubab 2022	26	300	8.67	[5.74; 12.44]
Shin 2022	102	1074	9.50	[7.81; 11.41]
Brennan 2022	30	400	7.50	[5.12; 10.53]
**Random effects model**		**.**	**11.21**	**[4.02; 21.32]**

Heterogeneity: $I^2 = 97\%$, $\tau^2 = 0.0334$, $c^2_6 = 237.59$ ($p < 0.01$)

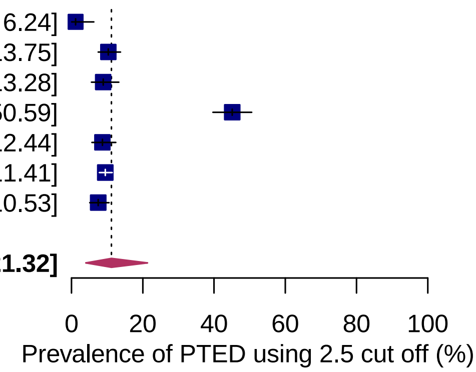

For peer review only - http://bmjopen.bmj.com/site/about/guidelines.xhtml

Supplementary figure 4. Any MIES items prevalence

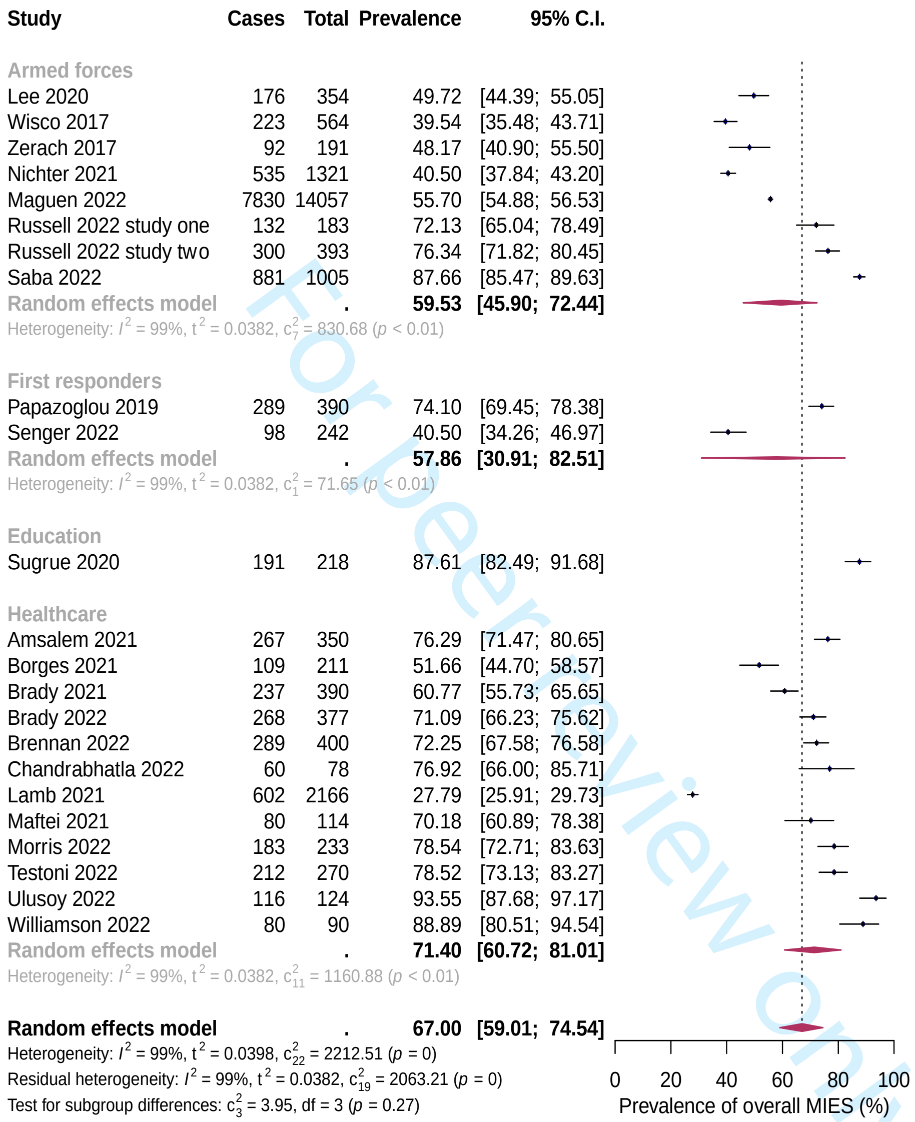

Supplementary figure 5. MIES perpetration prevalence

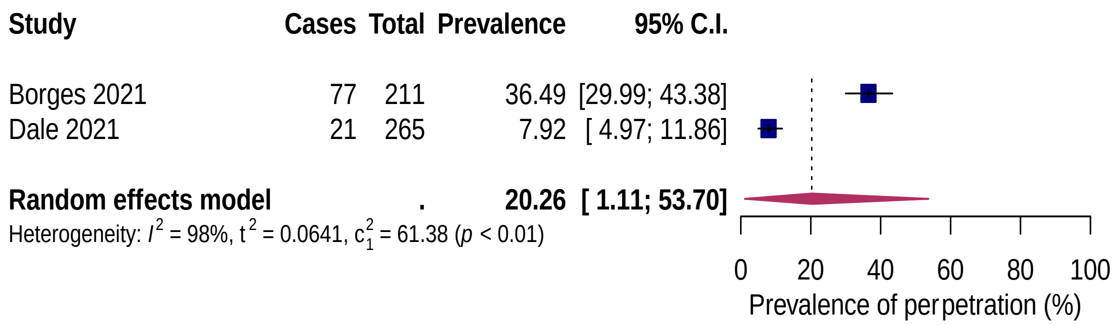

For peer review only - http://bmjopen.bmj.com/site/about/guidelines.xhtml

Supplementary figure 5. MIES transgressions-self prevalence

Study	Cases	Total	Prevalence	95% C.I.
**Armed forces**				
Bryan 2016	508	935	54.33	[51.08; 57.56]
Jordan 2017	214	867	24.68	[21.85; 27.69]
Lee 2020	115	354	32.49	[27.63; 37.64]
Wisco 2017	48	564	8.51	[6.34; 11.13]
Zerach 2017	42	191	21.99	[16.33; 28.54]
Richardson 2020	516	5227	9.87	[9.08; 10.71]
Nichter 2021	152	1321	11.51	[9.83; 13.35]
Maguen 2022	3064	14057	21.80	[21.12; 22.49]
Russell 2022 study one	97	183	53.01	[45.50; 60.41]
Russell 2022 study two	109	393	27.74	[23.36; 32.45]
Saba 2022	802	1005	79.80	[77.18; 82.24]
Levi−Belz 2022	44	190	23.16	[17.36; 29.81]
**Random effects model**		.	**29.30**	**[19.94; 39.64]**
Heterogeneity: I^2 = 100%, t^2 = 0.0364, c^2_{11} = 2850.04 (p = 0)				
**Education**				
Sugrue 2020	93	218	42.66	[36.01; 49.52]
**CPS**				
Haight 2017	9	38	23.68	[11.44; 40.24]
**Healthcare**				
Amsalem 2021	101	350	28.86	[24.16; 33.91]
Benatov 2022	94	296	31.76	[26.49; 37.39]
Brady 2021	92	390	23.59	[19.46; 28.12]
Brady 2022	92	377	24.40	[20.15; 29.06]
Brennan 2022	131	400	32.75	[28.17; 37.59]
Chandrabhatla 2022	23	78	29.49	[19.70; 40.89]
Dale 2021	61	265	23.02	[18.09; 28.56]
Maftei 2021	31	114	27.19	[19.28; 36.33]
Morris 2022	79	230	34.35	[28.23; 40.87]
Testoni 2022	78	270	28.89	[23.56; 34.69]
Ulusoy 2022	78	124	62.90	[53.77; 71.40]
Williamson 2022	51	90	56.67	[45.80; 67.08]
**Random effects model**		.	**33.16**	**[23.24; 43.90]**
Heterogeneity: I^2 = 90%, t^2 = 0.0364, c^2_{11} = 107.54 (p < 0.01)				
**First responders**				
Senger 2022	51	242	21.07	[16.11; 26.76]
**Random effects model**		.	**30.95**	**[24.72; 37.54]**

Heterogeneity: I^2 = 99%, t^2 = 0.0325, c^2_{26} = 3105.90 (p = 0)
Residual heterogeneity: I^2 = 99%, t^2 = 0.0364, c^2_{22} = 2957.58 (p = 0)
Test for subgroup differences: c^2_4 = 1.15, df = 4 (p = 0.89)

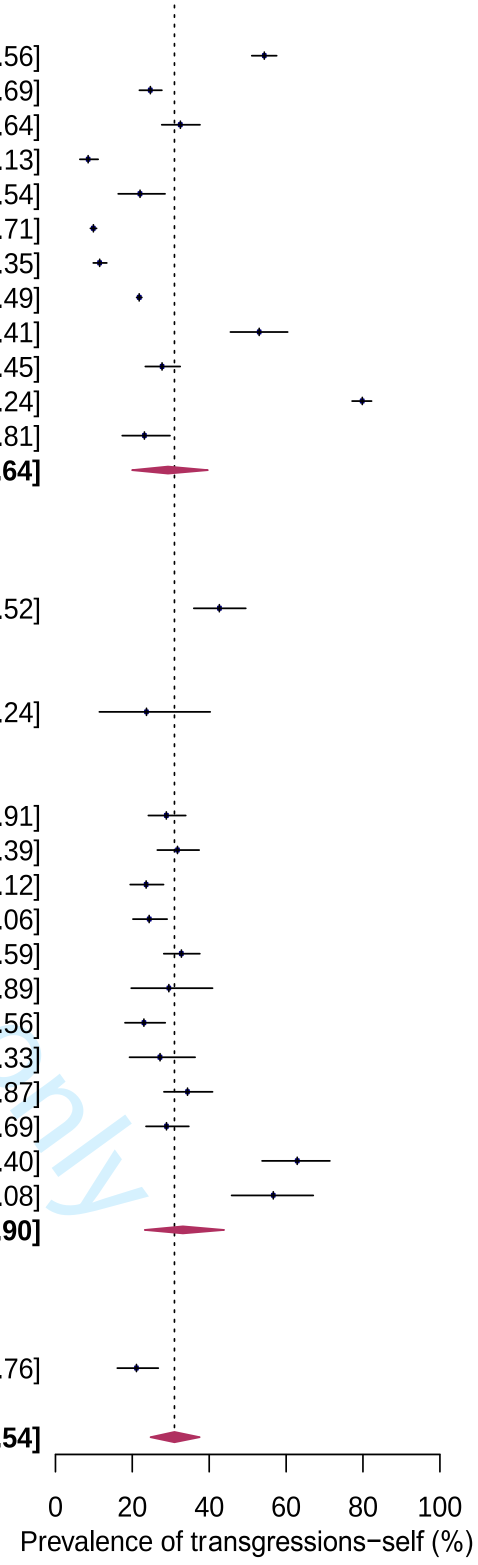

For peer review only

Supplementary figure 6. MIES transgressions-others prevalence

For peer review only - http://bmjopen.bmj.com/site/about/guidelines.xhtml

Study	Cases	Total	Prevalence	95% C.I.
**Armed forces**				
Bryan 2016	363	935	38.82	[35.69; 42.03]
Lee 2020	130	354	36.72	[31.69; 41.98]
Wisco 2017	126	564	22.34	[18.97; 26.01]
Zerach 2017	64	191	33.51	[26.86; 40.68]
Nichter 2021	316	1321	23.92	[21.64; 26.32]
Maguen 2022	6059	14057	43.10	[42.28; 43.93]
Russell 2022 study one	48	183	26.23	[20.02; 33.23]
Russell 2022 study two	62	393	15.78	[12.31; 19.76]
Saba 2022	303	1005	30.15	[27.32; 33.09]
Levi−Belz 2022	69	190	36.32	[29.48; 43.59]
**Random effects model**		.	**30.36**	**[20.07; 41.76]**
Heterogeneity: $I^2 = 98\%$, $t^2 = 0.0360$, $c_9^2 = 475.46$ ($p < 0.01$)				
**Education**				
Sugrue 2020	175	218	80.28	[74.37; 85.34]
**CPS**				
Haight 2017	35	38	92.11	[78.62; 98.34]
**Healthcare**				
Amsalem 2021	215	350	61.43	[56.11; 66.56]
Benatov 2022	146	296	49.32	[43.49; 55.17]
Brady 2021	189	390	48.46	[43.40; 53.55]
Brady 2022	197	377	52.25	[47.08; 57.39]
Brennan 2022	213	400	53.25	[48.23; 58.22]
Chandrabhatla 2022	49	77	63.64	[51.88; 74.30]
Dale 2021	6	265	2.26	[0.84; 4.86]
Maftei 2021	61	114	53.51	[43.93; 62.90]
Morris 2022	154	230	66.96	[60.47; 73.00]
Testoni 2022	166	270	61.48	[55.39; 67.32]
Ulusoy 2022	109	124	87.90	[80.83; 93.07]
Williamson 2022	63	90	70.00	[59.43; 79.21]
**Random effects model**		.	**54.88**	**[43.91; 65.62]**
Heterogeneity: $I^2 = 98\%$, $t^2 = 0.0360$, $c_{11}^2 = 591.65$ ($p < 0.01$)				
**First responders**				
Senger 2022	147	242	60.74	[54.28; 66.94]
**Random effects model**		.	**47.73**	**[38.04; 57.51]**

Heterogeneity: $I^2 = 98\%$, $t^2 = 0.0607$, $c_{24}^2 = 1464.59$ ($p < 0.01$)
Residual heterogeneity: $I^2 = 98\%$, $t^2 = 0.0360$, $c_{20}^2 = 1067.12$ ($p < 0.01$)
Test for subgroup differences: $c_4^2 = 20.01$, df = 4 ($p < 0.01$)

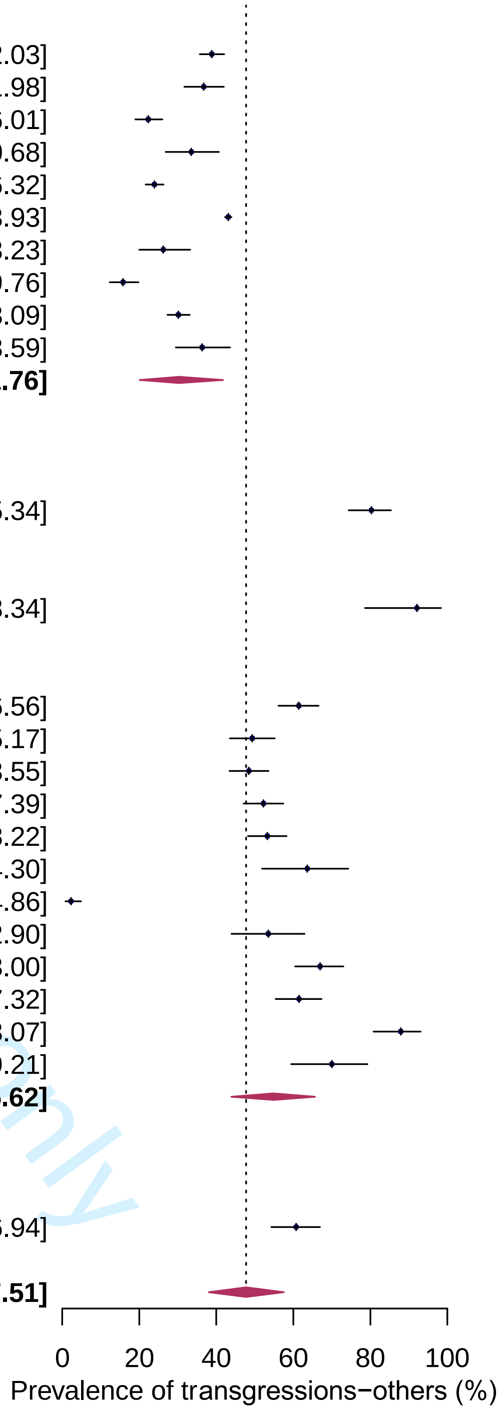

Supplementary figure 7. MIES betrayal prevalence

For peer review only

Study	Cases	Total	Prevalence	95% C.I.
**Armed forces**				
Bryan 2016	540	935	57.75	[54.51; 60.94]
Jordan 2017	252	867	29.07	[26.06; 32.21]
Lee 2020	121	354	34.18	[29.25; 39.38]
Wisco 2017	142	564	25.18	[21.64; 28.97]
Zerach 2017	59	191	30.89	[24.42; 37.97]
Nichter 2021	189	1321	14.31	[12.46; 16.31]
Lamb 2021	439	2166	20.27	[18.59; 22.02]
Maguen 2022	5651	14057	40.20	[39.39; 41.02]
Russell 2022 study one	99	183	54.10	[46.59; 61.47]
Russell 2022 study two	112	393	28.50	[24.09; 33.24]
Saba 2022	300	1005	29.85	[27.03; 32.79]
Levi−Belz 2022	67	190	35.26	[28.49; 42.51]
Random effects model		.	**32.67**	**[26.29; 39.38]**
Heterogeneity: $I^2 = 99\%$, $t^2 = 0.0147$, $c^2_{11} = 1001.5$ ($p < 0.01$)				
**First responders**				
Papazoglou 2019	250	390	64.10	[59.12; 68.87]
Senger 2022	97	242	40.08	[33.86; 46.55]
Random effects model		.	**52.32**	**[35.23; 69.13]**
Heterogeneity: $I^2 = 97\%$, $t^2 = 0.0147$, $c^2_{1} = 35.05$ ($p < 0.01$)				
**Education**				
Sugrue 2020	148	218	67.89	[61.25; 74.04]
**CPS**				
Haight 2017	12	38	31.58	[17.50; 48.65]
**Healthcare**				
Amsalem 2021	205	350	58.57	[53.21; 63.78]
Benatov 2022	184	296	62.16	[56.37; 67.71]
Borges 2021	88	211	41.71	[34.98; 48.67]
Brady 2021	166	390	42.56	[37.60; 47.64]
Brady 2022	210	377	55.70	[50.53; 60.79]
Brennan 2022	227	400	56.75	[51.74; 61.66]
Chandrabhatla 2022	37	78	47.44	[36.01; 59.07]
Maftei 2021	66	114	57.89	[48.29; 67.08]
Morris 2022	154	233	66.09	[59.63; 72.15]
Testoni 2022	166	270	61.48	[55.39; 67.32]
Ulusoy 2022	96	124	77.42	[69.04; 84.44]
Williamson 2022	62	90	68.89	[58.26; 78.23]
Random effects model		.	**58.12**	**[50.96; 65.12]**
Heterogeneity: $I^2 = 89\%$, $t^2 = 0.0147$, $c^2_{11} = 98.31$ ($p < 0.01$)				
**Random effects model**		**.**	**46.03**	**[39.54; 52.59]**

Heterogeneity: $I^2 = 98\%$, $t^2 = 0.0300$, $c^2_{27} = 1764.34$ ($p = 0$)
Residual heterogeneity: $I^2 = 98\%$, $t^2 = 0.0147$, $c^2_{23} = 1134.87$ ($p < 0.01$)
Test for subgroup differences: $c^2_4 = 30.29$, df = 4 ($p < 0.01$)

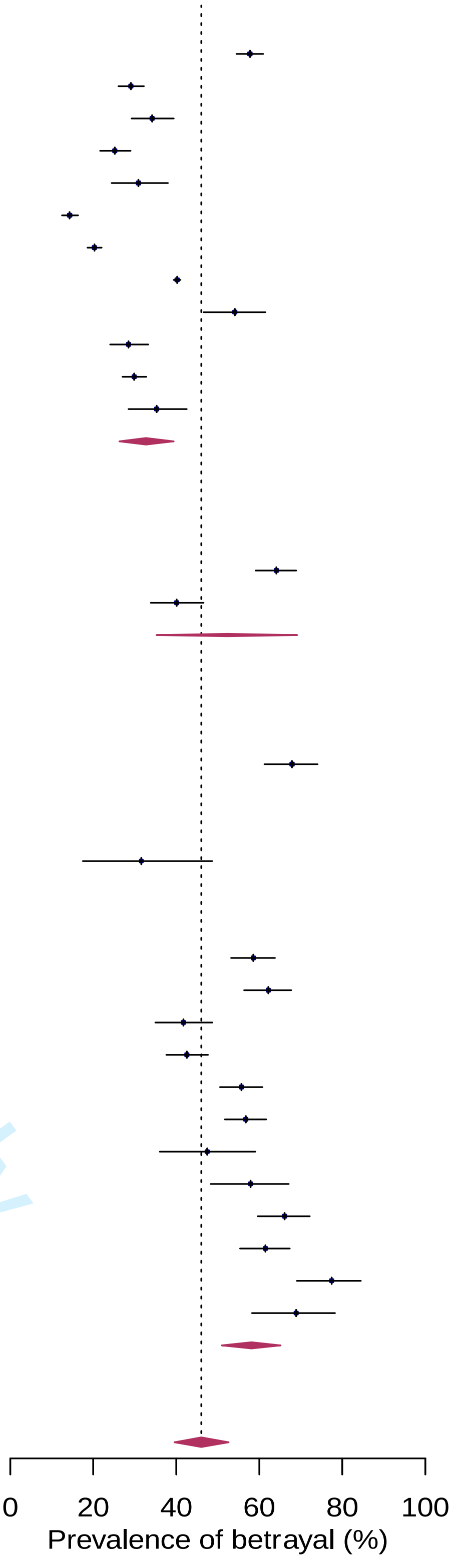

Supplementary figure 8. MISS-HP prevalence

For peer review only

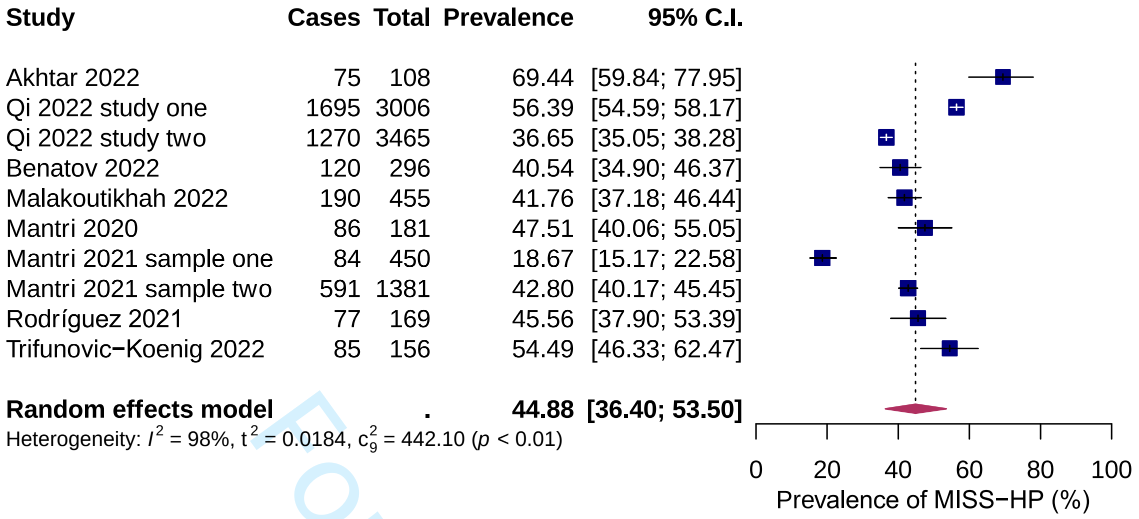

*Analyses using **pooled mean** estimates for PTED and moral injury, split by occupation*

Supplementary figure 9. PTED mean

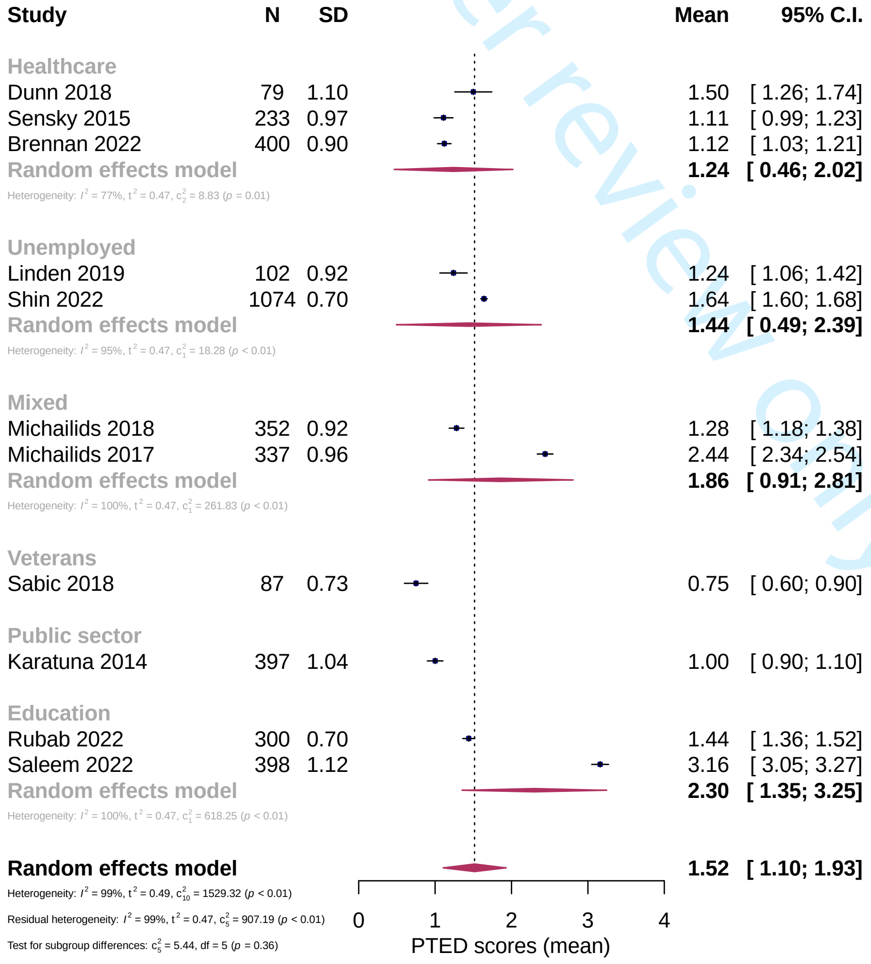

Supplementary figure 10: Overall MIES mean

For peer review only - http://bmjopen.bmj.com/site/about/guidelines.xhtml

For peer review only

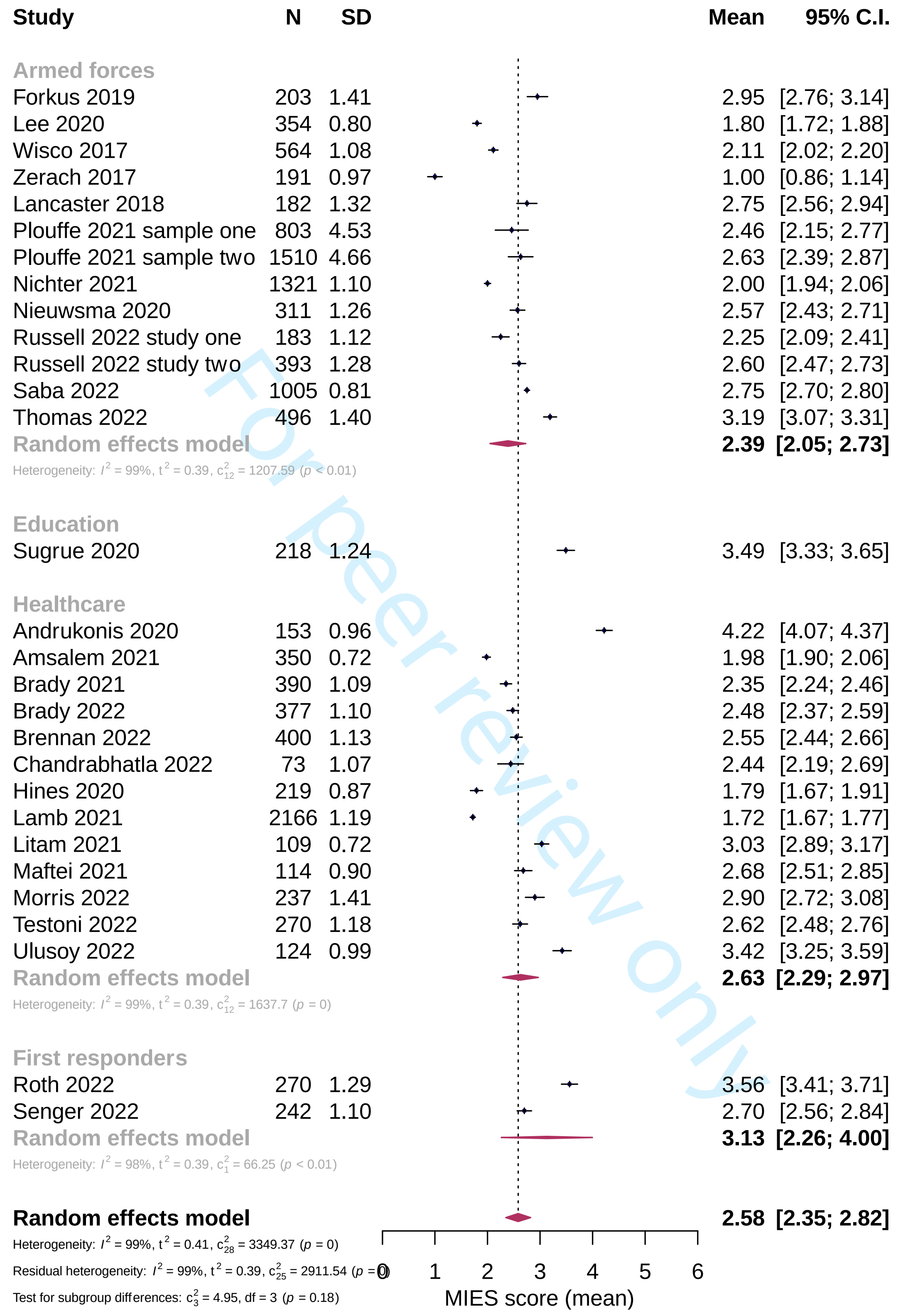

Supplementary figure 11: MIES perpetration mean

For peer review only - http://bmjopen.bmj.com/site/about/guidelines.xhtml

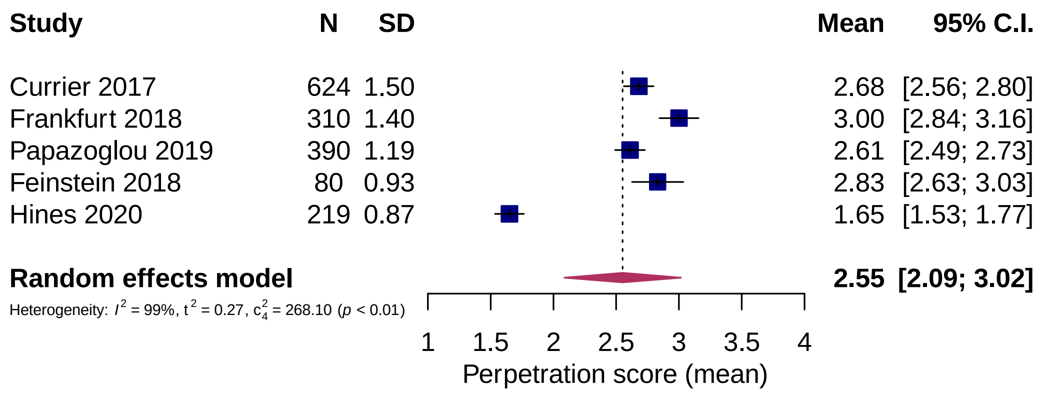

<u>Supplementary figure 12: MIES transgressions-self mean</u>

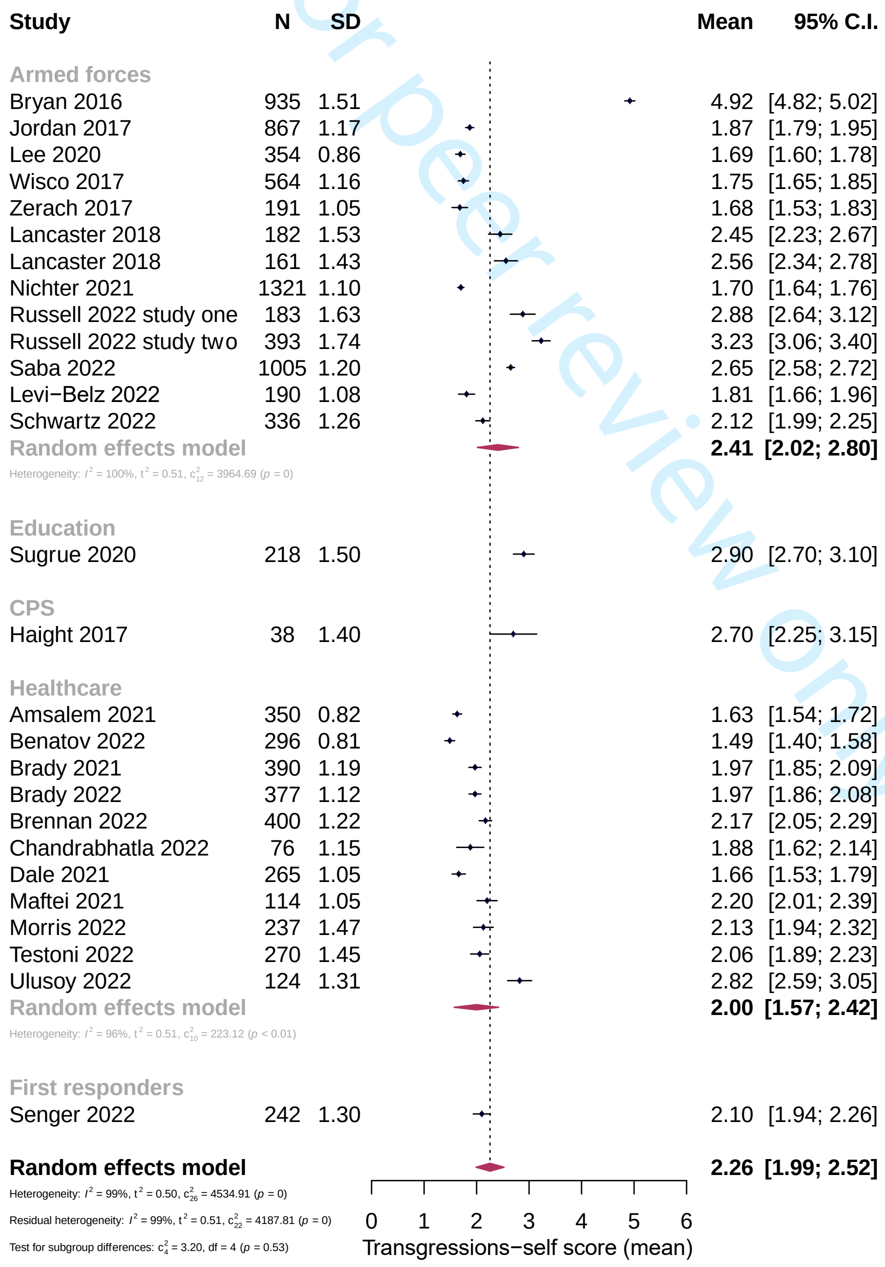

For peer review only

Supplementary figure 13: MIES transgressions-others mean

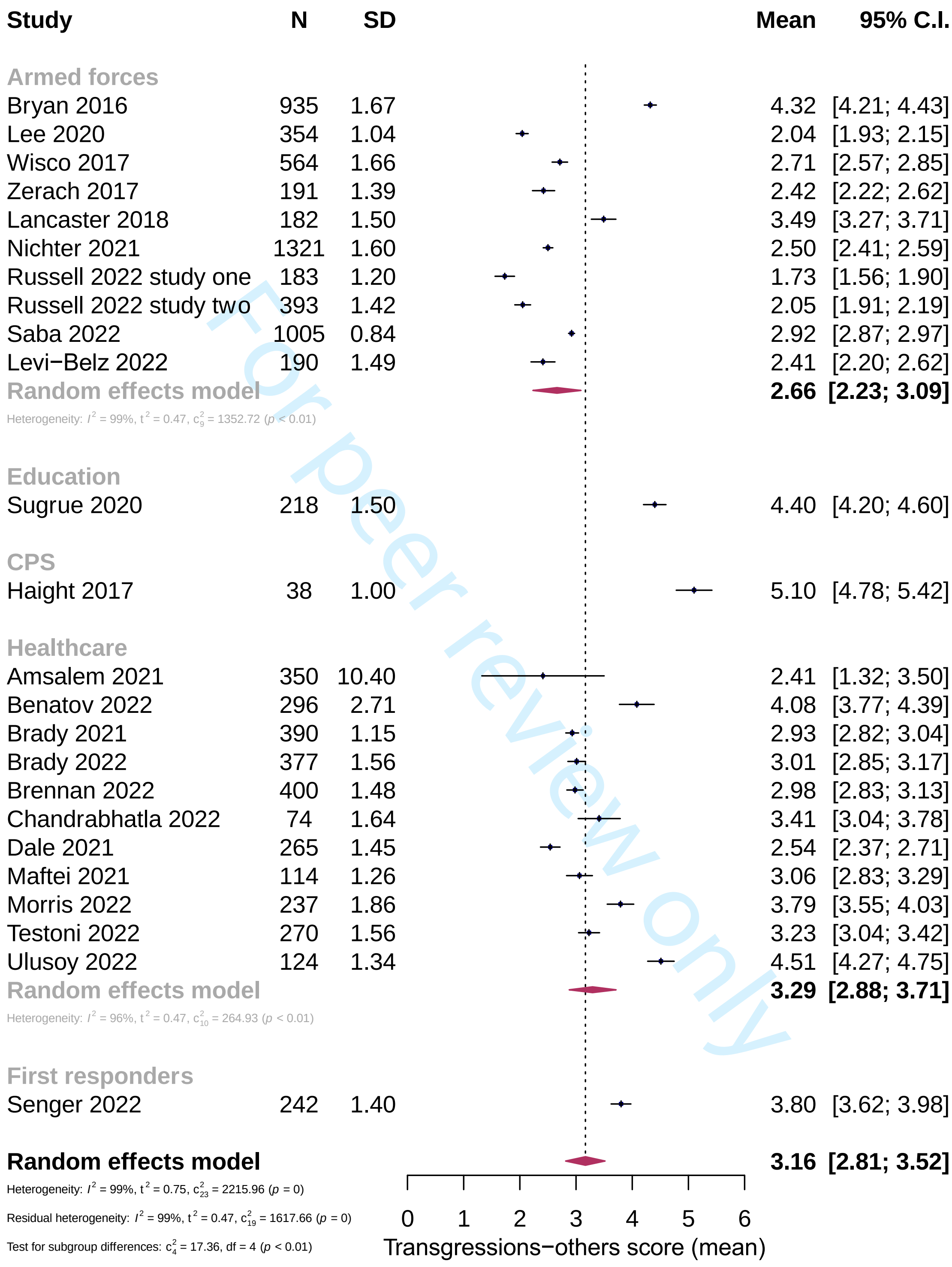

Supplementary figure 14: MIES betrayal mean

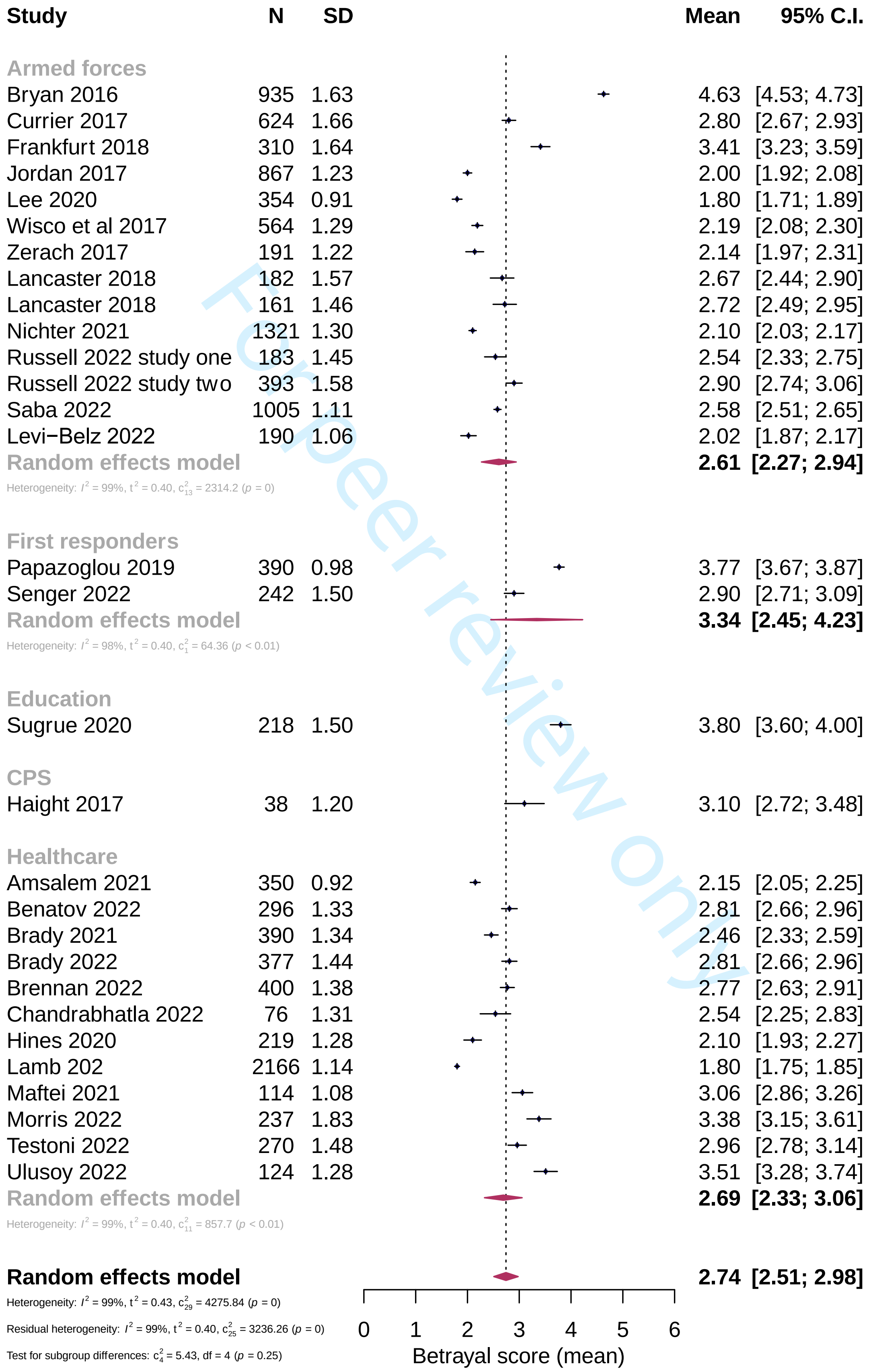

Supplementary figure 15: MIQ-M total mean

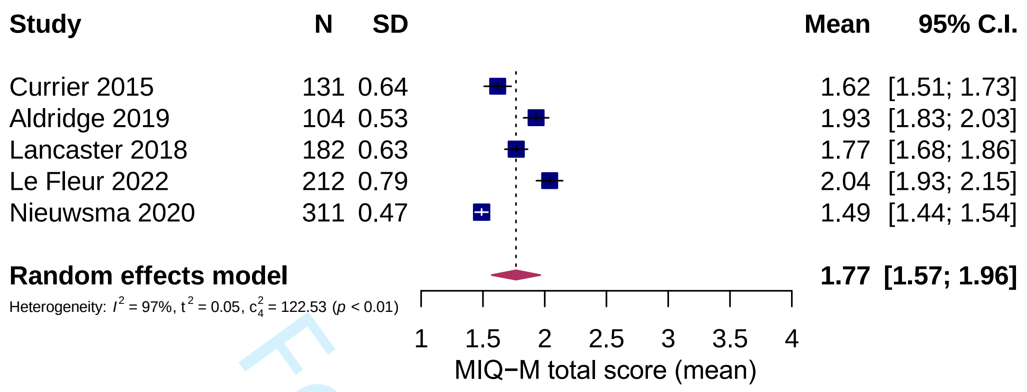

MIQ-M causes

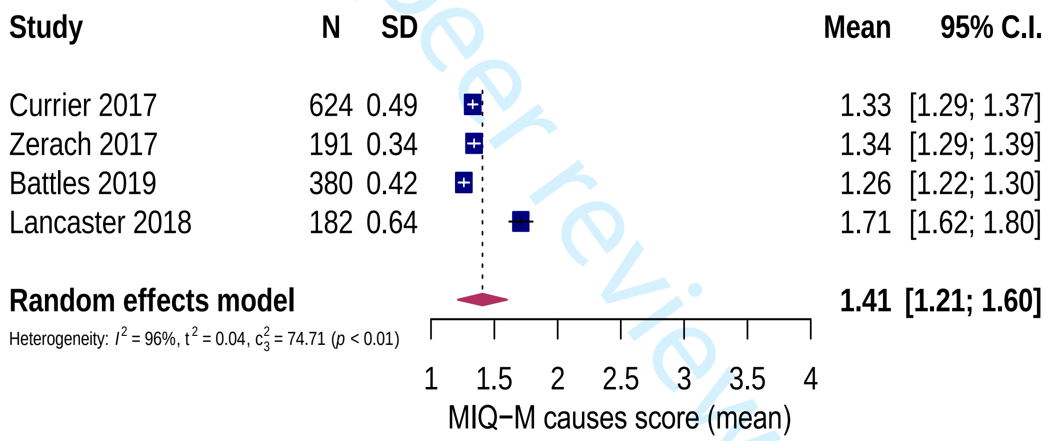

Supplementary figure 16: EMIS total mean

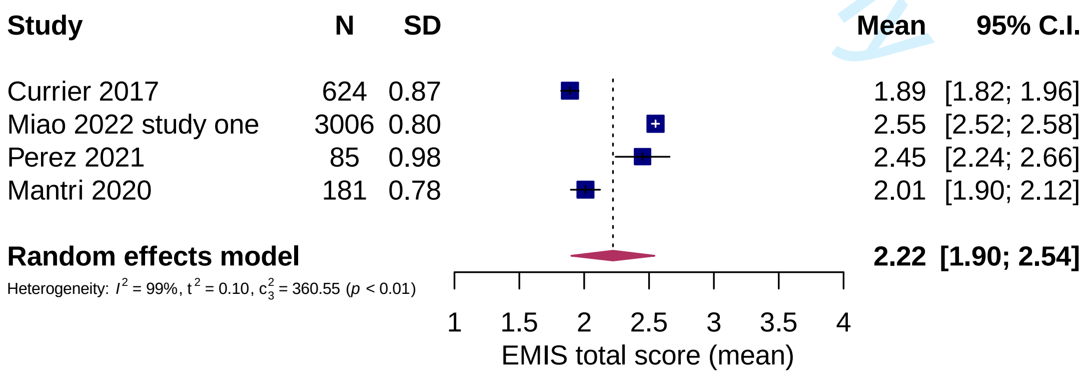

For peer review only - http://bmjopen.bmj.com/site/about/guidelines.xhtml

Supplementary figure 17: EMIS self mean

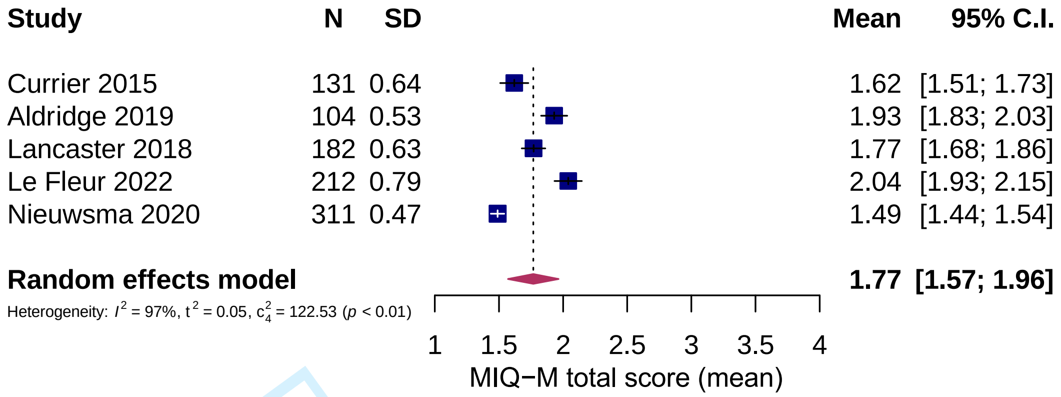

EMIS other

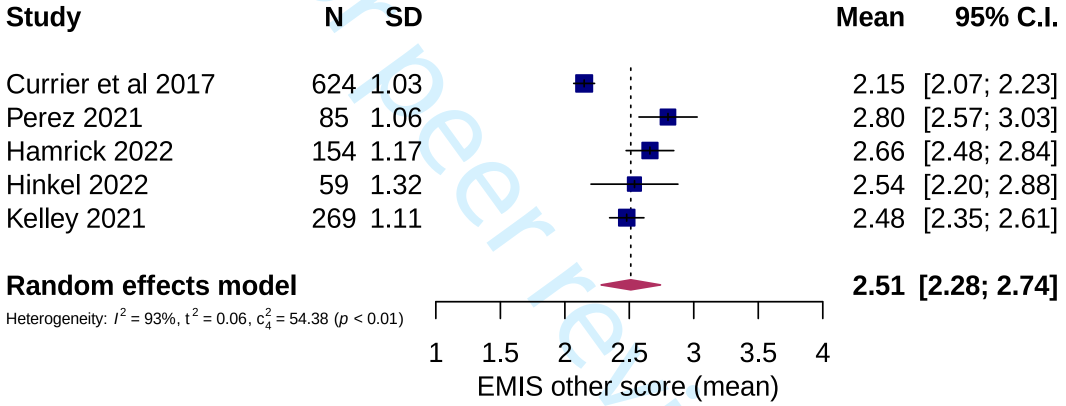

Supplementary figure 18: MISS-HP mean

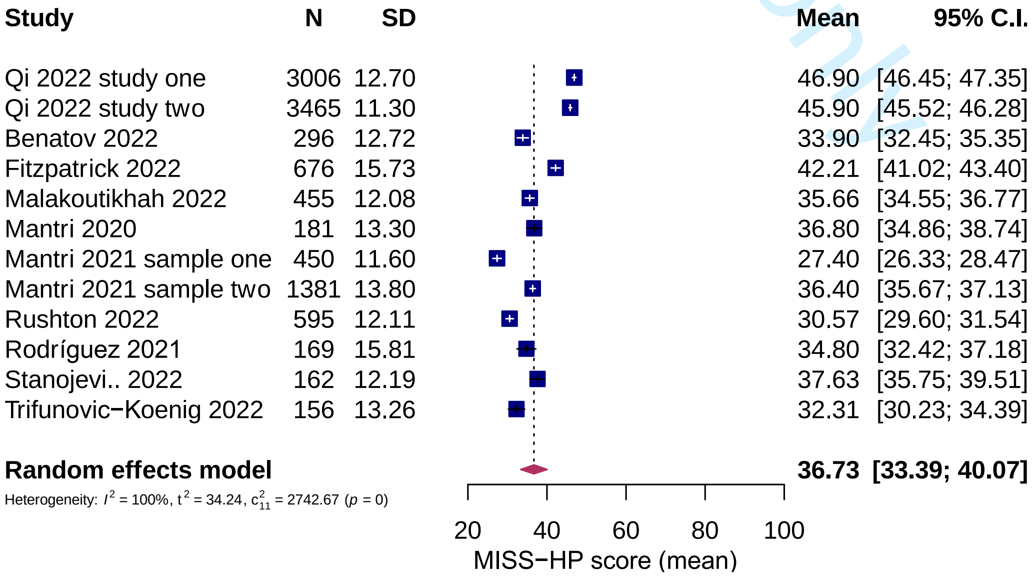

For peer review only

Supplementary figure 19: MIOS total mean

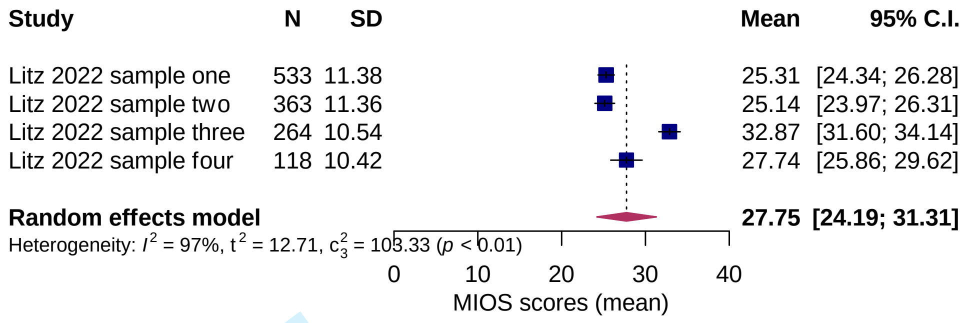

Supplementary figure 20: MIOS shame mean

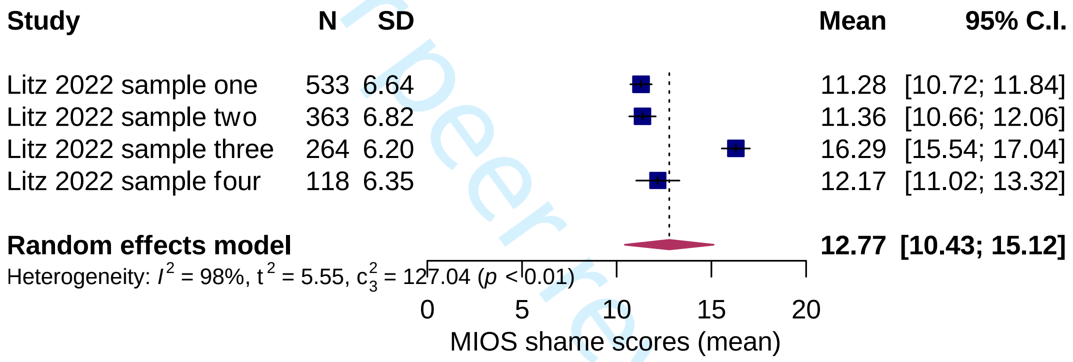

Supplementary figure 21: MIOS trust mean

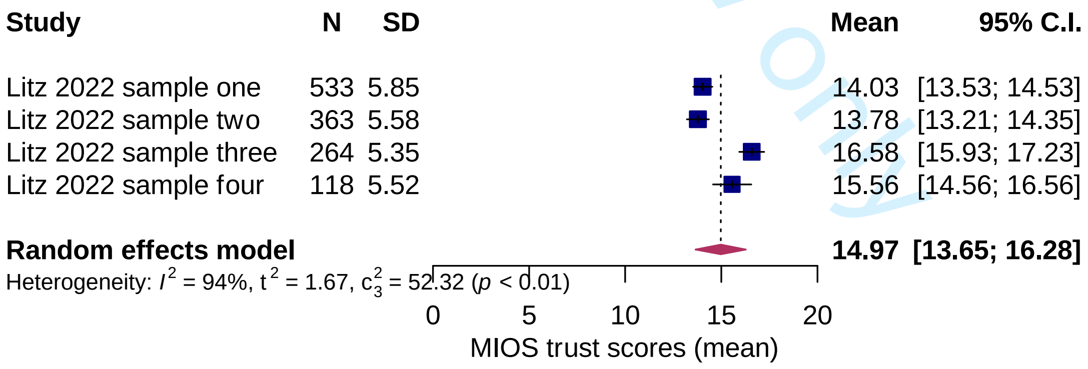

For peer review only

Supplementary table 3. Subgroup analyses based on study quality (mean and prevalence)

Measure	Medium risk			Low risk			
	k	Pooled mean (95% CI)	I2	k	Pooled mean (95% CI)	I2	P value
PTED	10	1.56 (1.11-2.01)	99%	1	1.12 (1.03-1.21)	-	0.566
MIES total	24	2.67 (2.42-2.92)	99%	5	2.18 (1.62-2.73)	97%	0.114
MIES transgressions-self	25	2.30 (2.02-2.58)	99%	2	2.17 (0.75-2.70)	0%	0.267
MIES transgressions-others	22	3.22 (2.85-3.58)	99%	2	2.60 (1.40-3.81)	85%	0.342
MIES betrayal	27	2.83 (2.60-3.06)	99%	3	2.03 (1.33-2.73)	97%	0.034
	k	Pooled prevalence (95% CI)	I2	k	Pooled prevalence (95% CI)	I2	P value
MIES any	18	72.30 (64.83-79.20)	97%	5	47.06 (32.42-61.92)	99%	0.003
Transgressions-self	22	34.92 (28.32-41.82)	99%	5	15.94 (84.7-25.21	98%	0.006
Transgressions-others	21	50.23 (39.64-60.81)	98%	4	35.11 (14.67-58.94)	99%	0.254
Betrayal	23	49.69 (43.09-56.29)	97%	5	30.33 (18.51-43.66)	99%	0.011

For peer review only - http://bmjopen.bmj.com/site/about/guidelines.xhtml

Supplementary table 4. Subgroup analyses based on cut off value (prevalence)

Measure	Slightly agree		Moderately agree		Strongly agree		Agree		NR[a]		
	k	Pooled %	*k*	Pooled %	*k*	Pooled %	*k*	Pooled %	*k*	Pooled %	P value
MIES overall	14	72.47 (63.07 to 80.98)	6	54.13 (38.88 to 69)		N/A	2	73.36 (47.45 to 92.77)	1	49.72 (15.70 to 83.88)	0.152
Transgressions self	20	31.93 (24.52 to 39.81)	3	22.06 (74.1 to 41.63)	1	54.33 (20.26 to 86.24)	2	28.04 (84.7 to 53.41)	1	23.02(01.68 to 58.11)	0.578
Transgressions others	18	52.22 (41.78 to 62.56)	3	37.46 (15.33 to 62.77)	1	38.82 (53.39 to 80.92)	2	57.56 (27 to 85.26)	1	02.26 (0 to 31.56)	0.057
Betrayal	19	46.87 (38.98 to 54.83)	6	37.54 (24.56 to 51.49)	1	57.75 (24.89 to 87.16)	1	58.24 (33.89 to 80.65)		-	0.409
Measure	28.5		36		36.5						
	k	Pooled %	*k*	Pooled %	*k*	Pooled %					P value
MISS-HP	1	54.49 (46.61 to 62.25)	8	44.17 (33.84 to 54.77)	1	41.76 (37.26 to 46.33)					0.802

[a] For MIES any this was "sometimes". MIES; Moral Injury Events Scale, MISS-HP; Moral Injury Symptom Scale-Healthcare Professionals

For peer review only

For peer review only - http://bmjopen.bmj.com/site/about/guidelines.xhtml

Supplementary table 5. Prevalence and norms for moral injury scales that were not combined for narrative synthesis (*k*=1)

Measure	Mean(SD)	Measure	Prevalence (%)
MIES-other (items 1-2, 7-9)	2.51 (1.40)	MIES-other (items 1-2, 7-9)	19.76%
MIES-commission (items 1-4)	1.71 (1.06)	MIES-commission (items 1-4)	14.28%
MIES-omission (items 5-6)	1.47 (1.1)	MIES-omission (items 5-6)	4.57%
MIQ-T	1.16 (0.56)	MIES-no moral distress	42%
MIQ-M modified		MIES-moral distress-other	19%
MIQ-M modified AoW	1.77 (0.63)	MIES-witnessing-only	16%
MIQ-M modified PCoW	1.99 (0.66)	MIES-moral distress-self	8%
MIQ-M modified LFB	2.12 (0.78)	MIES-moral distress-self and other	15%
MIQ-M modified AoW defining characteristics	2.21 (0.67)	EMIS-M self-directed ≥2.5 score <3.5	10.14%
MIQ-M modified PCoW defining characteristics	2.14 (0.66)	EMIS-M other-directed ≥2.5 score <3.5	29.19%
MIQ-M modified LFB defining characteristics	2.12 (0.67)		
BMIS total	3.71 (4.38)	BMIS event	44.1%
MIA-PSP total	59.6 (21.9)		
MIA-PSP emotional sequalae	25 (9.7)		

For peer review only - http://bmjopen.bmj.com/site/about/guidelines.xhtml

MIA-PSP perpetration	15 (8.5)
MIA-PSP betrayal	19.6 (7.7)
Toronto total	1.5 (1.27)
Toronto organisation	1.54 (1.32)
Toronto individual	1.18 (1.19)
Toronto online	1.68 (1.27)

PTED; post-traumatic embitterment disorder, MIES; moral injury events scale; MIES; Moral injury events scales, EMIS-M; Expressions of Moral Injury Scale-Military Version; MIA-PSP; Moral Injury Assessment for Public Safety Personnel, MIQ-M; Moral Injury Questionnaire—Military version, BMIS; Brief Moral Injury Screen, MIQ-T; Moral Injury Questionnaire—Teacher version, MIQ-M; Moral Injury Questionnaire—Military version; AoW; atrocities of war, LFB; leadership failure or betrayal, PCOW; psychological consequences of war

For peer review only

Appendix A. Data extraction table for included studies

Author (Year)	Study Design	N_a	Occupation	Sampling method	Mean age	% Male	% White	Location	RR	Measurement tool	Associations<br>(+) significant positive correlation<br>(-) significant negative correlation<br>(0) not significant	Prevalence<br>N (%)	Total M(SD)	Hoy	Conflict of Interest
PTED only studies															
Dunn & Sensky. 2018	Cross-sectional	79	Healthcare NHS staff.	Convenience<br>Occupational health department October 2015-March 2016.	44 (12)	33%	NR	UK	NR	PTED scale 1.6+ and 2+	(Mental) health<br>Depression (HADS) (+)<br>Individual and/or work characteristics<br>Affective rumination (WRRQ) (+)<br>Detachment (WRRQ) (-)<br>Problem solving pondering (WRRQ) (0)<br>Positive beliefs about rumination (PBRS) (0)<br>Sickness absence (0)<br>Demographics<br>Gender (0)	1.6 cut off: 37 (47%)<br>2 cut off: 28(35%)	1.5 (1.1)	5	NR

For peer review only

Linden & Rotter 2019	Cross-sectional	102	Unemployed<br>Duration > 1 year (32.4%)	Convenience	36.6 (12.1)	59%	NR	Germany	NR	PTED scale 2+	(Mental) health<br>Psychological distress (GHQ-28) (+)<br>Individual and/or work characteristics<br>Feelings of injustice (+)<br>Worse appraisal of unemployment (+)<br>Duration of unemployment (+)<br>Demographics<br>Gender (0)<br>Age (+)	Cut off 2: 26 (25.5%)	1.24 (0.92)	5	None
Michailidis & Cropley. 2018	Longitudinal (T1)	352	Multiple.	Convenience	NR	NR	NR	Cyprus	NR	PTED scale 1.6+	(Mental) health<br>Negative affect (+)<br>Individual and/or work characteristics<br>Organisational procedural justice (OJP) (-)	Cut off 1.6: 55 (35%)	1.28 (0.92)	5	None

For peer review only

											Organisational distributive justice (OJP) (-)<br>Organisational interpersonal justice (OJP) (-)<br>Organisational informational justice (OJP) (-)<br>Supervisory control (+)<br>Job demands (0)				
Sensky, Salimu, Ballard & Pereira. 2015	Cross sectional	236	NHS staff, primarily nurses (N = 67, 28%) and healthcare assistants (n = 49, 21%).<br>45% worked for the trust for <5 years.	Convenience<br>September-November 2009<br>Represents sample frame.	NR	45%	NR	UK.	NR	PTED scale 1.6+	Individual and/or work characteristics<br>Work attitudes - felt obligation (0)<br>Work attitudes - organisational support (-)<br>Work attitudes - employer-employee reciprocity (0)<br>Work attitudes - work effort (0)<br>Work attitudes - procedural justice (-) Management referral to occupational health (+)	Cut off 1.6: 68 (28.81%)<br>Cut off 2: 43 (18.2%)<br>Cut off 2.5: 21 (8.9%)	1.11(0.97)	4	None

For peer review only

Certified sickness absence (+)

Uncertified sickness absence (+)

Work stress - demands (HSE) (0)

Work stress - control (HSE) (0)

Work stress - management support (HSE) (-)

Work stress - peer support (HSE) (-)

Work stress – relationships (HSE) (0)

Work stress - role (HSE) (-)

Work stress – change (HSE) (-)

Demographics:

Gender (0)

Age (0)

Years since qualifying (0)

More years in present job (+)

For peer review only

Sabic, Sabic, & Baltic-Mujanovic, 2018	Cross sectional	87	War veterans of the war in Bosnia and Herzegovnia (1992-1995).<br>Average time since they were a soldier M = 23.86 years.	Convenience	51.42 (5.98)	100%	NR	Bosnia and Herzegovina	NR	PTED scale 1.6+, 2+ and 2.5+	NR	Cut off 1.6: 15 (17.24%)<br>Cut off 2:<br>9 (10.34%)<br>Cut off 2.5:<br>1 (1.15%)	0.75(0.73)	5	None
Michailidis & Cropley. 2017	Cross sectional	337	Multiple	Convenience	36.87 (12.4)	26.1 %	NR	NR	NR	PTED scale 1.6+, 2+ and 2.5+	(Mental) health<br>Negative affect (PANAS) (+)<br>Positive affect (PANAS) (-)<br>Individual and/or work characteristics<br>Affective rumination (WRRQ) (+)<br>Problem solving pondering (WRRQ) (+)<br>Detachment (WRRQ) (-)	Cut off 1.6: 253 (75%)<br>Cut off 2: 209 (62%)<br>Cut off 2.5: 152 (45%)	2.44(.96)	5	None

For peer review only

Job demands (JCQ) (0)

Workplace social support (-)

Organisational procedural justice (OJP) (-)

Organisational distributive justice (OJP) (-)

Organisational interpersonal justice (OJP) (-)

Organisational informational justice (OJP) (-)

Supervisory control (WCOWP) (+)

Demographics

Gender (+)

Age (0)

Dependent children (0)

Hours worked per week (+)

For peer review only

For peer review only - http://bmjopen.bmj.com/site/about/guidelines.xhtml

											Years in current job (+)				
Karatuna & Gok 2014	Cross-sectional	397	Public sector, social security institutions<br>Non-managerial position = 83.1%	Convenience	NR	57.2 %		Turkey	39.11%	PTED scale 1.6+, 2+, 2.5+	Individual and/or work characteristics<br>Workplace bulling (NAQ-R) (+)<br>Victimisation from bullying (+)<br>Victims of bulling (+)	Cut off 1.6: 106 (26.7%)<br>Cut off 2: 74 (18.6%)<br>Cut off 2.5: 41 (10.3%)	1.00(1.04)	5	NR
Rubab & Tariq 2022	Cross-sectional	300	Private school teachers.	Purposive.	31 (7.9)	50%	NR	NR	NR	PTED scale 1.6+, 2+, 2.5+	(Mental) health<br>Depression (DASS-21) (+)<br>Stress (DASS-21) (+)<br>Anxiety (DASS-21) (+)<br>Depression, stress, and anxiety total (DASS-21) (+)<br>Individual and/or work characteristics	Cut off 1.6: 121 (40.3%)<br>Cut off 2: 77 (25.7%)<br>Cut off 2.5: 26 (8.7%)	1.44 (0.70)	5	NR

For peer review only

											Generalised self-efficacy (GSES) (-)				
Saleem, Bashir & Abrar, 2022	Longitudinal (T2)	398	Public sector university academics.	Simple random sampling.	44.5	64%	NR	Pakistan	73 %	PTED scale (mean only)	(Mental) health<br>Employee well-being (-)<br>Individual and/or work characteristics<br>Leaders' ethical behaviours (-)<br>Employee core self-evaluation (-)	NR	3.16 (1.12)	5	None
Shin & You 2022	Cross-sectional	1074	Unemployed.	Convenience	47.1 (13.6)	47.6 %	NR	South Korea	NR	PTED scale 1.6+, >1.6 ≥ 2, 2.5+	Individual and/or work characteristics<br>Work types (0)<br>Unemployment duration (0)<br>Unemployment route (0)<br>Negative life events (+)<br>General belief in a just world (BJW) (-)	Cut off 1.6: 581 (54.1%)<br>Cut off 1.6 - 2.5: 479 (44.6%)<br>Cut off 2.5: 102 (9.5%)	1.64 (0.70)	5	None

For peer review only - http://bmjopen.bmj.com/site/about/guidelines.xhtml

For peer review only

											Personal BJW (-)<br>Demographics<br>Gender (0)<br>Age (+)<br>Marital status (divorced/separated) (+)<br>Educational level (-)<br>Income (-)				
PTED and moral injury study															
Brennan, McKay & Cole, 2022	Longitudinal (T1)	400	Health and social care<br>Allied health (14.5%)<br>Primary care practitioner (15.8%)<br>Managerial (10.3%)<br>Health info/science (9.3%)<br>Psychological (6.8%)	Convenience<br>September-October 2020.	36.84 (10.7)	24.5 %	63%	UK	89.5 %	MIES 4+ (slightly agree)<br>3 subscales (transgressions-others, transgressions-self, betrayal)<br>PTED 1.6+ and 2+ and 2.5	Transgressions-self<br>(Mental) health:<br>Current mental health diagnoses (0)<br>Individual and/or work characteristics<br>Exposure to occupational stressors (+)<br>Pessimism (LOTR) (+)<br>Consideration of immediate consequences (CFC-14) (+)	MIES:<br>Overall %<br>289 (72.3%)<br>Per subscale %<br>Transgressions-others: 213 (53.3%)<br>Transgressions-self: 131 (32.8%)<br>Betrayal: 227 (56.8%)	MIES:<br>Overall M/SD<br>2.55(1.13)<br>Subscale M/SD<br>Transgressions-others: 2.98(1.48)<br>Transgressions-self: 2.17(1.22)<br>Betrayal: 2.77(1.38)	2	ESRC and University funding

For peer review only - http://bmjopen.bmj.com/site/about/guidelines.xhtml

Corporate (16.3%)<br><br>Clinical support (26.3%)	Consideration of future consequences (CFC-14) (0)<br><br>Resilient coping style (BRCS) (0)<br><br>Self-esteem (RSES) (-)<br><br>Procedural justice (BJW) (-)<br><br>Distributive justice (BJW) (0)<br><br>Social desirability (-)<br><br>Demographics *<br><br>Age (0)<br><br>Gender (0)<br><br>Professional role (0)<br><br>Ethnicity (0)<br><br>Years in current role (0)<br><br>Transgressions-others<br><br>(Mental) health:<br><br>Current mental health diagnoses (0)<br><br>Individual and/or work characteristics	MIES per item %:<br><br>Item 1: 179 (44.8%)<br><br>Item 2: 167 (41.8%)<br><br>Item 3: 76 (19%)<br><br>Item 4: 94 (23.5%)<br><br>Item 5: 83 (20.8%)<br><br>Item 6: 78 (19.5%)<br><br>Item 7: 161 (40.3%)<br><br>Item 8: 124 (31%)<br><br>Item 9: 158 (39.5%)<br><br>PTED overall %:<br><br>1.6 cut off: 121 (30.3%)<br><br>2 cut off: 77 (19.3%)	PTED: Overall M/SD<br><br>1.12(0.90)

For peer review only

For peer review only - http://bmjopen.bmj.com/site/about/guidelines.xhtml

Exposure to occupational stressors (+)	2.5 cut off: 30 (7.5%)
Pessimism (LOTR) (+)	
Consideration of immediate consequences (CFC-14) (0)	
Consideration of future consequences (CFC-14) (0)	
Resilient coping style (BRCS) (+)	
Self-esteem (RSES) (-)	
Procedural justice (BJW) (-)	
Distributive justice (BJW) (0)	
Social desirability (-)	
Betrayal:	
Mental health:	
Current mental health diagnoses (+)	
Individual and/or work characteristics	

For peer review only - http://bmjopen.bmj.com/site/about/guidelines.xhtml

Exposure to occupational stressors (+)

Pessimism (LOTR) (+)

Consideration of immediate consequences (CFC-14) (+)

Consideration of future consequences (CFC-14) (0)

Resilient coping style (BRCS) (0)

Self-esteem (RSES) (-)

Procedural justice (BJW) (-)

Distributive justice (BJW) (-)

Optimism (LOTR) (-)

Social desirability (-)

PTED:

Mental health:

Current mental health diagnoses (+)

For peer review only - http://bmjopen.bmj.com/site/about/guidelines.xhtml

Individual and/or work characteristics

Exposure to occupational stressors (+)

Pessimism (LOTR) (+)

Consideration of immediate consequences (CFC-14) (+)

Consideration of future consequences (CFC-14) (0)

Resilient coping style (BRCS) (0)

Self-esteem (R-SES) (-)

Procedural justice (BJW) (-)

Distributive justice (BJW) (-)

Optimism (LOTR) (-)

Social desirability (-)

Demographics

Age (+)

For peer review only

For peer review only - http://bmjopen.bmj.com/site/about/guidelines.xhtml

											*PTED and all MIES subscales				
Moral injury only studies															
Bryan et al., 2016<br><br>Sample 2	Cross-sectional	935	Serving military personnel.<br><br>Army national guard (84%).	Convenience	27.05 (8.11)	82.3 %	57.4%	USA	NR	MIES 6+ (strongly agree)<br><br>3 subscales (transgressions-others, transgressions-self, betrayal)	Individual and/or work characteristics<br><br>Transgressions-others subscale:<br><br>Pre-deployment stressors (DDRI) (+)<br><br>Combat experiences (DDRI) (+)<br><br>Aftermath of battle (DDRI) (+)<br><br>Transgressions-self subscale:<br><br>Pre-deployment stressors (DDRI) (0)<br><br>Combat experiences (DDRI) (0)<br><br>Aftermath of battle (DDRI) (0)<br><br>Betrayal subscale:	Overall %<br><br>NR<br><br>Per subscale %<br><br>Cut off 6<br><br>Transgressions-others: 363 (38.8%)<br><br>Transgressions-self: 508 (54.3%)<br><br>Betrayal: 540 (47.8%)	Overall M/SD<br><br>NR<br><br>Subscale M/SD<br><br>Transgressions-others: 4.32(1.67)<br><br>Transgressions-self: 4.92(1.51)<br><br>Betrayal: 4.63(1.63)	5	None

For peer review only

											Pre-deployment stressors (DDRI) (+)<br>Combat experiences (DDRI) (0)<br>Aftermath of battle (DDRI) (0)<br>Demographics<br>Gender (0)				
Currier et al., 2015<br><br>Sample 1	Cross-sectional	131	Veterans.<br>Deployed to Iraq and/or Afghanistan<br>Marine Corps (38.32%), Active-Duty Army (35.1%) and Navy (22.9%).	Convenience	28.47<br>(5.87)	88%<br>Consistent with military demographic trends	26%<br>Consistent with ethnic diversity of the region of study	USA	NR	MIQ-M<br>20 items<br>Unidimensional.<br>Average 3.02 years since event (returning from war zone).	(Mental) health<br>Suicide risk (SQB-R) (0)<br>PTSD (PCL-C) (+)<br>Depression (PHQ-9) (+)<br>Individual and/or work characteristics:<br>Combat exposure (CES) (+)<br>Work and social adjustment (WSAS) (+)<br>Active duty and marine's vs other service branches (+)<br>Deployments (+)<br>Demographics	Overall<br>NR<br>Per subscale<br>NR	Overall M/SD<br>32.4(12.8)<br>Subscale M/SD<br>NR	3	NR

For peer review only

											Older age (+)<br>Gender (0)<br>Ethnicity (0)				
Currier et al., 2013	Cross sectional.	257	Teachers.<br>Classroom teachers (83.7%). 13.7% administrators e.g., principal (13.7%), and both classroom teacher and school administrator (2.7%)<br>Average time in role 15.44 (10.29).	Convenience<br>March 2012	42.02 (13.12)	31.4 %	NR	El Salvador	95 %	MIQ-T<br>12 items<br>Unidimensional.	(Mental) health<br>PTSD Reexperiencing (LASC) (+)<br>PTSD Avoidance (LASC) (+)<br>PTSD Hyperarousal (LASC) (+)<br>Physical burnout (SMBM) (+)<br>Cognitive burnout (+)<br>Emotional burnout (+)<br>Individual and/or characteristics<br>Violence exposure (SECV) (+)<br>Meaning made of stress comprehensibility (ISLES) (-)	Overall<br>NR<br>Per subscale<br>NR	Overall M/SD<br>13.92(6.71)<br>No subscales	4	NR

For peer review only

For peer review only - http://bmjopen.bmj.com/site/about/guidelines.xhtml

											Meaning made of stress footing in the world (ISLES) (-)				
Currier et al., 2017<br><br>Sample 2	Cross-sectional	624	Veterans.<br>1+ deployments to a war zone during their service.<br>49.2% (Army), 10.6% (Marine Corps), 22.1% (Navy), 21.8% (Air Force), 0.8% (Coast Guard).<br>97.4% (Active Duty), 11.9% (Military Reserves),	Convenience	NR	81.4 %	10.9%	USA	7.3 %	EMIS<br>2 subscales (self-directed MI and other-directed MI).<br>MIQ-M<br>7 items<br>1 subscale (causes)<br>MIES<br>2 subscales (perpetration and betrayal)	(Mental) health<br>PTSD (PCL 5) (+)<br>Depression (PHQ-8) (+)<br>Alcohol misuse (AUDIT-C) (+)<br>Anger (DAR-5) (-)<br>Guilt (PFQ-2) (+)<br>Shame (PFQ-2) (+)<br>Individual and/or work characteristics<br>Combat exposure – Survival threat (DDRI) (+)<br>Combat exposure – Aftermath of violence (DDRI) (+)	Overall<br>NR<br>Per subscale<br>NR	EMIS:<br>Overall M/SD<br>32.14(14.84)<br>Subscale M/SD<br>Self-MI 14.92(7.49)<br>Other-MI 17.22(8.23)<br>MIQ-M<br>Overall M/SD<br>NR<br>Subscale causes M/SD<br>9.29(3.41)<br>MIES<br>Overall M/SD<br>NR	5	NR

For peer review only

			6.3% (National Guard										Subscales<br>Perpetration 2.68(1.50)<br>Betrayal 2.80(1.66)		
Forkus, Juliana & Weiss. 2019	Cross sectional	203	Veterans.<br>Deployed to Iraq or Afghanistan.<br>Army (52.20%), Air Force (19.20%), Navy (15.80%), and Marine Corps (12.80%).	Convenience	35.08	77.30%	70.40%	NR	29.17%	MIES<br>Unidimensional	(Mental) health<br>PTSD (PCL-5) (+)<br>Depression (PHQ) (+)<br>Alcohol misuse (+)<br>Drug misuse (+)<br>Deliberate self-harm history (+)<br>Deliberate self-harm versatility (+)<br>Individual and/or work characteristics<br>Self-compassion (-)	NR	Overall M/SD<br>2.95(1.41)	5	NR
Frankfurt et al., 2018.	Longitudinal secondary data analysis.<br>MIES reported at T2	310	Veterans<br>Post 9/11.<br>Army (90.3%). Active	Convenience	40.67 (8.55).	76%	57%<br>Reflected geographic area	USA	NR	MIES<br>2 subscales (perpetration and betrayal)	(Mental) health<br>Military sexual trauma (DDRI) (+) for perpetration and betrayal subscales	NR	Overall M/SD<br>NR<br>Subscales<br>Perpetration 3.00(1.40)	4	None

For peer review only

			duty (96.8%).<br><br>Average years in role 13.50 (7.61)								Depression (+) for perpetration and betrayal subscales<br><br>PTSD (+) for perpetration and betrayal subscales<br><br>Shame (+) for perpetration and betrayal subscales<br><br>Guilt (+) for perpetration and betrayal subscales<br><br>Individual and/or work characteristics<br><br>Combat exposure (0)*<br><br>Combat exposure (+)**<br><br>*betrayal<br>**perpetration		Betrayal 3.41(1.64)		
Griffin et al., 2020<br><br>Narrative synthesis only	Cross sectional.	498	Military service members and veterans<br><br>Army (22.49%), Navy (17.47%), Air Force	Convenience	NR	73.90 %	NR	USA	NR	MIES<br><br>5 of 9 items to create 5 profiles	NR due to using 'profiles'	Overall %<br><br>NR<br><br>Subscales<br><br>No moral distress (42% of sample)	NR	5	NR

For peer review only

			(45.78%), Marines (11.24%), Coastguard (2.61%). Active Duty (29.52%).									Moral Distress-Other (19%)<br>Witnessing-Only (16%)<br>Moral distress-Self (8%)<br>Moral distress-Self and Other (15%)			
Jinkerson, Battles. 2019.	Cross sectional	72	Veterans<br>Vietnam and post-Vietnam eras.<br>Army (59.7%), Navy (13.9%), Air Force (12.5%), Marine Corps (12.5%)	Convenience	NR	87.5 %	45.8%	USA	76 %	MIQ-M<br>20 items<br>Unidimensional	(Mental) health<br>Meaning in life (-)<br>Depression (PHQ-2) (+)<br>Anxiety (GAD-2) (+)<br>PTSD (PCL-5) (+)<br>PTSD Reexperiencing (PCL-5) (+)<br>PTSD avoidance (PCL-5) (+)<br>PTSD Negative emotions (PCL-5) (+)	Per item % (top 5 items)<br>Betrayal by military/political leaders N = 32 (44.5%)<br>experiencing events that were chaotic and beyond control N = 32 (44.4%)<br>failing to save the life of someone in war N = 24 (33.3%)<br>surviving when others	Overall M/SD<br>3.48(0.35)<br>Subscales<br>NR	4	NR

For peer review only

For peer review only

											PTSD hyperarousal/hypervigilance (PCL-5) (+)<br>Global guilt (TRGI) (+)<br>Distress (TRGI) (+)<br>Guilty cognitions (TRGI) (+)<br>Wrongdoing (TRGI) (+)<br>Lack of justification (TRGI) (0)	didn't N = 21 (29.2%)<br>feeling betrayal by trusted civilians N = 32 (29.2%)			
Jordan et al., 2017.	Longitudinal.<br>Data from 1 month post deployment reported here and from cohort 4 from the parent study.	867	Currently serving active-duty marines from a single infantry battalion engaged in heavy ground combat while deployed to Afghanistan.	Convenience	NR	NR	NR	USA	NR	MIES 4+ (slightly agree)<br>Subscales: 2 subscales (transgressions-self and betrayal)	(Mental) health<br>PTSD (PLC-S) (+)<br>Guilt/shame (PANAS) (+) *<br>Anger (PANAS) (+) *<br>Individual and/or characteristics<br>Dissociation (PDEQ) (+) *<br>Combat exposure (CES) (+) *<br>* transgressions-self and betrayal subscales	Overall %<br>NR<br>Per subscale<br>Transgressions-self 214 (24.1%)<br>Betrayal 252 (28.4%)<br>Transgressions-self and betrayal: 333 (37.5%)<br>Per item %<br>Item 1: 165 (18.6%)	Overall M/SD<br>NR, only used below subscales<br>Subscales<br>Transgressions-self = 1.87(1.17)<br>Betrayal = 2(1.23)	5	NR

												Item 2: 89 (10%)<br>Item 3: 128 (14.4%)<br>Item 4: 103 (11.6%)<br>Item 7: 179 (20.2%)<br>Item 8: 133 (15%)<br>Item 9: 132 (14.9%)			
Lee Aldwin, Kang. 2020	Cross sectional.	367<br>354 responded to MIES	Veterans<br>Deployed to the Vietnam war in 1664-1973.<br>Army (88%).	Convenience	72 (2.66)	100%	NR	Korea	37.4 %	MIES 3+ (sometimes) and 4+ (often) 5-point scale not 6 (1=never, 2=seldom, 3=sometimes, 4=often, 5=always).<br>3 subscales (transgressions-others, transgressions-self, betrayal)	(Mental) health<br>PTSD (Checklist for DSM-5) (+)<br>Depression (BSI) (+)<br>Anxiety (BSI) (+)<br>Individual and/or work characteristics<br>Optimism (LOT) (-)<br>Unit cohesion (-)<br>Homecoming experience (DDRI-2) (0)	Overall %<br>Cut off 4+:<br>104(29.4%)<br>Cut off 3+:<br>176(49.7%)<br>Subscales %<br>Transgressions -others cut off 4+: 57(16.1%)<br>Transgressions -self cut off 4+: 53(14.9%)	Overall M/SD<br>1.80(0.80)<br>Subscales<br>Transgressions -others: 2.04(1.04)<br>Transgressions -self: 1.69(0.86)<br>Betrayal: 1.80(0.91)	5	None

										40-50 years since event	Combat exposure (CES) (+)<br>Malevolent environments (CES) (+)<br>Perceived threat (CES) (+)<br>Demographics<br>Age (0)<br>Education (-)<br>Marital status (0)<br>Income (0)	Betrayal cut off 4+: 59(16.7%)<br>Transgressions -others cut off 3+: 130(36.7%)<br>Transgressions -self cut off 3+: 115(32.5%)<br>Betrayal cut off 3+: 121(34.2%)			
Ogle, Reichwald, Rutland 2018<br>Narrative synthesis only	Cross sectional	356	Serving military analysts<br>Geospatial intelligence analysts (29%), cryptologic language analysts (54.8%) who provide support to remote combat and global vigilance	Convenience	NR	70.3 %	NR	USA	NR	MIES 10 items instead of 9 3 subscales (transgressions-others, transgressions-self, betrayal)	(Mental) health<br>PTSD (PCL-5) (+) *<br>PTSD Avoidance (PCL-5) (+) *<br>PTSD Mood/cognition (PCL-5) (+) *<br>PTSD Hypervigilance (PCL-5) (+) *<br>Reliving (PCL-5) (+) *	Overall %<br>NR<br>Subscales %<br>NR<br>Per item % split by combat exposure level<br>Item 1: None = 9.1%, Low = 21%, Middle = 26%, High = 47.3%	Overall M/SD<br>NR<br>Subscales M/SD<br>NR	5	NR

For peer review only - http://bmjopen.bmj.com/site/about/guidelines.xhtml

operations and who have routine exposure to related video, audio, still images, and other media.	Social adjustment (-) *<br><br>Affect work/home (+) *<br><br>Individual and/or work characteristics<br><br>Combat exposure (CES) (+) *<br><br>Work satisfaction (-) *transgressions-self and betrayal<br><br>Work satisfaction (0) transgressions-others<br><br>Combat ambiguity (+) *<br><br>* Transgressions-others, transgressions-self and betrayal subscales	Item 2: None = 11.6%, %, Low = 11.6%, Middle = 6.2%, High = 15.4%<br><br>Item 3: None = 4.7%, %, Low = 29%, Middle = 46.4%, High = 60.5%<br><br>Item 4: None = 2.3%, %, Low =8.2%, Middle = 5%, High = 11%,<br><br>Item 5: None = 2.3%, Low = 9.4%, Middle = 4.1%, High = 9.9%<br><br>Item 6: None = 4.6%, Low =7.1%, Middle = 2%, High = 8.8%<br><br>Item 7: None = 2.3%, Low =6.9%, Middle = 2%,

For peer review only

												High = 7.7%, r_s = .109<br><br>Item 8: None = 11.3%, Low =17.5%, Middle = 10.1%, High = 20.9%<br><br>Item 9: None = 7%, Low =11.7%, Middle = 3%, High = 13.2%<br><br>Item 10: None = 7%, Low =10.5%, Middle = 5%, High = 7.7%			
Wisco et al., 2017.	Cross sectional.	564	Veterans.<br><br>Experienced combat.<br><br>Army 41%.	Epidemiological sampling, recruited from the National Health and Resilience in Veterans Study (NHRVS).	NR	93%	76.2%	USA	25 %	MIES: 5+ (moderate) 3 subscales (transgressions-others, transgressions-self, betrayal) 33.7 years (19.1) since event (last combat deployment)	Individual and/or work characteristics<br><br>Vietnam war era (0)<br><br>Military branch (army) (+)<br><br>Multiple deployments (+)<br><br>Moderate/heavy combat (+)<br><br>Demographics<br><br>Age (0)	*Reports weighted % and SE<br><br>Overall % 223, (41.8%, 2.1%))<br><br>Subscale % Transgressions -Others 126, (25.5%, 1.8%))<br><br>Transgressions -Self 48 (10.8%, 1.3%))	Overall M/SD<br><br>2.11(1.08)<br><br>Subscales M/SD<br><br>Transgressions -others 2.71(1.66)<br><br>Transgressions -self 1.75(1.16)<br><br>Betrayal	1	None

For peer review only

											Gender (0)<br>White race (-)<br>College education (-)<br>Annual income >60k (-)<br>Currently employed (+)	Betrayal 142 (25.1%, 1.8%)<br>Per item<br>Item 1: 115 (23.8%, 1.8%)<br>Item 2: 65, (14.1%, 1.5%)<br>Item 3: 37 (8.9%, 1.2%)<br>Item 4: 30 (5.9%, 1%)<br>Item 5: 21 (3.9%, 0.8%)<br>Item 6: 19 (3.6%, 0.8%)<br>Item 7: 107 (18.6%, 1.7%)<br>Item 8: 81 (15%, 1.5%)<br>Item 9: 31 (5%, 0.9%)	2.19(1.29)		
Zerach, Levi-Belz. 2017	Cross sectional	191	Veterans.<br>100% army.	Convenience<br>March-July 2017.	25.39 (2.37)	85.4 %	NR	Israel	86.8 %	MIES 4+ (slightly agree)<br>3 subscales (transgressions-others, transgressions-self, betrayal)<br>Time since deployment 4.36(2.27) years.	(Mental) health<br>Depression (DAQ) and MIQ-M (0)<br>Depression (DAQ) and MIES (+) *<br>PTSD (PCL-5) and betrayal (+)<br>PTSD (PCL-5) and transgression-others, transgressions-self and MIQ-M (0)	Overall %<br>92(48.4%)<br>Subscales<br>Transgressions -others 64 (33.7%)<br>Transgressions -self 42 (21.9%)	Overall M/SD<br>2.00(0.97)<br>Subscales MIES<br>Transgressions -others 2.42(1.39)<br>Transgressions -self 1.68(1.05)	4	NR

For peer review only - http://bmjopen.bmj.com/site/about/guidelines.xhtml

MIQ-M 2+ (seldom)<br>14 causal items<br>1 subscale (causes)	Guilt-global (TRGI) and MIQ-M and MIES (+) *<br>Guilt-distress (TRGI) and MIQ-M and MIES (+) *<br>Guilt-cognitions (TRGI) and MIQ-M and MIES (+) *<br>Shame-intrinsic (TSRI) and MIQ-M and MIES (+) *<br>Shame-extrinsic (TSRI) and MIQ-M and MIES (+) *<br>Self-disgust-self (TRSI) and MIQ-M and MIES (0) *<br>Self-disgust -ways (TSRI) and betrayal (+),<br>Self-disgust ways (TSRI) and transgressions-self, transgressions-others, and MIQ-M (0)<br>Individual and/or work characteristics	Betrayal 59 (31%)<br>Per item MIES %<br>Item 1: 55 (29%) Item 2: 42 (22%)<br>Item 3: 23 (12%) Item 4: 25 (13%)<br>Item 5: 19 (10%)<br>Item 6: 23 (12%)<br>Item 7: 48 (25%) Item 8: 32 (17%) Item 9: 29 (15%)<br>Per item MIQ-M %<br>Item 4: 86 (45.2%) Item 8: 60 (31.4%)	Betrayal 2.14(1.22)<br>Subscales MIQ-M<br>MIQ causes 1.34(0.34)

For peer review only

											Combat exposure (CES) (+) MIQ-M<br>Combat exposure (0) MIES *<br>*transgressions-self, transgressions-other and betrayal subscales				
Papazoglou et al., 2019.	Cross sectional.	390	Service police officers.<br>National Police of Finland.<br>Average years of police experience 16.87(9.11)	Convenience	41.21 (8.42)	73.5 %	100%	Finland	86 %	MIES 5+ (moderately agree) and 6+ (strongly agree) Item 6 not included 2 subscales (perpetration and betrayal)	(Mental) health<br>PTSD (PCL-C) (+) *<br>Compassion fatigue (CSF) (+) *<br>Compassion satisfaction (+) *<br>Individual and/or work characteristics<br>Dark triad (SD3) (+) *<br>*Perpetration and betrayal subscales	Overall %<br>Overall, MI (moderate): 289 (74%)<br>Subscale %<br>Perpetration 152 (39%) (moderate)<br>Betrayal 250 (64%) (moderate)<br>Perpetration 39 (10%) (high)<br>Betrayal 94 (24%) (high)	Overall M/SD<br>NR<br>Subscale M/SD<br>Perpetration 2.61 (1.19)<br>Betrayal 3.77 (.98)	4	NR
Richardson et al., 2020	Longitudinal (T2)	5,227	Veterans.<br>Recently separated	Epidemiological, Recruited from The Veteran Metrics	NR	82.1 %	66.9%	USA	10.67%	MIES 4+ (slightly agree) 2 subscales, (MI-self	NR	Overall %<br>NR<br>Subscale %	Overall M/SD<br>2.24(1.07)	2	None

from service<br><br>Army (36.8%), Navy (22.4%), Air Force (20.5%), marines (20.2%).	Initiative (TVMI) study.		and MI-other) Within 90 days since event (separation from service)	MI-Self: 516 (9.87%)<br><br>MI-Other: 1033 (19.76%)<br><br>Per item prevalence<br><br>Item 1: 2432 (46.53%)<br><br>Item 2: 1496 (28.62%)<br><br>Item 3: 833(15.94%)<br><br>Item 4: 750(14.35%)<br><br>Item 5: 813(15.55%)<br><br>Item 6: 741(14.18%)<br><br>Item 7: 1955(37.4%)<br><br>Item 8: 1472<br><br>(28.16%)<br><br>Item 9: 1039<br><br>(19.88%)	Subscale M/SD<br><br>MI-Self: 1.79(1.25)<br><br>MI-Other: 2.51(1.40)

For peer review only

For peer review only - http://bmjopen.bmj.com/site/about/guidelines.xhtml

Feinstein<br>,<br>Pavisian,<br>Storm.<br>2018	Cross sectional	80	Journalists.<br>Years in role 18.28 (8.09)<br>months covering the refugee crisis 19.65 (19.41)	Convenience	42.95 (8.44)	58.8 %	70.40%	Nine European and American news organisations	70.2 %	MIES<br>6 items<br>1 subscale (perpetration)	(Mental) health<br>PTSD intrusion (IES-R) (+)<br>PTSD avoidance (IES-R) (+)<br>PTSD arousal (IES-R) (+)<br>Depression (BDI-II) (0)<br>Alcohol consumption (0)<br>Individual and/or work characteristics<br>Higher workload in past year (+)<br>Control over resources (-)<br>Guilt (+)<br>Demographics<br>Having children (+) t-test<br>Gender (0)<br>Age (0)<br>Education (0)<br>Marriage (0)	Overall %<br>NR<br>Subscales %<br>NR	Overall M/SD<br>NR (only one subscale)<br>Subscales<br>Perpetration 2.83(0.93)	5	HR is director of INSI

For peer review only

											Marital status (0)				
Sugrue, 2020	Cross sectional	218	K-12 education<br><br>Classroom teacher (50.5%), special education staff (19.3%), Non-classroom instructional support (10.1%), School social workers (5%), English language learner (ELL) teachers (5%), Specialist teachers (4.6%), School psychologi	Convenience	42.6 (11.9)	22.9 %	77.1%	USA	<7 %	MIES 4+ (slightly agree)<br>3 subscales (transgressions-others, transgressions-self, betrayal)	(Mental) health<br><br>Transgressions-others subscale:<br><br>Personal burnout (CBI) (+)<br><br>Work burnout (CBI) (+) *<br><br>Client burnout (CBI) (+)<br><br>Global guilt (TRGI) (+)<br><br>Distress Scale (TRGI) (+)<br><br>Hindsight bias (TRGI)<br><br>Wrongdoing (TRGI)<br><br>Lack of justification (TRGI) (0)<br><br>Stress of conscience (internal and external	Overall %<br><br>191 (85.3%).<br><br>Subscale %<br><br>Transgressions -others, 175 (80.2%)<br><br>Transgressions -self, 98 (45.4%)<br><br>Betrayal, 148 (68.4%)<br><br>Transgressions -others, 115(52.7%) – moderate (5) cut off	Overall M/SD<br><br>3.49(1.24)<br><br>Subscale M/SD<br><br>Transgressions -<br><br>others 4.4(1.5)<br><br>Transgressions -self, 2.9(1.5)<br><br>Betrayal 3.8(1.5)	5	NR

For peer review only

sts (3.7%), School counsellors (0.9%), school nurses (0.5%)<br><br>Average years working in education 15.0(10.1)	subscales and total (SCQ)) (+)<br><br>Transgressions-self subscale:<br><br>Personal burnout (CBI) (+)<br><br>Work burnout (CBI) (+) *<br><br>Client burnout (CBI) (0)<br><br>Global guilt (TRGI) (+)<br><br>Distress Scale (TRGI) (+)<br><br>Hindsight bias (TRGI)<br><br>Wrongdoing (TRGI)<br><br>Lack of justification (TRGI) (+)<br><br>Stress of conscience (internal and external subscales and total (SCQ)) (+)<br><br>Betrayal subscale:<br><br>Personal burnout (CBI) (+)

For peer review only

Work burnout (CBI) (+) *

Client burnout (CBI) (+)

Global guilt (TRGI) (+)

Distress Scale (TRGI) (+)

Hindsight bias (TRGI)

Wrongdoing (TRGI)

Lack of justification (TRGI) (0)

Stress of conscience (internal and external subscales and total (SCQ)) (+)

Individual and/or work characteristics

Transgressions-others subscale

Intention to leave (ILS) (+)

Transgressions-self subscale

Intention to leave (ILS) (0)

For peer review only

For peer review only - http://bmjopen.bmj.com/site/about/guidelines.xhtml

											Betrayal subscale<br>Intention to leave (ILS) (+)				
Aldridge, Scott, Paskell. 2019.	Cross sectional.	104	Veterans.<br>British Army, Royal Navy or Royal Air Force and had experienced active deployment.	Convenience<br>Feb 2017-Nov 2017.	47 (10.8)	95.2 %	NR	UK	NR	MIQ-M<br>19 items<br>Unidimensional.	(Mental) health<br>PTSD (PCL-M) (+)<br>Individual characteristics<br>Guilt proneness (TOSCA)<br>(-)<br>Shame proneness (TOSCA) (0)<br>Demographics<br>Age (0)<br>Gender (0)<br>Length of service (0)<br>Previous therapy (0)	Overall %<br>NR<br>Subscale %<br>NR	Overall M/SD<br>36.75(10.12)<br>Subscale M/SD<br>NR	5	NR

For peer review only

Andrukonis, Potopopova. 2020.	Cross sectional.	153	Animal shelter.<br><br>Shelter director (n=16), assistant director (4), animal shelter supervisor/ manager (21), animal control officer (18), kennel attendant/animal care tech (29), customer service representative (13), vet/vet tech/medical (22), behaviour/training (9), volunteer/rescue/community (3), other (22)<br><br>Years in job M=5.9	Convenience<br><br>Dec 2017-April 2018.	NR	NR	NR	USA	NR	MIES Higher score represents lower moral injury Unidimensional.	Individual and/or work characteristics<br><br>Shelter type (0)<br><br>Admission type (0)<br><br>Euthanizes in job (-)<br><br>Demographics<br><br>Months on the job (0)	Overall %<br><br>NR<br><br>Subscale %<br><br>NR	Overall M/SD<br><br>0.96(4.22)<br><br>Subscale M/SD<br><br>NR	5	None

For peer review only

Haight., Sugrue, Calhoun. 2017.	Cross sectional	38	CPS<br>Social worker (n = 13) County attorney (n = 5)<br>Guardians ad litem (n = 5)<br>Student attorneys (n = 10)<br>Law school clinic and parent support staff (n = 5)<br>Years in CPS 8.5(8.6)	Convenience	NR	18%	66%	USA	56.33%	MIES 4+ (slightly agree)<br>3 subscales (transgressions-others, transgressions-self, betrayal)	Demographics<br>Age (0)<br>Gender (0)<br>Race (0)<br>Years of experience in CPS (0)<br>Years of education and transgressions-self and betrayal (-)	Overall %<br>NR<br>Subscale %<br>Transgressions-others 35(92%)<br>Transgressions-self 9(24%)<br>Betrayal 12(32%)	Overall M/SD<br>NR<br>Subscale M/SD<br>Transgression-others 5.1(1)<br>Transgression-self 2.7(1.4)<br>Betrayal 3.1(1.2)	5	NR
Battles et al. 2019.	Cross sectional	380	Currently serving military and veterans that had deployed to 1+ war	Convenience	35.29 (9.58)	68.42%	71.9% of men and 61.7% of women	USA	NR	MIQ-M<br>12 causal items<br>1 subscale (causes)	(Mental) health<br>Spiritual injury (+)<br>Total alcohol use (AUDIT) in men (+)<br>Total alcohol use in women (0)<br>Alcohol consumption (AUDIT) in men (+)	Overall %<br>NR<br>Subscale %<br>NR	Overall M/SD<br>NR<br>Subscale M/SD (causes 12 items)<br>1.26(0.42)	5	NR

For peer review only

			zones for 3+ months.<br><br>Navy (43.5% of men, 53.3% of women).<br><br>No longer serving (67.1% of men, 66.7%) of women.<br><br>Length of time serving (men 12.88, 10, women, 9.84, 8)								Alcohol consumption (AUDIT) in women (0)<br>Alcohol dependence (AUDIT) in men (+)<br>Alcohol dependence (AUDIT) in women (0)<br><br>Individual and/or work characteristics<br>Combat exposure (CES) (+)<br>Military status (+) (0)<br>Military branch (+)<br><br>Demographics<br>Years in service (0)				
Braitman et al., 2018.<br><br>Narrative synthesis only	Cross sectional	328	Current military or veterans with deployed experience<br><br>Veterans (69.5%)<br><br>National Guard/Reservists (19.5%)	Convenience	32.42 (7.71)	56.1 %	68.0%	USA	NR	MIQ-M modified 2+ (seldom)<br><br>27 MIE items plus 135 follow up questions (5 per MIE item)<br><br>3 subscales. (atrocities	(Mental) health<br>Depression (CES-D-10) (+)<br>Anxiety (KAS) (+)<br>PTSD (PCL-5) (+)<br>Hazardous alcohol use (AUDIT) (+)<br>Drug abuse (DAST) (+)<br>Drug abuse (DAST) (0) *<br><br>All subscales<br>*LFT and AoW	Overall %<br>NR<br><br>Subscale %<br>NR<br><br>Per item %<br>Item 7: 216 (65.9%)<br>Item 8: 174 (53%)<br>Item 10: 182(55.5%)<br>Item 11:	Overall M/SD<br><br>NR<br><br>Subscale M/SD<br><br>AoW MIEs 1.77(0.63)<br>PCoW MIEs 1.99(0.66)<br>LFB MIEs 2.12(0.78)	5	None

For peer review only - http://bmjopen.bmj.com/site/about/guidelines.xhtml

Active-duty members (9.5%)<br><br>Army (46.3%)<br>Navy (29.9%)<br>Air force (7.9%)<br>Marines (5.5%)<br>National guard (4%)<br>Reserves (4.6%).	at war (AoW); psychological consequences of war (PCoW), leadership failure or betrayal (LFB).	155(47.3%)<br>Item 15: 181(55.2%)<br>Item 16: 150(45.7%)<br>Item 17: 178(54.3%)<br>Item 19. 191(58.2%)<br>Item 22: 179(54.6%)<br>Item 23: 170(51.8%)<br>Item 24: 198(60.4%)<br>Item 26: 192(58.5%)<br>Item 27: 183(55.8%)<br>Item 6: 208 (63.4%)<br>Item 9: 231 (70.4%)<br>Item 13: 233 (71%)<br>Item 18: 227(69.2%)<br>Item 21: 220(67.1%)<br>Item 25: 199(60.7%)<br>Item 1: 218(66.5%)<br>Item 2: 225(68.6%)<br>Item 4. 239(72.9%)<br>Item 3: 200(61%)	AoW defining characteristics 2.21(0.67)<br>PCoW defining characteristics 2.14(0.66)<br>LFB defining characteristics 2.12(0.67)<br>All defining characteristics 2.11(0.66)

For peer review only

												Item 5:<br>168(51.2%)<br>Item 12:<br>208(63.4%)<br>Item 14:<br>174(53%)<br>Item 20.<br>214(65.2%)			
Lancaster, Harris, 2018.	Cross sectional	182	Veterans<br><br>Deployed to Iraq or Afghanistan<br><br>Army (61.0%)<br>Air Force (17.0%),<br>Marine Corps (14.8%),<br>Navy (7.1%).	Convenience	33.66 (7.23)	80.2 %	78.0%	NR	32.3 3%	MIES<br>3 subscales (transgressions-others, transgressions-self, betrayal)<br><br>MIQ-M<br><br>19 items<br><br>2 subscales (causes and effect)	(Mental) health<br>Anger (DAR-5) (+) *<br>Depression (PHQ) (+) *<br>Alcohol (AUDIT) (+) *<br>PTSD (PCL-5) (+) *<br>Guilt (+) *<br>Shame (+) *<br><br>Individual and/or work characteristics<br>Combat (CES) (+)*<br>CES (0)**<br><br>All subscales<br>*transgressions-others and transgressions-self subscales<br>**betrayal subscale	Overall %<br><br>NR<br><br>Subscale %<br><br>NR	Overall M/SD MIES<br>2.75(1.32)<br><br>Subscale M/SD MIES<br>Transgressions-others<br>3.49(1.50)<br><br>Transgressions-self<br>2.45(1.53)<br><br>Betrayal<br>2.67(1.57)<br><br>Overall M/SD MIQ-M<br>33.70(11.90)<br><br>Subscale M/SD MIQ-M<br>Effect<br>11.54(4.21)<br><br>Causes<br>22.19(8.32)	5	NR

For peer review only

Lancaster, 2018	Cross sectional	161	Veterans. Post 9/11<br><br>Army (43.5%), Navy (19.9%), Air Force (16.1%), Marine Corps (13.7%), Coast Guard (6.8%).	Convenience	35.08 (9.62)	71.4 %	73.9%	NR	5.18 %	MIES<br>7 of 9 items<br>2 subscales (transgressions-self and betrayal)	(Mental) health<br>PTSD (PTSD checklist 5) (+) *<br>Depression (PHQ) (+) *<br>Anger (+) *<br>Guilt/shame (SSGS) (+) *<br><br>Individual and/or work characteristics<br>Transgressive acts (TAS) (+) *<br>*transgressions-self and betrayal subscales	Overall %<br><br>NR<br><br>Subscales %<br><br>NR<br><br>*Range 12.6% to 23.3% on items	Overall M/SD<br>NR (only 2 subscales used)<br><br>Subscales M/SD<br>Transgressions-self, 2.56(1.43)<br>Betrayal 2.72(1.46)	5	NR
Akhtar, Faize, Malik & Tabusam, 2022	Cross-sectional	108	Healthcare<br><br>Doctors (51.9%)<br><br>Nurse (26.9%)<br><br>Paramedics (21.3%)	Purposive<br><br>August 2020-January 2021.	27.67 (6.17)	41.7 %	NR	Pakistan.	NR	MISS-HP 36+	Individual and/or work characteristics<br><br>Psychological resilience (CD-RISC-10): (-)<br><br>Demographics<br><br>Age (0)<br><br>Experience (-)<br><br>Work hours (+)<br><br>Female gender (+)<br><br>Marital status (0)<br><br>Hospital type (0)<br><br>Profession (0)	Overall %<br><br>75 (69.44%)	Overall M/SD<br><br>43.36 (9.64)	6	NR

For peer review only - http://bmjopen.bmj.com/site/about/guidelines.xhtml

											Speciality/department psychiatry (+)<br>Work area/duty station (0)				
Qi et al., 2022<br>Two studies.	Study 1:<br>Cross-sectional<br>Study 2:<br>Cross-sectional	Study 1:<br>3006<br>Study 2:<br>3465	Study 1:<br>Healthcare<br>Nurse (18%)<br>Internal medicine (19.4%)<br>Obstetrics/ gynaecology/paediatrics (34.7%)<br>Surgery (9.6%)<br>Psychiatry (11.4%)<br>Other<br>Study 2:<br>Healthcare<br>Nurse (14.7%)	Study 1:<br>Snowball.<br>March-April 2020.<br>Study 2:<br>Snowball.<br>March-April 2021	Study 1:<br>NR<br>Study 2:<br>NR	Study 1:<br>34.9 %<br>Study 2:<br>24.6 %	Study 1:<br>NR<br>Study 2:<br>NR	Study 1:<br>China<br>Study 2:<br>China	Study 1:<br>75 %<br>Study 2:<br>86 %	Study 1:<br>MISS-HP 36+<br>EMIS-SF<br>Study 2:<br>MIISS-HP 36+	Study 1:<br>(Mental) health<br>Depression (PHQ) (+)<br>Anxiety (GAD-7) (+)<br>Burnout emotional exhaustion (MBI-HSMP) (+)<br>Burnout reduced personal accomplishment (MBI-HSMP) (-)<br>Burnout depersonalisation (MBI-HSMP) (+)<br>Well-being (SFI) (-)<br>* EMIS and MISS-HP<br>Study 2:<br>NR at univariate level.	Study 1:<br>Overall, MISS-HP %<br>1695 (56.4%)<br>Study 2:<br>Overall, MISS-HP %<br>1270 (36.7)	Study 1:<br>MISS-HP overall:<br>46.9 (12.7)<br>EMIS-SF overall:<br>2.55 (0.80)<br>Study 2:<br>MISS-HP:<br>45.9(11.3)	5	None

For peer review only - http://bmjopen.bmj.com/site/about/guidelines.xhtml

			Internal medicine (44.7%)<br>Obstetrics/ gynaecology/paediatrics (21.1%)<br>Surgery (4.9%)<br>Psychiatry (8.5%)<br>Other (6.1)												
Amsalem et al., 2021	Longitudinal	350	Healthcare<br>Nurses (68%)<br>Physicians (15%)<br>Emergency medical technicians (9%)<br>Physical therapists (3%)<br>Pharmacists (2%)<br>Social workers	Convenience<br>September-December 2020	34.8 (11.5)	26%	73%	USA	NR	MIES: 3+ (agree)<br>Likert scale 1 (disagree), 2 (strongly disagree) 3 (agree) 4 (strongly agree)<br>3 subscales (transgressions-others, transgressions-self, betrayal)	(Mental) health<br>Depression (PHQ) (+)<br>Anxiety (GAD-7) (+)<br>Suicidal ideation (+)<br>PTSD (PC-PTSD) (+)<br>Demographics<br>Age (0)<br>Experience (-)<br>Work hours (+)<br>Female gender (+)<br>Marital status (0)	Overall %<br>267 (76.29%)<br>Per subscale %<br>Transgressions -others: 215 (61.43%)<br>Transgressions -self: 101 (28.86)<br>Betrayal: 205 (58.57%)<br>Per item:	Overall M/SD<br>1.98 (0.72)<br>Subscale M/SD<br>Transgressions -others: 2.41(1.04)<br>Transgressions -self: 1.63(0.82)<br>Betrayal: 2.15(0.92)	5	None

For peer review only

			and other therapists (1%)<br><br>Other (2%)								Hospital type (0)<br><br>Profession (0)<br><br>Speciality/department psychiatry (+)<br><br>Work area/duty station (0)	Item1: 192 (55%)<br><br>Item 2: 171 (49%)<br><br>Item 3: 66 (19%)<br><br>Item 4: 68 (19%)<br><br>Item 5: 71 (20%)<br><br>Item 6: 72 (21%)<br><br>Item 7: 157 (45%)<br><br>Item 8: 111 (32%)<br><br>Item 9: 152 (43%)			
Perez, Larson & Bair 2021	Cross-sectional	85	Veterans<br><br>Served between 2001-2014.<br><br>Army (63.5%)<br><br>Marines (8.2%)	Convenience	NR	68.2 %	78.8%	USA	NR	EMIS<br><br>2 subscales (self-directed MI and other-directed MI)	Individual and/or work characteristics<br><br>Fairness (MFQ) (0)<br><br>Loyalty (MFQ) (+) *<br><br>Loyalty (MFQ) (0) **<br><br>Authority (MFQ) (0)<br><br>Sanctity (MFQ) (0)	Overall %<br><br>NR<br><br>Per subscale<br><br>NR	Overall M/SD<br><br>2.45(0.98)<br><br>Subscale M/SD<br><br>Self-MI 2.16(1.08)<br><br>Other-MI 2.80(1.06)	5	NR

For peer review only

			Navy (10.6%)<br>Air Force (15.3%)<br>Coast Guard (2.4%)								*Self-MI<br>**Other-MI				
Benatov, Zerach & Levi-Belz 2022	Cross-sectional	296	Health and social care<br>Doctor (34.1%)<br>Nurse (including midwives) (44.4%)<br>Social and psychological care (17.4%)<br>Clinical support (5.9%)	Convenience	40.28 (10.8)	22.4 %	NR	Israel	NR	MIES 4+ (slightly agree)<br>3 subscales (transgressions-others, transgressions-self, betrayal)<br>MISS-HP 36+	(Mental) health<br>Depression (PHQ) (+)<br>Anxiety (GAD-7) (+)<br>Individual and/or work characteristics<br>Thwarted belongingness (INQ) (+)<br>*For MISS-HP and MIES scales	MIES:<br>Overall %:<br>NR<br>Per subscale %<br>Transgressions-others: 146 (49.3%)<br>Transgressions-self: 94 (31.8%)<br>Betrayal: 184 (62.2%)<br>Per item %:<br>Item 1: 135 (45.5)<br>Item 2: 108 (36.5%)	MIES:<br>Overall M/SD<br>(From supplement)<br>22.54(10.48)<br>Subscale M/SD<br>Transgressions-others: 4.08(2.71)<br>Transgressions-self: 1.49(0.81)<br>Betrayal: 2.81(1.33)<br>MISS-HP overall:<br>33.90(12.72)	5	NR

For peer review only

												Item 7: 135 (45.5%)<br>Item 9: 94 (31.8%)<br>MISS-HP overall %<br>120 (40.7%)			
Borges et al., 2021	Longitudinal	211	Healthcare<br>Physician (12.32%)<br>Nurse (10.43%)<br>Mental health provider (61.14%)<br>Occupational therapist (1.90%)<br>Nurse practitioner (2.37%)<br>Physician assistant (0.47%)	Convenience<br>May 2020 – March 2021	NR	16.26 %	89.16%	USA	NR	MIES 2+ (moderately agree)<br>Lower score indicates higher moral injury<br>2 subscales (perpetration and betrayal)	Demographics<br>Sex (0)<br>Race (0)<br>Profession (0)<br>No other variables reported at univariate level	Overall %<br>109 (51.66%)<br>Per subscale %<br>Perpetration: 77 (36.49%)<br>Betrayal: 88 (41.71%)	Overall M/SD<br>NR<br>Subscale M/SD<br>NR	5	None

For peer review only

Boscarino et al., 2022<br><br>Narrative synthesis only	Cross-sectional	1032	Military veterans.	Random sample of veterans on a registry care at a private hospital.<br><br>February 2016 – February 2017	NR	95.5 %	NR	USA	55 %	MIES 75th percentile<br><br>Unidimensional	(Mental) health<br><br>Cage positive alcohol score (+)<br><br>Used alc/drugs to cope (+)<br><br>Pain interferes (+)<br><br>Sleep problems (+)<br><br>Concussion history (+)<br><br>Mental health treatment in past year (+)<br><br>Opioid dependence past year (+)<br><br>Individual/work characteristics<br><br>Guard/reserve (-)<br><br>Combat high (+)<br><br>Drafted (+)<br><br>Vietnam war deployment (+)<br><br>Low unit support/morale (+)<br><br>High stress past year (+)	Overall %<br><br>764 (74%)<br><br>Subscale %<br><br>NR	Overall M/SD<br><br>NR<br><br>Subscale M/SD<br><br>NR	3	None

For peer review only

											High lifetime trauma (+)<br>Low homecoming support (+)<br>High child abuse/neglect (+)<br>High fear of death (+)<br>High neuroticism (+)<br>Low self-esteem (+)<br>Repression to cope (+)<br>High anomie (+)<br>Anti-social personality (+)<br>Used marijuana 50+ times (+)<br>Demographics<br>Age (+)				
Brady et al., 2021	Cross-sectional<br>.	390	Nursing home staff.<br>Nurses (30.8%)<br>Healthcare assistants (44.1%)	Convenience<br>November 2020 – January 2021.	NR	12.8 %	83.3%	Republic of Ireland.	10.2 %	MIES 4+ (slightly agree)<br>Cut off: 4+ (Slightly agree)<br>3 subscales (transgressions-others,	Demographics<br>HCA compared to non-clinical staff (+)	Overall %<br>237 (60.8%)<br>Per subscale %<br>Transgressions -others: 189 (48.5%)	Overall M/SD<br>2.35(1.09)<br>Subscale M/SD<br>Transgressions -others: 2.93(1.51)	4	Honoraria for board meeting.

For peer review only

			Non-clinical (25.1%)							transgressions-self, betrayal)		Transgressions-self: 92 (23.6%)<br>Betrayal: 166 (42.6%)	Transgressions-self: 1.97(1.19)<br>Betrayal: 2.46(1.34)		
Brady et al., 2022	Cross-sectional	377	Hospital<br>Doctors (48%)<br>Nurses (44%)<br>Radiographers (8%)	Convenience<br>January – March 2021	NR	22.3 %	90.5%	Republic of Ireland.<br>Dublin.	6%	MIES 4+ (slightly agree)<br>Cut off: 4+ (Slightly agree)<br>3 subscales (transgressions-others, transgressions-self, betrayal)	Demographics<br>Professional role - doctors (-)	Overall %<br>268 (71.1%)<br>Per subscale %<br>Transgressions-others: 197 (52.3%)<br>Transgressions-self: 92 (24.4%)<br>Betrayal: 210 (55.7%)	Overall M/SD<br>2.48(1.10)<br>Subscale M/SD<br>Transgressions-others: 3.01(1.56)<br>Transgressions-self: 1.97(1.12)<br>Betrayal: 2.81(1.44)	6	
Chandrabhatla et al., 2022	Cross-sectional		Hospitalist	Convenience<br>September 2019 – August 2020	NR	48%	NR	USA	52 %	MIES<br>Unidimensional	(Mental health):<br>Burnout (0)<br>Burnout during COVID subsample (+)	Overall %<br>60 (71.4%)*<br>Subscale %<br>Transgressions-others: 49(58.3%)**	Overall M/SD<br>2.44(1.07)*<br>Per subscale M/SD<br>Transgressions-others: 3.41(1.64)**	5	None

For peer review only

												Transgressions -self: 23(27.4)*<br>Betrayal:<br>37(44%)*<br>*N=78<br>**N=77	Transgressions -self: 1.88(1.15)***<br>Betrayal:<br>2.54(1.31)***<br>*N=73<br>**N=74<br>***N=76		
Dale et al., 2021	Longitudinal	265	Healthcare	Convenience<br>October 2020 – March 2021	37.62 (11.1)	18.1 %	77.7%	USA	NR	MIES 3 subscales (transgressions-others, transgressions-self, betrayal)	NR at univariate level	Overall %<br>NR (7 items only)<br>Subscale %<br>Transgressions -others: 6 (2.3%)<br>Transgressions -self only: 61 (23%)<br>Perpetration: 21 (7.9%)<br>Perpetration (high): 88 (33.2%)	Overall M/SD<br>NR<br>Per subscale M/SD<br>Transgressions -others: 2.54(1.45)<br>Transgressions -self: 1.66(1.05)	5	None

For peer review only

Plouffe et al., 2021<br><br>Two samples	Longitudinal (T2)	1: 803<br><br>2: 1510	Military.<br><br>Sample 1: active duty<br><br>Sample 2 - released.	Nationally representative sample.<br><br>January – May 2018 (T2)	NR	Sample 1:<br><br>87.53 %<br><br>Sample 2: 88.02 %	NR	Canada	68 %	MIES Unidimensional	NR		Overall %<br><br>NR for both samples<br><br>Subscale %<br><br>NR for both samples<br><br>Per item %<br><br>Sample 1:<br><br>Item 1: 64.6%<br><br>Item 2: 37.3%<br><br>Item 3: 16.9%<br><br>Item 4: 13.2%<br><br>Item 5: 19.9%<br>Item 6: 14.9%<br>Item 7: 48.5%<br><br>Item 8: 34.5%<br><br>Item 9: 27.5%<br><br>Sample 2:<br><br>Item 1: 66.1%<br><br>Item 2: 42.2%<br><br>Item 3: 20.4%<br><br>Item 4: 17.3%	Overall M/SD<br><br>Sample 1: 2.46(4.53)<br><br>Sample 2: 2.63(4.66)<br><br>Subscale M/SD<br><br>NR for both samples	2	None

For peer review only

												Item 5: 24.4%<br>Item 6: 21.3%<br>Item 7: 53%<br>Item 8: 37.8%<br>Item 9: 34.6%			
Nichter et al., 2021	Cross-sectional	1321	Veterans.<br>All reported exposure to combat/war zone.<br>Army (47.6%)<br>Navy (14.6%)<br>Air Force (6.3%)<br>Marines (6.3%)<br>National Guard/Reserves/Coast Guard (9.3%)	National random samples and apply post-stratification wrights to generalise to entire US veteran population.<br>2019-2020	59.1 (16.6)	93.7 %	75.1%	USA	NR	MIES 5+ (moderate) 3 subscales (transgressions-others, transgressions-self, betrayal)	NR at univariate level.	*Reports weighted % and SE<br>Overall % 535, (36.8%, 1.3%)<br>Subscale % Transgressions -Others 316, (23.1%, 1.2%)<br>Transgressions -Self 152 (11.2%, 0.9%)<br>Betrayal 189 (24.5%, 1.3%)<br>Per item<br>Item 1: 279 (21%, 1.1%)<br>Item 2: 189, (13.6%, 1.0%)<br>Item 3: 90 (6.5%, 0.7%)<br>Item 4: 88 (6.0%, 0.8%)	Overall M/SD<br>2.0(1.1)<br>Subscales M/SD<br>Transgressions -others 2.5(1.6)<br>Transgressions -self 1.7(1.1)<br>Betrayal 2.1(1.3)	1	NR

For peer review only

For peer review only - http://bmjopen.bmj.com/site/about/guidelines.xhtml

												Item 5: 77 (6.0%, 0.7%)<br>Item 6: 71 (5.9%, 0.7%)<br>Item 7: 289 (17.9%, 1.1%)<br>Item 8: 214 (14.9%, 1.0%)<br><br>Item 9: 79 (6.5%, 0.6%)			
Fitzpatrick et al., 2022	Cross-sectional.	676	Nurses.<br><br>Staff nurses (65%)<br><br>Nurse leaders (35%)	Convenience<br><br>June - August 2021.	43.88 (1.99)	8.3%	89.6%	USA	NR	MISS-HP	(Mental) health<br><br>Lower well-being (+)<br><br>Individual and/or work characteristics<br><br>Resilience (-)<br><br>Demographics<br><br>Staff nurses (+)	Overall % NR	Overall M/SD<br><br>42.21(15.73)	5	NR
Hagerty & Williams, 2022<br><br>Narrative synthesis only	Cross-sectional	1122	Healthcare<br><br>Nurse (60.4%)<br><br>Physician (5%)<br><br>Physician assistant (1.7%)<br><br>Respiratory therapist (3.6%)	Convenience<br><br>May 2020 – August 2020.	39.29	11.2 %	NR	USA	NR	MIES 4+ (slightly agree)<br><br>3 subscales (transgressions-others, transgressions-self, betrayal)	(Mental) health<br><br>Loneliness (DJGLS) (+)<br><br>Demographics<br><br>Age (-)<br><br>Access to PPE (-)<br><br>Number of COVID-19 patient deaths that respondents personally treated (+)	Overall % NR<br><br>Subscale % NR<br><br>Per item %<br><br>Item 2: 525 (47.2%)<br>Item 7: 747 (67.2%)<br>Item 4: 234 (21%)	Overall M/SD<br><br>NR<br><br>Subscale M/SD<br><br>NR	5	None

For peer review only

			Other health-care roles (28%)<br><br>Provided care in ICU during the pandemic (47.6%)												
Hamrick et al., 2022	Cross-sectional	154	Military (serving and veterans).<br><br>Former/retired (77.9%).<br><br>Army (20.8%)<br><br>Navy (52.6%)<br><br>Airforce (16.2%)<br><br>Marines (4.5%)<br><br>Coast Guard (0.6%)	Convenience<br><br>February – April 2019	36.6 (11.8)	0%	72.1%	USA	NR	EMIS-M<br><br>1 subscale (other-directed)	(Mental) health<br><br>Depression (+)<br><br>Anxiety (+)<br><br>Suicidal ideation (0)<br><br>Substance use (0)<br><br>Individual and/or work characteristics<br><br>Sexual harassment (+)<br><br>*All correlations with the other-directed MI subscale.	Overall % NR<br><br>Subscale % NR	Overall M/SD<br><br>NR<br><br>Subscale M/SD<br><br>Other-directed MI 2.66(1.17)	5	None

For peer review only

			National Guard (7.8%)<br>(Above) reserves (20.8%)<br>Other (0.6%)												
Hines et al., 2020	Cross-sectional	219	Health and social care<br>Attending physician (47%)<br>Fellow physician (18%)<br>Resident physician (20%)<br>Other (15%)	Convenience<br>March – April 2020	39.10 (11.1)	43%	NR	USA	26 %	MIES<br>2 subscales (perpetration, and betrayal).	NR at univariate level.	Overall % NR<br>Subscale % NR	Overall M/SD<br>1.79 (0.87)<br>Subscale M/SD<br>Perpetration 1.65 (0.87)<br>Betrayal 2.10 (1.28)	5	NR
Hinkel et al., 2022	Cross-sectional	59	Veterans.	Convenience<br>November 2018 – November 2019	NR	86.4 %	59.3%	USA	NR	EMIS-M<br>2 subscales (self-directed MI and other-directed MI)	(Mental) health<br>PTSD (PCL-5) (+)<br>MDD (PHQ-8) (+)<br>Chronic pain (BPI-SF) (+)	Overall % NR<br>Subscale % NR	Overall M/SD<br>NR<br>Subscale M/SD<br>Self-directed MI 2.29(1.09)	5	NR

For peer review only - http://bmjopen.bmj.com/site/about/guidelines.xhtml

													Other-directed MI 2.54(1.32)		
Kelley et al., 2021	Cross-sectional	269	Veterans.<br>Navy (45.4%)<br>Marine Corps (21.9%)<br>Army (19.7%)	Convenience	36.5 (11)	49.8 %	74.3%	USA	NR	EMIS-M<br>2 subscales (self-directed MI and other-directed MI)	(Mental) health<br>Suicidal ideation (+)<br>PTSD diagnosis (+)<br>Individual/work characteristics<br>Presence of meaning in life (MLQ) (-)<br>Searching for meaning in life (MLQ) (0)<br>Demographics<br>Veteran status (+)<br>Age (0)<br>*Both EMIS subscales	Overall % NR<br>Subscale % NR	Overall M/SD<br>NR<br>Subscale M/SD<br>Self-directed MI 1.83(0.86)<br>Other-directed MI 2.48(1.11)	5	None
La Fleur et al., 2020	Cross-sectional	212	Veterans.<br>Marine Corps (13.7%)	Convenience	NR	80.7 %	74.1%	USA	NR	MIQ-M 2.80+<br>19 items<br>Unidimensional.	(Mental) health<br>Life satisfaction (0)<br>Individual/work characteristics	Overall % 27%<br>Overall % NR	Overall M/SD<br>2.04(0.79)<br>Subscale M/SD	6	None

For peer review only - http://bmjopen.bmj.com/site/about/guidelines.xhtml

			Army (26.4%)<br><br>Navy (13.7%)<br><br>Air Force (5.7%)<br><br>Reserves (2.8%)<br><br>National Guard (4.2%)<br><br>Multiple (33.5%)								Disclosure support (0)<br><br>Disclosure concerns (+)		NR		
Lamb et al., 2021	Cross-sectional (cohort).	2166	Healthcare<br><br>Doctor (11.7%)<br><br>Nurse (29.9%)<br><br>Other clinical (26.8%)<br><br>Non-clinical (32.3%)	Convenience<br><br>April-June 2020.	NR	59.4 %	24.6%	UK	12 %	MIES 5+ (moderately agree)<br><br>Subscales: 3 subscales (commission, omission and betrayal)	Demographics<br><br>Age (-)<br><br>Sex other (+)<br><br>Relationship status divorced/separated/widowed/single (+)<br><br>Ethnicity Asian (+)<br><br>Country of birth (0)<br><br>Length of time living in UK (-)<br><br>Main role nurse (+)	*Reports weighted %<br><br>Overall %<br><br>602 (25.09%)<br><br>Subscale %<br><br>Commission: 346 (14.28%)<br><br>Omission: 111 (4.57%)<br><br>Betrayal: 439 (18.11%)	Overall M/SD<br><br>1.72 (1.19)<br><br>Subscale M/SD<br><br>Commission: 1.71 (1.06)<br><br>Omission: 1.47 (1.1)<br><br>Betrayal: 1.8 (1.14)	2	Grant.

For peer review only - http://bmjopen.bmj.com/site/about/guidelines.xhtml

Litam et al., 2021	Cross-sectional	109	Healthcare<br>Physicians (37%)<br>Nurses (57%)<br>Other professionals (6.4%)	Convenience<br>May-July 2020	37.50 (12.4)	24%	75.2%	USA	NR	MIES<br>Lower score indicates higher moral injury<br>Unidimensional	(Mental) health<br>Compassion satisfaction (PROQOL) (+)<br>Burnout (PROQOL) (+)<br>Secondary traumatic stress (PROQOL) (+)	Overall % NR<br>Subscale %<br>NR	Overall M/SD<br>3.03(0.72)<br>Subscale M/SD<br>NR	5	None.
Litz et al., 2022<br>Four studies	Cross-sectional	1:<br>533<br>2:<br>363<br>3:<br>264<br>4:<br>118	Samples 1, 2 and 3:<br>Veterans<br>Sample 4:<br>Defence members and veterans.	Sample 1:<br>Convenience<br>Samples 2, 3 and 4:<br>NR	Sample 1:<br>51.87 (9.77)<br>Samples 2, 3 and 4:<br>NR	Sample 1:<br>71%<br>Sample 2:<br>73.6 %<br>Sample 3:<br>67%<br>Sample 4:<br>65.9 %	NR for all	Sample 1:<br>Canada<br>Sample 2:<br>USA<br>Sample 3:<br>UK<br>Sample 4:<br>Australia	NR for all	MIOS<br>2 subscales (shame-related outcomes and trust violation-related outcomes)	NR at univariate level.	Sample 1:<br>Overall %<br>366 (68.7%)<br>Subscale %<br>MI-Self: 265 (49.7%)<br>MI-Other: 378 (71%)<br>Impact from an MI-Other: 440 (82.5%)<br>Sample 2:<br>Overall %<br>283 (78%)<br>Subscale %	Sample 1:<br>Overall M/SD<br>25.31(11.38)<br>Subscale M/SD<br>Shame-related outcomes: 11.28(6.64)<br>Trust violation outcomes:<br>14.03(5.85)<br>Sample 2:<br>Overall M/SD<br>25.14(11.36)<br>Subscale M/SD	5<br>4:<br>6	None

For peer review only - http://bmjopen.bmj.com/site/about/guidelines.xhtml

MI-Self: 142 (39%)<br><br>MI-Other: 142 (39%)<br><br>Impact from an MI-Other: 142 (39%)<br><br><u>Sample 3:</u><br><br>Overall %<br><br>264 (100%)<br><br>Subscale %<br><br>MI-Self: 174 (65.9%)<br><br>MI-Other: 170 (64.4%)<br><br>Impact from an MI-Other: 186 (70.1%)<br><br><u>Sample 4:</u><br><br>Overall %<br><br>NR<br><br>Subscale %<br><br>MI-Self: 65 (55%)<br><br>MI-Other: 93 (79%)	Shame-related outcomes: 11.36(6.82)<br><br>Trust violation outcomes:<br><br>13.78(5.58)<br><br><u>Sample 3:</u><br><br>Overall M/SD<br><br>32.87(10.54)<br><br>Subscale M/SD<br><br>Shame-related outcomes: 16.29(6.20)<br><br>Trust violation outcomes:<br><br>16.58(5.35)<br><br><u>Sample 4:</u><br><br>Overall M/SD<br><br>27.74(10.42)<br><br>Subscale M/SD<br><br>Shame-related outcomes: 12.17(6.35)

For peer review only - http://bmjopen.bmj.com/site/about/guidelines.xhtml

												Impact from an MI-Other: 103 (87%	Trust violation outcomes: 15.56(5.52)		
Maftei & Holman (2021)	Cross-sectional	114	Physicians	Convenience April 2020	38.85 (9.82)	24.6 %	NR	Romania	1.14 %	MIES 4+ (agree) 1-5 Likert scale 3 subscales (transgressions-others, transgressions-self, betrayal)	(Mental health) Emotional impact (0) * Emotional impact (+) ** Physical impact (+) Demographics Age (0) Working experience (0) *non-covid-19 medical unit ** covid-19 medical unit	Overall % 80 (70.2%) Per subscale % Transgressions -others: 61(53.5%) Transgressions -self: 31(27.2%) Betrayal 66(57.9%) Per item % Item 1: 45 (39.5%) Item 2: 39 (34.2%) Item 3: 14 (12.3%)	Overall 2.68(0.90) Subscale M/SD Transgressions -others: 3.06(1.26) Transgressions -self: 2.20(1.05) Betrayal 3.06(1.08)	5	None

For peer review only - http://bmjopen.bmj.com/site/about/guidelines.xhtml

												Item 4: 15 (13.2%)<br>Item 5: 20 (17.5%)<br>Item 6: 22 (19.3%)<br>Item 7: 35 (30.7%)<br>Item 8: 37 (32.5%)<br>Item 9: 33 (33%)			
Maguen et al., 2022	Cross-sectional	14057	Post 9/11 veterans.<br>Army (50.5%)<br>Navy/Coast Guard (17.9%)<br>Marines (13.3%)<br>Air Force (18.3%)	Nationally representative, population-based sampling frame<br>April – August 2018	NR	82.3 %	66.2%	USA	40 %	MIES 4+ (slightly agree)<br>3 subscales (transgressions-others, transgressions-self, betrayal)	Demographics<br>Female gender (+) *<br>Female gender (-) **<br>*transgressions-others and betrayal<br>** transgressions-self	Overall %<br>7830 (55.7%)<br>Per subscale %<br>Transgressions-others<br>6059 (43.1%)<br>Transgressions-self 3064 (21.8%)<br>Betrayal<br>5651 (40.2%)	Overall M/SD<br>NR<br>Subscale M/SD<br>NR	2	None

For peer review only - http://bmjopen.bmj.com/site/about/guidelines.xhtml

Malakoutikhah et al., 2022	Cross-sectional	455	Healthcare<br>Operating room (6.3%)<br>Nursing (73.7%)<br>Anaesthesiology (8.2%)<br>Medicine (7.5%)<br>Midwifery (2.7%)<br>Nursing assistant (1.6%)	Convenience<br>September 2021 – February 2022	NR	16.9 %	NR	Iran	NR	MISS-HP 36.5+<br>Item content varied	(Mental) health<br>General health (GHQ) (+)<br>PTSD (IES-R) (+)	Overall %<br>190 (42.7%)<br>Per item %<br>Scoring strongly agree:<br>Item 2: 13 (5.1%)<br>Item 3: 5 (2%)<br>Item 4: 15 (6%)<br>Item 5: 71(28%)<br>Item 6: 37 (14.6%)<br>Item 7: 71(28%)<br>Item 8: 39 (15.5%)<br>Item 9: 4(1.6%)<br>Item 10: 1 (0.4%)<br>Item 11: 3 (1.2%)	Overall M/SD<br>35.66(12.08)	5	None.

For peer review only

Mantri et al., 2020	Cross-sectional	181	Healthcare<br>Physicians (70.7%)<br>Nurse (9.4%)<br>Other (19.9%)	Convenience<br>November 2019 – March 2020	NR	63.5 %	74.5%	USA	NR	MISS-HP 36+<br>EMIS-M<br>2 subscales (self-directed MI and other-directed MI)	(Mental) health<br>Burnout (MBI) (+)<br>Anxiety (GAD-7) (+)<br>Depression (PHQ-9) (+)<br>Individual/work characteristics<br>Religiosity (-)<br>*MISS-HP correlations	MISS-HP<br>Overall %<br>86 (47.5%)	MISS-HP<br>Overall M/SD<br>36.8(13.3)<br>EMIS-HP<br>Overall M/SD<br>2.01(0.78)<br>Subscales M/SD<br>NR	5	None
Mantri et al., 2021<br>Two samples	Cross-sectional	Sample 1: 450<br>Sample 2: 1381	Healthcare<br>Sample 1:<br>Nurse (31.8%)<br>Physician (25.8%)<br>APP (18.9%)<br>Chaplaincy (14.2%)<br>Social worker (2.9%)<br>Other (6.4%)	Convenience.<br>Sample 1:<br>April-May 2020<br>Sample 2:<br>October-November 2020	NR	Sample 1:<br>12.4 %<br>Sample 2:<br>9.5%	Sample 1:<br>88.7%<br>Sample 2:<br>93.3%	Global<br>Primarily North America (USA)	Sample 1:<br>72.8 %<br>Sample 2:<br>73.7 %	MISS-HP 36+	(Mental) health<br>Burnout emotional exhaustion (aMBI) (-)<br>Burnout depersonalisation (aMBI) (-)<br>Burnout reduced personal/professional accomplishment (aMBI) (+)<br>Individual/work characteristics<br>COVID experience (0) *<br>COVID experience (+) **	Overall %<br>Sample 1:<br>84 (18.6%)<br>Sample 2:<br>591 (42.8%)	Overall M/SD<br>Sample 1:<br>27.4(11.6)<br>Sample 2:<br>36.4(13.8)	5	None

For peer review only - http://bmjopen.bmj.com/site/about/guidelines.xhtml

			Sample 2:<br>Nurse (56.5%)<br>Physician (12%)<br>APP (19.8%)<br>Housekeeping (0.1%)<br>Food services (0.1%)<br>Interpreter (0.1%)<br>Chaplaincy (0.1%)<br>Social worker (1.3%)<br>Other (19.9%)								Religiosity (DUREL) (-)<br>Demographics<br>Age (-)<br>Sex (0)<br>Race (0)<br>Marital status (0) *<br>Marital status (+) **<br>Profession (-)<br>*sample 1<br>** sample 2				
Morris, Webb & Devlin, 2022	Cross-sectional	237	Healthcare<br>Dietetics (2.5%)<br>Education (2.5%)	Convenience	NR	34.2 %	78.9%	UK	NR	MIES 6+ (strongly agree)<br>3 subscales (transgressions-others, transgressio	(Mental) health<br>Burnout (ProQoL) (+)<br>Secondary traumatic stress (ProQoL) (+)	Overall %<br>183 (78.5%) *<br>Subscale %<br>Transgressions-others: 154 (67%) **	Overall M/SD<br>2.90 (1.41)<br>Subscale M/SD	5	None

For peer review only - http://bmjopen.bmj.com/site/about/guidelines.xhtml

For peer review only

Medical Doctor (0.8%)	ns-self, betrayal)	Compassion satisfaction (ProQoL) (-)	Transgressions -self: 79 (34.3%) **	Transgressions -others: 3.79(1.86)
Nursing (Registered) (26.3%)		Individual/work characteristics	Betrayal: 154 (66.1%) *	Transgressions -self:
Nursing (Unregistered) (46.6%)		Impact of COVID-19 (0)	*n = 233	2.13(1.47)
Occupational Therapy (5.1%)		Demographics	**n = 230	Betrayal
Psychology (11.4%)		Gender (0)		3.38(1.83)
Psychiatry (1.3%)		Ethnicity (0)		
Recreational Co-ordinator (1.7%)		Gendered ethnicity (0)		
Social Work (0.8%)				
Other (0.4%)				

Rushton et al., 2022	Cross-sectional	595	Healthcare<br>Nurse (58.1%)<br>Physician (11.8%)<br>Respiratory therapist (6.3%)<br>Nurse practitioner/physician assistant (7.1%)<br>Other (16.7%)	Convenience<br>June 2020 – November 2020	NR	NR	82.4%	USA	NR	MISS-HP 36+	Individual/work characteristics<br>Ethical concerns (ECI) (+)<br>Involvement in COVID-19 clinical care (+)<br>Practice location (0)<br>Speciality (0)<br>Demographics<br>Years in profession (-)<br>Profession (0)<br>Education (0)<br>Religious/spiritual preference (+)	Overall %<br>32.4%	Overall M/SD<br>30.57(12.11)	5	None
Nieuwsma et al., 2020	Cross-sectional	315	Post 9/11 veterans with warzone experience.<br>Army (67%)<br>Navy (13.2%)<br>Air Force (9.9%)	Convenience	46.36 (10.4)	86.6 %	55.6%	USA	33.5 %	MIES<br>2 subscales (perpetration and betrayal)<br>MIQ-M<br>20 items<br>Unidimensional.	(Mental) health<br>Depression (BDI-2) (+)<br>Suicidal ideation (BSI) (+)<br>Alcohol use disorder (AUDIT) (+)<br>Drug abuse (DAST) (+)<br>PTSD (DTS) (+)	Overall %<br>NR<br>Subscale %<br>BMIS-E<br>137 (44.1%)<br>NR for BMIS-S subscale<br>*n = 311	Overall M/SD<br>BMIS<br>3.71(4.38)<br>MIES<br>2.57(1.26)<br>MIQ-M<br>1.49(0.47)	5	NR

For peer review only - http://bmjopen.bmj.com/site/about/guidelines.xhtml

			Marines (9.9%)							BMIS 2+ (agree)<br>2 subscales (event and sequela).	*all correlations positive for BMIS, MIES and MIQ-M		Subscale M/SD<br>NR for all scales<br>*n = 311		
Osmann et al., 2022	Cross-sectional	159	Journalists	Convenience	44.72 (12.1)	52%	NR	NR	72 %	Toronto Moral Injury Scale for Journalists<br>3 subscales (organisational, Individuals, and online)	(Mental) health<br>PTSD (PLC-5) (+)<br>Anxiety (BAI) (+)<br>Depression (BDI-II) (+)<br>Demographics<br>Marital status (0)<br>Education (0)	Overall %<br>NR<br>Subscale %<br>NR	Overall M/SD<br>1.50(1.27)<br>Subscale M/SD<br>Organisational /management: 1.54(1.32)<br>Individuals/Nonmanagement: 1.18(1.19)<br>Online: 1.68(1.27)	5	None
Rodríguez et al., 2021	Cross-sectional	169	Healthcare<br>Physicians (86.39%)<br>Nurses (13.61%)	Convenience	36.04 (10.5)	23.7 %	NR	Honduras	NR	MISS-HP 36+	(Mental) health<br>Depression (PHQ-9 (+)<br>Anxiety (GAD-7) (+)	Overall %<br>77 (45.6%)	Overall M/SD<br>34.80(15.81)	5	None

For peer review only - http://bmjopen.bmj.com/site/about/guidelines.xhtml

Roth et al., 2022	Cross-sectional	270	Personal Safety Personnel (PSP).<br>Firefighter (19%)<br>Paramedic (44%)<br>Police (17%)<br>Dispatcher (6%)<br>Other (14%)	Convenience	35 (9.5)	63%	85%	Canada and USA	NR	MIA-PSP<br>3 subscales (perpetration, betrayal and emotional sequalae)<br>MIES<br>Unidimensional.	(Mental) health<br>Shame (TRSI) (+)<br>Guilt (TRGI) (+)<br>PTSD (PCL-5) (+)<br>Depression, anxiety and stress (DASS-21) (+)<br>Dissociation (MDI) (+)<br>Disability, functioning and health (WHODAS) (+)	Overall %<br>NR for either scale	Overall M/SD<br>MIA-PSP<br>59.6(21.9)<br>MIES<br>3.56(1.39)<br>Subscale M/SD<br>MIA-PSP<br>Emotional Sequalae: 25(9.7)<br>Perpetration: 15(8.5)<br>Betrayal: 19.6(7.7)<br>Subscale M/SD<br>MIES<br>NR	5	None

For peer review only - http://bmjopen.bmj.com/site/about/guidelines.xhtml

Russell & Mussap, 2022<br><br>Two studies	Cross-sectional	Study 1:<br>183<br>Study 2:<br>393	Study 1:<br>Current and former military personnel.<br>Currently serving (50.3%)<br>Study 2:<br>Current and former military personnel.<br>Currently serving (39.7%)	Study 1 and 2:<br>Convenience.<br>.	Study 1:<br>43.3 (11.9)<br>Study 2:<br>44 (12)	Study 1:<br>83.6 %<br>Study 2:<br>81.4 %	Study 1:<br>84.7%<br>Study 2:<br>81.7%	Study 1:<br>Global but mainly Australian (89%)<br>Study 2:<br>Global but primarily Australian (85%)	Study 1:<br>NR<br>Study 2:<br>NR	Study 1:<br>MIES 4+ (slightly agree)<br>3 subscales (transgressions-others, transgressions-self, betrayal)<br>Study 2:<br>MIES 4+ (slightly agree)<br>3 subscales (transgressions-others, transgressions-self, betrayal)	Study 1:<br>(Mental) health<br>PTSD (PCL-M) (+)<br>Post-traumatic growth (PTGI-X) (0)<br>Personal well-being (PWI) (-)<br>Individual/work characteristics:<br>Intrusive rumination (ERRI) (+)<br>Deliberate rumination (ERRI) (+)<br>Exposure to danger/combat (EDC) (0) *<br>Exposure to danger/combat (EDC) (+) **<br>Witnessing the consequences of war (WDW) (+)<br>Demographics<br>Age (0)<br>Study 2:<br>(Mental) health	Study 1:<br>Overall %<br>132 (72.1%)<br>Subscale %<br>Transgressions-self: 97 (53%)<br>Transgressions-others: 48 (26.2%)<br>Betrayal: 99 (53.8%)<br>Study 2:<br>Overall %<br>300 (76.6%)<br>Subscale %<br>Transgressions-self: 109 (59.4%)<br>Transgressions-others: 62 (33.8%)<br>Betrayal: 112 (61.4%)	Study 1:<br>Overall M/SD:<br>2.25(1.12)<br>Subscale M/SD:<br>Transgressions-self: 2.88(1.63)<br>Transgressions-others: 1.73(1.20)<br>Betrayal: 2.54(1.45)<br>Study 2:<br>Overall M/SD:<br>2.60(1.28)<br>Subscale M/SD:<br>Transgressions-self: 3.23(1.74)<br>Transgressions-others: 2.05(1.42)<br>Betrayal: 2.9(1.58)	5	None

For peer review only - http://bmjopen.bmj.com/site/about/guidelines.xhtml

PTSD (PCL-M) (+)

Post-traumatic growth (PTGI-X) (0)

Personal well-being (PWI) (-)

Individual/work characteristics:

Threat bias (IBIP) (+)

Exposure to danger/combat (EDC) (+) ***

Exposure to danger/combat (EDC) (0) ****

Witnessing the consequences of war (WDW) (+)

Demographics

Age (0)

*Transgressions-self
**Transgressions-other and betrayal
***Transgressions-self and transgressions-others
****Betrayal

For peer review only

Saba et al., 2022	Longitudinal.<br><br>12-month time point.	1005	Veterans.<br><br>Air Force (14.53%)<br><br>Army (65.27%)<br><br>Marine Corps (9.65%)<br><br>Navy (10.55%	Convenience<br><br>February 2021.	34.83 (3.61)	91.14 %	84.58%	NR	21.3 %	MIES 4+ (slightly agree)<br><br>3 subscales (transgressions-others, transgressions-self, betrayal)	(Mental) health<br><br>PTSD (PCL-5) (+)<br><br>Depression (PHQ-9) (+)<br><br>Anxiety (GAD-7) (+)<br><br>Anger (DAR-R) (+)	Overall %<br><br>881 (87.66%)<br><br>Subscale %<br><br>Transgressions-self: 802 (79.80%)<br><br>Transgressions-others: 303 (30.15%)<br><br>Betrayal: 300 (29.85%)	Overall M/SD:<br><br>2.75(0.81)<br><br>Subscale M/SD:<br><br>Transgressions-self: 2.65(1.20)<br><br>Transgressions-others:<br><br>2.92(0.84)<br><br>Betrayal: 2.58(1.11)	5	None
Senger, Torres & Ratcliff, 2022	Cross-sectional	242	First responders.<br><br>In person sample: (n=91)<br><br>Firefighter (37.4%)<br><br>Law enforcement (20.90%)<br><br>Paramedic (2.20%)<br><br>EMT (4.40)	Convenience<br><br>January – December 2020	NR	In person sample:<br><br>97.8 %<br><br>Online sample:	Online sample:<br><br>83.30%<br><br>In person sample:<br><br>88.70%	USA	NR	MIES 4+ (slightly agree)<br><br>3 subscales (transgressions-others, transgressions-self, betrayal)	Individual/work characteristics:<br><br>Pre vs post COVID (-)<br><br>Military service (-)<br><br>Demographics<br><br>Occupation type (+)<br><br>Time in occupation (+)<br><br>Age (+)<br><br>Gender (0)<br><br>Race/ethnicity (0)<br><br>Education (0)	Overall %<br><br>98 (40.65%)<br><br>Subscale %<br><br>Transgressions-self: 51 (21.23%)<br><br>Transgressions-others: 147 (60.75%)<br><br>Betrayal: 97 (39.97%)<br><br>Per item %<br><br>Item 1: 174 (72.2%)	Overall M/SD:<br><br>2.7 (1.1)<br><br>Subscale M/SD:<br><br>Transgressions-self: 2.1 (1.3)<br><br>Transgressions-others: 3.8 (1.4)<br><br>Betrayal: 2.9 (1.5)	6	

For peer review only

Multiple (35.20%)<br><br>Online sample (n=151):<br><br>Firefighter (6%)<br><br>Law enforcement (38.40%)<br><br>Paramedic (16.60%)<br><br>EMT (5.30%)<br><br>Search and rescue worker (3.30%)<br><br>Other (1.30%)<br><br>Multiple (29.10%)	75.50 %	Marital status (+)<br><br>Income (0)<br><br>Religion (0)	Item 2: 119 (49.3%)<br><br>Item 3: 48 (19.7%)<br><br>Item 4: 56 (23.3%)<br><br>Item 5: 53 (21.8%)<br><br>Item 6: 49 (20.1%)<br><br>Item 7: 102 (42%)<br><br>Item 8: 94 (38.7%)<br><br>Item 9: 95 (39.2%)

For peer review only

Stanojević & Čartolovni, 2022	Cross-sectional	162	Nurses.<br>Palliative nurses (47.5%)<br>Oncology nurses (52.5%)	Convenience<br>January - June 2021.	NR	13%	NR	Croatia	64.8 %	MISS-HP 36+	(Mental) health:<br>Moral distress (+)<br>Individual/work characteristics:<br>Left or considered leaving clinical position (+)<br>Considering about leaving clinical position (+)<br>Demographics<br>Age (0)<br>Work experience (0)<br>Education (0)<br>Gender (0)<br>Specialisation (0)	Overall %<br>NR	Overall M/SD<br>37.63(12.19)	5	None
Testoni et al., 2022	Cross-sectional	270	Healthcare professionals.<br>Physician (65%<br>Other healthcare provider (35%)	Convenience<br>July - December 2020	44.5 (12.2)	30%	NR	Italy	NR	MIES 4+ (slightly agree)<br>3 subscales (transgressions-others, transgressions-self, betrayal)	(Mental) health:<br>Alienation (+)<br>Individual/work characteristics:<br>Self-efficacy (NYPQS) (-)<br>Humanisation patient (HTAS) (0)	Overall %<br>212 (78.5%)<br>Subscale %<br>Transgressions-self: 78 (28.9%)<br>Transgressions-others: 166 (61.5%)	Overall M/SD<br>2.62(1.18)<br>Subscale M/SD<br>Transgressions-self: 2.06(1.45)<br>Transgressions-others: 3.23(1.56)	5	None

For peer review only

											Humanisation colleague (HTAS) (-)<br>Overall humanisation (HTAS) (-)<br>Dehumanisation patient (HTAS) (+)<br>Dehumanisation colleague (HTAS) (+)<br>Overall dehumanisation (HTAS) (+)	Betrayal 166 (61.55)	Betrayal 2.96(1.48)		
Thomas, Weiss, Forkus & Contractor, 2022	Cross-sectional	496	Veterans.<br>Army (63.7%)<br>Navy (9.9%)<br>Air Force (19%)<br>Marines (6.3%)<br>Coast Guard (1.2%)	Convenience	37.80 (11.4)	70.5 %	71.1%	USA	18.8 %	MIES<br>3 subscales (transgressions-others, transgressions-self, betrayal)	(Mental) health:<br>Alcohol misuse (AUDIT) (+)<br>Individual/work characteristics:<br>Total religiosity (DUREL) (+)<br>Organisational religiosity (DUREL) (+)<br>Nonorganizational religiosity (DUREL) (0)<br>Intrinsic religiosity (DUREL) (0)	Overall %<br>NR<br>Subscale %<br>NR	Overall M/SD<br>3.19(1.40)<br>Subscale M/SD<br>NR	5	NR

For peer review only - http://bmjopen.bmj.com/site/about/guidelines.xhtml

Trifunovic-Koenig et al., 2022	Cross-sectional	156	Healthcare<br><br>Physician (16%)<br><br>Nurse (general) (33.3%)<br><br>Paediatric nurse (1.3%)<br><br>Paramedic (23.7%)<br><br>Geriatric nurse (9%)<br><br>Speech therapist (clinical linguistic) (7.1%)<br><br>Medical assistant (3.2%)<br><br>Medical student (1.3%)<br><br>Physiotherapist (0.6%)<br><br>Midwife (0.6%)	Convenience<br><br>March - June 2021	40.7 (10.2)	34.6 %	NR	German speaking	3.5 %	MISS-HP 28.5+<br><br>9 item scale	(Mental) health:<br><br>Psychological distress (+)<br><br>Physical distress (+)<br><br>Individual/work characteristics:<br><br>Colleague support (+)<br><br>Supervisor support (+)<br><br>Institutional support (+)<br><br>Professional self-efficacy (+)<br><br>Turnover intentions (+)<br><br>Absenteeism (+)<br><br>Resilience (+)	Overall %<br><br>85 (54.5%)	Overall M/SD<br><br>32.31(13.26)	5	None

For peer review only

			Respiratory therapist (1.3%)<br>Administration worker/Technician (0.6%)												
Ulusoy & Çelik 2022	Cross-sectional	124	Healthcare workers.<br>Doctors (67.7%)<br>Nurses (21.8%)<br>Medical secretaries (4.8%)<br>Other support staff (5.6%)	Convenience<br>January - April 2021	NR	NR	NR	Turkey	36 %	MIES 4+ (slightly agree)<br>Unidimensional	(Mental) health<br>Burnout emotional exhaustion (MBI) (+)<br>Burnout depersonalisation (MBI) (+)<br>Burnout reduced personal accomplishment (MBI) (0)<br>Obstruction to valued living (VQ) (+)<br>Depression, anxiety, and stress (DASS-21) (+)<br>Psychological inflexibility and experiential avoidance (AAQ-II) (+)	Overall %<br>116 (93.5%)<br>Subscale %<br>Transgressions -self: 78 (62.9%)<br>Transgressions -others: 109 (87.9%)<br>Betrayal: 96 (77.4%)	Overall M/SD<br>3.42(0.99)<br>Subscale M/SD<br>Transgressions -self: 2.82(1.31)<br>Transgressions -others: 4.51(1.34)<br>Betrayal: 3.51(1.28)	5	None

For peer review only - http://bmjopen.bmj.com/site/about/guidelines.xhtml

Williamson Murphy & Greenberg, 2022	Cross-sectional	90	Veterinary	Convenience<br>December 2020 – May 2021	36.8 (10.2)	12.2 %	70%	UK	27 %	MIES 5+ (moderately agree)<br>3 subscales (transgressions-others, transgressions-self, betrayal)	(Mental) health<br>PTSD (ITQ) (+)<br>Alcohol (AUDIT-C) (0)<br>Common mental disorders (PHQ-4)) (0)<br>Demographics<br>Age (0)<br>Years of experience (0)<br>Role type (0)	Overall %<br>80 (88.9%)<br>Subscale %<br>Transgressions-self: 51 (56.7%)<br>Transgressions-others: 63 (70%)<br>Betrayal: 62 (68.9%)	Overall M/SD<br>NR<br>Subscale M/SD<br>NR	5	None
Zahiriharsini et al., 2022<br>Narrative synthesis	Cross-sectional	572	Healthcare workers and leaders<br>Nurse (60.5%)<br>Patient support worker (2.9%)<br>Physician (1%)<br>Manager (11.1)	Convenience<br>February - June 2021	40.30 (10.5)	11.4 %	NR	Canada Quebec	NR	EMIS-M low MI: <2.5, moderate MI: 2.5 ≤ score <3.5, and high MI: ≤ 3.5<br>2 subscales (Self-directed MI and other-directed MI)	NR at univariate level	Overall %<br>Moderate to high: 79 (13.81%)<br>Subscale %<br>Self-directed MI:<br>Moderate to high: 58 (10.14%)<br>Other-directed MI:	Overall M/SD:<br>NR<br>Subscale M/SD:<br>NR	5	None

For peer review only

			Others (25%)									Moderate to high: 167 (29.19%)			
Levi-Belz Shemesh & Zerach 2022	Cross-sectional	190	Combat veterans.<br>Infantry units (58.4%)<br>Armoured corps (13.2%)<br>Artillery corps (7.4%)<br>Navy (4.7%)<br>Military police, intelligence, or Air Force (13.6%)	Convenience<br>January - July 2018.	26 (2.61)	87%	NR	Israel	NR	MIES 4+ (slightly agree)<br>3 subscales (transgressions-others, transgressions-self, betrayal)<br>Average time since discharge 4.26(2.37)	(Mental) health<br>Depression (PHQ-8) (+)<br>Current suicide ideation (SBQ-R) (+) *<br>current suicide ideation (SBQ-R) (0) **<br>Self-disclosure (DDI) (0)<br>Individual/work characteristics<br>Combat exposure (+)<br>Time from demobilisation (0)<br>*Transgressions-self and betrayal	Overall %<br>NR<br>Per subscale %<br>Transgressions-others: 69 (36.1%)<br>Transgressions-self: 44 (23.2%)<br>Betrayal: 67 (35.2%)<br>Per item %<br>Item 1: 61 (32.1%)<br>Item 2: 47 (24.7%)	Overall M/SD<br>NR<br>Subscale M/SD<br>Transgressions-others: 2.41(1.49)<br>Transgressions-self: 1.81(1.08)<br>Betrayal: 2.02(1.06)	5	NR

For peer review only - http://bmjopen.bmj.com/site/about/guidelines.xhtml

For peer review only

											**Transgressions-others	Item 7: 52 (27.4%)			
Schwartz Halperin & Levi-Belz, 2022	Cross-sectional	336	Veterans<br><br>Armoured corps (9.2%)<br><br>Artillery corps (8.3%)<br><br>Combat engineering (8%)<br><br>Other (34.6%)	Convenience<br><br>August - November 2019	26.16 (3.06)	83.9 %	NR	Israel	58 %	MIES 4+ (slightly agree)<br><br>1 subscale (transgressions-self)<br><br>Average time since discharge 4.2(2.45)	(Mental) health<br><br>Shame (TRSI) (+)<br><br>Current suicidal ideation (SBQ-R) (+)<br><br>Individual/work characteristics<br><br>Collective hatred (HAS) (0)<br><br>All correlations are for transgressions-self subscale.	Overall %<br><br>NR<br><br>Per subscale %<br><br>NR<br><br>Per item %<br><br>Item 3: (24.18%)	Overall M/SD<br><br>NR<br><br>Subscale M/SD<br><br>Transgressions-self: 2.12(1.26)	5	None

Note: For longitudinal studies, data extracted from baseline wave unless only available at a subsequent wave. Associations reported use total scale but if no total scale associations are reported, then subscales associations were reported.CPS; Child Protection Services, RR; Response rate, NR, No response, MIES; Moral injury events scales, EMIS-M; Expressions of Moral Injury Scale-Military Version; MISS-HP; Moral Injury Symptoms Scale – Health Professionals, MIA-PSP; Moral Injury Assessment for Public Safety Personnel, MIQ-M; Moral Injury Questionnaire—Military version, BMIS; Brief Moral Injury Screen, BMIS-E; Brief Moral Injury Screen Event subscale; EMIS-SF; Expressions of Moral Injury Scale-Short Form, MIQ-T; Moral Injury Questionnaire—Teacher version, MIQ-M; Moral Injury Questionnaire—Military version, MIOS; Moral Injury Outcome Scale

For peer review only - http://bmjopen.bmj.com/site/about/guidelines.xhtml

PRISMA 2020 Checklist

Section and Topic	Item #	Checklist item	Location where item is reported
**TITLE**			
Title	1	Identify the report as a systematic review.	1
**ABSTRACT**			
Abstract	2	See the PRISMA 2020 for Abstracts checklist.	2
**INTRODUCTION**			
Rationale	3	Describe the rationale for the review in the context of existing knowledge.	4-6
Objectives	4	Provide an explicit statement of the objective(s) or question(s) the review addresses.	6
**METHODS**			
Eligibility criteria	5	Specify the inclusion and exclusion criteria for the review and how studies were grouped for the syntheses.	1 & 11
Information sources	6	Specify all databases, registers, websites, organisations, reference lists and other sources searched or consulted to identify studies. Specify the date when each source was last searched or consulted.	7-8
Search strategy	7	Present the full search strategies for all databases, registers and websites, including any filters and limits used.	8 & Supp 1
Selection process	8	Specify the methods used to decide whether a study met the inclusion criteria of the review, including how many reviewers screened each record and each report retrieved, whether they worked independently, and if applicable, details of automation tools used in the process.	8
Data collection process	9	Specify the methods used to collect data from reports, including how many reviewers collected data from each report, whether they worked independently, any processes for obtaining or confirming data from study investigators, and if applicable, details of automation tools used in the process.	8
Data items	10a	List and define all outcomes for which data were sought. Specify whether all results that were compatible with each outcome domain in each study were sought (e.g. for all measures, time points, analyses), and if not, the methods used to decide which results to collect.	8
	10b	List and define all other variables for which data were sought (e.g. participant and intervention characteristics, funding sources). Describe any assumptions made about any missing or unclear information.	8
Study risk of bias assessment	11	Specify the methods used to assess risk of bias in the included studies, including details of the tool(s) used, how many reviewers assessed each study and whether they worked independently, and if applicable, details of automation tools used in the process.	9
Effect measures	12	Specify for each outcome the effect measure(s) (e.g. risk ratio, mean difference) used in the synthesis or presentation of results.	8
Synthesis methods	13a	Describe the processes used to decide which studies were eligible for each synthesis (e.g. tabulating the study intervention characteristics and comparing against the planned groups for each synthesis (item #5)).	7
	13b	Describe any methods required to prepare the data for presentation or synthesis, such as handling of missing summary statistics, or data conversions.	8
	13c	Describe any methods used to tabulate or visually display results of individual studies and syntheses.	9-10
	13d	Describe any methods used to synthesize results and provide a rationale for the choice(s). If meta-analysis was performed, describe the model(s), method(s) to identify the presence and extent of statistical heterogeneity, and software package(s) used.	9-10
	13e	Describe any methods used to explore possible causes of heterogeneity among study results (e.g. subgroup analysis, meta-regression).	9-10
	13f	Describe any sensitivity analyses conducted to assess robustness of the synthesized results.	10
Reporting bias assessment	14	Describe any methods used to assess risk of bias due to missing results in a synthesis (arising from reporting biases).	9
Certainty assessment	15	Describe any methods used to assess certainty (or confidence) in the body of evidence for an outcome.	9-10

For peer review only - http://bmjopen.bmj.com/site/about/guidelines.xhtml

PRISMA 2020 Checklist

Section and Topic	Item #	Checklist item	Location where item is reported
**RESULTS**			
Study selection	16a	Describe the results of the search and selection process, from the number of records identified in the search to the number of studies included in the review, ideally using a flow diagram.	11
	16b	Cite studies that might appear to meet the inclusion criteria, but which were excluded, and explain why they were excluded.	11
Study characteristics	17	Cite each included study and present its characteristics.	Supp 26-103
Risk of bias in studies	18	Present assessments of risk of bias for each included study.	Supp 2-7
Results of individual studies	19	For all outcomes, present, for each study: (a) summary statistics for each group (where appropriate) and (b) an effect estimate and its precision (e.g. confidence/credible interval), ideally using structured tables or plots.	16-21 & supp 8-22
Results of syntheses	20a	For each synthesis, briefly summarise the characteristics and risk of bias among contributing studies.	11
	20b	Present results of all statistical syntheses conducted. If meta-analysis was done, present for each the summary estimate and its precision (e.g. confidence/credible interval) and measures of statistical heterogeneity. If comparing groups, describe the direction of the effect.	16-21 & supp 8-22
	20c	Present results of all investigations of possible causes of heterogeneity among study results.	16-21 & supp 8-22
	20d	Present results of all sensitivity analyses conducted to assess the robustness of the synthesized results.	18-21
Reporting biases	21	Present assessments of risk of bias due to missing results (arising from reporting biases) for each synthesis assessed.	16-21 & supp 8-22
Certainty of evidence	22	Present assessments of certainty (or confidence) in the body of evidence for each outcome assessed.	16-21 & supp 8-22
**DISCUSSION**			
Discussion	23a	Provide a general interpretation of the results in the context of other evidence.	23-28
	23b	Discuss any limitations of the evidence included in the review.	24-25
	23c	Discuss any limitations of the review processes used.	24-25
	23d	Discuss implications of the results for practice, policy, and future research.	27-28
**OTHER INFORMATION**			
Registration and protocol	24a	Provide registration information for the review, including register name and registration number, or state that the review was not registered.	7
	24b	Indicate where the review protocol can be accessed, or state that a protocol was not prepared.	7
	24c	Describe and explain any amendments to information provided at registration or in the protocol.	7
Support	25	Describe sources of financial or non-financial support for the review, and the role of the funders or sponsors in the review.	29
Competing interests	26	Declare any competing interests of review authors.	29
Availability of data, code and other materials	27	Report which of the following are publicly available and where they can be found: template data collection forms; data extracted from included studies; data used for all analyses; analytic code; any other materials used in the review.	30

For peer review only

PRISMA 2020 Checklist

From: Page MJ, McKenzie JE, Bossuyt PM, Boutron I, Hoffmann TC, Mulrow CD, et al. The PRISMA 2020 statement: an updated guideline for reporting systematic reviews. BMJ 2021;372:n71. doi: 10.1136/bmj.n71
For more information, visit: http://www.prisma-statement.org/

For peer review only

BMJ Open

A systematic review and meta-analysis on the prevalence of occupational moral injury and post-traumatic embitterment disorder

Journal:	*BMJ Open*
Manuscript ID	bmjopen-2023-071776.R1
Article Type:	Original research
Date Submitted by the Author:	17-Oct-2023
Complete List of Authors:	Brennan, Chloe; University of Liverpool, Psychology<br>Roberts, Carl; Institute of Psychology, Health and Society, University of Liverpool, UK, Department of Psychological Sciences,<br>Cole, Jon; University of Liverpool
**Primary Subject Heading**:	Epidemiology
Secondary Subject Heading:	Mental health, Health policy, Global health, Epidemiology
Keywords:	OCCUPATIONAL & INDUSTRIAL MEDICINE, Systematic Review, EPIDEMIOLOGY, MENTAL HEALTH, PSYCHIATRY

SCHOLARONE™
Manuscripts

I, the Submitting Author has the right to grant and does grant on behalf of all authors of the Work (as defined in the below author licence), an exclusive licence and/or a non-exclusive licence for contributions from authors who are: i) UK Crown employees; ii) where BMJ has agreed a CC-BY licence shall apply, and/or iii) in accordance with the terms applicable for US Federal Government officers or employees acting as part of their official duties; on a worldwide, perpetual, irrevocable, royalty-free basis to BMJ Publishing Group Ltd ("BMJ") its licensees and where the relevant Journal is co-owned by BMJ to the co-owners of the Journal, to publish the Work in this journal and any other BMJ products and to exploit all rights, as set out in our licence.

The Submitting Author accepts and understands that any supply made under these terms is made by BMJ to the Submitting Author unless you are acting as an employee on behalf of your employer or a postgraduate student of an affiliated institution which is paying any applicable article publishing charge ("APC") for Open Access articles. Where the Submitting Author wishes to make the Work available on an Open Access basis (and intends to pay the relevant APC), the terms of reuse of such Open Access shall be governed by a Creative Commons licence – details of these licences and which Creative Commons licence will apply to this Work are set out in our licence referred to above.

Other than as permitted in any relevant BMJ Author's Self Archiving Policies, I confirm this Work has not been accepted for publication elsewhere, is not being considered for publication elsewhere and does not duplicate material already published. I confirm all authors consent to publication of this Work and authorise the granting of this licence.

For peer review only

A systematic review and meta-analysis on the prevalence of occupational moral injury and post-traumatic embitterment disorder

Chloe J. Brennan[1], Carl A. Roberts[1], Jon C. Cole[1]

[1] Department of Psychology, University of Liverpool, United Kingdom

For peer review only

Keywords: Mental health, Psychiatry, Occupational & Industrial Medicine, Systematic review, Epidemiology

Word count: 5904

[1] **Correspondence to**
Chloe Brennan
Eleanor Rathbone Building
Bedford Street South
Liverpool
L69 7ZT
Telephone: +44 (0)151 794 2000
cbrennan@liverpool.ac.uk

For peer review only - http://bmjopen.bmj.com/site/about/guidelines.xhtml

Abstract:

Objectives Occupational moral injury and post-traumatic embitterment disorder (PTED), describe the psychological distress caused by exposure to unjust at work. This is the first meta-analysis to pool prevalence rates of occupational moral injury and PTED.

Design A systematic review and meta-analysis.

Data sources Google Scholar, PubMed, APA PsycINFO, Web of Science Core Collection, Scopus, ScienceDirect and Sage Journals Online were searched in June 2020 and updated in November 2022.

Eligibility criteria for selecting studies Observational studies that measured prevalence or average scores of moral injury, or PTED in any occupational group and any geographical location.

Data extraction and synthesis Two independent reviewers screened and coded eligible studies. Study design, participant demographics, sampling method, location, measurement tool and prevalence or average scores were extracted. Risk of bias was assessed using the Quality Assessment Checklist for Prevalence Studies tool. Meta-analysis was conducted using random effects models. Results that could not be combined were summarised qualitatively in a narrative synthesis using the Guidance for Systematic Reviews.

Results In total, 88 studies across armed forces and veterans, healthcare, first responders, educators, journalists, child protection service employees, the unemployed, public-sector employees and mixed occupations were included. Studies included in each separate meta-analysis based on the measure used ranged from two to 30. The pooled prevalence of clinically relevant moral injury in healthcare professionals was 45%, and exposure to any potentially morally injurious event (PMIE) across occupations was 67%. Exposure to transgressions by others and betrayal based morally injurious events were significantly lower

For peer review only

in the armed forces than civilian occupations. Pooled prevalence of PTED across occupations was 26%.

Conclusion Exposure to PMIEs, moral injury symptoms, and PTED are prevalent at work and exposure to transgressions by others and betrayal events are more likely in civilian occupations than the armed forces.

PROSPERO registration number CRD42020191766.

Strengths and limitations:

- Guidelines from the Preferred Reporting Items for Systematic Reviews and Meta-Analyses Protocols (PRISMA) were used to prepare this review.
- The Joanna Briggs Institute (JBI) methodology for prevalence and incidence studies was used to develop the eligibility criteria, a search strategy, data extraction and risk of bias assessment.
- A comprehensive computer literature search of seven databases (Google Scholar, PubMed, APA PsycINFO, Web of Science Core Collection, Scopus, ScienceDirect and Sage Journals Online) was run in June 2020 and updated in November 2022.
- There was high heterogeneity amongst studies, even after employing subgroup analyses so findings must be interpreted with caution.
- Only peer-reviewed articles written in English were included which may lead to publication bias.

Introduction:

Exposure to traumatic events at work is commonplace, and meta-analyses have identified certain occupational groups that do not have special training to deal with traumatic events,[1] are at a higher risk of developing work-related post-traumatic stress disorder (PTSD).[1, 2] Individuals may also experience morally traumatic events at work, through

For peer review only

interpersonal conflicts, making challenging ethical decisions, and exposure to events that contradict what is ‘right’.[3-5] Moral injury and post-traumatic embitterment disorder (PTED) are two concepts that have emerged to explain the harm caused by exposure to unjust and potentially morally injurious events (PMIEs).

Moral injury refers to the psychological distress caused by exposure to PMIEs that involve perpetrating, witnessing, or learning about acts that violate moral beliefs[4] or experiences of betrayal[5]. Traditionally, moral injury has been studied in the armed forces after witnessing the horrors of war, but in 2018 a meta-analysis showed that occupational moral injury is present in civilian occupations such as journalists, veterinarians, the police, and teachers[6]. Moral injury shares similarities in aetiology and symptomology with PTED[7, 8], which is a reactive disorder caused by exposure to an unjust event and is characterised by feelings of embitterment, helplessness, and hostility[3]. Research has shown that PTED often occurs at work, for instance, 73% of PTED patients reported the triggering event as work-related[9]. PTED is evident in occupations such as the armed forces[10, 11] , educators[12, 13], healthcare professionals[7, 14, 15], the unemployed[16, 17], and in a mix of occupations[18, 19].

Despite this, there is no clear evidence for how widespread occupational moral injury and PTED are. As far as we are aware, no meta-analyses exist which combine prevalence estimates of PTED, and current attempts to combine the prevalence of morally transgressive acts[20] and moral injury symptoms[21] rely on a narrative synthesis of military studies only. Since certain occupational groups that do not have special training to deal with traumatic events are at a higher risk of developing work-related PTSD[1], it is possible that certain occupations have a higher risk of developing work-related moral injury and PTED following ethically challenging events. To accurately estimate the prevalence of occupational moral

For peer review only

injury and PTED, and investigate whether rates differ depending on occupation, systematic methods are needed.

It is challenging to estimate the pooled prevalence of moral injury and PTED as there is no gold standard measurement approach. A review by Koenig, Pearce, and Youssef[22] identified 17 scales that measure one or two aspects of moral injury, and 5 comprehensive measures of either exposure to PMIEs, moral injury symptoms, or both. Researchers report prevalence using inconsistent cut off values, and some only report average scores. Similarly, when reporting prevalence of PTED, researchers can use the PTED diagnostic interview[23], or the PTED self-rating scale, though three different cut off values are used to indicate clinically relevant embitterment at three different levels of severity.[3] Despite variability between studies, generating pooled prevalence estimates in different occupations is valuable because it can inform preventive strategies and policy decisions.

This current systematic review and meta-analyses aims to; (i) determine the prevalence of occupational moral injury and PTED in military and civilian professions, (ii) establish whether prevalence estimates differ depending on occupation, (iii) establish whether methodological differences between studies influence prevalence estimates, and (iv) record the variables associated with occupational moral injury and PTED.

Methods:

Guidelines from the Preferred Reporting Items for Systematic Reviews and Meta-Analyses Protocols (PRISMA)[24] were used to prepare this review. The Joanna Briggs Institute (JBI) methodology for prevalence and incidence studies[25] was used to develop the eligibility criteria (Table 1), a search strategy (Supplementary Table 1), data extraction (Supplementary Table 2) and risk of bias assessment (Supplementary Table 3). This review was registered with the international prospective register of systematic reviews PROSPERO network (registration no. CRD42020191766)[26].

For peer review only - http://bmjopen.bmj.com/site/about/guidelines.xhtml

Search Strategy and Study Selection:

A computer literature search was run in June 2020 using seven databases, Google Scholar, PubMed, APA PsycINFO through the EBSCOhost interface, Web of Science Core Collection, Scopus, ScienceDirect and Sage Journals online. Articles related to PTED were included from 2003, which was when the first conceptual definition for PTED was developed[27], and articles related to moral injury were included from 2009 in line with the first comprehensive scientific definition of moral injury.[4] Search terms were used as free text terms and combined with Boolean operators so the same keywords could be replicated in each database. The search was restricted to peer-reviewed articles written in English. References were managed in EndNote (Clarivate Analytics, Philadelphia) and Excel. The searches were updated in November 2022. Details of the search are listed in Supplementary Table 1. One researcher (CB) screened all titles, abstracts, and full texts and a second reviewer (CR) screened 15% of the titles and abstracts and 100% of the full texts against the eligibility criteria. Any discrepancies over the inclusion of studies were resolved through discussion. At every stage of screening, inter-rater agreement was >90%, with Cohen's kappa >0.90, indicating almost perfect agreement.[28]

The Condition, Context, Population (CoCoPop) mnemonic[25] was used to determine inclusion and exclusion criteria and are outlined in Table 1. Studies were included if they measured the prevalence or average scores of PTED or moral injury (including PMIEs) using the PTED diagnostic interview,[23] PTED self-rating scale,[3] or a comprehensive and standardised measure of moral injury. Studies included in this review measured moral injury as a whole construct rather than measuring the separate theoretical elements, such as guilt and self-forgiveness (i.e., see [22] for a review). For inclusion, moral injury and PTED must have been measured in an occupational group, in relation to work, or in unemployed or retired samples. Any working age, sex, ethnicity, country, educational status, and socio-demographic

For peer review only

For peer review only - http://bmjopen.bmj.com/site/about/guidelines.xhtml

status was included. Secondary outcomes of interest were univariate associates of moral injury or PTED. The review included observational studies (i.e., cross-sectional, retrospective, prospective, cohort and longitudinal) and excluded intervention studies or reviews. Only peer-reviewed articles written in English were eligible. When multiple papers were identified using the same data, the most relevant paper was included.

Data extraction

The following data was extracted; author and year published, study design, number of participants, mean age, the proportion of males, proportion of white ethnicity, socio-economic status, geographical location, occupation, length of time in the job, sampling method including setting and dates, the measurement tool for moral injury or PTED, prevalence (N and proportion), average scores (Mean and SD), and any conflict of interest. Secondary outcomes included univariate associates of moral injury and PTED including the variable name, the measure used, and the direction of effect (positive, negative or no association). For longitudinal and cohort studies, prevalence estimates or average scores from the baseline wave were extracted unless only subsequent waves were reported. CB contacted the authors to obtain relevant information that was not available in the text. One researcher (CB) completed the data extraction, and a second reviewer (CR) screened 100% of the data extraction to check for accuracy.

Table 1.
Inclusion and exclusion criteria

Inclusion criteria	
Condition (outcome of interest)	• Studies measuring the prevalence (%) or average scores (M and SD) of PTED, including chronic embitterment.<br>• Studies measuring prevalence (%) or average scores (M and SD) of moral injury.<br>• Uses PTED diagnostic interview, PTED self-rating scale, or a comprehensive moral injury measure.
Context	• All geographical locations.
Population	• PTED or moral injury must have been measured in relation to occupation including studies reporting on multiple occupations, the unemployed or retired.<br>• 18+, any sex, ethnicity, educational status, or socio-economic status.

For peer review only - http://bmjopen.bmj.com/site/about/guidelines.xhtml

Exclusion criteria	
Condition	• Studies using the Bern Embitterment Inventory (BEI) to measure PTED.<br>• Studies which only measured theoretical subcomponents or limited dimensional scales of moral injury.
Context	• Clinical patients in treatment or intervention studies.
Population	• Student only samples.<br>• Entirely clinical samples or all exposed to a specific stressor i.e., bullied at work, all exposed to a moral transgression, all injured at work etc.

PTED; post-traumatic embitterment disorder

Quality assessment

Quality assessment was conducted using the Quality Assessment Checklist for Prevalence Studies created by Hoy et al.,[29]. This tool assesses the possibility of bias in design, conduct and analysis including sample representativeness, sampling, non-response bias, data collection method, appropriateness of case definition, study instrument, and statistical analysis. Each item is coded as 0 (yes, low risk), or 1 (no, high risk) and quality assessment scores were summed, and categorised into low (0-3), moderate (4-6) and high (7-9) risk. Two researchers (CB, CR) conducted the quality assessment, and any discrepancies were resolved through discussion (see Supplementary Table 3).

Data synthesis and analysis

We conducted the meta-analyses using R (4.2.1) with the Meta package.[30] All meta-analyses used a random effects model using the restricted maximum-likelihood estimator (REML) due to the high heterogeneity associated with observational studies.[31] Separate meta-analyses were conducted with the prevalence of PTED as the outcome, and then the prevalence of moral injury as the outcome. For PTED prevalence, three separate analyses were conducted based on a cut-off $\geq$1.6, then 2 and then 2.5 which represents different levels of clinical severity.[3] For moral injury, due to the heterogeneity in moral injury measures (see Table 2 for an overview), a separate meta-analysis was conducted for each moral injury measure and their associated subscales. Some moral injury scales do not

For peer review only

use cut-off values to estimate the prevalence and only report average scores, so additional meta-analyses were conducted with the mean of PTED and mean of moral injury scores as the outcome.

Subgroup analyses were conducted if there were sufficient studies of k ≥10[32], grouping studies based on occupation. Forest plots were generated, displaying the prevalence and 95% CIs for each estimate, the overall pooled estimate, and 95% CIs for each occupational group. Additional subgroup analyses were conducted based on study quality (low or medium risk) and on cut-off value used for moral injury prevalence meta-analyses. If a study reported the prevalence of moral injury using multiple cut-off scores in the same paper, then the most common across studies was chosen for inclusion. A random effect model with a common estimate for τ2 was used in subgroup analyses due to k≤5 in some of the subgroups.[33] In order to determine whether any of the subgroups were statistically different, a *Q*-test with $p<0.05$ was used based on the overall subgroup results. No overlap in 95% CIs was used to determine which groups were significantly different from one another.[34]

For meta-analyses that used prevalence estimates as the outcome, the Freeman-Tukey double arcsine transformation method was applied, which is effective when there are small sample sizes and extreme prevalence estimates.[35, 36] Heterogeneity was assessed using *I*2 with a cut-off of >50% suggesting high heterogeneity.[37] Significance was assessed using *x2* for Cochrane's Q ($p<0.05$). Funnel plots are not suitable to assess publication bias in meta-analyses of proportions.[38] Therefore, a sensitivity analysis was used, comparing prevalence and average scores when outliers were removed, which were extremely small or large values that fell outside of the CI of the pooled effect.[39]

Finally, where prevalence or average scores could not be pooled due to inconsistent reporting or insufficient studies (k=1), narrative synthesis was used to describe estimates. The

For peer review only - http://bmjopen.bmj.com/site/about/guidelines.xhtml

narrative synthesis also described the univariate associates of PTED and moral injury using the Guidance for Systematic Reviews.[40] Associates were grouped into demographic, mental health and individual/work variables, and the percentage of studies that reported each type of variable was calculated. Vote counting was used to determine whether the overall relationship between variables with PTED and moral injury was positive, negative or no association.

Patient and public involvement

We conducted no primary research involving patients because the systematic review focused on published literature.

Results:

Study characteristics:

The PRISMA flow chart delineates the review process (see Figure 1). Of the 81 full texts included in the review[10-19, 41-112], some reported multiple studies in the same paper, so there were 88 separate studies included in the review. Of these, six reported prevalence estimates or average scores that could not be pooled, so they were included in the narrative synthesis only. Of the 88 studies included, 10 measured PTED, one measured both PTED and moral injury, and 77 measured moral injury. The number of studies included in each separate meta-analysis based on the measure used ranged from two to 30.

The characteristics of included studies are in Supplementary Table 2 and the risk of bias information is in Supplementary Table 3. Of the 88 studies, 10 had a low risk of bias (11%) and all others were medium risk, 77 (87.5%) were cross-sectional and all others were longitudinal, and sample sizes ranged from 38 to 14,057. Despite a small number of PTED studies, there was a varied geographical distribution, with three studies being conducted in the United Kingdom (UK) and one study each conducted in Germany, Turkey, Bosnia and Herzegovina, Cyprus, Pakistan, and South Korea. Occupations also varied with samples

For peer review only - http://bmjopen.bmj.com/site/about/guidelines.xhtml

including healthcare (n = 4), educators (n = 2), mixed occupations (n = 2), the unemployed (n = 2), veterans (n = 1) and the public sector (n = 1). In contrast, most moral injury studies were conducted in the United States (US) (n = 41), and in military samples (n = 39). Health and social care workers (HSCWs) were the second biggest occupational group (n = 32), and other occupations were less represented including first responders (n = 2), educators (n = 2), journalists (n = 2), and Child Protection Services (CPS) employees (n = 1). Geographically, aside from the US, studies were conducted in the UK (n = 6), Canada (n = 5), Israel (n = 4), Australia (n = 3), Republic of Ireland (n = 2), China (n = 2), and other countries with only one study included El Salvador, Korea, Finland, Pakistan, Romania, Iran, Honduras, Italy, Croatia, German-speaking populations, and Turkey.

Table 2 shows the characteristics of the PTED, and moral injury measurement scales included in the review. All studies measuring PTED used the PTED-self rating scale, in contrast to studies measuring moral injury which included 11 distinct scales, five of which conflated PMIEs with symptoms in the same items, two scales assessed both PMIEs and symptoms but separately, and four assessed moral injury symptoms only. The most common moral injury scale was the Moral Injury Events Scale (MIES)[113] (n = 38) which conflates events and symptoms, followed by the moral injury symptom scale for healthcare professionals (MISS-HP)[92] which assesses clinically relevant symptoms of moral injury in healthcare professionals (n = 12). Most moral injury scales are occupation-specific, and there was variation in reporting regarding the number of items, scoring, the cut-off value used, and subscale factor structure endorsed across the moral injury scales.

For peer review only - http://bmjopen.bmj.com/site/about/guidelines.xhtml

Table 2
PTED and moral injury measurement scales included in the systematic review

Measure	*k*	Events or symptoms	Decision regarding classification	Subscales	Cut off value	Scale information
PTED self-rating scale[3]	11	Symptoms linked to event	Assess embitterment reactions to event(s) specified in instructions	Unidimensional	≥1.6, ≥2, ≥2.5	19 items (0-4)
MIES[113]	38	Combined	Assesses exposure to PMIEs and effects (i.e., feeling troubled by those acts).	Bryan et al.,[42] 3 factors: transgressions-others, transgressions-self, and betrayal. Nash et al.,[113] 2 factors: perpetration and betrayal. Richardson et al.,[56] 2 factors: MI-self and MI-other. Lamb et al., 2021[86] 3 factors: commission, omission, and betrayal.	4+ (slightly agree) 5+ (moderately agree) 6+ (strongly agree)b	9 items (1-6 Likert)
MIQ-M[43]	9	Combined	Assesses military specific PMIEs, and effects.	Primarily unidimensional.<br><br>Lancaster and Harris[64] 2 factors: MIQ-causes and MIQ-effect	None	19 items (1-4 Likert)
MIQ-T[44]	1	Combined	Assesses teacher specific and effects	Unidimensional	None	12 items (1-4 Likert)
MIQ-M modified[63]	1	Events and symptoms	Modified PMIE items from the original MIQ-M which conflated PMIEs and effects into PMIEs and defining characteristics	3 PMIEs subscales AoW, PCoW and LFB and 3 subscales for their defining characteristics	2+ (seldom) on each item	22 PMIEs items and 5 defining characteristics items per PMIE (1-4 Likert)
EMIS-M[45]	7	Symptoms	Assesses guilt, shame, moral concerns, self-condemnation, social withdrawal, and inability to forgive self, anger, feelings of betrayal, revenge, and disgust.	2 subscales: self-directed and other-directed	None	17 items (1-5 Likert)
EMIS-SF[112]	1	Symptoms	Assesses emotions (guilt and shame) and beliefs/attitudes (moral disgust and mistrust).	Unidimensional	None	4 items (1-5 Likert)

For peer review only - http://bmjopen.bmj.com/site/about/guidelines.xhtml

MISS-HP[92]	12	Symptoms	Assesses betrayal, guilt, shame, moral concerns, loss of trust, loss of meaning, difficulty forgiving, self-condemnation, religious struggle, and loss of religious/spiritual faith.	Unidimensional	>36a	10 items (1-10 visual analogue Likert)
MIOS[88]	4	Symptoms linked to PMIE	The symptom scale refers to the most distressing PMIE. Symptoms include self-blame, loss of faith, trust, worthiness, goodness in others, honesty, pride, self-hatred, disgust and anger.	2 subscales: Shame-related (SR) and trust violation-related (TVR)	Nonec	14 items (0-4)
BMIS[96]	1	Events and symptoms	Endorsement of at least one PMIE assessed needed to complete symptom questions	2 subscales: BMIS-event (BMIS-E) and BMIS-sequalae (BMIS-S)	2+ PMIE Symptom subscale: None	7 items (0-3).
MIA-PSP[99]	1	Combined	Assesses feeling "bothered" by PSP specific PMIEs, and symptoms (i.e., feelings of sadness, anger, self-hatred, guilt, shame, disgust, and fear).	3 subscales: perpetration, betrayal and emotional Sequalae	None	17 items (1-6)
Toronto scale for Journalists[97]	1	Combined	Assesses journalism specific PMIEs and effects (i.e., feeling troubled by those PMIEs).	3 subscales: organisational/management; individuals/Non-management, and online	None	9 items (0-4)

Note: The number of items and scoring is based on the typical use of the scale. aThe German MISS-HP (G-MISS-HP) uses a 9 item scale with a cut off >28.5[106], The Persian MISS-HP uses a cut off >36.5[91] bSome papers modified the Likert scale and used a cut off of 3+ "sometimes" and 4+ "often[51] or 3+ "agree"[68, 89] cPMIE questions precede the MIOS symptoms scale and can be quantified as endorsed (yes/no). PTED; post-traumatic embitterment disorder, MIES; moral injury events scale; EMIS-M; Expressions of Moral Injury Scale-Military Version; MISS-HP; Moral Injury Symptoms Scale – Health Professionals, MIA-PSP; Moral Injury Assessment for Public Safety Personnel, MIQ-M; Moral Injury Questionnaire—Military version, BMIS; Brief Moral Injury Screen; EMIS-SF; Expressions of Moral Injury Scale-Short Form, MIQ-T; Moral Injury Questionnaire—Teacher version, AoW; Atrocities of War, PCoW; Psychological consequences of War, LFB; Leadership Failure or Betrayal, MIOS; Moral Injury Outcome Scale

For peer review only

Prevalence meta-analyses:

Supplementary Figures 1-9 show the forest plots with pooled prevalence estimates for PTED and moral injury, split by occupation where possible (k>10).

PTED

The pooled prevalence of PTED when using a cut-off of ≥1.6 was 37% 95% CI [24.4%, 49.9%], when using a cut-off of ≥2 was 26% 95% CI [16.1%, 37.4%], and when using a cut-off of ≥2.5 was 11% 95% CI [4%, 21.3%]. No subgroup analyses were conducted due to k≤10.

Moral injury

Using the MISS-HP, the pooled prevalence of clinically relevant signs of moral injury in healthcare professionals was 45% 95% CI [36.4%, 53.5%]. Across occupations, the pooled prevalence of exposure to any PMIE on the MIES scale was 67% 95% CI [59.0%, 74.5%], exposure to perpetration based PMIEs was 20% 95% CI [1.1%, 53.7%], transgressions by self PMIEs was 31% 95% CI [24.7%, 37.5%], transgressions by others PMIEs was 48% 95% CI [38.0%, 57.5%], and betrayal based PMIEs was 46% 95% CI [39.5%, 52.6%]. Whilst not significantly different, exposure to any PMIE was lowest in first responders (58%, 95% CI [30.9%, 82.5%]) followed by the armed forces (59%, 95% CI [45.9%, 72.4%]), healthcare professionals (71%, 95% CI [60.7%, 81.0%]) and educators (88%, 95% CI [82.5%, 91.7%]). Furthermore, whilst not significantly different, exposure to transgressions by self were also lowest in first responders (21%, 95% CI [16.1%, 26.8%]), followed by CPS professionals (24%, 95% CI [11.4%, 40.2%]), the armed forces (29%, 95% CI [19.9%, 39.6%]), healthcare professionals (33%, 95% CI [23.4%, 43.9%]) and educators (43%, 95% CI [36.0%, 49.5%]).

There were significant differences in the prevalence of exposure to transgressions by others, and betrayal based PMIEs based on occupation (*p*'s<0.01). Lack of overlap in CI shows the armed forces were exposed to significantly fewer transgressions by others (31%,

For peer review only - http://bmjopen.bmj.com/site/about/guidelines.xhtml

95% CI [20.1%, 41.8%]) compared to healthcare professionals (55%, 95% CI [43.9%, 65.6%]), first responders (61%, 95% CI [54.3%, 66.9%]), educators (80%, 95% CI [74.4%, 85.3%]) and CPS professionals (92%, 95% CI [78.6%, 98.3%]), though first responders, educators and CPS professionals included only one study each. Furthermore, the armed forces were exposed to significantly fewer betrayal events (33%, 95% CI [26.3%, 39.4%]) compared to healthcare workers (58%, 95% CI [51.0%, 65.1%]) and educators (68%, 95% CI [61.3%, 74.0%]). Whilst not significantly different, first responders had higher estimates than the armed forces (52%, 95% CI [35.2%, 69.1%]), and those in the CPS had the lowest exposure to betrayal, but this was based on only one study (32%, 95% CI [17.5%, 48.7%]).

Mean scores meta-analyses:

Supplementary Figures 10-24 show the forest plots with pooled mean estimates for PTED and moral injury, split by occupation where possible (k>10).

PTED

The pooled mean for the PTED scale across occupations was 1.52, and this was not significantly different according to occupation due to wide confidence intervals.

Moral injury

Since most moral injury scales only report average scores (i.e., Mean and SD) and not prevalence estimates, pooled mean estimates for the Moral Injury Questionnaire—Military version (MIQ-M), Expressions of Moral Injury Scale (EMIS), Moral Injury Outcome Scale (MIOS), MISS-HP and MIES were conducted. Only the pooled mean for transgressions by others on the MIES (M=3.16) significantly differed across occupations ($p<0.01$), with significantly lower scores in the armed forces (2.66, 95% CI [2.23, 3.09]) compared to first responders, educators, and CPS.

Sensitivity analyses: outliers removed

For peer review only - http://bmjopen.bmj.com/site/about/guidelines.xhtml

Table 3 shows the pooled prevalence and mean estimates, before and after removing outliers for PTED and moral injury.

Prevalence

Pooled prevalence estimates for PTED decreased when outliers were removed, using all three cut-offs. Prevalence was reduced to 31% 95% CI [23.9%, 39.2%] I2 = 84%, when using a cut off ≥1.6, reduced to 21% 95% CI [16.4%, 26.4%] I2 = 75%, when using a cut off ≥2 and decreased to 8% 95% CI [6.4%, 10.1%] I2 = 62%, when using a cut off ≥2.5.

The prevalence of moral injury in healthcare professionals on the MISS-HP was 43%, 95% CI [39.3%, 47.4%] I2 = 85%, and the prevalence of exposure to transgressions by self PMIEs decreased to 23% 95% [CI 21.6%, 24.3%] I2 = 4%. All other types of PMIEs increased, for example, exposure to any PMIE increased to 73% 95% CI [70.2%, 76.4%] I2 = 76%, transgressions by others increased to 49% 95% CI [43.8%, 54.6%] I2 = 93%, and betrayal stayed relatively similar at 46% 95% CI [41.0%, 51.3%] I2 = 93%. Except for exposure to transgressions by self, heterogeneity remained high even after removing outliers (≥50%).

Mean scores

Pooled mean estimates decreased for six of the moral injury scales or subscales after removing outliers and increased for six of the scales (see Table 3). Except for the MIOS trust subscale, heterogeneity remained high even after removing outliers (≥50%).

<u>Subgroup analyses: study quality and cut-off value</u>

Additional subgroup analyses were conducted based on the risk of bias where possible (k≥10; Supplementary Table 4). For prevalence meta-analyses, exposure to any PMIE was significantly lower in low-risk studies (47%, 95% CI [32.4, 61.9], *k*=5) compared to medium-risk (72%, 95% CI [64.8%, 79.2%], *k*=18, *p*=0.003). Furthermore, exposure to transgressions by self was significantly lower in low-risk studies (16%, 95% CI [84.7%, 25.2%], *k*=4)

For peer review only - http://bmjopen.bmj.com/site/about/guidelines.xhtml

compared to medium-risk (35%, 95% CI [28.3%, 41.8%], *k*=22, *p*=0.006), and exposure to betrayal PMIEs was significantly lower in low-risk studies (30%, 95% CI [18.5%, 43.7%], *k*=5) compared to medium-risk studies (50%, 95% CI [43.1%, 56.3%], *k*=23, p=0.011). The pooled prevalence estimates of transgressions by others were not significantly different based on the risk of bias (*p*=0.254). When comparing pooled mean estimates based on the risk of bias, only the pooled mean for betrayal was significantly lower in low-risk studies (2.03, 95% CI [1.33, 2.73], *k*=3) compared to medium-risk (2.83, 95% CI [2.60, 3.06], *k*=27, p=0.034). For the prevalence meta-analyses, different cut-off values were used to signify moral injury on the MIES and the MISS-HP (see Table 2). Therefore, additional subgroup analyses based on the cut-off value were conducted (see Supplementary Table 5) and found no significant differences in pooled prevalence estimates depending on the cut-off value (p's>0.05).

Table 3.
Pooled prevalence and mean estimates for PTED and moral injury, with and without outliers

Measure	With outliers			Without outliers		
	k	Pooled % [95% CI]	I2	*k*	Pooled % [95% CI]	I2
PTED 1.6	9	36.65 [24.38, 49.85]	98%	6	31.27 [23.87, 39.17]	84%
PTED 2	8	26.03 [16.09, 37.38]	97%	7	21.21 16.40, 26.44]	75%
PTED 2.5	6	22.31 [4.02, 21.32]	98%	7	8.16 [6.37, 10.14]	62%
MIES any	23	67 [59.01, 75.54]	98%	11	73.36 [70.18, 76.42]	76%
MIES transgressions-self	27	30.95 [24.72, 37.54]	99%	11	22.99 [21.62, 24.32]	4%
MIES transgressions-others	25	47.73 [38.04, 57.51]	98%	14	49.20 [43.82, 54.59]	93%
MIES betrayal	28	46.03 [39.54, 52.59]	98%	11	46.12 [40.97, 51.32]	91%
MIES perpetration	2	20.26 [1.11, 52.70]	98%		No outliers	
MISS-HP	10	44.88 [36.40, 53.50]	98%	7	43.33 [39.30, 47.41]	85%
	k	Pooled mean [95% CI]	I2	*k*	Pooled mean [95% CI]	I2

For peer review only - http://bmjopen.bmj.com/site/about/guidelines.xhtml

PTED	11	1.52 [1.10, 1.93]	99%	7	1.33 [1.18, 1.49]	97%
MIES total	29	2.58 [2.35, 2.82]	99%	16	2.61 [2.52, 2.70]	86%
MIES transgressions-self	27	2.26 [1.99, 2.52]	99%	12	2.16 [2.95, 2.28]	77%
MIES transgressions-others	24	3.16 [2.81, 3.52]	99%	10	3.05 [2.89, 3.20]	83%
MIES betrayal	30	2.74 [2.51, 2.98]	99%	15	2.77 [2.67, 2.86]	80%
MIES perpetration	5	2.55 [2.09, 3.02]	99%	4	2.77 [2.60, 2.95]	83%
MIQ-M total	5	1.77 [1.58, 1.96]	97%	4	1.84 [1.66, 2.02]	91%
MIQ-M causes	4	1.42 [1.21, 1.60]	96%	3	1.31 [1.26, 1.36]	75%
EMIS total	4	2.22 [1.90, 2.54]	99%		No outliers	
EMIS self	4	1.96 [1.68, 2.24]	92%		No outliers	
EMIS others	5	2.51 [2.28, 2.74]	93%	4	2.61 [2.46, 2.76]	55%
MISS-HP	12	36.73 [33.39, 40.07]	99%	7	35.42 [34.14, 39.87]	76%
MIOS total	4	27.75 [24.19, 31.31]	95%	3	25.88 [24.45, 27.20]	66%
MIOS shame	4	12.77 [10.43, 15.12]	98%	3	11.42 [11.01, 11.83]	0%
MIOS trust	4	14.97 [13.65, 16.28]	94%		No outliers	

PTED; post-traumatic embitterment disorder, MIES; moral injury events scale; EMIS; Expressions of Moral Injury Scale; MISS-HP; Moral Injury Symptoms Scale – Health Professionals, MIQ-M; Moral Injury Questionnaire—Military version, MIOS; Moral Injury Outcome Scale

For peer review only

Narrative synthesis:

Prevalence and mean scores

Supplementary Table 6 shows the prevalence and average scores for moral injury scales that could not be combined (*k*=1) including different variations of MIES subscales (i.e., MI-other; 20%, MI-commission; 14%, MI-omission; 5%), or moral injury profiles based on the MIES (no moral distress; 42%, moral distress-other; 19%, witnessing-only; 16%, moral distress-self; 8%, moral distress-self and other; 15%). Variations of the MIQ-M include the teacher version (MIQ-T; M=1.16), or the modified version. Other moral injury scales

For peer review only - http://bmjopen.bmj.com/site/about/guidelines.xhtml

were only included in one study each, such as the Moral Injury Assessment for Public Safety Personnel (MIA-PSP; M=59.6), or the Toronto moral injury scale for journalists (M=1.5). See supplementary Table 6 for specific average scores or prevalence estimates for the associated subscales.

Univariate associates of PTED and moral injury

A narrative review was used to synthesise the associates of PTED and moral injury due to the heterogeneity of variables measured, with the exception of mental health variables which were frequently measured in moral injury studies, however, meta-analyses of this relationship have been conducted recently.[22, 114, 115]

Demographic variables:

Of the PTED studies, six (55%) measured demographic variables. Overall, there was no association with gender, a positive association with length of time in the job, and a mixed association with age, with two papers finding a positive association, and two papers finding no association. Many variables were measured less frequently across studies (i.e., k<3), such as ethnicity, professional role, hours worked, having dependents, marital status, income, and education level. Of the moral injury studies, 31 (40%) measured demographic variables. Overall, there was no association with gender, age, length of time in the job, marital status, ethnicity/race/country of birth, education, and income. Several variables were measured less frequently (i.e., k<3), such as being unemployed and having dependents, and several papers measured variables specific to the work environment such as professional role, though varied job roles preclude comparison.

Mental health variables:

Of the PTED studies, seven (64%) measured mental health variables and found positive associations with depression, stress, anxiety, existing mental health diagnoses, psychological distress, negative affect, and a negative association with positive affect and

For peer review only - http://bmjopen.bmj.com/site/about/guidelines.xhtml

employee well-being, though none of these variables were measured frequently (k<3). Of the moral injury studies, 51 (65%) measured mental health variables and found positive associations with depression, PTSD or PTSD subscales, guilt or shame, anger, anxiety, burnout or its subscales, drug and alcohol use, and a negative association to well-being. Other variables related to mental health appeared less frequently (i.e., k<3).

Individual/work characteristics:

Of the PTED studies, 10 (91%) measured individual/work characteristics. Justice beliefs were negatively associated with PTED. Other variables were measured less frequently (k<3) such as rumination, unemployment duration and appraisal, supervisory control, job demands, work attitudes, social support, self-efficacy, leadership behaviours, and personality traits such as optimism, self-esteem, and resilience, though generally PTED was associated with undesirable work factors. Of the moral injury studies, 40 (51%) measured individual/work characteristics. The most common positive association was with combat exposure. Religiosity was negatively associated with moral injury, although two papers found a positive association, turnover intentions were positively associated with moral injury, and resilience associations were mixed, showing positive, negative, and no associations with moral injury. COVID-related factors were measured across several studies, but variations in the type of COVID variables measured preclude comparison. Many variables related to personality variables, beliefs, and specific work environmental factors were measured less frequently (k<3).

Discussion

This is the first meta-analysis of the prevalence and average scores of occupational moral injury and PTED so the first aim of the paper has been met. The results showed that across occupations, 37% of staff scored above a differentiation point for elevated embitterment (≥1.6), 26% had clinically relevant embitterment (≥2), and 11% showed

For peer review only - http://bmjopen.bmj.com/site/about/guidelines.xhtml

embitterment of a clinically significant intensity with improved clinical practicability and specificity (≥2.5). Findings also showed that across occupations, 67% were exposed to at least one PMIE, 20% were exposed to a perpetration based PMIE, 31% committed a moral transgression, 48% witnessed a transgression by others, and 46% were exposed to betrayal PMIEs at work. The second aim was to establish whether prevalence estimates differ depending on occupation and this aim has been partially met because some moral injury scales were occupation specific or used different subscales meaning they could not be pooled across occupations. Despite this, exposure to transgressions by others and betrayal were significantly lower in the armed forces compared with healthcare workers and educators. Finally, clinical levels of moral injury in healthcare professionals, according to the MISS-HP, was 45%. Since several moral injury scales did not provide prevalence estimates, mean scores were also pooled, and for PTED, did not differ according to occupation. Mean scores of exposure to transgressions-by others were significantly lower in the armed forces compared to first responders, CPS, and educators.

The third aim was to determine whether methodological differences between studies influenced prevalence estimates, and this was shown by methodological differences between studies precluding some analyses. In addition, prevalence estimates for exposure to any PMIE, transgressions by self and betrayals were lower in low risk compared to medium-risk studies, and the mean scores for betrayal was also lower in low-risk studies. Furthermore, prevalence estimates for moral injury were not significantly different based on the cut-off value used by the researcher. The final aim of the systematic review was to record the variables associated with moral injury and PTED. A narrative review identified variables measured in association with moral injury and PTED which fell into three categories: demographics, mental health, and individual/work characteristics. Gender was found to have no association with moral injury and PTED, whereas the length of time in the job had a

For peer review only

positive association, and age had a positive or no association with PTED. In contrast, age, length of time in the job, marital status, ethnicity/race/country of birth, education and income all had no association with moral injury. Other demographic variables varied and were usually only included in one or two studies. Moral injury and PTED showed a positive association with mental health variables including depression, stress, anxiety, and employee well-being, and moral injury studies studied the relationship with a wider range of disorders such as PTSD and anxiety, and emotional (i.e., guilt, anger, shame), behavioural (i.e., burnout, compassion fatigue) and spiritual variables. Finally, individual/work variables differed between the two constructs, with moral injury studies focusing primarily on the combat environment as a risk for military samples and PTED focusing on justice beliefs as protective in civilian samples. Furthermore, religiosity and resilience were measured in relation to moral injury with mixed results, and turnover intentions were positively associated with moral injury. Individual risk and resiliency factors such as optimism, self-esteem and personality traits were also identified, but less frequently. Due to the infrequency of variables measured, the associations reported here are inconclusive, and this aim has only been partially met.

The results of this study should be considered in light of several strengths and limitations. The study protocol was pre-registered on Prospero and followed the Joanna Briggs Institute guidance for systematic reviews of prevalence and incidence data which ensures the methods were robust.[25] In line with guidance from the Cochrane handbook[116] two researchers screened 15% of all abstracts and titles, and 100% of full texts, data extraction and quality assessment. However, using three screeners would improve objectivity. Despite using robust methods, there was high heterogeneity within individual studies, and amongst studies, even after employing subgroup analyses to explain the variability based on study quality, the cut-off score used and sensitivity analyses to remove

For peer review only - http://bmjopen.bmj.com/site/about/guidelines.xhtml

outliers. Whilst heterogeneity is often high in prevalence meta-analyses, and the median *I*2 was found to be 96.9% in a review of 134 prevalence meta-analyses[117], caution must be taken when interpreting our findings. Furthermore, only 11% of studies had a low risk of bias, and often the primary aim of the included studies was not to produce prevalence data. This may explain the use of inappropriate statistical methods (i.e., lack of confidence intervals or weighting), and variability in reporting only average scores or prevalence data based on the various subscale configurations or using different cut-off values. To deal with this heterogeneity, separate meta-analyses were conducted based on the type of data reported (i.e., prevalence or average scores), and the measure used, reducing the number of studies included in each meta-analysis and thus power, meaning that subgroup analyses based on occupation were not always possible. Finally, whilst we searched a relatively high number of databases, it is possible that references from the grey literature have been missed.

Despite the limitations, this review adds to the growing literature on the prevalence of moral injury and PTED. Prior estimates of PTED in the general population were at 2.5%[3] and this compares to 26% across occupations in this review. This confirms the findings by Linden et al.,[9] which found that 73% of PTED patients reported the triggering event as work-related and shows unjust and embittering events often occur in the workplace. Using the most stringent cut-off, occupational health services could expect 11% of their staff to display clinically severe levels of PTED according to these findings. There were not enough studies to conduct subgroup analyses based on the prevalence of PTED across occupations, though subgroup analyses using the mean PTED scores showed no significant difference based on occupation. This might suggest that PTED is widespread across professions. However, this analysis was based on only 11 studies, and the inclusion of more studies would be needed to confirm that this effect is not due to low statistical power.

For peer review only - http://bmjopen.bmj.com/site/about/guidelines.xhtml

Previous attempts to collate prevalence estimates for moral injury have relied on a narrative synthesis of the prevalence of morally transgressive acts[20] or moral injury symptoms in military samples.[21] Findings from this meta-analysis shows that 67% of people across occupations have been exposed to at least one PMIE in their jobs, which is a high proportion. Furthermore, staff were more often exposed to transgressions by others (48%) and betrayal PMIEs (46%) than PMIEs perpetrated by themselves (31%) which mirrors qualitative findings during the pandemic, which shows healthcare staff experienced institutional betrayal more than perpetration based moral injuries.[118, 119] Estimates of exposure to PMIEs are taken from the MIES,[20] which was the most widely used scale to measure moral injury across studies in this review. Whilst it is arguable that not all individuals exposed to PMIEs will experience moral injury symptoms, the MIES does confound events and effects by asking participants if they feel troubled by their exposure to moral violations.[22] This suggests that a high proportion of staff are not only exposed to events that are morally injurious, but are also affected by this. This is supported by further findings from this meta-analysis which showed that 45% of healthcare workers had clinically relevant signs of moral injury according to the MISS-HP, which is the only moral injury symptom scale which uses a clinical cut off.[92] Since the MISS-HP is healthcare specific, and the other symptom scales in this review only reported mean scores and not prevalence estimates, it is not possible to determine whether any occupations are at a greater risk of clinically relevant moral injury.

However, the review was able to show that members of the armed forces were exposed to significantly fewer transgressions by others and betrayal PMIEs than healthcare workers and educators, and the mean scores for transgressions by others were significantly lower in the armed forces than first responders, CPS, and educators. Therefore, despite the definitions of moral injury originating from the military[4, 5], this shows that civilian

For peer review only - http://bmjopen.bmj.com/site/about/guidelines.xhtml

occupations are at an even greater risk of exposure to PMIEs and of feeling troubled by these. This mirrors findings which show that certain occupations have a higher risk of developing work-related PTSD, such as professions that do not have special training to deal with traumatic events.[1] It is possible that military personnel expect to be exposed to moral traumas and are less troubled as a result.

This meta-analysis identified several methodological issues. Prevalence of exposure to any PMIE, transgressions by self and betrayals were lower in low risk compared to medium-risk studies, and the prevalence of PTED differed depending on the cut-off value used. As highlighted by Koenig, Pearce, and Youssef[22], there are several moral injury scales, which conflate events and symptoms (MIES, MIQ), and our review has identified more recently developed scales which also do this (i.e., MIA-PSP, Toronto scale for journalists). Other scales only measure symptoms (i.e., EMIS, MIOS, MISS-HP), while many scales are occupation-specific precluding comparisons across occupations, and not all scales have established cut-off values, which contributes to inconsistencies in this area. Furthermore, there is a lack of randomly selected, nationally representative, epidemiological studies on the prevalence of moral injury and PTED in specific populations of interest (i.e., the armed forces, HSCWs, first responders, educators, journalists, CPS employees) and in the general working population.

These findings reveal several implications and gaps that can form the basis of future research. First, clinically relevant signs of PTED are much higher in work settings than in the general population, and just under half of the healthcare professionals showed clinical levels of moral injury. Furthermore, occupations such as healthcare professionals, educators, and the CPS are especially likely to experience transgressions by others and healthcare professionals and educators are also more likely to experience betrayals at work than the armed forces. Understanding the prevalence of moral injury and PTED in different

For peer review only - http://bmjopen.bmj.com/site/about/guidelines.xhtml

occupations is critical for developing targeted prevention strategies and can guide policy. However, these occupations relate to statutory organisations and frontline services, and it is possible that other civilian occupations have a lower, or moderate risk of exposure to PMIEs, and moral injury. Research across a broader range of professionals to understand which civilian occupations are at low, moderate, and high risk of exposure to PMIEs, moral injury and PTED would be valuable. The narrative synthesis showed that moral injury and PTED are associated with poor mental health outcomes such as depression, stress and anxiety, and poor work outcomes such as sickness absence, and turnover intentions. Therefore, employers, occupational health, and clinicians should be aware of these conditions and provide appropriate support. Currently, there are no proven recommended treatments for moral injury and PTED, and this research shows organisations will need strategies to support employees given the high risk of experiencing moral injury and PTED. Standardised measurements of moral injury and PTED, and proven treatment options need to be developed. In addition, whilst the number of studies on moral injury has grown exponentially in recent years, research on occupational PTED still represents a significant gap, and the lack of studies precluded prevalence subgroup analyses based on occupation. More studies are needed in this area. Second, whilst there were more moral injury studies, most were conducted with military or healthcare professionals, and other occupations such as educators, journalists, CPS, and first responders were represented in only one or two studies. Prevalence estimates indicate educators and CPS employees may be at an especially high risk, and more studies are needed to confirm these findings. Finally, most moral injury studies were conducted in the US, and studies are needed across more geographical locations.

In conclusion, we are the first to provide meta-analytic evidence that the prevalence of moral injury and PTED at work are high. We identified occupations at a greater risk of exposure to PMIEs but inconsistencies in measurement scales, subscale configurations or cut-

For peer review only - http://bmjopen.bmj.com/site/about/guidelines.xhtml

off values used amongst studies precluded some analyses. To strengthen the reliability of these findings, researchers must agree on a gold standard approach to measuring moral injury and PTED, across a range of low, moderate, and high-risk occupations with appropriate cut off values. Despite this, high prevalence rates of moral injury and PTED show that proven, recommended treatment options and guidance should be developed to support employees and can inform policy makers on which areas of research and prevention to prioritise.

Figure 1.
Preferred Reporting Items for Systematic Reviews and Meta-Analyses (PRISMA) flow diagram.

Contributor and guarantor information:

CB designed the project and data collection tools, collected the data, cleaned, analysed and interpreted the data and drafted and revised the paper. She is guarantor. CR assisted with the study design, data collection and revised the paper. JC assisted with the study design and revised the paper. All authors gave final approval for the publication of this manuscript. The corresponding author attests that all listed authors meet authorship criteria and that no others meeting the criteria have been omitted.

Copyright:

The Corresponding Author has the right to grant on behalf of all authors and does grant on behalf of all authors, an exclusive licence (or non-exclusive for government employees) on a worldwide basis to the BMJ Publishing Group Ltd to permit this article (if accepted) to be published in BMJ editions and any other BMJPGL products and sublicences such use and exploit all subsidiary rights, as set out in our licence.

For peer review only - http://bmjopen.bmj.com/site/about/guidelines.xhtml

Competing interest statement:

None declared

Funding:

CB was supported by the Economic and Social Research Council UK [ES/P000665/1].

Role of sponsor:

The study sponsors did not play a role in the study design, management, data collection, analyses, in the interpretation of the data or in the writing of the manuscript or the decision to submit for publication.

Transparency declaration:

The lead author affirms that this manuscript is an honest, accurate, and transparent account of the study being reported; that no important aspects of the study have been omitted; and that any discrepancies from the study as planned have been explained.

Ethical approval:

Ethical approval is not required for this study design.

Data availability statement:

No additional data are available.

Dissemination declaration:

Results from this study will be disseminated to relevant audiences through University of Liverpool communications.

For peer review only

References:

1. Skogstad M, Skorstad M, Lie A, et al. Work-related post-traumatic stress disorder. Occupational medicine. 2013 Apr 1;63(3):175-82. https://doi.org/10.1093/occmed/kqt003

2. Lee W, Lee YR, Yoon JH, et al. Occupational post-traumatic stress disorder: an updated systematic review. BMC public health. 2020 Dec;20(1):1-2. https://doi.org/10.1186/s12889-020-08903-2

3. Linden M, Baumann K, Lieberei B, et al. The post-traumatic embitterment disorder self-rating scale (PTED scale). Clinical Psychology & Psychotherapy: An International Journal of Theory & Practice. 2009 Mar;16(2):139-47. https://doi.org/10.1002/cpp.610

4. Litz BT, Stein N, Delaney E, et al. Moral injury and moral repair in war veterans: A preliminary model and intervention strategy. Clinical psychology review. 2009 1;29(8):695-706. https://doi.org/10.1016/j.cpr.2009.07.003

5. Shay J. Moral injury. Psychoanalytic psychology. 2014 Apr;31(2):182. https://doi.org/10.1037/a0036090

6. Williamson V, Stevelink SA, Greenberg N. Occupational moral injury and mental health: systematic review and meta-analysis. The British Journal of Psychiatry. 2018 Jun;212(6):339-46. DOI: https://doi.org/10.1192/bjp.2018.55

7. Brennan CJ, McKay MT, Cole JC. Morally injurious events and post-traumatic embitterment disorder in UK health and social care professionals during COVID-19: a cross-sectional web survey. BMJ open. 2022 May 1;12(5):e054062. doi: 10.1136/bmjopen-2021-054062

8. Carter C. Organisational injustice in UK frontline services and onset of Moral Injury, Post Traumatic Embitterment Disorder (PTED) and PTSD. International

For peer review only

journal of law, crime and justice. 2021 Sep 1;66:100483 https://doi.org/10.1016/j.ijlcj.2021.100483

9. Linden M, Rotter M, Baumann K, et al. Posttraumatic embitterment disorder: Definition, evidence, diagnosis, treatment. Hogrefe & Huber Publishers; 2007.

10. Sabic D, Sabic A, Batic-Mujanovic O. Embitterment in war veterans with posttraumatic stress disorder. Medical Archives. 2018 Apr;72(2):125. doi: 10.5455/medarh.2018.72.125-130

11. Muschalla B, Rau H, Willmund GD, Knaevelsrud C. Work disability in soldiers with posttraumatic stress disorder, posttraumatic embitterment disorder, and not-event-related common mental disorders. Psychological trauma: theory, research, practice, and policy. 2018 Jan;10(1):30. https://doi.org/10.1037/tra0000293

12. Rubab M, Tariq F. Embitterment, Self-Efficacy and Mental Health Problems in private school teachers in Pakistan. PSYCHOLOGY AND EDUCATION. 2022;59(2):572-81.

13. Saleem A, Bashir M, Abrar M. Ethical behaviors by leaders act as a stimulant to the wellbeing of employees by restraining workplace embitterment. Frontiers in Public Health. 2022 Sep 29;10:974642. DOI: 10.3389/fpubh.2022.974642

14. Dunn JM, Sensky T. Psychological processes in chronic embitterment: The potential contribution of rumination. Psychological Trauma: Theory, Research, Practice, and Policy. 2018 Jan;10(1):7. https://doi.org/10.1037/tra0000291

15. Sensky T, Salimu R, Ballard J, et al. Associations of chronic embitterment among NHS staff. Occupational Medicine. 2015 Aug 1;65(6):431-6. https://doi.org/10.1093/occmed/kqv089

16. Shin J, You M. Embitterment among the unemployed: A multiple mediation model of belief in a just world. Work. 2022: 1-0. DOI: 10.3233/WOR-205228

For peer review only - http://bmjopen.bmj.com/site/about/guidelines.xhtml

17. Linden M, Rotter M. Unemployment and embitterment in contrast to general psychological distress. Work. 2019 Jan 1;62(1):133-8. DOI: 10.3233/WOR-182848

18. Michailidis E, Cropley M. Exploring predictors and consequences of embitterment in the workplace. Ergonomics. 2017 Sep 2;60(9):1197-206. https://doi.org/10.1080/00140139.2016.1255783

19. Michailidis E, Cropley M. Investigating the predictors of workplace embitterment using a longitudinal design. Occupational Medicine. 2018 Nov 16;68(8):523-9. https://doi.org/10.1093/occmed/kqy121

20. Frankfurt S, Frazier P. A review of research on moral injury in combat veterans. Military psychology. 2016 Jun 9;28(5):318-30. https://doi.org/10.1037/mil0000132

21. Koenig HG, Al Zaben F. Moral injury: An increasingly recognized and widespread syndrome. Journal of religion and health. 2021 Oct;60(5):2989-3011. https://doi.org/10.1007/s10943-021-01328-0

22. Koenig HG, Youssef NA, Pearce M. Assessment of moral injury in veterans and active duty military personnel with PTSD: A review. Frontiers in psychiatry. 2019 Jun 28;10:443. https://doi.org/10.3389/fpsyt.2019.00443

23. Linden M, Baumann K, Rotter M, Schippan B. Diagnostic criteria and the standardized diagnostic interview for posttraumatic embitterment disorder (PTED). International Journal of Psychiatry in Clinical Practice. 2008 Jan 1;12(2):93-6. https://doi.org/10.1080/13651500701580478

24. Page MJ, McKenzie JE, Bossuyt PM, Boutron I, Hoffmann TC, Mulrow CD, Shamseer L, Tetzlaff JM, Akl EA, Brennan SE, Chou R. The PRISMA 2020

For peer review only

statement: an updated guideline for reporting systematic reviews. Systematic reviews. 2021 Dec;10(1):1-1. https://doi.org/10.1186/s13643-021-01626-4

25. Munn Z, Moola S, Lisy K, Riitano D, Tufanaru C. Methodological guidance for systematic reviews of observational epidemiological studies reporting prevalence and cumulative incidence data. International journal of evidence-based healthcare. 2015 Sep 1;13(3):147-53. DOI: 10.1097/XEB.0000000000000054

26. Brennan C, Roberts C. A systematic review examining the prevalence of work-related moral injury and post-traumatic embitterment disorder (PTED). PROSPERO. 2020 Jul 3. CRD42020191766. Available from: https://www.crd.york.ac.uk/prospero/display_record.php?ID=CRD42020191766

27. Linden M. Posttraumatic embitterment disorder. Psychotherapy and psychosomatics. 2003;72(4):195-202. https://doi.org/10.1159/000070783

28. McHugh ML. Interrater reliability: the kappa statistic. Biochemia medica. 2012 Oct 15;22(3):276-82.30

29. Hoy D, Brooks P, Woolf A, et al. Assessing risk of bias in prevalence studies: modification of an existing tool and evidence of interrater agreement. Journal of clinical epidemiology. 2012 Sep 1;65(9):934-9. https://doi.org/10.1016/j.jclinepi.2011.11.014

30. Balduzzi S, Rücker G, Schwarzer G. How to perform a meta-analysis with R: a practical tutorial. Evidence-Based Mental Health. 2019. p. 153–60.

31. Langan D, Higgins JP, Jackson D, Bowden J, Veroniki AA, Kontopantelis E, Viechtbauer W, Simmonds M. A comparison of heterogeneity variance estimators in simulated random-effects meta-analyses. Research synthesis methods. 2019 Mar;10(1):83-98. https://doi.org/10.1002/jrsm.1316

For peer review only - http://bmjopen.bmj.com/site/about/guidelines.xhtml

32. Schwarzer G. Meta-Analysis in R. Systematic Reviews in Health Research: Meta-Analysis in Context. 2022 Apr 22:510-34. https://doi.org/10.1002/9781119099369.ch26

33. Borenstein M, Hedges LV, Higgins JP, Rothstein HR. Introduction to meta-analysis. John Wiley & Sons; 2021 Apr 6.

34. Cumming G, Finch S. Inference by eye: confidence intervals and how to read pictures of data. American psychologist. 2005 Feb;60(2):170. https://doi.org/10.1037/0003-066X.60.2.170

35. Lipsey MW, Wilson DB. Practical meta-analysis. SAGE publications, Inc; 2001.

36. Viechtbauer W. Conducting meta-analyses in R with the metafor package. Journal of statistical software. 2010;36(3):1-48. DOI: 10.18637/jss.v036.i03

37. Higgins JP, Thompson SG. Quantifying heterogeneity in a meta-analysis. Statistics in medicine. 2002 Jun 15;21(11):1539-58. https://doi.org/10.1002/sim.1186

38. Wang N. How to conduct a meta-analysis of proportions in R: a comprehensive tutorial. New York, NY, John Jay College of Criminal Justice, 0–62. 2018 Jun.

39. Viechtbauer W, Cheung MW. Outlier and influence diagnostics for meta-analysis. Research synthesis methods. 2010 Apr;1(2):112-25. https://doi.org/10.1002/jrsm.11

40. Popay J, Roberts H, Sowden A, Petticrew M, Arai L, Rodgers M, Britten N, Roen K, Duffy S. Guidance on the conduct of narrative synthesis in systematic reviews. A product from the ESRC methods programme Version. 2006 Apr 1;1(1):b92.

41. Karatuna I, Gök S. A study analyzing the association between post-traumatic embitterment disorder and workplace bullying. Journal of Workplace Behavioral Health. 2014 Apr 3;29(2):127-42

For peer review only

42. Bryan CJ, Bryan AO, Anestis MD, et al. Measuring moral injury: Psychometric properties of the moral injury events scale in two military samples. Assessment. 2016 Oct;23(5):557-70. https://doi.org/10.1177/1073191115590855

43. Currier JM, Holland JM, Drescher K, et al. Initial psychometric evaluation of the Moral Injury Questionnaire—Military version. Clinical psychology & psychotherapy. 2015 Jan;22(1):54-63. https://doi.org/10.1002/cpp.1866

44. Currier JM, Holland JM, Rojas-Flores L, et al. Morally injurious experiences and meaning in Salvadorian teachers exposed to violence. Psychological trauma: theory, research, practice, and policy. 2013;7(1):24. https://doi.org/10.1037/a0034092

45. Currier JM, Farnsworth JK, Drescher KD, et al. Development and evaluation of the Expressions of Moral Injury Scale—Military Version. Clinical psychology & psychotherapy. 2017 May;25(3):474-88. https://doi.org/10.1002/cpp.2170

46. Forkus SR, Breines JG, Weiss NH. Morally injurious experiences and mental health: The moderating role of self-compassion. Psychological trauma: theory, research, practice, and policy. 2019 Sep;11(6):630. https://doi.org/10.1037/tra0000446

47. Frankfurt SB, DeBeer BB, Morissette SB, Kimbrel NA, La Bash H, Meyer EC. Mechanisms of moral injury following military sexual trauma and combat in post-9/11 US war veterans. Frontiers in psychiatry. 2018 Nov 2;9:520. https://doi.org/10.3389/fpsyt.2018.00520

48. Griffin BJ, Williams CL, Shaler L, Dees RF, Cowden RG, Bryan CJ, Litz B, Purcell N, Burkman K, Maguen S. Profiles of moral distress and associated outcomes among student veterans. Psychological Trauma: Theory, Research, Practice, and Policy. 2020 Oct;12(7):669. https://doi.org/10.1037/tra0000584

For peer review only - http://bmjopen.bmj.com/site/about/guidelines.xhtml

49. Jinkerson JD, Battles AR. Relationships between moral injury syndrome model variables in combat veterans. Traumatology. 2019 Mar;25(1):33. https://doi.org/10.1037/trm0000163

50. Jordan AH, Eisen E, Bolton E, Nash WP, Litz BT. Distinguishing war-related PTSD resulting from perpetration-and betrayal-based morally injurious events. Psychological Trauma: Theory, Research, Practice, and Policy. 2017 Nov;9(6):627. https://doi.org/10.1037/tra0000249

51. Lee H, Aldwin CM, Kang S. Do different types of war stressors have independent relations with mental health? Findings from the Korean Vietnam Veterans Study. Psychological trauma: theory, research, practice, and policy. 2022 Jul;14(5):840. https://doi.org/10.1037/tra0000557

52. Ogle AD, Reichwald R, Rutland JB. Psychological impact of remote combat/graphic media exposure among US Air Force intelligence personnel. Military Psychology. 2018 Nov 2;30(6):476-86. https://doi.org/10.1080/08995605.2018.1502999

53. Wisco BE, Marx BP, May CL, Martini B, Krystal JH, Southwick SM, Pietrzak RH. Moral injury in US combat veterans: Results from the national health and resilience in veterans study. Depression and anxiety. 2017 Apr;34(4):340-7. https://doi.org/10.1002/da.22614

54. Zerach G, Levi-Belz Y. Moral injury process and its psychological consequences among Israeli combat veterans. Journal of Clinical Psychology. 2018 Sep;74(9):1526-44. https://doi.org/10.1002/jclp.22598

55. Papazoglou K, Blumberg D, Briones-Chiongbian V, Russo C, Koskelainen M. Exploring the roles of moral injury and personality in police traumatization.

For peer review only

Crisis, Stress, and Human Resilience: An International Journal. 2019 Apr 25;1(1):32-56.

56. Richardson CB, Chesnut RP, Morgan NR, et al. Examining the factor structure of the moral injury events scale in a veteran sample. Military medicine. 2020 Jan;185(1-2):e75-83. https://doi.org/10.1093/milmed/usz129

57. Feinstein A, Pavisian B, Storm H. Journalists covering the refugee and migration crisis are affected by moral injury not PTSD. JRSM open. 2018 Feb;9(3):2054270418759010. https://doi.org/10.1177/2054270418759010

58. Sugrue EP. Moral injury among professionals in K–12 education. American Educational Research Journal. 2020 Feb;57(1):43-68. https://doi.org/10.3102/00028312198486

59. Aldridge V, Scott H, Paskell R. Investigating the relationship between guilt and shame proneness and moral injury in veterans that have experienced active deployment. Military Behavioral Health. 2019 Jan 2;7(1):83-91. https://doi.org/10.1080/21635781.2019.1580641

60. Andrukonis A, Protopopova A. Occupational health of animal shelter employees by live release rate, shelter type, and euthanasia-related decision. Anthrozoös. 2020 Jan 2;33(1):119-31.

61. Haight W, Sugrue EP, Calhoun M. Moral injury among child protection professionals: Implications for the ethical treatment and retention of workers. Children and Youth Services Review. 2017 Nov 1;82:27-41. https://doi.org/10.1016/j.childyouth.2017.08.030

62. Battles AR, Kelley ML, Jinkerson JD, C. Hamrick H, F. Hollis B. Associations among exposure to potentially morally injurious experiences, spiritual injury, and

For peer review only

alcohol use among combat veterans. Journal of Traumatic Stress. 2019 Jun;32(3):405-13. https://doi.org/10.1002/jts.22404

63. Braitman AL, Battles AR, Kelley ML, Hamrick HC, Cramer RJ, Ehlke S, Bravo AJ. Psychometric properties of a Modified Moral Injury Questionnaire in a military population. Traumatology. 2018 Dec;24(4):301. https://doi.org/10.1037/trm0000158

64. Lancaster SL, Harris JI. Measures of morally injurious experiences: A quantitative comparison. Psychiatry research. 2018 Jun 1;264:15-9. https://doi.org/10.1016/j.psychres.2018.03.057

65. Lancaster SL. Negative outcomes after morally injurious experiences: A replication and extension. Psychological Trauma: Theory, Research, Practice, and Policy. 2018 Jul;10(4):456. https://doi.org/10.1037/tra0000341

66. Akhtar M, Faize FA, Malik RZ, et al. Moral injury and psychological resilience among healthcare professionals amid COVID-19 pandemic. Pakistan Journal of Medical Sciences. 2022 May 18;38(5). https://doi.org/10.12669/pjms.38.5.5122

67. Qi M, Hu X, Liu J, et al. The impact of the COVID-19 pandemic on the prevalence and risk factors of workplace violence among healthcare workers in China. Frontiers in public health. 2022;10. https://doi.org/10.3389/fpubh.2022.938423

68. Amsalem D, Lazarov A, Markowitz JC, et al. Psychiatric symptoms and moral injury among US healthcare workers in the COVID-19 era. BMC psychiatry. 2021 Dec;21(1):1-8. https://doi.org/10.1186/s12888-021-03565-9

69. Perez D, Larson P, Bair J. US veterans experience moral injury differently based on moral foundations preferences. Journal of Community Engagement and Scholarship. 2021 Aug;13(4):13.

For peer review only - http://bmjopen.bmj.com/site/about/guidelines.xhtml

70. Benatov J, Zerach G, Levi-Belz Y. Moral injury, depression, and anxiety symptoms among health and social care workers during the COVID-19 pandemic: The moderating role of belongingness. International journal of social psychiatry. 2022:00207640221099421. https://doi.org/10.1177/00207640221099421

71. Borges LM, Holliday R, Barnes SM, et al. A longitudinal analysis of the role of potentially morally injurious events on COVID-19-related psychosocial functioning among healthcare providers. PloS one. 2021 Nov 12;16(11):e0260033. https://doi.org/10.1371/journal.pone.0260033

72. Boscarino JA, Adams RE, Wingate TJ, et al. Impact and risk of moral injury among deployed veterans: implications for veterans and mental health. Frontiers in psychiatry. 2022;13. https://doi.org/10.3389/fpsyt.2022.899084

73. Brady C, Fenton C, Loughran O, et al. Dublin hospital workers' mental health during the peak of Ireland's COVID-19 pandemic. Irish Journal of Medical Science (1971-). 2022 Jun 22:1-0. https://doi.org/10.1007/s11845-022-03056-0

74. Brady C, Fenton C, Loughran O, et al. Nursing home staff mental health during the Covid-19 pandemic in the Republic of Ireland. International journal of geriatric psychiatry. 2021; 37(1), 1-10. https://doi.org/10.1002/gps.5648

75. Chandrabhatla T, Asgedom H, Gaudiano ZP, de Avila L, Roach KL, Venkatesan C, Weinstein AA, Younossi ZM. Second victim experiences and moral injury as predictors of hospitalist burnout before and during the COVID-19 pandemic. Plos one. 2022 Oct 4;17(10):e0275494. https://doi.org/10.1371/journal.pone.0275494

76. Dale LP, Cuffe SP, Sambuco N, et al. Morally distressing experiences, moral injury, and burnout in Florida healthcare providers during the COVID-19 pandemic. International Journal of Environmental Research and Public Health. 2021 Nov 24;18(23):12319. https://doi.org/10.3390/ijerph182312319

For peer review only - http://bmjopen.bmj.com/site/about/guidelines.xhtml

77. Plouffe RA, Easterbrook B, Liu A, et al. Psychometric evaluation of the moral injury events scale in two Canadian armed forces samples. Assessment. 2023 Jan;30(1):111-23. https://doi.org/10.1177/10731911211044198

78. Nichter B, Norman SB, Maguen S, et al. Moral injury and suicidal behavior among US combat veterans: Results from the 2019–2020 National Health and Resilience in Veterans Study. Depression and anxiety. 2021 Jun;38(6):606-14. https://doi.org/10.1002/da.23145

79. Fitzpatrick JJ, Pignatiello G, Kim M, et al. Moral Injury, Nurse Well-being, and Resilience Among Nurses Practicing During the COVID-19 Pandemic. JONA: The Journal of Nursing Administration. 2022 Jul 1;52(7/8):392-8. DOI: 10.1097/NNA.0000000000001171

80. Hagerty SL, Williams LM. Moral Injury, Traumatic Stress, and Threats to Core Human Needs in Health-Care Workers: The COVID-19 Pandemic as a Dehumanizing Experience. Clinical Psychological Science. 2022 Jan 10:21677026211057554. https://doi.org/10.1177/21677026211057554

81. Hamrick HC, Ehlke SJ, Davies RL, et al. Moral injury as a mediator of the associations between sexual harassment and mental health symptoms and substance use among women veterans. Journal of interpersonal violence. 2022 Jun;37(11-12):NP10007-35. https://doi.org/10.1177/0886260520985485

82. Hines SE, Chin KH, Levine AR, et al. Initiation of a survey of healthcare worker distress and moral injury at the onset of the COVID-19 surge. American journal of industrial medicine. 2020 Sep 1. DOI: 10.1002/ajim.23157

83. Hinkel HM, Currier JM, Isaak SL, et al. Moral injury and chronic pain among military veterans in an integrated behavioral health clinic. Psychological trauma:

For peer review only

theory, research, practice, and policy. 2022 Mar 24. https://doi.org/10.1037/tra0001230

84. Kelley ML, Chae JW, Bravo AJ, et al. Own soul's warning: Moral injury, suicidal ideation, and meaning in life. Psychological trauma: theory, research, practice, and policy. 2021 Aug 5. https://doi.org/10.1037/tra0001047

85. La Fleur R, McDonald M, Senecal G, et al. The Impact of Moral Injury and Disclosure of Military Experiences on Veterans. Journal of Humanistic Psychology. 2020 Nov 20:0022167820972214. https://doi.org/10.1177/0022167820972214

86. Lamb D, Gnanapragasam S, Greenberg N, Bhundia R, et al. Psychosocial impact of the COVID-19 pandemic on 4378 UK healthcare workers and ancillary staff: initial baseline data from a cohort study collected during the first wave of the pandemic. Occupational and environmental medicine. 2021 Nov 1;78(11):801-8. http://dx.doi.org/10.1136/oemed-2020-107276

87. Litam SD, Balkin RS. Moral injury in health-care workers during COVID-19 pandemic. Traumatology. 2021 Mar;27(1):14. https://doi.org/10.1037/trm0000290

88. Litz BT, Plouffe RA, Nazarov A, et al. Defining and assessing the syndrome of moral injury: initial findings of the Moral Injury Outcome Scale Consortium. Frontiers in psychiatry. 2022;13. https://doi.org/10.3389/fpsyt.2022.923928

89. Maftei A, Holman AC. The prevalence of exposure to potentially morally injurious events among physicians during the COVID-19 pandemic. European Journal of Psychotraumatology. 2021 Jan 1;12(1):1898791. https://doi.org/10.1080/20008198.2021.1898791

For peer review only

For peer review only - http://bmjopen.bmj.com/site/about/guidelines.xhtml

90. Maguen S, Griffin BJ, Vogt D, et al. Moral injury and peri-and post-military suicide attempts among post-9/11 veterans. Psychological medicine. 2022 Jan 17:1-0. https://doi.org/10.1017/S0033291721005274

91. Malakoutikhah A, Zakeri MA, Koenig HG, et al. The psychometric properties of the Persian version of the moral injury symptoms scale-health care professionals version. Frontiers in psychology. 2022:4803. https://doi.org/10.3389/fpsyg.2022.978572

92. Mantri S, Lawson JM, Wang Z, et al. Identifying moral injury in healthcare professionals: The moral injury symptom scale-HP. Journal of religion and health. 2020 Oct;59(5):2323-40. https://doi.org/10.1007/s10943-020-01065-w

93. Mantri S, Song YK, Lawson JM, et al. Moral injury and burnout in health care professionals during the COVID-19 pandemic. The Journal of Nervous and Mental Disease. 2021 Oct 1;209(10):720-6. DOI: 10.1097/NMD.0000000000001367

94. Morris DJ, Webb EL, Devlin P. Moral injury in secure mental healthcare part II: experiences of potentially morally injurious events and their relationship to wellbeing in health professionals in secure services. The Journal of Forensic Psychiatry & Psychology. 2022 Sep 3;33(5):726-44. https://doi.org/10.1080/14789949.2022.2111319

95. Rushton CH, Thomas TA, Antonsdottir IM, Net al. Moral injury and moral resilience in health care workers during COVID-19 pandemic. Journal of palliative medicine. 2022 May 1;25(5):712-9. https://doi.org/10.1089/jpm.2021.0076

96. Nieuwsma JA, Brancu M, Wortmann J, et al. Screening for moral injury and comparatively evaluating moral injury measures in relation to mental illness

For peer review only

symptomatology and diagnosis. Clinical Psychology & Psychotherapy. 2021 Jan;28(1):239-50. https://doi.org/10.1002/cpp.2503

97. Osmann J, Page-Gould E, Inbar Y, et al. Validation of the Toronto Moral Injury Scale for Journalists. Traumatology. 2022 Aug 25. https://doi.org/10.1037/trm0000409

98. Rodríguez EA, Agüero-Flores M, Landa-Blanco M, et al. Moral injury and light triad traits: anxiety and depression in health-care personnel during the coronavirus-2019 pandemic in Honduras. Hispanic health care international. 2021 Dec;19(4):230-8. https://doi.org/10.1177/1540415321104237

99. Roth SL, Andrews K, Protopopescu A, et al. Development and preliminary evaluation of the moral injury assessment for public safety personnel. Traumatology. 2022 Mar 3. https://doi.org/10.1037/trm0000367

100. Russell B, Mussap AJ. Rumination and threat-biased interpretation mediate posttraumatic stress and growth responses to military stressors. Military Psychology. 2022 Oct 2:1-6. https://doi.org/10.1080/08995605.2022.2127618

101. Saba SK, Davis JP, Lee DS, et al. Moral injury events and behavioral health outcomes among American veterans. Journal of anxiety disorders. 2022 Aug 1;90:102605. https://doi.org/10.1016/j.janxdis.2022.102605

102. Senger AR, Torres D, Ratcliff CG. Potentially morally injurious events as a mediator of the association of gratitude and mindfulness with distress. Psychological Trauma: Theory, Research, Practice, and Policy. 2022 Mar 31. https://doi.org/10.1037/tra0001233

103. Stanojević S, Čartolovni A. Moral distress and moral injury and their interplay as a challenge for leadership and management: The case of Croatia. Journal of Nursing Management. 2022 Oct 4. https://doi.org/10.1111/jonm.13835

For peer review only - http://bmjopen.bmj.com/site/about/guidelines.xhtml

104. Testoni I, Brondolo E, Ronconi L, et al. Burnout following moral injury and dehumanization: A study of distress among Italian medical staff during the first COVID-19 pandemic period. Psychological Trauma: Theory, Research, Practice, and Policy. 2022 Aug 4. https://doi.org/10.1037/tra0001346

105. Thomas ED, Weiss NH, Forkus SR, et al. Examining the interaction between potentially morally injurious events and religiosity in relation to alcohol misuse among military veterans. Journal of Traumatic Stress. 2022 Feb;35(1):314-20. https://doi.org/10.1002/jts.22682

106. Trifunovic-Koenig M, Strametz R, Gerber B, Mantri S, Bushuven S. Validation of the German Version of the Moral Injury Symptom and Support Scale for Health Professionals (G-MISS-HP) and Its Correlation to the Second Victim Phenomenon. International Journal of Environmental Research and Public Health. 2022 Jan;19(8):4857. https://doi.org/10.3390/ijerph19084857

107. Ulusoy S, Çelik Z. The Silent Cry of Healthcare Workers: A Cross-Sectional Study on Levels and Determinants of Burnout among Healthcare Workers after First Year of the Pandemic in Turkey. Psychiatry and Clinical Psychopharmacology. 2022 Mar 1;32(1):63-71. DOI:10.5152/pcp.2022.21248

108. Williamson V, Murphy D, Greenberg N. Experiences and impact of moral injury in UK veterinary professional wellbeing. European journal of psychotraumatology. 2022 Dec 31;13(1):2051351. https://doi.org/10.1080/20008198.2022.2051351

109. Zahiriharsini A, Gilbert-Ouimet M, Langlois L, Biron C, Pelletier J, Beaulieu M, Truchon M. Associations between psychosocial stressors at work and moral injury in frontline healthcare workers and leaders facing the COVID-19 pandemic in

For peer review only - http://bmjopen.bmj.com/site/about/guidelines.xhtml

Quebec, Canada: A cross-sectional study. Journal of Psychiatric Research. 2022 Nov 1;155:269-78. https://doi.org/10.1016/j.jpsychires.2022.09.006

110. Levi-Belz Y, Shemesh S, Zerach G. Moral injury and suicide ideation among combat veterans: The moderating role of self-disclosure. Crisis: The Journal of Crisis Intervention and Suicide Prevention. 2022 Feb 9. https://doi.org/10.1027/0227-5910/a000849

111. Schwartz G, Halperin E, Levi-Belz Y. Moral injury and suicide ideation among combat veterans: The role of trauma-related shame and collective hatred. Journal of interpersonal violence. 2022 Aug;37(15-16):NP13952-77. DOI: 10.1177/08862605211007932

112. Currier JM, Isaak SL, McDermott RC. Validation of the Expressions of Moral Injury Scale-Military version-short form. Clinical psychology & psychotherapy. 2020 Jan;27(1):61-8. https://doi.org/10.1002/cpp.2407

113. Nash WP, Marino Carper TL, Mills MA, Au T, Goldsmith A, Litz BT. Psychometric evaluation of the moral injury events scale. Military medicine. 2013 Jun 1;178(6):646-52. https://doi.org/10.7205/MILMED-D-13-00017

114. Hall NA, Everson AT, Billingsley MR, Miller MB. Moral injury, mental health and behavioural health outcomes: A systematic review of the literature. Clinical psychology & psychotherapy. 2022 Jan;29(1):92-110. https://doi.org/10.1002/cpp.2607

115. McEwen C, Alisic E, Jobson L. Moral injury and mental health: A systematic review and meta-analysis. Traumatology. 2021 Sep;27(3):303. https://doi.org/10.1037/trm0000287

116. Cumpston M, Li T, Page MJ, Chandler J, Welch VA, Higgins JP, Thomas J. Updated guidance for trusted systematic reviews: a new edition of the Cochrane

For peer review only - http://bmjopen.bmj.com/site/about/guidelines.xhtml

Handbook for Systematic Reviews of Interventions. The Cochrane database of systematic reviews. 2019(10).

117. Migliavaca CB, Stein C, Colpani V, Barker TH, Ziegelmann PK, Munn Z, Falavigna M, Prevalence Estimates Reviews—Systematic Review Methodology Group (PERSyst). Meta-analysis of prevalence: I 2 statistic and how to deal with heterogeneity. Research synthesis methods. 2022 May;13(3):363-7. https://doi.org/10.1002/jrsm.1547

118. French L, Hanna P, Huckle C. “If I die, they do not care”: UK National Health Service staff experiences of betrayal-based moral injury during COVID-19. Psychological Trauma: Theory, Research, Practice, and Policy. 2022 Mar;14(3):516. https://doi.org/10.1037/tra0001134

119. Hegarty S, Lamb D, Stevelink SA, Bhundia R, Raine R, Doherty MJ, Scott HR, Marie Rafferty A, Williamson V, Dorrington S, Hotopf M. ‘It hurts your heart’: frontline healthcare worker experiences of moral injury during the COVID-19 pandemic. European journal of psychotraumatology. 2022 Dec 19;13(2):2128028. https://doi.org/10.1080/20008066.2022.2128028

For peer review only

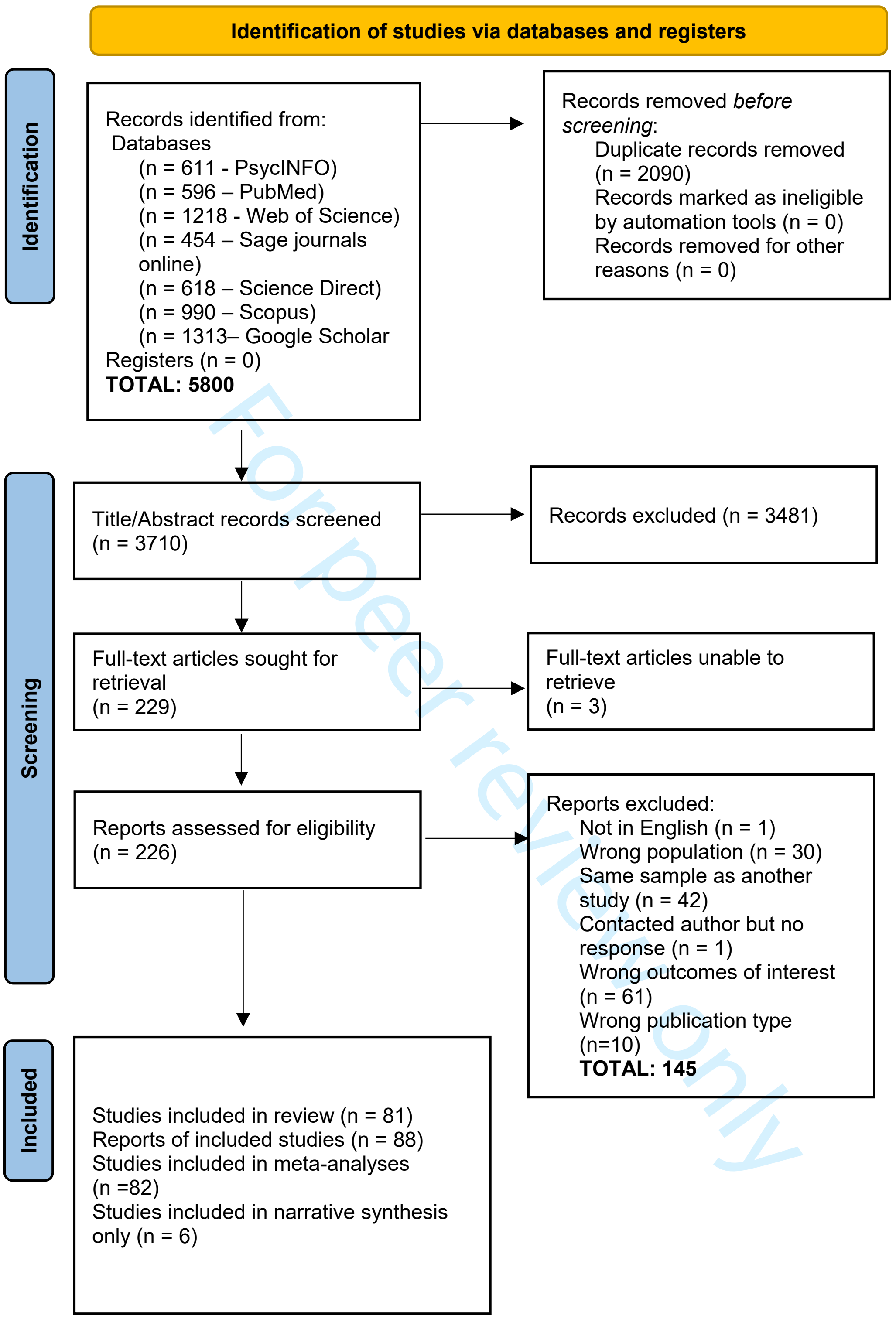

For peer review only

Supplementary materials

Supplementary Table 1.
Search strategy

Search date: 22 June 2020 and updated 2 November 2022

Moral injury search strategy

APA PsycINFO (EBSCOhost interface)

((“moral injur*” OR “transgressive act*” OR “moral transgression”)) AND ((Veteran* OR military OR army OR marine* OR "national service" OR "territorial army" OR "armed forces" OR "special forces" OR RAF OR "air force" OR navy OR "service personnel" OR "ex-service personnel" OR submariner* OR sailor* OR seamen OR seaman OR airmen OR airman OR soldier* OR troop* OR "enlisted personnel" OR "armed forces" OR "armed services" OR police* OR fireman OR firemen OR firefighter* OR teacher* OR "humanitarian aid worker" OR "relief aid worker" OR "social worker" OR "disaster responder" OR policeman OR policemen OR policewoman OR "law enforcement" OR healthcare OR nurse OR doctor OR frontline OR "health professional" OR "hospital staff" OR "hospital worker" OR work OR occupation OR job OR "emergency service"))). Filters Published: 2009-2022; Expanders - Apply equivalent subjects; Narrow by Language: - English, Search modes - Find all my search terms.

PubMed

("moral injur*" OR "morally injurious" OR PMIE* OR "potentially morally injurious" OR "moral transgression" OR "transgressive act*") AND (Veteran* OR military OR army OR marine* OR "national service" OR "territorial army" OR "armed forces" OR "special forces" OR RAF OR "air force" OR navy OR "service personnel" OR "ex-service personnel" OR submariner* OR sailor* OR seamen OR seaman OR airmen OR airman OR soldier* OR troop* OR "enlisted personnel" OR "armed forces" OR "armed services" OR police* OR fireman OR firemen OR firefighter* OR teacher* OR "humanitarian aid worker" OR "relief aid worker" OR "social worker" OR "disaster responder" OR policeman OR policemen OR policewoman OR "law enforcement" OR healthcare OR nurse OR doctor OR frontline OR "health professional" OR "hospital staff" OR "hospital worker" OR work OR job OR occupation OR "emergency service*") Filters: English; Published 2009-2022; all fields.

Web of Science Core Collection

For peer review only - http://bmjopen.bmj.com/site/about/guidelines.xhtml

(TS=((mora* injur* OR moral transgression*) AND (Veteran* OR military OR army OR marine* OR "national service" OR "territorial army" OR "armed forces" OR "special forces" OR RAF OR "air force" OR navy OR "service personnel" OR "ex-service personnel" OR submariner* OR sailor* OR seamen OR seaman OR airmen OR airman OR soldier* OR troop* OR "enlisted personnel" OR "armed forces" OR "armed services" OR police* OR fireman OR firemen OR firefighter* OR teacher* OR "humanitarian aid worker" OR "relief aid worker" OR "social worker" OR "disaster responder" OR policeman OR policemen OR policewoman OR "law enforcement" OR healthcare OR nurse OR doctor OR frontline OR "health professional" OR "hospital staff" OR "hospital worker" OR work OR job OR "emergency service" OR occupation)) and Article (Document Types) and English (Languages) and 2009-2022 (Publication Years)

Sage Journals Online

[[All "moral injur*"] OR [All "transgressive act*"] OR [All "moral transgression*"]] AND [[All veteran*] OR [All military] OR [All army] OR [All marine*] OR [All "national service"] OR [All "territorial army"] OR [All "armed forces"] OR [All "special forces"] OR [All raf] OR [All "air force"] OR [All navy] OR [All "service personnel"] OR [All "ex-service personnel"] OR [All submariner*] OR [All sailor*] OR [All seamen] OR [All seaman] OR [All airmen] OR [All airman] OR [All soldier*] OR [All troop*] OR [All "enlisted personnel"] OR [All "armed forces"] OR [All "armed services"] OR [All police*] OR [All fireman] OR [All firemen] OR [All firefighter*] OR [All teacher*] OR [All "humanitarian aid worker"] OR [All "relief aid worker"] OR [All "social worker"] OR [All "disaster responder"] OR [All policeman] OR [All policemen] OR [All policewoman] OR [All "law enforcement"] OR [All healthcare] OR [All nurse] OR [All doctor] OR [All frontline] OR [All "health professional"] OR [All "hospital staff"] OR [All "hospital worker"] OR [All work] OR [All occupation] OR [All "emergency service*"] OR [All job]]
Filters: Research articles, Published 2009-2022.

ScienceDirect

all = ("moral injury" OR "morally injurious" OR "moral transgression" OR "transgressive act") AND (veteran OR police OR job OR healthcare OR occupation). Filters: Research articles; Published 2009-2022.

Scopus

TITLE-ABS-KEY (("mora*injur*" OR "transgression") AND (veteran* OR police OR military OR "law enforcement" OR healthcare OR occupation)). Filters: DOCTYPE “ar”; Published 2009-2022; Language "English".

For peer review only - http://bmjopen.bmj.com/site/about/guidelines.xhtml

Google Scholar

All in the title: "moral injury" . Filters: = Published 2009-2022; Language "English".

PTED search strategy

APA PsycINFO (EBSCOhost interface)

((PTED or "post traumatic embitterment disorder" OR embittered OR embitterment)) AND ((Veteran* OR military OR army OR marine* OR "national service" OR "territorial army" OR "armed forces" OR "special forces" OR RAF OR "air force" OR navy OR "service personnel" OR "ex-service personnel" OR submariner* OR sailor* OR seamen OR seaman OR airmen OR airman OR soldier* OR troop* OR "enlisted personnel" OR "armed forces" OR "armed services" OR police* OR fireman OR firemen OR firefighter* OR teacher* OR "humanitarian aid worker" OR "relief aid worker" OR "social worker" OR "disaster responder" OR policeman OR policemen OR policewoman OR "law enforcement" OR healthcare OR nurse OR doctor OR frontline OR "health professional" OR "hospital staff" OR "hospital worker" OR work OR occupation OR job OR emergency service*)).
Filters - Published: 2003-2022; Expanders - Apply equivalent subjects; Narrow by Language: - English'; Exclude Dissertations; Search modes - Find all my search terms.

PubMed

("post traumatic embitterment disorder" OR PTED OR embittered OR embitterment) AND (Veteran* OR military OR army OR marine* OR "national service" OR "territorial army" OR "armed forces" OR "special forces" OR RAF OR "air force" OR navy OR "service personnel" OR "ex-service personnel" OR submariner* OR sailor* OR seamen OR seaman OR airmen OR airman OR soldier* OR troop* OR "enlisted personnel" OR "armed forces" OR "armed services" OR police* OR fireman OR firemen OR firefighter* OR teacher* OR "humanitarian aid worker" OR "relief aid worker" OR "social worker" OR "disaster responder" OR policeman OR policemen OR policewoman OR "law enforcement" OR healthcare OR nurse OR doctor OR frontline OR "health professional" OR "hospital staff" OR "hospital worker" OR work OR job OR occupation OR "emergency service"). Filters: English; Published 2009-2022; all fields.

Web of Science Core Collection

For peer review only - http://bmjopen.bmj.com/site/about/guidelines.xhtml

(TS=("post traumatic embitterment disorder" OR PTED OR embittered OR embitterment) AND TS=(Veteran* OR military OR army OR marine* OR "national service" OR "territorial army" OR "armed forces" OR "special forces" OR RAF OR "air force" OR navy OR "service personnel" OR "ex-service personnel" OR submariner* OR sailor* OR seamen OR seaman OR airmen OR airman OR soldier* OR troop* OR "enlisted personnel" OR "armed forces" OR "armed services" OR police* OR fireman OR firemen OR firefighter* OR teacher* OR "humanitarian aid worker" OR "relief aid worker" OR "social worker" OR "disaster responder" OR policeman OR policemen OR policewomen OR "law enforcement" OR healthcare OR nurse OR doctor OR frontline OR "health professional" OR "hospital staff" OR "hospital worker" OR work OR occupation OR job OR emergency service*)) and Article (Document Types) and English (Languages) and 2003-2022 (Publication Years)

Sage Journals Online

[[All "post traumatic embitterment disorder"] OR [All pted]] OR [All "chronic embitterment"]] AND [[All veteran*] OR [All military] OR [All army] OR [All marine*] OR [All "national service"] OR [All "territorial army"] OR [All "armed forces"] OR [All "special forces"] OR [All raf] OR [All "air force"] OR [All navy] OR [All "service personnel"] OR [All "ex-service personnel"] OR [All submariner*] OR [All sailor*] OR [All seamen] OR [All seaman] OR [All airmen] OR [All airman] OR [All soldier*] OR [All troop*] OR [All "enlisted personnel"] OR [All "armed forces"] OR [All "armed services"] OR [All police*] OR [All fireman] OR [All firemen] OR [All firefighter*] OR [All teacher*] OR [All "humanitarian aid worker"] OR [All "relief aid worker"] OR [All "social worker"] OR [All "disaster responder"] OR [All policeman] OR [All policemen] OR [All policewoman] OR [All "law enforcement"] OR [All healthcare] OR [All nurse] OR [All doctor] OR [All frontline] OR [All "health professional"] OR [All "hospital staff"] OR [All "hospital worker"] OR [All work] OR [All occupation] OR [All job] OR [All "emergency service*"]]. Filters: Research articles, Published 2003-2022.

ScienceDirect

("post traumatic embitterment disorder" OR pted OR embitterment OR embittered) AND (veteran OR police OR "law enforcement" OR healthcare OR occupation). Filters: Research articles; Published 2003-2022.

Scopus

ALL ((pted OR "post traumatic embitterment disorder" OR embittered OR embitterment) AND (veteran* OR police OR military OR job OR healthcare OR work OR occupation)). Filters: DOCTYPE “ar”; Published 2003-2022; Language "English".

For peer review only - http://bmjopen.bmj.com/site/about/guidelines.xhtml

**Google Scholar**
All: pted OR embitterment OR "post traumatic embitterment disorder". Filters: = Published 2009-2022; Language "English".

For peer review only

For peer review only - http://bmjopen.bmj.com/site/about/guidelines.xhtml

Supplementary Table 2.

Data extraction table for studies included in the systematic review

Author (Year)	Study Design	N_a	Occupation	Sampling method	Mean age	% Male	% White	Location	RR	Measurement tool	Associations (+) significant positive correlation (-) significant negative correlation (0) not significant	Prevalence N (%)	Total M(SD)	Hoy	Conflict of Interest
PTED only studies															
Dunn & Sensky. 2018	Cross-sectional	79	Healthcare NHS staff.	Convenience Occupational health department October 2015-March 2016.	44 (12)	33%	NR	UK	NR	PTED scale 1.6+ and 2+	(Mental) health<br>Depression (HADS) (+)<br>Individual and/or work characteristics<br>Affective rumination (WRRQ) (+)<br>Detachment (WRRQ) (-)<br>Problem solving pondering (WRRQ) (0)<br>Positive beliefs about rumination (PBRS) (0)<br>Sickness absence (0)<br>Demographics<br>Gender (0)	1.6 cut off: 37 (47%)<br>2 cut off: 28(35%)	1.5 (1.1)	5	NR
Linden & Rotter 2019	Cross-sectional	102	Unemployed Duration > 1 year (32.4%)	Convenience	36.6 (12.1)	59%	NR	Germany	NR	PTED scale 2+	(Mental) health<br>Psychological distress (GHQ-28) (+)<br><br>Individual and/or work characteristics<br>Feelings of injustice (+)<br>Worse appraisal of unemployment (+)<br>Duration of unemployment (+)<br>Demographics<br>Gender (0)<br>Age (+)	Cut off 2: 26 (25.5%)	1.24 (0.92)	5	None

For peer review only

Michailids & Cropley. 2018	Longitudinal (T1)	352	Multiple.	Convenience	NR	NR	NR	Cyprus	NR	PTED scale 1.6+	(Mental) health Negative affect (+) Individual and/or work characteristics Organisational procedural justice (OJP) (-) Organisational distributive justice (OJP) (-) Organisational interpersonal justice (OJP) (-) Organisational informational justice (OJP) (-) Supervisory control (+) Job demands (0)	Cut off 1.6: 55 (35%)	1.28 (0.92)	5	None
Sensky, Salimu, Ballard & Pereira. 2015	Cross sectional	236	NHS staff, primarily nurses (N = 67, 28%) and healthcare assistants (n = 49, 21%). 45% worked for the trust for <5 years.	Convenience September-November 2009 Represents sample frame.	NR	45%	NR	UK.	NR	PTED scale 1.6+	Individual and/or work characteristics Work attitudes - felt obligation (0) Work attitudes - organisational support (-) Work attitudes - employer-employee reciprocity (0) Work attitudes - work effort (0) Work attitudes - procedural justice (-) Management referral to occupational health (+) Certified sickness absence (+)	Cut off 1.6: 68 (28.81%) Cut off 2: 43 (18.2%) Cut off 2.5: 21 (8.9%)	1.11(0.97)	4	None

For peer review only - http://bmjopen.bmj.com/site/about/guidelines.xhtml

											Uncertified sickness absence (+)<br>Work stress - demands (HSE) (0)<br>Work stress - control (HSE) (0)<br>Work stress - management support (HSE) (-)<br>Work stress - peer support (HSE) (-)<br>Work stress – relationships (HSE) (0)<br>Work stress - role (HSE) (-)<br>Work stress – change (HSE) (-)<br>Demographics:<br>Gender (0)<br>Age (0)<br>Years since qualifying (0)<br>More years in present job (+)				
Sabic, Sabic, & Baltic-Mujanovic, 2018	Cross sectional	87	War veterans of the war in Bosnia and Herzegovnia (1992-1995). Average time since they were a soldier M = 23.86 years.	Convenience	51.42 (5.98)	100%	NR	Bosnia and Herzegovina	NR	PTED scale 1.6+, 2+ and 2.5+	NR	Cut off 1.6: 15 (17.24%)<br>Cut off 2: 9 (10.34%)<br>Cut off 2.5: 1 (1.15%)	0.75(0.73)	5	None

For peer review only

Michailidis & Cropley. 2017	Cross sectional	337	Multiple	Convenience	36.87 (12.4)	26.1 %	NR	NR	NR	PTED scale 1.6+, 2+ and 2.5+	(Mental) health<br>Negative affect (PANAS) (+)<br>Positive affect (PANAS) (-)<br><br>Individual and/or work characteristics<br>Affective rumination (WRRQ) (+)<br>Problem solving pondering (WRRQ) (+)<br>Detachment (WRRQ) (-)<br>Job demands (JCQ) (0)<br>Workplace social support (-)<br>Organisational procedural justice (OJP) (-)<br>Organisational distributive justice (OJP) (-)<br>Organisational interpersonal justice (OJP) (-)<br>Organisational informational justice (OJP) (-)<br>Supervisory control (WCOWP) (+)<br><br>Demographics<br>Gender (+)<br>Age (0)<br>Dependent children (0)	Cut off 1.6: 253 (75%)<br>Cut off 2: 209 (62%)<br>Cut off 2.5: 152 (45%)	2.44(.96)	5	None

For peer review only - http://bmjopen.bmj.com/site/about/guidelines.xhtml

											Hours worked per week (+)<br>Years in current job (+)				
Karatuna & Gok 2014	Cross-sectional	397	Public sector, social security institutions Non-managerial position = 83.1%	Convenience	NR	57.2 %		Turkey	39.1 1%	PTED scale 1.6+, 2+, 2.5+	Individual and/or work characteristics<br>Workplace bulling (NAQ-R) (+)<br>Victimisation from bullying (+)<br>Victims of bulling (+)	Cut off 1.6: 106 (26.7%)<br>Cut off 2: 74 (18.6%)<br>Cut off 2.5: 41 (10.3%)	1.00(1.04)	5	NR
Rubab & Tariq 2022	Cross-sectional	300	Private school teachers.	Purposive.	31 (7.9)	50%	NR	NR	NR	PTED scale 1.6+, 2+, 2.5+	(Mental) health<br>Depression (DASS-21) (+)<br>Stress (DASS-21) (+)<br>Anxiety (DASS-21) (+)<br>Depression, stress, and anxiety total (DASS-21) (+)<br><br>Individual and/or work characteristics<br>Generalised self-efficacy (GSES) (-)	Cut off 1.6: 121 (40.3%)<br>Cut off 2: 77 (25.7%)<br>Cut off 2.5: 26 (8.7%)	1.44 (0.70)	5	NR
Saleem, Bashir & Abrar, 2022	Longitudinal (T2)	398	Public sector university academics.	Simple random sampling.	44.5	64%	NR	Pakistan	73 %	PTED scale (mean only)	(Mental) health<br>Employee well-being (-)<br>Individual and/or work characteristics	NR	3.16 (1.12)	5	None

For peer review only - http://bmjopen.bmj.com/site/about/guidelines.xhtml

											Leaders' ethical behaviours (-)<br>Employee core self-evaluation (-)				
Shin & You 2022	Cross-sectional	1074	Unemployed.	Convenience	47.1 (13.6)	47.6 %	NR	South Korea	NR	PTED scale 1.6+, >1.6 ≥ 2, 2.5+	Individual and/or work characteristics<br>Work types (0)<br>Unemployment duration (0)<br>Unemployment route (0)<br>Negative life events (+)<br>General belief in a just world (BJW) (-)<br>Personal BJW (-)<br>Demographics<br>Gender (0)<br>Age (+)<br>Marital status (divorced/separated) (+)<br>Educational level (-)<br>Income (-)	Cut off 1.6: 581 (54.1%)<br>Cut off 1.6 - 2.5: 479 (44.6%)<br>Cut off 2.5: 102 (9.5%)	1.64 (0.70)	5	None
PTED and moral injury study															
Brennan, McKay & Cole, 2022	Longitudinal (T1)	400	Health and social care<br>Allied health (14.5%)<br>Primary care practitioner (15.8%)<br>Managerial (10.3%)<br>Health info/science (9.3%)	Convenience September-October 2020.	36.84 (10.7)	24.5 %	63%	UK	89.5 %	MIES 4+ (slightly agree)<br>3 subscales (transgressions-others, transgressions-self, betrayal)<br><br>PTED 1.6+ and 2+ and 2.5	Transgressions-self (Mental) health:<br>Current mental health diagnoses (0)<br>Individual and/or work characteristics<br>Exposure to occupational stressors (+)<br>Pessimism (LOTR) (+)<br>Consideration of immediate	MIES:<br>Overall %<br>289 (72.3%)<br>Per subscale %<br>Transgressions-others: 213 (53.3%)<br><br>Transgressions-self: 131 (32.8%)<br>Betrayal: 227 (56.8%)	MIES:<br>Overall M/SD<br>2.55(1.13)<br>Subscale M/SD<br>Transgressions-others: 2.98(1.48)<br><br>Transgressions-self: 2.17(1.22)<br>Betrayal: 2.77(1.38)	2	ESRC and University funding

For peer review only - http://bmjopen.bmj.com/site/about/guidelines.xhtml

Psychological (6.8%)<br>Corporate (16.3%)<br>Clinical support (26.3%)	consequences (CFC-14) (+)<br>Consideration of future consequences (CFC-14) (0)<br>Resilient coping style (BRCS) (0)<br>Self-esteem (RSES) (-)<br>Procedural justice (BJW) (-)<br>Distributive justice (BJW) (0)<br>Social desirability (-)<br>Demographics *<br>Age (0)<br>Gender (0)<br>Professional role (0)<br>Ethnicity (0)<br>Years in current role (0)<br>Transgressions-others (Mental) health:<br>Current mental health diagnoses (0)<br>Individual and/or work characteristics<br>Exposure to occupational stressors (+)<br>Pessimism (LOTR) (+)<br>Consideration of immediate consequences (CFC-14) (0)<br>Consideration of future consequences (CFC-14) (0)	MIES per item %:<br>Item 1: 179 (44.8%)<br>Item 2: 167 (41.8%)<br>Item 3: 76 (19%)<br>Item 4: 94 (23.5%)<br>Item 5: 83 (20.8%)<br>Item 6: 78 (19.5%)<br>Item 7: 161 (40.3%)<br>Item 8: 124 (31%)<br>Item 9: 158 (39.5%)<br>PTED overall %:<br>1.6 cut off: 121 (30.3%)<br>2 cut off: 77 (19.3%)<br>2.5 cut off: 30 (7.5%)	PTED:<br>Overall M/SD<br>1.12(0.90)

For peer review only

For peer review only - http://bmjopen.bmj.com/site/about/guidelines.xhtml

Resilient coping style (BRCS) (+)
Self-esteem (RSES) (-)
Procedural justice (BJW) (-)
Distributive justice (BJW) (0)
Social desirability (-)
Betrayal:
Mental health:
Current mental health diagnoses (+)
Individual and/or work characteristics
Exposure to occupational stressors (+)
Pessimism (LOTR) (+)
Consideration of immediate consequences (CFC-14) (+)
Consideration of future consequences (CFC-14) (0)
Resilient coping style (BRCS) (0)
Self-esteem (RSES) (-)
Procedural justice (BJW) (-)
Distributive justice (BJW) (-)
Optimism (LOTR) (-)
Social desirability (-)
PTED:
Mental health:

For peer review only

	Current mental health diagnoses (+)<br>Individual and/or work characteristics<br>Exposure to occupational stressors (+)<br>Pessimism (LOTR) (+)<br>Consideration of immediate consequences (CFC-14) (+)<br>Consideration of future consequences (CFC-14) (0)<br>Resilient coping style (BRCS) (0)<br>Self-esteem (R-SES) (-)<br>Procedural justice (BJW) (-)<br>Distributive justice (BJW) (-)<br>Optimism (LOTR) (-)<br>Social desirability (-)<br>Demographics<br>Age (+)<br>*PTED and all MIES subscales
Moral injury only studies	

For peer review only

For peer review only - http://bmjopen.bmj.com/site/about/guidelines.xhtml

Bryan et al., 2016<br><br>Sample 2	Cross-sectional	935	Serving military personnel. Army national guard (84%).	Convenience	27.05 (8.11)	82.3 %	57.4%	USA	NR	MIES 6+ (strongly agree)<br>3 subscales (transgressions-others, transgressions-self, betrayal)	Individual and/or work characteristics<br>Transgressions-others subscale:<br>Pre-deployment stressors (DDRI) (+)<br>Combat experiences (DDRI) (+)<br>Aftermath of battle (DDRI) (+)<br>Transgressions-self subscale:<br>Pre-deployment stressors (DDRI) (0)<br>Combat experiences (DDRI) (0)<br>Aftermath of battle (DDRI) (0)<br>Betrayal subscale:<br>Pre-deployment stressors (DDRI) (+)<br>Combat experiences (DDRI) (0)<br>Aftermath of battle (DDRI) (0)<br>Demographics<br>Gender (0)	Overall %<br>NR<br>Per subscale %<br>Cut off 6<br>Transgressions-others: 363 (38.8%)<br>Transgressions-self: 508 (54.3%)<br>Betrayal: 540 (47.8%)	Overall M/SD<br>NR<br>Subscale M/SD<br>Transgressions-others: 4.32(1.67)<br>Transgressions-self: 4.92(1.51)<br>Betrayal: 4.63(1.63)	5	None
Currier, Holland, Drescher et al., 2015<br><br>Sample 1	Cross-sectional	131	Veterans. Deployed to Iraq and/or Afghanistan<br>Marine Corps (38.32%), Active-Duty Army (35.1%)	Convenience	28.47 (5.87)	88%<br><br>Consistent with military demographic trends	26%<br><br>Consistent with ethnic diversity of the region of study	USA	NR	MIQ-M<br>20 items<br>Unidimensional.<br>Average 3.02 years since event (returning from war zone).	(Mental) health<br>Suicide risk (SQB-R) (0)<br>PTSD (PCL-C) (+)<br>Depression (PHQ-9) (+)<br>Individual and/or work characteristics:<br>Combat exposure (CES) (+)	Overall<br>NR<br>Per subscale<br>NR	Overall M/SD<br>32.4(12.8)<br>Subscale M/SD<br>NR	3	NR

For peer review only

			and Navy (22.9%).								Work and social adjustment (WSAS) (+)<br>Active duty and marine's vs other service branches (+)<br>Deployments (+)<br>Demographics<br>Older age (+)<br>Gender (0)<br>Ethnicity (0)				
Currier, Holland, Rojas-Flores et al., 2015	Cross sectional.	257	Teachers. Classroom teachers (83.7%). 13.7% administrators e.g., principal (13.7%), and both classroom teacher and school administrator (2.7%) Average time in role 15.44 (10.29).	Convenience March 2012	42.02 (13.12)	31.4 %	NR	El Salvador	95 %	MIQ-T 12 items Unidimensional.	(Mental) health<br>PTSD Reexperiencing (LASC) (+)<br>PTSD Avoidance (LASC) (+)<br>PTSD Hyperarousal (LASC) (+)<br>Physical burnout (SMBM) (+)<br>Cognitive burnout (+)<br>Emotional burnout (+)<br>Individual and/or characteristics<br>Violence exposure (SECV) (+)<br>Meaning made of stress comprehensibility (ISLES) (-)<br>Meaning made of stress footing in the world (ISLES) (-)	Overall NR<br>Per subscale NR	Overall M/SD 13.92(6.71)<br>No subscales	4	NR

For peer review only

Currier et al., 2017<br><br>Sample 2	Cross-sectional	624	Veterans. 1+ deployments to a war zone during their service.<br><br>49.2% (Army), 10.6% (Marine Corps), 22.1% (Navy), 21.8% (Air Force), 0.8% (Coast Guard).<br><br>97.4% (Active Duty), 11.9% (Military Reserves), 6.3% (National Guard	Convenience	NR	81.4 %	10.9%	USA	7.3 %	EMIS<br>2 subscales (self-directed MI and other-directed MI).<br>MIQ-M<br>7 items<br>1 subscale (causes)<br>MIES<br>2 subscales (perpetration and betrayal)	(Mental) health<br>PTSD (PCL 5) (+)<br>Depression (PHQ-8) (+)<br>Alcohol misuse (AUDIT-C) (+)<br>Anger (DAR-5) (-)<br>Guilt (PFQ-2) (+)<br>Shame (PFQ-2) (+)<br>Individual and/or work characteristics<br>Combat exposure – Survival threat (DDRI) (+)<br>Combat exposure – Aftermath of violence (DDRI) (+)	Overall<br>NR<br>Per subscale<br>NR	EMIS:<br>Overall M/SD<br>32.14(14.84)<br>Subscale M/SD<br>Self-MI<br>14.92(7.49)<br>Other-MI<br>17.22(8.23)<br>MIQ-M<br>Overall M/SD<br>NR<br>Subscale causes M/SD<br>9.29(3.41)<br>MIES<br>Overall M/SD<br>NR<br>Subscales<br>Perpetration<br>2.68(1.50)<br>Betrayal<br>2.80(1.66)	5	NR

For peer review only

Forkus, Juliana & Weiss. 2019	Cross sectional	203	Veterans. Deployed to Iraq or Afghanistan.<br>Army (52.20%), Air Force (19.20%), Navy (15.80%), and Marine Corps (12.80%).	Convenience	35.08	77.30 %	70.40%	NR	29.17%	MIES Unidimensional	(Mental) health<br>PTSD (PCL-5) (+)<br>Depression (PHQ) (+)<br>Alcohol misuse (+)<br>Drug misuse (+)<br>Deliberate self-harm history (+)<br>Deliberate self-harm versatility (+)<br>Individual and/or work characteristics<br>Self-compassion (-)	NR	Overall M/SD 2.95(1.41)	5	NR
Frankfurt et al., 2018.	Longitudinal secondary data analysis. MIES reported at T2	310	Veterans<br><br>Post 9/11.<br><br>Army (90.3%). Active duty (96.8%).<br><br>Average years in role 13.50 (7.61)	Convenience	40.67 (8.55) .	76%	57%<br><br>Reflected geographic area	USA	NR	MIES<br>2 subscales (perpetration and betrayal)	(Mental) health<br>Military sexual trauma (DDRI) (+) for perpetration and betrayal subscales<br>Depression (+) for perpetration and betrayal subscales<br>PTSD (+) for perpetration and betrayal subscales<br>Shame (+) for perpetration and betrayal subscales<br>Guilt (+) for perpetration and betrayal subscales<br>Individual and/or work characteristics<br>Combat exposure (0)*<br>Combat exposure (+)**<br>*betrayal<br>**perpetration	NR	Overall M/SD<br>NR<br>Subscales<br>Perpetration 3.00(1.40)<br>Betrayal 3.41(1.64)	4	None

For peer review only

Griffin et al., 2020<br><br>Narrative synthesis only	Cross sectional.	498	Military service members and veterans<br><br>Army (22.49%), Navy (17.47%), Air Force (45.78%), Marines (11.24%), Coastguard (2.61%). Active Duty (29.52%).	Convenience	NR	73.90 %	NR	USA	NR	MIES<br>5 of 9 items to create 5 profiles	NR due to using 'profiles'	Overall %<br>NR<br>Subscales<br>No moral distress (42% of sample)<br>Moral Distress-Other (19%)<br>Witnessing-Only (16%)<br>Moral distress-Self (8%)<br>Moral distress-Self and Other (15%)	NR	5	NR
Jinkerson Battles. 2019.	Cross sectional	72	Veterans Vietnam and post-Vietnam eras.<br><br>Army (59.7%), Navy (13.9%), Air Force (12.5%), Marine Corps (12.5%)	Convenience	NR	87.5 %	45.8%	USA	76 %	MIQ-M<br>20 items<br>Unidimensional	(Mental) health<br>Meaning in life (-)<br>Depression (PHQ-2) (+)<br>Anxiety (GAD-2) (+)<br>PTSD (PCL-5) (+)<br>PTSD Reexperiencing (PCL-5) (+)<br>PTSD avoidance (PCL-5) (+)<br>PTSD Negative emotions (PCL-5) (+)<br>PTSD hyperarousal/hypervigilance (PCL-5) (+)<br>Global guilt (TRGI) (+)<br>Distress (TRGI) (+)	Per item %<br>(top 5 items)<br>Betrayal by military/political leaders N = 32 (44.5%)<br>experiencing events that were chaotic and beyond control N = 32 (44.4%)<br>failing to save the life of someone in war N = 24 (33.3%)<br>surviving when others	Overall M/SD<br>3.48(0.35)<br>Subscales<br>NR	4	NR

For peer review only

											Guilty cognitions (TRGI) (+)<br>Wrongdoing (TRGI) (+)<br>Lack of justification (TRGI) (0)	didn't N = 21 (29.2%)<br>feeling betrayal by trusted civilians N = 32 (29.2%)			
Jordan et al., 2017.	Longitudinal. Data from 1 month post deployment reported here and from cohort 4 from the parent study.	867	Currently serving active-duty marines from a single infantry battalion engaged in heavy ground combat while deployed to Afghanistan.	Convenience	NR	NR	NR	USA	NR	MIES 4+ (slightly agree)<br>Subscales: 2 subscales (transgressions-self and betrayal)	(Mental) health<br>PTSD (PLC-S) (+)<br>Guilt/shame (PANAS) (+) *<br>Anger (PANAS) (+) *<br>Individual and/or characteristics<br>Dissociation (PDEQ) (+) *<br>Combat exposure (CES) (+) *<br>* transgressions-self and betrayal subscales	Overall % NR<br>Per subscale<br>Transgressions-self 214 (24.1%)<br>Betrayal 252 (28.4%)<br>Transgressions-self and betrayal: 333 (37.5%)<br>Per item %<br>Item 1: 165 (18.6%)<br>Item 2: 89 (10%)<br>Item 3: 128 (14.4%)<br>Item 4: 103 (11.6%)<br>Item 7: 179 (20.2%)<br>Item 8: 133 (15%)<br>Item 9: 132 (14.9%)	Overall M/SD NR, only used below subscales<br>Subscales<br><br>Transgressions-self = 1.87(1.17)<br><br>Betrayal = 2(1.23)	5	NR

For peer review only

For peer review only - http://bmjopen.bmj.com/site/about/guidelines.xhtml

Lee Aldwin, Kang. 2022	Cross sectional.	367<br><br>354 responded to MIES	Veterans<br><br>Deployed to the Vietnam war in 1664-1973.<br><br>Army (88%).	Convenience	72 (2.66)	100%	NR	Korea	37.4 %	MIES 3+ (sometimes) and 4+ (often)<br>5-point scale not 6 (1=never, 2=seldom, 3=sometimes, 4=often, 5=always).<br><br>3 subscales (transgressions-others, transgressions-self, betrayal)<br><br>40-50 years since event	(Mental) health<br>PTSD (Checklist for DSM-5) (+)<br>Depression (BSI) (+)<br>Anxiety (BSI) (+)<br><br>Individual and/or work characteristics<br>Optimism (LOT) (-)<br>Unit cohesion (-)<br>Homecoming experience (DDRI-2) (0)<br>Combat exposure (CES) (+)<br>Malevolent environments (CES) (+)<br>Perceived threat (CES) (+)<br>Demographics<br>Age (0)<br>Education (-)<br>Marital status (0)<br>Income (0)	Overall %<br>Cut off 4+: 104(29.4%)<br>Cut off 3+: 176(49.7%)<br>Subscales %<br>Transgressions-others cut off 4+: 57(16.1%)<br>Transgressions-self cut off 4+: 53(14.9%)<br>Betrayal cut off 4+: 59(16.7%)<br>Transgressions-others cut off 3+: 130(36.7%)<br>Transgressions-self cut off 3+: 115(32.5%)<br>Betrayal cut off 3+: 121(34.2%)	Overall M/SD<br>1.80(0.80)<br>Subscales<br>Transgressions-others: 2.04(1.04)<br>Transgressions-self: 1.69(0.86)<br>Betrayal: 1.80(0.91)	5	None
Ogle, Reichwald, Rutland 2018<br><br>Narrative synthesis only	Cross sectional	356	Serving military analysts<br><br>Geospatial intelligence analysts (29%), cryptologic language analysts (54.8%) who	Convenience	NR	70.3 %	NR	USA	NR	MIES<br>10 items instead of 9<br>3 subscales (transgressions-others, transgressions-self, betrayal)	(Mental) health<br>PTSD (PCL-5) (+) *<br>PTSD Avoidance (PCL-5) (+) *<br>PTSD Mood/cognition (PCL-5) (+) *<br>PTSD Hypervigilance (PCL-5) (+) *<br>Reliving (PCL-5) (+) *	Overall %<br>NR<br>Subscales %<br>NR<br>Per item % split by combat exposure level<br>Item 1: None = 9.1%, Low = 21%, Middle =	Overall M/SD<br>NR<br>Subscales M/SD<br>NR	5	NR

For peer review only

For peer review only

provide support to remote combat and global vigilance operations and who have routine exposure to related video, audio, still images, and other media.	Social adjustment (-) * Affect work/home (+) * Individual and/or work characteristics Combat exposure (CES) (+) * Work satisfaction (-) *transgressions-self and betrayal Work satisfaction (0) transgressions-others Combat ambiguity (+) * * Transgressions-others, transgressions-self and betrayal subscales	26%, High = 47.3% Item 2: None = 11.6%, %, Low = 11.6%, Middle = 6.2%, High = 15.4% Item 3: None = 4.7%, %, Low = 29%, Middle = 46.4%, High = 60.5% Item 4: None = 2.3%, %, Low =8.2%, Middle = 5%, High = 11%, Item 5: None = 2.3%, Low = 9.4%, Middle = 4.1%, High = 9.9% Item 6: None = 4.6%, Low =7.1%, Middle = 2%, High = 8.8% Item 7: None = 2.3%, Low =6.9%, Middle = 2%, High = 7.7%, r_s = .109 Item 8: None = 11.3%, Low =17.5%,

For peer review only

												Middle = 10.1%, High = 20.9%<br>Item 9: None = 7%, Low =11.7%, Middle = 3%, High = 13.2%<br>Item 10: None = 7%, Low =10.5%, Middle = 5%, High = 7.7%			
Wisco et al., 2017.	Cross sectional.	564	Veterans. Experienced combat. Army 41%.	Epidemiological sampling, recruited from the National Health and Resilience in Veterans Study (NHRVS).	NR	93%	76.2%	USA	25 %	MIES: 5+ (moderate) 3 subscales (transgressions-others, transgressions-self, betrayal) 33.7 years (19.1) since event (last combat deployment)	Individual and/or work characteristics<br>Vietnam war era (0)<br>Military branch (army) (+)<br>Multiple deployments (+)<br>Moderate/heavy combat (+)<br>Demographics<br>Age (0)<br>Gender (0)<br>White race (-)<br>College education (-)<br>Annual income >60k (-)<br>Currently employed (+)	*Reports weighted % and SE<br><br>Overall %<br>223, (41.8%, 2.1%))<br><br>Subscale %<br>Transgressions -Others 126, (25.5%, 1.8%))<br><br>Transgressions -Self 48 (10.8%, 1.3%)<br><br>Betrayal 142 (25.1%, 1.8%)<br><br>Per item<br>Item 1: 115 (23.8%, 1.8%)<br>Item 2: 65, (14.1%, 1.5%)	Overall M/SD 2.11(1.08)<br>Subscales M/SD<br><br>Transgressions -others 2.71(1.66)<br><br>Transgressions -self 1.75(1.16)<br><br>Betrayal 2.19(1.29)	1	None

For peer review only - http://bmjopen.bmj.com/site/about/guidelines.xhtml

												Item 3: 37<br>(8.9%, 1.2%)<br>Item 4: 30<br>(5.9%, 1%)<br>Item 5: 21<br>(3.9%, 0.8%)<br>Item 6: 19<br>(3.6%, 0.8%)<br>Item 7: 107<br>(18.6%, 1.7%)<br>Item 8: 81<br>(15%, 1.5%)<br>Item 9: 31<br>(5%, 0.9%)			
Zerach, Levi-Belz. 2017	Cross sectional	191	Veterans. 100% army.	Convenience<br><br>March-July 2017.	25.39 (2.37)	85.4 %	NR	Israel	86.8 %	MIES 4+ (slightly agree)<br>3 subscales (transgressions-others, transgressions-self, betrayal)<br>Time since deployment 4.36(2.27) years.<br><br>MIQ-M 2+ (seldom)<br>14 causal items<br>1 subscale (causes)	(Mental) health<br>Depression (DAQ) and MIQ-M (0)<br>Depression (DAQ) and MIES (+) *<br>PTSD (PCL-5) and betrayal (+)<br>PTSD (PCL-5) and transgression-others, transgressions-self and MIQ-M (0)<br>Guilt-global (TRGI) and MIQ-M and MIES (+) *<br>Guilt-distress (TRGI) and MIQ-M and MIES (+) *<br>Guilt-cognitions (TRGI) and MIQ-M and MIES (+) *<br>Shame-intrinsic (TSRI) and MIQ-M and MIES (+) *	Overall %<br>92(48.4%)<br>Subscales<br>Transgressions-others 64 (33.7%)<br>Transgressions-self 42 (21.9%)<br><br>Betrayal 59 (31%)<br><br>Per item MIES %<br><br>Item 1: 55 (29%) Item 2: 42 (22%)<br>Item 3: 23 (12%) Item 4: 25 (13%)<br>Item 5: 19 (10%)	Overall M/SD 2.00(0.97)<br>Subscales MIES<br>Transgressions-others 2.42(1.39)<br>Transgressions-self 1.68(1.05)<br>Betrayal 2.14(1.22)<br>Subscales MIQ-M<br>MIQ causes 1.34(0.34)	4	NR

For peer review only

For peer review only - http://bmjopen.bmj.com/site/about/guidelines.xhtml

											Shame-extrinsic (TSRI) and MIQ-M and MIES (+) * Self-disgust-self (TRSI) and MIQ-M and MIES (0) * Self-disgust -ways (TSRI) and betrayal (+), Self-disgust ways (TSRI) and transgressions-self, transgressions-others, and MIQ-M (0) Individual and/or work characteristics Combat exposure (CES) (+) MIQ-M Combat exposure (0) MIES * *transgressions-self, transgressions-other and betrayal subscales	Item 6: 23 (12%) Item 7: 48 (25%) Item 8: 32 (17%) Item 9: 29 (15%) Per item MIQ-M %<br><br>Item 4: 86 (45.2%) Item 8: 60 (31.4%)			
Papazoglou et al., 2019.	Cross sectional.	390	Service police officers. National Police of Finland. Average years of police experience 16.87(9.11)	Convenience	41.21 (8.42)	73.5 %	100%	Finland	86 %	MIES 5+ (moderately agree) and 6+ (strongly agree) Item 6 not included 2 subscales (perpetration and betrayal)	(Mental) health PTSD (PCL-C) (+) * Compassion fatigue (CSF) (+) * Compassion satisfaction (+) *<br><br>Individual and/or work characteristics Dark triad (SD3) (+) * *Perpetration and betrayal subscales	Overall % Overall, MI (moderate): 289 (74%) Subscale % Perpetration 152 (39%) (moderate) Betrayal 250 (64%) (moderate) Perpetration 39 (10%) (high)	Overall M/SD NR Subscale M/SD Perpetration 2.61 (1.19)<br><br>Betrayal 3.77 (.98)	4	NR

For peer review only - http://bmjopen.bmj.com/site/about/guidelines.xhtml

												Betrayal 94 (24%) (high)			
Richardson, Chesnut et al., 2020	Longitudinal (T2)	5,227	Veterans. Recently separated from service<br><br>Army (36.8%), Navy (22.4%), Air Force (20.5%), marines (20.2%).	Epidemiological, Recruited from The Veteran Metrics Initiative (TVMI) study.	NR	82.1 %	66.9%	USA	10.67%	MIES 4+ (slightly agree)<br>2 subscales, (MI-self and MI-other)<br>Within 90 days since event (separation from service)	NR	Overall % NR<br>Subscale %<br>MI-Self: 516 (9.87%)<br>MI-Other: 1033 (19.76%)<br>Per item prevalence<br>Item 1: 2432 (46.53%)<br>Item 2: 1496 (28.62%)<br>Item 3: 833(15.94%)<br>Item 4: 750(14.35%)<br>Item 5: 813(15.55%)<br>Item 6: 741(14.18%)<br>Item 7: 1955(37.4%)<br>Item 8: 1472 (28.16%)<br>Item 9: 1039 (19.88%)	Overall M/SD 2.24(1.07)<br>Subscale M/SD<br>MI-Self: 1.79(1.25)<br>MI-Other: 2.51(1.40)	2	None

For peer review only

For peer review only - http://bmjopen.bmj.com/site/about/guidelines.xhtml

Feinstein, Pavisian, Storm. 2018	Cross sectional	80	Journalists. Years in role 18.28 (8.09)<br><br>months covering the refugee crisis 19.65 (19.41)	Convenience	42.95 (8.44)	58.8 %	70.40%	Nine European and American news organisations	70.2 %	MIES<br>6 items<br>1 subscale (perpetration)	(Mental) health<br>PTSD intrusion (IES-R) (+)<br>PTSD avoidance (IES-R) (+)<br>PTSD arousal (IES-R) (+)<br>Depression (BDI-II) (0)<br>Alcohol consumption (0)<br>Individual and/or work characteristics<br>Higher workload in past year (+)<br>Control over resources (-)<br>Guilt (+)<br>Demographics<br>Having children (+) t-test<br>Gender (0)<br>Age (0)<br>Education (0)<br>Marriage (0)<br>Marital status (0)	Overall % NR<br>Subscales % NR	Overall M/SD NR (only one subscale)<br>Subscales<br>Perpetration 2.83(0.93)	5	HR is director of INSI
Sugrue, 2020	Cross sectional	218	K-12 education<br><br>Classroom teacher (50.5%), special education staff (19.3%), Non-classroom instruction	Convenience	42.6 (11.9)	22.9 %	77.1%	USA	<7 %	MIES 4+ (slightly agree)<br>3 subscales (transgressions-others, transgressions-self, betrayal)	(Mental) health<br>Transgressions-others subscale:<br>Personal burnout (CBI) (+)<br>Work burnout (CBI) (+) *<br>Client burnout (CBI) (+)<br>Global guilt (TRGI) (+)<br>Distress Scale (TRGI) (+)	Overall % 191 (85.3%).<br>Subscale %<br><br>Transgressions-others, 175 (80.2%)<br><br>Transgressions-self, 98 (45.4%)	Overall M/SD 3.49(1.24)<br>Subscale M/SD<br>Transgressions-others 4.4(1.5)<br>Transgressions-self, 2.9(1.5)<br>Betrayal 3.8(1.5)	5	NR

For peer review only - http://bmjopen.bmj.com/site/about/guidelines.xhtml

For peer review only

al support (10.1%), School social workers (5%), English language learner (ELL) teachers (5%), Specialist teachers (4.6%), School psychologists (3.7%), School counsellors (0.9%), school nurses (0.5%)<br><br>Average years working in education 15.0(10.1)	Hindsight bias (TRGI)<br>Wrongdoing (TRGI)<br>Lack of justification (TRGI) (0)<br>Stress of conscience (internal and external subscales and total (SCQ)) (+)<br>Transgressions-self subscale:<br>Personal burnout (CBI) (+)<br>Work burnout (CBI) (+) *<br>Client burnout (CBI) (0)<br>Global guilt (TRGI) (+)<br>Distress Scale (TRGI) (+)<br>Hindsight bias (TRGI)<br>Wrongdoing (TRGI)<br>Lack of justification (TRGI) (+)<br>Stress of conscience (internal and external subscales and total (SCQ)) (+)<br>Betrayal subscale:<br>Personal burnout (CBI) (+)<br>Work burnout (CBI) (+) *<br>Client burnout (CBI) (+)<br>Global guilt (TRGI) (+)	Betrayal, 148 (68.4%)<br><br>Transgressions -others, 115(52.7%) – moderate (5) cut off

For peer review only

											Distress Scale (TRGI) (+)<br>Hindsight bias (TRGI)<br>Wrongdoing (TRGI)<br>Lack of justification (TRGI) (0)<br>Stress of conscience (internal and external subscales and total (SCQ)) (+)<br>Individual and/or work characteristics<br>Transgressions-others subscale<br>Intention to leave (ILS) (+)<br>Transgressions-self subscale<br>Intention to leave (ILS) (0)<br>Betrayal subscale<br>Intention to leave (ILS) (+)				
Aldridge, Scott, Paskell. 2019.	Cross sectional.	104	Veterans.<br><br>British Army, Royal Navy or Royal Air Force and had experienced active deployment.	Convenience<br><br>Feb 2017-Nov 2017.	47 (10.8)	95.2 %	NR	UK	NR	MIQ-M 19 items Unidimensional.	(Mental) health<br>PTSD (PCL-M) (+)<br>Individual characteristics<br>Guilt proneness (TOSCA) (-)<br>Shame proneness (TOSCA) (0)<br>Demographics<br>Age (0)<br>Gender (0)<br>Length of service (0)<br>Previous therapy (0)	Overall % NR<br>Subscale % NR	Overall M/SD 36.75(10.12)<br>Subscale M/SD NR	5	NR

For peer review only - http://bmjopen.bmj.com/site/about/guidelines.xhtml

Andrukonis, Potopopova. 2020.	Cross sectional.	153	Animal shelter.<br><br>Shelter director (n=16), assistant director (4), animal shelter supervisor/manager (21), animal control officer (18), kennel attendant/animal care tech (29), customer service representative (13), vet/vet tech/medical (22), behaviour/training (9), volunteer/rescue/community (3), other (22)	Convenience<br><br>Dec 2017-April 2018.	NR	NR	NR	USA	NR	MIES Higher score represents lower moral injury Unidimensional.	Individual and/or work characteristics<br>Shelter type (0)<br>Admission type (0)<br>Euthanizes in job (-)<br>Demographics<br>Months on the job (0)	Overall % NR<br><br>Subscale % NR	Overall M/SD 0.96(4.22)<br>Subscale M/SD NR	5	None

For peer review only - http://bmjopen.bmj.com/site/about/guidelines.xhtml

			Years in job M=5.9												
Haight., Sugrue, Calhoun. 2017.	Cross sectional	38	CPS Social worker (n = 13) County attorney (n = 5) Guardians ad litem (n = 5) Student attorneys (n = 10) Law school clinic and parent support staff (n = 5) Years in CPS 8.5(8.6)	Convenience	NR	18%	66%	USA	56.3 3%	MIES 4+ (slightly agree)<br><br>3 subscales (transgressions-others, transgressions-self, betrayal)	Demographics Age (0) Gender (0) Race (0) Years of experience in CPS (0) Years of education and transgressions-self and betrayal (-)	Overall % NR Subscale %<br><br>Transgressions-others 35(92%)<br><br>Transgressions-self 9(24%)<br><br>Betrayal 12(32%)	Overall M/SD NR Subscale M/SD Transgression-others 5.1(1) Transgression-self 2.7(1.4) Betrayal 3.1(1.2)	5	NR

For peer review only - http://bmjopen.bmj.com/site/about/guidelines.xhtml

Battles et al. 2019.	Cross sectional	380	Currently serving military and veterans that had deployed to 1+ war zones for 3+ months. Navy (43.5% of men, 53.3% of women). No longer serving (67.1% of men, 66.7%) of women. Length of time serving (men 12.88, 10, women, 9.84, 8)	Convenience	35.29 (9.58)	68.42 %	71.9% of men and 61.7% of women	USA	NR	MIQ-M 12 causal items 1 subscale (causes)	(Mental) health Spiritual injury (+) Total alcohol use (AUDIT) in men (+) Total alcohol use in women (0) Alcohol consumption (AUDIT) in men (+) Alcohol consumption (AUDIT) in women (0) Alcohol dependence (AUDIT) in men (+) Alcohol dependence (AUDIT) in women (0)<br><br>Individual and/or work characteristics Combat exposure (CES) (+) Military status (+) (0) Military branch (+)<br><br>Demographics Years in service (0)	Overall % NR Subscale % NR	Overall M/SD NR Subscale M/SD (causes 12 items) 1.26(0.42)	5	NR
Braitman et al., 2018.<br><br>Narrative synthesis only	Cross sectional	328	Current military or veterans with deployed experience Veterans (69.5%) National Guard/Reservists	Convenience	32.42 (7.71)	56.1 %	68.0%	USA	NR	MIQ-M modified 2+ (seldom) 27 PMIE items plus 135 follow up questions (5 per PMIE item)	(Mental) health Depression (CES-D-10) (+) Anxiety (KAS) (+) PTSD (PCL-5) (+) Hazardous alcohol use (AUDIT) (+) Drug abuse (DAST) (+) Drug abuse (DAST) (0) *	Overall % NR<br><br>Subscale % NR<br><br>Per item % Item 7: 216 (65.9%) Item 8: 174 (53%)	Overall M/SD NR Subscale M/SD<br><br>AoW PMIEs 1.77(0.63) PCoW PMIEs 1.99(0.66) LFB PMIEs 2.12(0.78)	5	None

For peer review only - http://bmjopen.bmj.com/site/about/guidelines.xhtml

(19.5%)<br>Active-duty members (9.5%)<br>Army (46.3%)<br>Navy (29.9%)<br>Air force (7.9%)<br>Marines (5.5%)<br>National guard (4%)<br>Reserves (4.6%).	3 subscales. (atrocities at war (AoW); psychological consequences of war (PCoW), leadership failure or betrayal (LFB).	All subscales<br>*LFT and AoW	Item 10: 182(55.5%)<br>Item 11: 155(47.3%)<br>Item 15: 181(55.2%)<br>Item 16: 150(45.7%)<br>Item 17: 178(54.3%)<br>Item 19. 191(58.2%)<br>Item 22: 179(54.6%)<br>Item 23: 170(51.8%)<br>Item 24: 198(60.4%)<br>Item 26: 192(58.5%)<br>Item 27: 183(55.8%)<br>Item 6: 208 (63.4%)<br>Item 9: 231 (70.4%)<br>Item 13: 233 (71%)<br>Item 18: 227(69.2%)<br>Item 21: 220(67.1%)<br>Item 25: 199(60.7%)<br>Item 1: 218(66.5%)<br>Item 2: 225(68.6%)<br>Item 4.	AoW defining characteristics 2.21(0.67)<br>PCoW defining characteristics 2.14(0.66)<br>LFB defining characteristics 2.12(0.67)<br>All defining characteristics 2.11(0.66)

For peer review only

												239(72.9%)<br>Item 3:<br>200(61%)<br>Item 5:<br>168(51.2%)<br>Item 12:<br>208(63.4%)<br>Item 14:<br>174(53%)<br>Item 20.<br>214(65.2%)			
Lancaster Harris, 2018.	Cross sectional	182	Veterans Deployed to Iraq or Afghanistan<br>Army (61.0%)<br>Air Force (17.0%),<br>Marine Corps (14.8%),<br>Navy (7.1%).	Convenience	33.66 (7.23)	80.2 %	78.0%	NR	32.3 3%	MIES<br>3 subscales (transgressions-others, transgressions-self, betrayal)<br><br>MIQ-M<br>19 items<br>2 subscales (causes and effect)	(Mental) health<br>Anger (DAR-5) (+) *<br>Depression (PHQ) (+) *<br>Alcohol (AUDIT) (+) *<br>PTSD (PCL-5) (+) *<br>Guilt (+) *<br>Shame (+) *<br><br>Individual and/or work characteristics<br>Combat (CES) (+)*<br>CES (0)**<br><br>All subscales<br>*transgressions-others and transgressions-self subscales<br>**betrayal subscale	Overall %<br>NR<br><br>Subscale %<br>NR	Overall M/SD MIES<br>2.75(1.32)<br><br>Subscale M/SD MIES<br>Transgressions-others<br>3.49(1.50)<br><br>Transgressions-self<br>2.45(1.53)<br><br>Betrayal<br>2.67(1.57)<br><br>Overall M/SD MIQ-M<br>33.70(11.90)<br><br>Subscale M/SD MIQ-M<br>Effect<br>11.54(4.21)<br><br>Causes<br>22.19(8.32)	5	NR

For peer review only - http://bmjopen.bmj.com/site/about/guidelines.xhtml

Lancaster, 2018	Cross sectional	161	Veterans. Post 9/11<br><br>Army (43.5%), Navy (19.9%), Air Force (16.1%), Marine Corps (13.7%), Coast Guard (6.8%).	Convenience	35.08 (9.62)	71.4 %	73.9%	NR	5.18 %	MIES<br>7 of 9 items<br>2 subscales (transgressions-self and betrayal)	(Mental) health<br>PTSD (PTSD checklist 5) (+) *<br>Depression (PHQ) (+) *<br>Anger (+) *<br>Guilt/shame (SSGS) (+) *<br><br>Individual and/or work characteristics<br>Transgressive acts (TAS) (+) *<br>*transgressions-self and betrayal subscales	Overall %<br>NR<br>Subscales %<br>NR<br>*Range 12.6% to 23.3% on items	Overall M/SD<br>NR (only 2 subscales used)<br><br>Subscales M/SD<br>Transgressions-self, 2.56(1.43)<br>Betrayal 2.72(1.46)	5	NR
Akhtar, Faize, Malik & Tabusam, 2022	Cross-sectional	108	Healthcare Doctors (51.9%)<br>Nurse (26.9%)<br>Paramedics (21.3%)	Purposive<br><br>August 2020-January 2021.	27.67 (6.17)	41.7 %	NR	Pakistan.	NR	MISS-HP 36+	Individual and/or work characteristics<br>Psychological resilience (CD-RISC-10): (-)<br>Demographics<br>Age (0)<br>Experience (-)<br>Work hours (+)<br>Female gender (+)<br>Marital status (0)<br>Hospital type (0)<br>Profession (0)<br>Speciality/department psychiatry (+)<br><br>Work area/duty station (0)	Overall %<br>75 (69.44%)	Overall M/SD<br><br>43.36 (9.64)	6	NR

For peer review only - http://bmjopen.bmj.com/site/about/guidelines.xhtml

Qi et al., 2022<br><br>Two studies.	Study 1: Cross-sectional<br>Study 2: Cross-sectional	Study 1: 3006<br>Study 2:<br><br>3465	Study 1: Healthcare Nurse (18%)<br>Internal medicine (19.4%)<br>Obstetrics/ gynaecology/paediatrics (34.7%)<br>Surgery (9.6%)<br>Psychiatry (11.4%)<br>Other<br>Study 2: Healthcare Nurse (14.7%)<br>Internal medicine (44.7%)<br>Obstetrics/ gynaecology/paediatrics (21.1%)<br>Surgery (4.9%)<br>Psychiatry (8.5%)<br>Other (6.1)	Study 1: Snowball. March-April 2020.<br>Study 2: Snowball. March-April 2021	Study 1: NR<br>Study 2:<br><br>NR	Study 1: 34.9 %<br>Study 2: 24.6 %	Study 1: NR<br>Study 2: NR	Study 1: China<br>Study 2: China	Study 1: 75 %<br>Study 2:<br><br>86 %	Study 1: MISS-HP 36+<br>EMIS-SF<br>Study 2: MIISS-HP 36+	Study 1: (Mental) health<br>Depression (PHQ) (+)<br>Anxiety (GAD-7) (+)<br>Burnout emotional exhaustion (MBI-HSMP) (+)<br>Burnout reduced personal accomplishment (MBI-HSMP) (-)<br>Burnout depersonalisation (MBI-HSMP) (+)<br>Well-being (SFI) (-)<br>* EMIS and MISS-HP<br>Study 2: NR at univariate level.	Study 1: Overall, MISS-HP % 1695 (56.4%)<br>Study 2: Overall, MISS-HP % 1270 (36.7)	Study 1: MISS-HP overall: 46.9 (12.7)<br>EMIS-SF overall: 2.55 (0.80)<br>Study 2: MISS-HP: 45.9(11.3)	5	None

For peer review only

For peer review only - http://bmjopen.bmj.com/site/about/guidelines.xhtml

Amsalem et al., 2021	Longitudinal	350	Healthcare Nurses (68%) Physicians (15%) Emergency medical technicians (9%) Physical therapists (3%) Pharmacists (2%) Social workers and other therapists (1%) Other (2%)	Convenience September-December 2020	34.8 (11.5)	26%	73%	USA	NR	MIES: 3+ (agree) Likert scale 1 (disagree), 2 (strongly disagree) 3 (agree) 4 (strongly agree)<br><br>3 subscales (transgressions-others, transgressions-self, betrayal)	(Mental) health Depression (PHQ) (+) Anxiety (GAD-7) (+) Suicidal ideation (+) PTSD (PC-PTSD) (+) Demographics Age (0) Experience (-) Work hours (+) Female gender (+) Marital status (0) Hospital type (0) Profession (0) Speciality/department psychiatry (+) Work area/duty station (0)	Overall % 267 (76.29%) Per subscale % Transgressions -others: 215 (61.43%)<br><br>Transgressions -self: 101 (28.86) Betrayal: 205 (58.57%) Per item: Item1: 192 (55%) Item 2: 171 (49%) Item 3: 66 (19%) Item 4: 68 (19%) Item 5: 71 (20%) Item 6: 72 (21%) Item 7: 157 (45%) Item 8: 111 (32%) Item 9: 152 (43%)	Overall M/SD 1.98 (0.72) Subscale M/SD Transgressions -others: 2.41(1.04)<br><br>Transgressions -self: 1.63(0.82) Betrayal: 2.15(0.92)	5	None

For peer review only - http://bmjopen.bmj.com/site/about/guidelines.xhtml

Perez, Larson & Bair 2021	Cross-sectional	85	Veterans Served between 2001-2014. Army (63.5%) Marines (8.2%) Navy (10.6%) Air Force (15.3%) Coast Guard (2.4%)	Convenience	NR	68.2 %	78.8%	USA	NR	EMIS 2 subscales (self-directed MI and other-directed MI)	Individual and/or work characteristics Fairness (MFQ) (0) Loyalty (MFQ) (+) * Loyalty (MFQ) (0) ** Authority (MFQ) (0) Sanctity (MFQ) (0) *Self-MI **Other-MI	Overall % NR Per subscale NR	Overall M/SD 2.45(0.98) Subscale M/SD Self-MI 2.16(1.08) Other-MI 2.80(1.06)	5	NR
Benatov, Zerach & Levi-Belz 2022	Cross-sectional	296	Health and social care Doctor (34.1%) Nurse (including midwives) (44.4%) Social and psychological care (17.4%) Clinical support (5.9%)	Convenience	40.28 (10.8)	22.4 %	NR	Israel	NR	MIES 4+ (slightly agree) 3 subscales (transgressions-others, transgressions-self, betrayal)<br><br>MISS-HP 36+	(Mental) health Depression (PHQ) (+) Anxiety (GAD-7) (+) Individual and/or work characteristics Thwarted belonginess (INQ) (+)<br><br>*For MISS-HP and MIES scales	MIES: Overall %: NR Per subscale % Transgressions -others: 146 (49.3%)<br><br>Transgressions -self: 94 (31.8%) Betrayal: 184 (62.2%) Per item %: Item 1: 135 (45.5) Item 2: 108 (36.5%) Item 7: 135 (45.5%) Item 9: 94 (31.8%)	MIES: Overall M/SD (From supplement) 22.54(10.48) Subscale M/SD<br><br>Transgressions -others: 4.08(2.71) Transgressions -self: 1.49(0.81) Betrayal: 2.81(1.33) MISS-HP overall: 33.90(12.72)	5	NR

For peer review only

												MISS-HP overall % 120 (40.7%)			
Borges et al., 2021	Longitudinal	211	Healthcare Physician (12.32%) Nurse (10.43%) Mental health provider (61.14%) Occupational therapist (1.90%) Nurse practitioner (2.37%) Physician assistant (0.47%)	Convenience May 2020 – March 2021	NR	16.26 %	89.16%	USA	NR	MIES 2+ (moderately agree) Lower score indicates higher moral injury 2 subscales (perpetration and betrayal)	Demographics Sex (0) Race (0) Profession (0) No other variables reported at univariate level	Overall % 109 (51.66%) Per subscale % Perpetration: 77 (36.49%) Betrayal: 88 (41.71%)	Overall M/SD NR Subscale M/SD NR	5	None
Boscarino et al., 2022<br><br>Narrative synthesis only	Cross-sectional	1032	Military veterans.	Random sample of veterans on a registry care at a private hospital.<br><br>February 2016 – February 2017	NR	95.5 %	NR	USA	55 %	MIES 75th percentile Unidimensional	(Mental) health Cage positive alcohol score (+) Used alc/drugs to cope (+) Pain interferes (+) Sleep problems (+) Concussion history (+)	Overall % 764 (74%) Subscale % NR	Overall M/SD NR Subscale M/SD NR	3	None

For peer review only - http://bmjopen.bmj.com/site/about/guidelines.xhtml

											Mental health treatment in past year (+)<br>Opioid dependence past year (+)<br>Individual/work characteristics<br>Guard/reserve (-)<br>Combat high (+)<br>Drafted (+)<br>Vietnam war deployment (+)<br>Low unit support/morale (+)<br>High stress past year (+)<br>High lifetime trauma (+)<br>Low homecoming support (+)<br>High child abuse/neglect (+)<br>High fear of death (+)<br>High neuroticism (+)<br>Low self-esteem (+)<br>Repression to cope (+)<br>High anomie (+)<br>Anti-social personality (+)<br>Used marijuana 50+ times (+)<br>Demographics<br>Age (+)				
Brady et al., 2021	Cross-sectional<br>.	390	Nursing home staff. Nurses (30.8%)	Convenience November 2020 – January 2021.	NR	12.8 %	83.3%	Republic of Ireland.	10.2 %	MIES 4+ (slightly agree)	Demographics<br>HCA compared to non-clinical staff (+)	Overall %<br>237 (60.8%)<br>Per subscale %	Overall M/SD<br>2.35(1.09)<br>Subscale M/SD	4	Honoraria for board meeting.

For peer review only - http://bmjopen.bmj.com/site/about/guidelines.xhtml

			Healthcare assistants (44.1%)<br>Non-clinical (25.1%)							Cut off: 4+ (Slightly agree)<br>3 subscales (transgressions-others, transgressions-self, betrayal)		Transgressions -others: 189 (48.5%)<br>Transgressions -self: 92 (23.6%)<br><br>Betrayal: 166 (42.6%)	Transgressions -others: 2.93(1.51)<br>Transgressions -self: 1.97(1.19)<br>Betrayal: 2.46(1.34)		
Brady et al., 2022	Cross-sectional	377	Hospital Doctors (48%)<br>Nurses (44%)<br>Radiographers (8%)	Convenience January – March 2021	NR	22.3 %	90.5%	Republic of Ireland. Dublin.	6%	MIES 4+ (slightly agree)<br>Cut off: 4+ (Slightly agree)<br>3 subscales (transgressions-others, transgressions-self, betrayal)	Demographics<br>Professional role - doctors (-)	Overall %<br>268 (71.1%)<br>Per subscale %<br>Transgressions -others: 197 (52.3%)<br>Transgressions -self: 92 (24.4%)<br>Betrayal: 210 (55.7%)	Overall M/SD<br>2.48(1.10)<br>Subscale M/SD<br>Transgressions -others: 3.01(1.56)<br>Transgressions -self: 1.97(1.12)<br>Betrayal: 2.81(1.44)	6	
Chandrabhatla et al., 2022	Cross-sectional		Hospitalist	Convenience September 2019 – August 2020	NR	48%	NR	USA	52 %	MIES Unidimensional	(Mental health):<br>Burnout (0)<br>Burnout during COVID subsample (+)	Overall %<br>60 (71.4%)*<br>Subscale %<br>Transgressions -others: 49(58.3%)**<br>Transgressions -self: 23(27.4)*<br>Betrayal: 37(44%)*<br>*N=78<br>**N=77	Overall M/SD<br>2.44(1.07)*<br>Per subscale M/SD<br>Transgressions -others: 3.41(1.64)**<br>Transgressions -self: 1.88(1.15)***<br>Betrayal: 2.54(1.31)***<br>*N=73<br>**N=74<br>***N=76	5	None

For peer review only - http://bmjopen.bmj.com/site/about/guidelines.xhtml

Dale et al., 2021	Longitudinal	265	Healthcare	Convenience<br>October 2020 – March 2021	37.62 (11.1)	18.1 %	77.7%	USA	NR	MIES<br>3 subscales (transgressions-others, transgressions-self, betrayal)	NR at univariate level	Overall %<br>NR (7 items only)<br>Subscale %<br>Transgressions -others: 6 (2.3%)<br>Transgressions -self only: 61 (23%)<br>Perpetration: 21 (7.9%)<br>Perpetration (high): 88 (33.2%)	Overall M/SD<br>NR<br>Per subscale M/SD<br>Transgressions -others: 2.54(1.45)<br>Transgressions -self: 1.66(1.05)	5	None
Plouffe et al., 2021<br><br>Two samples	Longitudinal (T2)	1: 803<br><br>2: 1510	Military.<br>Sample 1: active duty<br>Sample 2 - released.	Nationally representative sample.<br>January – May 2018 (T2)	NR	Sample 1: 87.53 %<br>Sample 2: 88.02 %	NR	Canada	68 %	MIES<br>Unidimensional	NR	Overall %<br>NR for both samples<br>Subscale %<br>NR for both samples<br><br>Per item %<br>Sample 1:<br>Item 1: 64.6%<br>Item 2: 37.3%<br>Item 3: 16.9%<br>Item 4: 13.2%<br>Item 5: 19.9%<br>Item 6: 14.9%<br>Item 7: 48.5%<br>Item 8: 34.5%<br>Item 9: 27.5%<br>Sample 2:<br>Item 1: 66.1%<br>Item 2: 42.2%<br>Item 3: 20.4%<br>Item 4: 17.3%	Overall M/SD<br>Sample 1: 2.46(4.53)<br>Sample 2: 2.63(4.66)<br><br>Subscale M/SD<br>NR for both samples	2	None

For peer review only

												Item 5: 24.4%<br>Item 6: 21.3%<br>Item 7: 53%<br>Item 8: 37.8%<br>Item 9: 34.6%			
Nichter et al., 2021	Cross-sectional	1321	Veterans. All reported exposure to combat/war zone. Army (47.6%) Navy (14.6%) Air Force (6.3%) Marines (6.3%) National Guard/Reserves/Coast Guard (9.3%)	National random samples and apply post-stratification wrights to generalise to entire US veteran population. 2019-2020	59.1 (16.6)	93.7 %	75.1%	USA	NR	MIES 5+ (moderate) 3 subscales (transgressions-others, transgressions-self, betrayal)	NR at univariate level.	*Reports weighted % and SE<br><br>Overall % 535, (36.8%, 1.3%)<br><br>Subscale % Transgressions -Others 316, (23.1%, 1.2%)<br><br>Transgressions -Self 152 (11.2%, 0.9%)<br><br>Betrayal 189 (24.5%, 1.3%)<br><br>Per item<br>Item 1: 279 (21%, 1.1%)<br>Item 2: 189, (13.6%, 1.0%)<br>Item 3: 90 (6.5%, 0.7%)<br>Item 4: 88 (6.0%, 0.8%)	Overall M/SD 2.0(1.1)<br>Subscales M/SD<br><br>Transgressions -others 2.5(1.6)<br><br>Transgressions -self 1.7(1.1)<br><br>Betrayal 2.1(1.3)	1	NR

For peer review only - http://bmjopen.bmj.com/site/about/guidelines.xhtml

												Item 5: 77 (6.0%, 0.7%) Item 6: 71 (5.9%, 0.7%) Item 7: 289 (17.9%, 1.1%) Item 8: 214 (14.9%, 1.0%) Item 9: 79 (6.5%, 0.6%)			
Fitzpatric k et al., 2022	Cross-sectional.	676	Nurses. Staff nurses (65%) Nurse leaders (35%)	Convenience June - August 2021.	43.88 (1.99)	8.3%	89.6%	USA	NR	MISS-HP	(Mental) health Lower well-being (+) Individual and/or work characteristics Resilience (-) Demographics Staff nurses (+)	Overall % NR	Overall M/SD 42.21(15.73)	5	NR
Hagerty & Williams , 2022 Narrative synthesis only	Cross-sectional	1122	Healthcare Nurse (60.4%) Physician (5%) Physician assistant (1.7%) Respirator y therapist (3.6%) Other health-care roles (28%) Provided care in ICU during the	Convenience May 2020 – August 2020.	39.29	11.2 %	NR	USA	NR	MIES 4+ (slightly agree) 3 subscales (transgressi ons-others, transgressio ns-self, betrayal)	(Mental) health Loneliness (DJGLS) (+) Demographics Age (-) Access to PPE (-) Number of COVID-19 patient deaths that respondents personally treated (+)	Overall % NR Subscale % NR Per item % Item 2: 525 (47.2%) Item 7: 747 (67.2%) Item 4: 234 (21%)	Overall M/SD NR Subscale M/SD NR	5	None

For peer review only

			pandemic (47.6%)												
Hamrick et al., 2022	Cross-sectional	154	Military (serving and veterans). Former/retired (77.9%). Army (20.8%) Navy (52.6%) Airforce (16.2%) Marines (4.5%) Coast Guard (0.6%) National Guard (7.8%) (Above) reserves (20.8%) Other (0.6%)	Convenience February – April 2019	36.6 (11.8)	0%	72.1%	USA	NR	EMIS-M 1 subscale (other-directed)	(Mental) health Depression (+) Anxiety (+) Suicidal ideation (0) Substance use (0) Individual and/or work characteristics Sexual harassment (+)<br><br>*All correlations with the other-directed MI subscale.	Overall % NR<br><br>Subscale % NR	Overall M/SD NR Subscale M/SD Other-directed MI 2.66(1.17)	5	None

For peer review only

Hines et al., 2020	Cross-sectional	219	Health and social care Attending physician (47%) Fellow physician (18%) Resident physician (20%) Other (15%)	Convenience March – April 2020	39.10 (11.1)	43%	NR	USA	26 %	MIES 2 subscales (perpetration, and betrayal).	NR at univariate level.	Overall % NR<br><br>Subscale % NR	Overall M/SD 1.79 (0.87) Subscale M/SD Perpetration 1.65 (0.87) Betrayal 2.10 (1.28)	5	NR
Hinkel et al., 2022	Cross-sectional	59	Veterans.	Convenience November 2018 – November 2019	NR	86.4 %	59.3%	USA	NR	EMIS-M 2 subscales (self-directed MI and other-directed MI)	(Mental) health PTSD (PCL-5) (+) MDD (PHQ-8) (+) Chronic pain (BPI-SF) (+)	Overall % NR<br><br>Subscale % NR	Overall M/SD NR Subscale M/SD Self-directed MI 2.29(1.09) Other-directed MI 2.54(1.32)	5	NR
Kelley et al., 2021	Cross-sectional	269	Veterans. Navy (45.4%) Marine Corps (21.9%) Army (19.7%)	Convenience	36.5 (11)	49.8 %	74.3%	USA	NR	EMIS-M 2 subscales (self-directed MI and other-directed MI)	(Mental) health Suicidal ideation (+) PTSD diagnosis (+) Individual/work characteristics Presence of meaning in life (MLQ) (-) Searching for meaning in life (MLQ) (0) Demographics Veteran status (+) Age (0) *Both EMIS subscales	Overall % NR<br><br>Subscale % NR	Overall M/SD NR Subscale M/SD Self-directed MI 1.83(0.86) Other-directed MI 2.48(1.11)	5	None

For peer review only - http://bmjopen.bmj.com/site/about/guidelines.xhtml

La Fleur et al., 2020	Cross-sectional	212	Veterans. Marine Corps (13.7%) Army (26.4%) Navy (13.7%) Air Force (5.7%) Reserves (2.8%) National Guard (4.2%) Multiple (33.5%)	Convenience	NR	80.7 %	74.1%	USA	NR	MIQ-M 2.80+ 19 items Unidimensional.	(Mental) health Life satisfaction (0) Individual/work characteristics Disclosure support (0) Disclosure concerns (+)	Overall % 27% Overall % NR	Overall M/SD 2.04(0.79) Subscale M/SD NR	6	None
Lamb et al., 2021	Cross-sectional (cohort).	2166	Healthcare Doctor (11.7%) Nurse (29.9%) Other clinical (26.8%) Non-clinical (32.3%)	Convenience April-June 2020.	NR	59.4 %	24.6%	UK	12 %	MIES 5+ (moderately agree) Subscales: 3 subscales (commission, omission and betrayal)	Demographics Age (-) Sex other (+) Relationship status divorced/separated/widowed/single (+) Ethnicity Asian (+) Country of birth (0) Length of time living in UK (-) Main role nurse (+)	*Reports weighted % Overall % 602 (25.09%) Subscale % Commission: 346 (14.28%) Omission: 111 (4.57%) Betrayal: 439 (18.11%)	Overall M/SD 1.72 (1.19) Subscale M/SD Commission: 1.71 (1.06) Omission: 1.47 (1.1) Betrayal: 1.8 (1.14)	2	Grant.
Litam et al., 2021	Cross-sectional	109	Healthcare Physicians (37%) Nurses (57%) Other professionals (6.4%)	Convenience May-July 2020	37.50 (12.4)	24%	75.2%	USA	NR	MIES Lower score indicates higher moral injury Unidimensional	(Mental) health Compassion satisfaction (PROQOL) (+) Burnout (PROQOL) (+) Secondary traumatic stress (PROQOL) (+)	Overall % NR Subscale % NR	Overall M/SD 3.03(0.72) Subscale M/SD NR	5	None.

For peer review only - http://bmjopen.bmj.com/site/about/guidelines.xhtml

Litz et al., 2022<br><br>Four studies	Cross-sectional	1: 533<br>2: 363<br>3: 264<br>4: 118	Samples 1, 2 and 3: Veterans<br>Sample 4: Defence members and veterans.	Sample 1: Convenience<br>Samples 2, 3 and 4: NR	Sample 1: 51.87 (9.77)<br>Samples 2, 3 and 4: NR	Sample 1: 71%<br>Sample 2: 73.6 %<br>Sample 3: 67%<br>Sample 4: 65.9 %	NR for all	Sample 1: Canada<br>Sample 2: USA<br>Sample 3: UK<br>Sample 4: Australia	NR for all	MIOS<br>2 subscales (shame-related outcomes and trust violation-related outcomes)	NR at univariate level.	Sample 1:<br>Overall %<br>366 (68.7%)<br>Subscale %<br>MI-Self: 265 (49.7%)<br>MI-Other: 378 (71%)<br>Impact from an MI-Other: 440 (82.5%)<br>Sample 2:<br>Overall %<br>283 (78%)<br>Subscale %<br>MI-Self: 142 (39%)<br>MI-Other: 142 (39%)<br>Impact from an MI-Other: 142 (39%)<br>Sample 3:<br>Overall %<br>264 (100%)<br>Subscale %<br>MI-Self: 174 (65.9%)<br>MI-Other: 170 (64.4%)<br>Impact from an MI-Other: 186 (70.1%)<br>Sample 4:<br>Overall %<br>NR<br>Subscale %<br>MI-Self: 65 (55%)	Sample 1:<br>Overall M/SD<br>25.31(11.38)<br>Subscale M/SD<br>Shame-related outcomes: 11.28(6.64)<br>Trust violation outcomes: 14.03(5.85)<br>Sample 2:<br>Overall M/SD<br>25.14(11.36)<br>Subscale M/SD<br>Shame-related outcomes: 11.36(6.82)<br>Trust violation outcomes: 13.78(5.58)<br>Sample 3:<br>Overall M/SD<br>32.87(10.54)<br>Subscale M/SD<br>Shame-related outcomes: 16.29(6.20)<br>Trust violation outcomes: 16.58(5.35)<br>Sample 4:<br>Overall M/SD<br>27.74(10.42)<br>Subscale M/SD	5<br><br>4: 6	None

For peer review only

												MI-Other: 93 (79%)<br><br>Impact from an MI-Other: 103 (87%	Shame-related outcomes: 12.17(6.35)<br>Trust violation outcomes: 15.56(5.52)		
Maftei & Holman (2021)	Cross-sectional	114	Physicians	Convenience April 2020	38.85 (9.82)	24.6 %	NR	Romania	1.14 %	MIES 4+ (agree)<br>1-5 Likert scale<br>3 subscales (transgressions-others, transgressions-self, betrayal)	(Mental health)<br>Emotional impact (0) *<br>Emotional impact (+) **<br>Physical impact (+)<br>Demographics<br>Age (0)<br>Working experience (0)<br>*non-covid-19 medical unit<br>** covid-19 medical unit	Overall %<br>80 (70.2%)<br><br>Per subscale %<br>Transgressions-others: 61(53.5%)<br>Transgressions-self: 31(27.2%)<br>Betrayal 66(57.9%)<br>Per item %<br>Item 1: 45 (39.5%)<br>Item 2: 39 (34.2%)<br>Item 3: 14 (12.3%)<br>Item 4: 15 (13.2%)<br>Item 5: 20 (17.5%)<br>Item 6: 22 (19.3%)<br>Item 7: 35 (30.7%)<br>Item 8: 37 (32.5%)	Overall<br>2.68(0.90)<br><br>Subscale M/SD<br>Transgressions-others: 3.06(1.26)<br>Transgressions-self: 2.20(1.05)<br>Betrayal 3.06(1.08)	5	None

For peer review only - http://bmjopen.bmj.com/site/about/guidelines.xhtml

												Item 9: 33 (33%)			
Maguen et al., 2022	Cross-sectional	14057	Post 9/11 veterans.<br><br>Army (50.5%)<br>Navy/Coast Guard (17.9%)<br>Marines (13.3%)<br>Air Force (18.3%)	Nationally representative, population-based sampling frame<br>April – August 2018	NR	82.3 %	66.2%	USA	40 %	MIES 4+ (slightly agree)<br>3 subscales (transgressions-others, transgressions-self, betrayal)	Demographics<br>Female gender (+) *<br>Female gender (-) **<br><br>*transgressions-others and betrayal<br>** transgressions-self	Overall %<br>7830 (55.7%)<br>Per subscale %<br>Transgressions-others 6059 (43.1%)<br>Transgressions-self 3064 (21.8%)<br>Betrayal 5651 (40.2%)	Overall M/SD NR<br>Subscale M/SD NR	2	None
Malakoutikhah et al., 2022	Cross-sectional	455	Healthcare<br>Operating room (6.3%)<br>Nursing (73.7%)<br>Anaesthesiology (8.2%)<br>Medicine (7.5%)<br>Midwifery (2.7%)<br>Nursing assistant (1.6%)	Convenience<br>September 2021 – February 2022	NR	16.9 %	NR	Iran	NR	MISS-HP 36.5+<br>Item content varied	(Mental) health<br>General health (GHQ) (+)<br>PTSD (IES-R) (+)	Overall %<br>190 (42.7%)<br>Per item %<br>Scoring strongly agree:<br>Item 2: 13 (5.1%)<br>Item 3: 5 (2%)<br>Item 4: 15 (6%)<br>Item 5: 71(28%)<br>Item 6: 37 (14.6%)<br>Item 7: 71(28%)<br>Item 8: 39 (15.5%)<br>Item 9: 4(1.6%)<br>Item 10: 1 (0.4%)	Overall M/SD 35.66(12.08)	5	None.

For peer review only - http://bmjopen.bmj.com/site/about/guidelines.xhtml

For peer review only

												Item 11: 3<br>(1.2%)			
Mantri et al., 2020	Cross-sectional	181	Healthcare<br>Physicians (70.7%)<br>Nurse (9.4%)<br>Other (19.9%)	Convenience<br>November 2019 – March 2020	NR	63.5 %	74.5%	USA	NR	MISS-HP 36+<br><br>EMIS-M 2 subscales (self-directed MI and other-directed MI)	(Mental) health<br>Burnout (MBI) (+)<br>Anxiety (GAD-7) (+)<br>Depression (PHQ-9) (+)<br>Individual/work characteristics<br>Religiosity (-)<br>*MISS-HP correlations	MISS-HP<br><br>Overall %<br>86 (47.5%)	MISS-HP<br>Overall M/SD<br>36.8(13.3)<br>EMIS-HP<br>Overall M/SD<br>2.01(0.78)<br>Subscales M/SD<br>NR	5	None
Mantri et al., 2021<br><br>Two samples	Cross-sectional	Sample 1: 450<br><br>Sample 2: 1381	Healthcare<br>Sample 1:<br>Nurse (31.8%)<br>Physician (25.8%)<br>APP (18.9%)<br>Chaplaincy (14.2%)<br>Social worker (2.9%)<br>Other (6.4%)<br>Sample 2:<br>Nurse (56.5%)<br>Physician (12%)	Convenience.<br><br>Sample 1:<br><br>April-May 2020<br><br>Sample 2:<br><br>October-November 2020	NR	Sample 1:<br><br>12.4 %<br><br>Sample 2:<br><br>9.5%	Sample 1:<br><br>88.7%<br><br>Sample 2:<br><br>93.3%	Global<br><br>Primarily North America (USA)	Sample 1:<br><br>72.8 %<br><br>Sample 2:<br>73.7 %	MISS-HP 36+	(Mental) health<br>Burnout emotional exhaustion (aMBI) (-)<br>Burnout depersonalisation (aMBI) (-)<br>Burnout reduced personal/professional accomplishment (aMBI) (+)<br>Individual/work characteristics<br>COVID experience (0) *<br>COVID experience (+) **<br>Religiosity (DUREL) (-)<br>Demographics<br>Age (-)	Overall %<br>Sample 1:<br>84 (18.6%)<br>Sample 2:<br>591 (42.8%)	Overall M/SD<br>Sample 1:<br>27.4(11.6)<br>Sample 2:<br>36.4(13.8)	5	None

For peer review only

			APP (19.8%)<br>Housekeeping (0.1%)<br>Food services (0.1%)<br>Interpreter (0.1%)<br>Chaplaincy (0.1%)<br>Social worker (1.3%)<br>Other (19.9%)								Sex (0)<br>Race (0)<br>Marital status (0) *<br>Marital status (+) **<br>Profession (-)<br>*sample 1<br>** sample 2				
Morris, Webb & Devlin, 2022	Cross-sectional	237	Healthcare<br>Dietetics (2.5%)<br>Education (2.5%)<br>Medical Doctor (0.8%)<br>Nursing (Registered) (26.3%)<br>Nursing (Unregistered) (46.6%)<br>Occupational Therapy (5.1%)<br>Psychology (11.4%)<br>Psychiatry (1.3%)	Convenience	NR	34.2 %	78.9%	UK	NR	MIES 6+ (strongly agree)<br>3 subscales (transgressions-others, transgressions-self, betrayal)	(Mental) health<br>Burnout (ProQoL) (+)<br>Secondary traumatic stress (ProQoL) (+)<br>Compassion satisfaction (ProQoL) (-)<br>Individual/work characteristics<br>Impact of COVID-19 (0)<br>Demographics<br>Gender (0)<br>Ethnicity (0)<br>Gendered ethnicity (0)	Overall %<br>183 (78.5%) *<br>Subscale %<br>Transgressions-others: 154 (67%) **<br>Transgressions-self: 79 (34.3%) **<br>Betrayal: 154 (66.1%) *<br>*n = 233<br>**n = 230	Overall M/SD<br>2.90 (1.41)<br>Subscale M/SD<br>Transgressions-others: 3.79(1.86)<br>Transgressions-self: 2.13(1.47)<br>Betrayal 3.38(1.83)	5	None

For peer review only - http://bmjopen.bmj.com/site/about/guidelines.xhtml

			Recreational Co-ordinator (1.7%) Social Work (0.8%) Other (0.4%)												
Rushton et al., 2022	Cross-sectional	595	Healthcare Nurse (58.1%) Physician (11.8%) Respiratory therapist (6.3%) Nurse practitioner/physician assistant (7.1%) Other (16.7%)	Convenience June 2020 – November 2020	NR	NR	82.4%	USA	NR	MISS-HP 36+	Individual/work characteristics Ethical concerns (ECI) (+) Involvement in COVID-19 clinical care (+) Practice location (0) Speciality (0) Demographics Years in profession (-) Profession (0) Education (0) Religious/spiritual preference (+)	Overall % 32.4%	Overall M/SD 30.57(12.11)	5	None
Nieuwsma et al., 2020	Cross-sectional	315	Post 9/11 veterans with warzone experience. Army (67%) Navy (13.2%) Air Force (9.9%) Marines (9.9%)	Convenience	46.36 (10.4)	86.6 %	55.6%	USA	33.5 %	MIES 2 subscales (perpetration and betrayal) MIQ-M 20 items Unidimensional. BMIS 2+ (agree) 2 subscales (event and sequela).	(Mental) health Depression (BDI-2) (+) Suicidal ideation (BSI) (+) Alcohol use disorder (AUDIT) (+) Drug abuse (DAST) (+) PTSD (DTS) (+) *all correlations positive for BMIS, MIES and MIQ-M	Overall % NR Subscale % BMIS-E 137 (44.1%) NR for BMIS-S subscale *n = 311	Overall M/SD BMIS 3.71(4.38) MIES 2.57(1.26) MIQ-M 1.49(0.47) Subscale M/SD NR for all scales *n = 311	5	NR

For peer review only - http://bmjopen.bmj.com/site/about/guidelines.xhtml

Osmann et al., 2022	Cross-sectional	159	Journalists	Convenience	44.72 (12.1)	52%	NR	NR	72 %	Toronto Moral Injury Scale for Journalists<br>3 subscales (organisational, Individuals, and online)	(Mental) health<br>PTSD (PLC-5) (+)<br>Anxiety (BAI) (+)<br>Depression (BDI-II) (+)<br>Demographics<br>Marital status (0)<br>Education (0)	Overall % NR<br>Subscale % NR	Overall M/SD 1.50(1.27)<br>Subscale M/SD<br>Organisational/management: 1.54(1.32)<br>Individuals/Nonmanagement: 1.18(1.19)<br>Online: 1.68(1.27)	5	None
Rodríguez et al., 2021	Cross-sectional	169	Healthcare Physicians (86.39%)<br>Nurses (13.61%)	Convenience	36.04 (10.5)	23.7 %	NR	Honduras	NR	MISS-HP 36+	(Mental) health<br>Depression (PHQ-9 (+)<br>Anxiety (GAD-7) (+)	Overall % 77 (45.6%)	Overall M/SD 34.80(15.81)	5	None
Roth et al., 2022	Cross-sectional	270	Personal Safety Personnel (PSP).<br>Firefighter (19%)<br>Paramedic (44%)<br>Police (17%)<br>Dispatcher (6%)<br>Other (14%)	Convenience	35 (9.5)	63%	85%	Canada and USA	NR	MIA-PSP<br>3 subscales (perpetration, betrayal and emotional sequalae)<br><br>MIES<br>Unidimensional.	(Mental) health<br>Shame (TRSI) (+)<br>Guilt (TRGI) (+)<br>PTSD (PCL-5) (+)<br>Depression, anxiety and stress (DASS-21) (+)<br>Dissociation (MDI) (+)<br>Disability, functioning and health (WHODAS) (+)	Overall % NR for either scale	Overall M/SD<br>MIA-PSP 59.6(21.9)<br>MIES 3.56(1.39)<br>Subscale M/SD<br>MIA-PSP<br>Emotional Sequalae: 25(9.7)<br>Perpetration: 15(8.5)<br>Betrayal: 19.6(7.7)	5	None

For peer review only - http://bmjopen.bmj.com/site/about/guidelines.xhtml

												Subscale M/SD<br>MIES<br>NR		
Russell & Mussap, 2022<br><br>Two studies	Cross-sectional	Study 1: 183<br>Study 2: 393	Study 1:<br><br>Current and former military personnel.<br><br>Currently serving (50.3%)<br><br>Study 2:<br><br>Current and former military personnel.<br><br>Currently serving (39.7%)	Study 1 and 2: Convenience.<br><br>.	Study 1: 43.3 (11.9)<br>Study 2: 44 (12)	Study 1: 83.6 %<br>Study 2: 81.4 %	Study 1: 84.7%<br>Study 2: 81.7%	Study 1: Global but mainly Australian (89%)<br>Study 2: Global but primarily Australian (85%)	Study 1: NR<br>Study 2: NR	Study 1:<br>MIES 4+ (slightly agree)<br>3 subscales (transgressions-others, transgressions-self, betrayal)<br>Study 2:<br>MIES 4+ (slightly agree)<br>3 subscales (transgressions-others, transgressions-self, betrayal)	Study 1:<br>(Mental) health<br>PTSD (PCL-M) (+)<br>Post-traumatic growth (PTGI-X) (0)<br>Personal well-being (PWI) (-)<br>Individual/work characteristics:<br>Intrusive rumination (ERRI) (+)<br>Deliberate rumination (ERRI) (+)<br>Exposure to danger/combat (EDC) (0) *<br>Exposure to danger/combat (EDC) (+) **<br>Witnessing the consequences of war (WDW) (+)<br>Demographics<br>Age (0)<br>Study 2:<br>(Mental) health<br>PTSD (PCL-M) (+)<br>Post-traumatic growth (PTGI-X) (0)	Study 1:<br>Overall %<br>132 (72.1%)<br>Subscale %<br>Transgressions-self: 97 (53%)<br>Transgressions-others: 48 (26.2%)<br>Betrayal: 99 (53.8%)<br>Study 2:<br>Overall %<br>300 (76.6%)<br>Subscale %<br>Transgressions-self: 109 (59.4%)<br>Transgressions-others: 62 (33.8%)<br>Betrayal: 112 (61.4%)	Study 1:<br>Overall M/SD: 2.25(1.12)<br>Subscale M/SD:<br>Transgressions-self: 2.88(1.63)<br>Transgressions-others: 1.73(1.20)<br>Betrayal: 2.54(1.45)<br>Study 2:<br>Overall M/SD: 2.60(1.28)<br>Subscale M/SD:<br>Transgressions-self: 3.23(1.74)<br>Transgressions-others: 2.05(1.42)<br>Betrayal: 2.9(1.58)	5	None

For peer review only - http://bmjopen.bmj.com/site/about/guidelines.xhtml

											Personal well-being (PWI) (-)<br>Individual/work characteristics:<br>Threat bias (IBIP) (+)<br>Exposure to danger/combat (EDC) (+) ***<br>Exposure to danger/combat (EDC) (0) ****<br>Witnessing the consequences of war (WDW) (+)<br>Demographics<br>Age (0)<br>*Transgressions-self<br>**Transgressions-other and betrayal<br>***Transgressions-self and transgressions-others<br>****Betrayal				
Saba et al., 2022	Longitudinal.<br><br>12-month time point.	1005	Veterans. Air Force (14.53%)<br>Army (65.27%)<br>Marine Corps (9.65%)<br>Navy (10.55%	Convenience February 2021.	34.83 (3.61)	91.14 %	84.58%	NR	21.3 %	MIES 4+ (slightly agree)<br>3 subscales (transgressions-others, transgressions-self, betrayal)	(Mental) health<br>PTSD (PCL-5) (+)<br>Depression (PHQ-9) (+)<br>Anxiety (GAD-7) (+)<br>Anger (DAR-R) (+)	Overall %<br>881 (87.66%)<br>Subscale %<br>Transgressions -self: 802 (79.80%)<br>Transgressions -others: 303 (30.15%)<br>Betrayal: 300 (29.85%)	Overall M/SD: 2.75(0.81)<br>Subscale M/SD:<br>Transgressions -self: 2.65(1.20)<br>Transgressions -others: 2.92(0.84)<br>Betrayal: 2.58(1.11)	5	None

For peer review only

Senger, Torres & Ratcliff, 2022	Cross-sectional	242	First responders. In person sample: (n=91) Firefighter (37.4%) Law enforcement (20.90%) Paramedic (2.20%) EMT (4.40) Multiple (35.20%) Online sample (n=151): Firefighter (6%) Law enforcement (38.40%) Paramedic (16.60%) EMT (5.30%) Search and rescue worker (3.30%) Other (1.30%) Multiple (29.10%)	Convenience January – December 2020	NR	In person sample: 97.8 % Online sample: 75.50 %	Online sample: 83.30% In person sample: 88.70%	USA	NR	MIES 4+ (slightly agree) 3 subscales (transgressions-others, transgressions-self, betrayal)	Individual/work characteristics: Pre vs post COVID (-) Military service (-) Demographics Occupation type (+) Time in occupation (+) Age (+) Gender (0) Race/ethnicity (0) Education (0) Marital status (+) Income (0) Religion (0)	Overall % 98 (40.65%) Subscale % Transgressions-self: 51 (21.23%) Transgressions-others: 147 (60.75%) Betrayal: 97 (39.97%) Per item % Item 1: 174 (72.2%) Item 2: 119 (49.3%) Item 3: 48 (19.7%) Item 4: 56 (23.3%) Item 5: 53 (21.8%) Item 6: 49 (20.1%) Item 7: 102 (42%) Item 8: 94 (38.7%) Item 9: 95 (39.2%)	Overall M/SD: 2.7 (1.1) Subscale M/SD: Transgressions-self: 2.1 (1.3) Transgressions-others: 3.8 (1.4) Betrayal: 2.9 (1.5)	6

For peer review only

Stanojević & Čartolovni, 2022	Cross-sectional	162	Nurses. Palliative nurses (47.5%) Oncology nurses (52.5%)	Convenience January - June 2021.	NR	13%	NR	Croatia	64.8 %	MISS-HP 36+	(Mental) health: Moral distress (+) Individual/work characteristics: Left or considered leaving clinical position (+) Considering about leaving clinical position (+) Demographics Age (0) Work experience (0) Education (0) Gender (0) Specialisation (0)	Overall % NR	Overall M/SD 37.63(12.19)	5	None
Testoni et al., 2022	Cross-sectional	270	Healthcare professionals. Physician (65% Other healthcare provider (35%)	Convenience July - December 2020	44.5 (12.2)	30%	NR	Italy	NR	MIES 4+ (slightly agree) 3 subscales (transgressions-others, transgressions-self, betrayal)	(Mental) health: Alienation (+) Individual/work characteristics: Self-efficacy (NYPQS) (-) Humanisation patient (HTAS) (0) Humanisation colleague (HTAS) (-) Overall humanisation (HTAS) (-) Dehumanisation patient (HTAS) (+) Dehumanisation colleague (HTAS) (+) Overall dehumanisation (HTAS) (+)	Overall % 212 (78.5%) Subscale % Transgressions-self: 78 (28.9%) Transgressions-others: 166 (61.5%) Betrayal 166 (61.55)	Overall M/SD 2.62(1.18) Subscale M/SD Transgressions-self: 2.06(1.45) Transgressions-others: 3.23(1.56) Betrayal 2.96(1.48)	5	None

For peer review only

For peer review only - http://bmjopen.bmj.com/site/about/guidelines.xhtml

Thomas, Weiss, Forkus & Contractor, 2022	Cross-sectional	496	Veterans. Army (63.7%) Navy (9.9%) Air Force (19%) Marines (6.3%) Coast Guard (1.2%)	Convenience	37.80 (11.4)	70.5 %	71.1%	USA	18.8 %	MIES 3 subscales (transgressions-others, transgressions-self, betrayal)	(Mental) health: Alcohol misuse (AUDIT) (+) Individual/work characteristics: Total religiosity (DUREL) (+) Organisational religiosity (DUREL) (+) Nonorganizational religiosity (DUREL) (0) Intrinsic religiosity (DUREL) (0)	Overall % NR Subscale % NR	Overall M/SD 3.19(1.40) Subscale M/SD NR	5	NR
Trifunovic-Koenig et al., 2022	Cross-sectional	156	Healthcare Physician (16%) Nurse (general) (33.3%) Paediatric nurse (1.3%) Paramedic (23.7%) Geriatric nurse (9%) Speech therapist (clinical linguistic) (7.1%) Medical assistant (3.2%) Medical student (1.3%)	Convenience March - June 2021	40.7 (10.2)	34.6 %	NR	German speaking	3.5 %	MISS-HP 28.5+ 9 item scale	(Mental) health: Psychological distress (+) Physical distress (+) Individual/work characteristics: Colleague support (+) Supervisor support (+) Institutional support (+) Professional self-efficacy (+) Turnover intentions (+) Absenteeism (+) Resilience (+)	Overall % 85 (54.5%)	Overall M/SD 32.31(13.26)	5	None

For peer review only - http://bmjopen.bmj.com/site/about/guidelines.xhtml

			Physiotherapist (0.6%)<br>Midwife (0.6%)<br>Respiratory therapist (1.3%)<br>Administration worker/Technician (0.6%)												
Ulusoy & Çelik 2022	Cross-sectional	124	Healthcare workers.<br>Doctors (67.7%)<br>Nurses (21.8%)<br>Medical secretaries (4.8%)<br>Other support staff (5.6%)	Convenience<br>January - April 2021	NR	NR	NR	Turkey	36 %	MIES 4+ (slightly agree)<br>Unidimensional	(Mental) health<br>Burnout emotional exhaustion (MBI) (+)<br>Burnout depersonalisation (MBI) (+)<br>Burnout reduced personal accomplishment (MBI) (0)<br>Obstruction to valued living (VQ) (+)<br>Depression, anxiety, and stress (DASS-21) (+)<br>Psychological inflexibility and experiential avoidance (AAQ-II) (+)	Overall %<br>116 (93.5%)<br><br>Subscale %<br>Transgressions -self: 78 (62.9%)<br>Transgressions -others: 109 (87.9%)<br>Betrayal: 96 (77.4%)	Overall M/SD<br>3.42(0.99)<br><br>Subscale M/SD<br>Transgressions -self: 2.82(1.31)<br>Transgressions -others: 4.51(1.34)<br>Betrayal: 3.51(1.28)	5	None

For peer review only

Williamson Murphy & Greenberg, 2022	Cross-sectional	90	Veterinary	Convenience December 2020 – May 2021	36.8 (10.2)	12.2 %	70%	UK	27 %	MIES 5+ (moderately agree)<br>3 subscales (transgressions-others, transgressions-self, betrayal)	(Mental) health<br>PTSD (ITQ) (+)<br>Alcohol (AUDIT-C) (0)<br>Common mental disorders (PHQ-4)) (0)<br><br>Demographics<br>Age (0)<br>Years of experience (0)<br>Role type (0)	Overall %<br>80 (88.9%)<br><br>Subscale %<br><br>Transgressions-self: 51 (56.7%)<br>Transgressions-others: 63 (70%)<br>Betrayal: 62 (68.9%)	Overall M/SD<br>NR<br>Subscale M/SD<br>NR	5	None
Zahiriharsini et al., 2022<br><br>Narrative synthesis	Cross-sectional	572	Healthcare workers and leaders<br>Nurse (60.5%)<br>Patient support worker (2.9%)<br>Physician (1%)<br>Manager (11.1)<br>Others (25%)	Convenience February - June 2021	40.30 (10.5)	11.4 %	NR	Canada Quebec	NR	EMIS-M<br>low MI: <2.5, moderate MI: 2.5 ≤ score <3.5, and high MI: ≤ 3.5<br><br>2 subscales (Self-directed MI and other-directed MI)	NR at univariate level	Overall %<br>Moderate to high: 79 (13.81%)<br>Subscale %<br>Self-directed MI:<br>Moderate to high: 58 (10.14%)<br>Other-directed MI:<br>Moderate to high: 167 (29.19%)	Overall M/SD: NR<br>Subscale M/SD: NR	5	None

For peer review only

Levi-Belz Shemesh & Zerach 2022	Cross-sectional	190	Combat veterans. Infantry units (58.4%) Armoured corps (13.2%) Artillery corps (7.4%) Navy (4.7%) Military police, intelligence, or Air Force (13.6%)	Convenience<br><br>January - July 2018.	26 (2.61)	87%	NR	Israel	NR	MIES 4+ (slightly agree)<br><br>3 subscales (transgressions-others, transgressions-self, betrayal)<br><br>Average time since discharge 4.26(2.37)	(Mental) health Depression (PHQ-8) (+) Current suicide ideation (SBQ-R) (+) * current suicide ideation (SBQ-R) (0) ** Self-disclosure (DDI) (0) Individual/work characteristics Combat exposure (+) Time from demobilisation (0) *Transgressions-self and betrayal **Transgressions-others	Overall % NR Per subscale % Transgressions -others: 69 (36.1%) Transgressions -self: 44 (23.2%) Betrayal: 67 (35.2%) Per item % Item 1: 61 (32.1%) Item 2: 47 (24.7%) Item 7: 52 (27.4%)	Overall M/SD NR Subscale M/SD Transgressions -others: 2.41(1.49) Transgressions -self: 1.81(1.08) Betrayal: 2.02(1.06)	5	NR
Schwartz Halperin & Levi-Belz, 2022	Cross-sectional	336	Veterans<br><br>Armoured corps (9.2%) Artillery corps (8.3%) Combat engineering (8%) Other (34.6%)	Convenience<br><br>August - November 2019	26.16 (3.06)	83.9 %	NR	Israel	58 %	MIES 4+ (slightly agree)<br><br>1 subscale (transgressions-self)<br><br>Average time since discharge 4.2(2.45)	(Mental) health Shame (TRSI) (+) Current suicidal ideation (SBQ-R) (+) Individual/work characteristics Collective hatred (HAS) (0) All correlations are for transgressions-self subscale.	Overall % NR Per subscale % NR Per item % Item 3: (24.18%)	Overall M/SD NR Subscale M/SD Transgressions -self: 2.12(1.26)	5	None

Note. For longitudinal studies, data extracted from baseline wave unless only available at a subsequent wave. Associations reported use total scale but if no total scale associations are reported, then subscales associations were reported. CPS = Child Protection Services; RR = Response rate; NR = No response; MIES = Moral injury events scales; EMIS-M = Expressions of Moral Injury Scale-Military Version; MISS-HP = Moral Injury Symptoms Scale – Health Professionals; MIA-PSP = Moral Injury Assessment for Public Safety Personnel; MIQ-M = Moral Injury Questionnaire—Military version, BMIS = Brief Moral Injury Screen; BMIS-E = Brief Moral Injury Screen Event subscale, EMIS-SF = Expressions of Moral Injury Scale-Short Form; MIQ-T = Moral Injury Questionnaire—Teacher version; MIQ-M = Moral Injury Questionnaire—Military version; MIOS = Moral Injury Outcome Scale

For peer review only

Supplementary Table 3.
Hoy Risk of Bias for studies included in the systematic review

Authors (Year)	Representative of national population	Representative of target population	Random selection	Non-response bias	Directly collected	Acceptable case definition	Reliable and valid instrument	Same mode of data collection	Appropriate numerator(s) and denominator(s) for the parameter of interest	Hoy score
**PTED only studies**										
Dunn & Sensky 2018	1	1	1	1	0	0	0	0	1	5
Linden & Rotter 2019	1	1	1	1	0	0	0	0	1	5
Michailidis & Cropley 2018	1	1	1	1	0	0	0	0	1	5
Sensky, Salimu, Ballard & Pereira 2015	1	0	1	1	0	0	0	0	1	4
Sabic, Sabic, & O Baltic-Mujanovic 2018	1	1	1	1	0	0	0	0	1	5
Michailids & Cropley 2017	1	1	1	1	0	0	0	0	1	5
Karatuna & Gok 2014	1	1	1	1	0	0	0	0	1	5
Rubab & Tariq 2022	1	1	1	1	0	0	0	0	1	5
Saleem, Bashir & Abrar 2022	1	1	0	1	0	0	0	1	1	5
Shin & You 2021	1	1	1	1	0	0	0	0	1	5
**Moral injury and PTED study**										

For peer review only - http://bmjopen.bmj.com/site/about/guidelines.xhtml

Brennan, McKay & Cole 2022	1	0	1	0	0	0	0	0	0	2
**Moral injury only studies**										
Bryan et al., 2016	1	1	1	1	0	0	0	0	1	5
Currier, Holland, Drescher & Foy 2015	0	0	1	1	0	0	0	0	1	3
Currier, Holland, Rojas-Flores, Herrera & Foy 2015	1	1	1	0	0	0	0	0	1	4
Currier et al., 2017	1	1	1	1	0	0	0	0	1	5
Forkus,Juliana & Weiss 2019	1	1	1	1	0	0	0	0	1	5
Frankfurt et al., 2018.	1	0	1	1	0	0	0	0	1	4
Griffin et al., 2020	1	1	1	1	0	0	0	0	1	5
Jinkerson & Battles 2019	1	1	1	0	0	0	0	0	1	4
Jordan & Nash 2017	1	1	1	1	0	0	0	0	1	5
Lee, Aldwin & Kang 2022	1	1	1	1	0	0	0	0	1	5
Ogle, Reichwald, & Rutland 2018	1	1	1	1	0	0	0	0	1	5
Wisco et al., 2017	0	0	0	1	0	0	0	0	0	1
Zerach & Levi-Belz., 2017	1	1	1	0	0	0	0	0	1	4
Papazoglou et al., 2019	1	1	1	0	0	0	0	0	1	4
Richardson, Chesnut et al., 2020	0	0	0	1	0	0	0	0	1	2
Feinstein, Pavisian, Storm., 2018	1	1	1	1	0	0	0	0	1	5
Sugrue 2020	1	1	1	1	0	0	0	0	1	5

For peer review only

For peer review only - http://bmjopen.bmj.com/site/about/guidelines.xhtml

Aldridge, Scott, Paskell 2019	1	1	1	1	0	0	0	0	1	5
Andrukonis, Protopopov. 2020	1	1	1	1	0	0	0	0	1	5
Haight, Sugrue & Calhoun 2017	1	1	1	1	0	0	0	0	1	5
Battles et al., 2019	1	1	1	1	0	0	0	0	1	5
Braitman et al., 2018	1	1	1	1	0	0	0	0	1	5
Lancaster & Harris 2018	1	1	1	1	0	0	0	0	1	5
Lancaster 2018	1	1	1	1	0	0	0	0	1	5
Akhtar et al., 2022	1	1	1	1	0	0	0	1	1	6
Qi et al., 2022	1	1	1	1	0	0	0	0	1	5
Ansalem et al., 2021	1	1	1	1	0	0	0	0	1	5
Perez, Larson & Bair 2021	1	1	1	1	0	0	0	0	1	5
Benatoz, Zerach & Levi-Belz 2022	1	1	1	1	0	0	0	0	1	5
Borges et al., 2021	1	1	1	1	0	0	0	0	1	5
Boscarino et al., 2022	1	0	0	1	0	0	0	0	1	3
Brady et al., 2021	0	0	1	1	0	0	0	0	1	4
Brady et al., 2022	1	1	1	1	0	0	0	0	1	6
Chandrabhatla et al., 2022	1	1	1	1	0	0	0	0	1	5
Dale et al., 2021	1	1	1	1	0	0	0	0	1	5
Plouffe et al., 2021	0	0	0	1	0	0	0	0	1	2

For peer review only

For peer review only - http://bmjopen.bmj.com/site/about/guidelines.xhtml

Nichter et al., 2020	0	0	0	1	0	0	0	0	0	1
Fitzpatrick et al., 2022	1	1	1	1	0	0	0	0	1	5
Hagerty & Williams 2022	1	1	1	1	0	0	0	0	1	5
Hamrick et al., 2022	1	1	1	1	0	0	0	0	1	5
Hines et al., 2020	1	1	1	1	0	0	0	0	1	5
Hinkel et al., 2022	1	1	1	1	0	0	0	0	1	5
Kelley et al., 2021	1	1	1	1	0	0	0	0	1	5
La Fleur et al., 2020	1	1	1	1	0	0	0	1	1	6
Lamb et al., 2021	1	0	0	1	0	0	0	0	0	2
Litam et al., 2021	1	1	1	1	0	0	0	0	1	5
Litz et al., 2022 Samples 1, 2 and 3	1	1	1	1	0	0	0	0	1	5
Litz et al., 2022 Sample 4	1	1	1	1	0	0	0	1	1	6
Maftei & Holman 2021	1	1	1	1	0	0	0	0	1	5
Maguen et al., 2021	0	0	0	1	0	0	0	0	1	2
Malakoutikhah et al., 2022	1	1	1	1	0	0	0	0	1	5
Mantri et al., 2020	1	1	1	1	0	0	0	0	1	5
Mantri et al., 2021	1	1	1	1	0	0	0	0	1	5
Morris, Webb & Devlin 2022	1	1	1	1	0	0	0	0	1	5

For peer review only - http://bmjopen.bmj.com/site/about/guidelines.xhtml

Rushton et al., 2022	1	1	1	1	0	0	0	0	1	5
Nieuwsma et al., 2020	1	1	1	1	0	0	0	0	1	5
Osmann et al., 2022	1	1	1	1	0	0	0	0	1	5
Rodríguez et al., 2021	1	1	1	1	0	0	0	0	1	5
Roth et al., 2022	1	1	1	1	0	0	0	0	1	5
Russel & Mussap 2022	1	1	1	1	0	0	0	0	1	5
Saba et al., 2022	1	1	1	1	0	0	0	0	1	5
Senger, Torres & Ratcliff 2022	1	1	1	1	0	0	0	1	1	6
Stanojević & Čartolovni 2022	1	1	1	1	0	0	0	0	1	5
Testoni et al., 2022	1	1	1	1	0	0	0	0	1	5
Thomas et al., 2022	1	1	1	1	0	0	0	0	1	5
Trifunovic-Koenig, et al 2022	1	1	1	1	0	0	0	0	1	5
Ulusoy & Çelik 2022	1	1	1	1	0	0	0	0	1	5
Williamson, Murphy & Greenberg 2022	1	1	1	1	0	0	0	0	1	5
Zahiriharsini et al., 2022	1	1	1	1	0	0	0	0	1	5
Levi-Belz, Shemesh & Zerach 2022	1	1	1	1	0	0	0	0	1	5
Schwartz, Halperin & Levi-Belz 2022	1	1	1	1	0	0	0	0	1	5

Note. 1 = yes (high risk), 0 = no (low risk) 7-9 = High, 4-6 = Medium, 0-3 = Low. PTED = post-traumatic embitterment disorder

For peer review only

For peer review only - http://bmjopen.bmj.com/site/about/guidelines.xhtml

Supplementary Figures: Forest Plots for Meta-Analysis Findings

*Analyses using **prevalence** estimates as outcome*

Supplementary Figure 1.

PTED pooled prevalence estimates, using 1.6 cut off.

Study	Cases	Total	Prevalence	95% C.I.
Brennan 2022	121	400	30.25	[25.78; 35.01]
Dunn 2018	37	79	46.84	[35.51; 58.40]
Katatuna 2014	106	397	26.70	[22.41; 31.34]
Michailids 2018	55	352	15.62	[11.99; 19.85]
Michailids 2017	253	337	75.07	[70.10; 79.60]
Rubab 2022	121	300	40.33	[34.74; 46.12]
Sabic 2018	15	87	17.24	[9.98; 26.84]
Sensky 2015	68	236	28.81	[23.12; 35.05]
Shin 2022	581	1074	54.10	[51.06; 57.11]
**Random effects model**		**.**	**36.65**	**[24.38; 49.85]**

Heterogeneity: I^2 = 98%, t^2 = 0.0403, c_8^2 = 455.07 (p < 0.01)

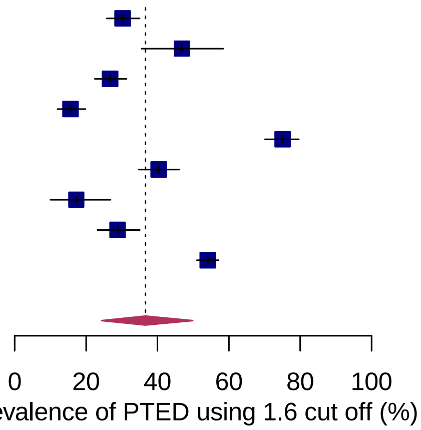

For peer review only

Supplementary Figure 2.

PTED pooled prevalence estimates, using 2 cut off.

Study	Cases	Total	Prevalence	95% C.I.
Brennan 2022	77	400	19.25	[15.50; 23.46]
Dunn 2018	28	79	35.44	[25.00; 47.01]
Katatuna 2014	74	397	18.64	[14.93; 22.83]
Linden 2019	26	102	25.49	[17.38; 35.08]
Michailidis 2017	209	337	62.02	[56.60; 67.22]
Rubab 2022	77	300	25.67	[20.82; 31.00]
Sabic 2018	9	87	10.34	[4.84; 18.73]
Sensky 2015	43	236	18.22	[13.51; 23.75]
**Random effects model**		**.**	**26.03**	**[16.09; 37.38]**

Heterogeneity: I^2 = 97%, t^2 = 0.0293, c_7^2 = 232.77 (p < 0.01)

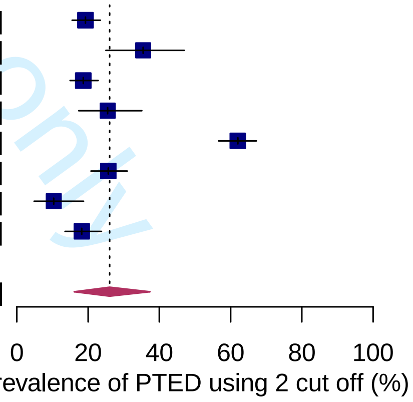

For peer review only - http://bmjopen.bmj.com/site/about/guidelines.xhtml

Supplementary Figure 3.
PTED pooled prevalence estimates, using 2.5 cut off.

Study	Cases	Total	Prevalence	95% C.I.
Brennan 2022	30	400	7.50	[5.12; 10.53]
Katatuna 2014	41	397	10.33	[7.51; 13.75]
Michailidis 2017	152	337	45.10	[39.71; 50.59]
Rubab 2022	26	300	8.67	[5.74; 12.44]
Sabic 2018	1	87	1.15	[0.03; 6.24]
Sensky 2015	21	236	8.90	[5.59; 13.28]
Shin 2022	102	1074	9.50	[7.81; 11.41]
**Random effects model**		**.**	**11.21**	**[4.02; 21.32]**

Heterogeneity: I^2 = 97%, t^2 = 0.0334, c^2_6 = 237.59 (p < 0.01)

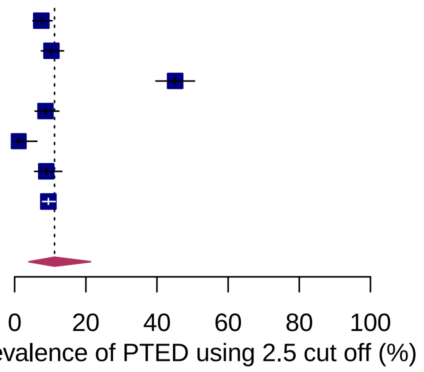

For peer review only

Supplementary Figure 4.
Any MIES item pooled prevalence estimates, split by occupation.

Study	Cases	Total	Prevalence	95% C.I.
**Healthcare**				
Amsalem 2021	267	350	76.29	[71.47; 80.65]
Borges 2021	109	211	51.66	[44.70; 58.57]
Brady 2021	237	390	60.77	[55.73; 65.65]
Brady 2022	268	377	71.09	[66.23; 75.62]
Brennan 2022	289	400	72.25	[67.58; 76.58]
Chandrabhatla 2022	60	78	76.92	[66.00; 85.71]
Lamb 2021	602	2166	27.79	[25.91; 29.73]
Maftei 2021	80	114	70.18	[60.89; 78.38]
Morris 2022	183	233	78.54	[72.71; 83.63]
Testoni 2022	212	270	78.52	[73.13; 83.27]
Ulusoy 2022	116	124	93.55	[87.68; 97.17]
Williamson 2022	80	90	88.89	[80.51; 94.54]
**Random effects model**		**.**	**71.40**	**[60.72; 81.01]**
Heterogeneity: I^2 = 99%, t^2 = 0.0382, c^2_{11} = 1160.88 (p < 0.01)				
**Armed forces**				
Lee 2020	176	354	49.72	[44.39; 55.05]
Maguen 2022	7830	14057	55.70	[54.88; 56.53]
Nichter 2021	535	1321	40.50	[37.84; 43.20]
Russell 2022 study one	132	183	72.13	[65.04; 78.49]
Russell 2022 study two	300	393	76.34	[71.82; 80.45]
Saba 2022	881	1005	87.66	[85.47; 89.63]
Wisco 2017	223	564	39.54	[35.48; 43.71]
Zerach 2017	92	191	48.17	[40.90; 55.50]
**Random effects model**		**.**	**59.53**	**[45.90; 72.44]**
Heterogeneity: I^2 = 99%, t^2 = 0.0382, c^2_7 = 830.68 (p < 0.01)				
**First responders**				
Papazoglou 2019	289	390	74.10	[69.45; 78.38]
Senger 2022	98	242	40.50	[34.26; 46.97]
**Random effects model**		**.**	**57.86**	**[30.91; 82.51]**
Heterogeneity: I^2 = 99%, t^2 = 0.0382, c^2_1 = 71.65 (p < 0.01)				
**Education**				
Sugrue 2020	191	218	87.61	[82.49; 91.68]
**Random effects model**		**.**	**67.00**	**[59.01; 74.54]**

Heterogeneity: I^2 = 99%, t^2 = 0.0398, c^2_{22} = 2212.51 (p = 0)
Residual heterogeneity: I^2 = 99%, t^2 = 0.0382, c^2_{19} = 2063.21 (p = 0)
Test for subgroup differences: c^2_3 = 3.95, df = 3 (p = 0.27)

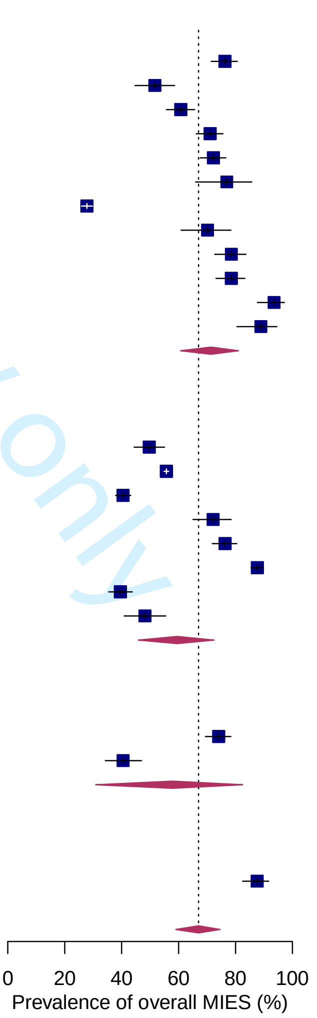

For peer review only - http://bmjopen.bmj.com/site/about/guidelines.xhtml

Supplementary Figure 5.

MIES pooled perpetration prevalence estimates.

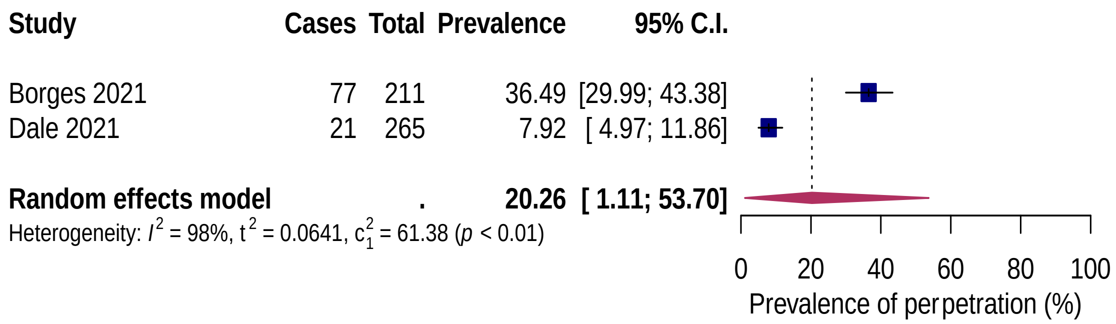

Supplementary Figure 6.

MIES transgressions-self pooled prevalence estimates, split by occupation.

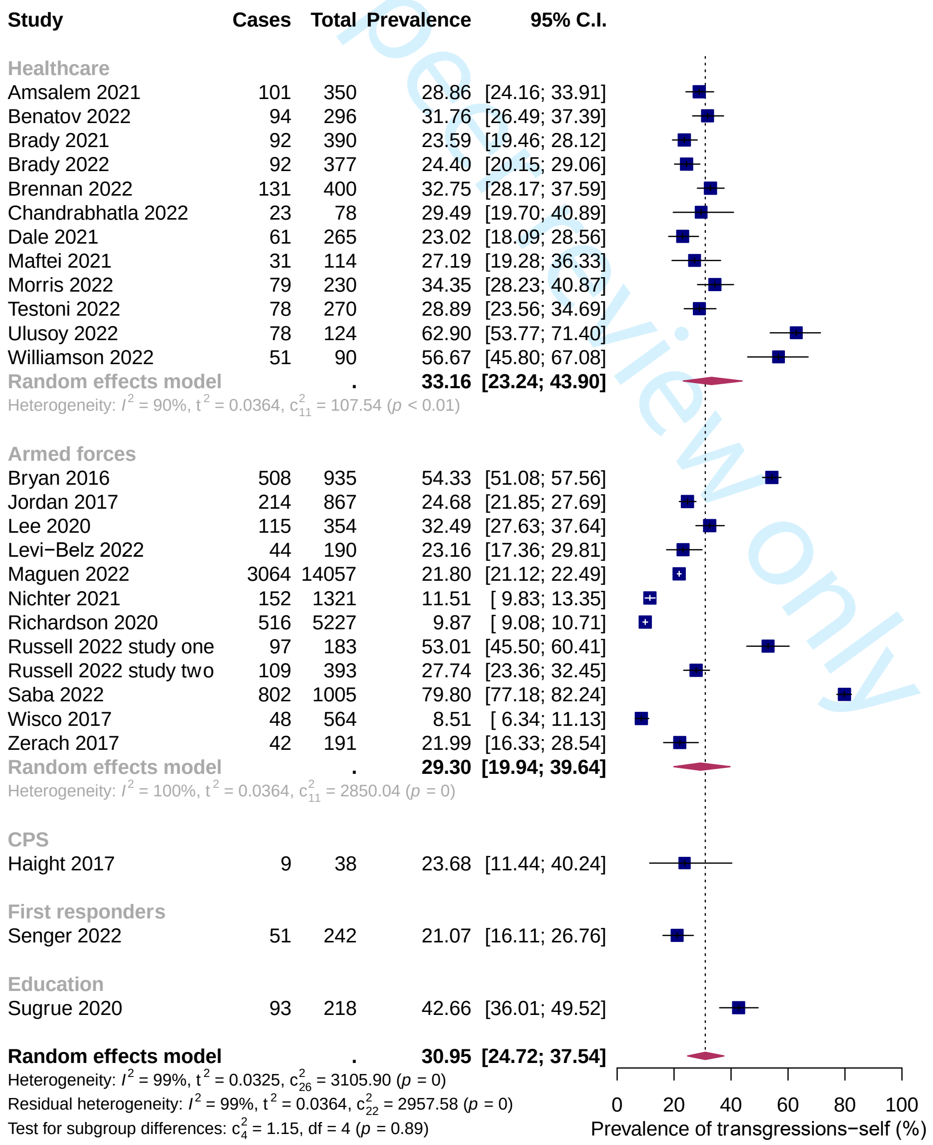

For peer review only

Supplementary Figure 7.
MIES transgressions-others pooled prevalence estimates, split by occupation.

Study	Cases	Total	Prevalence	95% C.I.
**Healthcare**				
Amsalem 2021	215	350	61.43	[56.11; 66.56]
Benatov 2022	146	296	49.32	[43.49; 55.17]
Brady 2021	189	390	48.46	[43.40; 53.55]
Brady 2022	197	377	52.25	[47.08; 57.39]
Brennan 2022	213	400	53.25	[48.23; 58.22]
Chandrabhatla 2022	49	77	63.64	[51.88; 74.30]
Dale 2021	6	265	2.26	[0.84; 4.86]
Maftei 2021	61	114	53.51	[43.93; 62.90]
Morris 2022	154	230	66.96	[60.47; 73.00]
Testoni 2022	166	270	61.48	[55.39; 67.32]
Ulusoy 2022	109	124	87.90	[80.83; 93.07]
Williamson 2022	63	90	70.00	[59.43; 79.21]
Random effects model		.	**54.88**	**[43.91; 65.62]**
Heterogeneity: $I^2 = 98\%$, $t^2 = 0.0360$, $c^2_{11} = 591.65$ ($p < 0.01$)				
**Armed forces**				
Bryan 2016	363	935	38.82	[35.69; 42.03]
Lee 2020	130	354	36.72	[31.69; 41.98]
Levi−Belz 2022	69	190	36.32	[29.48; 43.59]
Maguen 2022	6059	14057	43.10	[42.28; 43.93]
Nichter 2021	316	1321	23.92	[21.64; 26.32]
Russell 2022 study one	48	183	26.23	[20.02; 33.23]
Russell 2022 study two	62	393	15.78	[12.31; 19.76]
Saba 2022	303	1005	30.15	[27.32; 33.09]
Wisco 2017	126	564	22.34	[18.97; 26.01]
Zerach 2017	64	191	33.51	[26.86; 40.68]
Random effects model		.	**30.36**	**[20.07; 41.76]**
Heterogeneity: $I^2 = 98\%$, $t^2 = 0.0360$, $c^2_9 = 475.46$ ($p < 0.01$)				
**CPS**				
Haight 2017	35	38	92.11	[78.62; 98.34]
**First responders**				
Senger 2022	147	242	60.74	[54.28; 66.94]
**Education**				
Sugrue 2020	175	218	80.28	[74.37; 85.34]
**Random effects model**		**.**	**47.73**	**[38.04; 57.51]**

Heterogeneity: $I^2 = 98\%$, $t^2 = 0.0607$, $c^2_{24} = 1464.59$ ($p < 0.01$)
Residual heterogeneity: $I^2 = 98\%$, $t^2 = 0.0360$, $c^2_{20} = 1067.12$ ($p < 0.01$)
Test for subgroup differences: $c^2_4 = 20.01$, df = 4 ($p < 0.01$)

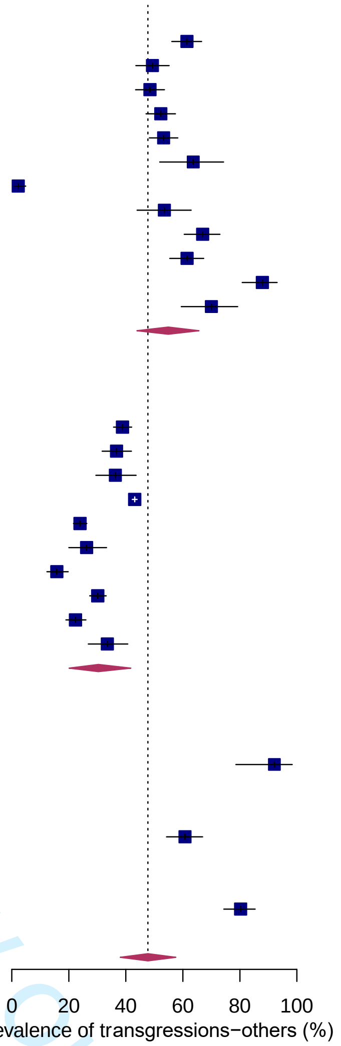

For peer review only

Supplementary Figure 8.

MIES betrayal pooled prevalence estimates, split by occupation.

Study	Cases	Total	Prevalence	95% C.I.
**Healthcare**				
Amsalem 2021	205	350	58.57	[53.21; 63.78]
Benatov 2022	184	296	62.16	[56.37; 67.71]
Borges 2021	88	211	41.71	[34.98; 48.67]
Brady 2021	166	390	42.56	[37.60; 47.64]
Brady 2022	210	377	55.70	[50.53; 60.79]
Brennan 2022	227	400	56.75	[51.74; 61.66]
Chandrabhatla 2022	37	78	47.44	[36.01; 59.07]
Maftei 2021	66	114	57.89	[48.29; 67.08]
Morris 2022	154	233	66.09	[59.63; 72.15]
Testoni 2022	166	270	61.48	[55.39; 67.32]
Ulusoy 2022	96	124	77.42	[69.04; 84.44]
Williamson 2022	62	90	68.89	[58.26; 78.23]
Random effects model		.	**58.12**	**[50.96; 65.12]**
Heterogeneity: $I^2 = 89\%$, $t^2 = 0.0147$, $c^2_{11} = 98.31$ ($p < 0.01$)				
**Armed forces**				
Bryan 2016	540	935	57.75	[54.51; 60.94]
Jordan 2017	252	867	29.07	[26.06; 32.21]
Lamb 2021	439	2166	20.27	[18.59; 22.02]
Lee 2020	121	354	34.18	[29.25; 39.38]
Levi−Belz 2022	67	190	35.26	[28.49; 42.51]
Maguen 2022	5651	14057	40.20	[39.39; 41.02]
Nichter 2021	189	1321	14.31	[12.46; 16.31]
Russell 2022 study one	99	183	54.10	[46.59; 61.47]
Russell 2022 study two	112	393	28.50	[24.09; 33.24]
Saba 2022	300	1005	29.85	[27.03; 32.79]
Wisco 2017	142	564	25.18	[21.64; 28.97]
Zerach 2017	59	191	30.89	[24.42; 37.97]
Random effects model		.	**32.67**	**[26.29; 39.38]**
Heterogeneity: $I^2 = 99\%$, $t^2 = 0.0147$, $c^2_{11} = 1001.5$ ($p < 0.01$)				
**CPS**				
Haight 2017	12	38	31.58	[17.50; 48.65]
**First responders**				
Papazoglou 2019	250	390	64.10	[59.12; 68.87]
Senger 2022	97	242	40.08	[33.86; 46.55]
Random effects model		.	**52.32**	**[35.23; 69.13]**
Heterogeneity: $I^2 = 97\%$, $t^2 = 0.0147$, $c^2_{1} = 35.05$ ($p < 0.01$)				
**Education**				
Sugrue 2020	148	218	67.89	[61.25; 74.04]
**Random effects model**		.	**46.03**	**[39.54; 52.59]**

Heterogeneity: $I^2 = 98\%$, $t^2 = 0.0300$, $c^2_{27} = 1764.34$ ($p = 0$)
Residual heterogeneity: $I^2 = 98\%$, $t^2 = 0.0147$, $c^2_{23} = 1134.87$ ($p < 0.01$)
Test for subgroup differences: $c^2_4 = 30.29$, df = 4 ($p < 0.01$)

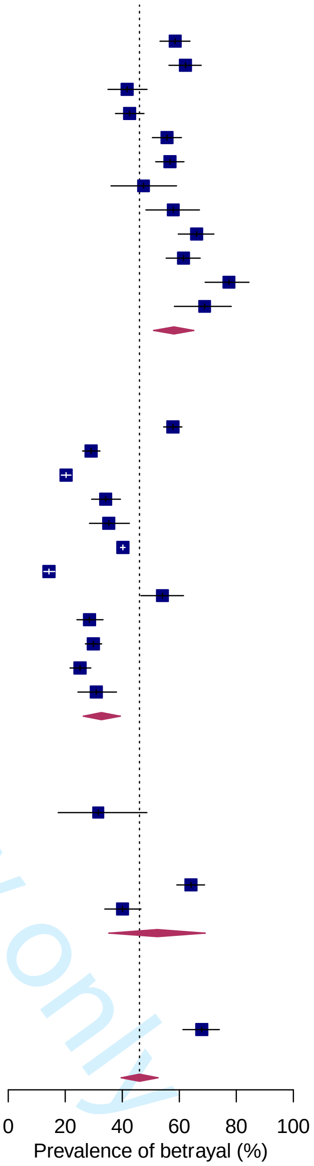

For peer review only

Supplementary Figure 9.

MISS-HP pooled prevalence estimates.

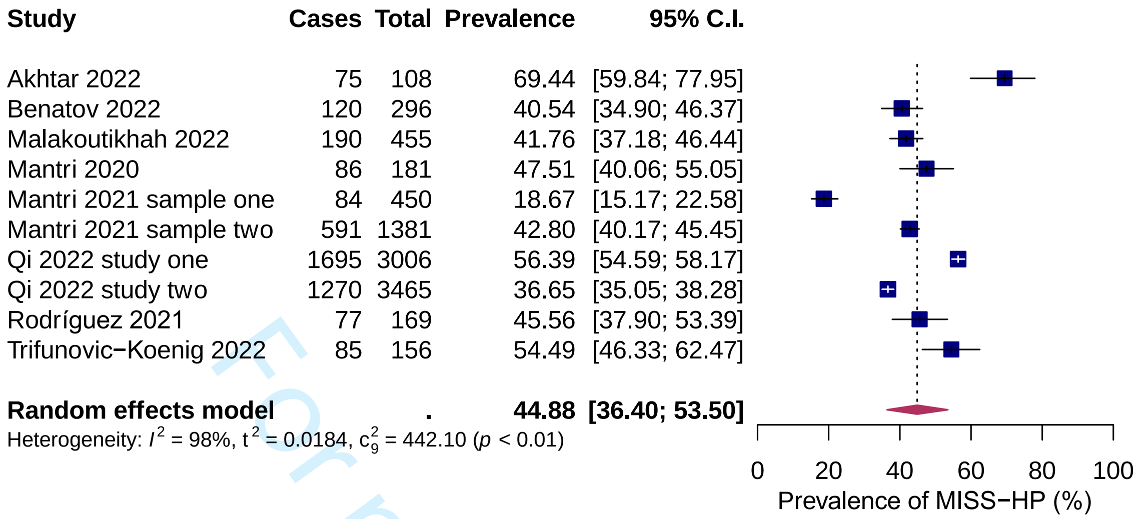

*Analyses using **pooled mean** estimates for PTED and moral injury, split by occupation*

Supplementary Figure 10.

PTED pooled mean estimates, split by occupation.

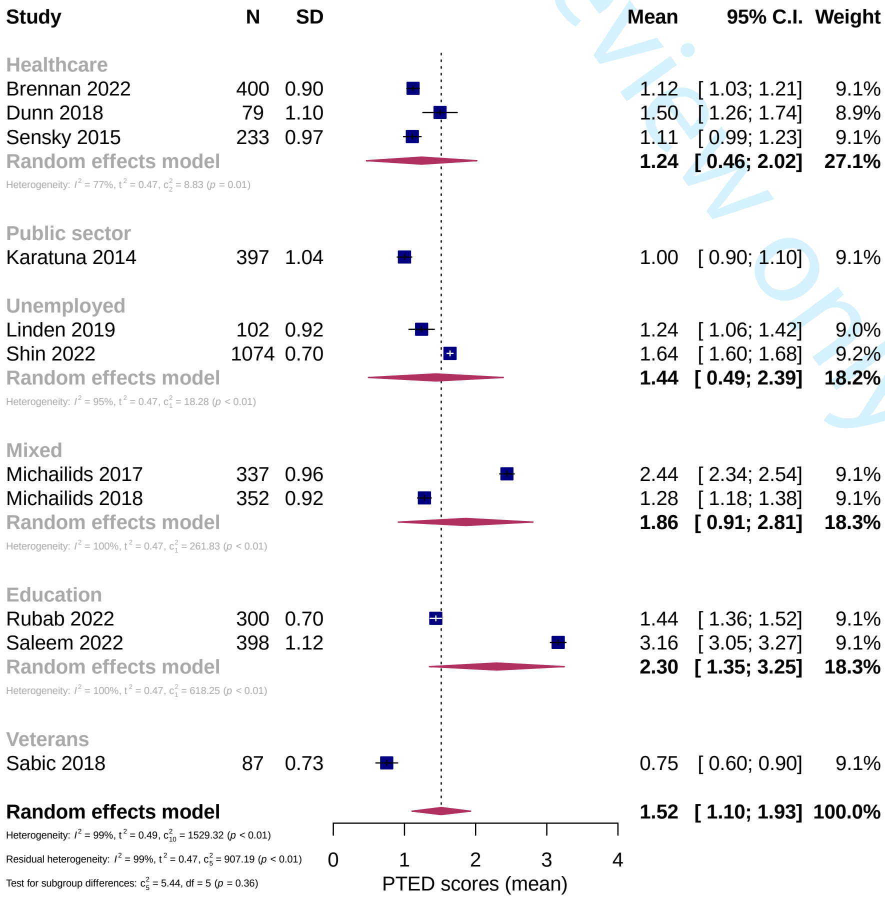

For peer review only

Supplementary Figure 11.
Overall MIES pooled mean estimates, split by occupation.

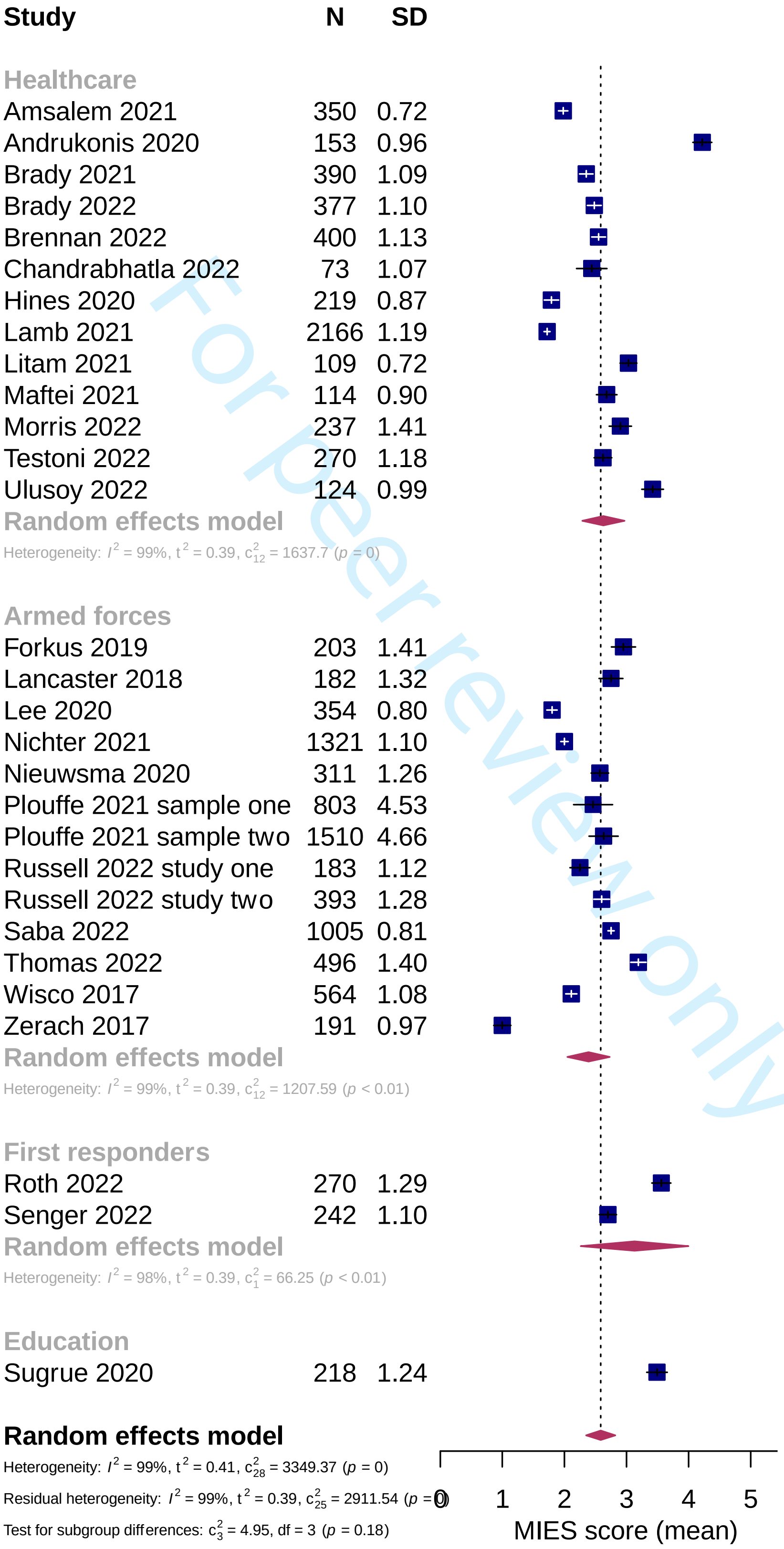

For peer review only - http://bmjopen.bmj.com/site/about/guidelines.xhtml

Supplementary Figure 12.
MIES perpetration pooled mean estimates.

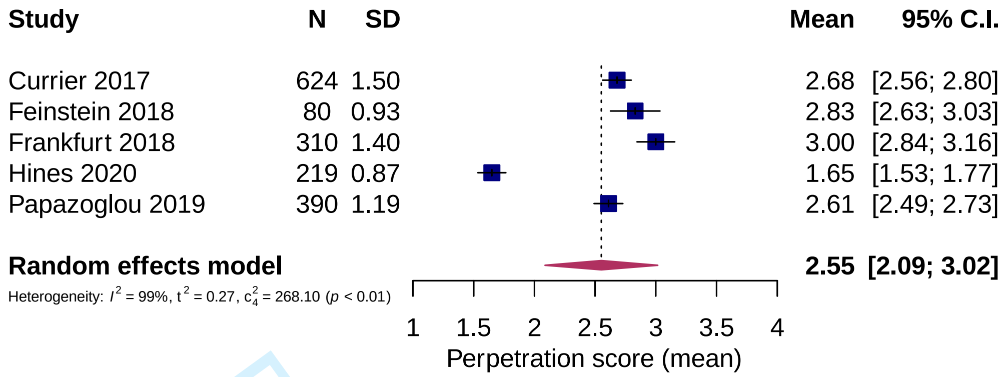

Supplementary Figure 13.
MIES transgressions-self pooled mean estimates, split by occupation.

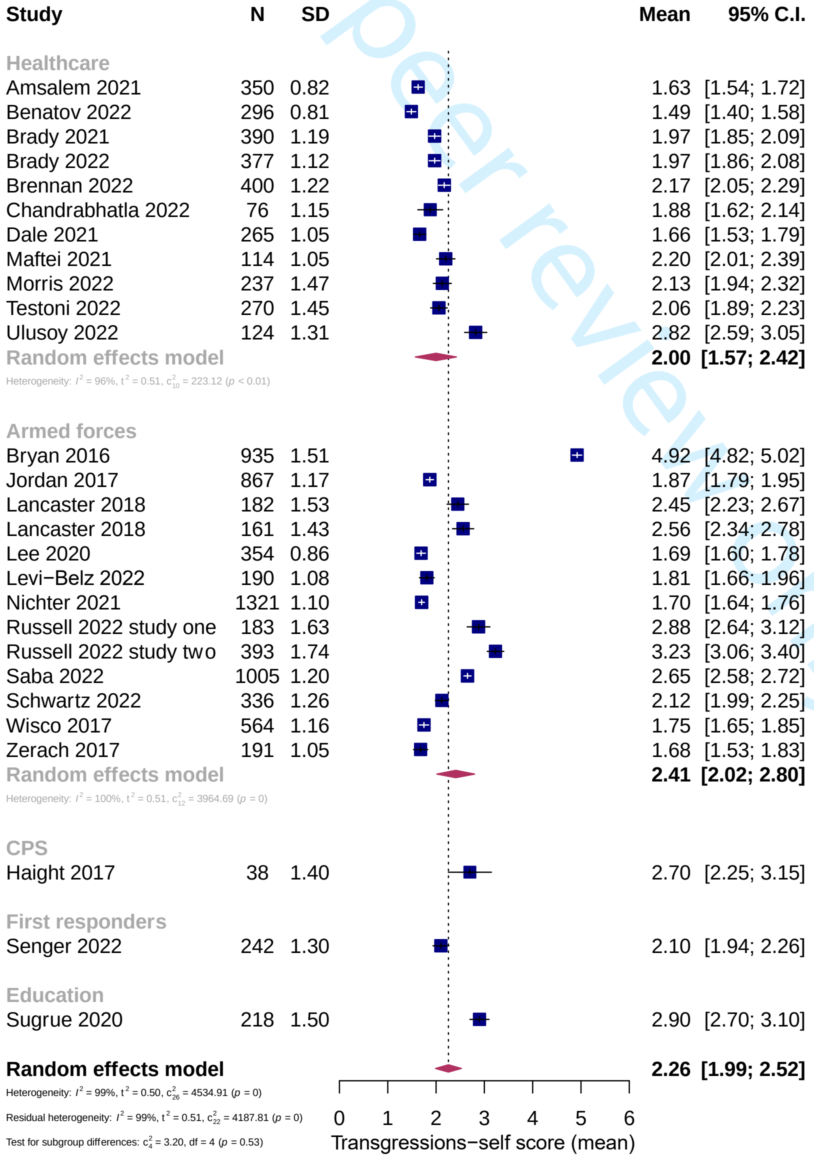

For peer review only

Supplementary Figure 14.
MIES transgressions-others pooled mean estimates, split by occupation.

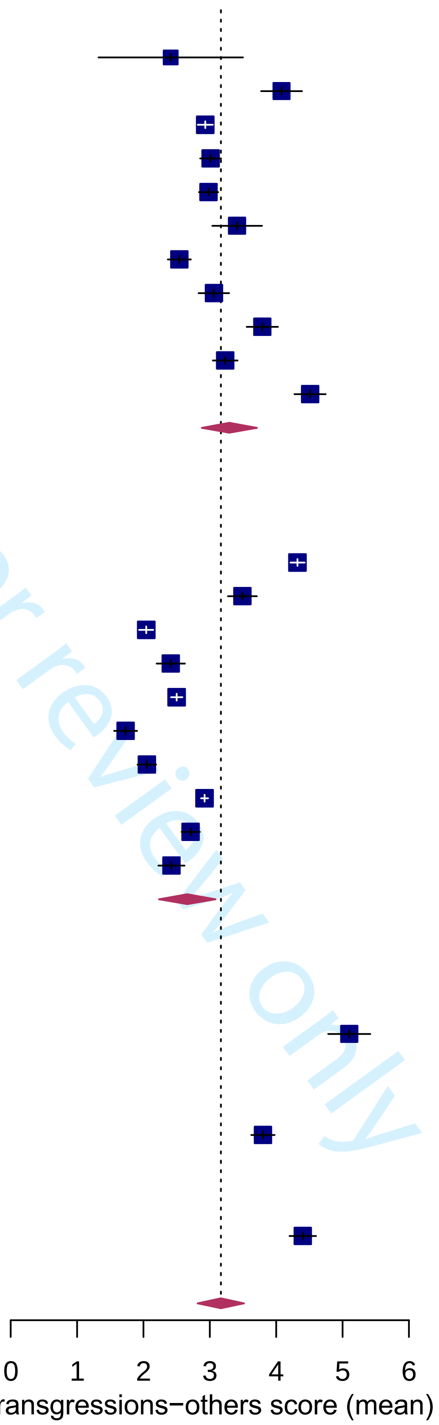

Study	N	SD	Mean	95% C.I.
**Healthcare**				
Amsalem 2021	350	10.40	2.41	[1.32; 3.50]
Benatov 2022	296	2.71	4.08	[3.77; 4.39]
Brady 2021	390	1.15	2.93	[2.82; 3.04]
Brady 2022	377	1.56	3.01	[2.85; 3.17]
Brennan 2022	400	1.48	2.98	[2.83; 3.13]
Chandrabhatla 2022	74	1.64	3.41	[3.04; 3.78]
Dale 2021	265	1.45	2.54	[2.37; 2.71]
Maftei 2021	114	1.26	3.06	[2.83; 3.29]
Morris 2022	237	1.86	3.79	[3.55; 4.03]
Testoni 2022	270	1.56	3.23	[3.04; 3.42]
Ulusoy 2022	124	1.34	4.51	[4.27; 4.75]
**Random effects model**			**3.29**	**[2.88; 3.71]**
Heterogeneity: $I^2 = 96\%$, $t^2 = 0.47$, $c^2_{10} = 264.93$ ($p < 0.01$)				
**Armed forces**				
Bryan 2016	935	1.67	4.32	[4.21; 4.43]
Lancaster 2018	182	1.50	3.49	[3.27; 3.71]
Lee 2020	354	1.04	2.04	[1.93; 2.15]
Levi−Belz 2022	190	1.49	2.41	[2.20; 2.62]
Nichter 2021	1321	1.60	2.50	[2.41; 2.59]
Russell 2022 study one	183	1.20	1.73	[1.56; 1.90]
Russell 2022 study two	393	1.42	2.05	[1.91; 2.19]
Saba 2022	1005	0.84	2.92	[2.87; 2.97]
Wisco 2017	564	1.66	2.71	[2.57; 2.85]
Zerach 2017	191	1.39	2.42	[2.22; 2.62]
**Random effects model**			**2.66**	**[2.23; 3.09]**
Heterogeneity: $I^2 = 99\%$, $t^2 = 0.47$, $c^2_{9} = 1352.72$ ($p < 0.01$)				
**CPS**				
Haight 2017	38	1.00	5.10	[4.78; 5.42]
**First responders**				
Senger 2022	242	1.40	3.80	[3.62; 3.98]
**Education**				
Sugrue 2020	218	1.50	4.40	[4.20; 4.60]
**Random effects model**			**3.16**	**[2.81; 3.52]**

Heterogeneity: $I^2 = 99\%$, $t^2 = 0.75$, $c^2_{23} = 2215.96$ ($p = 0$)

Residual heterogeneity: $I^2 = 99\%$, $t^2 = 0.47$, $c^2_{19} = 1617.66$ ($p = 0$)

Test for subgroup differences: $c^2_4 = 17.36$, df = 4 ($p < 0.01$)

For peer review only - http://bmjopen.bmj.com/site/about/guidelines.xhtml

Supplementary Figure 15.
MIES betrayal pooled mean estimates, split by occupation.

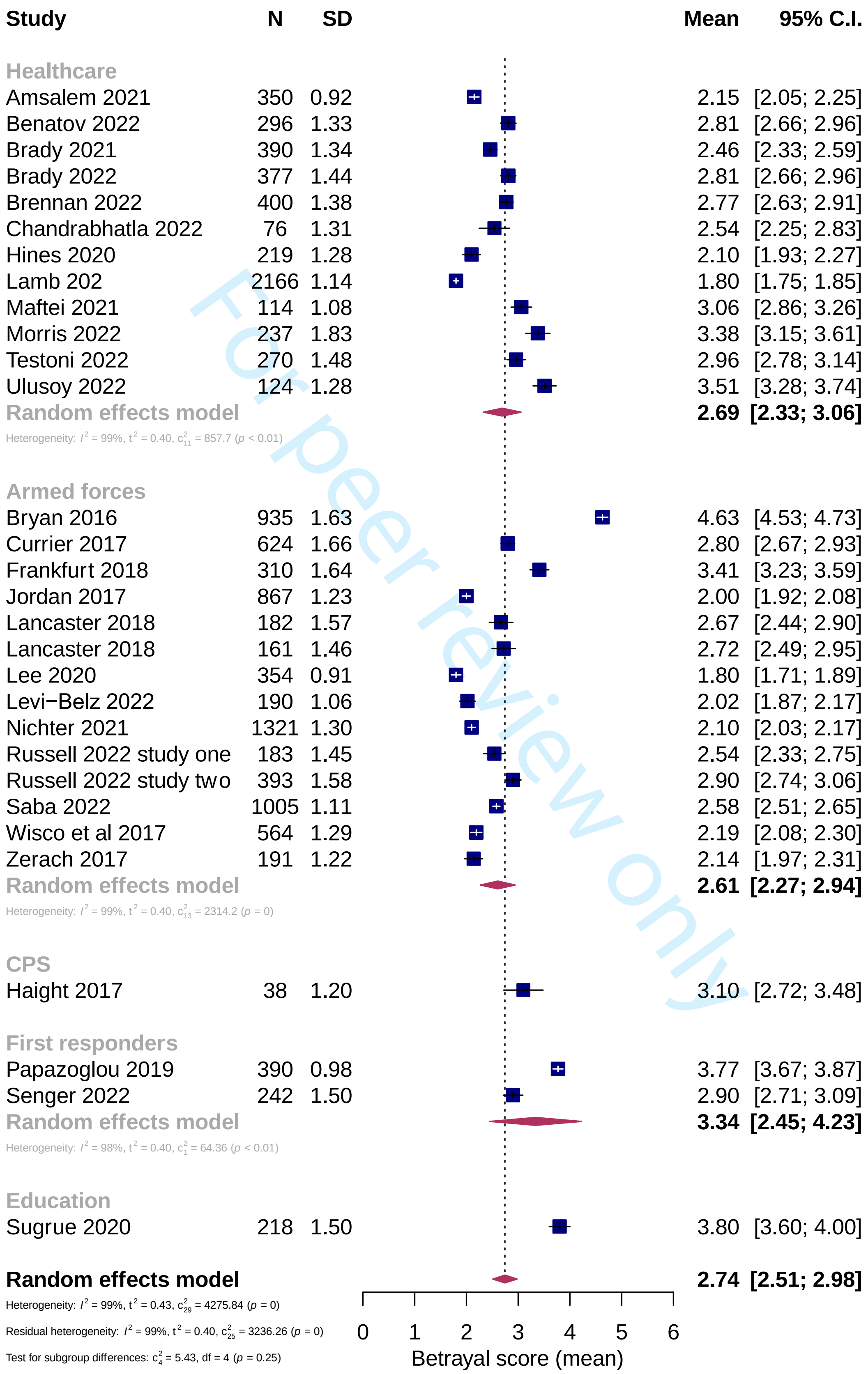

For peer review only

For peer review only - http://bmjopen.bmj.com/site/about/guidelines.xhtml

Supplementary Figure 16.
MIQ-M total pooled mean estimates.

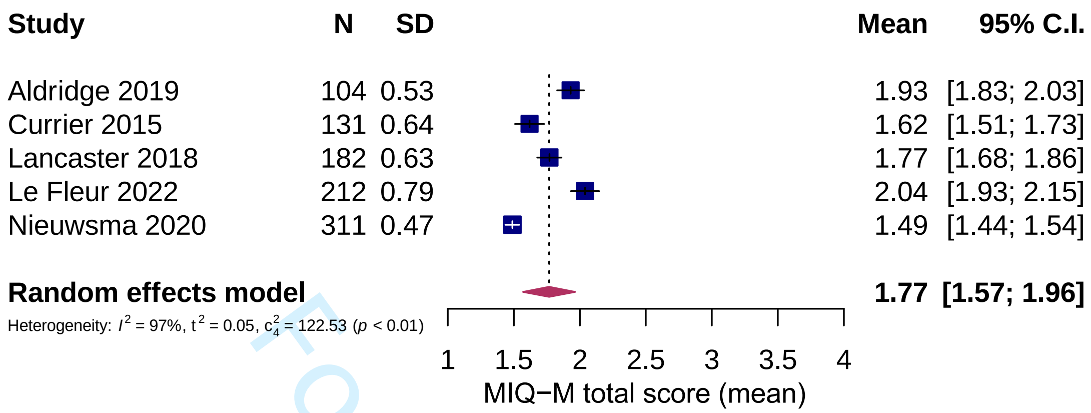

Supplementary Figure 17.
MIQ-M causes pooled mean estimates.

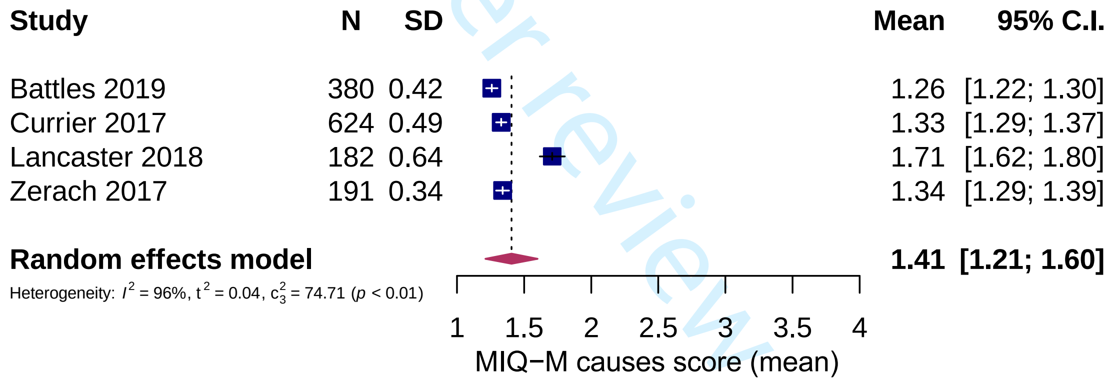

Supplementary Figure 18.
EMIS total pooled mean estimates.

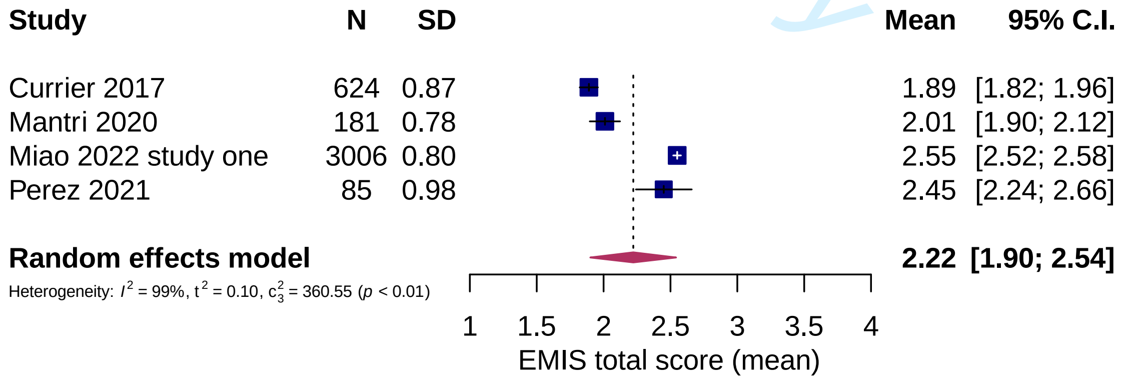

For peer review only

Supplementary Figure 19.
EMIS self pooled mean estimates.

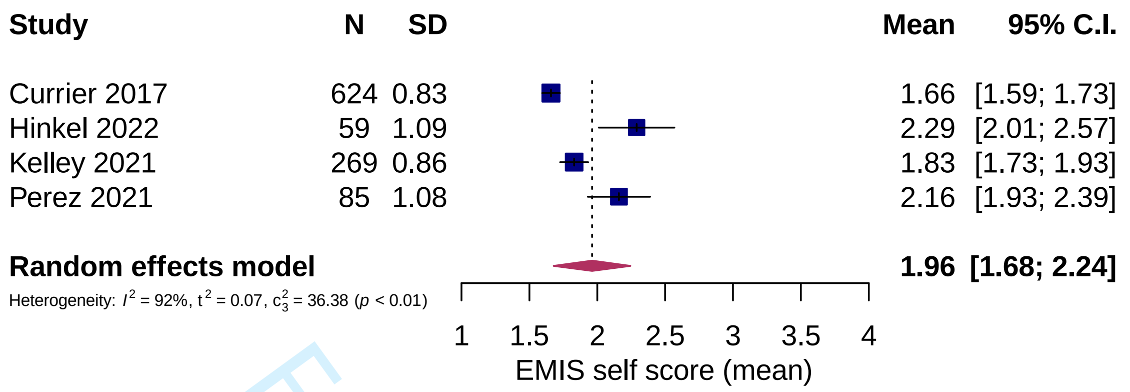

Supplementary Figure 20.
EMIS other pooled mean estimates.

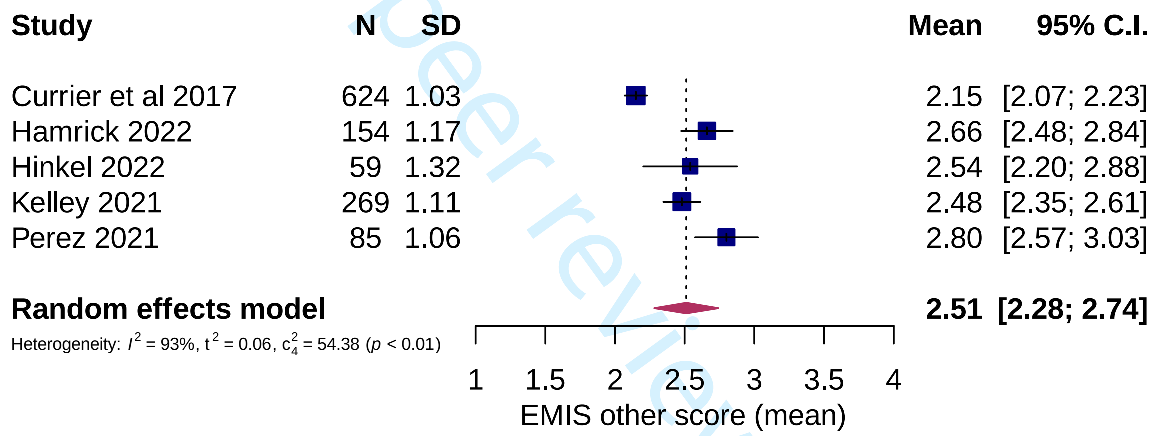

Supplementary Figure 21.
MISS-HP pooled mean estimates.

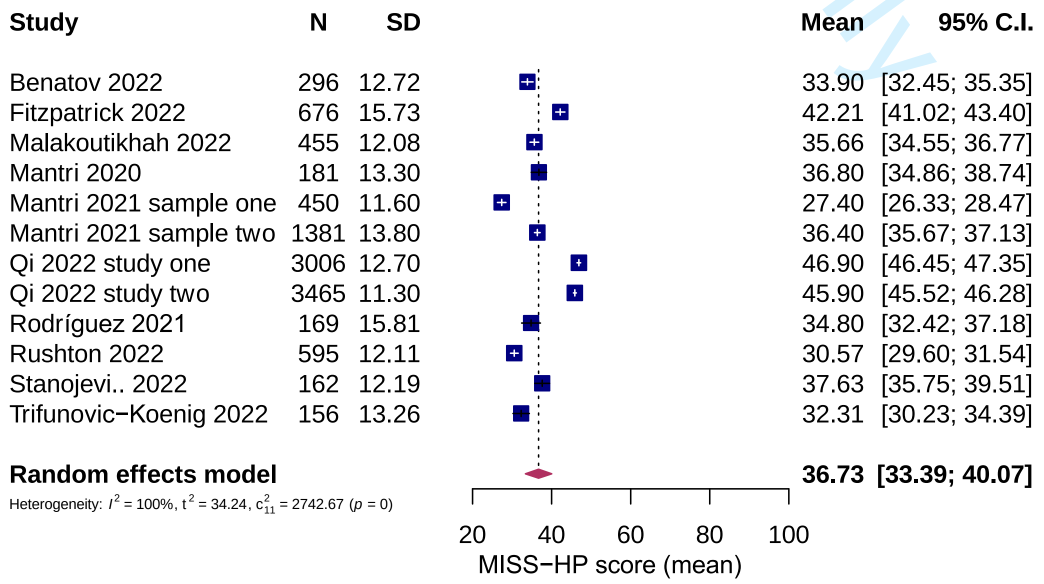

For peer review only

Supplementary Figure 22.
MIOS total pooled mean estimates.

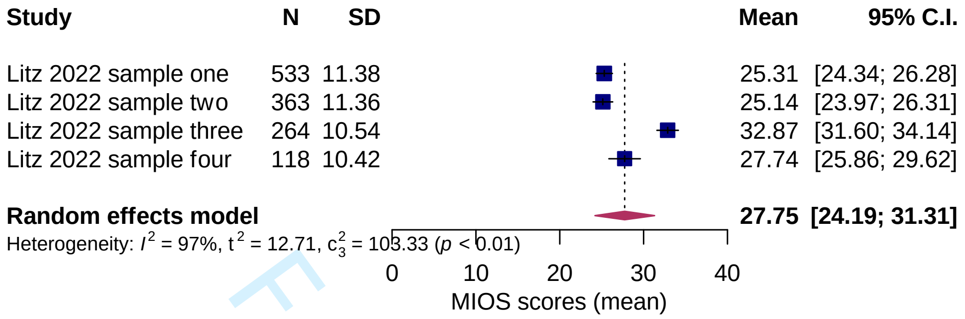

Supplementary Figure 23.
MIOS shame pooled mean estimates.

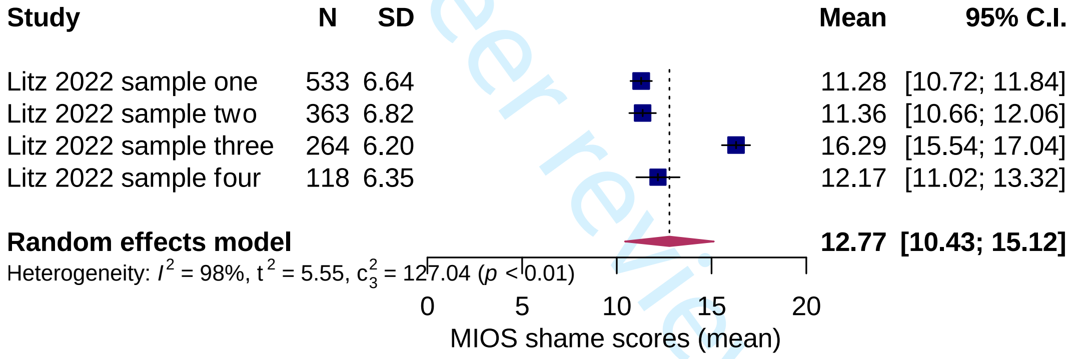

Supplementary Figure 24.
MIOS trust pooled mean estimates.

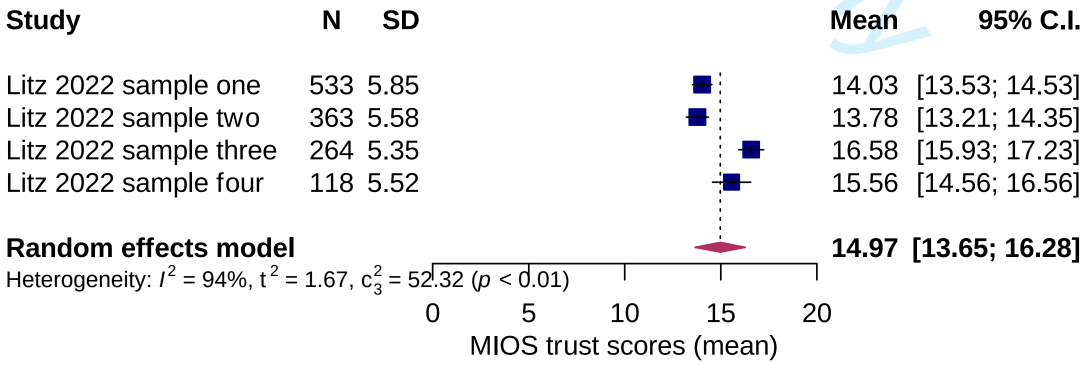

For peer review only

Supplementary Table 4.

Subgroup analyses based on study quality (mean and prevalence)

Measure	Medium risk			Low risk			
	k	Pooled mean [95% CI]	I^2	*k*	Pooled mean [95% CI]	I^2	P value
PTED	10	1.56 [1.11, 2.01]	99%	1	1.12 [1.03, 1.21]	-	0.566
MIES total	24	2.67 [2.42, 2.92]	99%	5	2.18 [1.62, 2.73]	97%	0.114
MIES transgressions-self	25	2.30 [2.02, 2.58]	99%	2	2.17 [0.75, 2.70]	0%	0.267
MIES transgressions-others	22	3.22 [2.85, 3.58]	99%	2	2.60 [1.40, 3.81]	85%	0.342
MIES betrayal	27	2.83 [2.60, 3.06]	99%	3	2.03 [1.33, 2.73]	97%	0.034
	k	Pooled prevalence (95% CI)	I^2	*k*	Pooled prevalence [95% CI]	I^2	P value
MIES	18	72.30 [64.83, 79.20]	97%	5	47.06 [32.42, 61.92]	99%	0.003
Transgressions-self	22	34.92 [28.32, 41.82]	99%	5	15.94 [84.7, 25.21]	98%	0.006
Transgressions-others	21	50.23 [39.64, 60.81]	98%	4	35.11 [14.67, 58.94]	99%	0.254
Betrayal	23	49.69 [43.09, 56.29]	97%	5	30.33 [18.51, 43.66]	99%	0.011

Note. PTED = post-traumatic embitterment disorder, MIES = Moral Injury Events Scale.

For peer review only

Supplementary Table 5.

Subgroup analyses based on cut off value (prevalence)

Measure	Slightly agree		Moderately agree		Strongly agree		Agree		NR[a]		
	k	Pooled %	*k*	Pooled %	*k*	Pooled %	*k*	Pooled %	*k*	Pooled %	P value
MIES overall	14	72.47 [63.07, 80.98]	6	54.13 [38.88, 69]		N/A	2	73.36 [47.45, 92.77]	1	49.72 [15.70, 83.88]	0.152
Transgressions -self	20	31.93 [24.52, 39.81]	3	22.06 [74.1, 41.63]	1	54.33 [20.26, 86.24]	2	28.04 [84.7, 53.41]	1	23.02 [1.68, 58.11]	0.578
Transgressions -others	18	52.22 [41.78, 62.56]	3	37.46 [15.33, 62.77]	1	38.82 [53.39, 80.92]	2	57.56 [27, 85.26]	1	02.26 [0, 31.56]	0.057
Betrayal	19	46.87 [38.98, 54.83]	6	37.54 [24.56, 51.49]	1	57.75 [24.89, 87.16]	1	58.24 [33.89, 80.65]		-	0.409

Measure	28.5		36		36.5		
	k	Pooled %	*k*	Pooled %	*k*	Pooled %	P value
MISS-HP	1	54.49 [46.61, 62.25]	8	44.17 [33.84, 54.77]	1	41.76 [37.26, 46.33]	0.802

Note. [a] For MIES any this was "sometimes". MIES = Moral Injury Events Scale; MISS-HP = Moral Injury Symptom Scale-Healthcare Professionals

For peer review only - http://bmjopen.bmj.com/site/about/guidelines.xhtml

For peer review only

Supplementary Table 6.
Prevalence and average scores for moral injury scales that were not combined for narrative synthesis (k=1)

Measure	Mean(SD)	Measure	Prevalence (%)
MIES-other (items 1-2, 7-9)	2.51 (1.40)	MIES-other (items 1-2, 7-9)	19.76%
MIES-commission (items 1-4)	1.71 (1.06)	MIES-commission (items 1-4)	14.28%
MIES-omission (items 5-6)	1.47 (1.1)	MIES-omission (items 5-6)	4.57%
MIQ-T	1.16 (0.56)	MIES-no moral distress	42%
MIQ-M modified		MIES-moral distress-other	19%
MIQ-M modified AoW	1.77 (0.63)	MIES-witnessing-only	16%
MIQ-M modified PCoW	1.99 (0.66)	MIES-moral distress-self	8%
MIQ-M modified LFB	2.12 (0.78)	MIES-moral distress-self and other	15%
MIQ-M modified AoW defining characteristics	2.21 (0.67)	EMIS-M self-directed ≥2.5 score <3.5	10.14%
MIQ-M modified PCoW defining characteristics	2.14 (0.66)	EMIS-M other-directed ≥2.5 score <3.5	29.19%
MIQ-M modified LFB defining characteristics	2.12 (0.67)		
BMIS total	3.71 (4.38)	BMIS event	44.1%
MIA-PSP total	59.6 (21.9)		
MIA-PSP emotional sequalae	25 (9.7)		
MIA-PSP perpetration	15 (8.5)		
MIA-PSP betrayal	19.6 (7.7)		
Toronto total	1.5 (1.27)		
Toronto organisation	1.54 (1.32)		
Toronto individual	1.18 (1.19)		
Toronto online	1.68 (1.27)		

Note. MI = moral injury; MIES = Moral Injury Events Scale; EMIS-M = Expressions of Moral Injury Scale-Military Version; MIA-PSP = Moral Injury Assessment for Public Safety Personnel; MIQ-M = Moral Injury Questionnaire—Military version; BMIS = Brief Moral Injury Screen; MIQ-T = Moral Injury Questionnaire—Teacher version; AoW = atrocities of war; LFB = leadership failure or betrayal; PCoW = psychological consequences of war

For peer review only

For peer review only

For peer review only - http://bmjopen.bmj.com/site/about/guidelines.xhtml

BMJ Open

A systematic review and meta-analysis on the prevalence of occupational moral injury and post-traumatic embitterment disorder

Journal:	*BMJ Open*
Manuscript ID	bmjopen-2023-071776.R2
Article Type:	Original research
Date Submitted by the Author:	20-Jan-2024
Complete List of Authors:	Brennan, Chloe; University of Liverpool, Psychology<br>Roberts, Carl; Institute of Psychology, Health and Society, University of Liverpool, UK, Department of Psychological Sciences,<br>Cole, Jon; University of Liverpool
**Primary Subject Heading**:	Epidemiology
Secondary Subject Heading:	Mental health, Health policy, Global health, Epidemiology
Keywords:	OCCUPATIONAL & INDUSTRIAL MEDICINE, Systematic Review, EPIDEMIOLOGY, MENTAL HEALTH, PSYCHIATRY

SCHOLARONE™
Manuscripts

I, the Submitting Author has the right to grant and does grant on behalf of all authors of the Work (as defined in the below author licence), an exclusive licence and/or a non-exclusive licence for contributions from authors who are: i) UK Crown employees; ii) where BMJ has agreed a CC-BY licence shall apply, and/or iii) in accordance with the terms applicable for US Federal Government officers or employees acting as part of their official duties; on a worldwide, perpetual, irrevocable, royalty-free basis to BMJ Publishing Group Ltd ("BMJ") its licensees and where the relevant Journal is co-owned by BMJ to the co-owners of the Journal, to publish the Work in this journal and any other BMJ products and to exploit all rights, as set out in our licence.

The Submitting Author accepts and understands that any supply made under these terms is made by BMJ to the Submitting Author unless you are acting as an employee on behalf of your employer or a postgraduate student of an affiliated institution which is paying any applicable article publishing charge ("APC") for Open Access articles. Where the Submitting Author wishes to make the Work available on an Open Access basis (and intends to pay the relevant APC), the terms of reuse of such Open Access shall be governed by a Creative Commons licence – details of these licences and which Creative Commons licence will apply to this Work are set out in our licence referred to above.

Other than as permitted in any relevant BMJ Author's Self Archiving Policies, I confirm this Work has not been accepted for publication elsewhere, is not being considered for publication elsewhere and does not duplicate material already published. I confirm all authors consent to publication of this Work and authorise the granting of this licence.

For peer review only

A systematic review and meta-analysis on the prevalence of occupational moral injury and post-traumatic embitterment disorder

Chloe J. Brennan[1], Carl A. Roberts[1], Jon C. Cole[1]

[1] Department of Psychology, University of Liverpool, United Kingdom

For peer review only

Keywords: Mental health, Psychiatry, Occupational & Industrial Medicine, Systematic review, Epidemiology

Word count: 5995

[1] **Correspondence to**
Chloe Brennan
Eleanor Rathbone Building
Bedford Street South
Liverpool
L69 7ZT
Telephone: +44 (0)151 794 2000
cbrennan@liverpool.ac.uk

For peer review only - http://bmjopen.bmj.com/site/about/guidelines.xhtml

Abstract:

Objectives Occupational moral injury and post-traumatic embitterment disorder (PTED), describe the psychological distress caused by exposure to injustice at work. This meta-analysis aims to determine the prevalence of occupational moral injury and PTED and establish whether prevalence estimates differ depending on occupation.

Design A systematic review and meta-analysis.

Data sources Google Scholar, PubMed, APA PsycINFO, Web of Science Core Collection, Scopus, ScienceDirect and Sage Journals Online were searched in June 2020 and updated in November 2022.

Eligibility criteria for selecting studies Observational studies that measured prevalence or average scores of moral injury, or PTED in any occupational group and any geographical location.

Data extraction and synthesis Two independent reviewers screened and coded eligible studies. Study design, participant demographics, sampling method, location, measurement tool and prevalence or average scores were extracted. Risk of bias was assessed using the Quality Assessment Checklist for Prevalence Studies tool. Meta-analysis was conducted using random effects models. Results that could not be combined were summarised qualitatively in a narrative synthesis using the Guidance for Systematic Reviews.

Results In total, 88 studies across armed forces and veterans, healthcare, first responders, educators, journalists, child protection service employees, the unemployed, public-sector employees and mixed occupations were included. Studies included in each separate meta-analysis based on the measure used ranged from two to 30. The pooled prevalence of clinically relevant moral injury in healthcare professionals was 45%, and exposure to any potentially morally injurious event (PMIE) across occupations was 67%. Exposure to

For peer review only - http://bmjopen.bmj.com/site/about/guidelines.xhtml

transgressions by others and betrayal were significantly lower in the armed forces than civilian occupations. Pooled prevalence of PTED across occupations was 26%.

Conclusion Exposure to PMIEs, moral injury symptoms, and PTED are prevalent at work and exposure to transgressions by others and betrayal are more likely in civilian occupations than the armed forces.

PROSPERO registration number CRD42020191766.

Strengths and limitations:

- Guidelines from the Preferred Reporting Items for Systematic Reviews and Meta-Analyses Protocols (PRISMA) were used to prepare this review.
- The Joanna Briggs Institute (JBI) methodology for prevalence and incidence studies was used to develop the eligibility criteria, a search strategy, data extraction and risk of bias assessment.
- A comprehensive computer literature search of seven databases (Google Scholar, PubMed, APA PsycINFO, Web of Science Core Collection, Scopus, ScienceDirect and Sage Journals Online) was run in June 2020 and updated in November 2022.
- There was high heterogeneity amongst studies, even after employing subgroup analyses so findings must be interpreted with caution.
- Only peer-reviewed articles written in English were included which may lead to publication bias.

Introduction:

Exposure to traumatic events at work is commonplace, and meta-analyses have identified certain occupational groups that do not have special training to deal with traumatic events,[1] are at a higher risk of developing work-related post-traumatic stress disorder (PTSD).[1, 2] Individuals may also experience morally traumatic events at work, through

For peer review only

interpersonal conflicts, making challenging ethical decisions, and exposure to events that contradict what the person perceives to be ‘right’ based on their perception, values, beliefs and morals.[3-5] Moral injury and post-traumatic embitterment disorder (PTED) are two concepts that have emerged to explain the harm caused by exposure to unjust and potentially morally injurious events (PMIEs). Neither moral injury, nor PTED are categorised in current diagnostic manuals, such as the Diagnostic and Statistical Manual of Mental Disorders, version 5 (DSM-5) or International Classification of Diseases, version 11 (ICD-11), but they can have a disabling impact on individuals, such as social isolation, difficulty with relationships and difficulty maintaining employment. Organisations may experience higher rates of sickness absence and ill health retirement in employees and embitterment can lead to protracted litigation against employers [6, 7].

Moral injury refers to the psychological distress caused by exposure to PMIEs that involve perpetrating, witnessing, or learning about acts that violate moral beliefs[4] or experiences of betrayal[5]. Traditionally, moral injury has been studied in the armed forces after witnessing the horrors of war, but in 2018 a meta-analysis showed that occupational moral injury is present in civilian occupations such as journalists, veterinarians, the police, and teachers[8]. Moral injury shares similarities in aetiology and symptomology with PTED[9, 10], which is a reactive disorder caused by exposure to an unjust event and is characterised by feelings of embitterment, helplessness, and hostility[3]. Research has shown that PTED often occurs at work, for instance, 73% of PTED patients reported the triggering event as work-related[11]. PTED is evident in occupations such as the armed forces[12, 13] , educators[14, 15], healthcare professionals[9, 16, 17], the unemployed[18, 19], and in a mix of occupations[20, 21].

Despite this, there is no clear evidence for how widespread occupational moral injury and PTED are. As far as we are aware, no meta-analyses exist which combine prevalence

For peer review only

estimates of PTED, and current attempts to combine the prevalence of morally transgressive acts[22] and moral injury symptoms[23] rely on a narrative synthesis of military studies only. Since certain occupational groups that do not have special training to deal with traumatic events are at a higher risk of developing work-related PTSD[1], it is possible that certain occupations have a higher risk of developing work-related moral injury and PTED following ethically challenging events. To accurately estimate the prevalence of occupational moral injury and PTED, and investigate whether rates differ depending on occupation, systematic methods are needed.

It is challenging to estimate the pooled prevalence of moral injury and PTED as there is no gold standard measurement approach. A review by Koenig, Pearce, and Youssef[24] identified 17 scales that measure one or two aspects of moral injury, and 5 comprehensive measures of either exposure to PMIEs, moral injury symptoms, or both. Researchers report prevalence using inconsistent cut off values, and some only report average scores. Similarly, when reporting prevalence of PTED, researchers can use the PTED diagnostic interview[25], or the PTED self-rating scale, though three different cut off values are used to indicate clinically relevant embitterment at three different levels of severity.[3] Despite variability between studies, generating pooled prevalence estimates in different occupations is valuable because it can inform preventive strategies and policy decisions.

This current systematic review and meta-analyses aims to; (i) determine the prevalence of occupational moral injury and PTED in military and civilian professions, (ii) establish whether prevalence estimates differ depending on occupation, (iii) establish whether methodological differences between studies influence prevalence estimates, and (iv) record the variables associated with occupational moral injury and PTED.

Methods:

For peer review only - http://bmjopen.bmj.com/site/about/guidelines.xhtml

Guidelines from the Preferred Reporting Items for Systematic Reviews and Meta-Analyses Protocols (PRISMA)[26] were used to prepare this review. The Joanna Briggs Institute (JBI) methodology for prevalence and incidence studies[27] was used to develop the eligibility criteria (Table 1), a search strategy (Supplementary Table 1), data extraction (Supplementary Table 2) and risk of bias assessment (Supplementary Table 3). This review was registered with the international prospective register of systematic reviews PROSPERO network (registration no. CRD42020191766)[28].

Search Strategy and Study Selection:

A computer literature search was run in June 2020 using seven databases, Google Scholar, PubMed, APA PsycINFO through the EBSCOhost interface, Web of Science Core Collection, Scopus, ScienceDirect and Sage Journals online. Articles related to PTED were included from 2003, which was when the first conceptual definition for PTED was developed[29], and articles related to moral injury were included from 2009 in line with the first comprehensive scientific definition of moral injury.[4] Search terms were used as free text terms and combined with Boolean operators so the same keywords could be replicated in each database. The search was restricted to peer-reviewed articles written in English. References were managed in EndNote (Clarivate Analytics, Philadelphia) and Excel. The searches were updated in November 2022. Details of the search are listed in Supplementary Table 1. One researcher (CB) screened all titles, abstracts, and full texts and a second reviewer (CR) screened 15% of the titles and abstracts and 100% of the full texts against the eligibility criteria. Any discrepancies over the inclusion of studies were resolved through discussion. At every stage of screening, inter-rater agreement was >90%, with Cohen's kappa >0.90, indicating almost perfect agreement.[30]

The Condition, Context, Population (CoCoPop) mnemonic[25] was used to determine inclusion and exclusion criteria and are outlined in Table 1. Studies were included if they

For peer review only

measured the prevalence or average scores of PTED or moral injury (including PMIEs) using the PTED diagnostic interview,[25] PTED self-rating scale,[3] or a comprehensive and standardised measure of moral injury. Studies included in this review measured moral injury as a whole construct rather than measuring the separate theoretical elements, such as guilt and self-forgiveness (i.e., see [24] for a review). For inclusion, moral injury and PTED must have been measured in an occupational group, in relation to work, or in unemployed or retired samples. Any working age, sex, ethnicity, country, educational status, and socio-demographic status was included. Secondary outcomes of interest were univariate associates of moral injury or PTED. The review included observational studies (i.e., cross-sectional, retrospective, prospective, cohort and longitudinal) and excluded intervention studies or reviews. Only peer-reviewed articles written in English were eligible. When multiple papers were identified using the same data, the most relevant paper was included.

Data extraction

The following data was extracted; author and year published, study design, number of participants, mean age, the proportion of males, proportion of white ethnicity, socio-economic status, geographical location, occupation, length of time in the job, sampling method including setting and dates, the measurement tool for moral injury or PTED, prevalence (N and proportion), average scores (Mean and SD), and any conflict of interest. Secondary outcomes included univariate associates of moral injury and PTED including the variable name, the measure used, and the direction of effect (positive, negative or no association). For longitudinal and cohort studies, prevalence estimates or average scores from the baseline wave were extracted unless only subsequent waves were reported. CB contacted the authors to obtain relevant information that was not available in the text. One researcher (CB) completed the data extraction, and a second reviewer (CR) screened 100% of the data extraction to check for accuracy.

For peer review only - http://bmjopen.bmj.com/site/about/guidelines.xhtml

Table 1.
Inclusion and exclusion criteria

Inclusion criteria	
Condition (outcome of interest)	• Studies measuring the prevalence (%) or average scores (M and SD) of PTED, including chronic embitterment.<br>• Studies measuring prevalence (%) or average scores (M and SD) of moral injury.<br>• Uses PTED diagnostic interview, PTED self-rating scale, or a comprehensive moral injury measure.
Context	• All geographical locations.
Population	• PTED or moral injury must have been measured in relation to occupation including studies reporting on multiple occupations, the unemployed or retired.<br>• 18+, any sex, ethnicity, educational status, or socio-economic status.
Exclusion criteria	
Condition	• Studies using the Bern Embitterment Inventory (BEI) to measure PTED.<br>• Studies which only measured theoretical subcomponents or limited dimensional scales of moral injury.
Context	• Clinical patients in treatment or intervention studies.
Population	• Student only samples.<br>• Entirely clinical samples or all exposed to a specific stressor i.e., bullied at work, all exposed to a moral transgression, all injured at work etc.

PTED; post-traumatic embitterment disorder

For peer review only

Quality assessment

Quality assessment was conducted using the Quality Assessment Checklist for Prevalence Studies created by Hoy et al.,[31]. This tool assesses the possibility of bias in design, conduct and analysis including sample representativeness, sampling, non-response bias, data collection method, appropriateness of case definition, study instrument, and statistical analysis. Each item is coded as 0 (yes, low risk), or 1 (no, high risk) and quality assessment scores were summed, and categorised into low (0-3), moderate (4-6) and high (7-9) risk. Two researchers (CB, CR) conducted the quality assessment, and any discrepancies were resolved through discussion (see Supplementary Table 3).

Data synthesis and analysis

We conducted the meta-analyses using R (4.2.1) with the Meta package.[32] All meta-analyses used a random effects model using the restricted maximum-likelihood estimator (REML) due to the high heterogeneity associated with observational studies.[33]

For peer review only - http://bmjopen.bmj.com/site/about/guidelines.xhtml

Separate meta-analyses were conducted with the prevalence of PTED as the outcome, and then the prevalence of moral injury as the outcome. For PTED prevalence, three separate analyses were conducted based on a cut-off $\geq$1.6, then 2 and then 2.5 which represents different levels of clinical severity.[3] For moral injury, due to the heterogeneity in moral injury measures (see Table 2 for an overview), a separate meta-analysis was conducted for each moral injury measure and their associated subscales. Some moral injury scales do not use cut-off values to estimate the prevalence and only report average scores, so additional meta-analyses were conducted with the mean of PTED and mean of moral injury scores as the outcome.

Subgroup analyses were conducted if there were sufficient studies of k $\geq$10[34], grouping studies based on occupation. Forest plots were generated, displaying the prevalence and 95% CIs for each estimate, the overall pooled estimate, and 95% CIs for each occupational group. Additional subgroup analyses were conducted based on study quality (low or medium risk) and on cut-off value used for moral injury prevalence meta-analyses. If a study reported the prevalence of moral injury using multiple cut-off scores in the same paper, then the most common across studies was chosen for inclusion. A random effect model with a common estimate for $\tau 2$ was used in subgroup analyses due to k$\leq$5 in some of the subgroups.[35] In order to determine whether any of the subgroups were statistically different, a Q-test with $p<0.05$ was used based on the overall subgroup results. No overlap in 95% CIs was used to determine which groups were significantly different from one another.[36]

For meta-analyses that used prevalence estimates as the outcome, the Freeman-Tukey double arcsine transformation method was applied, which is effective when there are small sample sizes and extreme prevalence estimates.[37, 38] Heterogeneity was assessed using $I2$ with a cut-off of >50% suggesting high heterogeneity.[39] Significance was assessed using

For peer review only - http://bmjopen.bmj.com/site/about/guidelines.xhtml

$x2$ for Cochrane's Q (p<0.05). Funnel plots are not suitable to assess publication bias in meta-analyses of proportions.[40] Therefore, a sensitivity analysis was used, comparing prevalence and average scores when outliers were removed, which were extremely small or large values that fell outside of the CI of the pooled effect.[41]

Finally, where prevalence or average scores could not be pooled due to inconsistent reporting or insufficient studies (k=1), narrative synthesis was used to describe estimates. The narrative synthesis also described the univariate associates of PTED and moral injury using the Guidance for Systematic Reviews.[42] Associates were grouped into demographic, mental health and individual/work variables, and the percentage of studies that reported each type of variable was calculated. Vote counting was used to determine whether the overall relationship between variables with PTED and moral injury was positive, negative or no association.

Patient and public involvement

We conducted no primary research involving patients because the systematic review focused on published literature.

Results:

Study characteristics:

The PRISMA flow chart delineates the review process (see Figure 1). Of the 81 full texts included in the review[12-21, 43-114], some reported multiple studies in the same paper, so there were 88 separate studies included in the review. Of these, six reported prevalence estimates or average scores that could not be pooled, so they were included in the narrative synthesis only. Of the 88 studies included, 10 measured PTED, one measured both PTED and moral injury, and 77 measured moral injury. The number of studies included in each separate meta-analysis based on the measure used ranged from two to 30.

For peer review only - http://bmjopen.bmj.com/site/about/guidelines.xhtml

The characteristics of included studies are in Supplementary Table 2 and the risk of bias information is in Supplementary Table 3. Of the 88 studies, 10 had a low risk of bias (11%) and all others were medium risk, 77 (87.5%) were cross-sectional and all others were longitudinal, and sample sizes ranged from 38 to 14,057. Despite a small number of PTED studies, there was a varied geographical distribution, with three studies being conducted in the United Kingdom (UK) and one study each conducted in Germany, Turkey, Bosnia and Herzegovina, Cyprus, Pakistan, and South Korea. Occupations also varied with samples including healthcare (n = 4), educators (n = 2), mixed occupations (n = 2), the unemployed (n = 2), veterans (n = 1) and the public sector (n = 1). In contrast, most moral injury studies were conducted in the United States (US) (n = 41), and in military samples (n = 39). Health and social care workers (HSCWs) were the second biggest occupational group (n = 32), and other occupations were less represented including first responders (n = 2), educators (n = 2), journalists (n = 2), and Child Protection Services (CPS) employees (n = 1). Geographically, aside from the US, studies were conducted in the UK (n = 6), Canada (n = 5), Israel (n = 4), Australia (n = 3), Republic of Ireland (n = 2), China (n = 2), and other countries with only one study included El Salvador, Korea, Finland, Pakistan, Romania, Iran, Honduras, Italy, Croatia, German-speaking populations, and Turkey.

Table 2 shows the characteristics of the PTED, and moral injury measurement scales included in the review. All studies measuring PTED used the PTED-self rating scale, in contrast to studies measuring moral injury which included 11 distinct scales, five of which conflated PMIEs with symptoms in the same items, two scales assessed both PMIEs and symptoms but separately, and four assessed moral injury symptoms only. The most common moral injury scale was the Moral Injury Events Scale (MIES)[115] (n = 38) which conflates events and symptoms, followed by the moral injury symptom scale for healthcare professionals (MISS-HP)[94] which assesses clinically relevant symptoms of moral injury in

For peer review only - http://bmjopen.bmj.com/site/about/guidelines.xhtml

healthcare professionals (n = 12). Most moral injury scales are occupation-specific, and there was variation in reporting regarding the number of items, scoring, the cut-off value used, and subscale factor structure endorsed across the moral injury scales.

For peer review only

Table 2
PTED and moral injury measurement scales included in the systematic review

Measure	*k*	Events or symptoms	Decision regarding classification	Subscales	Cut off value	Scale information
PTED self-rating scale[3]	11	Symptoms linked to event	Assess embitterment reactions to event(s) specified in instructions	Unidimensional	≥1.6, ≥2, ≥2.5	19 items (0-4)
MIES[115]	38	Combined	Assesses exposure to PMIEs and effects (i.e., feeling troubled by those acts).	Bryan et al.,[44] 3 factors: transgressions-others, transgressions-self, and betrayal. Nash et al.,[115] 2 factors: perpetration and betrayal. Richardson et al.,[58] 2 factors: MI-self and MI-other. Lamb et al., 2021[88] 3 factors: commission, omission, and betrayal.	4+ (slightly agree) 5+ (moderately agree) 6+ (strongly agree)b	9 items (1-6 Likert)
MIQ-M[45]	9	Combined	Assesses military specific PMIEs, and effects.	Primarily unidimensional.<br><br>Lancaster and Harris[66] 2 factors: MIQ-causes and MIQ-effect	None	19 items (1-4 Likert)
MIQ-T[46]	1	Combined	Assesses teacher specific and effects	Unidimensional	None	12 items (1-4 Likert)
MIQ-M modified[65]	1	Events and symptoms	Modified PMIE items from the original MIQ-M which conflated PMIEs and effects into PMIEs and defining characteristics	3 PMIEs subscales AoW, PCoW and LFB and 3 subscales for their defining characteristics	2+ (seldom) on each item	22 PMIEs items and 5 defining characteristics items per PMIE (1-4 Likert)
EMIS-M[47]	7	Symptoms	Assesses guilt, shame, moral concerns, self-condemnation, social withdrawal, and inability to forgive self, anger, feelings of betrayal, revenge, and disgust.	2 subscales: self-directed and other-directed	None	17 items (1-5 Likert)
EMIS-SF[114]	1	Symptoms	Assesses emotions (guilt and shame) and beliefs/attitudes (moral disgust and mistrust).	Unidimensional	None	4 items (1-5 Likert)

For peer review only - http://bmjopen.bmj.com/site/about/guidelines.xhtml

MISS-HP[94]	12	Symptoms	Assesses betrayal, guilt, shame, moral concerns, loss of trust, loss of meaning, difficulty forgiving, self-condemnation, religious struggle, and loss of religious/spiritual faith.	Unidimensional	>36a	10 items (1-10 visual analogue Likert)
MIOS[90]	4	Symptoms linked to PMIE	The symptom scale refers to the most distressing PMIE. Symptoms include self-blame, loss of faith, trust, worthiness, goodness in others, honesty, pride, self-hatred, disgust and anger.	2 subscales: Shame-related (SR) and trust violation-related (TVR)	Nonec	14 items (0-4)
BMIS[98]	1	Events and symptoms	Endorsement of at least one PMIE assessed needed to complete symptom questions	2 subscales: BMIS-event (BMIS-E) and BMIS-sequalae (BMIS-S)	2+ PMIE Symptom subscale: None	7 items (0-3).
MIA-PSP[101]	1	Combined	Assesses feeling “bothered” by PSP specific PMIEs, and symptoms (i.e., feelings of sadness, anger, self-hatred, guilt, shame, disgust, and fear).	3 subscales: perpetration, betrayal and emotional Sequalae	None	17 items (1-6)
Toronto scale for Journalists[99]	1	Combined	Assesses journalism specific PMIEs and effects (i.e., feeling troubled by those PMIEs).	3 subscales: organisational/management; individuals/Non-management, and online	None	9 items (0-4)

Note: The number of items and scoring is based on the typical use of the scale. aThe German MISS-HP (G-MISS-HP) uses a 9 item scale with a cut off >28.5[108], The Persian MISS-HP uses a cut off >36.5[93] bSome papers modified the Likert scale and used a cut off of 3+ “sometimes” and 4+ “often[51] or 3+ “agree”[70, 91] cPMIE questions precede the MIOS symptoms scale and can be quantified as endorsed (yes/no). PTED; post-traumatic embitterment disorder, MIES; moral injury events scale; EMIS-M; Expressions of Moral Injury Scale-Military Version; MISS-HP; Moral Injury Symptoms Scale – Health Professionals, MIA-PSP; Moral Injury Assessment for Public Safety Personnel, MIQ-M; Moral Injury Questionnaire—Military version, BMIS; Brief Moral Injury Screen; EMIS-SF; Expressions of Moral Injury Scale-Short Form, MIQ-T; Moral Injury Questionnaire—Teacher version, AoW; Atrocities of War, PCoW; Psychological consequences of War, LFB; Leadership Failure or Betrayal, MIOS; Moral Injury Outcome Scale

For peer review only - http://bmjopen.bmj.com/site/about/guidelines.xhtml

Prevalence meta-analyses:

Supplementary Figures 1-9 show the forest plots with pooled prevalence estimates for PTED and moral injury, split by occupation where possible (k>10).

PTED

The pooled prevalence of PTED when using a cut-off of ≥1.6 was 37% 95% CI [24.4%, 49.9%], when using a cut-off of ≥2 was 26% 95% CI [16.1%, 37.4%], and when using a cut-off of ≥2.5 was 11% 95% CI [4%, 21.3%]. No subgroup analyses were conducted due to k≤10.

Moral injury

Using the MISS-HP, the pooled prevalence of clinically relevant signs of moral injury in healthcare professionals was 45% 95% CI [36.4%, 53.5%]. Across occupations, the pooled prevalence of exposure to any PMIE on the MIES scale was 67% 95% CI [59.0%, 74.5%], exposure to perpetration based PMIEs was 20% 95% CI [1.1%, 53.7%], transgressions by self PMIEs was 31% 95% CI [24.7%, 37.5%], transgressions by others PMIEs was 48% 95% CI [38.0%, 57.5%], and betrayal based PMIEs was 46% 95% CI [39.5%, 52.6%]. Whilst not significantly different, exposure to any PMIE was lowest in first responders (58%, 95% CI [30.9%, 82.5%]) followed by the armed forces (59%, 95% CI [45.9%, 72.4%]), healthcare professionals (71%, 95% CI [60.7%, 81.0%]) and educators (88%, 95% CI [82.5%, 91.7%]). Furthermore, whilst not significantly different, exposure to transgressions by self were also lowest in first responders (21%, 95% CI [16.1%, 26.8%]), followed by CPS professionals (24%, 95% CI [11.4%, 40.2%]), the armed forces (29%, 95% CI [19.9%, 39.6%]), healthcare professionals (33%, 95% CI [23.4%, 43.9%]) and educators (43%, 95% CI [36.0%, 49.5%]).

There were significant differences in the prevalence of exposure to transgressions by others, and betrayal based PMIEs based on occupation (*p*'s<0.01). Lack of overlap in CI shows the armed forces were exposed to significantly fewer transgressions by others (31%,

For peer review only - http://bmjopen.bmj.com/site/about/guidelines.xhtml

95% CI [20.1%, 41.8%]) compared to healthcare professionals (55%, 95% CI [43.9%, 65.6%]), first responders (61%, 95% CI [54.3%, 66.9%]), educators (80%, 95% CI [74.4%, 85.3%]) and CPS professionals (92%, 95% CI [78.6%, 98.3%]), though first responders, educators and CPS professionals included only one study each. Furthermore, the armed forces were exposed to significantly fewer betrayal events (33%, 95% CI [26.3%, 39.4%]) compared to healthcare workers (58%, 95% CI [51.0%, 65.1%]) and educators (68%, 95% CI [61.3%, 74.0%]). Whilst not significantly different, first responders had higher estimates than the armed forces (52%, 95% CI [35.2%, 69.1%]), and those in the CPS had the lowest exposure to betrayal, but this was based on only one study (32%, 95% CI [17.5%, 48.7%]).

Mean scores meta-analyses:

Supplementary Figures 10-24 show the forest plots with pooled mean estimates for PTED and moral injury, split by occupation where possible (k>10).

PTED

The pooled mean for the PTED scale across occupations was 1.52, and this was not significantly different according to occupation due to wide confidence intervals.

Moral injury

Since most moral injury scales only report average scores (i.e., Mean and SD) and not prevalence estimates, pooled mean estimates for the Moral Injury Questionnaire—Military version (MIQ-M), Expressions of Moral Injury Scale (EMIS), Moral Injury Outcome Scale (MIOS), MISS-HP and MIES were conducted. Only the pooled mean for transgressions by others on the MIES (M=3.16) significantly differed across occupations (p<0.01), with significantly lower scores in the armed forces (2.66, 95% CI [2.23, 3.09]) compared to first responders, educators, and CPS.

Sensitivity analyses: outliers removed

For peer review only

Table 3 shows the pooled prevalence and mean estimates, before and after removing outliers for PTED and moral injury.

Prevalence

Pooled prevalence estimates for PTED decreased when outliers were removed, using all three cut-offs. Prevalence was reduced to 31% 95% CI [23.9%, 39.2%] I2 = 84%, when using a cut off ≥1.6, reduced to 21% 95% CI [16.4%, 26.4%] I2 = 75%, when using a cut off ≥2 and decreased to 8% 95% CI [6.4%, 10.1%] I2 = 62%, when using a cut off ≥2.5.

The prevalence of moral injury in healthcare professionals on the MISS-HP was 43%, 95% CI [39.3%, 47.4%] I2 = 85%, and the prevalence of exposure to transgressions by self PMIEs decreased to 23% 95% [CI 21.6%, 24.3%] I2 = 4%. All other types of PMIEs increased, for example, exposure to any PMIE increased to 73% 95% CI [70.2%, 76.4%] I2 = 76%, transgressions by others increased to 49% 95% CI [43.8%, 54.6%] I2 = 93%, and betrayal stayed relatively similar at 46% 95% CI [41.0%, 51.3%] I2 = 93%. Except for exposure to transgressions by self, heterogeneity remained high even after removing outliers (≥50%).

Mean scores

Pooled mean estimates decreased for six of the moral injury scales or subscales after removing outliers and increased for six of the scales (see Table 3). Except for the MIOS trust subscale, heterogeneity remained high even after removing outliers (≥50%).

<u>Subgroup analyses: study quality and cut-off value</u>

Additional subgroup analyses were conducted based on the risk of bias where possible (k≥10; Supplementary Table 4). For prevalence meta-analyses, exposure to any PMIE was significantly lower in low-risk studies (47%, 95% CI [32.4, 61.9], k=5) compared to medium-risk (72%, 95% CI [64.8%, 79.2%], k=18, p=0.003). Furthermore, exposure to transgressions by self was significantly lower in low-risk studies (16%, 95% CI [84.7%, 25.2%], k=4)

For peer review only - http://bmjopen.bmj.com/site/about/guidelines.xhtml

compared to medium-risk (35%, 95% CI [28.3%, 41.8%], *k*=22, *p*=0.006), and exposure to betrayal PMIEs was significantly lower in low-risk studies (30%, 95% CI [18.5%, 43.7%], *k*=5) compared to medium-risk studies (50%, 95% CI [43.1%, 56.3%], *k*=23, p=0.011). The pooled prevalence estimates of transgressions by others were not significantly different based on the risk of bias (*p*=0.254). When comparing pooled mean estimates based on the risk of bias, only the pooled mean for betrayal was significantly lower in low-risk studies (2.03, 95% CI [1.33, 2.73], *k*=3) compared to medium-risk (2.83, 95% CI [2.60, 3.06], *k*=27, p=0.034). For the prevalence meta-analyses, different cut-off values were used to signify moral injury on the MIES and the MISS-HP (see Table 2). Therefore, additional subgroup analyses based on the cut-off value were conducted (see Supplementary Table 5) and found no significant differences in pooled prevalence estimates depending on the cut-off value (p's>0.05).

Table 3.
Pooled prevalence and mean estimates for PTED and moral injury, with and without outliers

Measure	With outliers			Without outliers		
	k	Pooled % [95% CI]	I2	*k*	Pooled % [95% CI]	I2
PTED 1.6	9	36.65 [24.38, 49.85]	98%	6	31.27 [23.87, 39.17]	84%
PTED 2	8	26.03 [16.09, 37.38]	97%	7	21.21 16.40, 26.44]	75%
PTED 2.5	6	22.31 [4.02, 21.32]	98%	7	8.16 [6.37, 10.14]	62%
MIES any	23	67 [59.01, 75.54]	98%	11	73.36 [70.18, 76.42]	76%
MIES transgressions-self	27	30.95 [24.72, 37.54]	99%	11	22.99 [21.62, 24.32]	4%
MIES transgressions-others	25	47.73 [38.04, 57.51]	98%	14	49.20 [43.82, 54.59]	93%
MIES betrayal	28	46.03 [39.54, 52.59]	98%	11	46.12 [40.97, 51.32]	91%
MIES perpetration	2	20.26 [1.11, 52.70]	98%		No outliers	
MISS-HP	10	44.88 [36.40, 53.50]	98%	7	43.33 [39.30, 47.41]	85%
	k	Pooled mean [95% CI]	I2	*k*	Pooled mean [95% CI]	I2

For peer review only - http://bmjopen.bmj.com/site/about/guidelines.xhtml

PTED	11	1.52 [1.10, 1.93]	99%	7	1.33 [1.18, 1.49]	97%
MIES total	29	2.58 [2.35, 2.82]	99%	16	2.61 [2.52, 2.70]	86%
MIES transgressions-self	27	2.26 [1.99, 2.52]	99%	12	2.16 [2.95, 2.28]	77%
MIES transgressions-others	24	3.16 [2.81, 3.52]	99%	10	3.05 [2.89, 3.20]	83%
MIES betrayal	30	2.74 [2.51, 2.98]	99%	15	2.77 [2.67, 2.86]	80%
MIES perpetration	5	2.55 [2.09, 3.02]	99%	4	2.77 [2.60, 2.95]	83%
MIQ-M total	5	1.77 [1.58, 1.96]	97%	4	1.84 [1.66, 2.02]	91%
MIQ-M causes	4	1.42 [1.21, 1.60]	96%	3	1.31 [1.26, 1.36]	75%
EMIS total	4	2.22 [1.90, 2.54]	99%		No outliers	
EMIS self	4	1.96 [1.68, 2.24]	92%		No outliers	
EMIS others	5	2.51 [2.28, 2.74]	93%	4	2.61 [2.46, 2.76]	55%
MISS-HP	12	36.73 [33.39, 40.07]	99%	7	35.42 [34.14, 39.87]	76%
MIOS total	4	27.75 [24.19, 31.31]	95%	3	25.88 [24.45, 27.20]	66%
MIOS shame	4	12.77 [10.43, 15.12]	98%	3	11.42 [11.01, 11.83]	0%
MIOS trust	4	14.97 [13.65, 16.28]	94%		No outliers	

PTED; post-traumatic embitterment disorder, MIES; moral injury events scale; EMIS; Expressions of Moral Injury Scale; MISS-HP; Moral Injury Symptoms Scale – Health Professionals, MIQ-M; Moral Injury Questionnaire—Military version, MIOS; Moral Injury Outcome Scale

For peer review only

Narrative synthesis:

Prevalence and mean scores

Supplementary Table 6 shows the prevalence and average scores for moral injury scales that could not be combined (*k*=1) including different variations of MIES subscales (i.e., MI-other; 20%, MI-commission; 14%, MI-omission; 5%), or moral injury profiles based on the MIES (no moral distress; 42%, moral distress-other; 19%, witnessing-only; 16%, moral distress-self; 8%, moral distress-self and other; 15%). Variations of the MIQ-M include the teacher version (MIQ-T; M=1.16), or the modified version. Other moral injury scales

For peer review only - http://bmjopen.bmj.com/site/about/guidelines.xhtml

were only included in one study each, such as the Moral Injury Assessment for Public Safety Personnel (MIA-PSP; M=59.6), or the Toronto moral injury scale for journalists (M=1.5). See supplementary Table 6 for specific average scores or prevalence estimates for the associated subscales.

Univariate associates of PTED and moral injury

A narrative review was used to synthesise the associates of PTED and moral injury due to the heterogeneity of variables measured, with the exception of mental health variables which were frequently measured in moral injury studies, however, meta-analyses of this relationship have been conducted recently.[24, 116, 117]

Demographic variables:

Of the PTED studies, six (55%) measured demographic variables. Overall, there was no association with gender, a positive association with length of time in the job, and a mixed association with age, with two papers finding a positive association, and two papers finding no association. Many variables were measured less frequently across studies (i.e., k<3), such as ethnicity, professional role, hours worked, having dependents, marital status, income, and education level. Of the moral injury studies, 31 (40%) measured demographic variables. Overall, there was no association with gender, age, length of time in the job, marital status, ethnicity/race/country of birth, education, and income. Several variables were measured less frequently (i.e., k<3), such as being unemployed and having dependents, and several papers measured variables specific to the work environment such as professional role, though varied job roles preclude comparison.

Mental health variables:

Of the PTED studies, seven (64%) measured mental health variables and found positive associations with depression, stress, anxiety, existing mental health diagnoses, psychological distress, negative affect, and a negative association with positive affect and

For peer review only - http://bmjopen.bmj.com/site/about/guidelines.xhtml

employee well-being, though none of these variables were measured frequently (k<3). Of the moral injury studies, 51 (65%) measured mental health variables and found positive associations with depression, PTSD or PTSD subscales, guilt or shame, anger, anxiety, burnout or its subscales, drug and alcohol use, and a negative association to well-being. Other variables related to mental health appeared less frequently (i.e., k<3).

Individual/work characteristics:

Of the PTED studies, 10 (91%) measured individual/work characteristics. Justice beliefs were negatively associated with PTED. Other variables were measured less frequently (k<3) such as rumination, unemployment duration and appraisal, supervisory control, job demands, work attitudes, social support, self-efficacy, leadership behaviours, and personality traits such as optimism, self-esteem, and resilience, though generally PTED was associated with undesirable work factors. Of the moral injury studies, 40 (51%) measured individual/work characteristics. The most common positive association was with combat exposure. Religiosity was negatively associated with moral injury, although two papers found a positive association, turnover intentions were positively associated with moral injury, and resilience associations were mixed, showing positive, negative, and no associations with moral injury. COVID-related factors were measured across several studies, but variations in the type of COVID variables measured preclude comparison. Many variables related to personality variables, beliefs, and specific work environmental factors were measured less frequently (k<3).

Discussion

This is the first meta-analysis of the prevalence and average scores of occupational moral injury and PTED so the first aim of the paper has been met. The results showed that across occupations, 37% of staff scored above a differentiation point for elevated embitterment (≥1.6), 26% had clinically relevant embitterment (≥2), and 11% showed

For peer review only - http://bmjopen.bmj.com/site/about/guidelines.xhtml

embitterment of a clinically significant intensity with improved clinical practicability and specificity (≥2.5). Findings also showed that across occupations, 67% were exposed to at least one PMIE, 20% were exposed to a perpetration based PMIE, 31% committed a moral transgression, 48% witnessed a transgression by others, and 46% were exposed to betrayal PMIEs at work. The second aim was to establish whether prevalence estimates differ depending on occupation and this aim has been partially met because some moral injury scales were occupation specific or used different subscales meaning they could not be pooled across occupations. Despite this, exposure to transgressions by others and betrayal were significantly lower in the armed forces compared with healthcare workers and educators. Finally, clinical levels of moral injury in healthcare professionals, according to the MISS-HP, was 45%. Since several moral injury scales did not provide prevalence estimates, mean scores were also pooled, and for PTED, did not differ according to occupation. Mean scores of exposure to transgressions-by others were significantly lower in the armed forces compared to first responders, CPS, and educators.

The third aim was to determine whether methodological differences between studies influenced prevalence estimates, and this was shown by methodological differences between studies precluding some analyses. In addition, prevalence estimates for exposure to any PMIE, transgressions by self and betrayals were lower in low risk compared to medium-risk studies, and the mean scores for betrayal was also lower in low-risk studies. Furthermore, prevalence estimates for moral injury were not significantly different based on the cut-off value used by the researcher. The final aim of the systematic review was to record the variables associated with moral injury and PTED. A narrative review identified variables measured in association with moral injury and PTED which fell into three categories: demographics, mental health, and individual/work characteristics. Gender was found to have no association with moral injury and PTED, whereas the length of time in the job had a

For peer review only - http://bmjopen.bmj.com/site/about/guidelines.xhtml

positive association, and age had a positive or no association with PTED. In contrast, age, length of time in the job, marital status, ethnicity/race/country of birth, education and income all had no association with moral injury. Other demographic variables varied and were usually only included in one or two studies. Moral injury and PTED showed a positive association with mental health variables including depression, stress, anxiety, and employee well-being, and moral injury studies studied the relationship with a wider range of disorders such as PTSD and anxiety, and emotional (i.e., guilt, anger, shame), behavioural (i.e., burnout, compassion fatigue) and spiritual variables. Finally, individual/work variables differed between the two constructs, with moral injury studies focusing primarily on the combat environment as a risk for military samples and PTED focusing on justice beliefs as protective in civilian samples. Furthermore, religiosity and resilience were measured in relation to moral injury with mixed results, and turnover intentions were positively associated with moral injury. Individual risk and resiliency factors such as optimism, self-esteem and personality traits were also identified, but less frequently. Due to the infrequency of variables measured, the associations reported here are inconclusive, and this aim has only been partially met.

The results of this study should be considered in light of several strengths and limitations. The study protocol was pre-registered on Prospero and followed the Joanna Briggs Institute guidance for systematic reviews of prevalence and incidence data which ensures the methods were robust.[27] In line with guidance from the Cochrane handbook[118] two researchers screened 15% of all abstracts and titles, and 100% of full texts, data extraction and quality assessment. However, using three screeners would improve objectivity. Despite using robust methods, there was high heterogeneity within individual studies, and amongst studies, even after employing subgroup analyses to explain the variability based on study quality, the cut-off score used and sensitivity analyses to remove

For peer review only - http://bmjopen.bmj.com/site/about/guidelines.xhtml

outliers. Whilst heterogeneity is often high in prevalence meta-analyses, and the median *I*2 was found to be 96.9% in a review of 134 prevalence meta-analyses[119], caution must be taken when interpreting our findings. Furthermore, only 11% of studies had a low risk of bias, and often the primary aim of the included studies was not to produce prevalence data. This may explain the use of inappropriate statistical methods (i.e., lack of confidence intervals or weighting), and variability in reporting only average scores or prevalence data based on the various subscale configurations or using different cut-off values. To deal with this heterogeneity, separate meta-analyses were conducted based on the type of data reported (i.e., prevalence or average scores), and the measure used, reducing the number of studies included in each meta-analysis and thus power, meaning that subgroup analyses based on occupation were not always possible. Finally, whilst we searched a relatively high number of databases, it is possible that references from the grey literature have been missed.

Despite the limitations, this review adds to the growing literature on the prevalence of moral injury and PTED. Prior estimates of PTED in the general population were at 2.5%[3] and this compares to 26% across occupations in this review. This confirms the findings by Linden et al.,[11] which found that 73% of PTED patients reported the triggering event as work-related and shows unjust and embittering events often occur in the workplace. Using the most stringent cut-off, occupational health services could expect 11% of their staff to display clinically severe levels of PTED according to these findings. There were not enough studies to conduct subgroup analyses based on the prevalence of PTED across occupations, though subgroup analyses using the mean PTED scores showed no significant difference based on occupation. This might suggest that PTED is widespread across professions. However, this analysis was based on only 11 studies, and the inclusion of more studies would be needed to confirm that this effect is not due to low statistical power.

For peer review only - http://bmjopen.bmj.com/site/about/guidelines.xhtml

Previous attempts to collate prevalence estimates for moral injury have relied on a narrative synthesis of the prevalence of morally transgressive acts[22] or moral injury symptoms in military samples.[23] Findings from this meta-analysis shows that 67% of people across occupations have been exposed to at least one PMIE in their jobs, which is a high proportion. Furthermore, staff were more often exposed to transgressions by others (48%) and betrayal PMIEs (46%) than PMIEs perpetrated by themselves (31%) which mirrors qualitative findings during the pandemic, which shows healthcare staff experienced institutional betrayal more than perpetration based moral injuries.[120, 121] Estimates of exposure to PMIEs are taken from the MIES,[22] which was the most widely used scale to measure moral injury across studies in this review. Whilst it is arguable that not all individuals exposed to PMIEs will experience moral injury symptoms, the MIES does confound events and effects by asking participants if they feel troubled by their exposure to moral violations.[24] This suggests that a high proportion of staff are not only exposed to events that are morally injurious, but are also affected by this. This is supported by further findings from this meta-analysis which showed that 45% of healthcare workers had clinically relevant signs of moral injury according to the MISS-HP, which is the only moral injury symptom scale which uses a clinical cut off.[94] Since the MISS-HP is healthcare specific, and the other symptom scales in this review only reported mean scores and not prevalence estimates, it is not possible to determine whether any occupations are at a greater risk of clinically relevant moral injury.

However, the review was able to show that members of the armed forces were exposed to significantly fewer transgressions by others and betrayal PMIEs than healthcare workers and educators, and the mean scores for transgressions by others were significantly lower in the armed forces than first responders, CPS, and educators. Therefore, despite the definitions of moral injury originating from the military[4, 5], this shows that civilian

For peer review only - http://bmjopen.bmj.com/site/about/guidelines.xhtml

occupations are at an even greater risk of exposure to PMIEs and of feeling troubled by these. This mirrors findings which show that certain occupations have a higher risk of developing work-related PTSD, such as professions that do not have special training to deal with traumatic events.[1] It is possible that military training is a protective factor and armed forces are less troubled by betrayal trauma as a result.

This meta-analysis identified several methodological issues. Prevalence of exposure to any PMIE, transgressions by self and betrayals were lower in low risk compared to medium-risk studies, and the prevalence of PTED differed depending on the cut-off value used. As highlighted by Koenig, Pearce, and Youssef[24], there are several moral injury scales, which conflate events and symptoms (MIES, MIQ), and our review has identified more recently developed scales which also do this (i.e., MIA-PSP, Toronto scale for journalists). Other scales only measure symptoms (i.e., EMIS, MIOS, MISS-HP), while many scales are occupation-specific precluding comparisons across occupations, and not all scales have established cut-off values, which contributes to inconsistencies in this area. Furthermore, there is a lack of randomly selected, nationally representative, epidemiological studies on the prevalence of moral injury and PTED in specific populations of interest (i.e., the armed forces, HSCWs, first responders, educators, journalists, CPS employees) and in the general working population.

These findings reveal several implications and gaps that can form the basis of future research. First, clinically relevant signs of PTED are much higher in work settings than in the general population, and just under half of the healthcare professionals showed clinical levels of moral injury. Furthermore, occupations such as healthcare professionals, educators, and the CPS are especially likely to experience transgressions by others and healthcare professionals and educators are also more likely to experience betrayals at work than the armed forces. Understanding the prevalence of moral injury and PTED in different

For peer review only

For peer review only - http://bmjopen.bmj.com/site/about/guidelines.xhtml

occupations is critical for developing targeted prevention strategies and can guide policy. However, these occupations relate to statutory organisations and frontline services, and it is possible that other civilian occupations have a lower, or moderate risk of exposure to PMIEs, and moral injury. Research across a broader range of professionals to understand which civilian occupations are at low, moderate, and high risk of exposure to PMIEs, moral injury and PTED would be valuable. The narrative synthesis showed that moral injury and PTED are associated with poor mental health outcomes such as depression, stress and anxiety, and poor work outcomes such as sickness absence, and turnover intentions. Therefore, employers, occupational health, and clinicians should be aware of these conditions and provide appropriate support. Currently, there are no proven recommended treatments for moral injury and PTED, and this research shows organisations will need strategies to support employees given the high risk of experiencing moral injury and PTED. Standardised measurements of moral injury and PTED, and proven treatment options need to be developed. In addition, whilst the number of studies on moral injury has grown exponentially in recent years, research on occupational PTED still represents a significant gap, and the lack of studies precluded prevalence subgroup analyses based on occupation. More studies are needed in this area. Second, whilst there were more moral injury studies, most were conducted with military or healthcare professionals, and other occupations such as educators, journalists, CPS, and first responders were represented in only one or two studies. Prevalence estimates indicate educators and CPS employees may be at an especially high risk, and more studies are needed to confirm these findings. Finally, most moral injury studies were conducted in the US, and studies are needed across more geographical locations.

In conclusion, we are the first to provide meta-analytic evidence that the prevalence of moral injury and PTED at work are high. We identified occupations at a greater risk of exposure to PMIEs but inconsistencies in measurement scales, subscale configurations or cut-

For peer review only - http://bmjopen.bmj.com/site/about/guidelines.xhtml

off values used amongst studies precluded some analyses. To strengthen the reliability of these findings, researchers must agree on a gold standard approach to measuring moral injury and PTED, across a range of low, moderate, and high-risk occupations with appropriate cut off values. Despite this, high prevalence rates of moral injury and PTED show that proven, recommended treatment options and guidance should be developed to support employees and can inform policy makers on which areas of research and prevention to prioritise.

Figure 1.
Preferred Reporting Items for Systematic Reviews and Meta-Analyses (PRISMA) flow diagram.

Contributor and guarantor information:

CB designed the project and data collection tools, collected the data, cleaned, analysed and interpreted the data and drafted and revised the paper. She is guarantor. CR assisted with the study design, data collection and revised the paper. JC assisted with the study design and revised the paper. All authors gave final approval for the publication of this manuscript. The corresponding author attests that all listed authors meet authorship criteria and that no others meeting the criteria have been omitted.

Copyright:

The Corresponding Author has the right to grant on behalf of all authors and does grant on behalf of all authors, an exclusive licence (or non-exclusive for government employees) on a worldwide basis to the BMJ Publishing Group Ltd to permit this article (if accepted) to be published in BMJ editions and any other BMJPGL products and sublicences such use and exploit all subsidiary rights, as set out in our licence.

For peer review only - http://bmjopen.bmj.com/site/about/guidelines.xhtml

Competing interest statement:

None declared

Funding:

CB was supported by the Economic and Social Research Council UK [ES/P000665/1].

Role of sponsor:

The study sponsors did not play a role in the study design, management, data collection, analyses, in the interpretation of the data or in the writing of the manuscript or the decision to submit for publication.

Transparency declaration:

The lead author affirms that this manuscript is an honest, accurate, and transparent account of the study being reported; that no important aspects of the study have been omitted; and that any discrepancies from the study as planned have been explained.

Ethical approval:

Ethical approval is not required for this study design.

Data availability statement:

No additional data are available.

Dissemination declaration:

Results from this study will be disseminated to relevant audiences through University of Liverpool communications.

For peer review only

References:

1. Skogstad M, Skorstad M, Lie A, et al. Work-related post-traumatic stress disorder. Occupational medicine. 2013 Apr 1;63(3):175-82. https://doi.org/10.1093/occmed/kqt003
2. Lee W, Lee YR, Yoon JH, et al. Occupational post-traumatic stress disorder: an updated systematic review. BMC public health. 2020 Dec;20(1):1-2. https://doi.org/10.1186/s12889-020-08903-2
3. Linden M, Baumann K, Lieberei B, et al. The post-traumatic embitterment disorder self-rating scale (PTED scale). Clinical Psychology & Psychotherapy: An International Journal of Theory & Practice. 2009 Mar;16(2):139-47. https://doi.org/10.1002/cpp.610
4. Litz BT, Stein N, Delaney E, et al. Moral injury and moral repair in war veterans: A preliminary model and intervention strategy. Clinical psychology review. 2009 1;29(8):695-706. https://doi.org/10.1016/j.cpr.2009.07.003
5. Shay J. Moral injury. Psychoanalytic psychology. 2014 Apr;31(2):182. https://doi.org/10.1037/a0036090
6. Litz BT, Plouffe RA, Nazarov A, Murphy D, Phelps A, Coady A, Houle SA, Dell L, Frankfurt S, Zerach G, Levi-Belz Y. Defining and assessing the syndrome of moral injury: Initial findings of the moral injury outcome scale consortium. Frontiers in psychiatry. 2022 Jul 5;13:923928.
7. Linden M, Maercker A, editors. Embitterment: Societal, psychological, and clinical perspectives. Springer Science & Business Media; 2011 Feb 26.
8. Williamson V, Stevelink SA, Greenberg N. Occupational moral injury and mental health: systematic review and meta-analysis. The British Journal of Psychiatry. 2018 Jun;212(6):339-46. DOI: https://doi.org/10.1192/bjp.2018.55

For peer review only - http://bmjopen.bmj.com/site/about/guidelines.xhtml

9. Brennan CJ, McKay MT, Cole JC. Morally injurious events and post-traumatic embitterment disorder in UK health and social care professionals during COVID-19: a cross-sectional web survey. BMJ open. 2022 May 1;12(5):e054062. doi: 10.1136/bmjopen-2021-054062

10. Carter C. Organisational injustice in UK frontline services and onset of Moral Injury, Post Traumatic Embitterment Disorder (PTED) and PTSD. International journal of law, crime and justice. 2021 Sep 1;66:100483 https://doi.org/10.1016/j.ijlcj.2021.100483

11. Linden M, Rotter M, Baumann K, et al. Posttraumatic embitterment disorder: Definition, evidence, diagnosis, treatment. Hogrefe & Huber Publishers; 2007.

12. Sabic D, Sabic A, Batic-Mujanovic O. Embitterment in war veterans with posttraumatic stress disorder. Medical Archives. 2018 Apr;72(2):125. doi: 10.5455/medarh.2018.72.125-130

13. Muschalla B, Rau H, Willmund GD, Knaevelsrud C. Work disability in soldiers with posttraumatic stress disorder, posttraumatic embitterment disorder, and not-event-related common mental disorders. Psychological trauma: theory, research, practice, and policy. 2018 Jan;10(1):30. https://doi.org/10.1037/tra0000293

14. Rubab M, Tariq F. Embitterment, Self-Efficacy and Mental Health Problems in private school teachers in Pakistan. PSYCHOLOGY AND EDUCATION. 2022;59(2):572-81.

15. Saleem A, Bashir M, Abrar M. Ethical behaviors by leaders act as a stimulant to the wellbeing of employees by restraining workplace embitterment. Frontiers in Public Health. 2022 Sep 29;10:974642. DOI: 10.3389/fpubh.2022.974642

For peer review only

16. Dunn JM, Sensky T. Psychological processes in chronic embitterment: The potential contribution of rumination. Psychological Trauma: Theory, Research, Practice, and Policy. 2018 Jan;10(1):7. https://doi.org/10.1037/tra0000291

17. Sensky T, Salimu R, Ballard J, et al. Associations of chronic embitterment among NHS staff. Occupational Medicine. 2015 Aug 1;65(6):431-6. https://doi.org/10.1093/occmed/kqv089

18. Shin J, You M. Embitterment among the unemployed: A multiple mediation model of belief in a just world. Work. 2022: 1-0. DOI: 10.3233/WOR-205228

19. Linden M, Rotter M. Unemployment and embitterment in contrast to general psychological distress. Work. 2019 Jan 1;62(1):133-8. DOI: 10.3233/WOR-182848

20. Michailidis E, Cropley M. Exploring predictors and consequences of embitterment in the workplace. Ergonomics. 2017 Sep 2;60(9):1197-206. https://doi.org/10.1080/00140139.2016.1255783

21. Michailidis E, Cropley M. Investigating the predictors of workplace embitterment using a longitudinal design. Occupational Medicine. 2018 Nov 16;68(8):523-9. https://doi.org/10.1093/occmed/kqy121

22. Frankfurt S, Frazier P. A review of research on moral injury in combat veterans. Military psychology. 2016 Jun 9;28(5):318-30. https://doi.org/10.1037/mil0000132

23. Koenig HG, Al Zaben F. Moral injury: An increasingly recognized and widespread syndrome. Journal of religion and health. 2021 Oct;60(5):2989-3011. https://doi.org/10.1007/s10943-021-01328-0

For peer review only

For peer review only - http://bmjopen.bmj.com/site/about/guidelines.xhtml

24. Koenig HG, Youssef NA, Pearce M. Assessment of moral injury in veterans and active duty military personnel with PTSD: A review. Frontiers in psychiatry. 2019 Jun 28;10:443. https://doi.org/10.3389/fpsyt.2019.00443

25. Linden M, Baumann K, Rotter M, Schippan B. Diagnostic criteria and the standardized diagnostic interview for posttraumatic embitterment disorder (PTED). International Journal of Psychiatry in Clinical Practice. 2008 Jan 1;12(2):93-6. https://doi.org/10.1080/13651500701580478

26. Page MJ, McKenzie JE, Bossuyt PM, Boutron I, Hoffmann TC, Mulrow CD, Shamseer L, Tetzlaff JM, Akl EA, Brennan SE, Chou R. The PRISMA 2020 statement: an updated guideline for reporting systematic reviews. Systematic reviews. 2021 Dec;10(1):1-1. https://doi.org/10.1186/s13643-021-01626-4

27. Munn Z, Moola S, Lisy K, Riitano D, Tufanaru C. Methodological guidance for systematic reviews of observational epidemiological studies reporting prevalence and cumulative incidence data. International journal of evidence-based healthcare. 2015 Sep 1;13(3):147-53. DOI: 10.1097/XEB.0000000000000054

28. Brennan C, Roberts C. A systematic review examining the prevalence of work-related moral injury and post-traumatic embitterment disorder (PTED). PROSPERO. 2020 Jul 3. CRD42020191766. Available from: https://www.crd.york.ac.uk/prospero/display_record.php?ID=CRD42020191766

29. Linden M. Posttraumatic embitterment disorder. Psychotherapy and psychosomatics. 2003;72(4):195-202. https://doi.org/10.1159/000070783

30. McHugh ML. Interrater reliability: the kappa statistic. Biochemia medica. 2012 Oct 15;22(3):276-82.30

31. Hoy D, Brooks P, Woolf A, et al. Assessing risk of bias in prevalence studies: modification of an existing tool and evidence of interrater agreement. Journal of

For peer review only

clinical epidemiology. 2012 Sep 1;65(9):934-9. https://doi.org/10.1016/j.jclinepi.2011.11.014

32. Balduzzi S, Rücker G, Schwarzer G. How to perform a meta-analysis with R: a practical tutorial. Evidence-Based Mental Health. 2019. p. 153–60.

33. Langan D, Higgins JP, Jackson D, Bowden J, Veroniki AA, Kontopantelis E, Viechtbauer W, Simmonds M. A comparison of heterogeneity variance estimators in simulated random-effects meta-analyses. Research synthesis methods. 2019 Mar;10(1):83-98. https://doi.org/10.1002/jrsm.1316

34. Schwarzer G. Meta-Analysis in R. Systematic Reviews in Health Research: Meta-Analysis in Context. 2022 Apr 22:510-34. https://doi.org/10.1002/9781119099369.ch26

35. Borenstein M, Hedges LV, Higgins JP, Rothstein HR. Introduction to meta-analysis. John Wiley & Sons; 2021 Apr 6.

36. Cumming G, Finch S. Inference by eye: confidence intervals and how to read pictures of data. American psychologist. 2005 Feb;60(2):170. https://doi.org/10.1037/0003-066X.60.2.170

37. Lipsey MW, Wilson DB. Practical meta-analysis. SAGE publications, Inc; 2001.

38. Viechtbauer W. Conducting meta-analyses in R with the metafor package. Journal of statistical software. 2010;36(3):1-48. DOI: 10.18637/jss.v036.i03

39. Higgins JP, Thompson SG. Quantifying heterogeneity in a meta-analysis. Statistics in medicine. 2002 Jun 15;21(11):1539-58. https://doi.org/10.1002/sim.1186

40. Wang N. How to conduct a meta-analysis of proportions in R: a comprehensive tutorial. New York, NY, John Jay College of Criminal Justice, 0–62. 2018 Jun.

For peer review only

41. Viechtbauer W, Cheung MW. Outlier and influence diagnostics for meta-analysis. Research synthesis methods. 2010 Apr;1(2):112-25. https://doi.org/10.1002/jrsm.11

42. Popay J, Roberts H, Sowden A, Petticrew M, Arai L, Rodgers M, Britten N, Roen K, Duffy S. Guidance on the conduct of narrative synthesis in systematic reviews. A product from the ESRC methods programme Version. 2006 Apr 1;1(1):b92.

43. Karatuna I, Gök S. A study analyzing the association between post-traumatic embitterment disorder and workplace bullying. Journal of Workplace Behavioral Health. 2014 Apr 3;29(2):127-42

44. Bryan CJ, Bryan AO, Anestis MD, et al. Measuring moral injury: Psychometric properties of the moral injury events scale in two military samples. Assessment. 2016 Oct;23(5):557-70. https://doi.org/10.1177/1073191115590855

45. Currier JM, Holland JM, Drescher K, et al. Initial psychometric evaluation of the Moral Injury Questionnaire—Military version. Clinical psychology & psychotherapy. 2015 Jan;22(1):54-63. https://doi.org/10.1002/cpp.1866

46. Currier JM, Holland JM, Rojas-Flores L, et al. Morally injurious experiences and meaning in Salvadorian teachers exposed to violence. Psychological trauma: theory, research, practice, and policy. 2013;7(1):24. https://doi.org/10.1037/a0034092

47. Currier JM, Farnsworth JK, Drescher KD, et al. Development and evaluation of the Expressions of Moral Injury Scale—Military Version. Clinical psychology & psychotherapy. 2017 May;25(3):474-88. https://doi.org/10.1002/cpp.2170

48. Forkus SR, Breines JG, Weiss NH. Morally injurious experiences and mental health: The moderating role of self-compassion. Psychological trauma: theory,

For peer review only

For peer review only - http://bmjopen.bmj.com/site/about/guidelines.xhtml

research, practice, and policy. 2019 Sep;11(6):630. https://doi.org/10.1037/tra0000446

49. Frankfurt SB, DeBeer BB, Morissette SB, Kimbrel NA, La Bash H, Meyer EC. Mechanisms of moral injury following military sexual trauma and combat in post-9/11 US war veterans. Frontiers in psychiatry. 2018 Nov 2;9:520. https://doi.org/10.3389/fpsyt.2018.00520

50. Griffin BJ, Williams CL, Shaler L, Dees RF, Cowden RG, Bryan CJ, Litz B, Purcell N, Burkman K, Maguen S. Profiles of moral distress and associated outcomes among student veterans. Psychological Trauma: Theory, Research, Practice, and Policy. 2020 Oct;12(7):669. https://doi.org/10.1037/tra0000584

51. Jinkerson JD, Battles AR. Relationships between moral injury syndrome model variables in combat veterans. Traumatology. 2019 Mar;25(1):33. https://doi.org/10.1037/trm0000163

52. Jordan AH, Eisen E, Bolton E, Nash WP, Litz BT. Distinguishing war-related PTSD resulting from perpetration-and betrayal-based morally injurious events. Psychological Trauma: Theory, Research, Practice, and Policy. 2017 Nov;9(6):627. https://doi.org/10.1037/tra0000249

53. Lee H, Aldwin CM, Kang S. Do different types of war stressors have independent relations with mental health? Findings from the Korean Vietnam Veterans Study. Psychological trauma: theory, research, practice, and policy. 2022 Jul;14(5):840. https://doi.org/10.1037/tra0000557

54. Ogle AD, Reichwald R, Rutland JB. Psychological impact of remote combat/graphic media exposure among US Air Force intelligence personnel. Military Psychology. 2018 Nov 2;30(6):476-86. https://doi.org/10.1080/08995605.2018.1502999

For peer review only - http://bmjopen.bmj.com/site/about/guidelines.xhtml

55. Wisco BE, Marx BP, May CL, Martini B, Krystal JH, Southwick SM, Pietrzak RH. Moral injury in US combat veterans: Results from the national health and resilience in veterans study. Depression and anxiety. 2017 Apr;34(4):340-7. https://doi.org/10.1002/da.22614

56. Zerach G, Levi-Belz Y. Moral injury process and its psychological consequences among Israeli combat veterans. Journal of Clinical Psychology. 2018 Sep;74(9):1526-44. https://doi.org/10.1002/jclp.22598

57. Papazoglou K, Blumberg D, Briones-Chiongbian V, Russo C, Koskelainen M. Exploring the roles of moral injury and personality in police traumatization. Crisis, Stress, and Human Resilience: An International Journal. 2019 Apr 25;1(1):32-56.

58. Richardson CB, Chesnut RP, Morgan NR, et al. Examining the factor structure of the moral injury events scale in a veteran sample. Military medicine. 2020 Jan;185(1-2):e75-83. https://doi.org/10.1093/milmed/usz129

59. Feinstein A, Pavisian B, Storm H. Journalists covering the refugee and migration crisis are affected by moral injury not PTSD. JRSM open. 2018 Feb;9(3):2054270418759010. https://doi.org/10.1177/2054270418759010

60. Sugrue EP. Moral injury among professionals in K–12 education. American Educational Research Journal. 2020 Feb;57(1):43-68. https://doi.org/10.3102/00028312198486

61. Aldridge V, Scott H, Paskell R. Investigating the relationship between guilt and shame proneness and moral injury in veterans that have experienced active deployment. Military Behavioral Health. 2019 Jan 2;7(1):83-91. https://doi.org/10.1080/21635781.2019.1580641

For peer review only

62. Andrukonis A, Protopopova A. Occupational health of animal shelter employees by live release rate, shelter type, and euthanasia-related decision. Anthrozoös. 2020 Jan 2;33(1):119-31.

63. Haight W, Sugrue EP, Calhoun M. Moral injury among child protection professionals: Implications for the ethical treatment and retention of workers. Children and Youth Services Review. 2017 Nov 1;82:27-41. https://doi.org/10.1016/j.childyouth.2017.08.030

64. Battles AR, Kelley ML, Jinkerson JD, C. Hamrick H, F. Hollis B. Associations among exposure to potentially morally injurious experiences, spiritual injury, and alcohol use among combat veterans. Journal of Traumatic Stress. 2019 Jun;32(3):405-13. https://doi.org/10.1002/jts.22404

65. Braitman AL, Battles AR, Kelley ML, Hamrick HC, Cramer RJ, Ehlke S, Bravo AJ. Psychometric properties of a Modified Moral Injury Questionnaire in a military population. Traumatology. 2018 Dec;24(4):301. https://doi.org/10.1037/trm0000158

66. Lancaster SL, Harris JI. Measures of morally injurious experiences: A quantitative comparison. Psychiatry research. 2018 Jun 1;264:15-9. https://doi.org/10.1016/j.psychres.2018.03.057

67. Lancaster SL. Negative outcomes after morally injurious experiences: A replication and extension. Psychological Trauma: Theory, Research, Practice, and Policy. 2018 Jul;10(4):456. https://doi.org/10.1037/tra0000341

68. Akhtar M, Faize FA, Malik RZ, et al. Moral injury and psychological resilience among healthcare professionals amid COVID-19 pandemic. Pakistan Journal of Medical Sciences. 2022 May 18;38(5). https://doi.org/10.12669/pjms.38.5.5122

For peer review only

69. Qi M, Hu X, Liu J, et al. The impact of the COVID-19 pandemic on the prevalence and risk factors of workplace violence among healthcare workers in China. Frontiers in public health. 2022;10. https://doi.org/10.3389/fpubh.2022.938423

70. Amsalem D, Lazarov A, Markowitz JC, et al. Psychiatric symptoms and moral injury among US healthcare workers in the COVID-19 era. BMC psychiatry. 2021 Dec;21(1):1-8. https://doi.org/10.1186/s12888-021-03565-9

71. Perez D, Larson P, Bair J. US veterans experience moral injury differently based on moral foundations preferences. Journal of Community Engagement and Scholarship. 2021 Aug;13(4):13.

72. Benatov J, Zerach G, Levi-Belz Y. Moral injury, depression, and anxiety symptoms among health and social care workers during the COVID-19 pandemic: The moderating role of belongingness. International journal of social psychiatry. 2022:00207640221099421. https://doi.org/10.1177/00207640221099421

73. Borges LM, Holliday R, Barnes SM, et al. A longitudinal analysis of the role of potentially morally injurious events on COVID-19-related psychosocial functioning among healthcare providers. PloS one. 2021 Nov 12;16(11):e0260033. https://doi.org/10.1371/journal.pone.0260033

74. Boscarino JA, Adams RE, Wingate TJ, et al. Impact and risk of moral injury among deployed veterans: implications for veterans and mental health. Frontiers in psychiatry. 2022;13. https://doi.org/10.3389/fpsyt.2022.899084

75. Brady C, Fenton C, Loughran O, et al. Dublin hospital workers' mental health during the peak of Ireland's COVID-19 pandemic. Irish Journal of Medical Science (1971-). 2022 Jun 22:1-0. https://doi.org/10.1007/s11845-022-03056-0

For peer review only - http://bmjopen.bmj.com/site/about/guidelines.xhtml

76. Brady C, Fenton C, Loughran O, et al. Nursing home staff mental health during the Covid-19 pandemic in the Republic of Ireland. International journal of geriatric psychiatry. 2021; 37(1), 1-10. https://doi.org/10.1002/gps.5648

77. Chandrabhatla T, Asgedom H, Gaudiano ZP, de Avila L, Roach KL, Venkatesan C, Weinstein AA, Younossi ZM. Second victim experiences and moral injury as predictors of hospitalist burnout before and during the COVID-19 pandemic. Plos one. 2022 Oct 4;17(10):e0275494. https://doi.org/10.1371/journal.pone.0275494

78. Dale LP, Cuffe SP, Sambuco N, et al. Morally distressing experiences, moral injury, and burnout in Florida healthcare providers during the COVID-19 pandemic. International Journal of Environmental Research and Public Health. 2021 Nov 24;18(23):12319. https://doi.org/10.3390/ijerph182312319

79. Plouffe RA, Easterbrook B, Liu A, et al. Psychometric evaluation of the moral injury events scale in two Canadian armed forces samples. Assessment. 2023 Jan;30(1):111-23. https://doi.org/10.1177/10731911211044198

80. Nichter B, Norman SB, Maguen S, et al. Moral injury and suicidal behavior among US combat veterans: Results from the 2019–2020 National Health and Resilience in Veterans Study. Depression and anxiety. 2021 Jun;38(6):606-14. https://doi.org/10.1002/da.23145

81. Fitzpatrick JJ, Pignatiello G, Kim M, et al. Moral Injury, Nurse Well-being, and Resilience Among Nurses Practicing During the COVID-19 Pandemic. JONA: The Journal of Nursing Administration. 2022 Jul 1;52(7/8):392-8. DOI: 10.1097/NNA.0000000000001171

82. Hagerty SL, Williams LM. Moral Injury, Traumatic Stress, and Threats to Core Human Needs in Health-Care Workers: The COVID-19 Pandemic as a

For peer review only - http://bmjopen.bmj.com/site/about/guidelines.xhtml

Dehumanizing Experience. Clinical Psychological Science. 2022 Jan 10:21677026211057554. https://doi.org/10.1177/21677026211057554

83. Hamrick HC, Ehlke SJ, Davies RL, et al. Moral injury as a mediator of the associations between sexual harassment and mental health symptoms and substance use among women veterans. Journal of interpersonal violence. 2022 Jun;37(11-12):NP10007-35. https://doi.org/10.1177/0886260520985485

84. Hines SE, Chin KH, Levine AR, et al. Initiation of a survey of healthcare worker distress and moral injury at the onset of the COVID-19 surge. American journal of industrial medicine. 2020 Sep 1. DOI: 10.1002/ajim.23157

85. Hinkel HM, Currier JM, Isaak SL, et al. Moral injury and chronic pain among military veterans in an integrated behavioral health clinic. Psychological trauma: theory, research, practice, and policy. 2022 Mar 24. https://doi.org/10.1037/tra0001230

86. Kelley ML, Chae JW, Bravo AJ, et al. Own soul's warning: Moral injury, suicidal ideation, and meaning in life. Psychological trauma: theory, research, practice, and policy. 2021 Aug 5. https://doi.org/10.1037/tra0001047

87. La Fleur R, McDonald M, Senecal G, et al. The Impact of Moral Injury and Disclosure of Military Experiences on Veterans. Journal of Humanistic Psychology. 2020 Nov 20:0022167820972214. https://doi.org/10.1177/0022167820972214

88. Lamb D, Gnanapragasam S, Greenberg N, Bhundia R, et al. Psychosocial impact of the COVID-19 pandemic on 4378 UK healthcare workers and ancillary staff: initial baseline data from a cohort study collected during the first wave of the pandemic. Occupational and environmental medicine. 2021 Nov 1;78(11):801-8. http://dx.doi.org/10.1136/oemed-2020-107276

For peer review only

89. Litam SD, Balkin RS. Moral injury in health-care workers during COVID-19 pandemic. Traumatology. 2021 Mar;27(1):14. https://doi.org/10.1037/trm0000290

90. Litz BT, Plouffe RA, Nazarov A, et al. Defining and assessing the syndrome of moral injury: initial findings of the Moral Injury Outcome Scale Consortium. Frontiers in psychiatry. 2022;13. https://doi.org/10.3389/fpsyt.2022.923928

91. Maftei A, Holman AC. The prevalence of exposure to potentially morally injurious events among physicians during the COVID-19 pandemic. European Journal of Psychotraumatology. 2021 Jan 1;12(1):1898791. https://doi.org/10.1080/20008198.2021.1898791

92. Maguen S, Griffin BJ, Vogt D, et al. Moral injury and peri-and post-military suicide attempts among post-9/11 veterans. Psychological medicine. 2022 Jan 17:1-0. https://doi.org/10.1017/S0033291721005274

93. Malakoutikhah A, Zakeri MA, Koenig HG, et al. The psychometric properties of the Persian version of the moral injury symptoms scale-health care professionals version. Frontiers in psychology. 2022:4803. https://doi.org/10.3389/fpsyg.2022.978572

94. Mantri S, Lawson JM, Wang Z, et al. Identifying moral injury in healthcare professionals: The moral injury symptom scale-HP. Journal of religion and health. 2020 Oct;59(5):2323-40. https://doi.org/10.1007/s10943-020-01065-w

95. Mantri S, Song YK, Lawson JM, et al. Moral injury and burnout in health care professionals during the COVID-19 pandemic. The Journal of Nervous and Mental Disease. 2021 Oct 1;209(10):720-6. DOI: 10.1097/NMD.0000000000001367

96. Morris DJ, Webb EL, Devlin P. Moral injury in secure mental healthcare part II: experiences of potentially morally injurious events and their relationship to

For peer review only

wellbeing in health professionals in secure services. The Journal of Forensic Psychiatry & Psychology. 2022 Sep 3;33(5):726-44. https://doi.org/10.1080/14789949.2022.2111319

97. Rushton CH, Thomas TA, Antonsdottir IM, Net al. Moral injury and moral resilience in health care workers during COVID-19 pandemic. Journal of palliative medicine. 2022 May 1;25(5):712-9. https://doi.org/10.1089/jpm.2021.0076

98. Nieuwsma JA, Brancu M, Wortmann J, et al. Screening for moral injury and comparatively evaluating moral injury measures in relation to mental illness symptomatology and diagnosis. Clinical Psychology & Psychotherapy. 2021 Jan;28(1):239-50. https://doi.org/10.1002/cpp.2503

99. Osmann J, Page-Gould E, Inbar Y, et al. Validation of the Toronto Moral Injury Scale for Journalists. Traumatology. 2022 Aug 25. https://doi.org/10.1037/trm0000409

100. Rodríguez EA, Agüero-Flores M, Landa-Blanco M, et al. Moral injury and light triad traits: anxiety and depression in health-care personnel during the coronavirus-2019 pandemic in Honduras. Hispanic health care international. 2021 Dec;19(4):230-8. https://doi.org/10.1177/1540415321104237

101. Roth SL, Andrews K, Protopopescu A, et al. Development and preliminary evaluation of the moral injury assessment for public safety personnel. Traumatology. 2022 Mar 3. https://doi.org/10.1037/trm0000367

102. Russell B, Mussap AJ. Rumination and threat-biased interpretation mediate posttraumatic stress and growth responses to military stressors. Military Psychology. 2022 Oct 2:1-6. https://doi.org/10.1080/08995605.2022.2127618

For peer review only

103. Saba SK, Davis JP, Lee DS, et al. Moral injury events and behavioral health outcomes among American veterans. Journal of anxiety disorders. 2022 Aug 1;90:102605. https://doi.org/10.1016/j.janxdis.2022.102605

104. Senger AR, Torres D, Ratcliff CG. Potentially morally injurious events as a mediator of the association of gratitude and mindfulness with distress. Psychological Trauma: Theory, Research, Practice, and Policy. 2022 Mar 31. https://doi.org/10.1037/tra0001233

105. Stanojević S, Čartolovni A. Moral distress and moral injury and their interplay as a challenge for leadership and management: The case of Croatia. Journal of Nursing Management. 2022 Oct 4. https://doi.org/10.1111/jonm.13835

106. Testoni I, Brondolo E, Ronconi L, et al. Burnout following moral injury and dehumanization: A study of distress among Italian medical staff during the first COVID-19 pandemic period. Psychological Trauma: Theory, Research, Practice, and Policy. 2022 Aug 4. https://doi.org/10.1037/tra0001346

107. Thomas ED, Weiss NH, Forkus SR, et al. Examining the interaction between potentially morally injurious events and religiosity in relation to alcohol misuse among military veterans. Journal of Traumatic Stress. 2022 Feb;35(1):314-20. https://doi.org/10.1002/jts.22682

108. Trifunovic-Koenig M, Strametz R, Gerber B, Mantri S, Bushuven S. Validation of the German Version of the Moral Injury Symptom and Support Scale for Health Professionals (G-MISS-HP) and Its Correlation to the Second Victim Phenomenon. International Journal of Environmental Research and Public Health. 2022 Jan;19(8):4857. https://doi.org/10.3390/ijerph19084857

109. Ulusoy S, Çelik Z. The Silent Cry of Healthcare Workers: A Cross-Sectional Study on Levels and Determinants of Burnout among Healthcare Workers after

For peer review only - http://bmjopen.bmj.com/site/about/guidelines.xhtml

First Year of the Pandemic in Turkey. Psychiatry and Clinical Psychopharmacology. 2022 Mar 1;32(1):63-71. DOI:10.5152/pcp.2022.21248

110. Williamson V, Murphy D, Greenberg N. Experiences and impact of moral injury in UK veterinary professional wellbeing. European journal of psychotraumatology. 2022 Dec 31;13(1):2051351. https://doi.org/10.1080/20008198.2022.2051351

111. Zahiriharsini A, Gilbert-Ouimet M, Langlois L, Biron C, Pelletier J, Beaulieu M, Truchon M. Associations between psychosocial stressors at work and moral injury in frontline healthcare workers and leaders facing the COVID-19 pandemic in Quebec, Canada: A cross-sectional study. Journal of Psychiatric Research. 2022 Nov 1;155:269-78. https://doi.org/10.1016/j.jpsychires.2022.09.006

112. Levi-Belz Y, Shemesh S, Zerach G. Moral injury and suicide ideation among combat veterans: The moderating role of self-disclosure. Crisis: The Journal of Crisis Intervention and Suicide Prevention. 2022 Feb 9. https://doi.org/10.1027/0227-5910/a000849

113. Schwartz G, Halperin E, Levi-Belz Y. Moral injury and suicide ideation among combat veterans: The role of trauma-related shame and collective hatred. Journal of interpersonal violence. 2022 Aug;37(15-16):NP13952-77. DOI: 10.1177/08862605211007932

114. Currier JM, Isaak SL, McDermott RC. Validation of the Expressions of Moral Injury Scale-Military version-short form. Clinical psychology & psychotherapy. 2020 Jan;27(1):61-8. https://doi.org/10.1002/cpp.2407

115. Nash WP, Marino Carper TL, Mills MA, Au T, Goldsmith A, Litz BT. Psychometric evaluation of the moral injury events scale. Military medicine. 2013 Jun 1;178(6):646-52. https://doi.org/10.7205/MILMED-D-13-00017

For peer review only - http://bmjopen.bmj.com/site/about/guidelines.xhtml

116. Hall NA, Everson AT, Billingsley MR, Miller MB. Moral injury, mental health and behavioural health outcomes: A systematic review of the literature. Clinical psychology & psychotherapy. 2022 Jan;29(1):92-110. https://doi.org/10.1002/cpp.2607

117. McEwen C, Alisic E, Jobson L. Moral injury and mental health: A systematic review and meta-analysis. Traumatology. 2021 Sep;27(3):303. https://doi.org/10.1037/trm0000287

118. Cumpston M, Li T, Page MJ, Chandler J, Welch VA, Higgins JP, Thomas J. Updated guidance for trusted systematic reviews: a new edition of the Cochrane Handbook for Systematic Reviews of Interventions. The Cochrane database of systematic reviews. 2019(10).

119. Migliavaca CB, Stein C, Colpani V, Barker TH, Ziegelmann PK, Munn Z, Falavigna M, Prevalence Estimates Reviews—Systematic Review Methodology Group (PERSyst). Meta-analysis of prevalence: I 2 statistic and how to deal with heterogeneity. Research synthesis methods. 2022 May;13(3):363-7. https://doi.org/10.1002/jrsm.1547

120. French L, Hanna P, Huckle C. “If I die, they do not care”: UK National Health Service staff experiences of betrayal-based moral injury during COVID-19. Psychological Trauma: Theory, Research, Practice, and Policy. 2022 Mar;14(3):516. https://doi.org/10.1037/tra0001134

121. Hegarty S, Lamb D, Stevelink SA, Bhundia R, Raine R, Doherty MJ, Scott HR, Marie Rafferty A, Williamson V, Dorrington S, Hotopf M. ‘It hurts your heart’: frontline healthcare worker experiences of moral injury during the COVID-19 pandemic. European journal of psychotraumatology. 2022 Dec 19;13(2):2128028. https://doi.org/10.1080/20008066.2022.2128028

For peer review only - http://bmjopen.bmj.com/site/about/guidelines.xhtml

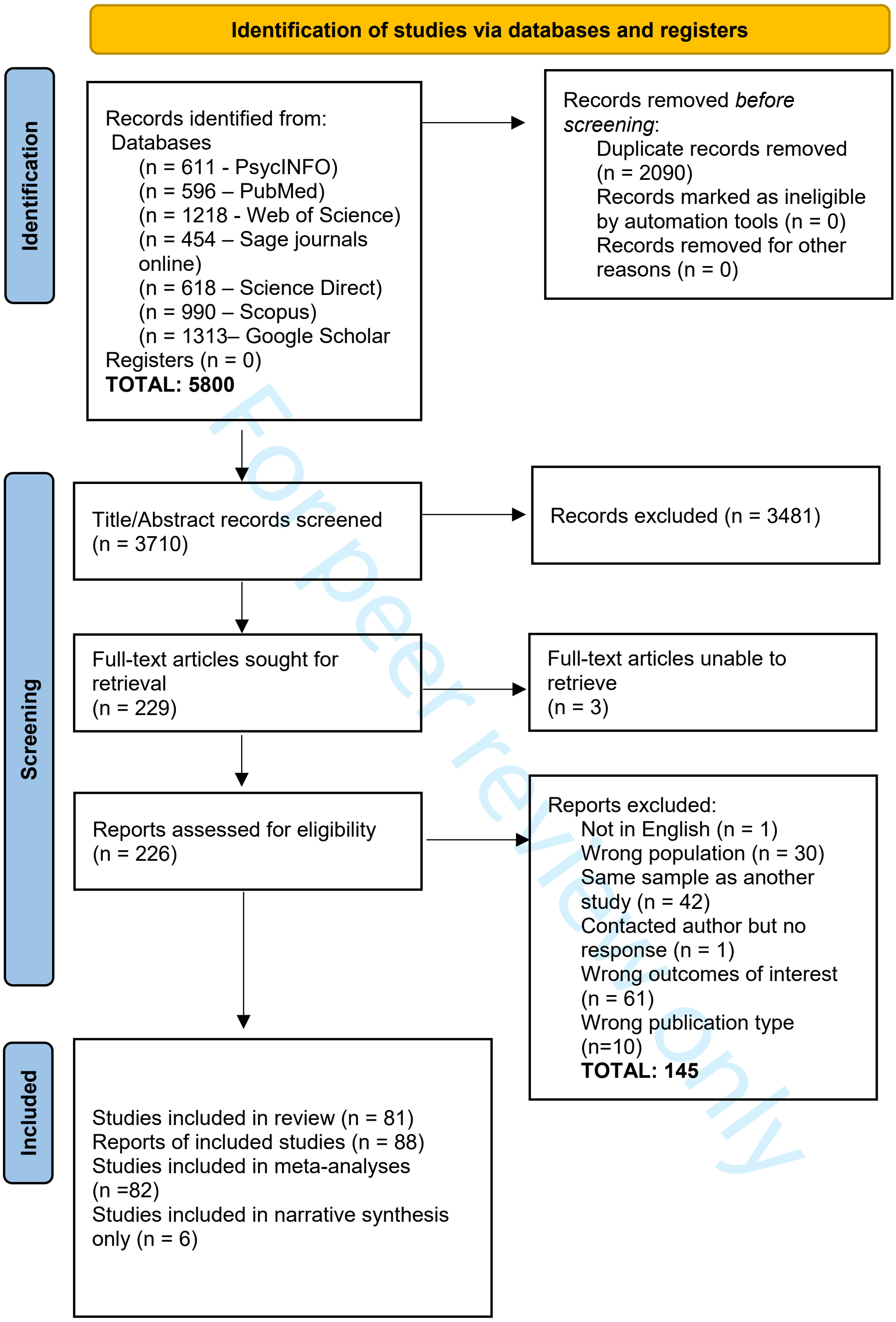

For peer review only

Supplementary materials

Supplementary Table 1.
Search strategy

Search date: 22 June 2020 and updated 2 November 2022

Moral injury search strategy

APA PsycINFO (EBSCOhost interface)

((“moral injur*” OR “transgressive act*” OR “moral transgression”)) AND ((Veteran* OR military OR army OR marine* OR "national service" OR "territorial army" OR "armed forces" OR "special forces" OR RAF OR "air force" OR navy OR "service personnel" OR "ex-service personnel" OR submariner* OR sailor* OR seamen OR seaman OR airmen OR airman OR soldier* OR troop* OR "enlisted personnel" OR "armed forces" OR "armed services" OR police* OR fireman OR firemen OR firefighter* OR teacher* OR "humanitarian aid worker" OR "relief aid worker" OR "social worker" OR "disaster responder" OR policeman OR policemen OR policewoman OR "law enforcement" OR healthcare OR nurse OR doctor OR frontline OR "health professional" OR "hospital staff" OR "hospital worker" OR work OR occupation OR job OR "emergency service"))). Filters Published: 2009-2022; Expanders - Apply equivalent subjects; Narrow by Language: - English, Search modes - Find all my search terms.

PubMed

("moral injur*" OR "morally injurious" OR PMIE* OR "potentially morally injurious" OR "moral transgression" OR "transgressive act*") AND (Veteran* OR military OR army OR marine* OR "national service" OR "territorial army" OR "armed forces" OR "special forces" OR RAF OR "air force" OR navy OR "service personnel" OR "ex-service personnel" OR submariner* OR sailor* OR seamen OR seaman OR airmen OR airman OR soldier* OR troop* OR "enlisted personnel" OR "armed forces" OR "armed services" OR police* OR fireman OR firemen OR firefighter* OR teacher* OR "humanitarian aid worker" OR "relief aid worker" OR "social worker" OR "disaster responder" OR policeman OR policemen OR policewoman OR "law enforcement" OR healthcare OR nurse OR doctor OR frontline OR "health professional" OR "hospital staff" OR "hospital worker" OR work OR job OR occupation OR "emergency service*") Filters: English; Published 2009-2022; all fields.

Web of Science Core Collection

For peer review only - http://bmjopen.bmj.com/site/about/guidelines.xhtml

(TS=((mora* injur* OR moral transgression*) AND (Veteran* OR military OR army OR marine* OR "national service" OR "territorial army" OR "armed forces" OR "special forces" OR RAF OR "air force" OR navy OR "service personnel" OR "ex-service personnel" OR submariner* OR sailor* OR seamen OR seaman OR airmen OR airman OR soldier* OR troop* OR "enlisted personnel" OR "armed forces" OR "armed services" OR police* OR fireman OR firemen OR firefighter* OR teacher* OR "humanitarian aid worker" OR "relief aid worker" OR "social worker" OR "disaster responder" OR policeman OR policemen OR policewoman OR "law enforcement" OR healthcare OR nurse OR doctor OR frontline OR "health professional" OR "hospital staff" OR "hospital worker" OR work OR job OR "emergency service" OR occupation)) and Article (Document Types) and English (Languages) and 2009-2022 (Publication Years)

Sage Journals Online

[[All "moral injur*"] OR [All "transgressive act*"] OR [All "moral transgression*"]] AND [[All veteran*] OR [All military] OR [All army] OR [All marine*] OR [All "national service"] OR [All "territorial army"] OR [All "armed forces"] OR [All "special forces"] OR [All raf] OR [All "air force"] OR [All navy] OR [All "service personnel"] OR [All "ex-service personnel"] OR [All submariner*] OR [All sailor*] OR [All seamen] OR [All seaman] OR [All airmen] OR [All airman] OR [All soldier*] OR [All troop*] OR [All "enlisted personnel"] OR [All "armed forces"] OR [All "armed services"] OR [All police*] OR [All fireman] OR [All firemen] OR [All firefighter*] OR [All teacher*] OR [All "humanitarian aid worker"] OR [All "relief aid worker"] OR [All "social worker"] OR [All "disaster responder"] OR [All policeman] OR [All policemen] OR [All policewoman] OR [All "law enforcement"] OR [All healthcare] OR [All nurse] OR [All doctor] OR [All frontline] OR [All "health professional"] OR [All "hospital staff"] OR [All "hospital worker"] OR [All work] OR [All occupation] OR [All "emergency service*"] OR [All job]]
Filters: Research articles, Published 2009-2022.

ScienceDirect

all = ("moral injury" OR "morally injurious" OR "moral transgression" OR "transgressive act") AND (veteran OR police OR job OR healthcare OR occupation). Filters: Research articles; Published 2009-2022.

Scopus

TITLE-ABS-KEY (("mora*injur*" OR "transgression") AND (veteran* OR police OR military OR "law enforcement" OR healthcare OR occupation)). Filters: DOCTYPE “ar”; Published 2009-2022; Language "English".

For peer review only - http://bmjopen.bmj.com/site/about/guidelines.xhtml

Google Scholar

All in the title: "moral injury" . Filters: = Published 2009-2022; Language "English".

PTED search strategy

APA PsycINFO (EBSCOhost interface)

((PTED or “post traumatic embitterment disorder” OR embittered OR embitterment)) AND ((Veteran* OR military OR army OR marine* OR "national service" OR "territorial army" OR "armed forces" OR "special forces" OR RAF OR "air force" OR navy OR "service personnel" OR "ex-service personnel" OR submariner* OR sailor* OR seamen OR seaman OR airmen OR airman OR soldier* OR troop* OR "enlisted personnel" OR "armed forces" OR "armed services" OR police* OR fireman OR firemen OR firefighter* OR teacher* OR "humanitarian aid worker" OR "relief aid worker" OR "social worker" OR "disaster responder" OR policeman OR policemen OR policewoman OR "law enforcement" OR healthcare OR nurse OR doctor OR frontline OR "health professional" OR "hospital staff" OR "hospital worker" OR work OR occupation OR job OR emergency service*)).
Filters - Published: 2003-2022; Expanders - Apply equivalent subjects; Narrow by Language: - English’; Exclude Dissertations; Search modes - Find all my search terms.

PubMed

(“post traumatic embitterment disorder” OR PTED OR embittered OR embitterment) AND (Veteran* OR military OR army OR marine* OR "national service" OR "territorial army" OR "armed forces" OR "special forces" OR RAF OR "air force" OR navy OR "service personnel" OR "ex-service personnel" OR submariner* OR sailor* OR seamen OR seaman OR airmen OR airman OR soldier* OR troop* OR "enlisted personnel" OR "armed forces" OR "armed services" OR police* OR fireman OR firemen OR firefighter* OR teacher* OR "humanitarian aid worker" OR "relief aid worker" OR "social worker" OR "disaster responder" OR policeman OR policemen OR policewoman OR "law enforcement" OR healthcare OR nurse OR doctor OR frontline OR "health professional" OR "hospital staff" OR "hospital worker" OR work OR job OR occupation OR "emergency service"). Filters: English; Published 2009-2022; all fields.

Web of Science Core Collection

For peer review only - http://bmjopen.bmj.com/site/about/guidelines.xhtml

(TS=("post traumatic embitterment disorder" OR PTED OR embittered OR embitterment) AND TS=(Veteran* OR military OR army OR marine* OR "national service" OR "territorial army" OR "armed forces" OR "special forces" OR RAF OR "air force" OR navy OR "service personnel" OR "ex-service personnel" OR submariner* OR sailor* OR seamen OR seaman OR airmen OR airman OR soldier* OR troop* OR "enlisted personnel" OR "armed forces" OR "armed services" OR police* OR fireman OR firemen OR firefighter* OR teacher* OR "humanitarian aid worker" OR "relief aid worker" OR "social worker" OR "disaster responder" OR policeman OR policemen OR policewomen OR "law enforcement" OR healthcare OR nurse OR doctor OR frontline OR "health professional" OR "hospital staff" OR "hospital worker" OR work OR occupation OR job OR emergency service*)) and Article (Document Types) and English (Languages) and 2003-2022 (Publication Years)

Sage Journals Online

[[All "post traumatic embitterment disorder"] OR [All pted]] OR [All "chronic embitterment"]] AND [[All veteran*] OR [All military] OR [All army] OR [All marine*] OR [All "national service"] OR [All "territorial army"] OR [All "armed forces"] OR [All "special forces"] OR [All raf] OR [All "air force"] OR [All navy] OR [All "service personnel"] OR [All "ex-service personnel"] OR [All submariner*] OR [All sailor*] OR [All seamen] OR [All seaman] OR [All airmen] OR [All airman] OR [All soldier*] OR [All troop*] OR [All "enlisted personnel"] OR [All "armed forces"] OR [All "armed services"] OR [All police*] OR [All fireman] OR [All firemen] OR [All firefighter*] OR [All teacher*] OR [All "humanitarian aid worker"] OR [All "relief aid worker"] OR [All "social worker"] OR [All "disaster responder"] OR [All policeman] OR [All policemen] OR [All policewoman] OR [All "law enforcement"] OR [All healthcare] OR [All nurse] OR [All doctor] OR [All frontline] OR [All "health professional"] OR [All "hospital staff"] OR [All "hospital worker"] OR [All work] OR [All occupation] OR [All job] OR [All "emergency service*"]]. Filters: Research articles, Published 2003-2022.

ScienceDirect

("post traumatic embitterment disorder" OR pted OR embitterment OR embittered) AND (veteran OR police OR "law enforcement" OR healthcare OR occupation). Filters: Research articles; Published 2003-2022.

Scopus

ALL ((pted OR "post traumatic embitterment disorder" OR embittered OR embitterment) AND (veteran* OR police OR military OR job OR healthcare OR work OR occupatio n)). Filters: DOCTYPE “ar”; Published 2003-2022; Language "English".

For peer review only - http://bmjopen.bmj.com/site/about/guidelines.xhtml

**Google Scholar**
All: pted OR embitterment OR "post traumatic embitterment disorder". Filters: = Published 2009-2022; Language "English".

For peer review only

For peer review only - http://bmjopen.bmj.com/site/about/guidelines.xhtml

Supplementary Table 2.

Data extraction table for studies included in the systematic review

Author (Year)	Study Design	N_a	Occupation	Sampling method	Mean age	% Male	% White	Location	RR	Measurement tool	Associations (+) significant positive correlation (-) significant negative correlation (0) not significant	Prevalence N (%)	Total M(SD)	Hoy	Conflict of Interest
PTED only studies															
Dunn & Sensky. 2018	Cross-sectional	79	Healthcare NHS staff.	Convenience Occupational health department October 2015-March 2016.	44 (12)	33%	NR	UK	NR	PTED scale 1.6+ and 2+	(Mental) health<br>Depression (HADS) (+)<br>Individual and/or work characteristics<br>Affective rumination (WRRQ) (+)<br>Detachment (WRRQ) (-)<br>Problem solving pondering (WRRQ) (0)<br>Positive beliefs about rumination (PBRS) (0)<br>Sickness absence (0)<br>Demographics<br>Gender (0)	1.6 cut off: 37 (47%)<br>2 cut off: 28(35%)	1.5 (1.1)	5	NR
Linden & Rotter 2019	Cross-sectional	102	Unemployed Duration > 1 year (32.4%)	Convenience	36.6 (12.1)	59%	NR	Germany	NR	PTED scale 2+	(Mental) health<br>Psychological distress (GHQ-28) (+)<br><br>Individual and/or work characteristics<br>Feelings of injustice (+)<br>Worse appraisal of unemployment (+)<br>Duration of unemployment (+)<br>Demographics<br>Gender (0)<br>Age (+)	Cut off 2: 26 (25.5%)	1.24 (0.92)	5	None

For peer review only - http://bmjopen.bmj.com/site/about/guidelines.xhtml

Michailidis & Cropley. 2018	Longitudinal (T1)	352	Multiple.	Convenience	NR	NR	NR	Cyprus	NR	PTED scale 1.6+	(Mental) health Negative affect (+) Individual and/or work characteristics Organisational procedural justice (OJP) (-) Organisational distributive justice (OJP) (-) Organisational interpersonal justice (OJP) (-) Organisational informational justice (OJP) (-) Supervisory control (+) Job demands (0)	Cut off 1.6: 55 (35%)	1.28 (0.92)	5	None
Sensky, Salimu, Ballard & Pereira. 2015	Cross sectional	236	NHS staff, primarily nurses (N = 67, 28%) and healthcare assistants (n = 49, 21%). 45% worked for the trust for <5 years.	Convenience September-November 2009 Represents sample frame.	NR	45%	NR	UK.	NR	PTED scale 1.6+	Individual and/or work characteristics Work attitudes - felt obligation (0) Work attitudes - organisational support (-) Work attitudes - employer-employee reciprocity (0) Work attitudes - work effort (0) Work attitudes - procedural justice (-) Management referral to occupational health (+) Certified sickness absence (+)	Cut off 1.6: 68 (28.81%) Cut off 2: 43 (18.2%) Cut off 2.5: 21 (8.9%)	1.11(0.97)	4	None

For peer review only - http://bmjopen.bmj.com/site/about/guidelines.xhtml
For peer review only

											Uncertified sickness absence (+)<br>Work stress - demands (HSE) (0)<br>Work stress - control (HSE) (0)<br>Work stress - management support (HSE) (-)<br>Work stress - peer support (HSE) (-)<br>Work stress – relationships (HSE) (0)<br>Work stress - role (HSE) (-)<br>Work stress – change (HSE) (-)<br>Demographics:<br>Gender (0)<br>Age (0)<br>Years since qualifying (0)<br>More years in present job (+)				
Sabic, Sabic, & Baltic-Mujanovic, 2018	Cross sectional	87	War veterans of the war in Bosnia and Herzegovnia (1992-1995). Average time since they were a soldier M = 23.86 years.	Convenience	51.42 (5.98)	100%	NR	Bosnia and Herzegovina	NR	PTED scale 1.6+, 2+ and 2.5+	NR	Cut off 1.6: 15 (17.24%)<br>Cut off 2: 9 (10.34%)<br>Cut off 2.5: 1 (1.15%)	0.75(0.73)	5	None

For peer review only

Michailidis & Cropley. 2017	Cross sectional	337	Multiple	Convenience	36.87 (12.4)	26.1 %	NR	NR	NR	PTED scale 1.6+, 2+ and 2.5+	(Mental) health<br>Negative affect (PANAS) (+)<br>Positive affect (PANAS) (-)<br><br>Individual and/or work characteristics<br>Affective rumination (WRRQ) (+)<br>Problem solving pondering (WRRQ) (+)<br>Detachment (WRRQ) (-)<br>Job demands (JCQ) (0)<br>Workplace social support (-)<br>Organisational procedural justice (OJP) (-)<br>Organisational distributive justice (OJP) (-)<br>Organisational interpersonal justice (OJP) (-)<br>Organisational informational justice (OJP) (-)<br>Supervisory control (WCOWP) (+)<br><br>Demographics<br>Gender (+)<br>Age (0)<br>Dependent children (0)	Cut off 1.6: 253 (75%)<br>Cut off 2: 209 (62%)<br>Cut off 2.5: 152 (45%)	2.44(.96)	5	None

For peer review only

											Hours worked per week (+)<br>Years in current job (+)				
Karatuna & Gok 2014	Cross-sectional	397	Public sector, social security institutions Non-managerial position = 83.1%	Convenience	NR	57.2 %		Turkey	39.1 1%	PTED scale 1.6+, 2+, 2.5+	Individual and/or work characteristics<br>Workplace bulling (NAQ-R) (+)<br>Victimisation from bullying (+)<br>Victims of bulling (+)	Cut off 1.6: 106 (26.7%)<br>Cut off 2: 74 (18.6%)<br>Cut off 2.5: 41 (10.3%)	1.00(1.04)	5	NR
Rubab & Tariq 2022	Cross-sectional	300	Private school teachers.	Purposive.	31 (7.9)	50%	NR	NR	NR	PTED scale 1.6+, 2+, 2.5+	(Mental) health<br>Depression (DASS-21) (+)<br>Stress (DASS-21) (+)<br>Anxiety (DASS-21) (+)<br>Depression, stress, and anxiety total (DASS-21) (+)<br><br>Individual and/or work characteristics<br>Generalised self-efficacy (GSES) (-)	Cut off 1.6: 121 (40.3%)<br>Cut off 2: 77 (25.7%)<br>Cut off 2.5: 26 (8.7%)	1.44 (0.70)	5	NR
Saleem, Bashir & Abrar, 2022	Longitudinal (T2)	398	Public sector university academics.	Simple random sampling.	44.5	64%	NR	Pakistan	73 %	PTED scale (mean only)	(Mental) health<br>Employee well-being (-)<br>Individual and/or work characteristics	NR	3.16 (1.12)	5	None

For peer review only - http://bmjopen.bmj.com/site/about/guidelines.xhtml

											Leaders' ethical behaviours (-)<br>Employee core self-evaluation (-)				
Shin & You 2022	Cross-sectional	1074	Unemployed.	Convenience	47.1 (13.6)	47.6 %	NR	South Korea	NR	PTED scale 1.6+, >1.6 ≥ 2, 2.5+	Individual and/or work characteristics<br>Work types (0)<br>Unemployment duration (0)<br>Unemployment route (0)<br>Negative life events (+)<br>General belief in a just world (BJW) (-)<br>Personal BJW (-)<br>Demographics<br>Gender (0)<br>Age (+)<br>Marital status (divorced/separated) (+)<br>Educational level (-)<br>Income (-)	Cut off 1.6: 581 (54.1%)<br>Cut off 1.6 - 2.5: 479 (44.6%)<br>Cut off 2.5: 102 (9.5%)	1.64 (0.70)	5	None
PTED and moral injury study															
Brennan, McKay & Cole, 2022	Longitudinal (T1)	400	Health and social care<br>Allied health (14.5%)<br>Primary care practitioner (15.8%)<br>Managerial (10.3%)<br>Health info/science (9.3%)	Convenience September-October 2020.	36.84 (10.7)	24.5 %	63%	UK	89.5 %	MIES 4+ (slightly agree)<br>3 subscales (transgressions-others, transgressions-self, betrayal)<br><br>PTED 1.6+ and 2+ and 2.5	Transgressions-self (Mental) health:<br>Current mental health diagnoses (0)<br>Individual and/or work characteristics<br>Exposure to occupational stressors (+)<br>Pessimism (LOTR) (+)<br>Consideration of immediate	MIES:<br>Overall %<br>289 (72.3%)<br>Per subscale %<br>Transgressions-others: 213 (53.3%)<br><br>Transgressions-self: 131 (32.8%)<br>Betrayal: 227 (56.8%)	MIES:<br>Overall M/SD<br>2.55(1.13)<br>Subscale M/SD<br>Transgressions-others: 2.98(1.48)<br><br>Transgressions-self: 2.17(1.22)<br>Betrayal: 2.77(1.38)	2	ESRC and University funding

For peer review only

	Psychological (6.8%)<br>Corporate (16.3%)<br>Clinical support (26.3%)		consequences (CFC-14) (+)<br>Consideration of future consequences (CFC-14) (0)<br>Resilient coping style (BRCS) (0)<br>Self-esteem (RSES) (-)<br>Procedural justice (BJW) (-)<br>Distributive justice (BJW) (0)<br>Social desirability (-)<br>Demographics *<br>Age (0)<br>Gender (0)<br>Professional role (0)<br>Ethnicity (0)<br>Years in current role (0)<br>Transgressions-others (Mental) health:<br>Current mental health diagnoses (0)<br>Individual and/or work characteristics<br>Exposure to occupational stressors (+)<br>Pessimism (LOTR) (+)<br>Consideration of immediate consequences (CFC-14) (0)<br>Consideration of future consequences (CFC-14) (0)	MIES per item %:<br>Item 1: 179 (44.8%)<br>Item 2: 167 (41.8%)<br>Item 3: 76 (19%)<br>Item 4: 94 (23.5%)<br>Item 5: 83 (20.8%)<br>Item 6: 78 (19.5%)<br>Item 7: 161 (40.3%)<br>Item 8: 124 (31%)<br>Item 9: 158 (39.5%)<br>PTED overall %:<br>1.6 cut off: 121 (30.3%)<br>2 cut off: 77 (19.3%)<br>2.5 cut off: 30 (7.5%)	PTED:<br>Overall M/SD<br>1.12(0.90)

For peer review only

For peer review only - http://bmjopen.bmj.com/site/about/guidelines.xhtml

Resilient coping style (BRCS) (+)
Self-esteem (RSES) (-)
Procedural justice (BJW) (-)
Distributive justice (BJW) (0)
Social desirability (-)
Betrayal:
Mental health:
Current mental health diagnoses (+)
Individual and/or work characteristics
Exposure to occupational stressors (+)
Pessimism (LOTR) (+)
Consideration of immediate consequences (CFC-14) (+)
Consideration of future consequences (CFC-14) (0)
Resilient coping style (BRCS) (0)
Self-esteem (RSES) (-)
Procedural justice (BJW) (-)
Distributive justice (BJW) (-)
Optimism (LOTR) (-)
Social desirability (-)
PTED:
Mental health:

For peer review only

For peer review only - http://bmjopen.bmj.com/site/about/guidelines.xhtml

	Current mental health diagnoses (+)<br>Individual and/or work characteristics<br>Exposure to occupational stressors (+)<br>Pessimism (LOTR) (+)<br>Consideration of immediate consequences (CFC-14) (+)<br>Consideration of future consequences (CFC-14) (0)<br>Resilient coping style (BRCS) (0)<br>Self-esteem (R-SES) (-)<br>Procedural justice (BJW) (-)<br>Distributive justice (BJW) (-)<br>Optimism (LOTR) (-)<br>Social desirability (-)<br>Demographics<br>Age (+)<br>*PTED and all MIES subscales
Moral injury only studies	

For peer review only - http://bmjopen.bmj.com/site/about/guidelines.xhtml

Bryan et al., 2016<br><br>Sample 2	Cross-sectional	935	Serving military personnel. Army national guard (84%).	Convenience	27.05 (8.11)	82.3 %	57.4%	USA	NR	MIES 6+ (strongly agree)<br>3 subscales (transgressions-others, transgressions-self, betrayal)	Individual and/or work characteristics<br>Transgressions-others subscale:<br>Pre-deployment stressors (DDRI) (+)<br>Combat experiences (DDRI) (+)<br>Aftermath of battle (DDRI) (+)<br>Transgressions-self subscale:<br>Pre-deployment stressors (DDRI) (0)<br>Combat experiences (DDRI) (0)<br>Aftermath of battle (DDRI) (0)<br>Betrayal subscale:<br>Pre-deployment stressors (DDRI) (+)<br>Combat experiences (DDRI) (0)<br>Aftermath of battle (DDRI) (0)<br>Demographics<br>Gender (0)	Overall %<br>NR<br>Per subscale %<br>Cut off 6<br>Transgressions-others: 363 (38.8%)<br>Transgressions-self: 508 (54.3%)<br>Betrayal: 540 (47.8%)	Overall M/SD<br>NR<br>Subscale M/SD<br>Transgressions-others: 4.32(1.67)<br>Transgressions-self: 4.92(1.51)<br>Betrayal: 4.63(1.63)	5	None
Currier, Holland, Drescher et al., 2015<br><br>Sample 1	Cross-sectional	131	Veterans. Deployed to Iraq and/or Afghanistan<br>Marine Corps (38.32%), Active-Duty Army (35.1%)	Convenience	28.47 (5.87)	88%<br><br>Consistent with military demographic trends	26%<br><br>Consistent with ethnic diversity of the region of study	USA	NR	MIQ-M<br>20 items<br>Unidimensional.<br>Average 3.02 years since event (returning from war zone).	(Mental) health<br>Suicide risk (SQB-R) (0)<br>PTSD (PCL-C) (+)<br>Depression (PHQ-9) (+)<br>Individual and/or work characteristics:<br>Combat exposure (CES) (+)	Overall<br>NR<br>Per subscale<br>NR	Overall M/SD<br>32.4(12.8)<br>Subscale M/SD<br>NR	3	NR

For peer review only

			and Navy (22.9%).								Work and social adjustment (WSAS) (+)<br>Active duty and marine's vs other service branches (+)<br>Deployments (+)<br>Demographics<br>Older age (+)<br>Gender (0)<br>Ethnicity (0)				
Currier, Holland, Rojas-Flores et al., 2015	Cross sectional.	257	Teachers. Classroom teachers (83.7%). 13.7% administrators e.g., principal (13.7%), and both classroom teacher and school administrator (2.7%) Average time in role 15.44 (10.29).	Convenience March 2012	42.02 (13.12)	31.4 %	NR	El Salvador	95 %	MIQ-T 12 items Unidimensional.	(Mental) health<br>PTSD Reexperiencing (LASC) (+)<br>PTSD Avoidance (LASC) (+)<br>PTSD Hyperarousal (LASC) (+)<br>Physical burnout (SMBM) (+)<br>Cognitive burnout (+)<br>Emotional burnout (+)<br>Individual and/or characteristics<br>Violence exposure (SECV) (+)<br>Meaning made of stress comprehensibility (ISLES) (-)<br>Meaning made of stress footing in the world (ISLES) (-)	Overall NR<br>Per subscale NR	Overall M/SD 13.92(6.71)<br>No subscales	4	NR

For peer review only

Currier et al., 2017<br><br>Sample 2	Cross-sectional	624	Veterans. 1+ deployments to a war zone during their service.<br><br>49.2% (Army), 10.6% (Marine Corps), 22.1% (Navy), 21.8% (Air Force), 0.8% (Coast Guard).<br><br>97.4% (Active Duty), 11.9% (Military Reserves), 6.3% (National Guard	Convenience	NR	81.4 %	10.9%	USA	7.3 %	EMIS<br>2 subscales (self-directed MI and other-directed MI).<br>MIQ-M<br>7 items<br>1 subscale (causes)<br>MIES<br>2 subscales (perpetration and betrayal)	(Mental) health<br>PTSD (PCL 5) (+)<br>Depression (PHQ-8) (+)<br>Alcohol misuse (AUDIT-C) (+)<br>Anger (DAR-5) (-)<br>Guilt (PFQ-2) (+)<br>Shame (PFQ-2) (+)<br>Individual and/or work characteristics<br>Combat exposure – Survival threat (DDRI) (+)<br>Combat exposure – Aftermath of violence (DDRI) (+)	Overall NR<br>Per subscale NR	EMIS:<br>Overall M/SD 32.14(14.84)<br>Subscale M/SD<br>Self-MI 14.92(7.49)<br>Other-MI 17.22(8.23)<br>MIQ-M<br>Overall M/SD NR<br>Subscale causes M/SD 9.29(3.41)<br>MIES<br>Overall M/SD NR<br>Subscales<br>Perpetration 2.68(1.50)<br>Betrayal 2.80(1.66)	5	NR

For peer review only

For peer review only - http://bmjopen.bmj.com/site/about/guidelines.xhtml

Forkus, Juliana & Weiss. 2019	Cross sectional	203	Veterans. Deployed to Iraq or Afghanistan. Army (52.20%), Air Force (19.20%), Navy (15.80%), and Marine Corps (12.80%).	Convenience	35.08	77.30 %	70.40%	NR	29.17%	MIES Unidimensional	(Mental) health<br>PTSD (PCL-5) (+)<br>Depression (PHQ) (+)<br>Alcohol misuse (+)<br>Drug misuse (+)<br>Deliberate self-harm history (+)<br>Deliberate self-harm versatility (+)<br>Individual and/or work characteristics<br>Self-compassion (-)	NR	Overall M/SD<br>2.95(1.41)	5	NR
Frankfurt et al., 2018.	Longitudinal secondary data analysis. MIES reported at T2	310	Veterans<br><br>Post 9/11.<br><br>Army (90.3%). Active duty (96.8%).<br><br>Average years in role 13.50 (7.61)	Convenience	40.67 (8.55) .	76%	57%<br><br>Reflected geographic area	USA	NR	MIES<br>2 subscales (perpetration and betrayal)	(Mental) health<br>Military sexual trauma (DDRI) (+) for perpetration and betrayal subscales<br>Depression (+) for perpetration and betrayal subscales<br>PTSD (+) for perpetration and betrayal subscales<br>Shame (+) for perpetration and betrayal subscales<br>Guilt (+) for perpetration and betrayal subscales<br>Individual and/or work characteristics<br>Combat exposure (0)*<br>Combat exposure (+)**<br>*betrayal<br>**perpetration	NR	Overall M/SD<br>NR<br>Subscales<br>Perpetration<br>3.00(1.40)<br>Betrayal<br>3.41(1.64)	4	None

For peer review only

Griffin et al., 2020<br><br>Narrative synthesis only	Cross sectional.	498	Military service members and veterans<br><br>Army (22.49%), Navy (17.47%), Air Force (45.78%), Marines (11.24%), Coastguard (2.61%). Active Duty (29.52%).	Convenience	NR	73.90 %	NR	USA	NR	MIES<br>5 of 9 items to create 5 profiles	NR due to using 'profiles'	Overall %<br>NR<br>Subscales<br>No moral distress (42% of sample)<br>Moral Distress-Other (19%)<br>Witnessing-Only (16%)<br>Moral distress-Self (8%)<br>Moral distress-Self and Other (15%)	NR	5	NR
Jinkerson Battles. 2019.	Cross sectional	72	Veterans Vietnam and post-Vietnam eras.<br><br>Army (59.7%), Navy (13.9%), Air Force (12.5%), Marine Corps (12.5%)	Convenience	NR	87.5 %	45.8%	USA	76 %	MIQ-M<br>20 items<br>Unidimensional	(Mental) health<br>Meaning in life (-)<br>Depression (PHQ-2) (+)<br>Anxiety (GAD-2) (+)<br>PTSD (PCL-5) (+)<br>PTSD Reexperiencing (PCL-5) (+)<br>PTSD avoidance (PCL-5) (+)<br>PTSD Negative emotions (PCL-5) (+)<br>PTSD hyperarousal/hypervigilance (PCL-5) (+)<br>Global guilt (TRGI) (+)<br>Distress (TRGI) (+)	Per item %<br>(top 5 items)<br>Betrayal by military/political leaders N = 32 (44.5%)<br>experiencing events that were chaotic and beyond control N = 32 (44.4%)<br>failing to save the life of someone in war N = 24 (33.3%)<br>surviving when others	Overall M/SD<br>3.48(0.35)<br>Subscales<br>NR	4	NR

For peer review only

For peer review only - http://bmjopen.bmj.com/site/about/guidelines.xhtml

											Guilty cognitions (TRGI) (+)<br>Wrongdoing (TRGI) (+)<br>Lack of justification (TRGI) (0)	didn't N = 21 (29.2%)<br>feeling betrayal by trusted civilians N = 32 (29.2%)			
Jordan et al., 2017.	Longitudinal. Data from 1 month post deployment reported here and from cohort 4 from the parent study.	867	Currently serving active-duty marines from a single infantry battalion engaged in heavy ground combat while deployed to Afghanistan.	Convenience	NR	NR	NR	USA	NR	MIES 4+ (slightly agree)<br>Subscales: 2 subscales (transgressions-self and betrayal)	(Mental) health<br>PTSD (PLC-S) (+)<br>Guilt/shame (PANAS) (+) *<br>Anger (PANAS) (+) *<br>Individual and/or characteristics<br>Dissociation (PDEQ) (+) *<br>Combat exposure (CES) (+) *<br>* transgressions-self and betrayal subscales	Overall %<br>NR<br>Per subscale<br>Transgressions -self 214 (24.1%)<br>Betrayal 252 (28.4%)<br>Transgressions -self and betrayal: 333 (37.5%)<br>Per item %<br>Item 1: 165 (18.6%)<br>Item 2: 89 (10%)<br>Item 3: 128 (14.4%)<br>Item 4: 103 (11.6%)<br>Item 7: 179 (20.2%)<br>Item 8: 133 (15%)<br>Item 9: 132 (14.9%)	Overall M/SD<br>NR, only used below subscales<br>Subscales<br><br>Transgressions -self = 1.87(1.17)<br><br>Betrayal = 2(1.23)	5	NR

For peer review only - http://bmjopen.bmj.com/site/about/guidelines.xhtml

Lee Aldwin, Kang. 2022	Cross sectional.	367<br><br>354 responded to MIES	Veterans<br><br>Deployed to the Vietnam war in 1664-1973.<br><br>Army (88%).	Convenience	72 (2.66)	100%	NR	Korea	37.4 %	MIES 3+ (sometimes) and 4+ (often)<br>5-point scale not 6 (1=never, 2=seldom, 3=sometimes, 4=often, 5=always).<br><br>3 subscales (transgressions-others, transgressions-self, betrayal)<br><br>40-50 years since event	(Mental) health<br>PTSD (Checklist for DSM-5) (+)<br>Depression (BSI) (+)<br>Anxiety (BSI) (+)<br><br>Individual and/or work characteristics<br>Optimism (LOT) (-)<br>Unit cohesion (-)<br>Homecoming experience (DDRI-2) (0)<br>Combat exposure (CES) (+)<br>Malevolent environments (CES) (+)<br>Perceived threat (CES) (+)<br>Demographics<br>Age (0)<br>Education (-)<br>Marital status (0)<br>Income (0)	Overall %<br>Cut off 4+: 104(29.4%)<br>Cut off 3+: 176(49.7%)<br>Subscales %<br>Transgressions -others cut off 4+: 57(16.1%)<br>Transgressions -self cut off 4+: 53(14.9%)<br>Betrayal cut off 4+: 59(16.7%)<br>Transgressions -others cut off 3+: 130(36.7%)<br>Transgressions -self cut off 3+: 115(32.5%)<br>Betrayal cut off 3+: 121(34.2%)	Overall M/SD<br>1.80(0.80)<br>Subscales<br>Transgressions -others: 2.04(1.04)<br>Transgressions -self: 1.69(0.86)<br>Betrayal: 1.80(0.91)	5	None
Ogle, Reichwald, Rutland 2018<br><br>Narrative synthesis only	Cross sectional	356	Serving military analysts<br><br>Geospatial intelligence analysts (29%), cryptologic language analysts (54.8%) who	Convenience	NR	70.3 %	NR	USA	NR	MIES<br>10 items instead of 9<br>3 subscales (transgressions-others, transgressions-self, betrayal)	(Mental) health<br>PTSD (PCL-5) (+) *<br>PTSD Avoidance (PCL-5) (+) *<br>PTSD Mood/cognition (PCL-5) (+) *<br>PTSD Hypervigilance (PCL-5) (+) *<br>Reliving (PCL-5) (+) *	Overall %<br>NR<br>Subscales %<br>NR<br>Per item % split by combat exposure level<br>Item 1: None = 9.1%, Low = 21%, Middle =	Overall M/SD<br>NR<br>Subscales M/SD<br>NR	5	NR

For peer review only

provide support to remote combat and global vigilance operations and who have routine exposure to related video, audio, still images, and other media.	Social adjustment (-) *<br>Affect work/home (+) *<br>Individual and/or work characteristics<br>Combat exposure (CES) (+) *<br>Work satisfaction (-) *transgressions-self and betrayal<br>Work satisfaction (0) transgressions-others<br>Combat ambiguity (+) *<br>* Transgressions-others, transgressions-self and betrayal subscales	26%, High = 47.3%<br>Item 2: None = 11.6%, %, Low = 11.6%, Middle = 6.2%, High = 15.4%<br>Item 3: None = 4.7%, %, Low = 29%, Middle = 46.4%, High = 60.5%<br>Item 4: None = 2.3%, %, Low =8.2%, Middle = 5%, High = 11%,<br>Item 5: None = 2.3%, Low = 9.4%, Middle = 4.1%, High = 9.9%<br>Item 6: None = 4.6%, Low =7.1%, Middle = 2%, High = 8.8%<br>Item 7: None = 2.3%, Low =6.9%, Middle = 2%, High = 7.7%, r_s = .109<br>Item 8: None = 11.3%, Low =17.5%,

For peer review only - http://bmjopen.bmj.com/site/about/guidelines.xhtml

												Middle = 10.1%, High = 20.9%<br>Item 9: None = 7%, Low =11.7%, Middle = 3%, High = 13.2%<br>Item 10: None = 7%, Low =10.5%, Middle = 5%, High = 7.7%			
Wisco et al., 2017.	Cross sectional.	564	Veterans. Experienced combat. Army 41%.	Epidemiological sampling, recruited from the National Health and Resilience in Veterans Study (NHRVS).	NR	93%	76.2%	USA	25 %	MIES: 5+ (moderate)<br>3 subscales (transgressions-others, transgressions-self, betrayal)<br>33.7 years (19.1) since event (last combat deployment)	Individual and/or work characteristics<br>Vietnam war era (0)<br>Military branch (army) (+)<br>Multiple deployments (+)<br>Moderate/heavy combat (+)<br>Demographics<br>Age (0)<br>Gender (0)<br>White race (-)<br>College education (-)<br>Annual income >60k (-)<br>Currently employed (+)	*Reports weighted % and SE<br><br>Overall %<br>223, (41.8%, 2.1%))<br><br>Subscale %<br>Transgressions -Others 126, (25.5%, 1.8%))<br><br>Transgressions -Self 48 (10.8%, 1.3%)<br><br>Betrayal 142 (25.1%, 1.8%)<br><br>Per item<br>Item 1: 115 (23.8%, 1.8%)<br>Item 2: 65, (14.1%, 1.5%)	Overall M/SD 2.11(1.08)<br>Subscales M/SD<br><br>Transgressions -others 2.71(1.66)<br><br>Transgressions -self 1.75(1.16)<br><br>Betrayal 2.19(1.29)	1	None

For peer review only - http://bmjopen.bmj.com/site/about/guidelines.xhtml

												Item 3: 37 (8.9%, 1.2%)<br>Item 4: 30 (5.9%, 1%)<br>Item 5: 21 (3.9%, 0.8%)<br>Item 6: 19 (3.6%, 0.8%)<br>Item 7: 107 (18.6%, 1.7%)<br>Item 8: 81 (15%, 1.5%)<br>Item 9: 31 (5%, 0.9%)			
Zerach, Levi-Belz. 2017	Cross sectional	191	Veterans. 100% army.	Convenience<br><br>March-July 2017.	25.39 (2.37)	85.4 %	NR	Israel	86.8 %	MIES 4+ (slightly agree)<br>3 subscales (transgressions-others, transgressions-self, betrayal)<br>Time since deployment 4.36(2.27) years.<br><br>MIQ-M 2+ (seldom)<br>14 causal items<br>1 subscale (causes)	(Mental) health<br>Depression (DAQ) and MIQ-M (0)<br>Depression (DAQ) and MIES (+) *<br>PTSD (PCL-5) and betrayal (+)<br>PTSD (PCL-5) and transgression-others, transgressions-self and MIQ-M (0)<br>Guilt-global (TRGI) and MIQ-M and MIES (+) *<br>Guilt-distress (TRGI) and MIQ-M and MIES (+) *<br>Guilt-cognitions (TRGI) and MIQ-M and MIES (+) *<br>Shame-intrinsic (TSRI) and MIQ-M and MIES (+) *	Overall %<br>92(48.4%)<br>Subscales<br>Transgressions-others 64 (33.7%)<br>Transgressions-self 42 (21.9%)<br><br>Betrayal 59 (31%)<br><br>Per item MIES %<br><br>Item 1: 55 (29%) Item 2: 42 (22%)<br>Item 3: 23 (12%) Item 4: 25 (13%)<br>Item 5: 19 (10%)	Overall M/SD<br>2.00(0.97)<br>Subscales<br>MIES<br>Transgressions-others<br>2.42(1.39)<br>Transgressions-self<br>1.68(1.05)<br>Betrayal<br>2.14(1.22)<br>Subscales<br>MIQ-M<br>MIQ causes<br>1.34(0.34)	4	NR

For peer review only

For peer review only - http://bmjopen.bmj.com/site/about/guidelines.xhtml

											Shame-extrinsic (TSRI) and MIQ-M and MIES (+) * Self-disgust-self (TRSI) and MIQ-M and MIES (0) * Self-disgust -ways (TSRI) and betrayal (+), Self-disgust ways (TSRI) and transgressions-self, transgressions-others, and MIQ-M (0) Individual and/or work characteristics Combat exposure (CES) (+) MIQ-M Combat exposure (0) MIES * *transgressions-self, transgressions-other and betrayal subscales	Item 6: 23 (12%) Item 7: 48 (25%) Item 8: 32 (17%) Item 9: 29 (15%) Per item MIQ-M %<br><br>Item 4: 86 (45.2%) Item 8: 60 (31.4%)			
Papazoglou et al., 2019.	Cross sectional.	390	Service police officers. National Police of Finland. Average years of police experience 16.87(9.11)	Convenience	41.21 (8.42)	73.5 %	100%	Finland	86 %	MIES 5+ (moderately agree) and 6+ (strongly agree) Item 6 not included 2 subscales (perpetration and betrayal)	(Mental) health PTSD (PCL-C) (+) * Compassion fatigue (CSF) (+) * Compassion satisfaction (+) *<br><br>Individual and/or work characteristics Dark triad (SD3) (+) * *Perpetration and betrayal subscales	Overall % Overall, MI (moderate): 289 (74%) Subscale % Perpetration 152 (39%) (moderate) Betrayal 250 (64%) (moderate) Perpetration 39 (10%) (high)	Overall M/SD NR Subscale M/SD Perpetration 2.61 (1.19)<br><br>Betrayal 3.77 (.98)	4	NR

For peer review only

												Betrayal 94 (24%) (high)			
Richardson, Chesnut et al., 2020	Longitudinal (T2)	5,227	Veterans. Recently separated from service<br><br>Army (36.8%), Navy (22.4%), Air Force (20.5%), marines (20.2%).	Epidemiological, Recruited from The Veteran Metrics Initiative (TVMI) study.	NR	82.1 %	66.9%	USA	10.67%	MIES 4+ (slightly agree)<br>2 subscales, (MI-self and MI-other)<br>Within 90 days since event (separation from service)	NR	Overall % NR<br>Subscale %<br>MI-Self: 516 (9.87%)<br>MI-Other: 1033 (19.76%)<br>Per item prevalence<br>Item 1: 2432 (46.53%)<br>Item 2: 1496 (28.62%)<br>Item 3: 833(15.94%)<br>Item 4: 750(14.35%)<br>Item 5: 813(15.55%)<br>Item 6: 741(14.18%)<br>Item 7: 1955(37.4%)<br>Item 8: 1472 (28.16%)<br>Item 9: 1039 (19.88%)	Overall M/SD 2.24(1.07)<br>Subscale M/SD<br>MI-Self: 1.79(1.25)<br>MI-Other: 2.51(1.40)	2	None

For peer review only - http://bmjopen.bmj.com/site/about/guidelines.xhtml

Feinstein<br>,<br>Pavisian,<br>Storm.<br>2018	Cross<br>sectional	80	Journalists.<br>Years in<br>role 18.28<br>(8.09)<br><br>months<br>covering<br>the refugee<br>crisis<br>19.65<br>(19.41)	Convenience	42.95<br>(8.44)	58.8<br>%	70.40%	Nine<br>European and<br>American<br>news<br>organisations	70.2<br>%	MIES<br>6 items<br>1 subscale<br>(perpetration)	(Mental) health<br>PTSD intrusion (IES-R) (+)<br>PTSD avoidance<br>(IES-R) (+)<br>PTSD arousal (IES-R) (+)<br>Depression (BDI-II)<br>(0)<br>Alcohol consumption<br>(0)<br>Individual and/or<br>work characteristics<br>Higher workload in<br>past year (+)<br>Control over<br>resources (-)<br>Guilt (+)<br>Demographics<br>Having children (+) t-test<br>Gender (0)<br>Age (0)<br>Education (0)<br>Marriage (0)<br>Marital status (0)	Overall %<br>NR<br>Subscales %<br>NR	Overall M/SD<br>NR (only one<br>subscale)<br>Subscales<br>Perpetration<br>2.83(0.93)	5	HR is<br>director<br>of INSI
Sugrue,<br>2020	Cross<br>sectional	218	K-12<br>education<br><br>Classroom<br>teacher<br>(50.5%),<br>special<br>education<br>staff<br>(19.3%),<br>Non-classroom<br>instruction	Convenience	42.6<br>(11.9)	22.9<br>%	77.1%	USA	<7<br>%	MIES 4+<br>(slightly<br>agree)<br>3 subscales<br>(transgressions-others,<br>transgressions-self,<br>betrayal)	(Mental) health<br>Transgressions-others<br>subscale:<br>Personal burnout<br>(CBI) (+)<br>Work burnout (CBI)<br>(+) *<br>Client burnout (CBI)<br>(+)<br>Global guilt (TRGI)<br>(+)<br>Distress Scale<br>(TRGI) (+)	Overall %<br>191 (85.3%).<br>Subscale %<br><br>Transgressions<br>-others, 175<br>(80.2%)<br><br>Transgressions<br>-self, 98<br>(45.4%)	Overall M/SD<br>3.49(1.24)<br>Subscale<br>M/SD<br>Transgressions<br>-<br>others 4.4(1.5)<br>Transgressions<br>-self, 2.9(1.5)<br>Betrayal<br>3.8(1.5)	5	NR

For peer review only - http://bmjopen.bmj.com/site/about/guidelines.xhtml

al support (10.1%), School social workers (5%), English language learner (ELL) teachers (5%), Specialist teachers (4.6%), School psychologists (3.7%), School counsellors (0.9%), school nurses (0.5%)<br><br>Average years working in education 15.0(10.1)	Hindsight bias (TRGI)<br>Wrongdoing (TRGI)<br>Lack of justification (TRGI) (0)<br>Stress of conscience (internal and external subscales and total (SCQ)) (+)<br>Transgressions-self subscale:<br>Personal burnout (CBI) (+)<br>Work burnout (CBI) (+) *<br>Client burnout (CBI) (0)<br>Global guilt (TRGI) (+)<br>Distress Scale (TRGI) (+)<br>Hindsight bias (TRGI)<br>Wrongdoing (TRGI)<br>Lack of justification (TRGI) (+)<br>Stress of conscience (internal and external subscales and total (SCQ)) (+)<br>Betrayal subscale:<br>Personal burnout (CBI) (+)<br>Work burnout (CBI) (+) *<br>Client burnout (CBI) (+)<br>Global guilt (TRGI) (+)	Betrayal, 148 (68.4%)<br><br>Transgressions -others, 115(52.7%) – moderate (5) cut off

For peer review only

For peer review only - http://bmjopen.bmj.com/site/about/guidelines.xhtml

											Distress Scale<br>(TRGI) (+)<br>Hindsight bias<br>(TRGI)<br>Wrongdoing (TRGI)<br>Lack of justification<br>(TRGI) (0)<br>Stress of conscience<br>(internal and external<br>subscales and total<br>(SCQ)) (+)<br>Individual and/or<br>work characteristics<br>Transgressions-others<br>subscale<br>Intention to leave<br>(ILS) (+)<br>Transgressions-self<br>subscale<br>Intention to leave<br>(ILS) (0)<br>Betrayal subscale<br>Intention to leave<br>(ILS) (+)				
Aldridge,<br>Scott,<br>Paskell.<br>2019.	Cross<br>sectional.	104	Veterans.<br><br>British<br>Army,<br>Royal<br>Navy or<br>Royal Air<br>Force and<br>had<br>experience<br>d active<br>deploymen<br>t.	Convenience<br><br>Feb 2017-<br>Nov 2017.	47<br>(10.8)	95.2<br>%	NR	UK	NR	MIQ-M<br>19 items<br>Unidimensi<br>onal.	(Mental) health<br>PTSD (PCL-M) (+)<br>Individual<br>characteristics<br>Guilt proneness<br>(TOSCA)<br>(-)<br>Shame proneness<br>(TOSCA) (0)<br>Demographics<br>Age (0)<br>Gender (0)<br>Length of service (0)<br>Previous therapy (0)	Overall %<br>NR<br>Subscale %<br>NR	Overall M/SD<br>36.75(10.12)<br>Subscale<br>M/SD<br>NR	5	NR

For peer review only

For peer review only - http://bmjopen.bmj.com/site/about/guidelines.xhtml

Andrukonis, Potopopova. 2020.	Cross sectional.	153	Animal shelter.<br><br>Shelter director (n=16), assistant director (4), animal shelter supervisor/manager (21), animal control officer (18), kennel attendant/animal care tech (29), customer service representative (13), vet/vet tech/medical (22), behaviour/training (9), volunteer/rescue/community (3), other (22)	Convenience<br><br>Dec 2017-April 2018.	NR	NR	NR	USA	NR	MIES Higher score represents lower moral injury Unidimensional.	Individual and/or work characteristics<br>Shelter type (0)<br>Admission type (0)<br>Euthanizes in job (-)<br>Demographics<br>Months on the job (0)	Overall %<br>NR<br><br>Subscale %<br>NR	Overall M/SD<br>0.96(4.22)<br>Subscale M/SD<br>NR	5	None

For peer review only

			Years in job M=5.9												
Haight., Sugrue, Calhoun. 2017.	Cross sectional	38	CPS Social worker (n = 13)<br>County attorney (n = 5)<br>Guardians ad litem (n = 5)<br>Student attorneys (n = 10)<br>Law school clinic and parent support staff (n = 5)<br>Years in CPS 8.5(8.6)	Convenience	NR	18%	66%	USA	56.3 3%	MIES 4+ (slightly agree)<br><br>3 subscales (transgressions-others, transgressions-self, betrayal)	Demographics<br>Age (0)<br>Gender (0)<br>Race (0)<br>Years of experience in CPS (0)<br>Years of education and transgressions-self and betrayal (-)	Overall %<br>NR<br>Subscale %<br><br>Transgressions -others 35(92%)<br><br>Transgressions -self 9(24%)<br><br>Betrayal 12(32%)	Overall M/SD<br>NR<br>Subscale M/SD<br>Transgression-others 5.1(1)<br>Transgression-self 2.7(1.4)<br>Betrayal 3.1(1.2)	5	NR

For peer review only

Battles et al. 2019.	Cross sectional	380	Currently serving military and veterans that had deployed to 1+ war zones for 3+ months. Navy (43.5% of men, 53.3% of women). No longer serving (67.1% of men, 66.7%) of women. Length of time serving (men 12.88, 10, women, 9.84, 8)	Convenience	35.29 (9.58)	68.42 %	71.9% of men and 61.7% of women	USA	NR	MIQ-M 12 causal items 1 subscale (causes)	(Mental) health Spiritual injury (+) Total alcohol use (AUDIT) in men (+) Total alcohol use in women (0) Alcohol consumption (AUDIT) in men (+) Alcohol consumption (AUDIT) in women (0) Alcohol dependence (AUDIT) in men (+) Alcohol dependence (AUDIT) in women (0)<br><br>Individual and/or work characteristics Combat exposure (CES) (+) Military status (+) (0) Military branch (+)<br><br>Demographics Years in service (0)	Overall % NR Subscale % NR	Overall M/SD NR Subscale M/SD (causes 12 items) 1.26(0.42)	5	NR
Braitman et al., 2018.<br><br>Narrative synthesis only	Cross sectional	328	Current military or veterans with deployed experience Veterans (69.5%) National Guard/Reservists	Convenience	32.42 (7.71)	56.1 %	68.0%	USA	NR	MIQ-M modified 2+ (seldom) 27 PMIE items plus 135 follow up questions (5 per PMIE item)	(Mental) health Depression (CES-D-10) (+) Anxiety (KAS) (+) PTSD (PCL-5) (+) Hazardous alcohol use (AUDIT) (+) Drug abuse (DAST) (+) Drug abuse (DAST) (0) *	Overall % NR<br><br>Subscale % NR<br><br>Per item % Item 7: 216 (65.9%) Item 8: 174 (53%)	Overall M/SD NR Subscale M/SD<br><br>AoW PMIEs 1.77(0.63) PCoW PMIEs 1.99(0.66) LFB PMIEs 2.12(0.78)	5	None

For peer review only - http://bmjopen.bmj.com/site/about/guidelines.xhtml

For peer review only

(19.5%)<br>Active-duty members (9.5%)<br>Army (46.3%)<br>Navy (29.9%)<br>Air force (7.9%)<br>Marines (5.5%)<br>National guard (4%)<br>Reserves (4.6%).	3 subscales. (atrocities at war (AoW); psychological consequences of war (PCoW), leadership failure or betrayal (LFB).	All subscales<br>*LFT and AoW	Item 10: 182(55.5%)<br>Item 11: 155(47.3%)<br>Item 15: 181(55.2%)<br>Item 16: 150(45.7%)<br>Item 17: 178(54.3%)<br>Item 19. 191(58.2%)<br>Item 22: 179(54.6%)<br>Item 23: 170(51.8%)<br>Item 24: 198(60.4%)<br>Item 26: 192(58.5%)<br>Item 27: 183(55.8%)<br>Item 6: 208 (63.4%)<br>Item 9: 231 (70.4%)<br>Item 13: 233 (71%)<br>Item 18: 227(69.2%)<br>Item 21: 220(67.1%)<br>Item 25: 199(60.7%)<br>Item 1: 218(66.5%)<br>Item 2: 225(68.6%)<br>Item 4.	AoW defining characteristics 2.21(0.67)<br>PCoW defining characteristics 2.14(0.66)<br>LFB defining characteristics 2.12(0.67)<br>All defining characteristics 2.11(0.66)

For peer review only

												239(72.9%)<br>Item 3:<br>200(61%)<br>Item 5:<br>168(51.2%)<br>Item 12:<br>208(63.4%)<br>Item 14:<br>174(53%)<br>Item 20.<br>214(65.2%)			
Lancaster Harris, 2018.	Cross sectional	182	Veterans Deployed to Iraq or Afghanistan<br>Army (61.0%)<br>Air Force (17.0%),<br>Marine Corps (14.8%),<br>Navy (7.1%).	Convenience	33.66 (7.23)	80.2 %	78.0%	NR	32.3 3%	MIES<br>3 subscales (transgressions-others, transgressions-self, betrayal)<br><br>MIQ-M<br>19 items<br>2 subscales (causes and effect)	(Mental) health<br>Anger (DAR-5) (+) *<br>Depression (PHQ) (+) *<br>Alcohol (AUDIT) (+) *<br>PTSD (PCL-5) (+) *<br>Guilt (+) *<br>Shame (+) *<br><br>Individual and/or work characteristics<br>Combat (CES) (+)*<br>CES (0)**<br><br>All subscales<br>*transgressions-others and transgressions-self subscales<br>**betrayal subscale	Overall %<br>NR<br><br>Subscale %<br>NR	Overall M/SD MIES<br>2.75(1.32)<br><br>Subscale M/SD MIES<br>Transgressions-others<br>3.49(1.50)<br><br>Transgressions-self<br>2.45(1.53)<br><br>Betrayal<br>2.67(1.57)<br><br>Overall M/SD MIQ-M<br>33.70(11.90)<br><br>Subscale M/SD MIQ-M<br>Effect<br>11.54(4.21)<br><br>Causes<br>22.19(8.32)	5	NR

For peer review only - http://bmjopen.bmj.com/site/about/guidelines.xhtml

Lancaster, 2018	Cross sectional	161	Veterans. Post 9/11<br><br>Army (43.5%), Navy (19.9%), Air Force (16.1%), Marine Corps (13.7%), Coast Guard (6.8%).	Convenience	35.08 (9.62)	71.4 %	73.9%	NR	5.18 %	MIES<br>7 of 9 items<br>2 subscales (transgressions-self and betrayal)	(Mental) health<br>PTSD (PTSD checklist 5) (+) *<br>Depression (PHQ) (+) *<br>Anger (+) *<br>Guilt/shame (SSGS) (+) *<br><br>Individual and/or work characteristics<br>Transgressive acts (TAS) (+) *<br>*transgressions-self and betrayal subscales	Overall %<br>NR<br>Subscales %<br>NR<br>*Range 12.6% to 23.3% on items	Overall M/SD<br>NR (only 2 subscales used)<br><br>Subscales M/SD<br>Transgressions-self, 2.56(1.43)<br>Betrayal 2.72(1.46)	5	NR
Akhtar, Faize, Malik & Tabusam, 2022	Cross-sectional	108	Healthcare<br>Doctors (51.9%)<br>Nurse (26.9%)<br>Paramedics (21.3%)	Purposive<br><br>August 2020-January 2021.	27.67 (6.17)	41.7 %	NR	Pakistan.	NR	MISS-HP 36+	Individual and/or work characteristics<br>Psychological resilience (CD-RISC-10): (-)<br>Demographics<br>Age (0)<br>Experience (-)<br>Work hours (+)<br>Female gender (+)<br>Marital status (0)<br>Hospital type (0)<br>Profession (0)<br>Speciality/department psychiatry (+)<br><br>Work area/duty station (0)	Overall %<br>75 (69.44%)	Overall M/SD<br><br>43.36 (9.64)	6	NR

For peer review only

For peer review only - http://bmjopen.bmj.com/site/about/guidelines.xhtml

Qi et al., 2022<br>Two studies.	Study 1: Cross-sectional<br>Study 2: Cross-sectional	Study 1: 3006<br>Study 2: 3465	Study 1: Healthcare Nurse (18%) Internal medicine (19.4%) Obstetrics/ gynaecology/paediatrics (34.7%) Surgery (9.6%) Psychiatry (11.4%) Other<br>Study 2: Healthcare Nurse (14.7%) Internal medicine (44.7%) Obstetrics/ gynaecology/paediatrics (21.1%) Surgery (4.9%) Psychiatry (8.5%) Other (6.1)	Study 1: Snowball. March-April 2020.<br>Study 2: Snowball. March-April 2021	Study 1: NR<br>Study 2: NR	Study 1: 34.9 %<br>Study 2: 24.6 %	Study 1: NR<br>Study 2: NR	Study 1: China<br>Study 2: China	Study 1: 75 %<br>Study 2: 86 %	Study 1: MISS-HP 36+ EMIS-SF<br>Study 2: MIISS-HP 36+	Study 1: (Mental) health Depression (PHQ) (+)<br>Anxiety (GAD-7) (+)<br>Burnout emotional exhaustion (MBI-HSMP) (+)<br>Burnout reduced personal accomplishment (MBI-HSMP) (-)<br>Burnout depersonalisation (MBI-HSMP) (+)<br>Well-being (SFI) (-)<br>* EMIS and MISS-HP<br>Study 2: NR at univariate level.	Study 1: Overall, MISS-HP % 1695 (56.4%)<br>Study 2: Overall, MISS-HP % 1270 (36.7)	Study 1: MISS-HP overall: 46.9 (12.7) EMIS-SF overall: 2.55 (0.80)<br>Study 2: MISS-HP: 45.9(11.3)	5	None

For peer review only

Amsalem et al., 2021	Longitudinal	350	Healthcare<br>Nurses (68%)<br>Physicians (15%)<br>Emergency medical technicians (9%)<br>Physical therapists (3%)<br>Pharmacists (2%)<br>Social workers and other therapists (1%)<br>Other (2%)	Convenience<br>September-December 2020	34.8 (11.5)	26%	73%	USA	NR	MIES: 3+ (agree)<br>Likert scale 1 (disagree), 2 (strongly disagree) 3 (agree) 4 (strongly agree)<br><br>3 subscales (transgressions-others, transgressions-self, betrayal)	(Mental) health<br>Depression (PHQ) (+)<br>Anxiety (GAD-7) (+)<br>Suicidal ideation (+)<br>PTSD (PC-PTSD) (+)<br>Demographics<br>Age (0)<br>Experience (-)<br>Work hours (+)<br>Female gender (+)<br>Marital status (0)<br>Hospital type (0)<br>Profession (0)<br>Speciality/department psychiatry (+)<br>Work area/duty station (0)	Overall %<br>267 (76.29%)<br>Per subscale %<br>Transgressions -others: 215 (61.43%)<br><br>Transgressions -self: 101 (28.86)<br>Betrayal: 205 (58.57%)<br>Per item:<br>Item1: 192 (55%)<br>Item 2: 171 (49%)<br>Item 3: 66 (19%)<br>Item 4: 68 (19%)<br>Item 5: 71 (20%)<br>Item 6: 72 (21%)<br>Item 7: 157 (45%)<br>Item 8: 111 (32%)<br>Item 9: 152 (43%)	Overall M/SD<br>1.98 (0.72)<br>Subscale M/SD<br>Transgressions -others: 2.41(1.04)<br><br>Transgressions -self: 1.63(0.82)<br>Betrayal: 2.15(0.92)	5	None

For peer review only

For peer review only - http://bmjopen.bmj.com/site/about/guidelines.xhtml

Perez, Larson & Bair 2021	Cross-sectional	85	Veterans Served between 2001-2014. Army (63.5%) Marines (8.2%) Navy (10.6%) Air Force (15.3%) Coast Guard (2.4%)	Convenience	NR	68.2 %	78.8%	USA	NR	EMIS 2 subscales (self-directed MI and other-directed MI)	Individual and/or work characteristics Fairness (MFQ) (0) Loyalty (MFQ) (+) * Loyalty (MFQ) (0) ** Authority (MFQ) (0) Sanctity (MFQ) (0) *Self-MI **Other-MI	Overall % NR Per subscale NR	Overall M/SD 2.45(0.98) Subscale M/SD Self-MI 2.16(1.08) Other-MI 2.80(1.06)	5	NR
Benatov, Zerach & Levi-Belz 2022	Cross-sectional	296	Health and social care Doctor (34.1%) Nurse (including midwives) (44.4%) Social and psychological care (17.4%) Clinical support (5.9%)	Convenience	40.28 (10.8)	22.4 %	NR	Israel	NR	MIES 4+ (slightly agree) 3 subscales (transgressions-others, transgressions-self, betrayal)<br><br>MISS-HP 36+	(Mental) health Depression (PHQ) (+) Anxiety (GAD-7) (+) Individual and/or work characteristics Thwarted belonginess (INQ) (+)<br><br>*For MISS-HP and MIES scales	MIES: Overall %: NR Per subscale % Transgressions -others: 146 (49.3%)<br><br>Transgressions -self: 94 (31.8%) Betrayal: 184 (62.2%) Per item %: Item 1: 135 (45.5) Item 2: 108 (36.5%) Item 7: 135 (45.5%) Item 9: 94 (31.8%)	MIES: Overall M/SD (From supplement) 22.54(10.48) Subscale M/SD<br><br>Transgressions -others: 4.08(2.71) Transgressions -self: 1.49(0.81) Betrayal: 2.81(1.33) MISS-HP overall: 33.90(12.72)	5	NR

For peer review only - http://bmjopen.bmj.com/site/about/guidelines.xhtml

												MISS-HP overall %<br>120 (40.7%)			
Borges et al., 2021	Longitudinal	211	Healthcare Physician (12.32%)<br>Nurse (10.43%)<br>Mental health provider (61.14%)<br>Occupational therapist (1.90%)<br>Nurse practitioner (2.37%)<br>Physician assistant (0.47%)	Convenience<br>May 2020 – March 2021	NR	16.26 %	89.16%	USA	NR	MIES 2+ (moderately agree)<br>Lower score indicates higher moral injury<br>2 subscales (perpetration and betrayal)	Demographics<br>Sex (0)<br>Race (0)<br>Profession (0)<br>No other variables reported at univariate level	Overall %<br>109 (51.66%)<br>Per subscale %<br>Perpetration: 77 (36.49%)<br>Betrayal: 88 (41.71%)	Overall M/SD<br>NR<br>Subscale M/SD<br>NR	5	None
Boscarino et al., 2022<br><br>Narrative synthesis only	Cross-sectional	1032	Military veterans.	Random sample of veterans on a registry care at a private hospital.<br><br>February 2016 – February 2017	NR	95.5 %	NR	USA	55 %	MIES 75th percentile<br>Unidimensional	(Mental) health<br>Cage positive alcohol score (+)<br>Used alc/drugs to cope (+)<br>Pain interferes (+)<br>Sleep problems (+)<br>Concussion history (+)	Overall %<br>764 (74%)<br>Subscale %<br>NR	Overall M/SD<br>NR<br>Subscale M/SD<br>NR	3	None

For peer review only

For peer review only - http://bmjopen.bmj.com/site/about/guidelines.xhtml

For peer review only

											Mental health treatment in past year (+)<br>Opioid dependence past year (+)<br>Individual/work characteristics<br>Guard/reserve (-)<br>Combat high (+)<br>Drafted (+)<br>Vietnam war deployment (+)<br>Low unit support/morale (+)<br>High stress past year (+)<br>High lifetime trauma (+)<br>Low homecoming support (+)<br>High child abuse/neglect (+)<br>High fear of death (+)<br>High neuroticism (+)<br>Low self-esteem (+)<br>Repression to cope (+)<br>High anomie (+)<br>Anti-social personality (+)<br>Used marijuana 50+ times (+)<br>Demographics<br>Age (+)				
Brady et al., 2021	Cross-sectional<br>.	390	Nursing home staff. Nurses (30.8%)	Convenience November 2020 – January 2021.	NR	12.8 %	83.3%	Republic of Ireland.	10.2 %	MIES 4+ (slightly agree)	Demographics<br>HCA compared to non-clinical staff (+)	Overall % 237 (60.8%)<br>Per subscale %	Overall M/SD 2.35(1.09)<br>Subscale M/SD	4	Honoraria for board meeting.

			Healthcare assistants (44.1%)<br>Non-clinical (25.1%)							Cut off: 4+ (Slightly agree)<br>3 subscales (transgressions-others, transgressions-self, betrayal)		Transgressions -others: 189 (48.5%)<br>Transgressions -self: 92 (23.6%)<br><br>Betrayal: 166 (42.6%)	Transgressions -others: 2.93(1.51)<br>Transgressions -self: 1.97(1.19)<br>Betrayal: 2.46(1.34)		
Brady et al., 2022	Cross-sectional	377	Hospital Doctors (48%)<br>Nurses (44%)<br>Radiographers (8%)	Convenience January – March 2021	NR	22.3 %	90.5%	Republic of Ireland. Dublin.	6%	MIES 4+ (slightly agree)<br>Cut off: 4+ (Slightly agree)<br>3 subscales (transgressions-others, transgressions-self, betrayal)	Demographics Professional role - doctors (-)	Overall % 268 (71.1%)<br>Per subscale %<br>Transgressions -others: 197 (52.3%)<br>Transgressions -self: 92 (24.4%)<br>Betrayal: 210 (55.7%)	Overall M/SD 2.48(1.10)<br>Subscale M/SD<br>Transgressions -others: 3.01(1.56)<br>Transgressions -self: 1.97(1.12)<br>Betrayal: 2.81(1.44)	6	
Chandrabhatla et al., 2022	Cross-sectional		Hospitalist	Convenience September 2019 – August 2020	NR	48%	NR	USA	52 %	MIES Unidimensional	(Mental health):<br>Burnout (0)<br>Burnout during COVID subsample (+)	Overall % 60 (71.4%)*<br>Subscale %<br>Transgressions -others: 49(58.3%)**<br>Transgressions -self: 23(27.4)*<br>Betrayal: 37(44%)*<br>*N=78<br>**N=77	Overall M/SD 2.44(1.07)*<br>Per subscale M/SD<br>Transgressions -others: 3.41(1.64)**<br>Transgressions -self: 1.88(1.15)***<br>Betrayal: 2.54(1.31)***<br>*N=73<br>**N=74<br>***N=76	5	None

For peer review only

Dale et al., 2021	Longitudinal	265	Healthcare	Convenience<br>October 2020 – March 2021	37.62 (11.1)	18.1 %	77.7%	USA	NR	MIES<br>3 subscales (transgressions-others, transgressions-self, betrayal)	NR at univariate level	Overall %<br>NR (7 items only)<br>Subscale %<br>Transgressions-others: 6 (2.3%)<br>Transgressions-self only: 61 (23%)<br>Perpetration: 21 (7.9%)<br>Perpetration (high): 88 (33.2%)	Overall M/SD<br>NR<br>Per subscale M/SD<br>Transgressions-others: 2.54(1.45)<br>Transgressions-self: 1.66(1.05)	5	None
Plouffe et al., 2021<br><br>Two samples	Longitudinal (T2)	<u>1:</u> 803<br><br><u>2:</u> 1510	Military.<br><u>Sample 1:</u> active duty<br><u>Sample 2 -</u> released.	Nationally representative sample.<br>January – May 2018 (T2)	NR	<u>Sample 1:</u> 87.53 %<br><u>Sample 2:</u> 88.02 %	NR	Canada	68 %	MIES<br>Unidimensional	NR	Overall %<br>NR for both samples<br>Subscale %<br>NR for both samples<br><br>Per item %<br><u>Sample 1:</u><br>Item 1: 64.6%<br>Item 2: 37.3%<br>Item 3: 16.9%<br>Item 4: 13.2%<br>Item 5: 19.9%<br>Item 6: 14.9%<br>Item 7: 48.5%<br>Item 8: 34.5%<br>Item 9: 27.5%<br><u>Sample 2:</u><br>Item 1: 66.1%<br>Item 2: 42.2%<br>Item 3: 20.4%<br>Item 4: 17.3%	Overall M/SD<br>Sample 1: 2.46(4.53)<br>Sample 2: 2.63(4.66)<br><br>Subscale M/SD<br>NR for both samples	2	None

For peer review only

												Item 5: 24.4%<br>Item 6: 21.3%<br>Item 7: 53%<br>Item 8: 37.8%<br>Item 9: 34.6%			
Nichter et al., 2021	Cross-sectional	1321	Veterans. All reported exposure to combat/war zone.<br>Army (47.6%)<br>Navy (14.6%)<br>Air Force (6.3%)<br>Marines (6.3%)<br>National Guard/Reserves/Coast Guard (9.3%)	National random samples and apply post-stratification wrights to generalise to entire US veteran population. 2019-2020	59.1 (16.6)	93.7 %	75.1%	USA	NR	MIES 5+ (moderate)<br>3 subscales (transgressions-others, transgressions-self, betrayal)	NR at univariate level.	*Reports weighted % and SE<br><br>Overall %<br>535, (36.8%, 1.3%)<br><br>Subscale %<br>Transgressions -Others 316, (23.1%, 1.2%)<br><br>Transgressions -Self 152 (11.2%, 0.9%)<br><br>Betrayal 189 (24.5%, 1.3%)<br><br>Per item<br>Item 1: 279 (21%, 1.1%)<br>Item 2: 189, (13.6%, 1.0%)<br>Item 3: 90 (6.5%, 0.7%)<br>Item 4: 88 (6.0%, 0.8%)	Overall M/SD 2.0(1.1)<br>Subscales M/SD<br><br>Transgressions -others 2.5(1.6)<br><br>Transgressions -self 1.7(1.1)<br><br>Betrayal 2.1(1.3)	1	NR

For peer review only

												Item 5: 77 (6.0%, 0.7%)<br>Item 6: 71 (5.9%, 0.7%)<br>Item 7: 289 (17.9%, 1.1%)<br>Item 8: 214 (14.9%, 1.0%)<br><br>Item 9: 79 (6.5%, 0.6%)			
Fitzpatric k et al., 2022	Cross-sectional.	676	Nurses.<br>Staff nurses (65%)<br>Nurse leaders (35%)	Convenience June - August 2021.	43.88 (1.99)	8.3%	89.6%	USA	NR	MISS-HP	(Mental) health<br>Lower well-being (+)<br>Individual and/or work characteristics<br>Resilience (-)<br>Demographics<br>Staff nurses (+)	Overall %<br>NR	Overall M/SD<br>42.21(15.73)	5	NR
Hagerty & Williams, 2022<br><br>Narrative synthesis only	Cross-sectional	1122	Healthcare<br>Nurse (60.4%)<br>Physician (5%)<br>Physician assistant (1.7%)<br>Respiratory therapist (3.6%)<br>Other health-care roles (28%)<br>Provided care in ICU during the	Convenience May 2020 – August 2020.	39.29	11.2 %	NR	USA	NR	MIES 4+ (slightly agree)<br>3 subscales (transgressions-others, transgressions-self, betrayal)	(Mental) health<br>Loneliness (DJGLS) (+)<br>Demographics<br>Age (-)<br>Access to PPE (-)<br>Number of COVID-19 patient deaths that respondents personally treated (+)	Overall %<br>NR<br><br>Subscale %<br>NR<br><br>Per item %<br><br>Item 2: 525 (47.2%)<br>Item 7: 747 (67.2%)<br>Item 4: 234 (21%)	Overall M/SD<br>NR<br>Subscale M/SD<br>NR	5	None

For peer review only - http://bmjopen.bmj.com/site/about/guidelines.xhtml

For peer review only

			pandemic (47.6%)												
Hamrick et al., 2022	Cross-sectional	154	Military (serving and veterans). Former/retired (77.9%). Army (20.8%) Navy (52.6%) Airforce (16.2%) Marines (4.5%) Coast Guard (0.6%) National Guard (7.8%) (Above) reserves (20.8%) Other (0.6%)	Convenience February – April 2019	36.6 (11.8)	0%	72.1%	USA	NR	EMIS-M 1 subscale (other-directed)	(Mental) health Depression (+) Anxiety (+) Suicidal ideation (0) Substance use (0) Individual and/or work characteristics Sexual harassment (+)<br><br>*All correlations with the other-directed MI subscale.	Overall % NR<br><br>Subscale % NR	Overall M/SD NR Subscale M/SD Other-directed MI 2.66(1.17)	5	None

For peer review only

For peer review only - http://bmjopen.bmj.com/site/about/guidelines.xhtml

Hines et al., 2020	Cross-sectional	219	Health and social care Attending physician (47%) Fellow physician (18%) Resident physician (20%) Other (15%)	Convenience March – April 2020	39.10 (11.1)	43%	NR	USA	26 %	MIES 2 subscales (perpetration, and betrayal).	NR at univariate level.	Overall % NR<br><br>Subscale % NR	Overall M/SD 1.79 (0.87) Subscale M/SD Perpetration 1.65 (0.87) Betrayal 2.10 (1.28)	5	NR
Hinkel et al., 2022	Cross-sectional	59	Veterans.	Convenience November 2018 – November 2019	NR	86.4 %	59.3%	USA	NR	EMIS-M 2 subscales (self-directed MI and other-directed MI)	(Mental) health PTSD (PCL-5) (+) MDD (PHQ-8) (+) Chronic pain (BPI-SF) (+)	Overall % NR<br><br>Subscale % NR	Overall M/SD NR Subscale M/SD Self-directed MI 2.29(1.09) Other-directed MI 2.54(1.32)	5	NR
Kelley et al., 2021	Cross-sectional	269	Veterans. Navy (45.4%) Marine Corps (21.9%) Army (19.7%)	Convenience	36.5 (11)	49.8 %	74.3%	USA	NR	EMIS-M 2 subscales (self-directed MI and other-directed MI)	(Mental) health Suicidal ideation (+) PTSD diagnosis (+) Individual/work characteristics Presence of meaning in life (MLQ) (-) Searching for meaning in life (MLQ) (0) Demographics Veteran status (+) Age (0) *Both EMIS subscales	Overall % NR<br><br>Subscale % NR	Overall M/SD NR Subscale M/SD Self-directed MI 1.83(0.86) Other-directed MI 2.48(1.11)	5	None

For peer review only - http://bmjopen.bmj.com/site/about/guidelines.xhtml

La Fleur et al., 2020	Cross-sectional	212	Veterans.<br>Marine Corps (13.7%)<br>Army (26.4%)<br>Navy (13.7%)<br>Air Force (5.7%)<br>Reserves (2.8%)<br>National Guard (4.2%)<br>Multiple (33.5%)	Convenience	NR	80.7 %	74.1%	USA	NR	MIQ-M 2.80+<br>19 items<br>Unidimensional.	(Mental) health<br>Life satisfaction (0)<br>Individual/work characteristics<br>Disclosure support (0)<br>Disclosure concerns (+)	Overall %<br>27%<br><br>Overall %<br>NR	Overall M/SD<br>2.04(0.79)<br>Subscale M/SD<br>NR	6	None
Lamb et al., 2021	Cross-sectional (cohort).	2166	Healthcare<br>Doctor (11.7%)<br>Nurse (29.9%)<br>Other clinical (26.8%)<br>Non-clinical (32.3%)	Convenience April-June 2020.	NR	59.4 %	24.6%	UK	12 %	MIES 5+ (moderately agree)<br>Subscales: 3 subscales (commission, omission and betrayal)	Demographics<br>Age (-)<br>Sex other (+)<br>Relationship status divorced/separated/widowed/single (+)<br>Ethnicity Asian (+)<br>Country of birth (0)<br>Length of time living in UK (-)<br>Main role nurse (+)	*Reports weighted %<br>Overall %<br>602 (25.09%)<br>Subscale %<br>Commission: 346 (14.28%)<br>Omission: 111 (4.57%)<br><br>Betrayal: 439 (18.11%)	Overall M/SD<br>1.72 (1.19)<br>Subscale M/SD<br>Commission: 1.71 (1.06)<br>Omission: 1.47 (1.1)<br>Betrayal: 1.8 (1.14)	2	Grant.
Litam et al., 2021	Cross-sectional	109	Healthcare<br>Physicians (37%)<br>Nurses (57%)<br>Other professionals (6.4%)	Convenience May-July 2020	37.50 (12.4)	24%	75.2%	USA	NR	MIES<br>Lower score indicates higher moral injury<br>Unidimensional	(Mental) health<br>Compassion satisfaction (PROQOL) (+)<br>Burnout (PROQOL) (+)<br>Secondary traumatic stress (PROQOL) (+)	Overall %<br>NR<br><br>Subscale %<br><br>NR	Overall M/SD<br>3.03(0.72)<br>Subscale M/SD<br>NR	5	None.

For peer review only

For peer review only - http://bmjopen.bmj.com/site/about/guidelines.xhtml

Litz et al., 2022<br><br>Four studies	Cross-sectional	1: 533<br>2: 363<br>3: 264<br>4: 118	Samples 1, 2 and 3: Veterans<br>Sample 4: Defence members and veterans.	Sample 1: Convenience<br>Samples 2, 3 and 4: NR	Sample 1: 51.87 (9.77)<br>Samples 2, 3 and 4: NR	Sample 1: 71%<br>Sample 2: 73.6 %<br>Sample 3: 67%<br>Sample 4: 65.9 %	NR for all	Sample 1: Canada<br>Sample 2: USA<br>Sample 3: UK<br>Sample 4: Australia	NR for all	MIOS<br>2 subscales (shame-related outcomes and trust violation-related outcomes)	NR at univariate level.	Sample 1:<br>Overall %<br>366 (68.7%)<br>Subscale %<br>MI-Self: 265 (49.7%)<br>MI-Other: 378 (71%)<br>Impact from an MI-Other: 440 (82.5%)<br>Sample 2:<br>Overall %<br>283 (78%)<br>Subscale %<br>MI-Self: 142 (39%)<br>MI-Other: 142 (39%)<br>Impact from an MI-Other: 142 (39%)<br>Sample 3:<br>Overall %<br>264 (100%)<br>Subscale %<br>MI-Self: 174 (65.9%)<br>MI-Other: 170 (64.4%)<br>Impact from an MI-Other: 186 (70.1%)<br>Sample 4:<br>Overall %<br>NR<br>Subscale %<br>MI-Self: 65 (55%)	Sample 1:<br>Overall M/SD<br>25.31(11.38)<br>Subscale M/SD<br>Shame-related outcomes: 11.28(6.64)<br>Trust violation outcomes: 14.03(5.85)<br>Sample 2:<br>Overall M/SD<br>25.14(11.36)<br>Subscale M/SD<br>Shame-related outcomes: 11.36(6.82)<br>Trust violation outcomes: 13.78(5.58)<br>Sample 3:<br>Overall M/SD<br>32.87(10.54)<br>Subscale M/SD<br>Shame-related outcomes: 16.29(6.20)<br>Trust violation outcomes: 16.58(5.35)<br>Sample 4:<br>Overall M/SD<br>27.74(10.42)<br>Subscale M/SD	5<br><br>4: 6	None

For peer review only

												MI-Other: 93 (79%)<br><br>Impact from an MI-Other: 103 (87%	Shame-related outcomes: 12.17(6.35)<br>Trust violation outcomes: 15.56(5.52)		
Maftei & Holman (2021)	Cross-sectional	114	Physicians	Convenience April 2020	38.85 (9.82)	24.6 %	NR	Romania	1.14 %	MIES 4+ (agree)<br>1-5 Likert scale<br>3 subscales (transgressions-others, transgressions-self, betrayal)	(Mental health)<br>Emotional impact (0) *<br>Emotional impact (+) **<br>Physical impact (+)<br>Demographics<br>Age (0)<br>Working experience (0)<br>*non-covid-19 medical unit<br>** covid-19 medical unit	Overall %<br>80 (70.2%)<br><br>Per subscale %<br>Transgressions-others: 61(53.5%)<br>Transgressions-self: 31(27.2%)<br>Betrayal 66(57.9%)<br>Per item %<br>Item 1: 45 (39.5%)<br>Item 2: 39 (34.2%)<br>Item 3: 14 (12.3%)<br>Item 4: 15 (13.2%)<br>Item 5: 20 (17.5%)<br>Item 6: 22 (19.3%)<br>Item 7: 35 (30.7%)<br>Item 8: 37 (32.5%)	Overall<br>2.68(0.90)<br><br>Subscale M/SD<br>Transgressions-others: 3.06(1.26)<br>Transgressions-self: 2.20(1.05)<br>Betrayal 3.06(1.08)	5	None

For peer review only - http://bmjopen.bmj.com/site/about/guidelines.xhtml

												Item 9: 33 (33%)			
Maguen et al., 2022	Cross-sectional	14057	Post 9/11 veterans.<br><br>Army (50.5%)<br>Navy/Coast Guard (17.9%)<br>Marines (13.3%)<br>Air Force (18.3%)	Nationally representative, population-based sampling frame<br>April – August 2018	NR	82.3%	66.2%	USA	40%	MIES 4+ (slightly agree)<br>3 subscales (transgressions-others, transgressions-self, betrayal)	Demographics<br>Female gender (+) *<br>Female gender (-) **<br><br>*transgressions-others and betrayal<br>** transgressions-self	Overall %<br>7830 (55.7%)<br>Per subscale %<br>Transgressions-others 6059 (43.1%)<br>Transgressions-self 3064 (21.8%)<br>Betrayal 5651 (40.2%)	Overall M/SD<br>NR<br>Subscale M/SD<br>NR	2	None
Malakoutikhah et al., 2022	Cross-sectional	455	Healthcare<br>Operating room (6.3%)<br>Nursing (73.7%)<br>Anaesthesiology (8.2%)<br>Medicine (7.5%)<br>Midwifery (2.7%)<br>Nursing assistant (1.6%)	Convenience<br>September 2021 – February 2022	NR	16.9%	NR	Iran	NR	MISS-HP 36.5+<br>Item content varied	(Mental) health<br>General health (GHQ) (+)<br>PTSD (IES-R) (+)	Overall %<br>190 (42.7%)<br>Per item %<br>Scoring strongly agree:<br>Item 2: 13 (5.1%)<br>Item 3: 5 (2%)<br>Item 4: 15 (6%)<br>Item 5: 71(28%)<br>Item 6: 37 (14.6%)<br>Item 7: 71(28%)<br>Item 8: 39 (15.5%)<br>Item 9: 4(1.6%)<br>Item 10: 1 (0.4%)	Overall M/SD<br>35.66(12.08)	5	None.

For peer review only - http://bmjopen.bmj.com/site/about/guidelines.xhtml

												Item 11: 3 (1.2%)			
Mantri et al., 2020	Cross-sectional	181	Healthcare Physicians (70.7%)<br>Nurse (9.4%)<br>Other (19.9%)	Convenience November 2019 – March 2020	NR	63.5 %	74.5%	USA	NR	MISS-HP 36+<br><br>EMIS-M 2 subscales (self-directed MI and other-directed MI)	(Mental) health<br>Burnout (MBI) (+)<br>Anxiety (GAD-7) (+)<br>Depression (PHQ-9) (+)<br>Individual/work characteristics<br>Religiosity (-)<br>*MISS-HP correlations	MISS-HP<br><br>Overall %<br>86 (47.5%)	MISS-HP Overall M/SD 36.8(13.3)<br>EMIS-HP Overall M/SD 2.01(0.78)<br>Subscales M/SD NR	5	None
Mantri et al., 2021<br><br>Two samples	Cross-sectional	Sample 1: 450<br><br>Sample 2: 1381	Healthcare<br>Sample 1:<br>Nurse (31.8%)<br>Physician (25.8%)<br>APP (18.9%)<br>Chaplaincy (14.2%)<br>Social worker (2.9%)<br>Other (6.4%)<br>Sample 2:<br>Nurse (56.5%)<br>Physician (12%)	Convenience.<br><br>Sample 1:<br><br>April-May 2020<br><br>Sample 2:<br><br>October-November 2020	NR	Sample 1:<br><br>12.4 %<br><br>Sample 2:<br><br>9.5%	Sample 1:<br><br>88.7%<br><br>Sample 2:<br><br>93.3%	Global<br><br>Primarily North America (USA)	Sample 1:<br><br>72.8 %<br><br>Sample 2:<br>73.7 %	MISS-HP 36+	(Mental) health<br>Burnout emotional exhaustion (aMBI) (-)<br>Burnout depersonalisation (aMBI) (-)<br>Burnout reduced personal/professional accomplishment (aMBI) (+)<br>Individual/work characteristics<br>COVID experience (0) *<br>COVID experience (+) **<br>Religiosity (DUREL) (-)<br>Demographics<br>Age (-)	Overall %<br>Sample 1:<br>84 (18.6%)<br>Sample 2:<br>591 (42.8%)	Overall M/SD<br>Sample 1:<br>27.4(11.6)<br>Sample 2:<br>36.4(13.8)	5	None

For peer review only

			APP (19.8%)<br>Housekeeping (0.1%)<br>Food services (0.1%)<br>Interpreter (0.1%)<br>Chaplaincy (0.1%)<br>Social worker (1.3%)<br>Other (19.9%)								Sex (0)<br>Race (0)<br>Marital status (0) *<br>Marital status (+) **<br>Profession (-)<br>*sample 1<br>** sample 2				
Morris, Webb & Devlin, 2022	Cross-sectional	237	Healthcare<br>Dietetics (2.5%)<br>Education (2.5%)<br>Medical Doctor (0.8%)<br>Nursing (Registered) (26.3%)<br>Nursing (Unregistered) (46.6%)<br>Occupational Therapy (5.1%)<br>Psychology (11.4%)<br>Psychiatry (1.3%)	Convenience	NR	34.2 %	78.9%	UK	NR	MIES 6+ (strongly agree)<br>3 subscales (transgressions-others, transgressions-self, betrayal)	(Mental) health<br>Burnout (ProQoL) (+)<br>Secondary traumatic stress (ProQoL) (+)<br>Compassion satisfaction (ProQoL) (-)<br>Individual/work characteristics<br>Impact of COVID-19 (0)<br>Demographics<br>Gender (0)<br>Ethnicity (0)<br>Gendered ethnicity (0)	Overall %<br>183 (78.5%) *<br>Subscale %<br>Transgressions-others: 154 (67%) **<br>Transgressions-self: 79 (34.3%) **<br>Betrayal: 154 (66.1%) *<br>*n = 233<br>**n = 230	Overall M/SD<br>2.90 (1.41)<br>Subscale M/SD<br>Transgressions-others: 3.79(1.86)<br>Transgressions-self: 2.13(1.47)<br>Betrayal 3.38(1.83)	5	None

For peer review only

			Recreational Co-ordinator (1.7%)<br>Social Work (0.8%)<br>Other (0.4%)												
Rushton et al., 2022	Cross-sectional	595	Healthcare<br>Nurse (58.1%)<br>Physician (11.8%)<br>Respiratory therapist (6.3%)<br>Nurse practitioner/physician assistant (7.1%)<br>Other (16.7%)	Convenience<br>June 2020 – November 2020	NR	NR	82.4%	USA	NR	MISS-HP 36+	Individual/work characteristics<br>Ethical concerns (ECI) (+)<br>Involvement in COVID-19 clinical care (+)<br>Practice location (0)<br>Speciality (0)<br>Demographics<br>Years in profession (-)<br>Profession (0)<br>Education (0)<br>Religious/spiritual preference (+)	Overall %<br>32.4%	Overall M/SD<br>30.57(12.11)	5	None
Nieuwsma et al., 2020	Cross-sectional	315	Post 9/11 veterans with warzone experience.<br>Army (67%)<br>Navy (13.2%)<br>Air Force (9.9%)<br>Marines (9.9%)	Convenience	46.36 (10.4)	86.6 %	55.6%	USA	33.5 %	MIES<br>2 subscales (perpetration and betrayal)<br>MIQ-M<br>20 items<br>Unidimensional.<br>BMIS 2+ (agree)<br>2 subscales (event and sequela).	(Mental) health<br>Depression (BDI-2) (+)<br>Suicidal ideation (BSI) (+)<br>Alcohol use disorder (AUDIT) (+)<br>Drug abuse (DAST) (+)<br>PTSD (DTS) (+)<br>*all correlations positive for BMIS, MIES and MIQ-M	Overall %<br>NR<br>Subscale %<br><br>BMIS-E<br>137 (44.1%)<br>NR for BMIS-S subscale<br>*n = 311	Overall M/SD<br>BMIS<br>3.71(4.38)<br>MIES<br>2.57(1.26)<br>MIQ-M<br>1.49(0.47)<br>Subscale M/SD<br><br>NR for all scales<br>*n = 311	5	NR

For peer review only

For peer review only - http://bmjopen.bmj.com/site/about/guidelines.xhtml

Osmann et al., 2022	Cross-sectional	159	Journalists	Convenience	44.72 (12.1)	52%	NR	NR	72 %	Toronto Moral Injury Scale for Journalists 3 subscales (organisational, Individuals, and online)	(Mental) health PTSD (PLC-5) (+) Anxiety (BAI) (+) Depression (BDI-II) (+) Demographics Marital status (0) Education (0)	Overall % NR Subscale % NR	Overall M/SD 1.50(1.27) Subscale M/SD Organisational /management: 1.54(1.32) Individuals/Nonmanagement: 1.18(1.19) Online: 1.68(1.27)	5	None
Rodríguez et al., 2021	Cross-sectional	169	Healthcare Physicians (86.39%) Nurses (13.61%)	Convenience	36.04 (10.5)	23.7 %	NR	Honduras	NR	MISS-HP 36+	(Mental) health Depression (PHQ-9 (+) Anxiety (GAD-7) (+)	Overall % 77 (45.6%)	Overall M/SD 34.80(15.81)	5	None
Roth et al., 2022	Cross-sectional	270	Personal Safety Personnel (PSP). Firefighter (19%) Paramedic (44%) Police (17%) Dispatcher (6%) Other (14%)	Convenience	35 (9.5)	63%	85%	Canada and USA	NR	MIA-PSP 3 subscales (perpetration, betrayal and emotional sequalae) MIES Unidimensional.	(Mental) health Shame (TRSI) (+) Guilt (TRGI) (+) PTSD (PCL-5) (+) Depression, anxiety and stress (DASS-21) (+) Dissociation (MDI) (+) Disability, functioning and health (WHODAS) (+)	Overall % NR for either scale	Overall M/SD MIA-PSP 59.6(21.9) MIES 3.56(1.39) Subscale M/SD MIA-PSP Emotional Sequalae: 25(9.7) Perpetration: 15(8.5) Betrayal: 19.6(7.7)	5	None

For peer review only - http://bmjopen.bmj.com/site/about/guidelines.xhtml
For peer review only

													Subscale M/SD MIES NR		
Russell & Mussap, 2022<br><br>Two studies	Cross-sectional	Study 1: 183 Study 2: 393	Study 1:<br><br>Current and former military personnel.<br><br>Currently serving (50.3%)<br><br>Study 2:<br><br>Current and former military personnel.<br><br>Currently serving (39.7%)	Study 1 and 2: Convenience .<br><br>.	Study 1: 43.3 (11.9) Study 2: 44 (12)	Study 1: 83.6 % Study 2: 81.4 %	Study 1: 84.7% Study 2: 81.7%	Study 1: Global but mainly Australian (89%) Study 2: Global but primarily Australian (85%)	Study 1: NR Study 2: NR	Study 1: MIES 4+ (slightly agree)<br>3 subscales (transgressions-others, transgressions-self, betrayal)<br>Study 2: MIES 4+ (slightly agree)<br>3 subscales (transgressions-others, transgressions-self, betrayal)	Study 1:<br>(Mental) health<br>PTSD (PCL-M) (+)<br>Post-traumatic growth (PTGI-X) (0)<br>Personal well-being (PWI) (-)<br>Individual/work characteristics:<br>Intrusive rumination (ERRI) (+)<br>Deliberate rumination (ERRI) (+)<br>Exposure to danger/combat (EDC) (0) *<br>Exposure to danger/combat (EDC) (+) **<br>Witnessing the consequences of war (WDW) (+)<br>Demographics<br>Age (0)<br>Study 2:<br>(Mental) health<br>PTSD (PCL-M) (+)<br>Post-traumatic growth (PTGI-X) (0)	Study 1:<br>Overall % 132 (72.1%)<br>Subscale %<br>Transgressions-self: 97 (53%)<br>Transgressions-others: 48 (26.2%)<br>Betrayal: 99 (53.8%)<br>Study 2:<br>Overall % 300 (76.6%)<br>Subscale %<br>Transgressions-self: 109 (59.4%)<br>Transgressions-others: 62 (33.8%)<br>Betrayal: 112 (61.4%)	Study 1:<br>Overall M/SD: 2.25(1.12)<br>Subscale M/SD:<br>Transgressions-self: 2.88(1.63)<br>Transgressions-others: 1.73(1.20)<br>Betrayal: 2.54(1.45)<br>Study 2:<br>Overall M/SD: 2.60(1.28)<br>Subscale M/SD:<br>Transgressions-self: 3.23(1.74)<br>Transgressions-others: 2.05(1.42)<br>Betrayal: 2.9(1.58)	5	None

For peer review only - http://bmjopen.bmj.com/site/about/guidelines.xhtml

											Personal well-being (PWI) (-)<br>Individual/work characteristics:<br>Threat bias (IBIP) (+)<br>Exposure to danger/combat (EDC) (+) ***<br>Exposure to danger/combat (EDC) (0) ****<br>Witnessing the consequences of war (WDW) (+)<br>Demographics<br>Age (0)<br>*Transgressions-self<br>**Transgressions-other and betrayal<br>***Transgressions-self and transgressions-others<br>****Betrayal				
Saba et al., 2022	Longitudinal.<br><br>12-month time point.	1005	Veterans.<br>Air Force (14.53%)<br>Army (65.27%)<br>Marine Corps (9.65%)<br>Navy (10.55%	Convenience February 2021.	34.83 (3.61)	91.14 %	84.58%	NR	21.3 %	MIES 4+ (slightly agree)<br>3 subscales (transgressions-others, transgressions-self, betrayal)	(Mental) health<br>PTSD (PCL-5) (+)<br>Depression (PHQ-9) (+)<br>Anxiety (GAD-7) (+)<br>Anger (DAR-R) (+)	Overall %<br>881 (87.66%)<br>Subscale %<br>Transgressions-self: 802 (79.80%)<br>Transgressions-others: 303 (30.15%)<br>Betrayal: 300 (29.85%)	Overall M/SD: 2.75(0.81)<br>Subscale M/SD:<br>Transgressions-self: 2.65(1.20)<br>Transgressions-others: 2.92(0.84)<br>Betrayal: 2.58(1.11)	5	None

For peer review only - http://bmjopen.bmj.com/site/about/guidelines.xhtml

Senger, Torres & Ratcliff, 2022	Cross-sectional	242	First responders. In person sample: (n=91) Firefighter (37.4%) Law enforcement (20.90%) Paramedic (2.20%) EMT (4.40) Multiple (35.20%) Online sample (n=151): Firefighter (6%) Law enforcement (38.40%) Paramedic (16.60%) EMT (5.30%) Search and rescue worker (3.30%) Other (1.30%) Multiple (29.10%)	Convenience January – December 2020	NR	In person sample: 97.8 % Online sample: 75.50 %	Online sample: 83.30% In person sample: 88.70%	USA	NR	MIES 4+ (slightly agree) 3 subscales (transgressions-others, transgressions-self, betrayal)	Individual/work characteristics: Pre vs post COVID (-) Military service (-) Demographics Occupation type (+) Time in occupation (+) Age (+) Gender (0) Race/ethnicity (0) Education (0) Marital status (+) Income (0) Religion (0)	Overall % 98 (40.65%) Subscale % Transgressions -self: 51 (21.23%) Transgressions -others: 147 (60.75%) Betrayal: 97 (39.97%) Per item % Item 1: 174 (72.2%) Item 2: 119 (49.3%) Item 3: 48 (19.7%) Item 4: 56 (23.3%) Item 5: 53 (21.8%) Item 6: 49 (20.1%) Item 7: 102 (42%) Item 8: 94 (38.7%) Item 9: 95 (39.2%)	Overall M/SD: 2.7 (1.1) Subscale M/SD: Transgressions -self: 2.1 (1.3) Transgressions -others: 3.8 (1.4) Betrayal: 2.9 (1.5)	6

For peer review only - http://bmjopen.bmj.com/site/about/guidelines.xhtml

Stanojević & Čartolovni, 2022	Cross-sectional	162	Nurses. Palliative nurses (47.5%) Oncology nurses (52.5%)	Convenience January - June 2021.	NR	13%	NR	Croatia	64.8 %	MISS-HP 36+	(Mental) health: Moral distress (+) Individual/work characteristics: Left or considered leaving clinical position (+) Considering about leaving clinical position (+) Demographics Age (0) Work experience (0) Education (0) Gender (0) Specialisation (0)	Overall % NR	Overall M/SD 37.63(12.19)	5	None
Testoni et al., 2022	Cross-sectional	270	Healthcare professionals. Physician (65% Other healthcare provider (35%)	Convenience July - December 2020	44.5 (12.2)	30%	NR	Italy	NR	MIES 4+ (slightly agree) 3 subscales (transgressions-others, transgressions-self, betrayal)	(Mental) health: Alienation (+) Individual/work characteristics: Self-efficacy (NYPQS) (-) Humanisation patient (HTAS) (0) Humanisation colleague (HTAS) (-) Overall humanisation (HTAS) (-) Dehumanisation patient (HTAS) (+) Dehumanisation colleague (HTAS) (+) Overall dehumanisation (HTAS) (+)	Overall % 212 (78.5%) Subscale % Transgressions -self: 78 (28.9%) Transgressions -others: 166 (61.5%) Betrayal 166 (61.55)	Overall M/SD 2.62(1.18) Subscale M/SD Transgressions -self: 2.06(1.45) Transgressions -others: 3.23(1.56) Betrayal 2.96(1.48)	5	None

For peer review only - http://bmjopen.bmj.com/site/about/guidelines.xhtml

Thomas, Weiss, Forkus & Contractor, 2022	Cross-sectional	496	Veterans. Army (63.7%) Navy (9.9%) Air Force (19%) Marines (6.3%) Coast Guard (1.2%)	Convenience	37.80 (11.4)	70.5 %	71.1%	USA	18.8 %	MIES 3 subscales (transgressions-others, transgressions-self, betrayal)	(Mental) health: Alcohol misuse (AUDIT) (+) Individual/work characteristics: Total religiosity (DUREL) (+) Organisational religiosity (DUREL) (+) Nonorganizational religiosity (DUREL) (0) Intrinsic religiosity (DUREL) (0)	Overall % NR Subscale % NR	Overall M/SD 3.19(1.40) Subscale M/SD NR	5	NR
Trifunovic-Koenig et al., 2022	Cross-sectional	156	Healthcare Physician (16%) Nurse (general) (33.3%) Paediatric nurse (1.3%) Paramedic (23.7%) Geriatric nurse (9%) Speech therapist (clinical linguistic) (7.1%) Medical assistant (3.2%) Medical student (1.3%)	Convenience March - June 2021	40.7 (10.2)	34.6 %	NR	German speaking	3.5 %	MISS-HP 28.5+ 9 item scale	(Mental) health: Psychological distress (+) Physical distress (+) Individual/work characteristics: Colleague support (+) Supervisor support (+) Institutional support (+) Professional self-efficacy (+) Turnover intentions (+) Absenteeism (+) Resilience (+)	Overall % 85 (54.5%)	Overall M/SD 32.31(13.26)	5	None

For peer review only - http://bmjopen.bmj.com/site/about/guidelines.xhtml

			Physiotherapist (0.6%)<br>Midwife (0.6%)<br>Respiratory therapist (1.3%)<br>Administration worker/Technician (0.6%)												
Ulusoy & Çelik 2022	Cross-sectional	124	Healthcare workers.<br>Doctors (67.7%)<br>Nurses (21.8%)<br>Medical secretaries (4.8%)<br>Other support staff (5.6%)	Convenience<br>January - April 2021	NR	NR	NR	Turkey	36 %	MIES 4+ (slightly agree)<br>Unidimensional	(Mental) health<br>Burnout emotional exhaustion (MBI) (+)<br>Burnout depersonalisation (MBI) (+)<br>Burnout reduced personal accomplishment (MBI) (0)<br>Obstruction to valued living (VQ) (+)<br>Depression, anxiety, and stress (DASS-21) (+)<br>Psychological inflexibility and experiential avoidance (AAQ-II) (+)	Overall %<br>116 (93.5%)<br><br>Subscale %<br>Transgressions -self: 78 (62.9%)<br>Transgressions -others: 109 (87.9%)<br>Betrayal: 96 (77.4%)	Overall M/SD<br>3.42(0.99)<br><br>Subscale M/SD<br>Transgressions -self: 2.82(1.31)<br>Transgressions -others: 4.51(1.34)<br>Betrayal: 3.51(1.28)	5	None

For peer review only

For peer review only - http://bmjopen.bmj.com/site/about/guidelines.xhtml

Williamson Murphy & Greenberg, 2022	Cross-sectional	90	Veterinary	Convenience December 2020 – May 2021	36.8 (10.2)	12.2 %	70%	UK	27 %	MIES 5+ (moderately agree)<br>3 subscales (transgressions-others, transgressions-self, betrayal)	(Mental) health<br>PTSD (ITQ) (+)<br>Alcohol (AUDIT-C) (0)<br>Common mental disorders (PHQ-4)) (0)<br><br>Demographics<br>Age (0)<br>Years of experience (0)<br>Role type (0)	Overall %<br>80 (88.9%)<br><br>Subscale %<br><br>Transgressions-self: 51 (56.7%)<br>Transgressions-others: 63 (70%)<br>Betrayal: 62 (68.9%)	Overall M/SD<br>NR<br>Subscale M/SD<br>NR	5	None
Zahiriharsini et al., 2022<br><br>Narrative synthesis	Cross-sectional	572	Healthcare workers and leaders<br>Nurse (60.5%)<br>Patient support worker (2.9%)<br>Physician (1%)<br>Manager (11.1)<br>Others (25%)	Convenience February - June 2021	40.30 (10.5)	11.4 %	NR	Canada Quebec	NR	EMIS-M<br>low MI: <2.5, moderate MI: 2.5 ≤ score <3.5, and high MI: ≤ 3.5<br><br>2 subscales (Self-directed MI and other-directed MI)	NR at univariate level	Overall %<br>Moderate to high: 79 (13.81%)<br>Subscale %<br>Self-directed MI:<br>Moderate to high: 58 (10.14%)<br>Other-directed MI:<br>Moderate to high: 167 (29.19%)	Overall M/SD:<br>NR<br>Subscale M/SD:<br>NR	5	None

For peer review only

Levi-Belz Shemesh & Zerach 2022	Cross-sectional	190	Combat veterans. Infantry units (58.4%) Armoured corps (13.2%) Artillery corps (7.4%) Navy (4.7%) Military police, intelligence, or Air Force (13.6%)	Convenience<br><br>January - July 2018.	26 (2.61)	87%	NR	Israel	NR	MIES 4+ (slightly agree)<br><br>3 subscales (transgressions-others, transgressions-self, betrayal)<br><br>Average time since discharge 4.26(2.37)	(Mental) health Depression (PHQ-8) (+) Current suicide ideation (SBQ-R) (+) * current suicide ideation (SBQ-R) (0) ** Self-disclosure (DDI) (0) Individual/work characteristics Combat exposure (+) Time from demobilisation (0) *Transgressions-self and betrayal **Transgressions-others	Overall % NR Per subscale % Transgressions-others: 69 (36.1%) Transgressions-self: 44 (23.2%) Betrayal: 67 (35.2%) Per item % Item 1: 61 (32.1%) Item 2: 47 (24.7%) Item 7: 52 (27.4%)	Overall M/SD NR Subscale M/SD Transgressions-others: 2.41(1.49) Transgressions-self: 1.81(1.08) Betrayal: 2.02(1.06)	5	NR
Schwartz Halperin & Levi-Belz, 2022	Cross-sectional	336	Veterans<br><br>Armoured corps (9.2%) Artillery corps (8.3%) Combat engineering (8%) Other (34.6%)	Convenience<br><br>August - November 2019	26.16 (3.06)	83.9 %	NR	Israel	58 %	MIES 4+ (slightly agree)<br><br>1 subscale (transgressions-self)<br><br>Average time since discharge 4.2(2.45)	(Mental) health Shame (TRSI) (+) Current suicidal ideation (SBQ-R) (+) Individual/work characteristics Collective hatred (HAS) (0) All correlations are for transgressions-self subscale.	Overall % NR Per subscale % NR Per item % Item 3: (24.18%)	Overall M/SD NR Subscale M/SD Transgressions-self: 2.12(1.26)	5	None

Note. For longitudinal studies, data extracted from baseline wave unless only available at a subsequent wave. Associations reported use total scale but if no total scale associations are reported, then subscales associations were reported. CPS = Child Protection Services; RR = Response rate; NR = No response; MIES = Moral injury events scales; EMIS-M = Expressions of Moral Injury Scale-Military Version; MISS-HP = Moral Injury Symptoms Scale – Health Professionals; MIA-PSP = Moral Injury Assessment for Public Safety Personnel; MIQ-M = Moral Injury Questionnaire—Military version, BMIS = Brief Moral Injury Screen; BMIS-E = Brief Moral Injury Screen Event subscale, EMIS-SF = Expressions of Moral Injury Scale-Short Form; MIQ-T = Moral Injury Questionnaire—Teacher version; MIQ-M = Moral Injury Questionnaire—Military version; MIOS = Moral Injury Outcome Scale

For peer review only - http://bmjopen.bmj.com/site/about/guidelines.xhtml

Supplementary Table 3.
Hoy Risk of Bias for studies included in the systematic review

Authors (Year)	Representative of national population	Representative of target population	Random selection	Non-response bias	Directly collected	Acceptable case definition	Reliable and valid instrument	Same mode of data collection	Appropriate numerator(s) and denominator(s) for the parameter of interest	Hoy score
**PTED only studies**										
Dunn & Sensky 2018	1	1	1	1	0	0	0	0	1	5
Linden & Rotter 2019	1	1	1	1	0	0	0	0	1	5
Michailidis & Cropley 2018	1	1	1	1	0	0	0	0	1	5
Sensky, Salimu, Ballard & Pereira 2015	1	0	1	1	0	0	0	0	1	4
Sabic, Sabic, & O Baltic-Mujanovic 2018	1	1	1	1	0	0	0	0	1	5
Michailids & Cropley 2017	1	1	1	1	0	0	0	0	1	5
Karatuna & Gok 2014	1	1	1	1	0	0	0	0	1	5
Rubab & Tariq 2022	1	1	1	1	0	0	0	0	1	5
Saleem, Bashir & Abrar 2022	1	1	0	1	0	0	0	1	1	5
Shin & You 2021	1	1	1	1	0	0	0	0	1	5
**Moral injury and PTED study**										

For peer review only

For peer review only - http://bmjopen.bmj.com/site/about/guidelines.xhtml

Brennan, McKay & Cole 2022	1	0	1	0	0	0	0	0	0	2
**Moral injury only studies**										
Bryan et al., 2016	1	1	1	1	0	0	0	0	1	5
Currier, Holland, Drescher & Foy 2015	0	0	1	1	0	0	0	0	1	3
Currier, Holland, Rojas-Flores, Herrera & Foy 2015	1	1	1	0	0	0	0	0	1	4
Currier et al., 2017	1	1	1	1	0	0	0	0	1	5
Forkus,Juliana & Weiss 2019	1	1	1	1	0	0	0	0	1	5
Frankfurt et al., 2018.	1	0	1	1	0	0	0	0	1	4
Griffin et al., 2020	1	1	1	1	0	0	0	0	1	5
Jinkerson & Battles 2019	1	1	1	0	0	0	0	0	1	4
Jordan & Nash 2017	1	1	1	1	0	0	0	0	1	5
Lee, Aldwin & Kang 2022	1	1	1	1	0	0	0	0	1	5
Ogle, Reichwald, & Rutland 2018	1	1	1	1	0	0	0	0	1	5
Wisco et al., 2017	0	0	0	1	0	0	0	0	0	1
Zerach & Levi-Belz., 2017	1	1	1	0	0	0	0	0	1	4
Papazoglou et al., 2019	1	1	1	0	0	0	0	0	1	4
Richardson, Chesnut et al., 2020	0	0	0	1	0	0	0	0	1	2
Feinstein, Pavisian, Storm., 2018	1	1	1	1	0	0	0	0	1	5
Sugrue 2020	1	1	1	1	0	0	0	0	1	5

For peer review only - http://bmjopen.bmj.com/site/about/guidelines.xhtml

For peer review only

Aldridge, Scott, Paskell 2019	1	1	1	1	0	0	0	0	1	5
Andrukonis, Protopopov. 2020	1	1	1	1	0	0	0	0	1	5
Haight, Sugrue & Calhoun 2017	1	1	1	1	0	0	0	0	1	5
Battles et al., 2019	1	1	1	1	0	0	0	0	1	5
Braitman et al., 2018	1	1	1	1	0	0	0	0	1	5
Lancaster & Harris 2018	1	1	1	1	0	0	0	0	1	5
Lancaster 2018	1	1	1	1	0	0	0	0	1	5
Akhtar et al., 2022	1	1	1	1	0	0	0	1	1	6
Qi et al., 2022	1	1	1	1	0	0	0	0	1	5
Ansalem et al., 2021	1	1	1	1	0	0	0	0	1	5
Perez, Larson & Bair 2021	1	1	1	1	0	0	0	0	1	5
Benatoz, Zerach & Levi-Belz 2022	1	1	1	1	0	0	0	0	1	5
Borges et al., 2021	1	1	1	1	0	0	0	0	1	5
Boscarino et al., 2022	1	0	0	1	0	0	0	0	1	3
Brady et al., 2021	0	0	1	1	0	0	0	0	1	4
Brady et al., 2022	1	1	1	1	0	0	0	0	1	6
Chandrabhatla et al., 2022	1	1	1	1	0	0	0	0	1	5
Dale et al., 2021	1	1	1	1	0	0	0	0	1	5
Plouffe et al., 2021	0	0	0	1	0	0	0	0	1	2

For peer review only

For peer review only - http://bmjopen.bmj.com/site/about/guidelines.xhtml

Nichter et al., 2020	0	0	0	1	0	0	0	0	0	1
Fitzpatrick et al., 2022	1	1	1	1	0	0	0	0	1	5
Hagerty & Williams 2022	1	1	1	1	0	0	0	0	1	5
Hamrick et al., 2022	1	1	1	1	0	0	0	0	1	5
Hines et al., 2020	1	1	1	1	0	0	0	0	1	5
Hinkel et al., 2022	1	1	1	1	0	0	0	0	1	5
Kelley et al., 2021	1	1	1	1	0	0	0	0	1	5
La Fleur et al., 2020	1	1	1	1	0	0	0	1	1	6
Lamb et al., 2021	1	0	0	1	0	0	0	0	0	2
Litam et al., 2021	1	1	1	1	0	0	0	0	1	5
Litz et al., 2022 Samples 1, 2 and 3	1	1	1	1	0	0	0	0	1	5
Litz et al., 2022 Sample 4	1	1	1	1	0	0	0	1	1	6
Maftei & Holman 2021	1	1	1	1	0	0	0	0	1	5
Maguen et al., 2021	0	0	0	1	0	0	0	0	1	2
Malakoutikhah et al., 2022	1	1	1	1	0	0	0	0	1	5
Mantri et al., 2020	1	1	1	1	0	0	0	0	1	5
Mantri et al., 2021	1	1	1	1	0	0	0	0	1	5
Morris, Webb & Devlin 2022	1	1	1	1	0	0	0	0	1	5

For peer review only - http://bmjopen.bmj.com/site/about/guidelines.xhtml

Rushton et al., 2022	1	1	1	1	0	0	0	0	1	5
Nieuwsma et al., 2020	1	1	1	1	0	0	0	0	1	5
Osmann et al., 2022	1	1	1	1	0	0	0	0	1	5
Rodríguez et al., 2021	1	1	1	1	0	0	0	0	1	5
Roth et al., 2022	1	1	1	1	0	0	0	0	1	5
Russel & Mussap 2022	1	1	1	1	0	0	0	0	1	5
Saba et al., 2022	1	1	1	1	0	0	0	0	1	5
Senger, Torres & Ratcliff 2022	1	1	1	1	0	0	0	1	1	6
Stanojević & Čartolovni 2022	1	1	1	1	0	0	0	0	1	5
Testoni et al., 2022	1	1	1	1	0	0	0	0	1	5
Thomas et al., 2022	1	1	1	1	0	0	0	0	1	5
Trifunovic-Koenig, et al 2022	1	1	1	1	0	0	0	0	1	5
Ulusoy & Çelik 2022	1	1	1	1	0	0	0	0	1	5
Williamson, Murphy & Greenberg 2022	1	1	1	1	0	0	0	0	1	5
Zahiriharsini et al., 2022	1	1	1	1	0	0	0	0	1	5
Levi-Belz, Shemesh & Zerach 2022	1	1	1	1	0	0	0	0	1	5
Schwartz, Halperin & Levi-Belz 2022	1	1	1	1	0	0	0	0	1	5

Note. 1 = yes (high risk), 0 = no (low risk) 7-9 = High, 4-6 = Medium, 0-3 = Low. PTED = post-traumatic embitterment disorder

For peer review only - http://bmjopen.bmj.com/site/about/guidelines.xhtml

For peer review only

Supplementary Figures: Forest Plots for Meta-Analysis Findings

*Analyses using **prevalence** estimates as outcome*

Supplementary Figure 1.

PTED pooled prevalence estimates, using 1.6 cut off.

Study	Cases	Total	Prevalence	95% C.I.
Brennan 2022	121	400	30.25	[25.78; 35.01]
Dunn 2018	37	79	46.84	[35.51; 58.40]
Katatuna 2014	106	397	26.70	[22.41; 31.34]
Michailids 2018	55	352	15.62	[11.99; 19.85]
Michailids 2017	253	337	75.07	[70.10; 79.60]
Rubab 2022	121	300	40.33	[34.74; 46.12]
Sabic 2018	15	87	17.24	[9.98; 26.84]
Sensky 2015	68	236	28.81	[23.12; 35.05]
Shin 2022	581	1074	54.10	[51.06; 57.11]
**Random effects model**		**.**	**36.65**	**[24.38; 49.85]**

Heterogeneity: I^2 = 98%, t^2 = 0.0403, c_8^2 = 455.07 (p < 0.01)

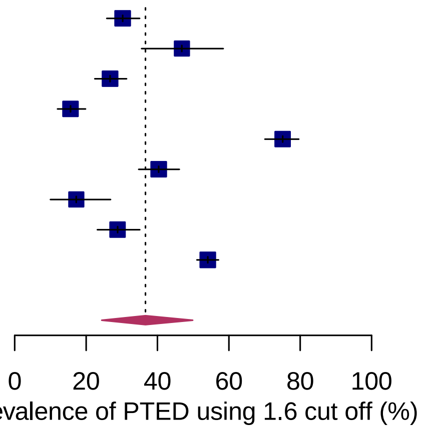

For peer review only

Supplementary Figure 2.

PTED pooled prevalence estimates, using 2 cut off.

Study	Cases	Total	Prevalence	95% C.I.
Brennan 2022	77	400	19.25	[15.50; 23.46]
Dunn 2018	28	79	35.44	[25.00; 47.01]
Katatuna 2014	74	397	18.64	[14.93; 22.83]
Linden 2019	26	102	25.49	[17.38; 35.08]
Michailidis 2017	209	337	62.02	[56.60; 67.22]
Rubab 2022	77	300	25.67	[20.82; 31.00]
Sabic 2018	9	87	10.34	[4.84; 18.73]
Sensky 2015	43	236	18.22	[13.51; 23.75]
**Random effects model**		**.**	**26.03**	**[16.09; 37.38]**

Heterogeneity: I^2 = 97%, t^2 = 0.0293, c_7^2 = 232.77 (p < 0.01)

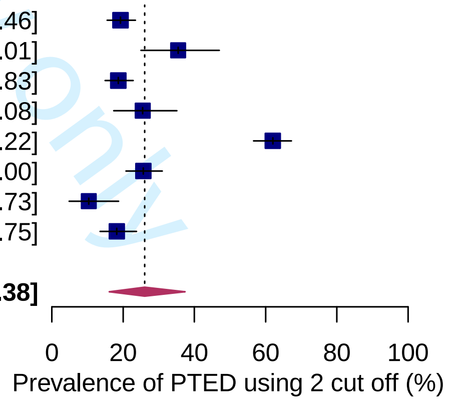

For peer review only - http://bmjopen.bmj.com/site/about/guidelines.xhtml

Supplementary Figure 3.
PTED pooled prevalence estimates, using 2.5 cut off.

Study	Cases	Total	Prevalence	95% C.I.
Brennan 2022	30	400	7.50	[5.12; 10.53]
Katatuna 2014	41	397	10.33	[7.51; 13.75]
Michailidis 2017	152	337	45.10	[39.71; 50.59]
Rubab 2022	26	300	8.67	[5.74; 12.44]
Sabic 2018	1	87	1.15	[0.03; 6.24]
Sensky 2015	21	236	8.90	[5.59; 13.28]
Shin 2022	102	1074	9.50	[7.81; 11.41]
**Random effects model**		**.**	**11.21**	**[4.02; 21.32]**

Heterogeneity: $I^2 = 97\%$, $t^2 = 0.0334$, $c^2_6 = 237.59$ ($p < 0.01$)

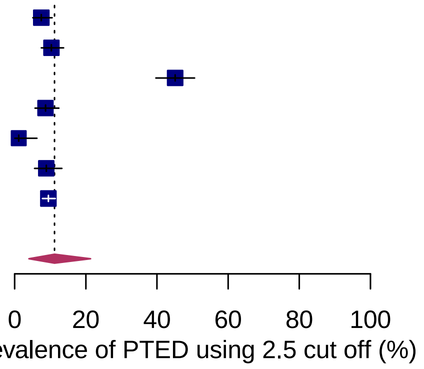

For peer review only

Supplementary Figure 4.
Any MIES item pooled prevalence estimates, split by occupation.

Study	Cases	Total	Prevalence	95% C.I.
Healthcare				
Amsalem 2021	267	350	76.29	[71.47; 80.65]
Borges 2021	109	211	51.66	[44.70; 58.57]
Brady 2021	237	390	60.77	[55.73; 65.65]
Brady 2022	268	377	71.09	[66.23; 75.62]
Brennan 2022	289	400	72.25	[67.58; 76.58]
Chandrabhatla 2022	60	78	76.92	[66.00; 85.71]
Lamb 2021	602	2166	27.79	[25.91; 29.73]
Maftei 2021	80	114	70.18	[60.89; 78.38]
Morris 2022	183	233	78.54	[72.71; 83.63]
Testoni 2022	212	270	78.52	[73.13; 83.27]
Ulusoy 2022	116	124	93.55	[87.68; 97.17]
Williamson 2022	80	90	88.89	[80.51; 94.54]
Random effects model		.	**71.40**	**[60.72; 81.01]**
Heterogeneity: $I^2 = 99\%$, $t^2 = 0.0382$, $c^2_{11} = 1160.88$ ($p < 0.01$)				
Armed forces				
Lee 2020	176	354	49.72	[44.39; 55.05]
Maguen 2022	7830	14057	55.70	[54.88; 56.53]
Nichter 2021	535	1321	40.50	[37.84; 43.20]
Russell 2022 study one	132	183	72.13	[65.04; 78.49]
Russell 2022 study two	300	393	76.34	[71.82; 80.45]
Saba 2022	881	1005	87.66	[85.47; 89.63]
Wisco 2017	223	564	39.54	[35.48; 43.71]
Zerach 2017	92	191	48.17	[40.90; 55.50]
Random effects model		.	**59.53**	**[45.90; 72.44]**
Heterogeneity: $I^2 = 99\%$, $t^2 = 0.0382$, $c^2_7 = 830.68$ ($p < 0.01$)				
First responders				
Papazoglou 2019	289	390	74.10	[69.45; 78.38]
Senger 2022	98	242	40.50	[34.26; 46.97]
Random effects model		.	**57.86**	**[30.91; 82.51]**
Heterogeneity: $I^2 = 99\%$, $t^2 = 0.0382$, $c^2_1 = 71.65$ ($p < 0.01$)				
Education				
Sugrue 2020	191	218	87.61	[82.49; 91.68]
**Random effects model**		**.**	**67.00**	**[59.01; 74.54]**

Heterogeneity: $I^2 = 99\%$, $t^2 = 0.0398$, $c^2_{22} = 2212.51$ ($p = 0$)
Residual heterogeneity: $I^2 = 99\%$, $t^2 = 0.0382$, $c^2_{19} = 2063.21$ ($p = 0$)
Test for subgroup differences: $c^2_3 = 3.95$, df = 3 ($p = 0.27$)

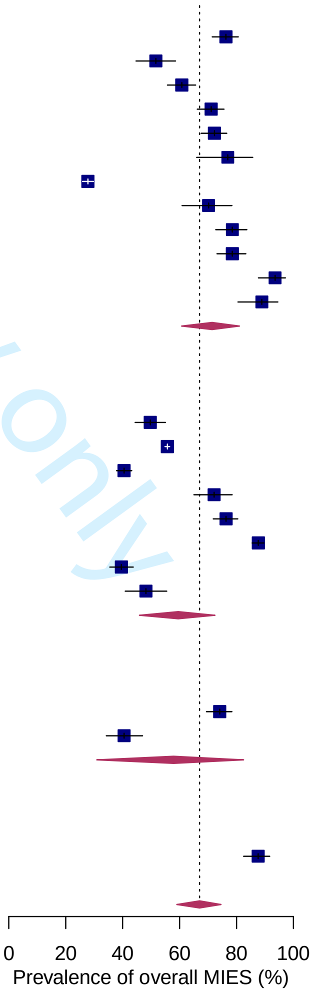

Supplementary Figure 5.

MIES pooled perpetration prevalence estimates.

Study	Cases	Total	Prevalence	95% C.I.
Borges 2021	77	211	36.49	[29.99; 43.38]
Dale 2021	21	265	7.92	[4.97; 11.86]
**Random effects model**		**.**	**20.26**	**[1.11; 53.70]**

Heterogeneity: I^2 = 98%, t^2 = 0.0641, c^2_1 = 61.38 (p < 0.01)

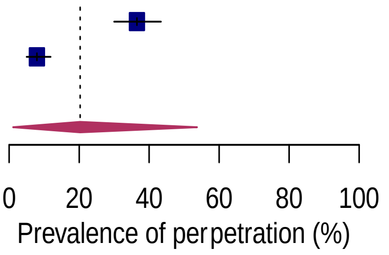

Supplementary Figure 6.

MIES transgressions-self pooled prevalence estimates, split by occupation.

Study	Cases	Total	Prevalence	95% C.I.
Healthcare				
Amsalem 2021	101	350	28.86	[24.16; 33.91]
Benatov 2022	94	296	31.76	[26.49; 37.39]
Brady 2021	92	390	23.59	[19.46; 28.12]
Brady 2022	92	377	24.40	[20.15; 29.06]
Brennan 2022	131	400	32.75	[28.17; 37.59]
Chandrabhatla 2022	23	78	29.49	[19.70; 40.89]
Dale 2021	61	265	23.02	[18.09; 28.56]
Maftei 2021	31	114	27.19	[19.28; 36.33]
Morris 2022	79	230	34.35	[28.23; 40.87]
Testoni 2022	78	270	28.89	[23.56; 34.69]
Ulusoy 2022	78	124	62.90	[53.77; 71.40]
Williamson 2022	51	90	56.67	[45.80; 67.08]
Random effects model		.	**33.16**	**[23.24; 43.90]**
Heterogeneity: I^2 = 90%, t^2 = 0.0364, c^2_{11} = 107.54 (p < 0.01)				
Armed forces				
Bryan 2016	508	935	54.33	[51.08; 57.56]
Jordan 2017	214	867	24.68	[21.85; 27.69]
Lee 2020	115	354	32.49	[27.63; 37.64]
Levi−Belz 2022	44	190	23.16	[17.36; 29.81]
Maguen 2022	3064	14057	21.80	[21.12; 22.49]
Nichter 2021	152	1321	11.51	[9.83; 13.35]
Richardson 2020	516	5227	9.87	[9.08; 10.71]
Russell 2022 study one	97	183	53.01	[45.50; 60.41]
Russell 2022 study two	109	393	27.74	[23.36; 32.45]
Saba 2022	802	1005	79.80	[77.18; 82.24]
Wisco 2017	48	564	8.51	[6.34; 11.13]
Zerach 2017	42	191	21.99	[16.33; 28.54]
Random effects model		.	**29.30**	**[19.94; 39.64]**
Heterogeneity: I^2 = 100%, t^2 = 0.0364, c^2_{11} = 2850.04 (p = 0)				
CPS				
Haight 2017	9	38	23.68	[11.44; 40.24]
First responders				
Senger 022	51	242	21.07	[16.11; 26.76]
Education				
Sugrue 2020	93	218	42.66	[36.01; 49.52]
**Random effects model**		**.**	**30.95**	**[24.72; 37.54]**

Heterogeneity: I^2 = 99%, t^2 = 0.0325, c^2_{26} = 3105.90 (p = 0)
Residual heterogeneity: I^2 = 99%, t^2 = 0.0364, c^2_{22} = 2957.58 (p = 0)
Test for subgroup differences: c^2_4 = 1.15, df = 4 (p = 0.89)

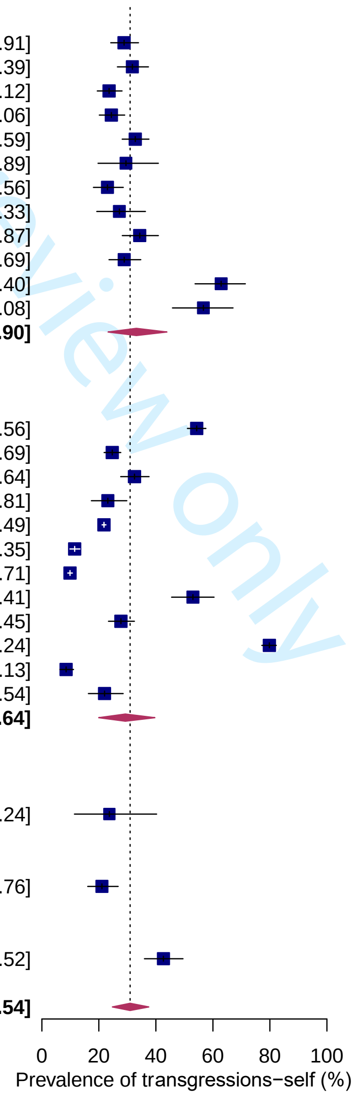

For peer review only

Supplementary Figure 7.

MIES transgressions-others pooled prevalence estimates, split by occupation.

Study	Cases	Total	Prevalence	95% C.I.
**Healthcare**				
Amsalem 2021	215	350	61.43	[56.11; 66.56]
Benatov 2022	146	296	49.32	[43.49; 55.17]
Brady 2021	189	390	48.46	[43.40; 53.55]
Brady 2022	197	377	52.25	[47.08; 57.39]
Brennan 2022	213	400	53.25	[48.23; 58.22]
Chandrabhatla 2022	49	77	63.64	[51.88; 74.30]
Dale 2021	6	265	2.26	[0.84; 4.86]
Maftei 2021	61	114	53.51	[43.93; 62.90]
Morris 2022	154	230	66.96	[60.47; 73.00]
Testoni 2022	166	270	61.48	[55.39; 67.32]
Ulusoy 2022	109	124	87.90	[80.83; 93.07]
Williamson 2022	63	90	70.00	[59.43; 79.21]
Random effects model		.	**54.88**	**[43.91; 65.62]**
Heterogeneity: I^2 = 98%, t^2 = 0.0360, c^2_{11} = 591.65 (p < 0.01)				
**Armed forces**				
Bryan 2016	363	935	38.82	[35.69; 42.03]
Lee 2020	130	354	36.72	[31.69; 41.98]
Levi−Belz 2022	69	190	36.32	[29.48; 43.59]
Maguen 2022	6059	14057	43.10	[42.28; 43.93]
Nichter 2021	316	1321	23.92	[21.64; 26.32]
Russell 2022 study one	48	183	26.23	[20.02; 33.23]
Russell 2022 study two	62	393	15.78	[12.31; 19.76]
Saba 2022	303	1005	30.15	[27.32; 33.09]
Wisco 2017	126	564	22.34	[18.97; 26.01]
Zerach 2017	64	191	33.51	[26.86; 40.68]
Random effects model		.	**30.36**	**[20.07; 41.76]**
Heterogeneity: I^2 = 98%, t^2 = 0.0360, c^2_{9} = 475.46 (p < 0.01)				
**CPS**				
Haight 2017	35	38	92.11	[78.62; 98.34]
**First responders**				
Senger 2022	147	242	60.74	[54.28; 66.94]
**Education**				
Sugrue 2020	175	218	80.28	[74.37; 85.34]
**Random effects model**		.	**47.73**	**[38.04; 57.51]**

Heterogeneity: I^2 = 98%, t^2 = 0.0607, c^2_{24} = 1464.59 (p < 0.01)
Residual heterogeneity: I^2 = 98%, t^2 = 0.0360, c^2_{20} = 1067.12 (p < 0.01)
Test for subgroup differences: c^2_4 = 20.01, df = 4 (p < 0.01)

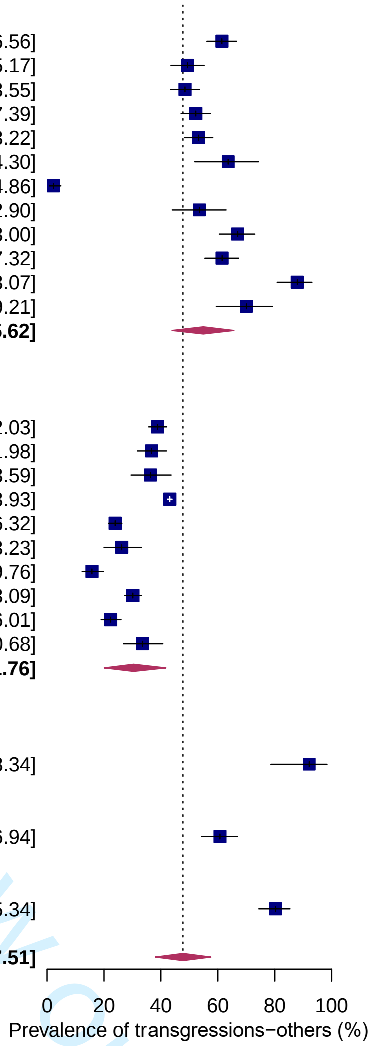

For peer review only

Supplementary Figure 8.

MIES betrayal pooled prevalence estimates, split by occupation.

Study	Cases	Total	Prevalence	95% C.I.
**Healthcare**				
Amsalem 2021	205	350	58.57	[53.21; 63.78]
Benatov 2022	184	296	62.16	[56.37; 67.71]
Borges 2021	88	211	41.71	[34.98; 48.67]
Brady 2021	166	390	42.56	[37.60; 47.64]
Brady 2022	210	377	55.70	[50.53; 60.79]
Brennan 2022	227	400	56.75	[51.74; 61.66]
Chandrabhatla 2022	37	78	47.44	[36.01; 59.07]
Maftei 2021	66	114	57.89	[48.29; 67.08]
Morris 2022	154	233	66.09	[59.63; 72.15]
Testoni 2022	166	270	61.48	[55.39; 67.32]
Ulusoy 2022	96	124	77.42	[69.04; 84.44]
Williamson 2022	62	90	68.89	[58.26; 78.23]
**Random effects model**		.	**58.12**	**[50.96; 65.12]**
Heterogeneity: $I^2 = 89\%$, $t^2 = 0.0147$, $c^2_{11} = 98.31$ ($p < 0.01$)				
**Armed forces**				
Bryan 2016	540	935	57.75	[54.51; 60.94]
Jordan 2017	252	867	29.07	[26.06; 32.21]
Lamb 2021	439	2166	20.27	[18.59; 22.02]
Lee 2020	121	354	34.18	[29.25; 39.38]
Levi−Belz 2022	67	190	35.26	[28.49; 42.51]
Maguen 2022	5651	14057	40.20	[39.39; 41.02]
Nichter 2021	189	1321	14.31	[12.46; 16.31]
Russell 2022 study one	99	183	54.10	[46.59; 61.47]
Russell 2022 study two	112	393	28.50	[24.09; 33.24]
Saba 2022	300	1005	29.85	[27.03; 32.79]
Wisco 2017	142	564	25.18	[21.64; 28.97]
Zerach 2017	59	191	30.89	[24.42; 37.97]
**Random effects model**		.	**32.67**	**[26.29; 39.38]**
Heterogeneity: $I^2 = 99\%$, $t^2 = 0.0147$, $c^2_{11} = 1001.5$ ($p < 0.01$)				
**CPS**				
Haight 2017	12	38	31.58	[17.50; 48.65]
**First responders**				
Papazoglou 2019	250	390	64.10	[59.12; 68.87]
Senger 2022	97	242	40.08	[33.86; 46.55]
**Random effects model**		.	**52.32**	**[35.23; 69.13]**
Heterogeneity: $I^2 = 97\%$, $t^2 = 0.0147$, $c^2_{1} = 35.05$ ($p < 0.01$)				
**Education**				
Sugrue 2020	148	218	67.89	[61.25; 74.04]
**Random effects model**		.	**46.03**	**[39.54; 52.59]**

Heterogeneity: $I^2 = 98\%$, $t^2 = 0.0300$, $c^2_{27} = 1764.34$ ($p = 0$)
Residual heterogeneity: $I^2 = 98\%$, $t^2 = 0.0147$, $c^2_{23} = 1134.87$ ($p < 0.01$)
Test for subgroup differences: $c^2_4 = 30.29$, df = 4 ($p < 0.01$)

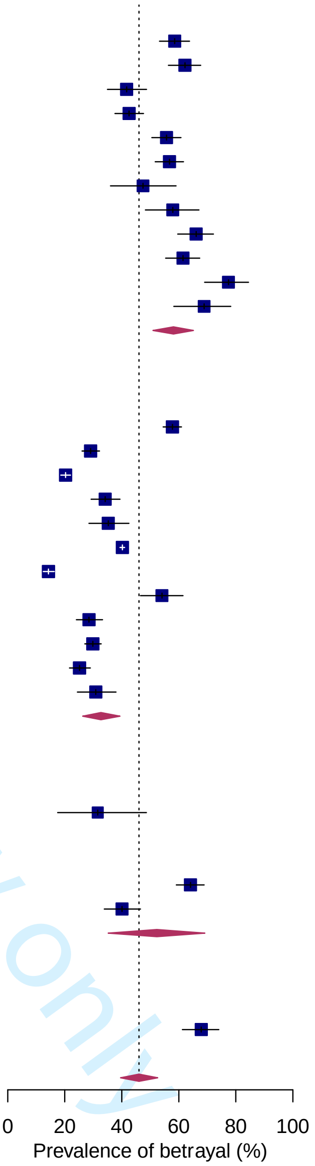

For peer review only

Supplementary Figure 9.

MISS-HP pooled prevalence estimates.

Study	Cases	Total	Prevalence	95% C.I.
Akhtar 2022	75	108	69.44	[59.84; 77.95]
Benatov 2022	120	296	40.54	[34.90; 46.37]
Malakoutikhah 2022	190	455	41.76	[37.18; 46.44]
Mantri 2020	86	181	47.51	[40.06; 55.05]
Mantri 2021 sample one	84	450	18.67	[15.17; 22.58]
Mantri 2021 sample two	591	1381	42.80	[40.17; 45.45]
Qi 2022 study one	1695	3006	56.39	[54.59; 58.17]
Qi 2022 study two	1270	3465	36.65	[35.05; 38.28]
Rodríguez 2021	77	169	45.56	[37.90; 53.39]
Trifunovic−Koenig 2022	85	156	54.49	[46.33; 62.47]
**Random effects model**		**.**	**44.88**	**[36.40; 53.50]**

Heterogeneity: $I^2 = 98\%$, $t^2 = 0.0184$, $c^2_9 = 442.10$ ($p < 0.01$)

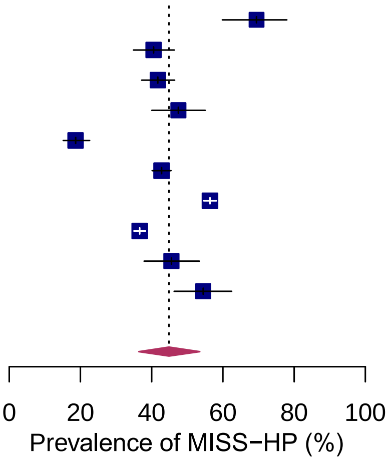

*Analyses using **pooled mean** estimates for PTED and moral injury, split by occupation*

Supplementary Figure 10.

PTED pooled mean estimates, split by occupation.

Study	N	SD	Mean	95% C.I.	Weight
**Healthcare**					
Brennan 2022	400	0.90	1.12	[1.03; 1.21]	9.1%
Dunn 2018	79	1.10	1.50	[1.26; 1.74]	8.9%
Sensky 2015	233	0.97	1.11	[0.99; 1.23]	9.1%
Random effects model			**1.24**	**[0.46; 2.02]**	**27.1%**
Heterogeneity: $I^2 = 77\%$, $t^2 = 0.47$, $c^2_2 = 8.83$ ($p = 0.01$)					
**Public sector**					
Karatuna 2014	397	1.04	1.00	[0.90; 1.10]	9.1%
**Unemployed**					
Linden 2019	102	0.92	1.24	[1.06; 1.42]	9.0%
Shin 2022	1074	0.70	1.64	[1.60; 1.68]	9.2%
Random effects model			**1.44**	**[0.49; 2.39]**	**18.2%**
Heterogeneity: $I^2 = 95\%$, $t^2 = 0.47$, $c^2_1 = 18.28$ ($p < 0.01$)					
**Mixed**					
Michailids 2017	337	0.96	2.44	[2.34; 2.54]	9.1%
Michailids 2018	352	0.92	1.28	[1.18; 1.38]	9.1%
Random effects model			**1.86**	**[0.91; 2.81]**	**18.3%**
Heterogeneity: $I^2 = 100\%$, $t^2 = 0.47$, $c^2_1 = 261.83$ ($p < 0.01$)					
**Education**					
Rubab 2022	300	0.70	1.44	[1.36; 1.52]	9.1%
Saleem 2022	398	1.12	3.16	[3.05; 3.27]	9.1%
Random effects model			**2.30**	**[1.35; 3.25]**	**18.3%**
Heterogeneity: $I^2 = 100\%$, $t^2 = 0.47$, $c^2_1 = 618.25$ ($p < 0.01$)					
**Veterans**					
Sabic 2018	87	0.73	0.75	[0.60; 0.90]	9.1%
**Random effects model**			**1.52**	**[1.10; 1.93]**	**100.0%**

Heterogeneity: $I^2 = 99\%$, $t^2 = 0.49$, $c^2_{10} = 1529.32$ ($p < 0.01$)

Residual heterogeneity: $I^2 = 99\%$, $t^2 = 0.47$, $c^2_5 = 907.19$ ($p < 0.01$)

Test for subgroup differences: $c^2_5 = 5.44$, df = 5 ($p = 0.36$)

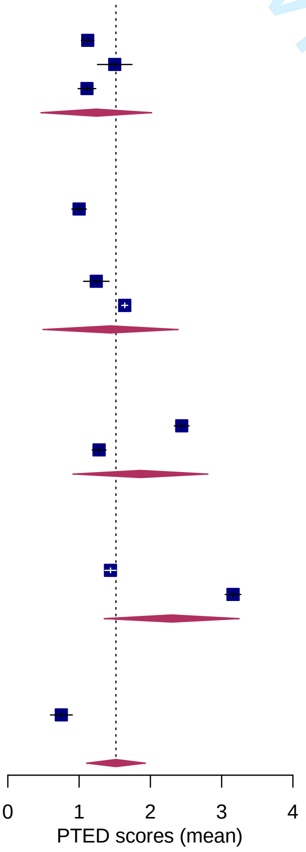

For peer review only

Supplementary Figure 11.
Overall MIES pooled mean estimates, split by occupation.

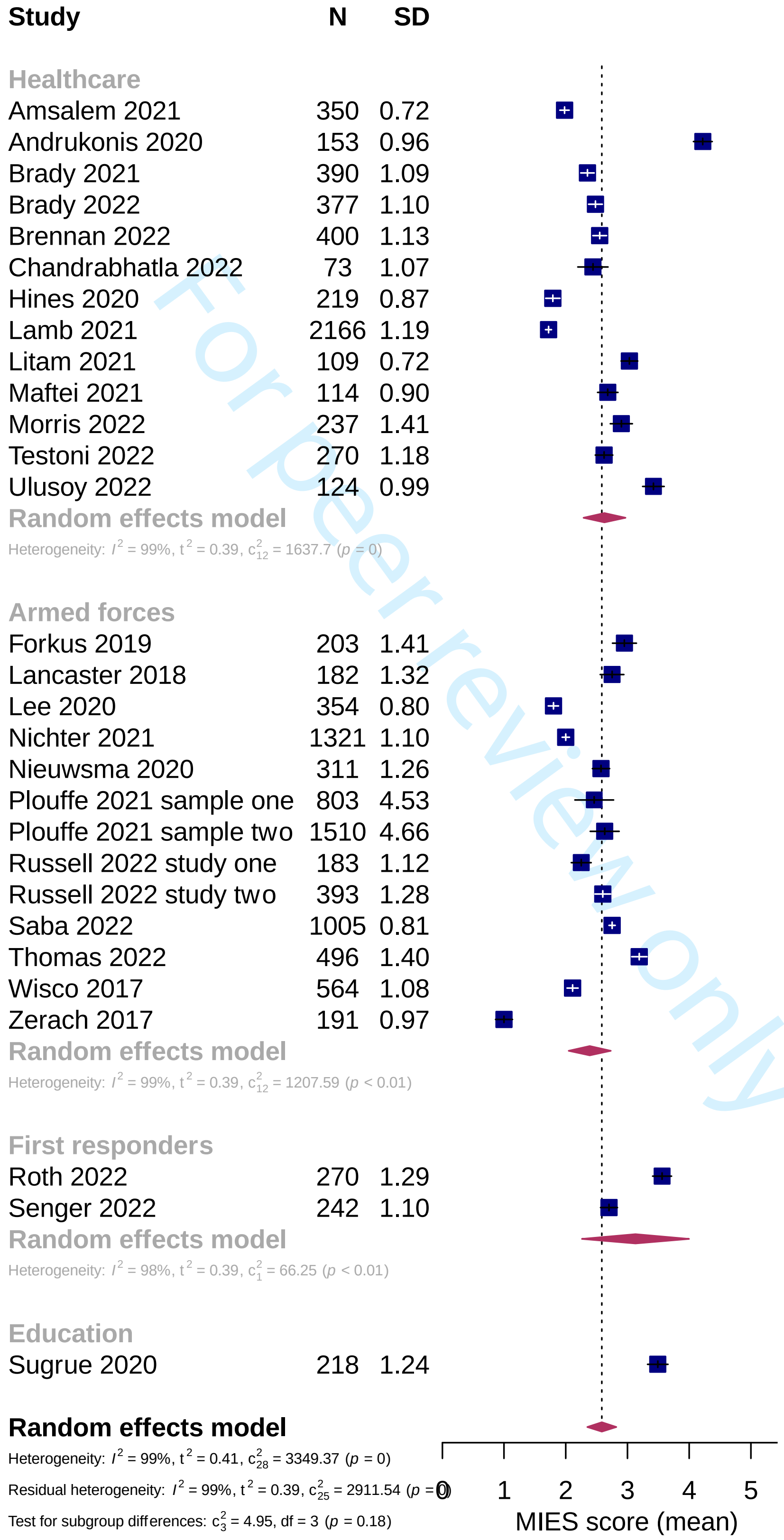

For peer review only - http://bmjopen.bmj.com/site/about/guidelines.xhtml

Supplementary Figure 12.

MIES perpetration pooled mean estimates.

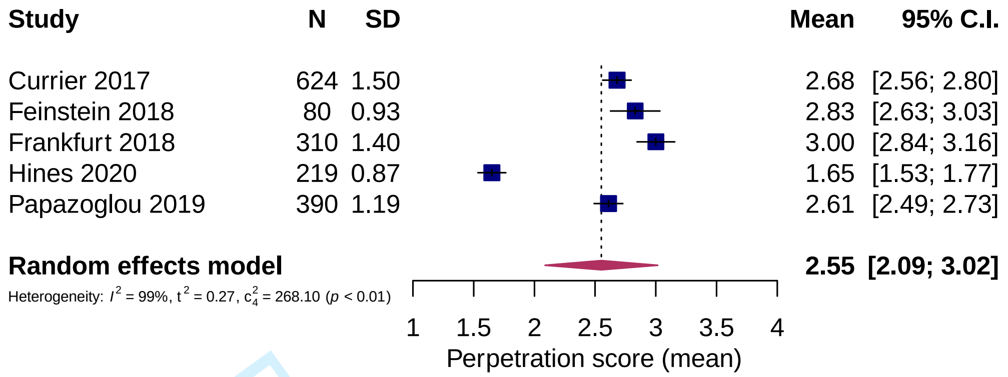

Supplementary Figure 13.

MIES transgressions-self pooled mean estimates, split by occupation.

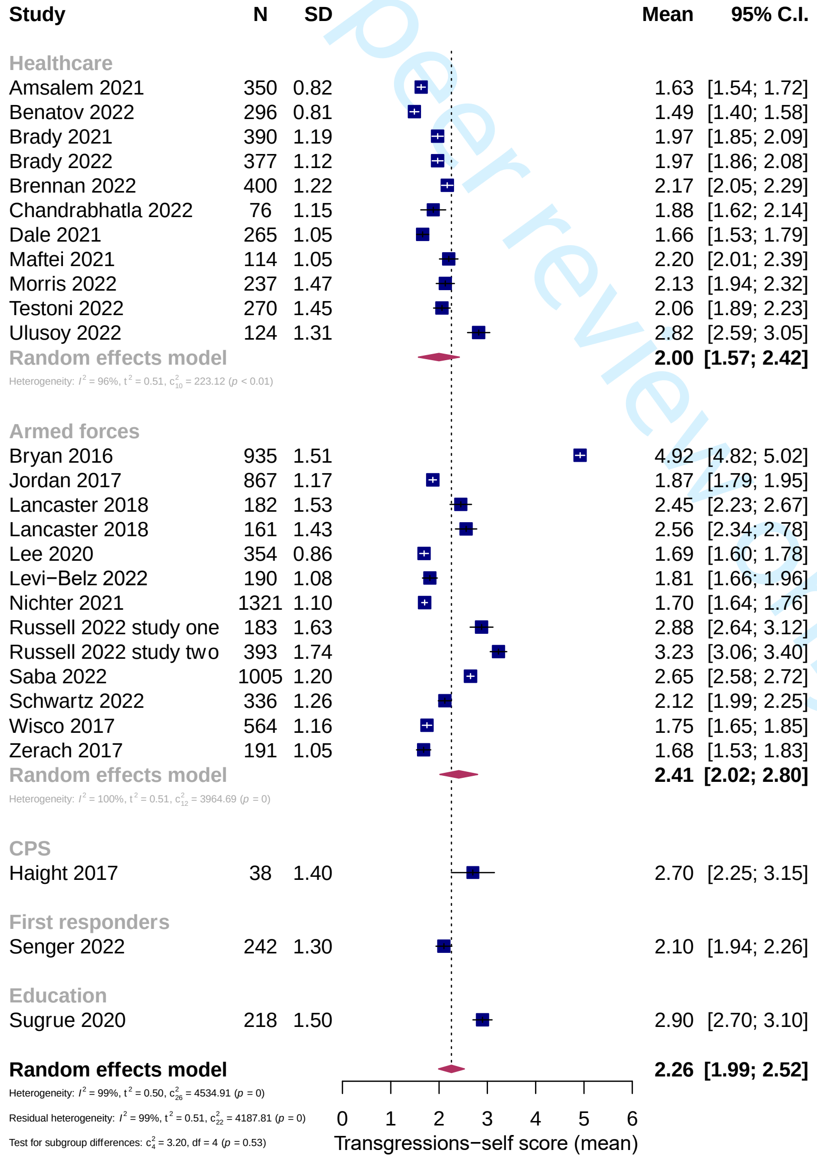

For peer review only

Supplementary Figure 14.

MIES transgressions-others pooled mean estimates, split by occupation.

Study	N	SD	Mean	95% C.I.
**Healthcare**				
Amsalem 2021	350	10.40	2.41	[1.32; 3.50]
Benatov 2022	296	2.71	4.08	[3.77; 4.39]
Brady 2021	390	1.15	2.93	[2.82; 3.04]
Brady 2022	377	1.56	3.01	[2.85; 3.17]
Brennan 2022	400	1.48	2.98	[2.83; 3.13]
Chandrabhatla 2022	74	1.64	3.41	[3.04; 3.78]
Dale 2021	265	1.45	2.54	[2.37; 2.71]
Maftei 2021	114	1.26	3.06	[2.83; 3.29]
Morris 2022	237	1.86	3.79	[3.55; 4.03]
Testoni 2022	270	1.56	3.23	[3.04; 3.42]
Ulusoy 2022	124	1.34	4.51	[4.27; 4.75]
Random effects model			**3.29**	**[2.88; 3.71]**
Heterogeneity: $I^2 = 96\%$, $\tau^2 = 0.47$, $\chi^2_{10} = 264.93$ ($p < 0.01$)				
**Armed forces**				
Bryan 2016	935	1.67	4.32	[4.21; 4.43]
Lancaster 2018	182	1.50	3.49	[3.27; 3.71]
Lee 2020	354	1.04	2.04	[1.93; 2.15]
Levi−Belz 2022	190	1.49	2.41	[2.20; 2.62]
Nichter 2021	1321	1.60	2.50	[2.41; 2.59]
Russell 2022 study one	183	1.20	1.73	[1.56; 1.90]
Russell 2022 study two	393	1.42	2.05	[1.91; 2.19]
Saba 2022	1005	0.84	2.92	[2.87; 2.97]
Wisco 2017	564	1.66	2.71	[2.57; 2.85]
Zerach 2017	191	1.39	2.42	[2.22; 2.62]
Random effects model			**2.66**	**[2.23; 3.09]**
Heterogeneity: $I^2 = 99\%$, $\tau^2 = 0.47$, $\chi^2_{9} = 1352.72$ ($p < 0.01$)				
**CPS**				
Haight 2017	38	1.00	5.10	[4.78; 5.42]
**First responders**				
Senger 2022	242	1.40	3.80	[3.62; 3.98]
**Education**				
Sugrue 2020	218	1.50	4.40	[4.20; 4.60]
**Random effects model**			**3.16**	**[2.81; 3.52]**

Heterogeneity: $I^2 = 99\%$, $\tau^2 = 0.75$, $\chi^2_{23} = 2215.96$ ($p = 0$)

Residual heterogeneity: $I^2 = 99\%$, $\tau^2 = 0.47$, $\chi^2_{19} = 1617.66$ ($p = 0$)

Test for subgroup differences: $\chi^2_4 = 17.36$, df = 4 ($p < 0.01$)

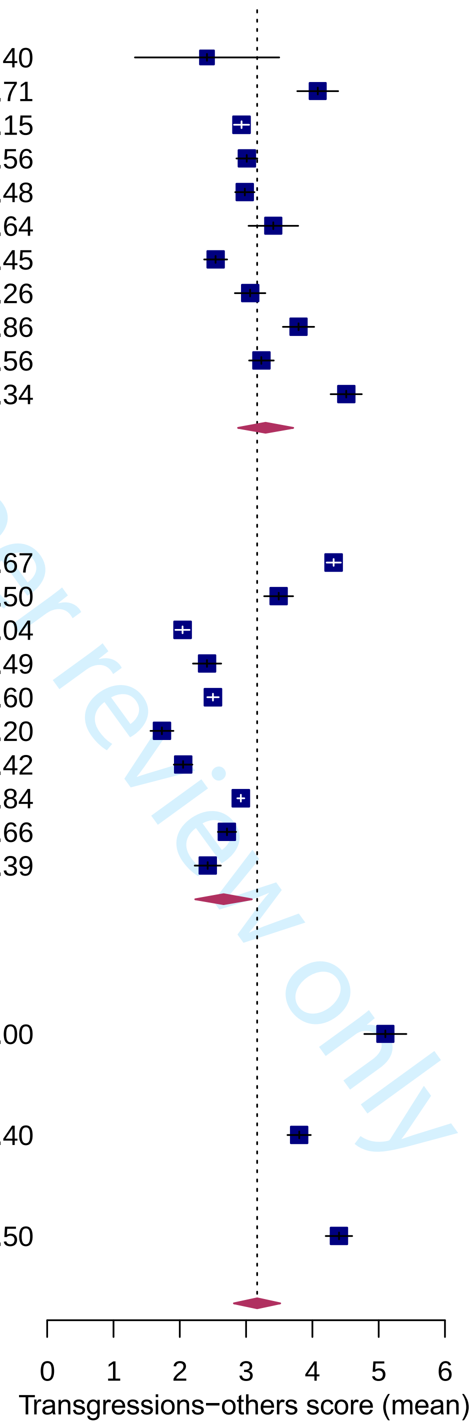

For peer review only

Supplementary Figure 15.
MIES betrayal pooled mean estimates, split by occupation.

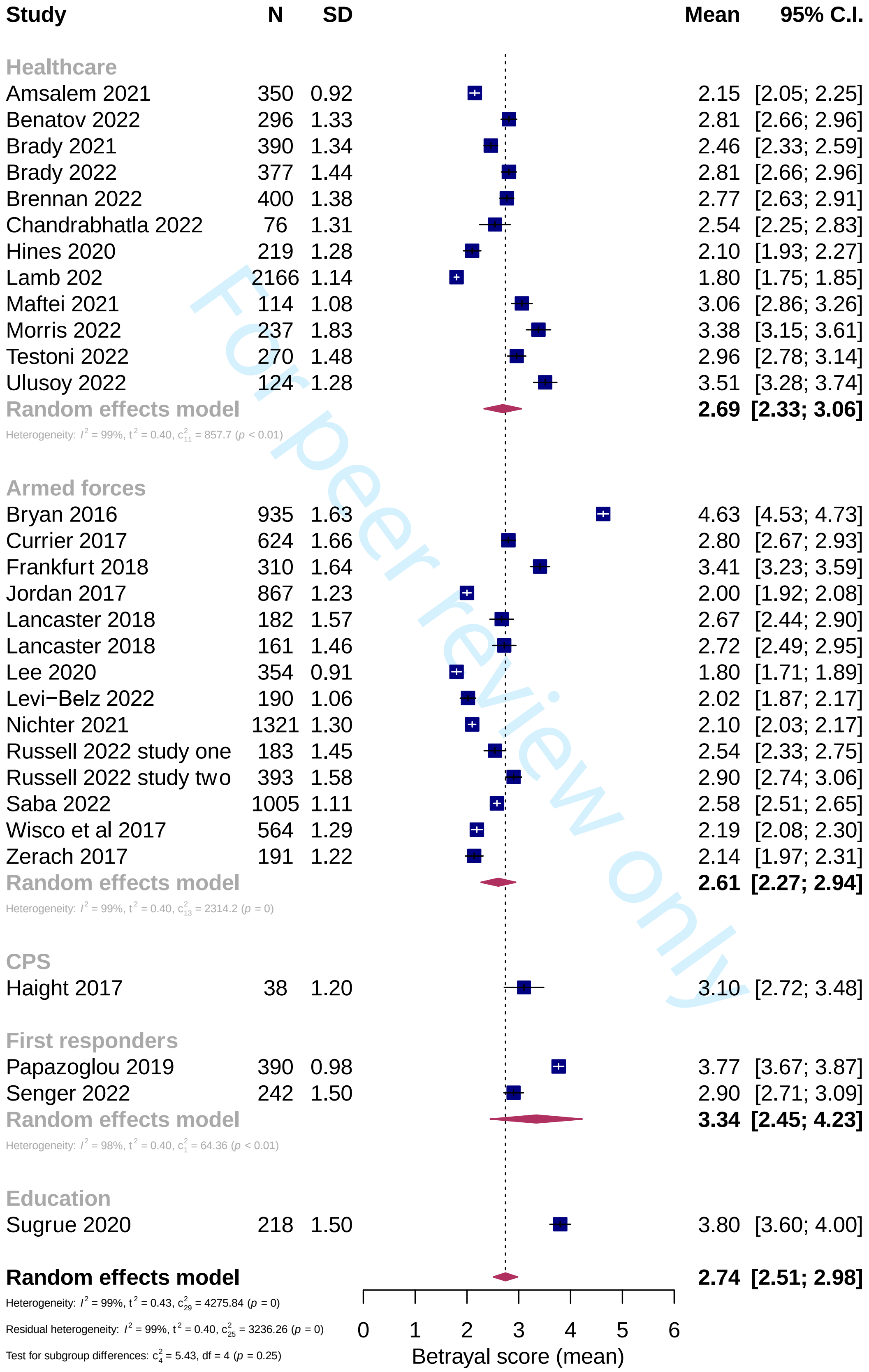

For peer review only

Supplementary Figure 16.
MIQ-M total pooled mean estimates.

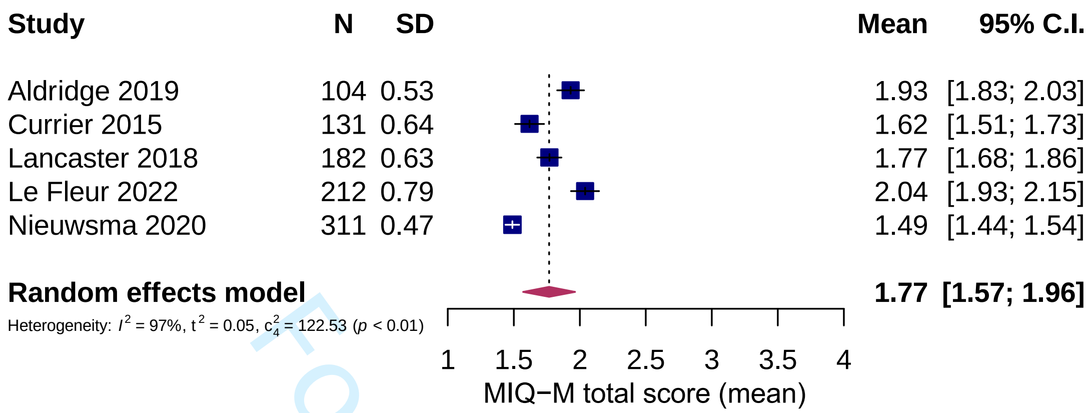

Supplementary Figure 17.
MIQ-M causes pooled mean estimates.

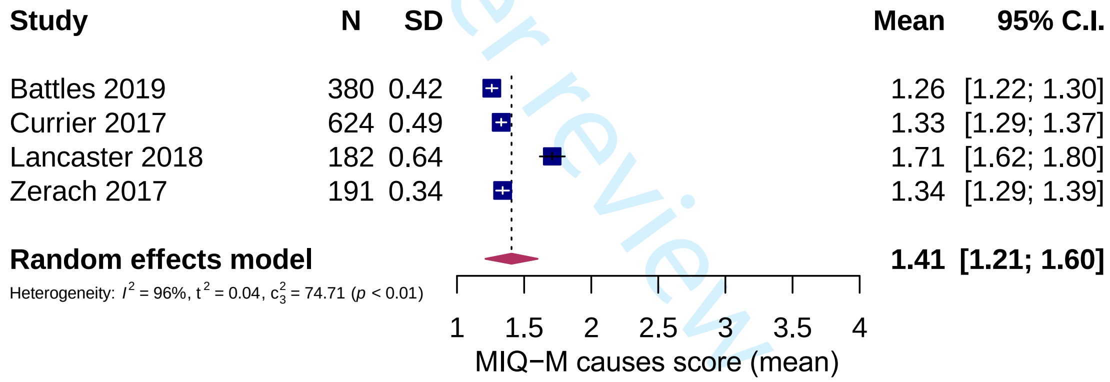

Supplementary Figure 18.
EMIS total pooled mean estimates.

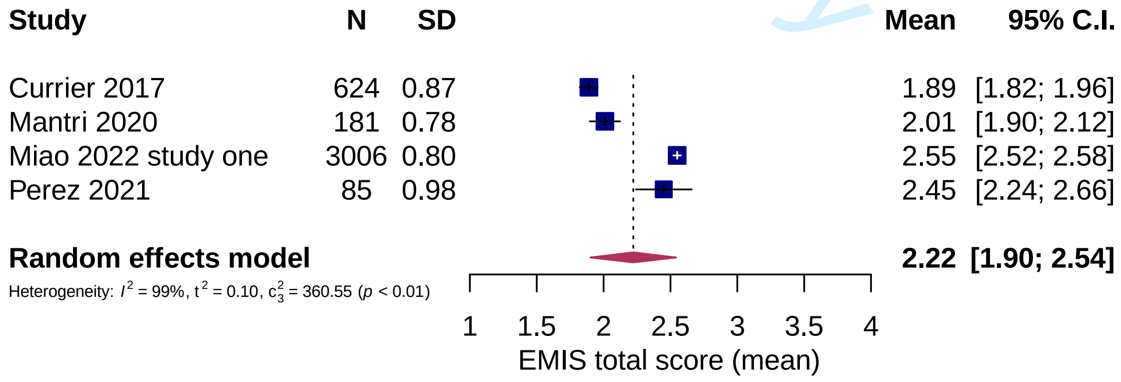

For peer review only - http://bmjopen.bmj.com/site/about/guidelines.xhtml

Supplementary Figure 19.
EMIS self pooled mean estimates.

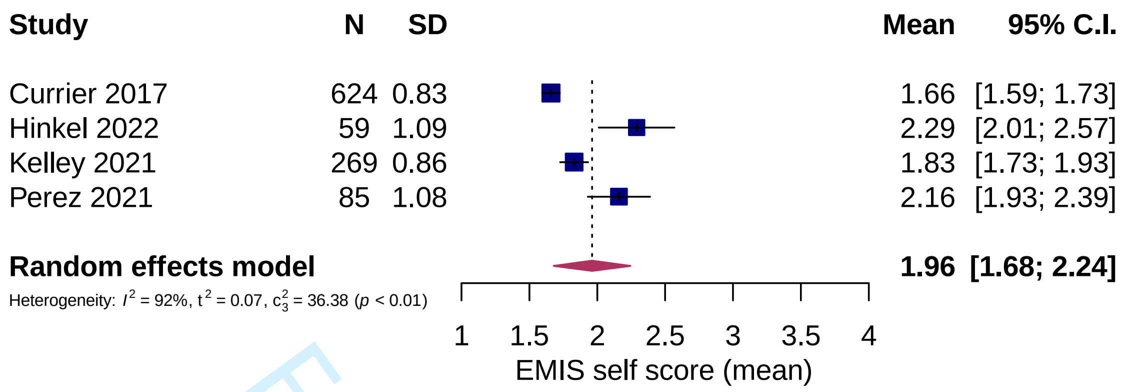

Supplementary Figure 20.
EMIS other pooled mean estimates.

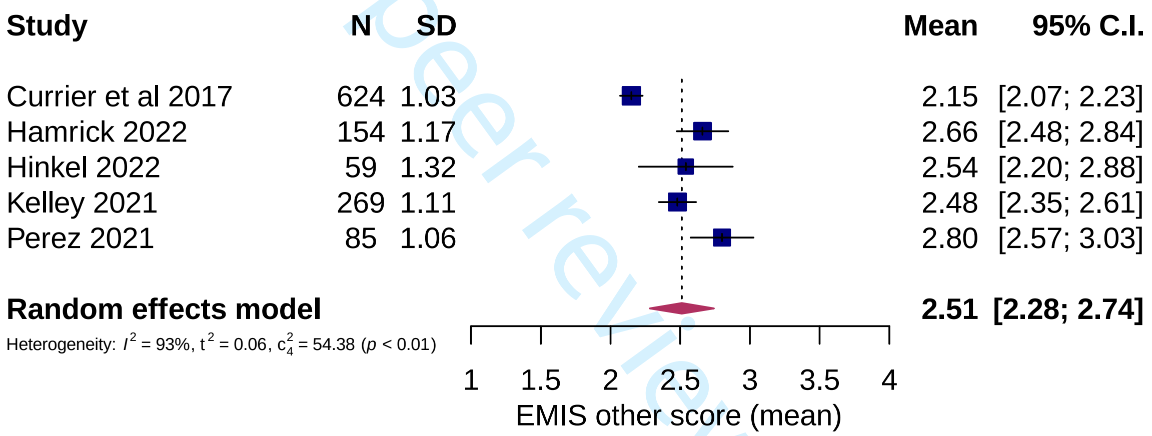

Supplementary Figure 21.
MISS-HP pooled mean estimates.

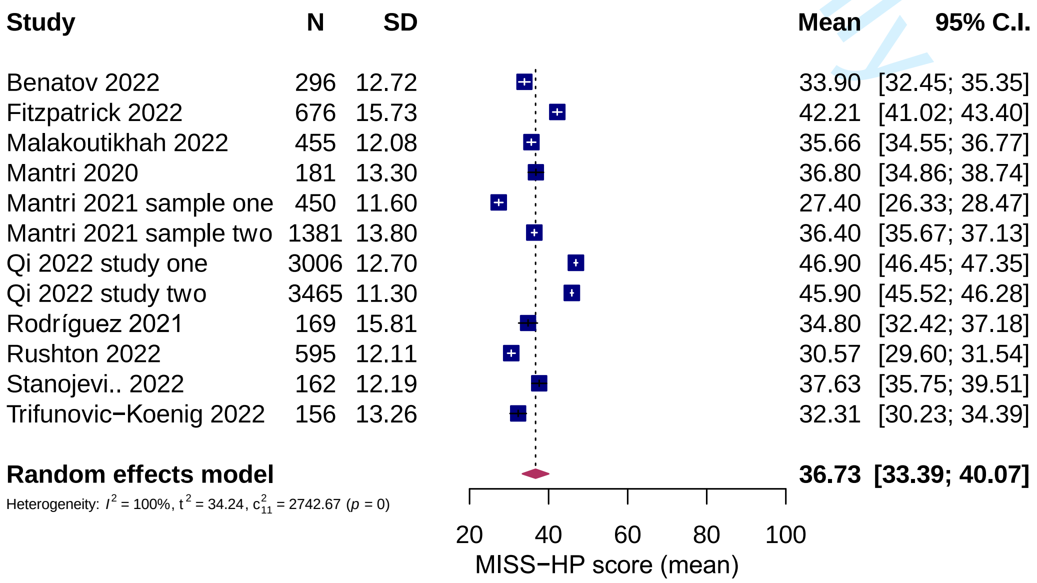

For peer review only

Supplementary Figure 22.
MIOS total pooled mean estimates.

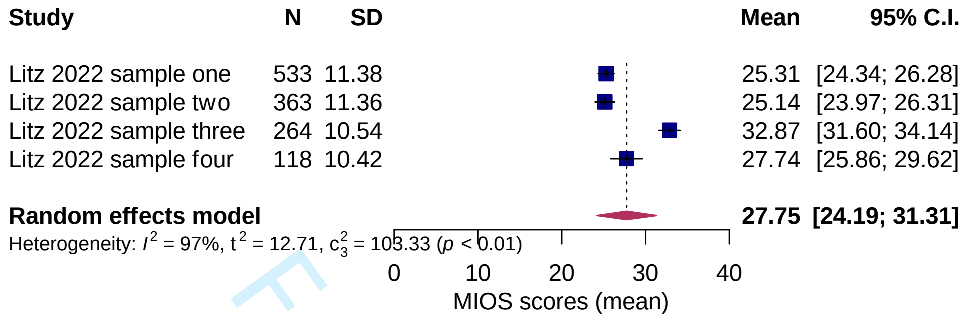

Supplementary Figure 23.
MIOS shame pooled mean estimates.

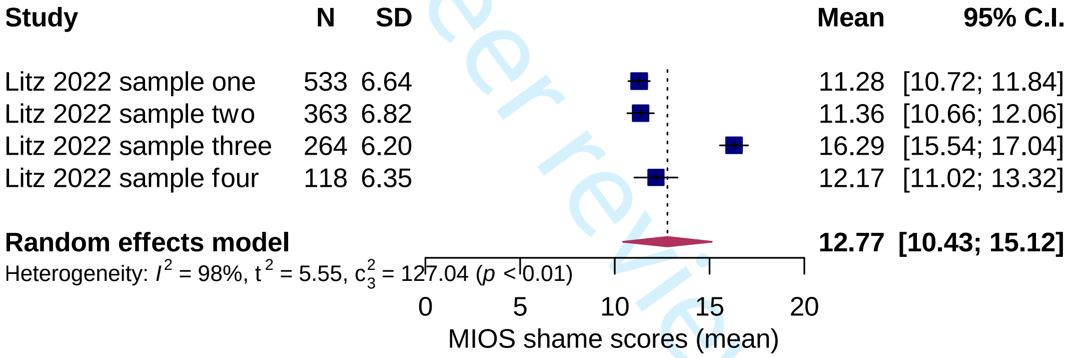

Supplementary Figure 24.
MIOS trust pooled mean estimates.

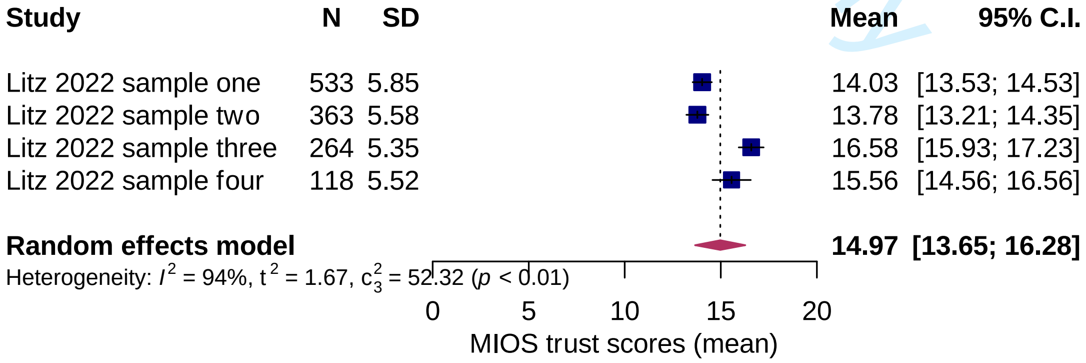

For peer review only

Supplementary Table 4.

Subgroup analyses based on study quality (mean and prevalence)

Measure	Medium risk			Low risk			
	k	Pooled mean [95% CI]	I^2	*k*	Pooled mean [95% CI]	I^2	P value
PTED	10	1.56 [1.11, 2.01]	99%	1	1.12 [1.03, 1.21]	-	0.566
MIES total	24	2.67 [2.42, 2.92]	99%	5	2.18 [1.62, 2.73]	97%	0.114
MIES transgressions-self	25	2.30 [2.02, 2.58]	99%	2	2.17 [0.75, 2.70]	0%	0.267
MIES transgressions-others	22	3.22 [2.85, 3.58]	99%	2	2.60 [1.40, 3.81]	85%	0.342
MIES betrayal	27	2.83 [2.60, 3.06]	99%	3	2.03 [1.33, 2.73]	97%	0.034
	k	Pooled prevalence (95% CI)	I^2	*k*	Pooled prevalence [95% CI]	I^2	P value
MIES	18	72.30 [64.83, 79.20]	97%	5	47.06 [32.42, 61.92]	99%	0.003
Transgressions-self	22	34.92 [28.32, 41.82]	99%	5	15.94 [84.7, 25.21]	98%	0.006
Transgressions-others	21	50.23 [39.64, 60.81]	98%	4	35.11 [14.67, 58.94]	99%	0.254
Betrayal	23	49.69 [43.09, 56.29]	97%	5	30.33 [18.51, 43.66]	99%	0.011

Note. PTED = post-traumatic embitterment disorder, MIES = Moral Injury Events Scale.

For peer review only

Supplementary Table 5.

Subgroup analyses based on cut off value (prevalence)

Measure	Slightly agree		Moderately agree		Strongly agree		Agree		NR[a]		
	k	Pooled %	*k*	Pooled %	*k*	Pooled %	*k*	Pooled %	*k*	Pooled %	P value
MIES overall	14	72.47 [63.07, 80.98]	6	54.13 [38.88, 69]		N/A	2	73.36 [47.45, 92.77]	1	49.72 [15.70, 83.88]	0.152
Transgressions -self	20	31.93 [24.52, 39.81]	3	22.06 [74.1, 41.63]	1	54.33 [20.26, 86.24]	2	28.04 [84.7, 53.41]	1	23.02 [1.68, 58.11]	0.578
Transgressions -others	18	52.22 [41.78, 62.56]	3	37.46 [15.33, 62.77]	1	38.82 [53.39, 80.92]	2	57.56 [27, 85.26]	1	02.26 [0, 31.56]	0.057
Betrayal	19	46.87 [38.98, 54.83]	6	37.54 [24.56, 51.49]	1	57.75 [24.89, 87.16]	1	58.24 [33.89, 80.65]		-	0.409

Measure	28.5		36		36.5		
	k	Pooled %	*k*	Pooled %	*k*	Pooled %	P value
MISS-HP	1	54.49 [46.61, 62.25]	8	44.17 [33.84, 54.77]	1	41.76 [37.26, 46.33]	0.802

Note. [a] For MIES any this was "sometimes". MIES = Moral Injury Events Scale; MISS-HP = Moral Injury Symptom Scale-Healthcare Professionals

For peer review only - http://bmjopen.bmj.com/site/about/guidelines.xhtml

For peer review only

Supplementary Table 6.
Prevalence and average scores for moral injury scales that were not combined for narrative synthesis (k=1)

Measure	Mean(SD)	Measure	Prevalence (%)
MIES-other (items 1-2, 7-9)	2.51 (1.40)	MIES-other (items 1-2, 7-9)	19.76%
MIES-commission (items 1-4)	1.71 (1.06)	MIES-commission (items 1-4)	14.28%
MIES-omission (items 5-6)	1.47 (1.1)	MIES-omission (items 5-6)	4.57%
MIQ-T	1.16 (0.56)	MIES-no moral distress	42%
MIQ-M modified		MIES-moral distress-other	19%
MIQ-M modified AoW	1.77 (0.63)	MIES-witnessing-only	16%
MIQ-M modified PCoW	1.99 (0.66)	MIES-moral distress-self	8%
MIQ-M modified LFB	2.12 (0.78)	MIES-moral distress-self and other	15%
MIQ-M modified AoW defining characteristics	2.21 (0.67)	EMIS-M self-directed ≥2.5 score <3.5	10.14%
MIQ-M modified PCoW defining characteristics	2.14 (0.66)	EMIS-M other-directed ≥2.5 score <3.5	29.19%
MIQ-M modified LFB defining characteristics	2.12 (0.67)		
BMIS total	3.71 (4.38)	BMIS event	44.1%
MIA-PSP total	59.6 (21.9)		
MIA-PSP emotional sequalae	25 (9.7)		
MIA-PSP perpetration	15 (8.5)		
MIA-PSP betrayal	19.6 (7.7)		
Toronto total	1.5 (1.27)		
Toronto organisation	1.54 (1.32)		
Toronto individual	1.18 (1.19)		
Toronto online	1.68 (1.27)		

Note. MI = moral injury; MIES = Moral Injury Events Scale; EMIS-M = Expressions of Moral Injury Scale-Military Version; MIA-PSP = Moral Injury Assessment for Public Safety Personnel; MIQ-M = Moral Injury Questionnaire—Military version; BMIS = Brief Moral Injury Screen; MIQ-T = Moral Injury Questionnaire—Teacher version; AoW = atrocities of war; LFB = leadership failure or betrayal; PCoW = psychological consequences of war

For peer review only - http://bmjopen.bmj.com/site/about/guidelines.xhtml

For peer review only

For peer review only - http://bmjopen.bmj.com/site/about/guidelines.xhtml
