# Supplementary material for: Prevalence of occupational moral injury and post-traumatic embitterment disorder: a systematic review and meta-analysis
Source: BMJ Open. 2024 Feb 20;14(2):e071776. doi: 10.1136/bmjopen-2023-071776 (PMC10882372; doi:10.1136/bmjopen-2023-071776)
Supplement: Reviewer comments [file bmjopen-2023-071776.reviewer_comments.pdf]

PEER REVIEW HISTORY

BMJ Open publishes all reviews undertaken for accepted manuscripts. Reviewers are asked to complete a checklist review form (**http://bmjopen.bmj.com/site/about/resources/checklist.pdf**) and are provided with free text boxes to elaborate on their assessment. These free text comments are reproduced below.

ARTICLE DETAILS

**TITLE (PROVISIONAL)**	A systematic review and meta-analysis on the prevalence of occupational moral injury and post-traumatic embitterment disorder
**AUTHORS**	Brennan, Chloe; Roberts, Carl; Cole, Jon

VERSION 1 – REVIEW

**REVIEWER**	Mentnech, Tisha<br>North Carolina State University at Raleigh
**REVIEW RETURNED**	09-Mar-2023

**GENERAL COMMENTS**	Full transparency. I am a librarian trained in evidence synthesis. I am not able to speak on the statistical / mathematical work. I've attached my edits and notes in a document. I also checked all your reference and as of 9 March none have been retracted or updated with an errata. I am happy to talk more and elaborate on any of my comments.

**REVIEWER**	Bath, Jennifer<br>Carilion Clinic, Trauma Services
**REVIEW RETURNED**	24-Apr-2023

**GENERAL COMMENTS**	Overall, this is a great article. Having just complete a study looking at moral distress and moral injury, I know there is not much in the literature. I have not seen much on PTED either. I would really like to see your article published. Congratulations on completing a meta-analysis, no small undertaking!<br><br>1. It is not recommended to use and/or, so pick one.<br>2. Spacing between end of a paragraph and subtitle, should it be multiple lines or just double spaced.<br>3. Review headers and sub-headers to make sure they follow the correct format.<br>4.Why are there "supplementary" tables? Do they provide data that is relevant to your hypothesis? If so, they should just be tables and included within the article.<br>5.Review thoroughly for verb tense. I mention some here but there were several places verb tense goes between present and past tense.<br>6. Watch punctuation, should be a comma before an 'and', 'but', 'or', and 'so'.<br>7. Review how your statistics are reported. Need to clean them up.<br>8. Determine what tables will be in article. Do supplementary tables add to the article? if so, the should not be supplementary.<br>Abstract Missing periods lines 24, 29, 33, 38<br>Article

	Article citation in text should be superscripted.<br>Page 4-line 3 does (mental) need to be in parentheses?<br>page 5- Line 8 PTSD and line 24 PTED need to spell out abbreviations the first time you use them.<br>Line 33 fix tense should be "increased and became..."<br>Line 33 sentence starting with Research should be made into 2 sentences. Period after event as work-related. Research has shown PTED...<br>page 6 line 8 is this referring to an appendix, table, reference? Not clear<br>Line 15 make 2 sentences period after teacher new sentence starting with Since the outbreak...<br>Line 54 remove and just disorders, so only some moral....<br>Page 7 line 12 should be "exists" and "establishes."<br>line 26 remove the current and change to This and should be aims to<br>Page 8 line 36 is this referring to an appendix, table, reference? Not clear<br>line 40 I believe you are saying that these studies were included so you need ot mention that if that is the case.<br>line 49 Start sentence with When not where.<br>page 9 line 5 need a comma after developed,<br>Line 31 what is (year) after author in parentheses? not clear here, are you citing a source?<br>page 10-line 22 need "the" before meta-analysis<br>line 33 need a space between cut-off ><br>line 38 (i.e., see table 2...) remove i.e., should just be see table 2), also need to add "a" before separate,<br>Page 11 line 56 remove as a and replace with The and add therefore after literature.<br>page 12 table 1 is presented here but is first mentioned on page 8. Should move table closer to its citation for ease of reference for the reader.<br>Line 33 mentions Figure 1. There is no figure 1 in the paper. On page 48 in the appendices there is an unlabeled PRISMA flow chart. If it is meant to be in the article, move it into the main text and add "figure 1" with a label saying PRISMA flow chart so readers know what the figure is for.<br>Line 38 needs a comma before so<br>line 40 remove "and" then add a comma before so and a "they" after so (pooled, so they were...).<br>Line 54 you mention cross-sectional studies, what were the remaining 23%?<br>Line 59 spell out one-digit numbers unless reporting statistics (3 should be three) also need to spell out UK-first time using this abbreviation.<br>page 13 line 10 need to spell out US-first time using this abbreviation.<br>Line 12 HCSW same as above, spell out.<br>line 17 CPS same, spell out.<br>Line 33, 35 and 38 spell out 5, 2, and 4<br>Lines 40 and 45 need reference citation for the scales<br>Line 47 add comma after specific, and also after there (specific, and there was also variation...).<br>Page 16 95% CI not reported correctly for example line 12 should read ...37% with a 95% CI (24.4%, 49.9%). Use the format 95% CI [LL, UL]<br>Line 36 end of line missing the 95% CI before the 39.5%<br>Page 17-line 10 missing comma before though first responders<br>line 12 change education to educators. Stay consistent in your

	labeling. You use educators earlier.<br>line 24 need a comma after betrayal, before but<br>line 47 MIQ-M, EMIS, MIOS first time mentioning these tools need to spell out abbreviations.<br>line 54 remove the before CPS.<br>Page 18-line 22 data in the () what area is this specific too? I would spell out for example the .... was 43%....<br>line 26 change i.e., in text to for example, use i.e., in parentheses only.<br>Page 20 line 53 i.e., should be inside the parentheses.<br>page 21-line 6 spell out PSP first time abbreviation used.<br>line 36 spell out 2 both times<br>page 22 line 8 add comma after affect and line 10 add comma after well-being.<br>page 23-line 8 period after PTED start new sentence The results show, change display to displayed (variations in verb tenses)<br>line 10 change show to showed.<br>line 24 comma after healthcare professionals<br>lines 26 comma after MISS-HP<br>line 33 remove the in front of CPS.<br>line 47 add which after PTED.<br>page 26-line 17 comma after MISS-HP.<br>line 33 remove the in front of CPS.<br>line 49 remove importantly start sentence with This.<br>line 54 add comma after studies.<br>line 56 add comma after Youssef.<br>Line 58 add comma after MIO),<br>page 27 line 6 add comma after ...MISS-HP), and add while after the comma HP), while many.<br>line 31 add comma after sickness, absences,<br>line 36 remove comma after conditions.<br>line 40 add comma after gap.<br>line 50 remove al after education.<br>Page 28 No conclusion.

**REVIEWER**	Carter, Claire<br>Independent
**REVIEW RETURNED**	05-Jun-2023

**GENERAL COMMENTS**	Thank you for asking me to review this paper on the prevalence of occupational Moral Injury (MI) and PTED, which is an area of growing interest. This review is unable to fully assess the validity of the author's methodology and statistical data. This review has a focus on content relating to MI and PTED.<br><br>The studies included in this research did not use the same design and models. Therefore, the pooled prevalence and outcomes may be unsubstantiated when using this method. A specialist statistical review may clarify the appropriateness of the methodology and the results. A comment on the limitations of this method and reliability of the results in the conclusion would perhaps benefit the reader.<br><br>The first research aim is to determine the prevalence of occupational moral injury and PTED. The results state exposure to MI across occupations is 67% and prevalence of PTED across occupations is 26%. Thereby achieving the first aim.<br><br>The second aim of the research was to establish whether prevalence estimates differ depending on occupation. The authors determine Moral Injury-'other,' is more prevalent in civilian

	occupations, compared with the military. However, Identifying which civilian occupations are high, moderate and low risk of exposure and of developing clinically significant MI and PTED, would perhaps be of greater value. I am of the opinion this research aim has not been sufficiently achieved. Yet, this may indicate that methodological differences between the studies used, prevents achieving this analysis, which may answer research aim (iii) to establish whether methodological differences between studies influence prevalence estimates.<br><br>Research aim iv) to record the variables associated with occupational moral injury and PTED is not fully met. The infrequency of the measurement of variables in the studies, renders the findings inconclusive. Highlighting this limitation in the conclusion may be beneficial to the reader.<br><br>There is no proven or recommended treatment for MI or PTED. However, trials for treatment are underway (such as Wisdom Therapy and Adaptive Disclosure). I suggest, identifying the prevalence of these conditions across occupations could demonstrate the need for the development of successful treatment. Organisations will need guidance and strategies for supporting employees if PTED or MI are high risk factors in their workplace. Does this research highlight the need for a standardised measure and treatment options across occupations? Perhaps, the authors would consider explaining why their research is important and who the beneficiaries may be.<br><br>The statement, "findings show civilian occupations are at greater risk of exposure to morally injurious events," is perhaps too generic when consideration is given to the fact that the studies relate to statutory organisations and frontline services, rather than civilian occupations more generally. For example roles more likely to expose employees to trauma and moral transgressions, such as healthcare, first responders, humanitarian relief, crown prosecution service, education system and social services. The research may benefit from making this distinction. Further research comparing MI and PTED between high, moderate and low risk civilian occupations would be valuable.<br><br>Suggestions for further research based on these findings would also be helpful. I would welcome a revised version of this research paper, with a more rounded conclusion for the benefit of the reader and to better showcase the valuable efforts of the authors to highlight a need for further research on the prevalence of occupational MI and PTED across a variety of low, moderate and high risk civilian occupations.<br><br>The authors reach the conclusion after analysing largely quantitative data that "Exposure to morally injurious events, moral injury symptoms, and PTED are prevalent across work settings and exposure to transgressions by others and betrayal events are more likely in civilian occupations."<br><br>In conclusion, I am of the opinion the research aims are partially met and the conclusion would benefit from including which aims were achieved and reasons why others are not, or a caveat that the results are tentative due to the variance in the studies used. Meta-analysis may not be appropriate for assessing the varied constructs

	of MI, where standardised measures are absent. I would suggest the authors explain why their research is important. Currently, I am of the opinion that this research does not add value to the study of MI and PTED, without a revision.

**REVIEWER**	Jenniskens, Kevin<br>Universitair Medisch Centrum Utrecht, Julius Center
**REVIEW RETURNED**	14-Jul-2023

**GENERAL COMMENTS**	I would like to compliment the researchers on a tremendous amount of work that has been put forward. Although I am not an expert in the clinical field, I can image that this overview of prevalences of moral injury and PTED, and whether there are specific subgroups that are affected to a more or lesser extend, is of great value and a nice starting point for future (clinical) studies.<br><br>However there is some room for improvement in my opinion, and as such I have provided some comments below for your consideration<br><br>General comments<br>• The abstract has hardly any methods in it. Without this methods section and the quality appraisal, I would not accept this manuscript<br>• You can really question whether the pooled prevalence is really relevant. I would focus more on the subgroups, and see whether there are any correlations between those.<br>• Statistically, your data allow for a more rigorous assessment. Meta-regression with study level covariates would fit nicely here, as you can statistically identify specific subgroups, and show certainty and magnitude of associations.<br>• Quality assessment is inherently subjective, hence having have performed it by only one individual makes the results unreliable (regardless of expertise of the researcher in question). I would recommend assessing quality of all included studies by two researchers, and resolving discrepancies<br>• I don't agree with your final conclusion that more high quality large scale studies on prevalence of PTED and moral injury are needed. You did not have any high risk studies, you have 88 included studies, and you did subgroup analysis on risk of bias in a sufficiently large sample. Enough is enough.<br>Rather focus on what we can do with this prevalence information; how can we direct other future research (such as trials, prediction research, priority setting) using this prevalence information.<br><br>Comments<br>• Abstract:<br>o Elaborate more on the methods. How did you search, in which databases, what time period, data that were extracted, any quality appraisal, how meta-analysis was performed<br>o Setting should be more specific<br>o I personally have no idea what constitutes as "moral injury"<br>o Narrative synthesis? What is this?<br><br>• Introduction:<br>o Can be a bit more concise, prevent elaborating on aspects that are not assessed in the rest of the review. Focus on the magnitude of the problem, what research has been done (i.e. reviews), and why this review is necessary<br>o Last paragraph: state the domain of your study (from the results I think it is important that it reflect civilian and military occupations)<br>o Recording and establishing seem very descriptive. Did you really

	assess (statistical) associations? Merely extracting<br><br>• Methods:<br>o I would make a heading for study selection, place eligibility criteria there, and move to data extraction from there<br><br>• Results:<br>o You can argue whether pooling heterogeneous studies for all meta-analyses is warranted. The pooled estimate is meaningless when there is not only random heterogeneity, but other sources of heterogeneity as well (i.e. high I2)<br>o So is this Test for subgroup differences: c 2/4 = … Cochrane's Q? Not sure what this measure in your forest plots is.<br>o You can write point estimates and intervals a bit more concise. So this (43%, 95% CI 36% to 49.5%) becomes 43% (36.0-49.5%) to increase readability<br>o What is the difference between these norms and prevalences? I am not sure how to interpret the norms, so perhaps provide some definition in the introduction or methods section. EDIT: from the discussion section I get these are mean or average scores. Why not just say that? (and define whether average is the arithmetic average or something else)<br>o Subgroup high risk of bias: all were either low or medium right?<br><br>• Discussion:<br>o "showed more practically relevant levels (>2)" What do you mean here?<br>o I would be interested in the main prevalence of PTED, moral injury, and what subgroups were predominantly identified. Try focusing first on the main take-away, and in the subsequent paragraph on additional details.<br>o "Whilst heterogeneity is often high in prevalence meta-analyses, and the median I2 was found to be 96.9% in a review of 134 prevalence meta-analyses[114], caution must be taken when interpreting our findings." The pooled estimates, but not so much the individual study results, which can still be viewed in the forest plots.<br>o You could assess the year in which studies were conducted as a covariate to see whether estimates have changed over time (see reference 37 for an example)<br>o Can you clarify what you mean by nationally representative? For the general working population?<br>o Two times the word finally in the last paragraph in the discussion

VERSION 1 – AUTHOR RESPONSE

Reviewer: 1
Prof. Tisha Mentnech, North Carolina State University at Raleigh

Comments to the Author:
Full transparency. I am a librarian trained in evidence synthesis. I am not able to speak on the statistical / mathematical work. I've attached my edits and notes in a document. I also checked all your reference and as of 9 March none have been retracted or updated with an errata. I am happy to talk more and elaborate on any of my comments.

Thank you for your suggestions to make the paper more reproducible and more likely to be discovered. Please see our response to your comments below.

We have cited the protocol and the R package "meta", spelt the "CoCoPop" mnemonic in full and listed the full search strategy for each database in the supplementary materials. We accessed the databases via the University of Liverpool webpage and have discussed the version and interface with our librarian. The APA PsycInfo interface is EBSCOhost though they cannot provide a "version" as it's a constantly updated resource. Similarly, for Sage Journals Online, the interface is Sage's own platform https://journals.sagepub.com/ and again there is no version number as it is constantly updated. The version of WoS is Core Collection and we have added the above information to the manuscript and full search strategy in the supplementary materials. We have also added to the search strategy section to explain search terms were free text so the same key words could be replicated in each databse as opposed to using subject headings which can differ between databases.

We have added inclusion of articles written in English in the eligibility section too. We have also changed the PPI statement from passive to active voice and included the occupations in the abstract to make the SR more discoverable so thanks for the tip. To ensure we have explained the methods used for our narrative review, in the method section we have outlined the method for conducting narrative review i.e., "Associates were grouped into demographic, mental health, and individual/work variables, and the percentage of studies that reported each type of variable was calculated. Vote counting was used to determine whether the overall relationship between variables with PTED and moral injury was positive, negative or no association." In addition, in the data extraction table, there in a column called "Associations" which lists each variable measures in association with MI or PTED and whether it is a (+) significant positive correlation, (-) significant negative correlation or (0) not significant.

Finally, whilst it would be ideal to have more than two screeners, we have followed guidance from the Cochrane handbook (4.6.4) which states you must use at least two people working independently to determine whether studies meet the eligibility criteria at the full text stage. However, we have also ensured two screeners are used at the title/abstract stage for 15% of papers. To address this comment, in the strengths/limitations section of the discussion, we have explained that having three screeners would have been more objective.

Reviewer: 2
Dr. Jennifer Bath, Carilion Clinic
Comments to the Author:
Overall, this is a great article. Having just complete a study looking at moral distress and moral injury, I know there is not much in the literature. I have not seen much on PTED either. I would really like to see your article published. Congratulations on completing a meta-analysis, no small undertaking!

Thank you for your review and positive comments.

1. It is not recommended to use and/or, so pick one.

We have changed all instances of and/or.

2. Spacing between end of a paragraph and subtitle, should it be multiple lines or just double spaced.

This spacing has been deleted now.

3. Review headers and sub-headers to make sure they follow the correct format.

The headers within the abstract have been changed (in line with the editor's request) and headings within the methods section have changed in line with reviewer 4's requests. Hopefully these changes have also addressed your comment.

4.Why are there "supplementary" tables? Do they provide data that is relevant to your hypothesis? If so, they should just be tables and included within the article.

In line with BMJ Open's guidelines for authors we have included less than five tables and figures in the main paper and have ensured some material is separated into supplementary files to make the main manuscript clearer for readers. Within the supplementary materials we have included the search strategy, the risk of bias and data extraction table of all included studies, and forest plots. Additional tables include subgroup analyses based on study quality and cut off value, and the prevalence and average scores for scales that were not able to be combined. Within the main paper, we have discussed the important features of these tables and refer readers to the supplementary information for more detailed information.

5.Review thoroughly for verb tense. I mention some here but there were several places verb tense goes between present and past tense.

We have reviewed verb tense throughout and thanks for spotting errors.

6. Watch punctuation, should be a comma before an 'and', 'but', 'or', and 'so'.

We have reviewed punctuation throughout.

7. Review how your statistics are reported. Need to clean them up.

We have cleaned up how statistics are reported by ensuring they all use the same number of decimal places and have changed how we reported CI's.

8. Determine what tables will be in article. Do supplementary tables add to the article? if so, the should not be supplementary.

Please see response to comment 4. All supplementary tables are discussed within the main text of the paper.

Abstract Missing periods lines 24, 29, 33, 38

Article

Article citation in text should be superscripted.

Citations have been superscripted now.

Page 4-line 3 does (mental) need to be in parentheses?

The parentheses have been removed throughout.

page 5- Line 8 PTSD and line 24 PTED need to spell out abbreviations the first time you use them.

Fixed.

Line 33 fix tense should be "increased and became..."

Fixed.

Line 33 sentence starting with Research should be made into 2 sentences. Period after event as work-related. Research has shown PTED...

Fixed.

page 6 line 8 is this referring to an appendix, table, reference? Not clear

This was the data extraction table and we have now changed this to supplementary table 2.

Line 15 make 2 sentences period after teacher new sentence starting with Since the outbreak...

Fixed.

Line 54 remove and just disorders, so only some moral....

Fixed.

Page 7 line 12 should be "exists" and "establishes."

Fixed.

line 26 remove the current and change to This and should be aims to

Fixed.

Page 8 line 36 is this referring to an appendix, table, reference? Not clear

This was the data extraction table and we have now changed this to supplementary table 2.

line 40 I believe you are saying that these studies were included so you need to mention that if that is the case.

Fixed.

line 49 Start sentence with When not where.

Fixed.

page 9 line 5 need a comma after developed,

Fixed.

Line 31 what is (year) after author in parentheses? not clear here, are you citing a source?

This is the year published, we have changed this now.

page 10-line 22 need "the" before meta-analysis

Fixed.

line 33 need a space between cut-off >

Fixed.

line 38 (i.e., see table 2...) remove i.e., should just be see table 2), also need to add "a" before separate,

We have removed i.e., and added “a” now.

Page 11 line 56 remove as a and replace with The and add therefore after literature.

This sentence has been changed from passive to active voice in line with reviewer 1’s comment.

page 12 table 1 is presented here but is first mentioned on page 8. Should move table closer to its citation for ease of reference for the reader.

Table 1 is now immediately after the paragraph with the in-text citation.

Line 33 mentions Figure 1. There is no figure 1 in the paper. On page 48 in the appendices there is

an unlabelled PRISMA flow chart. If it is meant to be in the article, move it into the main text and add "figure 1" with a label saying PRISMA flow chart so readers know what the figure is for.

BMJ Open author guidelines require authors to upload the figure as a separate file rather than embedded in the manuscript with the title at the end of the manuscript. Therefore, the PRISMA flow chart has been uploaded separately and the following title is at the end of the manuscript "Figure 1. Preferred Reporting Items for Systematic Reviews and Meta-Analyses (PRISMA) flow diagram."

Line 38 needs a comma before so

Fixed.

line 40 remove "and" then add a comma before so and a "they" after so (pooled, so they were...).

Fixed.

Line 54 you mention cross-sectional studies, what were the remaining 23%?

The remaining are longitudinal, and this has been added in now.

Line 59 spell out one-digit numbers unless reporting statistics (3 should be three) also need to spell out UK-first time using this abbreviation.

Fixed.

page 13 line 10 need to spell out US-first time using this abbreviation.

Fixed.

Line 12 HCSW same as above, spell out.

Fixed.

line 17 CPS same, spell out.

Fixed.

Line 33, 35 and 38 spell out 5, 2, and 4

Fixed.

Lines 40 and 45 need reference citation for the scales

Added in.

Line 47 add comma after specific, and also after there (specific, and there was also variation...).

Fixed.

Page 16 95% CI not reported correctly for example line 12 should read ...37% with a 95% CI (24.4%, 49.9%). Use the format 95% CI [LL, UL]

Changed format throughout.

Line 36 end of line missing the 95% CI before the 39.5%

Fixed.

Page 17-line 10 missing comma before though first responders

Fixed.

line 12 change education to educators. Stay consistent in your labeling. You use educators earlier.

Fixed.

line 24 need a comma after betrayal, before but

Fixed.

line 47 MIQ-M, EMIS, MIOS first time mentioning these tools need to spell out abbreviations.

Fixed.

line 54 remove the before CPS.

Fixed.

Page 18-line 22 data in the () what area is this specific too? I would spell out for example the .... was 43%....

We have now specified in healthcare professionals and removed 43% from the brackets.

line 26 change i.e., in text to for example, use i.e., in parentheses only.

Changed to for example.

Page 20 line 53 i.e., should be inside the parentheses.

Fixed.

page 21-line 6 spell out PSP first time abbreviation used.

Fixed.

line 36 spell out 2 both times

Fixed.

page 22 line 8 add comma after affect and line 10 add comma after well-being.

Fixed.

page 23-line 8 period after PTED start new sentence The results show, change display to displayed (variations in verb tenses)

Fixed.

line 10 change show to showed.

Fixed.

line 24 comma after healthcare professionals

Fixed.

lines 26 comma after MISS-HP

Fixed.

line 33 remove the in front of CPS.

Fixed.

line 47 add which after PTED.

Fixed.

page 26-line 17 comma after MISS-HP.

Fixed.

line 33 remove the in front of CPS.

Fixed.

line 49 remove importantly start sentence with This.

Fixed.

line 54 add comma after studies.

Fixed.

line 56 add comma after Youssef.

Fixed.

Line 58 add comma after MIO),

Fixed.

page 27 line 6 add comma after ...MISS-HP), and add while after the comma HP), while many.

Fixed.

line 31 add comma after sickness, absences,

It is sickness absences and we have added a comma.

line 36 remove comma after conditions.

Fixed.

line 40 add comma after gap.

Fixed.

line 50 remove al after education.

Fixed.

Page 28 No conclusion.

We have added a conclusion paragraph.

Reviewer: 3
Dr. Claire Carter, Independent

Comments to the Author:
Thank you for asking me to review this paper on the prevalence of occupational Moral Injury (MI) and PTED, which is an area of growing interest. This review is unable to fully assess the validity of the author's methodology and statistical data. This review has a focus on content relating to MI and PTED.

Thank you for taking the time to review this paper.

The studies included in this research did not use the same design and models. Therefore, the pooled prevalence and outcomes may be unsubstantiated when using this method. A specialist statistical review may clarify the appropriateness of the methodology and the results. A comment on the limitations of this method and reliability of the results in the conclusion would perhaps benefit the reader.

Due to studies using different models, we have pooled prevalence separately for each scale/subscale. We have outlined this limitation in several more places throughout the discussion so hopefully this limitation is clearer, and we have also added this to the conclusion.

The first research aim is to determine the prevalence of occupational moral injury and PTED. The results state exposure to MI across occupations is 67% and prevalence of PTED across occupations is 26%. Thereby achieving the first aim. The second aim of the research was to establish whether prevalence estimates differ depending on occupation. The authors determine Moral Injury-'other,' is more prevalent in civilian occupations, compared with the military. However, Identifying which civilian occupations are high, moderate and low risk of exposure and of developing clinically significant MI and PTED, would perhaps be of greater value. I am of the opinion this research aim has not been sufficiently achieved. Yet, this may indicate that methodological differences between the studies used, prevents achieving this analysis, which may answer research aim (iii) to establish whether methodological differences between studies influence prevalence estimates.

We agree that we are only partially able to determine whether prevalence estimates differ based on occupation since we can only conduct subgroup analyses of occupation on the MIES as all other moral injury scales are occupation specific. We have highlighted which aims have been fully met or only partially met and why at the beginning of the discussion when we describe each of the results.

We have highlighted which civilian occupations are at a higher risk (i.e., healthcare, educators, CPS) in the opening paragraph of the discussion and in the ‘prevalence meta-analyses’ section of the results. We have also added this to the implications/future research paragraph and explained the need for a wider range of professions to determine low, moderate, and high-risk occupations.

Research aim iv) to record the variables associated with occupational moral injury and PTED is not fully met. The infrequency of the measurement of variables in the studies, renders the findings inconclusive. Highlighting this limitation in the conclusion may be beneficial to the reader.

At the end of the second paragraph of the discussion we have highlighted that these findings are inconclusive.

There is no proven or recommended treatment for MI or PTED. However, trials for treatment are underway (such as Wisdom Therapy and Adaptive Disclosure). I suggest, identifying the prevalence of these conditions across occupations could demonstrate the need for the development of successful treatment. Organisations will need guidance and strategies for supporting employees if PTED or MI are high risk factors in their workplace. Does this research highlight the need for a standardised measure and treatment options across occupations? Perhaps, the authors would consider explaining why their research is important and who the beneficiaries may be.

We have outlined this issue toward the end of the implications/gaps paragraph and in the conclusion paragraph in the discussion now.

The statement, "findings show civilian occupations are at greater risk of exposure to morally injurious events," is perhaps too generic when consideration is given to the fact that the studies relate to statutory organisations and frontline services, rather than civilian occupations more generally.
For example roles more likely to expose employees to trauma and moral transgressions, such as healthcare, first responders, humanitarian relief, crown prosecution service, education system and social services. The research may benefit from making this distinction. Further research comparing MI and PTED between high, moderate and low risk civilian occupations would be valuable.

We have added to the implications and future research paragraph to clarify which occupations are at a higher risk (i.e., healthcare, educators, CPS) and explained that we need more research with a wider range of professions to determine low, moderate, and high-risk occupations.

Suggestions for further research based on these findings would also be helpful. I would welcome a revised version of this research paper, with a more rounded conclusion for the benefit of the reader and to better showcase the valuable efforts of the authors to highlight a need for further research on the prevalence of occupational MI and PTED across a variety of low, moderate and high risk civilian occupations.

We have added a conclusion which highlights the need for more research across low moderate and high-risk occupations, and the need for proven treatments and guidance for organisations.

The authors reach the conclusion after analysing largely quantitative data that "Exposure to morally injurious events, moral injury symptoms, and PTED are prevalent across work settings and exposure to transgressions by others and betrayal events are more likely in civilian occupations."

In conclusion, I am of the opinion the research aims are partially met and the conclusion would benefit from including which aims were achieved and reasons why others are not, or a caveat that the results are tentative due to the variance in the studies used. Meta-analysis may not be appropriate for assessing the varied constructs of MI, where standardised measures are absent. I would suggest the authors explain why their research is important. Currently, I am of the opinion that this research does not add value to the study of MI and PTED, without a revision.

In the first and second paragraph of the discussion we have added which aims have/have not been fully met and explained that some results are inconclusive. In the strengths/limitations paragraph we discuss the varied constructs of MI. We have also added this to the implications paragraph and have suggested gold standard measurements are needed. Finally, we have repeated the need for more research, with more consistent measurements to strengthen the reliability of the findings in the conclusion.

Reviewer: 4
Dr. Kevin Jenniskens, Universitair Medisch Centrum Utrecht

Comments to the Author:
I would like to compliment the researchers on a tremendous amount of work that has been put forward. Although I am not an expert in the clinical field, I can image that this overview of prevalences of moral injury and PTED, and whether there are specific subgroups that are affected to a more or lesser extend, is of great value and a nice starting point for future (clinical) studies. However there is some room for improvement in my opinion, and as such I have provided some comments below for your consideration

Thank you for your positive comments, and your suggestions to improve the paper.

General comments.

• The abstract has hardly any methods in it. Without this methods section and the quality appraisal, I would not accept this manuscript.

We have added to the abstract to include more methods and a second researcher has reviewed 100% of the quality appraisal now.

• You can really question whether the pooled prevalence is really relevant. I would focus more on the subgroups, and see whether there are any correlations between those.

To meet the first aim of our review (i.e., pooled prevalence) we have still reported this information. However, at several points in the discussion we have better indicated which subgroups had higher/lower prevalence rates.

• Statistically, your data allow for a more rigorous assessment. Meta-regression with study level covariates would fit nicely here, as you can statistically identify specific subgroups, and show certainty and magnitude of associations.

Whilst we appreciate the suggestion to use meta-regression, we have tried to remain consistent with our published Prospero protocol and have used subgroup analyses due the categorical nature of occupation. Also, there are only between 2 and 30 studies in each of our separate meta-analyses and the latest version of the Cochrane Handbook suggests a minimum of 10 studies per examined covariate in meta-regression. Due to the number of occupations, there would not be enough studies per occupation to do these analyses.

• Quality assessment is inherently subjective, hence having have performed it by only one individual makes the results unreliable (regardless of expertise of the researcher in question). I would recommend assessing quality of all included studies by two researchers, and resolving discrepancies

We agree that this would ensure our results are more rigorous and now two individuals have performed QA. This has been added to the manuscript.

• I don't agree with your final conclusion that more high quality large scale studies on prevalence of PTED and moral injury are needed. You did not have any high risk studies, you have 88 included studies, and you did subgroup analysis on risk of bias in a sufficiently large sample. Enough is enough.

We have removed this final conclusion.

Rather focus on what we can do with this prevalence information; how can we direct other future research (such as trials, prediction research, priority setting) using this prevalence information.

In the implications and conclusion paragraphs we have now explained how this prevalence information can inform guidance and policy makers on which areas of research/prevention to prioritise.

Comments
• Abstract:

o Elaborate more on the methods. How did you search, in which databases, what time period, data that were extracted, any quality appraisal, how meta-analysis was performed

We have included more information on the databases and time period of searches, data extraction, quality appraisal, that how the meta-analysis was performed.

o Setting should be more specific

Setting has been removed now in line with the editor's suggestions, but we have included "in any geographical location" as this was the eligibility criteria.

o I personally have no idea what constitutes as "moral injury"

We have explained that moral injury and post-traumatic embitterment disorder (PTED) are two constructs which describe the psychological distress caused by exposure to unjust and morally traumatic events in the abstract now.

o Narrative synthesis? What is this?

We have explained this better now i.e., results that could not be pooled have been summarised qualitatively in a narrative synthesis using the Guidance for Systematic Reviews.

• Introduction:

o Can be a bit more concise, prevent elaborating on aspects that are not assessed in the rest of the review. Focus on the magnitude of the problem, what research has been done (i.e. reviews), and why this review is necessary

We have changed the introduction to try and avoid discussing anything not directly relevant, and to focus more of the magnitude of the problem, the reviews so far and why this meta-analysis is needed. We have also discussed the variability in measuring moral injury and PTED as this relates to our third research aim (i.e., whether methodological differences influence prevalence estimates).

o Last paragraph: state the domain of your study (from the results I think it is important that it reflect civilian and military occupations)

In the final aims paragraph, we have specified this is in military and civilian occupations.

o Recording and establishing seem very descriptive. Did you really assess (statistical) associations? Merely extracting

Yes, we agree it is a descriptive method due to the infrequency of variables measured and because it was a secondary aim. Therefore, we have simply extracted the information, and used vote counting if there were enough studies (>3) to report an overall positive, negative or no association. This data is summarised qualitatively in a narrative synthesis. We have outlined the full method in the final paragraph of the method section.

• Methods:
o I would make a heading for study selection, place eligibility criteria there, and move to data extraction from there

We have now created a heading for search strategy and study selection where we have included information on the search strategy, study selection and eligibility criteria, then moved into the data extraction heading.

• Results:
o You can argue whether pooling heterogeneous studies for all meta-analyses is warranted. The pooled estimate is meaningless when there is not only random heterogeneity, but other sources of heterogeneity as well (i.e. high I2)

We agree that the heterogeneity in the included studies are high, which is an important limitation that we have highlighted several more times in the discussion now and is a key takeaway for future research i.e., we need more standardised ways of measuring MI/PTED in the future.

o So is this Test for subgroup differences: c 2/4 = … Cochrane's Q? Not sure what this measure in your forest plots is.

Yes, this is the Cochrane's Q value.

o You can write point estimates and intervals a bit more concise. So this (43%, 95% CI 36% to 49.5%) becomes 43% (36.0-49.5%) to increase readability

We have changed the CI estimates in line with this and reviewer 2's comments.

o What is the difference between these norms and prevalences? I am not sure how to interpret the norms, so perhaps provide some definition in the introduction or methods section. EDIT: from the discussion section I get these are mean or average scores. Why not just say that? (and define whether average is the arithmetic average or something else)

In the methods section, under the 'data extraction' heading, we have outlined that this is average scores (Mean and SD) and throughout we have referred to norms as average scores instead now.

o Subgroup high risk of bias: all were either low or medium right?

Yes, thank you for spotting this typo. We have corrected this now.

• Discussion:
o "showed more practically relevant levels (>2)" What do you mean here?

We have now changed the wording to "The results showed that across occupations, 37% of staff scored above a differentiation point for elevated embitterment (>1.6), 26% had clinically relevant embitterment (>2), and 11% showed embitterment of a clinically significant intensity with improved clinical practicability and specificity (>2.5)." Hopefully, this provides a better explanation.

o I would be interested in the main prevalence of PTED, moral injury, and what subgroups were predominantly identified. Try focusing first on the main take-away, and in the subsequent paragraph on additional details.

The first paragraph now only focuses on the key take-away (aim 1 and 2) which reports the prevalence of moral injury and PTED, and differences between occupations. The second paragraph outlines additional details regarding aims 3 and 4.

o "Whilst heterogeneity is often high in prevalence meta-analyses, and the median I2 was found to be 96.9% in a review of 134 prevalence meta-analyses[114], caution must be taken when interpreting our findings." The pooled estimates, but not so much the individual study results, which can still be viewed in the forest plots.

We have added to the sentence above to explain that there was high heterogeneity within individual studies too.

o You could assess the year in which studies were conducted as a covariate to see whether estimates have changed over time (see reference 37 for an example)

We published our intended analyses in our pre-registered protocol on Prospero and we did not intend to investigate study year as a covariate so would prefer not to deviate. Also, given the relative recency of all included studies i.e., all have been published from 2014 onwards and most in the last few years we do not believe this will add to the manuscript at this point. However, if (and when) there is an updated meta-analysis on this topic in the future it would become more relevant.

o Can you clarify what you mean by nationally representative? For the general working population?

Yes, the general working population, and also in specific occupations of interest i.e., the armed forces, HSCWs, first responders, educators, journalists, CPS employees since many studies with these groups were not randomly selected representative samples. We have added detail to reflect this now.

o Two times the word finally in the last paragraph in the discussion

Thanks for spotting this, we have changed this so finally only appears once now.

VERSION 2 – REVIEW

**REVIEWER**	Carter, Claire<br>Independent
**REVIEW RETURNED**	20-Oct-2023

**GENERAL COMMENTS**	Thank you for asking me to review this major revision, which has made a significant difference to the reader's experience, as well as understanding of the subject matter and demonstrating the value of the research. In my opinion the authors have succeeded in writing a well-rounded paper of interest, which invites further thought and study. I would like to make suggestions to improve a few of the statements, which are not essential changes.<br><br>[Neither PTED, nor Moral Injury are categorised in current diagnostic manuals, such as the Diagnostic and Statistical Manual of Mental Disorders, version 5 (DSM-5) or International Classification of Diseases, version 11 (ICD-11)]. "Moral Injury and post-traumatic embitterment disorder (PTED) are two concepts that have emerged in the literature to explain the harm caused by exposure to unjust and potentially morally injurious events (PMIEs)" (p.5). [PTED and Moral Injury can have a disabling impact on individuals, such as social isolation, difficulty with relationships and difficulty maintaining employment. Organisations may experience higher rates of sickness absence and ill health retirement in employees. Embitterment can lead to protracted litigation against employers.]<br><br>https://link.springer.com/book/10.1007/978-3-211-99741-3<br><br>Frontiers | Defining and Assessing the Syndrome of Moral Injury: Initial Findings of the Moral Injury Outcome Scale Consortium (frontiersin.org)<br><br>"Individuals may also experience morally traumatic events at work, through interpersonal conflicts, making challenging ethical decisions, and exposure to events that contradict what [the person perceives to be] right" (p.5). It would be helpful to clarify that Moral Injury and posttraumatic embitterment are subjective experiences, dependent on the individual's own perception, values, beliefs and morals, with this small change to the statement.<br><br>Discussion<br><br>I am of the opinion that the statement, "It is possible that military personnel expect to be exposed to moral traumas and are less troubled as a result," (p.31) should be removed or sharpened. For example, "It is possible that military training is a protective factor and armed forces are less troubled by betrayal trauma as a result." Other occupations, such as first responders, social care workers and child protection services expect exposure to significant moral trauma too. Shay states team cohesion, expert ethical leadership and training in the military may be protective factors. This suggests that differences in organisational leadership and working practices, may affect risk of exposure to moral trauma and may explain (in part) the reason for different outcomes between civilian and military occupations. Does the culture within the military support greater team cohesion, trust in leaders and peers, compared with civilian organisational culture?<br>https://summerstudy.yale.edu/sites/default/files/jonathan_shay._moral_injury.pdf<br><br>More research is needed within civilian occupations and I hope the authors will be in a position to continue their studies in this field. Particularly as the

	development of a 'gold standard' measurement for Moral Injury is underway through a consortium of researchers specialising in military Moral Injury. They state, "our results should be replicated with other samples, particularly among civilians."<br>Frontiers | Defining and Assessing the Syndrome of Moral Injury: Initial Findings of the Moral Injury Outcome Scale Consortium (frontiersin.org)

**REVIEWER**	Jenniskens, Kevin<br>Universitair Medisch Centrum Utrecht, Julius Center
**REVIEW RETURNED**	30-Oct-2023

**GENERAL COMMENTS**	I am satisfied with the revisions made by the authors on the manuscript. What a tremendous amount of work it must have been, my compliments!<br><br>Two small revision suggestions:<br>1. In the abstract it states "... by exposure to unjust at work.". Should this not be "unjust and morally traumatic events" as stated in the rebuttal to the reviewers? Or perhaps injustice to keep it short?<br><br>2. the objective in the abstract is not really an objective (unless being the first meta-analysis is the objective in and of itself). I would rephrase this to the overall aim as described in the last paragraph of the introduction.

VERSION 2 – AUTHOR RESPONSE

Reviewer: 3

Dr. Claire Carter, Independent

Comments to the Author:
Thank you for asking me to review this major revision, which has made a significant difference to the reader's experience, as well as understanding of the subject matter and demonstrating the value of the research. In my opinion the authors have succeeded in writing a well-rounded paper of interest, which invites further thought and study. I would like to make suggestions to improve a few of the statements, which are not essential changes.

Thank you.

[Neither PTED, nor Moral Injury are categorised in current diagnostic manuals, such as the Diagnostic and Statistical Manual of Mental Disorders, version 5 (DSM-5) or International Classification of Diseases, version 11 (ICD-11)]. "Moral Injury and post-traumatic embitterment disorder (PTED) are two concepts that have emerged in the literature to explain the harm caused by exposure to unjust and potentially morally injurious events (PMIEs)" (p.5). [PTED and Moral Injury can have a disabling impact on individuals, such as social isolation, difficulty with relationships and difficulty maintaining employment. Organisations may experience higher rates of sickness absence and ill health retirement in employees. Embitterment can lead to protracted litigation against employers.]

https://link.springer.com/book/10.1007/978-3-211-99741-3

Frontiers | Defining and Assessing the Syndrome of Moral Injury: Initial Findings of the Moral Injury

Outcome Scale Consortium (frontiersin.org)

We have now added this information.

“Individuals may also experience morally traumatic events at work, through interpersonal conflicts, making challenging ethical decisions, and exposure to events that contradict what [the person perceives to be] right” (p.5). It would be helpful to clarify that Moral Injury and posttraumatic embitterment are subjective experiences, dependent on the individual’s own perception, values, beliefs and morals, with this small change to the statement.

We have now changed this statement.

Discussion

I am of the opinion that the statement, “It is possible that military personnel expect to be exposed to moral traumas and are less troubled as a result,” (p.31) should be removed or sharpened. For example, “It is possible that military training is a protective factor and armed forces are less troubled by betrayal trauma as a result.” Other occupations, such as first responders, social care workers and child protection services expect exposure to significant moral trauma too. Shay states team cohesion, expert ethical leadership and training in the military may be protective factors. This suggests that differences in organisational leadership and working practices, may affect risk of exposure to moral trauma and may explain (in part) the reason for different outcomes between civilian and military occupations. Does the culture within the military support greater team cohesion, trust in leaders and peers, compared with civilian organisational culture?
https://summerstudy.yale.edu/sites/default/files/jonathan_shay._moral_injury.pdf

We have now sharpened this statement in line with your suggestion.

More research is needed within civilian occupations and I hope the authors will be in a position to continue their studies in this field. Particularly as the development of a ‘gold standard’ measurement for Moral Injury is underway through a consortium of researchers specialising in military Moral Injury. They state, “our results should be replicated with other samples, particularly among civilians.”
Frontiers | Defining and Assessing the Syndrome of Moral Injury: Initial Findings of the Moral Injury Outcome Scale Consortium (frontiersin.org)

Reviewer: 4
Dr. Kevin Jenniskens, Universitair Medisch Centrum Utrecht
Comments to the Author:
I am satisfied with the revisions made by the authors on the manuscript. What a tremendous amount of work it must have been, my compliments!

Thank you.

Two small revision suggestions:
1. In the abstract it states "... by exposure to unjust at work.". Should this not be "unjust and morally traumatic events" as stated in the rebuttal to the reviewers? Or perhaps injustice to keep it short?

We have changed this to “injustice”.

2. the objective in the abstract is not really an objective (unless being the first meta-analysis is the objective in and of itself). I would rephrase this to the overall aim as described in the last paragraph of the introduction.

We have now changed the objective to “This meta-analysis aims to determine the prevalence of occupational moral injury and PTED and establish whether prevalence estimates differ depending on occupation.” We have not included the full aims as described in the last paragraph of the introduction due to the 300-word limit.
